# Silence in Shamatha, Transcendental, and Stillness Meditation: An Evidence Synthesis Based on Expert Texts

**DOI:** 10.3389/fpsyg.2020.01259

**Published:** 2020-07-08

**Authors:** Toby J. Woods, Jennifer M. Windt, Olivia Carter

**Affiliations:** ^1^Melbourne School of Psychological Sciences, The University of Melbourne, Melbourne, VIC, Australia; ^2^Department of Philosophy, Monash University, Melbourne, VIC, Australia

**Keywords:** traditional accounts, shamatha, transcendental meditation, stillness meditation, contentless experience, pure consciousness, phenomenology, database

## Abstract

Shamatha, Transcendental, and Stillness Meditation are said to aim for “contentless” experiences, where mental content such as thoughts, perceptions, and mental images is absent. Silence is understood to be a central feature of those experiences. The main source of information about the experiences is texts by experts from within the three traditions. Previous research has tended not to use an explicit scientific method for selecting and reviewing expert texts on meditation. We have identified evidence synthesis as a robust and transparent method that is suitable for this purpose. In this paper we use evidence synthesis based on expert texts to examine silence/quietness as a feature of the contentless experiences in the three practices. Objective criteria were used to select a sample of 135 expert texts. A database containing the expert descriptions of the meditation techniques and experiences was produced by extracting the relevant material from the publications and coding that material to differentiate individual features. The database, which forms part of the Supplementary Material for this paper, identifies each feature of the contentless experiences referred to in the expert texts, including silence/quietness. Our key finding is that the experts indicate silence/quietness has a particular connection with stillness, and the absence of concepts, mental activity/noise, thoughts, and disturbance. Further analysis leads to the following insights. The silence/quietness reflects the absence of thoughts and sounds, and this fits neatly with a conception of silence/quietness as the absence of internal and external noise. In some cases the terms silence and quietness may also reflect the absence of other disturbances such as non-auditory perceptions, mental images, and negative feelings. That would fit with a conception of silence/quietness as complete calm or absence of disturbance. It is not clear from the expert texts how silence/quietness is distinct from other features such as stillness that also reflect the absence of disturbances. As a separate matter, silence/quietness has connections with all the other features of the contentless experiences, but the closeness of the connections varies. Our work uncovers fine distinctions and ambiguities which lead to new research questions that can be explored in future studies.

## Introduction

Numerous meditation practices are understood to aim for experiences that are “contentless,” in that mental content such as thoughts, sense-perceptions, body-perceptions, and mental images is absent (Stace, 1960/1961; Forman, 1990, 1998; Shear, 2006d)^1^. Contentless experiences are often treated as *pure consciousness*, or consciousness itself (Stace, 1960/1961; Forman, 1990, 1999; Shear, 1990b; Fasching, 2008), and are therefore of great interest in cognitive science and philosophy (Millière et al., 2018; Metzinger, 2020). In this paper we will concentrate on three of the practices: the shamatha meditation focused on by Alan Wallace (“Shamatha”) (Wallace, 2006a), Transcendental Meditation (“TM”) (Maharishi Mahesh Yogi, 1963/2001, 1967/1974), and Stillness Meditation (Meares, 1967/1968; McKinnon, 1983/2016).

Shamatha is a Tibetan Buddhist practice^2^. The Shamatha meditator cultivates attention on a meditation object, and then at a very advanced stage releases the object and enters the contentless experience (Wallace, 2006a). According to TM experts, the TM technique formed part of the Vedic tradition of ancient India (Shear, 2006c, pp. 23–24; Pearson, 2013, p. 397; Roth, 2018, p. 38; cf. Williamson, 2010, p. 86). Maharishi Mahesh Yogi is said to have revived the technique in the 1950s, distilling it from the wider set of traditional Vedic practices and understandings (Shear, 2006c, pp. 24, 47; Rosenthal, 2011/2012, p. 4; Pearson, 2013, pp. 28, 44, 397, 437). The TM meditator repeats a mantra silently in their mind, and this is said to initiate a movement toward and into the contentless experience where awareness of the mantra is lost (Faber et al., 2017). Stillness Meditation was developed by an Australian psychiatrist, Ainslie Meares, in the 1960s (Meares, 1967/1968; McKinnon, 1983/2016). The Stillness Meditation practitioner gives up the effort of *doing* anything, beyond maintaining the meditation posture, and this is said to lead to the contentless experience.

Shamatha and TM have been the subject of major research programs (e.g., Jacobs et al., 2011; Pearson, 2013, pp. 399–430, Rosenberg et al., 2015; Zanesco et al., 2019) and are therefore obvious candidates for further investigation. Stillness Meditation, in contrast, is not well known, and has received little research attention (Seymour, 1999; Hosemans, 2017). Our interest in Stillness Meditation derives from the experience of one of the authors (TW), who has practised it for over 20 years. We have included it as one of the practices that we are focusing on because it has a detailed literature on the contentless experience, and because it does not involve a meditation object, making it quite different to Shamatha and TM.

The contentless experiences aimed for in the three practices are referred to as experiences of silence or stillness. Wallace (2011b, p. 120) describes the “sheer silence, the stillness” in Shamatha. Travis et al. (2005, p. 128) quote a meditator who remarks of their TM practice, “I am completely still. It’s absolute silence.” Stillness Meditation is named as such because of the experience of stillness that is said to emerge. The experience is described as a “silent stillness” (Meares, 1984, p. 46) and as “only stillness … an all-encompassing silence” (McKinnon, 2011, p. 44).

Since thoughts and sounds are said to be absent, contentless experiences make an excellent subject for researchers interested in silence. Furthermore, since silence is central to the experiences, understanding the specific feature silence is essential for understanding each experience as a whole.

There are very few participant-based studies of the contentless experiences in Shamatha, TM or Stillness Meditation (e.g., Seymour, 1999; Travis and Pearson, 2000; Hosemans, 2017). The major source of information about the experiences is written accounts of experts from within each of the meditation traditions. We will refer to these as *expert texts*, *traditional publications*, or *traditional accounts*^3^. If researchers wish to understand meditation experiences, it is critical that they have regard to documents of this type (Lindahl et al., 2014, p. 1; Gyatso, 2016, p. ix).

The aim of this paper is to understand how silence is described in the expert texts^4^. How do meditators refer to a silence without thoughts and sounds? Does the silence purely reflect the absence of thoughts and sounds, or are there other facets that are important?

To the best of our knowledge, the paper is the first to examine the experience of silence in Shamatha, TM and Stillness Meditation based on traditional publications selected and reviewed using a scientific method. Since use of scientific method is a key contribution of our work, it will be helpful if we now explain why that approach is important in this area.

One challenge for researchers is that there is typically a large number of traditional publications for each form of meditation. For Stillness Meditation there are well over 50, and for Shamatha and TM there are likely thousands. Since it is rarely feasible for researchers to review all of the publications, they generally focus on a relatively small subset.

A limitation of the existing research is that it tends not to use an explicit scientific method for the selection or review of the traditional publications. This is understandable given that reliance on traditional accounts is still at a nascent stage, but it gives rise to the following problems.

If researchers wish to use an explicit scientific method, what should the method for selecting the traditional publications be? In other words, how can researchers select publications in a rigorous manner that will provide a strong understanding of how the meditation technique and experience is described within the tradition? It may be best for them to focus on high quality publications, but how should they decide which publications meet that standard? If they can decide, then which high quality publications should they look at first? Is there any point at which they can stop? And, if not, what if there are too many high quality publications to review?

There are further issues once the publications have been selected. How can the researchers review them in a rigorous manner? And how should they document the overall process to give confidence to the reader? Without an explicit scientific method, the reader can be rightly concerned that the researchers may have deliberately or inadvertently cherry-picked particular publications or points within them. Those publications and points may fail to properly capture key aspects of the technique and experience as understood within the tradition.

We have identified *evidence synthesis* as a method that can provide solutions to these problems. Evidence synthesis involves a structured process for selecting and reviewing the most relevant publications from a wider body of literature. A structured process is one that is rigorous and transparent, and addresses or tests a specific research question or hypothesis (Petticrew and Roberts, 2006; Pope et al., 2007; Briner and Denyer, 2012; Gough et al., 2012/2017; Bailey et al., 2015; Aromataris and Munn, 2017).

Use of a structured approach ensures that the process can be critiqued or audited, and that the findings are precise and reliable (Centre for Reviews and Dissemination, 2009; Booth et al., 2012/2016; Briner and Denyer, 2012). Pre-established guidelines and criteria are used to select the publications for review, and to extract from them and analyze the relevant material. As a result, there is much less scope for cherry-picking (Petticrew and Roberts, 2006; Booth et al., 2012/2016; Briner and Denyer, 2012).

Identifying evidence synthesis as an appropriate scientific method is only the first step toward establishing a detailed method for a particular research question. Researchers must next select the most suitable type of evidence synthesis from the wide variety that have been developed. Each type provides a particular framework for selecting and reviewing the publications. Once researchers have selected a suitable type, they must design detailed elements within the framework that are appropriate for the specific research question.

In this paper we use evidence synthesis based on traditional accounts to examine silence/quietness as a feature of the contentless experiences in Shamatha, TM and Stillness Meditation. The type of evidence synthesis and the detailed elements of that framework are described in the next section.

The paper is part of a broader project which we will refer to as the “Contentless Experience Project” or the “current project.” The project employs a distinction between the “goal-states” and the “interim-states” in the practices. The term goal-state refers to the subjective experience of a state aimed for in a practice. Since Shamatha, TM and Stillness Meditation aim for contentless experiences, in those practices the contentless experiences are the goal-states^5^. The term interim-state is used to describe an experience in a practice on the way to achieving the goal-state/s.

The Contentless Experience Project includes the present evidence synthesis and two additional evidence syntheses (Woods et al., 2020a, b). The present synthesis focuses on silence/quietness as a specific feature of the goal-states, and the other two syntheses will examine the meditation techniques, interim-states, and other features of the goal-states.

The three syntheses are based on a single database containing material extracted from the expert texts. The database includes the material on silence/quietness required for this paper, and material on the other features needed for the two additional syntheses. The material has been coded in the database to differentiate individual features.

The method described below principally concerns the production of the database. The main additional element is the explanation of how the database was used to compare the feature silence/quietness across the three practices. This paper presents the database and our findings concerning silence/quietness.

## Materials and Methods

### Use of Narrative Synthesis

The type of evidence synthesis that we selected for the current project was *narrative synthesis* (Popay et al., 2006), which is a form of *qualitative evidence synthesis*. Qualitative evidence synthesis involves the review of qualitative publications and provides options for the selection of texts where a comprehensive search is not feasible. That is the case for this project: For Shamatha and TM traditional publications likely number in the thousands, and for Shamatha they include texts dating back hundreds of years that are difficult to access outside Tibet and India. Another form of evidence synthesis, *systematic review*, requires comprehensive searching (Petticrew and Roberts, 2006; Pope et al., 2007), and is therefore not appropriate for the project.

Narrative synthesis is principally a textual, as distinct from quantitative or numerical, form of synthesis. It is distinguished from a *narrative review* – meaning a traditional literature review – on the basis that it is a structured approach, as described above (Mays et al., 2005a, b; Popay et al., 2006; Pope et al., 2007; Centre for Reviews and Dissemination, 2009).

The essential factor distinguishing narrative synthesis from other forms of qualitative evidence synthesis is that it is flexible (Popay et al., 2006; Madden et al., 2018). One aspect of that flexibility is that the reviewer can choose between a series of techniques identified by Popay et al. (2006), and then select particular forms of those techniques that are most appropriate for their project. The technique selected for the current project was thematic analysis (see section “Synthesis” below).

The decision to employ narrative synthesis rather than one of the more specialized qualitative evidence synthesis methods was made on the basis of fitness for purpose (Noyes and Lewin, 2011b; Noyes et al., 2015). A key factor was that, while many of the more specialized methods also utilize thematic analysis, they tend to use forms of it that are not appropriate for the objectives in the current project. For example, in *meta-aggregation* (Munn and Jordan, 2011; Lockwood et al., 2017; McArthur et al., 2017), *thematic synthesis* (Thomas and Harden, 2008), *meta-ethnography* (Noblit and Hare, 1988; Mays et al., 2005a; Britten and Pope, 2011/2012), and *grounded theory synthesis* (Whittemore et al., 2014; Bryant, 2017), once initial coding has been applied to material extracted from the selected publications, the process becomes about discerning higher level themes, which normally involve greater levels of abstraction. Identifying higher level themes was not the main objective in the current project. Coding was used to identify features of the practices within the extracted material (see section “Synthesis”)^6^. Those features could be broad (e.g., the general requirement to maintain a posture in a practice), or narrow (e.g., the specific requirement that the posture be symmetrical). The main objective was not to generate from the features higher level themes or groupings, but to explain how the experts refer to the features (whether broad or narrow) and to compare the features across the practices.

### Basic Components and Reporting

The narrative synthesis method comprises four basic components: (a) selection of the publications for review; (b) critical appraisal – assessing the quality of the publications; (c) data extraction – extracting relevant material; and (d) synthesis – coding that material and performing any additional analyses (Popay et al., 2006). In the current project, critical appraisal is dealt with as part of the selection of publications. That leaves three components (selection, extraction, synthesis), which are described below. These have been designed specifically to meet the research objectives, having regard to existing principles and frameworks for narrative synthesis and evidence synthesis more generally.

The project is compliant with the ENTREQ reporting guidelines for qualitative evidence synthesis (Tong et al., 2012). Supplementary Appendix A details the section/s of the present evidence synthesis that satisfy each of the 21 items on the ENTREQ checklist.

### Selection of Authors and Publications

Traditional accounts were selected using purposive sampling (Suri, 2011, 2014; Gough et al., 2012/2017; Patton, 2015). That involved the strategic identification of a sample of publications providing valuable insights into the practices. Purposive sampling was essential because comprehensive searching was not feasible (see above).

For each practice, the strategy for selecting publications involved two steps: (a) identifying a limited number of authors within the meditation tradition who have outstanding qualifications as experts in the practice and who write in clear English; and (b) identifying, by way of a structured process, samples of those authors’ publications that would reveal their understandings of the practice. Those steps are described in detail below.

The procedure constituted a form of critical appraisal, in that publications were only selected if the author had outstanding qualifications as an expert in the relevant practice (Greenhalgh et al., 2005; McArthur et al., 2017). The rationale for this approach was that such authors are able to provide higher quality descriptions of the practices than authors who are less qualified. Publications by less qualified authors were excluded.

#### Selection of Authors

Candidate authors were identified from scoping reading of selected publications of a broad range of authors, and from general background reading. From the list of candidate authors for each practice one or more authors with outstanding qualifications as an expert in the practice was identified. Assessment of whether an author has outstanding qualifications was made on the basis of the following criteria:

• The author is recognized as an expert by other meditation experts or laypeople;
• They have been in dialogue with meditation teachers or practitioners, including those regarded as senior or advanced;
• They have academic qualifications or achievements (including publications) in relation to the meditation tradition or otherwise;
• They hold or have held positions in relevant organizations, such as meditation bodies;
• They have meditation teaching experience and expertise;
• They maintain a personal meditation practice; and
• They have other qualifications that are material to the assessment.

For an author to be assessed as having outstanding qualifications it was required that they have high ratings on a number of these criteria. Having high ratings on at least the first two was treated as essential. It was not strictly necessary for the author to have high ratings on each of the other criteria. Having exemplary ratings on some criteria could on balance be treated as sufficient even where the author had lower ratings on one or more others.

Alan Wallace stood out as having outstanding qualifications with respect to Shamatha. Five authors (Craig Pearson, Norman Rosenthal, Bob Roth, Jonathan Shear, Fred Travis) stood out with respect to TM, and two authors (Ainslie Meares, Pauline McKinnon) with respect to Stillness Meditation. In each case TW made the initial assessment, and OC and JW confirmed the decisions.

The next step was to consider whether it would be sufficient to rely on those authors, or whether others should also be included. That assessment was made having regard to rules about sample size (sometimes referred to as *stopping rules*) that are applicable in the context of an evidence synthesis involving purposive sampling. Suri (2011) explains that there are two rules that can be applied. One is *data saturation*, which in the present context would mean that the existing authors provide virtually all of the available insights and perspectives relating to the practices. The other is *data sufficiency*, which would entail that the existing authors provide insights and perspectives that are sufficient for the purposes of the evidence synthesis. Data sufficiency can take into account practical constraints, such as limitations on time and resources. Suri indicates that data saturation is rarely achieved. Data sufficiency appears to better reflect what is considered appropriate in practice.

The scoping reading for Shamatha gave the impression that Wallace’s analysis is particularly rigorous and well informed. He provides clear and detailed descriptions of the Shamatha technique and experience, which integrate and contextualize traditional understandings with modern western perspectives and knowledge of other meditation practices. He also covers philosophical and historical aspects and has co-authored several scientific journal articles. Wallace discusses how key elements of his presentation of Shamatha are supported by other distinguished Buddhist practitioners. He makes what appear to be cogent arguments as to why alternative interpretations are problematic.

The scoping reading indicated that Wallace’s publications include extremely detailed instructions for Shamatha and tend to each add some important facet of the technique or experience rather than merely repeating content. It was therefore clear that, in order to get a complete understanding, it was necessary to review all of Wallace’s relevant publications. From the scoping reading it was evident that there were over 25 relevant publications by Wallace, including more than 15 books, and that the necessary detailed examination of those texts would involve a huge amount of work. It was not practicable to do the same level of reading and analysis with respect to other Shamatha authors. Having regard to the strengths of Wallace’s analysis (see paragraph above), we determined that reviewing his publications in a comprehensive and in-depth manner would lead to a higher quality understanding than reviewing the accounts of multiple Shamatha authors at a more superficial level. Based on these considerations, it was determined that the selection of Wallace achieved data sufficiency in relation to Shamatha.

The decision to focus on Wallace provides a concrete example of the way in which our method deals with the practical problem of how to reasonably limit the publications for review when faced with a very large body of expert literature. A further strength of our method is that, if other researchers feel that additional Shamatha authors may contribute further insights, they can readily extend the work to those authors using a similar process.

The five TM authors provide a range of perspectives. Pearson contextualizes TM relative to historical reports of contentless experience; Shear focuses on philosophical aspects; Travis conducts empirical studies; Roth is a meditation teacher; and Rosenthal is a psychiatrist. It was expected that each author might therefore draw out unique aspects of the technique and experience. The high-level impression based on the scoping reading was that, while that is correct, the authors also present the technique and experience in broadly similar terms. Adding another author may have yielded some additional insight, but the broad similarity in the five authors’ accounts suggested that these would be marginal. Consequently, it was concluded that the five authors together provide data sufficiency.

Data sufficiency with respect to Stillness Meditation was clearly achieved by the selection of Meares and McKinnon. Meares developed the practice, and McKinnon has taught it for over 35 years. Meares and McKinnon are responsible for all of the major publications concerning the practice.

#### Selection of Publications

The next step was to select publications of the authors identified above. Three of the TM authors (Pearson, Rosenthal, Roth) have recently published major works that appear to present their understandings of the practice in a consolidated form (Rosenthal, 2011/2012, 2016/2017; Pearson, 2013; Roth, 2018). For those authors those publications were therefore relied on alone^7^. Understandings of the other two TM authors (Shear, Travis), the Shamatha author (Wallace), and the Stillness Meditation authors (Meares, McKinnon) are spread across their publications. Relying only on their more recent materials would risk omitting important details. For that reason publications of those five authors were selected by applying eligibility criteria to lists of their full output.

The primary sources for the list/s of an author’s publications were searches in online databases, and reference lists and citations from relevant publications or websites. The rationale was that those are the main sources recommended for evidence syntheses (Gough et al., 2012/2017, 2013). Full details of the searches are provided in Supplementary Appendix B.

The searches were designed to capture all relevant publications, meaning that iterative searches were not required (ENTREQ checklist item 3). Iterative searches involve searching for select literature, reading it, conducting further searches on the basis of that reading, and so on (Gough et al., 2012/2017). In most cases, searches were conducted in Medline, PubMed, PsycInfo, Embase, Web of Science, Scopus, and Google Scholar. Other databases, including Amazon, PhilPapers, Philosopher’s Index, and Discovery (an internal University of Melbourne database) were also utilized where appropriate. Where possible, the author search field was narrowed using terms indexed in the databases, for example “wallace b alan.”

Details of the reference lists and citations that were relied on in addition to the searches are provided in Supplementary Appendix C. The searches, reference lists and citations were conducted or reviewed (as applicable) in January or February 2018. The only publication that was selected for review after February 2018 was Wallace’s (2018) book, *Fathoming the Mind*, which was released in October 2018. The justification for including that text was that it is a major work of more than 200 pages, and was released prior to 2019, when the analysis in the current project commenced. To the best of our knowledge, none of the eight authors published any other works that are commensurate in scale between March 2018 (the month following the searches) and March 2020 (the month in which the present paper was submitted for publication).

The eligibility criteria applied to each author’s list comprised inclusion criteria, and, in some cases, exclusion criteria. Publications that satisfied all inclusion criteria for an author, and that did not satisfy any applicable exclusion criteria, were selected for inclusion in the three evidence syntheses. The effect of the criteria was to excise from the full set of publications for an author at least some of the publications that were: (a) not relevant – i.e., that did not address the technique or experience; and/or (b) likely to merely duplicate relevant content from other publications in the set. To duplicate content in this context means to repeat the same idea without adding anything conceptually. It does not require word-for-word repetition.

The resulting sample comprised a core set of publications setting out the main elements of the author’s understanding of the practice, in some cases together with a small number of extraneous publications. The extraneous materials were mainly publications that were likely to merely duplicate the core content, but that had not been excised via the criteria. Where final samples contained some extraneous materials, that did not matter, since those publications could be identified as part of the extraction component (see section “Data Extraction” below).

The eligibility criteria included general criteria (see further below), which were the same for each author, and author-specific criteria. Author-specific criteria were utilized because they could incorporate particular features of an author’s publications in order to excise extraneous materials more effectively. As an example, the scoping reading indicated that Shear had published a major work in 1990 that appeared to present his understanding of TM in a consolidated form (Shear, 1990b). On that basis it was decided that Shear’s relevant publications from prior to 1990 would be unlikely to add anything to the understanding in his major work and later publications. An inclusion criterion that Shear’s publications be from 1990 onward was therefore utilized. Since the other authors had not published equivalent works in 1990, for them that inclusion criterion was not suitable.

The general criteria – applying for all five authors – were that the publication was a book, book chapter or journal article (not including encyclopedia entries), in English, and non-fiction. The author-specific criteria are set out in Supplementary Appendix C. As an example, the main Wallace criterion was that the publication includes two or more paragraphs (whether together or apart) on Buddhist shamatha technique or experience. That criterion had the effect of excising from Wallace’s full set of materials any publications that did not address the technique or experience, and that were therefore not relevant. It also excised any publications that contained minimal discussion (i.e., less than two paragraphs) about the technique or experience, on the basis that they likely merely duplicated content in the publications containing more detail.

As noted above, Supplementary Appendix C sets out the sources used to produce the author lists, and the author-specific eligibility criteria for each author. For ease of reading, it is structured as a step-by-step description of the process for selecting the publications of each of the five authors for whom the criteria were utilized. The general elements of that process are as described above, but the appendix provides the fine details, including:

• The author-specific criteria and the sources for the lists;
• Justifications for the criteria (in addition to those provided above); and
• Descriptions of how the criteria were applied at a practical level – for example, on the basis of citation, title, and abstract, or by reading the publication in full.

**FIGURE 1:**
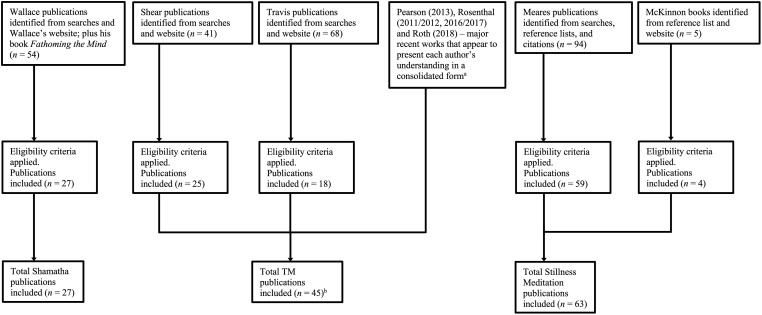
Flowchart summarizing the process for selecting publications. For additional detail (including qualifications), see Supplementary Appendix D. ^a^It was not necessary to apply eligibility criteria to these four publications. The purpose of the criteria was to identify samples of publications revealing authors’ understandings of the practices. For these three authors the four publications already comprised an appropriate sample (see section “Selection of Publications”). ^b^Two publications were selected for both Shear and Travis. These were only counted once in calculating the total TM publications.

A flowchart summarizing the selection process for all three practices is presented at Figure 1. PRISMA-style flowcharts with additional detail (including qualifications) are provided at Supplementary Appendix Figures D1–D3 of Supplementary Appendix D (ENTREQ checklist item 9; Tong et al., 2012, p. 6).

TW was responsible for the initial application of the eligibility criteria. For three of the five authors (Meares, McKinnon, Shear), the criteria could be applied in a manner that was straightforward, with no gray areas, for example by determining whether or not a text was published from a certain year onward. The only criterion for these authors that involved any appreciable subjective judgment was the Meares inclusion criterion that the publication refers to a practice that might be reasonably identified as Stillness Meditation. For that criterion, any gray area was removed by erring on the side of including all publications for which there was any doubt.

For the other two authors (Travis, Wallace), applying the criteria involved more scope for subjectivity, requiring for instance an assessment as to whether the main focus of the publication (or a specific portion of it) was the meditation technique and/or experience. To address that issue, guidelines were established for how to apply the criteria for those two authors (Supplementary Appendix E). In addition, for those authors OC acted as a second reviewer to test the reliability of the criteria. OC has no personal history with Shamatha, TM or Stillness Meditation. The criteria were applied by OC, independently of TW’s assessment, to random samples of 20% of the publications on each of the Travis and Wallace lists (Booth et al., 2013, p. 130; Campbell et al., 2014, p. 5). The independent assessment yielded 100% agreement with TW’s assessment, and on that basis the criteria and TW’s application of them were considered sufficiently reliable.

#### Final Publications Selected

The publications selected for inclusion in the evidence syntheses on the basis of the process above are listed in Supplementary Appendix Tables F1–F3 in Supplementary Appendix F for Shamatha, TM and Stillness Meditation, respectively. In most cases the entire publication was reviewed, but for a small number of books the review covered only the portion identified as relevant (see the three tables for details).

Figure 1 and Table 1 show for each practice the total number of publications included in the evidence syntheses. Table 1 additionally provides the number of books and the number of book chapters and journal articles. The totals for each practice are uneven, but that should not be taken to indicate that better coverage of one practice was obtained than for another. The evidence synthesis process was designed to ensure that excellent coverage was achieved for all three practices. Notwithstanding the difference in the number of publications, the sections of the Shamatha, TM and Stillness Meditation extraction tables (see below) on the features of the goal-state/s are similar in length, at 29, 35 and 32 pages, respectively. Furthermore, although considerably fewer publications were reviewed for Shamatha, the Shamatha extraction table (83 pages) is substantially longer than the tables for TM and Stillness Meditation (51 and 60). That mainly reflects that there are three types of Shamatha meditation, and that the Shamatha publications contain extremely detailed instructions on how to perform the technique.

**TABLE 1:** Number of publications included in the evidence syntheses.

**Practice**	**Books**	**Book chapters and journal articles**	**Total**
Shamatha	17	10	27
TM	5	40	45
Stillness Meditation	27	36	63

### Data Extraction

#### Types of Data to Be Extracted

##### General approach

The next step in the process was to extract particular data from the selected publications. The data that were extracted included: (a) descriptions of what it is like to experience the goal-states or the interim-states fundamental in achieving the goal-states (Nagel, 1974); and (b) descriptions of the features of the techniques fundamental in achieving the goal-states. These two types of information will be together referred to as information relating to the techniques and experiences. We treated as descriptions of what it is like to have an experience both descriptions of a specific experience at a particular point in time, and descriptions that were more generalized.

Information not required included theoretical, philosophical, and metaphysical understandings. That information was extracted only if it was thought necessary or helpful to properly interpret or contextualize the data relating to the techniques and experiences.

##### Teacher-related aspects

In order to compare the three practices on an even footing, the evidence syntheses do not focus on features specific to meditation sessions being led or supported by a teacher. For example, a teacher in Stillness Meditation will typically use calming touch on the meditator’s head and shoulders to provide a sense of safety and reassurance (Meares, 1979e, 1989; McKinnon, 1983/2016, 2011). Descriptions of the Stillness Meditation technique in the evidence syntheses do not refer to teacher-related aspects such as calming touch, but instead describe the technique as it would be performed by a meditator practising alone. According to Meares and McKinnon, the presence of a teacher normally leads to a deeper experience, but practising alone can still be effective (Meares, 1978/1986; McKinnon, 1983/2016, 2011). Key teacher-related aspects referred to in the traditional accounts of the three practices were still extracted, but this was done mainly for completeness and context.

##### Participant-based studies

The selected publications include certain participant-based academic empirical studies authored or co-authored by Shear, Travis or Wallace. Material in those studies was extracted if it indicated the expert’s understanding of the technique or experience that was separate to the participant-based findings. Across the full set of selected publications for all eight experts, material was also extracted if it was a participant-based finding about the experience of the goal-state/s, and the finding was from an academic empirical study and heavily relied on by the expert/s^8^.

#### Extraction Process

The first step in the extraction procedure was to read the selected publications (or the selected portions) in full and to mark the material that was potentially relevant, adopting a broad interpretation of that phrase. The second step was to work through those sections in detail and copy or summarize the material relating to the techniques and experiences in a Microsoft Word table for each practice. The order in which the publications were reviewed for this step is described in Supplementary Appendix G. Most of the material in the tables was extracted verbatim rather than summarized.

It was not necessary to copy *all* of the material relating to the techniques and experiences in the sections that had been marked. Material was extracted only if it added something conceptually to any material already in the relevant table. In this way, conceptual duplication of points in the table was avoided. A conservative approach was taken concerning whether material in the publications added anything conceptually to material in the table. For example, material could be taken as adding something where it:

• Expanded on or qualified a description in the table; or
• Simply repeated the description, but through the repetition indicated that the feature:
  • was regarded as central to the practice rather than marginal;
  • was recognized by multiple experts; or
  • remained part of the practice at a later point in time.

Supplementary Tables S1–S3 in the supplementary material are the final extraction tables for Shamatha, TM and Stillness Meditation, respectively. The tables together comprise the database for the current project. The set of tables is preceded by a document headed “Database Contents List” that lists the contents of the three tables and provides page numbers.

TW was responsible for the data extraction, and for the coding of the extracted material as described below (see section “Synthesis”). He was guided by regular feedback from OC and JW. OC also performed an independent review of a sample of the extraction and coding (Centre for Reviews and Dissemination, 2009; Noyes and Lewin, 2011a; Booth et al., 2013). For each of the three practices, one book was selected at random from the first five books reviewed for the practice. That meant that three books were selected in total (Meares, 1978/1986; Rosenthal, 2011/2012; Wallace, 2011b). In each of the three books, 10 pages were randomly selected from the pages that TW had marked in his initial review as containing potentially relevant material. Another 10 pages in each book were randomly selected from pages that TW had not marked. The independent review involved examining the extraction and coding that had been undertaken in respect of the initial set of 10 pages, as well as confirming that the second set of 10 pages did not include material that should have been extracted and coded. OC did not identify any issues with the extraction or coding for the initial set, and she confirmed that the second set did not contain relevant material.

### Synthesis

The final step in the process was to synthesize the extracted material. Synthesis is a form of analysis that involves generating new knowledge by forming connections within extracted material that have not been made before (Mays et al., 2005b; Suri, 2011, 2014; Gough et al., 2012/2017; Noyes et al., 2015). As referred to above, Popay et al. (2006) provide a series of synthesis techniques that the reviewer employing narrative synthesis can choose from and can tailor as appropriate for their extracted data. The critical requirement is that the technique/s be applied in a structured manner.

The technique selected for the current project was thematic analysis. This section describes the tailored form of thematic analysis that was utilized in the present evidence synthesis and how it was applied in a structured manner. The form of analysis used in the project involves several of the other techniques identified by Popay et al. (2006), including textual description, grouping of publications, tabulation, and translation of themes and concepts. It will be referred to in the project as a single technique, but it could alternatively be presented as multiple techniques.

Popay et al. (2006) provide a four component structure for the synthesis element, consisting of theory development, preliminary synthesis, detailed synthesis, and synthesis assessment. However, they emphasize that this is not prescriptive and that structuring will vary with individual syntheses (pp. 11, 12). In practice, reviewers tend to treat narrative synthesis as the second and third components, and either disregard the first and fourth, or address them separately, and without reference to the guidelines (e.g., Bailey et al., 2015; Madden et al., 2018).

A similar approach has been taken in the current project. On the basis that it is unnecessary and unhelpful for the purposes of the project, the first component (theory development) has been left out and no distinction has been drawn between the second and third components (the preliminary and detailed syntheses). The fourth component (synthesis assessment) is addressed in the “Discussion” section/s, independently of the guidelines.

Synthesis comprised both coding of the extracted material and the steps taken to compare the techniques and experiences across the three practices. To code the extracted material, the extraction tables for the three practices were each divided into two sections: “Techniques and Interim-States” and “Goal-state/s.” As material was extracted, it was placed in one or both of those sections, as appropriate. Within those sections, the material was placed under one or more additional subject headings. For example, extracted material describing a goal-state as wakeful was placed in the “Wakefulness” section of the “Goal-state/s” part of the table. In that way it was coded as both “Wakefulness” and “Goal-state/s.”

The codes “Techniques and interim-states” and “Goal-state/s” were developed deductively, meaning they were determined at the outset (ENTREQ checklist item 19). All other codes were developed inductively: They were created as required, based on the features referred to in the extracted material (ENTREQ checklist item 19; Popay et al., 2006; Thomas and Harden, 2008). Codes were applied to words, phrases, sentences, or paragraphs, as appropriate to capture the relevant feature (Gough et al., 2012/2017; Bryant, 2017). Coding was treated as provisional until the process was complete (Bryant, 2017). During the process, adjustments to the coding were made to ensure that it was consistent and precise, and that individual codes were appropriate in scope (Richards, 2006/2015; Thomas and Harden, 2008). The full coding is shown in the final extraction tables (Supplementary Tables S1–S3).

The feature silence/quietness was compared across the traditions by juxtaposing the database material coded silence/quietness for each practice (Supplementary Tables S1–S3, section “Silence, Quietness”). The steps for the comparison of the techniques and the other aspects of the experiences will be presented elsewhere (Woods et al., 2020a, b).

## Results

The database reveals that in each of the three practices the goal-state/s have a large number of experiential features, and that in each practice these overlap to various degrees (see Supplementary Tables S1–S3, section “Features”). Silence/quietness is one of many features that are reported or implied in all three traditions. Other examples are the meditator being conscious and awake, having no thoughts, perceptions, or mental images, and experiencing calm and rest.

In this section we report our findings regarding the feature silence/quietness. The extracted material for that feature is provided in the database, and for ease of reference it is also set out in Tables 2–4. Those three tables present the material for Shamatha, TM and Stillness Meditation, respectively.

**TABLE 2:** Shamatha extracted material for the feature silence/quietness.

**Expert text**	**Extracted material**
Wallace, 2011b	“… the sheer silence, the stillness, the lack of perturbation …” – p. 120
	“… a quiet, percolating, radiating sense of serenity, joy that is very malleable.” – p. 145
Wallace, 2009/2014	“… blissful, luminous, conceptually silent state …” – p. 93
Wallace, 2011a	“… a luminous, blissful, silent space of awareness …” – p. 40
	“… peaceful, luminous silence …” – p. 111
	“When the mind goes quiet, what remains is the substrate consciousness.” – p. 196^a^
	“The conceptual mind is quiet …” – p. 208
	“… the silent, luminous, blissful substrate consciousness.” – p. 249

*^*a*^Wallace uses the term *substrate consciousness* to refer to the state aimed for in Shamatha practice (Supplementary Table S1, section “Substrate Consciousness”). He indicates that substrate consciousness can also be accessed in sleep, death, coma, fainting, and hypnosis, but that the degree of attentional stability and vividness may differ.*

**TABLE 3:** TM extracted material for the feature silence/quietness.

**Expert text**	**Extracted material**
Pearson, 2013	Quoting a meditator describing their first session: “… I experienced a silent, inner state of no thoughts, just pure awareness and nothing else …” – p. 29
	“… a state of deep inner silence and peace …” – p. 29
	“[The mind] can become calm, quiet, silent, while remaining awake. This is [pure consciousness].” – p. 44
	“Your mind is awake but silent and serene.” – p. 44
	“[Pure consciousness] is the most simple and natural experience a person can have – the experience of awareness in its most quiet state.” – p. 49
	“[Pure consciousness] is still, silent …” – p. 50
	Quoting a meditator: “I will never forget the first experience I had of the [TM] technique … The movement of my awareness from the active level … to the field of silence within myself … was like diving into a pond of pure joy.” – p. 51
	Quoting a meditator: “[M]y mind settles down, thoughts become less and then suddenly all thought activity ceases and I slip into an unbounded ocean of awareness which is pure, quiet, unexcited …” – p. 52
	Quoting a meditator: “… I sometimes reach a state of complete silence …” – p. 52
	“… an inner sanctuary of infinite silence, peace, and joy.” – p. 175
Roth, 2018	“… [D]eep within is a level that is calm yet alert; silent yet wide awake.” – p. 17
	“It is your own quiet inner self …” – p. 17
	“… the quietest, deepest level …” – p. 34
	“… quiet, peaceful, transcendent state of awareness.” – p. 37
Shear and Jevning, 1999a	“The experience would appear to be one of consciousness alone by itself – pure, silent, and empty of all ‘phenomenal’ objects.” – p. 194
	“… silent and fully awake inside …” – p. 195
	“… alert silence of pure consciousness …” – p. 205
	“… simplest, non-active, completely silent state [of consciousness] …” – p. 205
Shear, 2006b	“… *absolute*, *pure silence*.” – p. xviii
Shear, 2006c	“… completely silent state …” – p. 26
Rosenthal, 2016/2017	“… peace and quiet.” – p. 31
	“What, then, are the usual elements of transcendence? That was the question I asked a group of students, at Loyola University’s Stritch School of Medicine in Chicago, who had learned to meditate. They replied with gusto: stillness, quiet, no boundaries, no thoughts, and bliss. In short, they captured the essential spirit of the state.” – p. 31
	“It is quiet there. Still. Peaceful. Thoughts may still come and go but sooner or later … silence … all thoughts have gone.” – p. 35
Rosenthal, 2011/2012	“… I use the mantra in a way that allows my mind to settle into quietude.” – p. 18
	“As I continue to meditate, all that mental noise quiets, and I welcome the silence.” – p. 18
Travis and Pearson, 2000	Quoting a participant: “… [A] couple of times per week I experience deep, unbounded silence, during which I am completely aware and awake, but no thoughts are present.” – p. 81
Arenander and Travis, 2004	“… complete silence …” – p. 114
Travis et al., 2005	Quoting a meditator: “… I am completely still. It’s absolute silence.” – p. 128
	“It is being awake in the midst of silence.” – p. 128
Travis, 2009	“This silent interiority of the mind has been called pure consciousness.” – p. 28

**TABLE 4:** Stillness Meditation extracted material for the feature silence/quietness.

**Expert text**	**Extracted material**
Meares, 1978/1986	“… [Stillness Meditation allows] the mind to come to a state of quiet and stillness.” – p. ix Meares coined the term “Mental Ataraxis” to refer to the meditation, and to distinguish it from other forms of meditation (pp. xi-xii, 6). He explains: “[The Greek term] ‘*ataraxis*’ simply means ‘an absence of disturbance.’ Mental Ataraxis concerns quiet of mind and peace of mind.” – p. xii Meares later stopped using the term on the basis that it was confusing for some people (Meares, 1989, pp. 108–109).
	“[The experience] comes as quietness, and an ease, pervading everything – our thoughts, our feelings, our whole being.” – pp. 24–25
Meares, 1989	“… [O]ur mind goes quiet of itself …” – p. 46
	“Nothing to disturb the quiet within.” – p. 122
	“When our mind is still, there is nothing. Just quietness. Just *being*.” – p. 115
Meares, 1987/1991	“Let the mind run quiet. Just quiet.” – p. 17 Meares pp. 21, 80, 115 makes similar comments.
	“… just quietness, a stillness of effortless tranquility.” – p. 114
Meares, 1988	“The idea is just to let our mind be quiet … It is just a quietness, a stillness of the mind. Thoughts may come … Just let them be, and in a few moments they fizzle out. A silence comes to our mind. Then a few more thoughts. Then silence again. It is just a coming and going.” – pp. 73–74
Meares, 1979b	Writing from the perspective of a notional patient in conversation with himself as Stillness Meditation teacher/therapist: “You harp on the silence [a]nd the stillness …” – p. 79
Meares, 1984	“… The silent stillness [o]f the meditation …” – p. 46
Meares, 1987a	“Ease is quietness of mind …” – p. 25
	“… The storm whips up the waves, [b]ut deeper down the world is quiet.” – p. 42
McKinnon, 1983/2016	“… silence and stillness …” – p. 228
McKinnon, 2011	“… the experience of only stillness … an all-encompassing silence.” – p. 44
	“… quiet and silence …” – p. 162
McKinnon, 1991	“*Only complete quiet*…” – p. 127
McKinnon, 2002/2008	“… completely still and silent …” – p. 52

There are four main findings regarding how the experts refer to silence/quietness. The first is that they frequently present the feature alongside other features of the goal-states. For example, Wallace (2011a, p. 249) refers to the Shamatha goal-state as “silent, luminous, [and] blissful.” Pearson (2013, p. 175) describes the TM experience as involving “silence, peace, and joy.” Meares (1978/1986, p. 24) indicates that in Stillness Meditation there is “quietness, and an ease.” In these examples, silence/quietness is presented alongside the features luminosity, bliss/joy, and ease/peacefulness.

The second finding is that the experts often use terms like silence or quietness without elaboration as to their precise meaning. This point is also illustrated by the quotations in the paragraph above. For example, Pearson’s (2013) reference to “silence, peace, and joy” indicates that those three qualities are features of the TM goal-state, but it does not provide further detail concerning those features. That leaves unresolved the precise nature of silence as a specific feature. In other words, what exactly does the word silence add to the description of the experience?

Other passages in the expert texts do provide elaboration as to the nature of the silence/quietness as a specific feature. Those passages are the subject of our third and fourth findings.

The third finding is that certain passages indicate a particular connection between the silence/quietness and the absence of concepts, mental activity/noise, thoughts, and disturbance, or between the silence/quietness and stillness. Wallace says that the Shamatha goal-state is “conceptually silent” (Wallace, 2009/2014, p. 93), and that the “conceptual mind is quiet” (Wallace, 2011a, p. 208). Pearson (2013, p. 51) quotes a TM meditator who describes the transition from an “active level” of awareness to the silence of the goal-state. Rosenthal (2011/2012, p. 18) describes the transition in TM by saying “all [the] mental noise quiets, and I welcome the silence.” Elsewhere he says: “Thoughts may still come and go but sooner or later … silence … all thoughts have gone” (Rosenthal, 2016/2017, p. 35). Meares (1988, p. 74) discusses the movement into the Stillness Meditation goal-states in very similar terms: “… [Thoughts] fizzle out. A silence comes to our mind. Then a few more thoughts. Then silence again.” He additionally associates quietness with the absence of disturbance in the experience (Meares, 1978/1986, p. xii, Meares, 1987a, p. 42, Meares, 1989, p. 122). For example, he notes that for a period of time he used the term “Mental Ataraxis” to describe the practice (see Table 4). He explains that *ataraxis* is a Greek term meaning “absence of disturbance,” and he adds that Mental Ataraxis concerns “quiet of mind” (Meares, 1978/1986, p. xii). In describing the goal-states he says that there is “[n]othing to disturb the quiet within” (Meares, 1989, p. 122).

Numerous passages in the expert texts indicate that silence/quietness is closely related to stillness (see section “Introduction” above, and Tables 2–4). Sometimes the term stillness appears to be focused on the absence of thoughts. For example, Roth (2018, p. 164) quotes a TM meditator who says, “Thoughts will come and go, and it will be five minutes before I can get to [the] stillness. I go to this incredible place where I’m not even thinking anything.” Other times the term stillness seems to be used in a broader manner that reflects both the absence of thoughts and other disturbances. Wallace (2011b, p. 62), for instance, indicates that in the Shamatha goal-state “the mind is quiescent … no turbulent thoughts or emotions arising … still.” He also refers to the “silence, the stillness, the lack of perturbation” (p. 120) in the experience. Pearson (2013, p. 25) describes what is aimed for in TM as a “state of perfect stillness, beyond all perceptions, thoughts, and feelings.” Meares (1977c, p. 131) says similarly that: “[Stillness Meditation] is characterized by stillness. There is an absence of intellectual activity, an absence of sensory experience and an absence of emotion.” McKinnon (1983/2016, p. 218) explains that in the stillness “nothing disturbs [the meditator] at all.”

The fourth and final finding is that the experts in each practice indicate that the silence/quietness is in some sense complete. As noted above, Wallace (2011b, p. 120) refers to the Shamatha goal-state as involving “sheer silence.” The TM experts describe the silence in TM as deep, complete, absolute, infinite, and pure (see Table 3), and they refer to the goal-state as the quietest experience possible (Pearson, 2013, p. 49; Roth, 2018, p. 34). In Stillness Meditation there are references to a complete and all-encompassing silence or quiet (see Table 4), and the goal-state experience is also described as “just quietness” (Meares, 1987/1991, p. 114, 1989, p. 115).

## Discussion

The current paper is an evidence synthesis examining silence/quietness as a specific feature of the goal-states in Shamatha, TM and Stillness Meditation. It is based on expert texts from within the three traditions. We have designed and presented a detailed evidence synthesis method that is appropriate for the research objectives. The method involves the production of the database comprising Supplementary Tables S1–S3. The database was produced by way of a detailed review of 135 traditional publications. It contains rich descriptions of the meditation techniques and experiences extracted from those texts. The descriptions have been organized – or coded – in the database to differentiate individual features. These include all the features of the goal-states referred to in the traditional accounts (Supplementary Tables S1–S3, section “Features”). Silence/quietness is one of the central features of the goal-states. Our findings concerning silence/quietness are based on the experts’ references to that feature in the database (see Tables 2–4).

The paper is part of the Contentless Experience Project, a broader project that includes three evidence syntheses. The present evidence synthesis presents our findings concerning silence/quietness as a specific feature of the goal-states. The other two syntheses (Woods et al., 2020a, b) will provide our findings concerning features of the meditation techniques and interim-states, and the features of the goal-states other than silence/quietness.

### Summary of the Findings

The evidence synthesis method and the database lead to four main findings concerning the feature silence/quietness. The first finding is that the experts in the three practices often refer to the silence/quietness alongside other features of the goal-states. Pearson (2013, p. 175), for example, describes the TM goal-state as involving “silence, peace, and joy.”

The second finding is that frequently the experts do not elaborate as to the precise meaning of terms such as silence and quietness. Pearson’s reference to “silence, peace, and joy,” for instance, does not provide any detail concerning the meaning of the term silence.

The third finding is that the experts draw a particular connection between the silence/quietness and the absence of concepts, mental activity/noise, thoughts, and disturbance, and between the silence/quietness and stillness. In Shamatha the silence/quietness is linked to the absence of concepts, in TM it is linked to the absence of mental activity/noise and thoughts, and in Stillness Meditation it is linked to the absence of thoughts and any other disturbance. The silence/quietness is closely associated with stillness in each of the practices. The experts use the term stillness in a range of ways. In its broader usage it appears to reflect the absence of thoughts and the absence of other disturbances such as perceptions, mental images, and negative feelings.

The fourth finding is that the silence/quietness is reported as being in some sense complete. Terms such as sheer, deep, absolute, pure, and all-encompassing are used.

### Silence/Quietness Reflects the Absence of All or Certain Types of Disturbance

In reflecting on the findings, we can begin by noting that in each practice the experts report or imply that the goal-states involve an absence of disturbance. In Stillness Meditation the term “absence of disturbance” is given a particular prominence, but the concept is also conveyed in the other two practices (Supplementary Tables S1–S3, section “… Absence of Disturbances”). Disturbances include (but are not limited to) thoughts, sounds, other perceptions, mental images, and negative feelings. Since there are no disturbances in the goal-states, meditators in the practices experience stillness in the broad sense of that term (see above).

From these observations, it is evident that in terms of the absence of disturbances the experiences in the three practices are at least broadly the same. The findings regarding silence/quietness (see above) indicate that the experts see that feature as reflecting at least the absence of certain types of disturbance, and possibly the absence of all forms of disturbance. To explain this point it is necessary to consider the most relevant categories of disturbance in turn.

For each of the practices it is clear that terms such as silence and quietness reflect at least the absence of thoughts. Rosenthal (2016/2017, p. 35) describes the silence in TM where “all thoughts have gone,” and Meares (1988, p. 74) says that in Stillness Meditation “[thoughts] fizzle out” and “[a] silence comes to … mind.” The Shamatha passages do not directly link the terms silence and quietness to the absence of thoughts, but they do link them to the absence of concepts. Thoughts involve concepts, and this implies that the terms also reflect the absence of thoughts.

In the passages in the database the experts do not expressly state that the term silence reflects the absence of sounds. However, the absence of sounds is reported in each practice, the term silence is conventionally understood as meaning or including the absence of noise^9^, the experts give no indication that the conventional understanding is not applicable, and they refer to the silence as sheer, absolute, all-encompassing, and so on. On this basis it seems implied that in each practice the experts intend the term silence to extend to the absence of sounds.

A similar analysis and conclusion applies for the term quietness, except that for Stillness Meditation the indications that quietness reflects the absence of sounds are more explicit. Discussing that practice, Meares expressly links the term quietness to the absence of all disturbances. He explains that “absence of disturbance” concerns “quiet of mind” (Meares, 1978/1986, p. xii), and makes clear that, in that context, “absence of disturbance” refers to the absence of *all* disturbances. He also says that there is “[n]othing to disturb the quiet within” (Meares, 1989, p. 122), and that, “When [the] mind is still, there is nothing. Just quietness” (Meares, 1989, p. 115).

Since Meares treats the term quietness as reflecting the absence of all disturbances, he sees the term as reflecting not just the absence of thoughts and sounds, but the absence of other disturbances as well. As noted above, these other disturbances include non-auditory perceptions, mental images, and negative feelings. It is not clear from the passages in the database whether the Shamatha and TM experts ever intend the word quietness to reflect the absence of these other disturbances. It is also not clear whether the experts in any of the three practices ever intend the word silence to extend beyond the absence of thoughts and sounds in this manner.

Wallace’s (2011b, p. 120) reference to the “silence, the stillness, the lack of perturbation” in the Shamatha goal-state provides an example of how the scope of terms such as silence and quietness can be unclear. The phrase “the stillness, the lack of perturbation” fits with Wallace’s (2011b, p. 62) earlier reference to the mind being still, without “turbulent thoughts or emotions arising.” In the framework that we have been discussing, the turbulent emotions constitute a form of disturbance other than thoughts and sounds. It appears that the word stillness is being used in the two passages in the broad sense (see above) covering the absence of all disturbances, including turbulent emotions. The scope of the term silence in the passage “the silence, the stillness, the lack of perturbation” is unclear. It could be interpreted as narrower in scope than the term stillness, reflecting only the absence of thoughts and sounds. Alternatively, the two terms could be treated as equal in scope, reflecting the absence of thoughts, sounds, *and* other disturbances, including turbulent emotions. That broader scope is also equivalent to Meares’ broad usage of the term quietness (see above).

Based on the passages in the database, each of these interpretations seems reasonable. As noted above, the term silence is conventionally understood as meaning or including the absence of noise. The passages in the database provide no indication that it would be inappropriate to understand the term silence in this way. If someone reading Wallace’s reference to silence (see above) conceived that term as being only about the absence of noise, they might interpret it as reflecting only the absence of thoughts and sounds. The reason for this is that thoughts and sounds can readily be understood as forms of noise, whereas it is harder to construe other disturbances such as non-auditory perceptions, mental images, and negative feelings in that way. Sounds, as we have been using that term, are auditory sense impressions from external sources. They clearly constitute noise. Thoughts can easily be conceived as a form of internal noise. Other disturbances could possibly be treated as noise in a metaphorical sense: As the meditator approaches the goal-state/s, the disturbance – or noise – is turned down, and in the goal-state/s it reaches zero. Clearly, though, treating these other disturbances as noise is more of a stretch.

Numerous passages in the database leave room for the term silence to be interpreted in a broader manner that extends beyond the absence of noise. In particular, they allow for the term to be interpreted as reflecting a complete calm or absence of disturbance^10^. If the person reading Wallace’s reference to silence had in mind this extended definition, they would likely see it as reflecting the absence of thoughts, sounds *and* other disturbances.

One further point is that the two interpretations can co-exist. The term silence could mean the absence of noise *and* the complete absence of disturbance. As such, when Wallace uses the word silence, at one level that may reflect simply that thoughts and sounds are absent, and at another it may reflect that there are no thoughts, sounds *or* other disturbances.

In summary, the analysis above provides the following insights. In each practice it is reported or implied that the goal-states involve an absence of disturbances including thoughts, sounds, other perceptions, mental images, and negative feelings. The terms silence and quietness reflect at least the absence of thoughts and sounds, and this fits neatly with a conception of silence/quietness as the absence of internal and external noise. For the most part, the experts do not clearly indicate that the terms silence and quietness can extend to the absence of disturbances other than thoughts and sounds, however they also do not rule this out. Interpreting the terms in that extended manner would fit with a conception of silence/quietness as complete calm or absence of disturbance.

In view of these conclusions, researchers seeking to understand silence/quietness in contentless experiences should keep in mind the potential for a single term to be used in different ways. In particular, the terms may reflect the absence of thoughts and sounds, the absence of thoughts, sounds and other disturbances, or they may have each of those meanings at the same time.

This conclusion fits with the observation that there is often a degree of flexibility in the terms the experts use to describe the goal-states. The terms tend not to be defined in a rigid and very specific manner. The flexibility seems important given that the goal-states are so unusual compared to ordinary waking experiences. They are said to involve an absence of concepts and an absence of subject-object duality, and can therefore be challenging to describe (see Supplementary Tables S1–S3, section “Features”). Wallace explicitly draws attention to the fact that a single term can have multiple meanings (see Wallace, 2011b, pp. xi–xii; Supplementary Table S1, section “The Term Nature of the Mind”).

### Silence/Quietness May Be a Unique Feature

Recognizing that the silence/quietness reflects the absence of all or certain types of disturbance is a major step toward understanding it. It is important, however, to appreciate that there can be more than one feature that reflects the absence of particular disturbances, and this does not necessarily mean that those features are identical. Each feature may still be unique.

As an example, we have noted above that the terms silence, quietness, and stillness may in some cases each be used to reflect the absence of all forms of disturbance. That indicates a substantial overlap between the features silence/quietness and stillness, but it does not necessarily mean that they are identical. Silence/quietness might be some combination of calm and the absence of noise, whereas stillness might combine calm and the absence of movement. Movement here refers to movements of the mind, such as fluctuations in thoughts or feelings (see, e.g., Supplementary Table S1, section “Stillness and Movement”). The absence of noise and the absence of movement are each implied by there being no disturbances, however it may be that experientially they are different.

The passages in the database in fact provide little detail as to whether or how the feature silence/quietness is unique. As explained above, the terms silence and quietness are frequently used without elaboration as to their precise meaning, and most of the elaboration that is provided is about the terms reflecting the absence of all or certain types of disturbance. The experts do not explicitly address how the silence/quietness is distinct from stillness. It may be that in some cases they intend the terms as synonyms, describing an identical experiential quality. In other cases, they may use the terms to refer to qualities that are overlapping but distinct.

To the extent that the experts are seeking to convey distinct qualities, they appear to mainly rely on the words silence, quietness, and stillness themselves. The words themselves may prompt in the reader some intuitive sense of the features silence/quietness and stillness, and how they may be different. The experts may consider that giving this intuitive impression is sufficient for the purposes of the texts. While the texts provide a great deal of detail about the experiences, they are not intended to provide exhaustive technical descriptions of every facet.

The experts may also consider that there is not much that can be said about the unique elements beyond what is conveyed using the terms themselves. As referred to above, the experts note that there are limitations when it comes to describing the goal-states in words.

### Silence/Quietness Is Connected to the Other Features of the Goal-States

**FIGURE 2:**
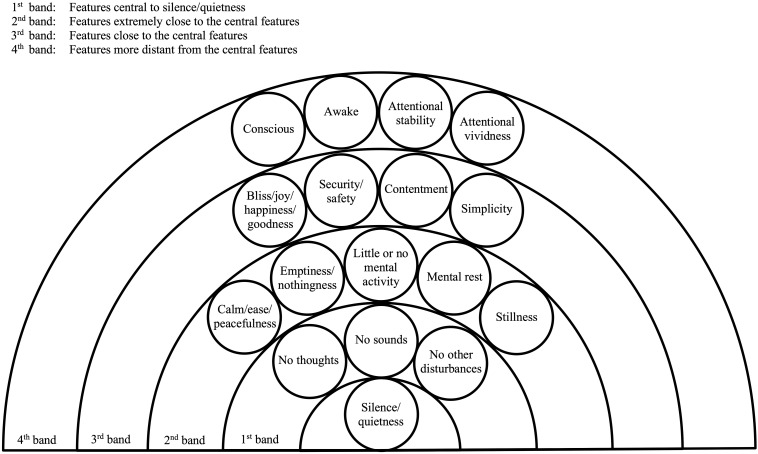
Closeness of connections between silence/quietness and other features of the contentless experiences. For each of the four bands the features in circles are examples of the features in that band. The present paper explains how the experts in Shamatha, TM and Stillness Meditation refer to the feature silence/quietness. The section headed “Features” in each of Supplementary Tables S1–S3 shows how the experts refer to the other features.

From the database as a whole it is evident that in each practice the feature silence/quietness is interconnected with all the other features of the goal-state/s. Figure 2 is a diagram that illustrates how silence/quietness has closer connections with some features than with others.

The full diagram represents the goal-state/s in any one of the three practices. Since silence/quietness is the focus of this paper it is placed in the middle of the diagram, at the bottom. Working outward from silence/quietness there are four bands of features which will be explained below. The features shown in circles are examples of the features in each band, not the full set of features. The features in circles are reported or implied in each of the three practices.

For clarity and simplicity, the features in circles are shown in the diagram as being distinct but connected. The reader should, however, bear in mind that an alternative way to represent the connections would be to show overlaps between the features. If that alternative approach was taken, the feature silence/quietness could, for example, be depicted as including the features in the first band (discussed below), or as otherwise overlapping with them to a large extent.

The experts themselves do not refer to there being four bands. We are dividing the features into four bands purely to illustrate the varying connections between the features.

A key conclusion from our earlier analysis was that silence/quietness can be conceived in different ways. The experts make clear that it reflects the absence of thoughts and sounds, and they leave scope for a broader conception in which it also reflects the absence of other disturbances. The features no thoughts and no sounds can be treated as central to – or intimately connected with – silence/quietness, and for this reason they are placed in the first band. On the broader conception of silence/quietness the feature no other disturbances is also central, so it is also in that band. The absence of other disturbances is represented as a single feature, but it could equally be presented as the component features no other perceptions, no mental images, and so on.

The features in the first band together represent the feature no disturbances. That feature appears equivalent or very nearly equivalent to the feature calm/ease/peacefulness. It also seems extremely close to the features emptiness/nothingness, little or no mental activity, mental rest, and stillness, where stillness is understood in the broad manner described earlier. These four features each reflect the absence of mental content such as thoughts, sounds, other perceptions, mental images, etc., but as explained above each may also have a unique experiential quality. In the diagram, the features calm/ease/peacefulness, emptiness/nothingness, little or no mental activity, mental rest, and stillness are in the second band. That indicates their extreme closeness to the features central to silence/quietness.

The third band represents features that may be slightly more removed from the central features but that are still closely connected to them. The examples in the diagram are bliss/joy/happiness/goodness, security/safety, contentment, and simplicity. Bliss/joy/happiness/goodness, for instance, appears different to the central features (i.e., the absence of disturbances), but it seems closely related to them, and in particular the absence of negative feelings. In Shamatha, for example, bliss or happiness is said to arise due to the mind no longer being “pummeled to death with afflictions, craving, hostility, and aversion” (Wallace, 2010, p. 32).

The fourth band represents features that are less closely connected to the central features. For this band, the examples are that the meditator is conscious (as opposed to not conscious), awake (as opposed to not awake), and has a degree of attentional stability and attentional vividness. These features have some connection with the absence of disturbances but it is not as close as for the other features above. For example, the feature conscious is connected to the absence of disturbances in that the meditator can only report the absence of disturbances because they were conscious of it during the goal-state. That connection is not specific to the absence of disturbances, however. There is a similar connection between the feature conscious and all the other features of the goal-states.

The present paper examines silence/quietness as a specific feature of the goal-states – i.e., as one of the many features that make up the experience as a whole. It is also possible to treat the silence/quietness as the entire goal-state/s in each practice. For example, a meditator may say that they had an “experience of silence,” and mean not just silence specifically, but rather all of the goal-state features, including silence, awakeness, bliss/joy/happiness/goodness, and so on. Researchers wishing to understand silence/quietness in this much broader sense would need to examine all of the goal-state features in detail. Our database contributes to this endeavour by identifying each of the goal-state features in the traditional accounts, and showing how the experts refer to them.

### The Method Provides a Strong Evidence Base

The analysis above is grounded in our findings concerning how silence/quietness is referred to in the database. Readers can have confidence that the findings accurately reflect how the feature is conceived within the traditions because the database and findings have been generated using an appropriate scientific method. The database represents a stronger evidence base than traditional descriptions identified by researchers in an unsystematic and/or opaque manner.

The method is both rigorous and transparent. Rigour is ensured by relying only on experts with outstanding qualifications; using objective criteria to identify samples of those authors’ publications that reveal their understandings of the practices; working through those publications systematically and in fine detail in order to extract relevant material; and fully coding the extracted text. Transparency is provided by documenting each element of the process, satisfying the ENTREQ reporting guidelines, and making the full method and database available.

In our analysis we conclude that certain aspects of the experts’ understandings are unclear. For example, it is unclear exactly if and when the experts intend the words silence and quietness to extend to disturbances other than thoughts and sounds, and it is unclear precisely how silence/quietness differs from stillness. The discovery of these ambiguities represents a strength of our method. A more superficial approach would likely fail to recognize the fine distinctions to which they relate. Identifying the ambiguities helps to generate research questions that can be examined in future studies to provide greater clarity (see section “Future Research” below).

### Limitations

There are three main limitations of the evidence synthesis.

#### Subjectivity

The method incorporates a series of structural elements designed to provide an objective basis for making decisions relating to the selection, extraction, and synthesis of expert texts or material within them. These include numerous rules applied by one of our team members and independent assessment by a second team member, for a sample of publications, to identify any issues with the first team member’s decisions. A limitation of the method is that there remains a degree of subjectivity in how the rules are applied. As an example, our judgment that a description concerns what it is like to have an experience and should therefore be extracted may differ to the assessment of other researchers.

All forms of evidence synthesis involve a degree of subjectivity (Centre for Reviews and Dissemination, 2009; Gough et al., 2012/2017; Bryant, 2017). A strength of our approach is that it involves much less subjectivity than most existing analyses of traditional accounts. Since existing analyses tend not to use an explicit scientific method they generally do not include the explicit structural elements that we have utilized in order to reduce subjectivity.

We have aimed to be as transparent as possible. The transparency allows others to critique or audit the process and see if the findings replicate. If experts in or outside the traditions consider that any facet of the method or its application is problematic, they are free to argue that point.

#### Sample of Publications

The second limitation is that the method involves selecting and reviewing only a sample of the traditional publications for the three practices rather than all of them. As noted earlier, the large number of traditional publications for the three practices means that it is not feasible to review them all. Examining additional publications (outside the sample) might clarify some aspects of the goal-states. However, the sampling strategy was explicitly designed to ensure that the selected publications allow a deep understanding of how the experiences are described in each practice.

#### Reliability of the Traditional Accounts

The third limitation is that there are various issues concerning the reliability of the descriptions of meditation experiences in the database. The data and findings allow us to address our specific research question, namely, “How is the silence of the goal-states described in the traditional texts?” However, it cannot be simply assumed that the descriptions in the traditional publications reflect with a high degree of accuracy the experiences that meditators have in practice.

For example, the traditional descriptions are likely to be normative to some degree and can be influenced by doctrinal considerations (Dunne, 2015; Markovic and Thompson, 2016). In addition, while the selected authors have outstanding qualifications as experts in the practices, they do not necessarily have the expertise in phenomenology or science that may be required to obtain or provide highly accurate descriptions of experience. Reports are generally regarded as more reliable if they concern a specific experience at a particular point in time, and if there is little delay between the experience and the report (Hurlburt and Schwitzgebel, 2007, pp. 14, 16–17). In that circumstance the reports are much less vulnerable to biases and other limitations of memory. Many of the descriptions in the database are generalized rather than specific, and do not indicate the delay. Some researchers may consider that the reliability of those descriptions is so questionable that they should be excluded altogether, rather than treated as indicating what it is like to have the experience.

The traditional accounts also have certain strengths when it comes to reliability. For instance, typically they take into consideration the expert’s personal experience during meditation and the experience of large numbers of other meditators, such as students, patients, or peers of the expert. The experts will often describe a particular feature of the experience and then provide examples from individual meditators (quoted or paraphrased) to support those descriptions. In numerous cases the reports from the individual meditators relate to a specific experience at a particular point in time.

Lutz and Thompson (2003, p. 38) have argued that the practical methods used to become aware of experience in Buddhist shamatha and insight practices are “far more developed” than in the western tradition of phenomenology (see also Depraz et al., 2002, pp. 206, 224). Wallace says that experienced meditators can provide “detailed, accurate [and] objective” reports (Wallace and Hodel, 2008, p. 178), although the current scientific evidence for this claim is inconclusive (Abdoun et al., 2019). Shear (2007, p. 41) contends that attaining contentless experience in TM or any other practice “provides an optimum platform for [then] investigating … contents of mind,” since the experience reduces internal noise and distortion.

All methods for examining experience have substantial limitations relating to reliability, even the popular *micro-phenomenology*, which claims to provide “great precision” (Petitmengin, 2006, pp. 230–231; Hurlburt and Schwitzgebel, 2007, pp. 186, 293, 2011b p. 208). Traditional accounts are a major source of information about meditation practices and meditation researchers generally agree that they have sufficient reliability to be of value (Studstill, 2005, pp. 52, 85; Lindahl et al., 2014; Sedlmeier et al., 2014, p. 620; Lutz et al., 2015; cf. Sharf, 1995). For this reason it makes sense to work out what the accounts have to say, while bearing in mind the reliability issues. Other methods should also be used, and any findings that converge are more likely to be credible (Hurlburt and Schwitzgebel, 2007, p. 186, 2011a; Millière et al., 2018, p. 18).

### Future Research

A sensible next step in exploring silence/quietness as a feature of the goal-states in the three practices would be to conduct participant-based studies. Those studies could use interviews (e.g., Lindahl et al., 2014), questionnaires (e.g., Kok and Singer, 2017), micro-phenomenology (e.g., Przyrembel and Singer, 2018; Petitmengin et al., 2019), or some other method. The studies could focus on meditation experts, layperson meditators, or both.

The current paper suggests two research questions that could be examined in participant-based studies: Do meditators conceive silence/quietness as reflecting purely the absence of thoughts and sounds, or do they see it as extending to the absence of other disturbances? And, how do meditators distinguish between silence/quietness and other features such as stillness?

One impediment to the participant-based research is that achieving the Shamatha goal-state is said to be extremely rare (Wallace, 1998/2005, p. 219, 2006a, p. 147). For Shamatha, researchers may be better off focusing on silence/quietness in advanced interim-states. In those states meditators are also said to be largely undisturbed (Supplementary Table S1, section “The Ten Stages”).

Another option for future research is to use evidence synthesis or participant-based studies to investigate silence/quietness as a feature of contentless experiences in other practices. The method and database presented in this paper could easily be extended to another tradition.

## Conclusion

The current paper is the first evidence synthesis based on expert texts to examine the experience of silence in Shamatha, TM and Stillness Meditation. Evidence synthesis is a robust and transparent scientific method. It was used in the present paper to generate a database containing descriptions of the meditation techniques and experiences extracted from the expert texts. The database runs to nearly 200 pages and is based on a detailed review of 135 publications. It has been coded – or organized – to identify individual features of the techniques and experiences, including silence/quietness and each other feature of the goal-states referred to in the expert texts.

Based on the database we made the following findings concerning the feature silence/quietness: (a) The experts frequently present it alongside other features; (b) The terms silence and quietness are often used without elaboration as to their precise meaning; (c) The experts suggest that it has a particular connection to stillness, and the absence of concepts, mental activity/noise, thoughts, and disturbance; and (d) They indicate that it is in some sense complete.

Analysis of the findings led to the following additional insights. The terms silence and quietness reflect the absence of thoughts and sounds. In general, the experts do not clearly indicate that the terms can extend to the absence of other disturbances, but they leave room for that interpretation. It is not clear from the expert texts exactly how the feature silence/quietness is distinct from other features such as stillness that may also reflect the absence of disturbances. As a separate matter, the absence of disturbances can be treated as central to silence/quietness, but silence/quietness is also connected to the other features of the goal-states in varying degrees.

As shown by the findings and insights above, the evidence synthesis identifies fine distinctions and areas of ambiguity within the expert texts. These lead to new research questions which can be investigated in participant-based studies or other text-based studies with the aim of achieving greater clarity. The findings, insights, and paths for future research in this paper demonstrate the value of investigating silence/quietness using evidence synthesis.

## Data Availability Statement

All datasets generated for this study are included in the article/Supplementary Material.

## Author Contributions

TW wrote the first draft of the manuscript and compiled the database. All authors contributed to the conception and design of the project, and to manuscript revision.

## Conflict of Interest

The authors declare that the research was conducted in the absence of any commercial or financial relationships that could be construed as a potential conflict of interest.

## Footnotes

1 In this paper we use the term *content* to refer to experiential qualities that meditators may report when describing what an experience was like. The term *contentless* refers to the absence of certain types of content, including thoughts, perceptions, and mental images. The term does not denote the absence of *all* forms of content. For example, meditators report that during contentless experiences they were conscious and awake, and this indicates that certain forms of content may be present in the experiences.

2 Wallace focuses principally on three types of Shamatha practice: *mindfulness of breathing*, *settling the mind in its natural state*, and *awareness of awareness*. He derives his presentation of mindfulness of breathing mainly from Tibetan and Theravadin Buddhist teachings (Wallace, 2005, p. 12). He bases his descriptions of the other two techniques primarily on teachings in the Tibetan Buddhist lineage known as Dzogchen (Wallace, 2001/2003, p. 119; Wallace, 2006a, p. 7; Van Waning, 2013/2014, p. 59).

3 Terms like traditional publications and traditional accounts are sometimes used by other researchers to refer only to old/historical accounts. Our use of the terms extends to modern texts.

4 In this paper we will focus on silence rather than stillness, but in examining silence we will also consider stillness to some degree.

5 Shallower goal-states in Stillness Meditation can involve very dull perceptions (see Supplementary Table S3, section “Reduced Awareness/Perception, Loss of Logical/Critical Faculties”). For simplicity, in this paper we will focus on the deeper goal-states where there are no perceptions.

6 In this paper the term *practice* includes both the meditation technique and experience.

7 One exception is that Pearson is a co-author of Travis and Pearson (2000), which we selected under the regime for Travis (see below). For simplicity, and since we are relying on Pearson (2013) for Pearson’s understanding, in this paper we will treat Travis and Pearson (2000) as a Travis publication not a Pearson one.

8 We focused on findings relating to the goal-state/s because *all* features of the goal-states needed to be captured, rather than merely features that were fundamental.

9 One definition of the term silence in the Oxford English Dictionary is “the absence of all sound or noise” (Oxford, 2020). The Merriam-Webster Dictionary includes an almost identical definition (“absence of sound or noise”) (Merriam-Webster, 2020).

10 This extended definition fits with other conventional understandings of the terms silence and quietness. For example, one definition of silence in the Oxford English Dictionary is “complete quietness or stillness,” and definitions of quietness include “calmness…”, and the “absence of… disturbance” (Oxford, 2020).
