# Supplementary material for: Silence in Shamatha, Transcendental, and Stillness Meditation: An Evidence Synthesis Based on Expert Texts
Source: Front Psychol. 2020 Jul 8;11:1259. doi: 10.3389/fpsyg.2020.01259 (PMC7360996; doi:10.3389/fpsyg.2020.01259)
Supplement: Supplementary file 1 [file Data_Sheet_1.pdf]

Appendix A: ENTREQ requirements

Table A1

ENTREQ Requirements

No.	Label	Requirement	Section/s in which requirement addressed
1	Aim	State the research question the synthesis addresses.	"Introduction" and "Reliability of Traditional Accounts"
2	Synthesis methodology	Identify the synthesis methodology or theoretical framework which underpins the synthesis, and describe the rationale for the choice of methodology.	"Use of Narrative Synthesis"
3	Approach to searching	Indicate whether the search was pre-planned or iterative.	"Selection of Publications"
4	Inclusion criteria	Specify the inclusion/exclusion criteria.	"Selection of Publications" and Appendix C
5	Data sources	Describe the information sources used and when the searches [were] conducted; provide the rationale for using the data sources.	"Selection of Publications" and Appendices B and C
6	Electronic search strategy	Describe the literature search.	"Selection of Publications" and Appendix B
7	Publication screening methods	Describe the process of publication screening and sifting.	"Selection of Publications" and Appendix C
8	Publication characteristics	Present the characteristics of the included publications.	Appendix F
9	Publication selection results	Identify the number of publications screened and provide reasons for publication exclusion.	"Selection of Publications" and Appendix D
10	Rationale for appraisal	Describe the rationale and approach used to appraise the included publications or selected findings.	"Basic Components and Reporting" and "Selection of Authors and Publications"
11	Appraisal items	State the tools, frameworks and criteria used to appraise the publications or selected findings.	"Selection of Authors and Publications"
12	Appraisal process	Indicate whether the appraisal was conducted independently by more than one reviewer and if consensus was required.	"Selection of Authors"
13	Appraisal results	Present results of the quality assessment and indicate which [publications], if any, were weighted/excluded based on the assessment and give the rationale.	"Selection of Authors and Publications"
14	Data extraction	Indicate which sections of the ... publications were analysed and how ... the data [were] extracted from the ... publications.	"Data Extraction" and Appendix F
15	Software	State the computer software used, if any.	"Extraction Process"
16	Number of reviewers	Identify who was involved in coding and analysis.	"Extraction Process"
17	Coding	Describe the process for coding of data.	"Synthesis"

18	Publication comparison	Describe how ... comparisons [were] made within and across publications.	“Synthesis”
19	Derivation of themes	Explain whether the process of deriving the themes or constructs was inductive or deductive.	“Synthesis”
20	Quotations	Provide quotations from the ... publications to illustrate themes/constructs, and identify whether the quotations were participant quotations or the ... interpretation [of the author of the publication].	“Results” and “Discussion”
21	Synthesis output	Present rich, compelling and useful results that go beyond a summary of the ... publications.	“Results” and “Discussion”

Note: The wording in the “Label” and “Requirements” columns is from the ENTREQ checklist. The terms “study” and “studies” in the ENTREQ checklist have been replaced with “publication” and “publications” respectively, to reflect the nature of the material that was reviewed in the present evidence synthesis.
