# Supplementary material for: Silence in Shamatha, Transcendental, and Stillness Meditation: An Evidence Synthesis Based on Expert Texts
Source: Front Psychol. 2020 Jul 8;11:1259. doi: 10.3389/fpsyg.2020.01259 (PMC7360996; doi:10.3389/fpsyg.2020.01259)
Supplement: Supplementary file 2 [file Data_Sheet_2.pdf]

Appendix B: Searches

Table B1
Wallace Books Searches

Database	Date	Search
Google Scholar	15 January 2018	Search for "(shamatha OR samatha) author:wallace"
Amazon U.S.	2 February 2018	Search using indexed author term "B. Alan Wallace"

Table B2
Wallace Other Publications Searches

Database	Date	Search
Google Scholar	28 January 2018	Search for "(shamatha OR samatha) author:wallace"
Medline	28 January 2018	Advanced search for "wallace" (author field; with indexed terms "wallace", "wallace a", "wallace b", "wallace ba"), combined with advanced search for "(shamatha OR samatha)" (keywords field)
PsycInfo	28 January 2018	Advanced search for "wallace" (author field; with indexed terms "wallace a", "wallace alan", "wallace b", "wallace b alan", "wallace bruce", "wallace bruce alan"), combined with advanced search for "(shamatha OR samatha)" (keywords field)
Embase	28 January 2018	Advanced search for "wallace" (author field; with indexed terms "wallace", "wallace a", "wallace b", "wallace ba") combined with advanced search for "(shamatha OR samatha)" (keywords field)
PubMed	28 January 2018	Advanced search for "wallace" (author field) and "(shamatha OR shamatha)" (all fields)
Web of Science	28 January 2018	Advanced search for "wallace" (author field) and "(shamatha OR samatha)" (topic field)
Scopus	28 January 2018	Search for "wallace" (author field) and "shamatha OR samatha" (author, abstract, keywords field)

Table B3
Shear Books and Other Publications Searches

Database	Date	Search
Google Scholar	8 January 2018	Search for "'transcendental meditation' author:shear"
Medline	8 January 2018	Advanced search for "shear" (author field; with indexed term "shear j"), combined with advanced search for "transcendental meditation" (keywords field)
PsycInfo	8 January 2018	Advanced search for "shear" (author field; with indexed term "shear jonathan"), combined with advanced search for "transcendental meditation" (keywords field)
Embase	8 January 2018	Advanced search for "shear" (author field; with indexed term "shear j") combined with advanced search for "transcendental meditation" (keywords field; with indexed term "transcendental meditation")
PubMed	8 January 2018	Advanced search for "shear" (author field) and "transcendental meditation" (all fields)
Web of Science	8 January 2018	Advanced search for "shear" (author field) and "transcendental meditation" (topic field)

Scopus	8 January 2018	Search for “shear” (author field) and “transcendental meditation” (author, abstract, keywords field)
PhilPapers	8 January 2018	Advanced search for “shear” and “transcendental meditation”
Philosopher’s Index	8 January 2018	Search for “shear” (author field) and “transcendental meditation” (all fields)
Amazon U.S.	8 January 2018	Search for “jonathan shear”

Table B4
Travis Books and Other Publications Searches

Database	Date	Search[a]
Google Scholar	8 January 2018	Search for “‘transcendental meditation’ author:travis”
Medline	8 January 2018	Advanced search for “travis” (author field; with indexed terms “travis f” and “travis ft”), combined with advanced search for “transcendental meditation” (keywords field)
PsycInfo	8 January 2018	Advanced search for “travis” (author field; with indexed terms “travis”, “travis fred”, “travis frederick”, “travis frederick t”, “travis fredrick”), combined with advanced search for “transcendental meditation” (keywords field)
Embase	8 January 2018	Advanced search for “travis” (author field; with indexed terms “travis”, “travis f”, “travis ft”) combined with advanced search for “transcendental meditation” (keywords field; with indexed term “transcendental meditation”)
PubMed	8 January 2018	Advanced search for “travis” (author field) and “transcendental meditation” (all fields)
Web of Science	8 January 2018	Advanced search for “travis” (author field) and “transcendental meditation” (topic field)
Scopus	8 January 2018	Search for “travis” (author field) and “transcendental meditation” (author, abstract, keywords field)
Amazon U.S.	9 January 2018	Search for “travis ‘transcendental meditation’”

[a]All searches were narrowed to 2000 onwards

Table B5
Meares Other Publications Searches

Database	Date	Search[a]
Google Scholar	31 January 2018	Search for “(meditation or hypnosis) author:meares”<br>Search for “author:a meares”
Medline	31 January 2018	Advanced search for “meares” (author field; with indexed term “meares a”)
PsycInfo	31 January 2018	Advanced search for “meares” (author field; with indexed terms “meares a” and “meares ainslie”)
Embase	31 January 2018	Advanced search for “meares” (author field; with indexed term “meares a”)
PubMed	31 January 2018	Advanced search for “meares” (author field)
Web of Science	31 January 2018	Advanced search for “meares” (author field) and “meditation” or “hypnosis” (topic field)

Scopus	31 January 2018	Search for “meares” (author field) and “meditation OR hypnosis” (author, abstract, keywords field)
Discovery	31 January 2018	Advanced search for “meares” (author field) and “(meditation or hypnosis)” (all fields)

[a] All searches were narrowed to 1957 onwards

Note: Unless otherwise specified, the searches in Tables B1 to B5 covered all years of the relevant database (i.e., the database inception date to the date of the search).
