# Supplementary material for: Silence in Shamatha, Transcendental, and Stillness Meditation: An Evidence Synthesis Based on Expert Texts
Source: Front Psychol. 2020 Jul 8;11:1259. doi: 10.3389/fpsyg.2020.01259 (PMC7360996; doi:10.3389/fpsyg.2020.01259)
Supplement: Supplementary file 3 [file Data_Sheet_3.pdf]

Appendix C: Process for Applying the Eligibility Criteria for Each Relevant Author

Shamatha

Wallace. The first step in the selection of Wallace's publications was to make a list of books and a list of other publications that he had authored or co-authored.

The books list incorporated all Wallace's books:

- identified via the searches described in Table B1; or
- included in the lists of academic and popular books on his website (www.alanwallace.org) as at 15 January 2018,

other than books:

- translated by Wallace but not including detailed commentaries by him; or
- for which he contributed only one or two chapters.[1]

As discussed in the "Selection of Publications" section, upon the publication of Wallace's book *Fathoming the Mind* in October 2018, it was added to the books list.

The other publications list incorporated all Wallace's other publications:

- identified via the searches described in Table B2; or
- listed on the "Academic essays" page of his website as at 28 January 2018.

Eligibility criteria were then applied to the list of books and the list of other publications. The author-specific inclusion criteria for the books list were that the book includes substantial comment on:

- what Buddhist shamatha technique[2] involves; and/or
- states attained in Buddhist shamatha practice (other than general statements about a state of attention or relaxation),

where substantial comment was indicated by two or more paragraphs (whether together or apart) focused mainly on those topics. The author-specific inclusion criteria for the other publications were that the publication includes substantial comment of the kind described above, or an empirical assessment of the subjective experience in one or more Buddhist shamatha sessions based on first-person reports of research participants.

---

1 These chapters were considered as part of Wallace's other publications, as described below.

2 The current project examines the shamatha mindfulness of breathing, settling the mind in its natural state, and awareness of awareness practices focused on by Wallace ("Shamatha"), rather than Buddhist shamatha practices more broadly. However, Wallace sometimes makes comments about the broader shamatha practices that are relevant to Shamatha. For this reason it was not required that the passages in the publications be about Shamatha specifically.

As explained in the "Selection of Publications" section, the rationale for the main inclusion criterion[3] was that it selected Wallace's publications with more than minimal relevant content (i.e., at least two paragraphs). In the "Selection of Authors" section it was noted that the scoping reading indicated that Wallace's publications (there meaning those with more than the minimal relevant content) tend to each add some important detail, rather than merely repeating content. For that reason it was decided that it was not sensible to try to exclude further publications on the grounds that they were likely to duplicate material. Exclusion criteria were therefore not required.

The eligibility criteria for all publications were applied by skimming the full text of each publication, and, where there was any doubt about how the criteria applied, reading in detail the passages that stood out as potentially relevant. Where electronic copies were available, electronic searches for relevant words[4] were also conducted, as a safeguard to ensure that all potentially relevant passages had been identified.

Transcendental Meditation

Shear. For Shear, the first step was to prepare a list of books and other publications that he had authored or co-authored. It incorporated all publications:

- identified via the searches described in Table B3; or
- listed on his profile page on the Virginia Commonwealth University website (https://philosophy.vcu.edu) as at 8 January 2018.

Next the eligibility criteria were applied to the list. The author-specific inclusion criterion was that the publication was from 1990 onwards. The exclusion criteria were that:

- the citation for the publication in the relevant database indicates that the text was published in an Indian journal and/or by an Indian publisher;[5] or
- the body of the publication does not include a reference to TM.

The rationale for the inclusion criterion is provided in the "Selection of Publications" section. Indian publications were excluded (see first exclusion criterion) because the scoping reading indicated that they tended to duplicate Shear's main understandings of TM that were incorporated in his large suite of Western publications. Criteria based on geography are common in evidence syntheses (Gough et al., 2013). Publications that did not refer to TM were excluded (see second criterion) on the basis that they were not relevant.

---

3 Meaning the criterion reading "includes substantial comment ...".
4 For example, shamatha, samatha, quiescence, attention, substrate, and bhavanga.
5 Indian publishers that are part of an international publisher were still classed as Indian.

Other than the second exclusion criterion, eligibility criteria were applied on the basis of the citation in the relevant database. Application of the second exclusion criterion involved a search for the term "Transcendental Meditation" in the full text.

Travis. For Travis, the first step was to make a list of books and other publications that he had authored or co-authored from 2000 onwards. That year was selected as the cut-off date on the basis that it was the year Travis and Pearson (2000) was published. Travis and Pearson included a participant-based study of the subjective experience in TM that is frequently relied on by the TM authors. Since the present evidence synthesis process involved obtaining a sample of Travis' publications (rather than all of them), it seemed sensible to focus on his materials prepared with knowledge of that study. A similar approach was taken in Bailey et al. (2015, pp. xiii, 18), in that their evidence synthesis was restricted to materials after the year of a seminal paper.

The Travis list incorporated all publications from 2000 onwards:

- identified via the searches described in Table B4; or
- listed on his profile page on the Maharishi University of Management website (www.mum.edu) as at 10 February 2018, provided not marked as "in press".

Travis' publications tend to be either participant-based empirical studies, or theoretical, conceptual or review papers. Participant-based studies were not the main focus of the current project. The aim in the project was to examine Travis' publications to identify: (a) his understandings of TM that are separate to the participant-based findings; and (b) any of his participant-based findings about the subjective experience of the TM goal-state/s that are relied on by him or may be relied on by other TM authors.

To ascertain the understandings of TM referred to in point (a), theoretical, conceptual and review papers were focused on, rather than the individual empirical studies. Scoping reading indicated that those papers tended to summarize a range of empirical studies, and to show how they supported Travis' pre-existing understandings of the practices. The papers therefore appeared to provide an information-rich source of those understandings. Theoretical, conceptual and review papers were only available in the period before 2017. As such, for that period those papers were focused on, and from 2017 the individual empirical studies were selected. To address point (b) above, an additional aim was to select any of Travis' participant-based studies about the subjective experience in TM.

The eligibility criteria applied to the list of Travis' publications were designed to implement the approach described in the paragraph above. One set of criteria was used for publications before 2017, and a slightly modified set for the period from 2017 onwards. The author-specific inclusion criteria for materials before 2017 were that the publication:

- is either:
    - non-empirical (i.e., a theoretical, conceptual, or review publication); or
    - empirical, and includes an empirical assessment of the subjective experience during one or more TM sessions based on first-person reports of research participants; and
- has as its main focus either:
    - examining states attained in, or flowing from, TM practice; or
    - the relationship or distinction between mindfulness practices and TM.

The exclusion criteria were that the publication:

- has as its main focus a specific population (e.g., leaders, musicians, refugees, children with a disorder) rather than a general population (e.g., adults, students, meditators);
- is a book aimed principally at a popular (as opposed to academic or expert) readership; or
- is a journal article labelled a supplement.

For the period from 2017 up to the time when the searches for the publications were conducted (8 and 9 February 2018), the same eligibility criteria as above were used, except that the first inclusion criterion[6] was omitted. That ensured that Travis' individual empirical studies were selected in that period, which was considered appropriate given the absence in that period of theoretical, conceptual or review papers.

The key criterion ensuring relevance was the provision that the main focus of the publication be examining states attained in or flowing from TM practice. The scoping reading indicated that Travis tended to set out his understandings of TM in most detail in those publications. The main rationale for the three exclusion criteria was that the publications that they refer to likely merely duplicate understandings of the practice and experiences contained in the core materials captured by the inclusion criteria. For example, publications focused on particular populations (as referred to in the first exclusion criterion) tend to be mainly concerned with the effects of practising TM. As such, they tend not to provide details of the TM technique or experience that add anything substantive to publications that are focused on those elements.

[6] Meaning the criterion reading "is either ... [n]on-empirical ... ; or [e]mpirical ...".

For all of the Travis materials a two-stage process was used to apply the eligibility criteria (Gough et al., 2012/2017; Petticrew and Roberts, 2006). The first stage involved using citations, titles and abstracts to identify publications that clearly failed to meet an inclusion criterion or that clearly satisfied an exclusion criterion. The second stage involved applying the criteria to all remaining publications on the basis of their full text.

Stillness Meditation

Meares. To select Meares' publications the starting point was to make a list of his books, and a list of his other publications, from 1957 onwards. That year was selected on the understanding that it preceded the development of Stillness Meditation, and would therefore ensure that all of Meares' publications relating to the practice would be captured.[7]

The books list incorporated all Meares' books from 1957 onwards:

- included in the Bruhn (2016, p. 36) list;
- included in the Meares (1989, p. iv) list; or
- included on the Wikipedia page "List of works by Ainslie Meares" as at 31 January 2018.

The other publications list incorporated all of his other publications from 1957 onwards:

- cited in Bruhn (2016);
- included in the Meares (1989, pp. 148-149) list;
- included in the Meares (1984, pp. 151-152) list;
- included on the Wikipedia page above as at 31 January 2018;
- identified via the searches described in Table B5; or
- cited or referenced in the publications identified from the above five sources.

Eligibility criteria were then applied to the two lists. The only author-specific inclusion criterion for the books was that they were published from 1967 onwards. This criterion was adopted on the basis that Meares' book, *Relief Without Drugs* (Meares, 1967/1968), was published in that year, and scoping reading of the earlier materials on the lists indicated that it provided the first comprehensive description of Stillness Meditation. The author-specific inclusion criteria for the other publications were that the publication:

- was published from 1967 onwards; and

[7] Meares' central theory with respect to Stillness Meditation was that the practice involves the mind functioning in a more simple and primitive manner (Meares, 1967/1968; see Table S3, 1.5 for further detail). He referred to this as *atavistic regression*. He first published the theory in 1957, in relation to his work on hypnosis (Meares, 1957). Since it is recognized that the initial publication of the theory preceded key events in the development of Stillness Meditation (Bruhn, 2016; Meares, 1969b), 1957 was considered an appropriate cut-off for ensuring that all of Meares' relevant materials were identified.

- refers to a practice that might reasonably be identified as Stillness Meditation.

As noted in the "Selection of Publications" section, where there was any doubt about whether the second criterion applied to a publication, the publication was included.

The effect of the inclusion criteria was to excise a large number of publications that were not relevant. The scoping reading indicated that most if not all of the remaining publications contained at least some relevant content. No features of the publications stood out that could be reliably used to exclude materials likely to merely duplicate content, and for that reason exclusion criteria were not utilized. The consequence of this was that all of Meares' relevant publications (whether duplicating or not) were included in the evidence synthesis. Inclusion of that comprehensive set of Meares' publications seemed appropriate, seeing as Meares is the central figure in Stillness Meditation and there has been little research on the practice.

The criterion relating to the year of publication was applied for all materials on the basis of the citation in the relevant database or source. The criterion concerning the nature of the practice was applied by examining the full text of the publication.

McKinnon. McKinnon's publications are all books, rather than book chapters or journal articles. The books list for McKinnon incorporated all of her books:

- included in the McKinnon (2016, p. 296) list; or
- listed on the Stillness Meditation Therapy Centre website (www.stillnessmeditation.com.au) as at 16 February 2018 (other than those in the childrens' books section).

The only author-specific inclusion criterion was that the publication was aimed principally at adults rather than children. The rationale for this criterion was that publications for children were likely to merely duplicate relevant material in those for adults.

Scoping reading made clear that each of McKinnon's four books for adults contained at least some material that was relevant and that did not duplicate material in the other books. On that basis there was no need to rely on exclusion criteria.

The inclusion criterion was applied on the basis of citation and/or blurb.

References

Bailey, C., Madden, A., Alfes, K., Fletcher, L., Robinson, D., Holmes, J., et al. (2015). Evaluating the evidence on employee engagement and its potential benefits to NHS staff: A narrative synthesis of the literature. *Health Services and Delivery Research* 3, 1–424. doi:10.3310/hsdr03260.

Bruhn, O. (2016). *Ainslie Meares on meditation*. Melbourne, Australia: Author.

Gough, D., Oliver, S., and Thomas, J. (2013). *Learning from research: Systematic reviews for informing policy decisions*. London: Nesta.

Gough, D., Oliver, S., and Thomas, J. (2012/2017). *An introduction to systematic reviews*. 2nd ed. London, United Kingdom: Sage.

McKinnon, P. (1983/2016). *In stillness conquer fear: How to break free from anxiety and phobias*. Rev. ed. Melbourne, Australia: Garratt.

Meares, A. (1957). A working hypothesis as to the nature of hypnosis. *AMA Archives of Neurology & Psychiatry* 77, 549–555.

Meares, A. (1967/1968). *Relief without drugs: The self-management of tension, anxiety, and pain*. London, United Kingdom: Souvenir Press.

Meares, A. (1969b). *Strange places and simple truths: A psychiatrist looks at eastern mysticism*. United Kingdom, London: Souvenir Press.

Meares, A. (1984). *A way of doctoring*. Melbourne, Australia: Hill of Content.

Meares, A. (1989). *A better life: The guide to meditation*. Melbourne, Australia: Greenhouse.

Petticrew, M., and Roberts, H. (2006). *Systematic reviews in the social sciences: A practical guide*. Malden, MA: Blackwell.

Travis, F., and Pearson, C. (2000). Pure consciousness: Distinct phenomenological and physiological correlates of “consciousness itself”. *International Journal of Neuroscience* 100, 77–89. doi:10.3109/00207450008999678.
