# Supplementary material for: Silence in Shamatha, Transcendental, and Stillness Meditation: An Evidence Synthesis Based on Expert Texts
Source: Front Psychol. 2020 Jul 8;11:1259. doi: 10.3389/fpsyg.2020.01259 (PMC7360996; doi:10.3389/fpsyg.2020.01259)
Supplement: Supplementary file 4 [file Data_Sheet_4.pdf]

Appendix D: Publications Selection Flowcharts

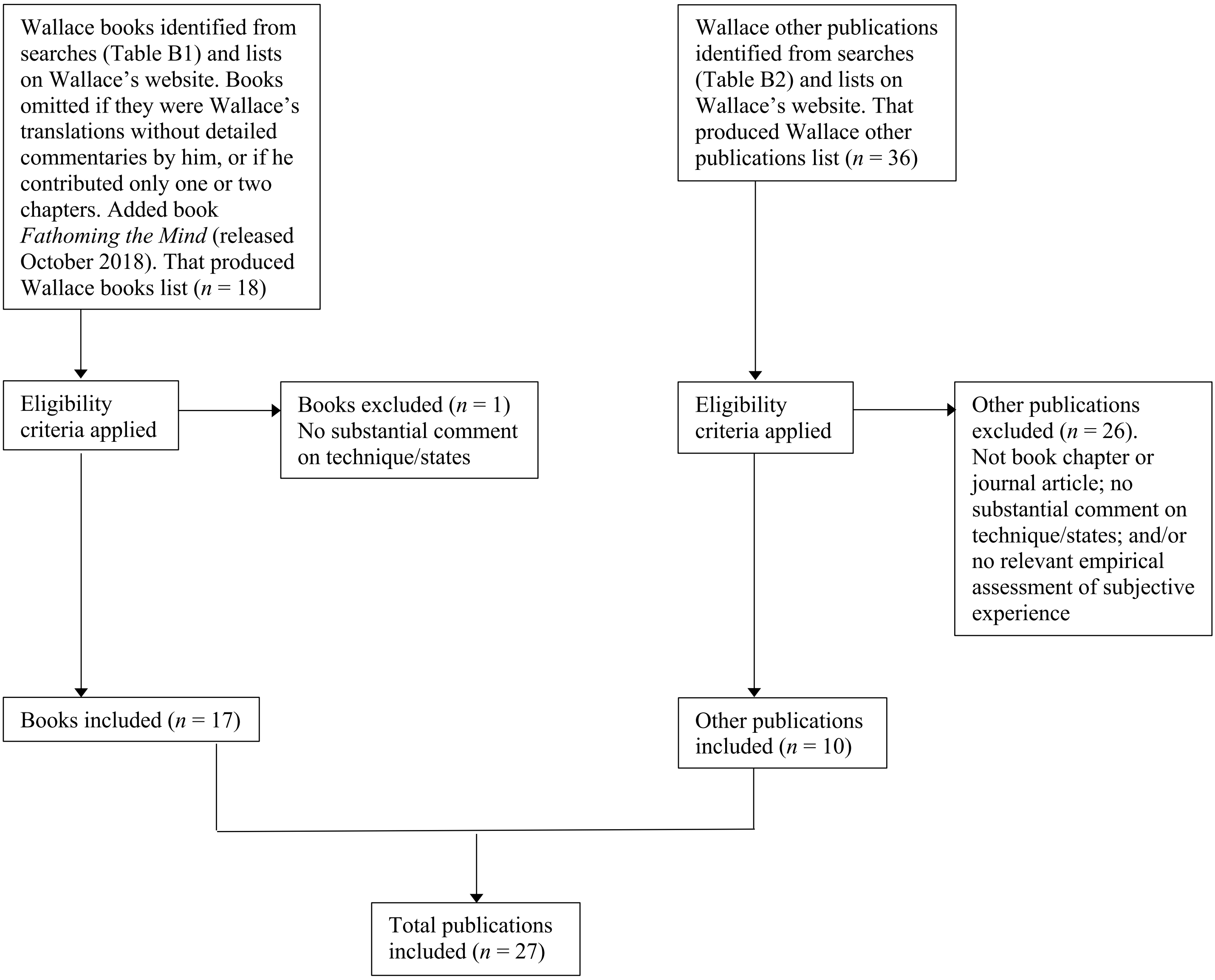

Figure D1. Flowchart summarizing the Shamatha publications selection process.

Note: A small number of the Shamatha, TM and Stillness Meditation publications have been published under multiple titles. In each of these cases of duplication, one publication alone was focused on. For ease of comprehension, the publication numbers in Figures D1 to D3 do not include the duplicates.

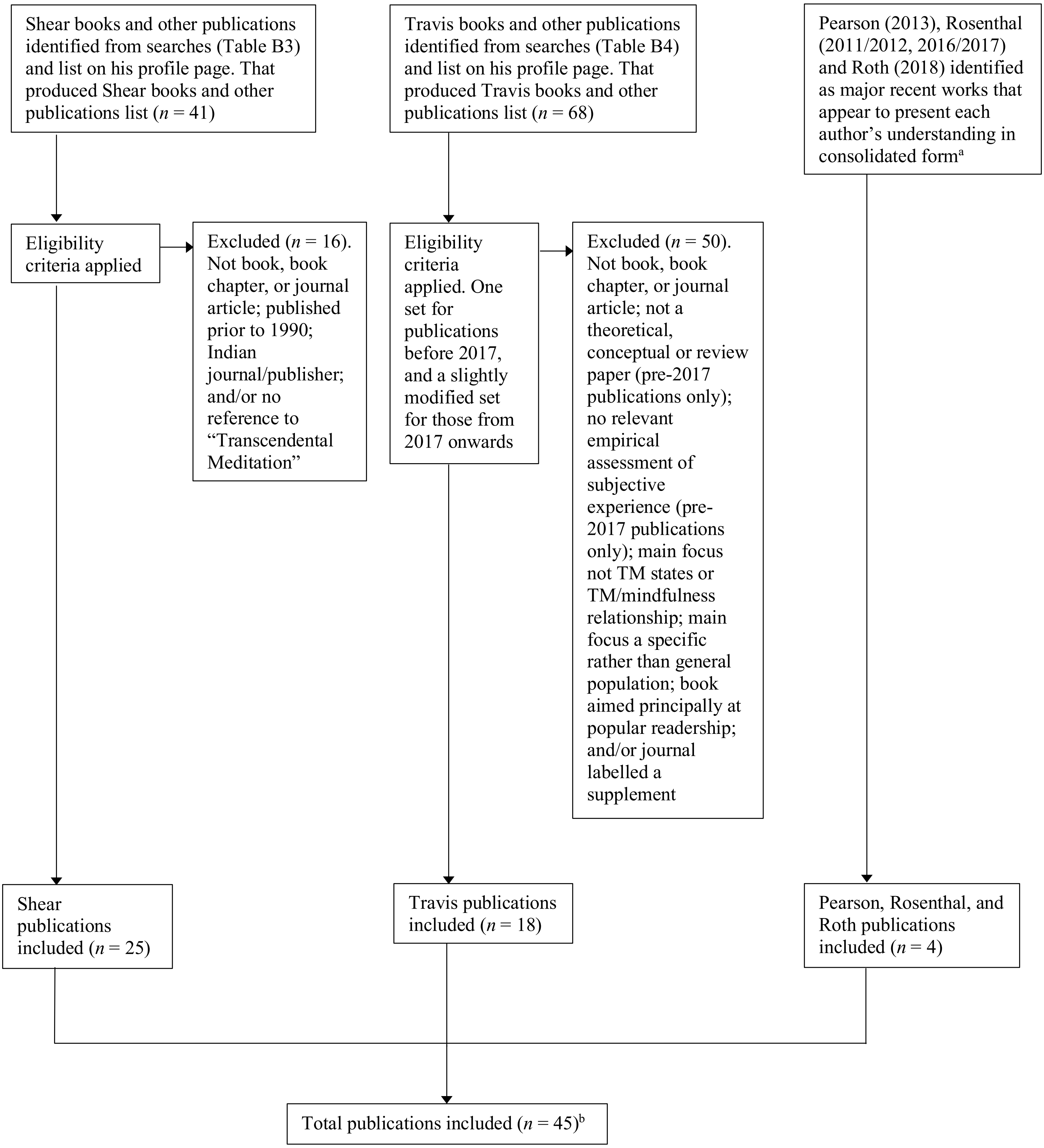

Figure D2. Flowchart summarizing the TM publications selection process. [a] It was not necessary to apply eligibility criteria to these four publications. The purpose of the criteria was to identify samples of publications revealing authors' understandings of the practices. For these three authors the four publications already comprised an appropriate sample (see section "Selection of Publications"). [b] Two publications were selected for both Shear and Travis. These were only counted once in calculating the total TM publications.

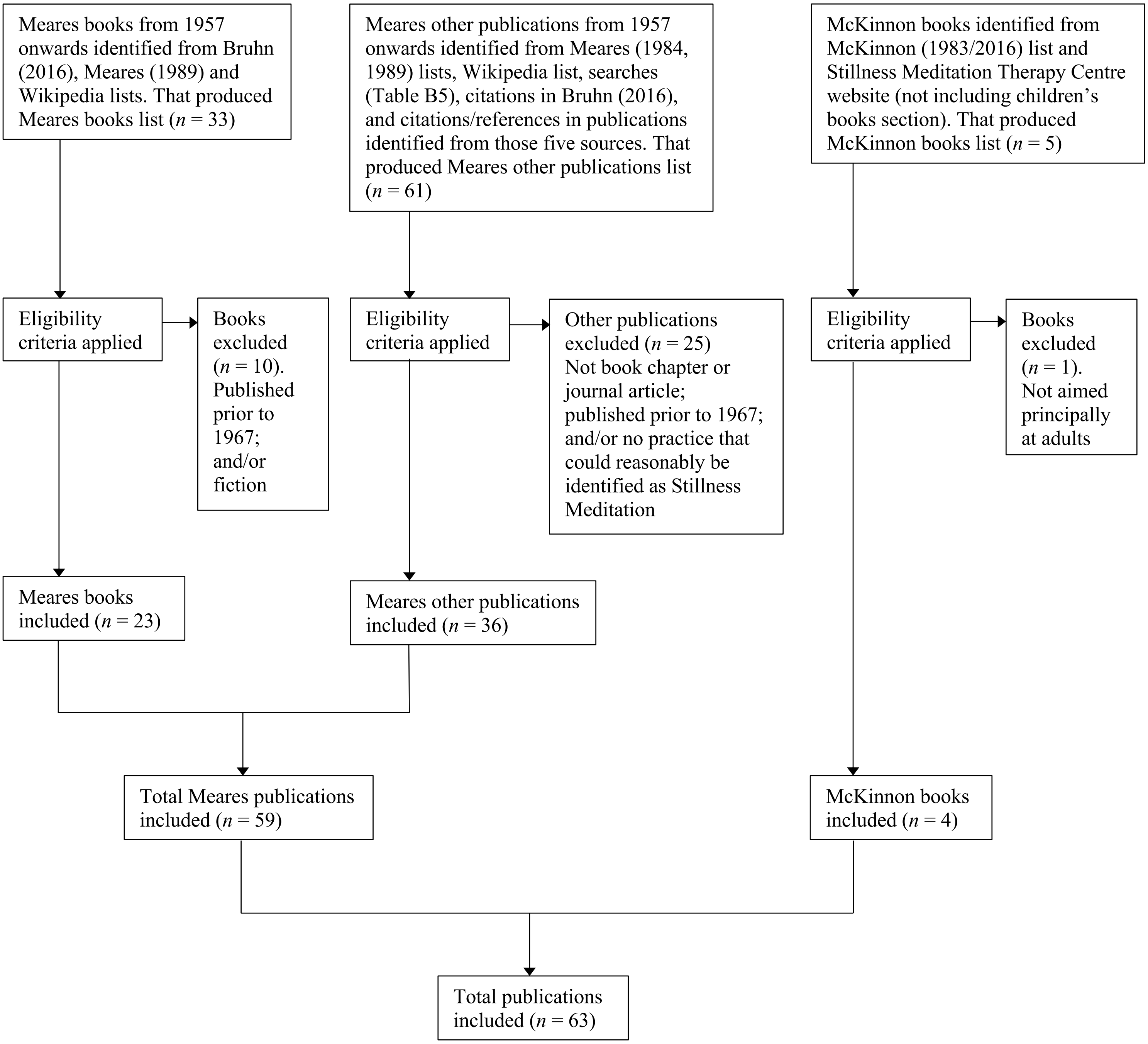

Figure D3. Flowchart summarizing the Stillness Meditation publications selection process.

References

Bruhn, O. (2016). *Ainslie Meares on meditation*. Melbourne, Australia: Author.

McKinnon, P. (1983/2016). *In stillness conquer fear: How to break free from anxiety and phobias*. Rev. ed. Melbourne, Australia: Garratt.

Meares, A. (1984). *A way of doctoring*. Melbourne, Australia: Hill of Content.

Meares, A. (1989). *A better life: The guide to meditation*. Melbourne, Australia: Greenhouse.

Pearson, C. (2013). *The supreme awakening: Experiences of enlightenment throughout time – and how you can cultivate them*. Fairfield, IA: Maharishi University of Management Press.

Rosenthal, N. E. (2011/2012). *Transcendence: Healing and transformation through Transcendental Meditation*. New York, NY: Penguin.

Rosenthal, N. E. (2016/2017). *Super Mind: How to boost performance and live a richer and happier life through Transcendental Meditation*. New York, NY: Penguin.

Roth, B. (2018). *Strength in stillness: The power of Transcendental Meditation*. London, United Kingdom: Simon & Schuster.

Wallace, B. A. (2018). *Fathoming the mind: Inquiry and insight in Düdjom Lingpa's Vajra Essence*. Somerville, MA: Wisdom.
