# Supplementary material for: Silence in Shamatha, Transcendental, and Stillness Meditation: An Evidence Synthesis Based on Expert Texts
Source: Front Psychol. 2020 Jul 8;11:1259. doi: 10.3389/fpsyg.2020.01259 (PMC7360996; doi:10.3389/fpsyg.2020.01259)
Supplement: Supplementary file 5 [file Data_Sheet_5.pdf]

## Appendix E: Guidelines on Application of Eligibility Criteria for Travis and Wallace

### Travis Criteria

**Main focus of the publication.** One of the author-specific inclusion criteria for the Travis publications is that the main focus is: "examining states attained in, or flowing from, TM practice"; or "the relationship or distinction between mindfulness practices and TM". In some cases the whole publication may be about one or both of these two points (the "target content"). In those cases it will be obvious that that content is the main focus of the publication. In other cases the publication may address some broader issue or topic. That issue or topic may not itself be the target content, but the publication may address it by relying heavily on that content. The target content is therefore pivotal/critical: The broader issue/topic is viewed through that prism. In that scenario the target content will be treated as the main focus of the publication. In a third category of publications, the target content will only comprise one aspect, which is no more important than one or more other aspects. The target content in that case will not be regarded as the main focus.

**States attained in, or flowing from, TM practice.** The phrase "examining states attained in, or flowing from, TM practice" in the inclusion criterion above is to be interpreted in a broad manner. It is not necessary for the content to be about the subjective experience of those states, or third-person measures of them such as EEG or neuroimaging. For example, if the content discusses the importance of the states, or their effects in daily life, that will be treated as sufficient. As a separate matter, the phrase "states ... flowing from ... TM practice" is intended to refer to the experience outside a TM session, where states achieved within TM practice are said to become somehow integrated with normal waking or sleeping experience. These integrated states are often referred to in TM publications as Cosmic Consciousness.

### Wallace Criteria

**States attained in Buddhist shamatha practice.** One of the author-specific inclusion criteria for the Wallace publications is that there is substantial comment on: "what Buddhist shamatha technique involves"; and/or "states attained in Buddhist shamatha practice (other than general statements about a state of relaxation or attention)". Wallace considers that the goal-state achieved in Shamatha practice is the same as the state referred to as *bhavanga* in Theravadin Buddhist practices (Table S1, 2.4; Wallace, 2005, 2006a, 2006b). Consequently, discussion about the bhavanga in a publication will be treated as comment on "states attained in Buddhist shamatha practice".

**General statements about relaxation or attention.** The empirical studies which Wallace has co-authored typically include a short description of shamatha meditation, as well as various general passages dealing with the development of attention which are not referenced to specific stages in those practices. Those general passages might, for example, address findings about attention made in other meditation studies or in broader cognitive science. These passages are examples of "general statements about a state of relaxation or attention" as referred to in the inclusion criterion above. They will therefore not be treated as comment on "states attained in Buddhist shamatha practice".

**Main focus.** The inclusion criterion states that "substantial comment" will be "indicated by two or more paragraphs (whether together or apart) focused mainly on [the prescribed] topics", namely the Buddhist shamatha technique or states. The phrase "focused mainly on" in this context has its ordinary meaning. If, for example, the prescribed topics are the subject of only one or two sentences in a paragraph of some length, those topics will not be treated as the main focus of the paragraph.

## References

Wallace, B. A. (2005). *Genuine happiness: Meditation as the path to fulfillment*. Hoboken, NJ: John Wiley.

Wallace, B. A. (2006a). *The attention revolution: Unlocking the power of the focused mind*. Somerville, MA: Wisdom.

Wallace, B. A. (2006b). "Vacuum states of consciousness: A Tibetan Buddhist view," in *Buddhist thought and applied psychological research: Transcending the boundaries*, eds. D. K. Nauriyal, M. S. Drummond, and Y. B. Lal (Oxon, United Kingdom: Routledge), 112–121.
