# Supplementary material for: Silence in Shamatha, Transcendental, and Stillness Meditation: An Evidence Synthesis Based on Expert Texts
Source: Front Psychol. 2020 Jul 8;11:1259. doi: 10.3389/fpsyg.2020.01259 (PMC7360996; doi:10.3389/fpsyg.2020.01259)
Supplement: Supplementary file 6 [file Data_Sheet_6.pdf]

Appendix F: Final Publications Selected

Table F1

Publications Selected for Shamatha

Citation[a]	Title	Publication type	Section/s reviewed
Wallace and Wilhelm (1993)	Tibetan Buddhism from the ground up: A practical approach for modern life	Book	Pages ix-x, 42-43, 105-118, 135-137, 148-151[b]
Wallace (1999a)	The Buddhist tradition of Samatha: Methods for refining and examining consciousness	Journal article	All
Wallace (1999b)	Training the attention and exploring consciousness in Tibetan Buddhism	Book chapter	All
Wallace (2000)	The taboo of subjectivity. Toward a new science of consciousness	Book	Pages 8-13, 103-120[c]
Wallace (2001a)	Intersubjectivity in Indo-Tibetan Buddhism	Journal article	All
Wallace (2001b)	The potential of emptiness: Vacuum states in physics and consciousness	Journal article	All
Wallace (2001/2003)	Buddhism with an attitude: The Tibetan seven-point mind-training	Book	Pages 7-11, 65-67, 76-154, 229-235, 273-278[b]
Wallace (1989/2003)	Choosing reality: A Buddhist view of physics and the mind	Book	Pages 149-151, 191-199[b]
Wallace (2004)	Scientific and Buddhist views of energy	Journal article	All
Wallace (1998/2005)	Balancing the mind: A Tibetan Buddhist approach to refining attention	Book	All
Wallace (2005)	Genuine happiness: Meditation as the path to fulfillment	Book	All
Wallace (2006a)	The attention revolution: Unlocking the power of the focused mind	Book	All
Wallace (2006b)	Vacuum states of consciousness: A Tibetan Buddhist view	Book chapter	All
Wallace and Shapiro (2006)	Mental balance and well-being: Building bridges between Buddhism and western psychology	Journal article	All
Wallace (2007a)	Contemplative science: Where Buddhism and neuroscience converge	Book	Pages 11-27, 58-108, 163-165[c, d]
Wallace (2007b)	Hidden dimensions: The unification of physics and consciousness	Book	Pages vii-xi, 36-49, 58-69, 85-121[c]
Wallace and Hodel (2008)	Embracing mind: The common ground of science & spirituality	Book	All
Wallace (2010)	The four immeasurables: Practices to open the heart	Book	Pages 31-86, 163-183[b]
Wallace (2011a)	Minding closely: The four applications of mindfulness	Book	All
Wallace (2011b)	Stilling the mind: Shamatha teachings from Düdjom Lingpa's Vajra Essence	Book	All
Desbordes et al. (2012)	Effects of mindful-attention and compassion meditation training on amygdala response to emotional stimuli in an ordinary, non-meditative state	Journal article	All
Wallace (2012)	Dreaming yourself awake: Lucid dreaming and Tibetan dream yoga for insight and transformation	Book	Pages 1-18, 135-150, 159-163[c]
Wallace (2012/2014)	Meditations of a Buddhist skeptic: A manifesto for the mind sciences and contemplative practice	Book	All
Wallace (2009/2014)	Mind in the balance: Meditation in science, Buddhism, and Christianity	Book	All
Kozasa et al. (2015)	Effects of a 9-day shamatha Buddhist meditation retreat on attention, mindfulness and self-compassion in participants with a broad range of meditation experience	Journal article	All

Zanesco et al. (2016)	Meditation training influences mind wandering and mindless reading	Journal article	All
Wallace (2018)	Fathoming the mind: Inquiry and insight in Düdjom Lingpa's Vajra Essence	Book	All

Note. [a] Publications by Wallace, as author or co-author, are ordered by year of publication (earliest to latest) of the version reviewed. [b] Content relating to the Shamatha practices, plus contextual material. [c] Relevant content relating to Tibetan Buddhism (including Dzogchen practices), plus contextual material. [d] Chapter 6 (*Worlds of Intersubjectivity*; pp. 109-134) was not reviewed, as the book notes (p. 180) that it was first published as Wallace (2001a), a publication that was separately reviewed. Similarly, Chapter 7 (*Samatha: The Contemplative Refinement of Attention*; pp. 135-148) was not reviewed, as the book notes (p. 182) that it was first published as Wallace (1999a), which was separately reviewed.

Table F2

Publications Selected for TM

Citation[a]	Title	Publication type	Section/s reviewed
Pearson			
Pearson (2013)	The supreme awakening: Experiences of enlightenment throughout time and how you can cultivate them	Book	All material relating to TM specifically (as distinct from material concerning experiences in other traditions, practices, or contexts)
Rosenthal			
Rosenthal (2011/2012)	Transcendence: Healing and transformation through Transcendental Meditation	Book	All
Rosenthal (2016/2017)	Super Mind: How to boost performance and live a richer and happier life through Transcendental Meditation	Book	All
Roth			
Roth (2018)	Strength in stillness: The power of Transcendental Meditation	Book	All
Shear			
Shear (1990a)	Mystical experience, hermeneutics, and rationality	Journal article	All
Shear (1990b)	The inner dimension: Philosophy and the experience of consciousness	Book	All
Shear (1994)	On mystical experiences as support for the perennial philosophy	Journal article	All
Shear (1995)	Mystical knowledge?	Journal article	All
Shear (1996a)	Back and forth with Huston Smith	Journal article	All
Shear (1996b)	On the existence of a culture independent core component of self	Book chapter	All
Shear (1995/1997a)	Introduction	Book chapter	All
Shear (1995/1997b)[b]	The hard problem: Closing the empirical gap	Book chapter	All
Gallagher and Shear (1999)	Editors' introduction	Book chapter	All
Shear (1998/1999)[c]	Experiential clarification of the problem of self	Book chapter	All
Shear and Jevning (1999a)[d]	Pure consciousness: Scientific exploration of meditation techniques	Journal article	All
Varela and Shear (1999b)[e]	First-person methodologies: What, why, how?	Journal article	All

Shear (2002)	Ethics and the experience of happiness	Book chapter	All
Shear (2001/2002)	Experimental studies of meditation and consciousness	Book chapter	All
Shear (2004)	Mysticism and scientific naturalism	Journal article	All
Shear (2006a)	Conclusion	Book chapter	All
Shear (2006b)	Introduction	Book chapter	All
Shear (2006c)	Transcendental Meditation	Book chapter	All
Shear (2007)	Eastern methods of investigating mind and consciousness	Book chapter	All
Travis and Shear (2010a)[f]	Focused attention, open monitoring and automatic self-transcending: Categories to organize meditations from Vedic, Buddhist and Chinese traditions	Journal article	All
Travis and Shear (2010b)[f]	Reply to Josipovic: Duality and non-duality in meditation research	Journal article	All
Shear (2011a)	Eastern approaches to altered states of consciousness	Book chapter	All
Shear (2011b)	State-enlivening and practice-makes-perfect approaches to meditation	Journal article	All
Shear (2014a)	Meditation as first-person methodology: Real promise—and problems	Book chapter	All
Shear (2014b)	Some reflections on meditation research and consciousness studies	Journal article	All
Travis			
Travis and Pearson (2000)[g]	Pure consciousness: Distinct phenomenological and physiological correlates of "consciousness itself"	Journal article	All
Travis (2001)	Autonomic and EEG patterns distinguish transcending from other experiences during Transcendental Meditation practice	Journal article	All
Arenander and Travis (2004)	Brain patterns of self-awareness	Journal article	All
Travis et al. (2005)	Maharishi Vedic science addresses the "hard" problem of consciousness	Journal article	All
Travis (2006)	From I to I: Concepts of self on an object-referral/self-referral continuum	Book chapter	All
Travis (2009)	Brain functioning as the ground for spiritual experiences and ethical behavior	Journal article	All
Travis (2010)	The Center for Brain, Consciousness, and Cognition at Maharishi University of Management	Journal article	All
Travis and Shear (2010a)[f]	Focused attention, open monitoring and automatic self-transcending: Categories to organize meditations from Vedic, Buddhist and Chinese traditions	Journal article	All
Travis and Shear (2010b)[f]	Reply to Josipovic: Duality and non-duality in meditation research	Journal article	All
Travis (2011)	States of consciousness beyond waking, dreaming and sleeping: Perspectives from research on meditation experiences	Book chapter	All
Heaton et al. (2012)	A consciousness-based approach to management education for integrity	Book chapter	All
Travis (2014)	Transcendental experiences during meditation practice	Journal article	All
Heaton and Travis (2014)	Consciousness, empathy, and the brain	Book chapter	All
Travis (2016)	Transcending as a driver of development	Journal article	All
Faber et al. (2017)	EEG microstates during different phases of Transcendental Meditation practice	Journal article	All
Travis and Parim (2017)	Default mode network activation and Transcendental Meditation practice: Focused attention or automatic self-transcending?	Journal article	All

Travis (2018)	Long-term changes in experienced yoga practitioners: Growth of higher states of consciousness	Book chapter	All
Gobec and Travis (2018)	Effects of Maharishi Yoga Asanas on mood states, happiness, and experiences during meditation	Journal article	All

Note. [a] Publications of each of the five main authors (Pearson, Rosenthal, Roth, Shear, Travis), as author or co-author, are ordered by year of publication (earliest to latest) of the version reviewed. [b] Originally published as Shear (1996c). [c] Originally published as Shear (1998). [d] Also published as Shear and Jevning (1999b). [e] Also published as Varela and Shear (1999a). [f] Travis and Shear (2010a, 2010b) are included in both the Shear and Travis sections of the table, as they were identified from the Travis searches and the Shear searches. [g] Also published as Travis and Pearson (2010). For the reasons provided in the section "Selection of Publications", Travis and Pearson (2000) is included in the Travis section but not the Pearson section.

Table F3

Publications Selected for Stillness Meditation

Citation[a]	Title	Publication type	Section/s reviewed
McKinnon			
McKinnon (1991)	Help yourself and your child to happiness	Book	All
McKinnon (2002/2008)	Let's be still: Teaching Stillness Meditation to children and adolescents	Book – Manual	All
McKinnon (2011)	Living calm in a busy world: Stillness Meditation in the Meares tradition	Book	All
McKinnon (1983/2016)	In stillness conquer fear: How to break free from anxiety and phobias	Book	All
Meares			
Meares (1967a)	Pain and the psychiatrist	Journal article	All
Meares (1967b)	Psychological control of organically determined pain	Journal article	All
Meares (1967c)	Suggestion, hypnosis and intuition	Journal article	All
Meares(1967d)	Teaching the patient control of organically determined pain	Journal article	All
Meares (1967e)	The space between	Journal article	All
Meares (1967/1968)	Relief without drugs: The self-management of tension, anxiety, and pain	Book	All
Meares (1968a)	Hypnotherapy without the phenomena of hypnosis	Journal article	All
Meares (1968b)	Psychological mechanisms in the relief of pain by hypnosis	Journal article	All
Meares (1968c)	Umm	Journal article	All
Meares (1969a)	Hypnosis and transcendental religious experience	Journal article	All
Meares (1969d)	The place of hypnosis in the treatment of anxiety	Journal article	All
Meares (1969b)	Strange places and simple truths: A psychiatrist looks at eastern mysticism	Book	All
Meares (1969c)	Student problems and a guide to study	Book	All
Meares (1970)	The way up: The practical psychology of success	Book	All
Meares (1971a)[b]	Group relaxing hypnosis	Journal article	All
Meares (1971c)	The nature, use and abuse of hypnosis	Journal article	All
Meares (1973a)	A psychiatric experiment in community service	Journal article	All
Meares (1973b)	Dialogue with youth	Book	All

Meares (1973c)	Nervous tension in professional life	Journal article	All
Meares (1974a)	The drop-out syndrome	Journal article	All
Meares (1974b)	The new woman: Women at the crossroads of social and psychological revolution	Book	All
Meares (1975)	Why be old? How to avoid the psychological reactions of ageing	Book	All
Meares (1976a)	Regression of cancer after intensive meditation	Journal article	All
Meares (1976b)	The relief of anxiety through relaxing meditation	Journal article	All
Meares (1977a)	Atavistic regression as a factor in the remission of cancer	Journal article	All
Meares (1977b)	Cancer: Another way?	Book – verse-style[c]	All
Meares (1977c)	The quality of meditation effective in the regression of cancer	Journal article	All
Meares (1978a)	Regression of osteogenic sarcoma metastases associated with intensive meditation	Journal article	All
Meares (1978b)	The hidden powers of leadership	Book	All
Meares (1978c)	Vivid visualization and dim visual awareness in the regression of cancer in meditation	Journal article	All
Meares (1979b)	Dialogue on meditation	Book – verse-style	All
Meares (1979c)[d]	Meditation and cancer	Journal article	All
Meares (1979d)	Meditation: A psychological approach to cancer treatment	Journal article	All
Meares (1979a)	Atavistic communication by touch in the psychological treatment of cancer by intensive meditation	Journal article	All
Meares (1979e)	Regression of cancer of the rectum after intensive meditation	Journal article	All
Meares (1979f)	The psychological treatment of cancer: The patient's confusion of the time for living with the time for dying	Journal article	All
Meares (1976/1980)	Let's be human: New reactions for old	Book	All
Meares (1980a)	Massage as an adjunct to meditation in the psychological treatment of cancer	Journal article	All
Meares (1980b)	Our attitude of mind in the psychological treatment of cancer	Journal article	All
Meares (1980c)	Remission of massive metastastis from undifferentiated carcinoma of the lung associated with intensive meditation	Journal article	All
Meares (1980e)	Thoughts	Book – verse-style	All
Meares (1980f)	What can the cancer patient expect from intensive meditation?	Journal article	All
Meares (1981a)	Cancer, psychosomatic illness, and hysteria	Journal article	All
Meares (1981b)	Prayer and beyond	Book – verse-style	All
Meares (1981c)	Regression of recurrence of carcinoma of the breast at mastectomy site associated with intensive meditation	Journal article	All
Meares (1982a)	Meditation and the prevention of the recurrence of cancer	Journal article	All
Meares (1982b)	My soul and I	Book – verse-style	All
Meares (1982c)	Stress, meditation and the regression of cancer	Journal article	All
Meares (1983a)	A form of intensive meditation associated with the regression of cancer	Journal article	All
Meares (1983b)	Psychological mechanisms in the regression of cancer	Journal article	All
Meares (1984)	A way of doctoring	Book – verse style	All
Meares (1976/1984)	From the quiet place	Book – verse-style	All
Meares (1978/1986)	The wealth within: Self-help through a system of relaxing meditation	Book	All

Meares (1987a)	Let's be at ease	Book – verse-style	All
Meares (1987b)	Man and woman	Book – verse-style	All
Meares (1988)	The silver years: Practical advice for a rewarding later life	Book	All
Meares (1989)	A better life: The guide to meditation	Book	All
Meares (1984/1989)	A kind of believing	Book – verse-style	All
Meares (1987/1991)	Life without stress: The self-management of stress	Book	All

Note. [a] Publications of each of Meares and McKinnon are ordered by year of publication (earliest to latest) of the version reviewed. [b] Also published as Meares (1971b). [c] Meares used the verse-style structure in certain of his books (as identified in the table) to encourage the meditator to "[read] between the lines", and consequently move beyond logic (Meares, 1983a, p. 120). For this reason he saw them as more like Zen Buddhist writings than poetry. [d] Also published as Meares (1980d).

References

Arenander, A., and Travis, F. (2004). "Brain patterns of self-awareness," in *Deficits of self-awareness in psychiatric disorders*, eds. B. Beitman and J. Nair (New York, NY: Norton), 112–126.

Desbordes, G., Negi, L. T., Pace, T. W., Wallace, B. A., Raison, C. L., and Schwartz, E. L. (2012). Effects of mindful-attention and compassion meditation training on amygdala response to emotional stimuli in an ordinary, non-meditative state. *Frontiers in Human Neuroscience* 6, 1–15. doi:10.3389/fnhum.2012.00292.

Faber, P. L., Travis, F., Milz, P., and Parim, N. (2017). EEG microstates during different phases of Transcendental Meditation practice. *Cognitive Processing* 18, 307–314. doi:10.1007/s10339-017-0812-y.

Gallagher, S., and Shear, J. (1999). "Editors' introduction," in *Models of the self*, eds. S. Gallagher and J. Shear (Devon, United Kingdom: Imprint), ix–xviii.

Gobec, S., and Travis, F. (2018). Effects of Maharishi Yoga Asanas on mood states, happiness, and experiences during meditation. *International Journal of Yoga* 11, 66–71. doi:10.4103/ijoy.IJOY_66_16.

Heaton, D. P., and Travis, F. (2014). "Consciousness, empathy, and the brain," in *Organizing through empathy*, eds. K. Pavlovich and K. Krahnke (New York, NY: Routledge), 17–33.

Heaton, D. P., Travis, F., and Subramaniam, R. (2012). "A consciousness-based approach to management education for integrity," in *Handbook of research on teaching ethics in business and management education*, eds. C. Wankel and A. Stachowicz-Stanusch (Hershey, PA: IGI Global), 65–79.

Kozasa, E. H., Lacerda, S. S., Menezes, C., Wallace, B. A., Radvany, J., Mello, L. E., et al. (2015). Effects of a 9-day shamatha Buddhist meditation retreat on attention, mindfulness and self-compassion in participants with a broad range of meditation experience. *Mindfulness* 6, 1235–1241. doi:10.1007/s12671-015-0385-8.

McKinnon, P. (1991). *Help yourself and your child to happiness*. Melbourne, Australia: David Lovell.

McKinnon, P. (2002/2008). *Let's be still: Teaching Stillness Meditation to children and adolescents*. Melbourne, Australia: David Lovell.

McKinnon, P. (2011). *Living calm in a busy world: Stillness Meditation in the Meares tradition*. Melbourne, Australia: David Lovell.

McKinnon, P. (1983/2016). *In stillness conquer fear: How to break free from anxiety and phobias*. Rev. ed. Melbourne, Australia: Garratt.

Meares, A. (1967a). Pain and the psychiatrist. *Australian and New Zealand Journal of Psychiatry* 1, 116–117. doi:10.3109/00048676709159179.

Meares, A. (1967b). Psychological control of organically determined pain. *Annals of the Australian College of Dental Surgeons* 1, 42–46.

Meares, A. (1967c). Suggestion, hypnosis and intuition. *Proceedings of the Medico-Legal Society of Victoria* 11, 73–84.

Meares, A. (1967d). Teaching the patient control of organically determined pain. *The Medical Journal of Australia* 1, 11–12. doi:10.5694/j.1326-5377.1967.tb20984.x.

Meares, A. (1967e). The space between. *The International Journal of Clinical and Experimental Hypnosis* 15, 156–159.

Meares, A. (1968a). Hypnotherapy without the phenomena of hypnosis. *International Journal of Clinical and Experimental Hypnosis* 16, 211–214. doi:10.1080/00207146808410594.

Meares, A. (1968b). Psychological mechanisms in the relief of pain by hypnosis. *American Journal of Clinical Hypnosis* 11, 55–57. doi:10.1080/00029157.1968.10402002.

Meares, A. (1967/1968). *Relief without drugs: The self-management of tension, anxiety, and pain*. London, United Kingdom: Souvenir Press.

Meares, A. (1968c). Umm. *The Medical Journal of Australia* 2, 638. doi:10.5694/j.1326-5377.1968.tb83053.x.

Meares, A. (1969a). Hypnosis and transcendental religious experience. *Journal of Existential Psychiatry* 7, 119–121.

Meares, A. (1969b). *Strange places and simple truths: A psychiatrist looks at eastern mysticism*. United Kingdom, London: Souvenir Press.

Meares, A. (1969c). *Student problems and a guide to study*. Melbourne, Australia: Hawthorn Press.

Meares, A. (1969d). The place of hypnosis in the treatment of anxiety. *Australian and New Zealand Journal of Psychiatry* 3, 299–300.

Meares, A. (1970). *The way up: The practical psychology of success*. London, United Kingdom: Souvenir Press.

Meares, A. (1971a). Group relaxing hypnosis. *The Medical Journal of Australia* 2, 675–676.

Meares, A. (1971b). Group relaxing hypnosis. *The Journal of the American Society of Psychosomatic Dentistry and Medicine* 19, 137–141.

Meares, A. (1971c). The nature, use and abuse of hypnosis. *Australian Journal of Forensic Sciences* 3, 157–161. doi:10.1080/00450617109413182.

Meares, A. (1973a). A psychiatric experiment in community service. *The Medical Journal of Australia* 15, 733–734. doi:10.5694/j.1326-5377.1973.tb110661.x.

Meares, A. (1973b). *Dialogue with youth*. London, United Kingdom: Collins.

Meares, A. (1973c). Nervous tension in professional life. *Proceedings of the Medico-Legal Society of Victoria* 12, 277–284.

Meares, A. (1974a). The drop-out syndrome. *Australian Family Physician* 3, 164–168.

Meares, A. (1974b). *The new woman: Women at the crossroads of social and psychological revolution*. London, United Kingdom: Collins.

Meares, A. (1975). *Why be old? How to avoid the psychological reactions of ageing*. London, United Kingdom: Collins.

Meares, A. (1976a). Regression of cancer after intensive meditation. *The Medical Journal of Australia* 2, 184. doi:10.5694/j.1326-5377.1976.tb134456.x.

Meares, A. (1976b). The relief of anxiety through relaxing meditation. *Australian Family Physician* 5, 906–910.

Meares, A. (1977a). Atavistic regression as a factor in the remission of cancer. *The Medical Journal of Australia* 2, 132–133. doi:10.5694/j.1326-5377.1977.tb99090.x.

Meares, A. (1977b). *Cancer: Another way?*. Melbourne, Australia: Hill of Content.

Meares, A. (1977c). The quality of meditation effective in the regression of cancer. *The Journal of the American Society of Psychosomatic Dentistry and Medicine* 25, 129–132.

Meares, A. (1978a). Regression of osteogenic sarcoma metastases associated with intensive meditation. *The Medical Journal of Australia* 2, 433. doi:10.5694/j.1326-5377.1978.tb76834.x.

Meares, A. (1978b). *The hidden powers of leadership*. Melbourne, Australia: Hill of Content.

Meares, A. (1978c). Vivid visualization and dim visual awareness in the regression of cancer in meditation. *The Journal of the American Society of Psychosomatic Dentistry and Medicine* 25, 85–88.

Meares, A. (1979a). Atavistic communication by touch in the psychological treatment of cancer by intensive meditation. *Journal of Holistic Health* 4, 120–124.

Meares, A. (1979b). *Dialogue on meditation*. Melbourne, Australia: Hill of Content.

Meares, A. (1979c). Meditation and cancer. *Yoga Quarterly* 14, 27–31.

Meares, A. (1979d). Meditation: A psychological approach to cancer treatment. *The Practitioner* 222, 119–122.

Meares, A. (1979e). Regression of cancer of the rectum after intensive meditation. *The Medical Journal of Australia* 2, 539–540. doi:10.5694/j.1326-5377.1979.tb127157.x.

Meares, A. (1979f). The psychological treatment of cancer: The patient's confusion of the time for living with the time for dying. *Australian Family Physician* 8, 801–805.

Meares, A. (1976/1980). *Let's be human: New reactions for old*. Melbourne, Australia: Hill of Content.

Meares, A. (1980a). Massage as an adjunct to meditation in the psychological treatment of cancer. *Australian Journal of Physiotherapy* 26, 25–26. doi:10.1016/s0004-9514(14)60799-6.

Meares, A. (1980b). Our attitude of mind in the psychological treatment of cancer. *Australian Nurses Journal* 9, 29–30.

Meares, A. (1980c). Remission of massive metastastis from undifferentiated carcinoma of the lung associated with intensive meditation. *The Journal of the American Society of Psychosomatic Dentistry and Medicine* 27, 40–41.

Meares, A. (1980d). The onflow of meditation in the psychological treatment of cancer. *Journal of the Helen Vale Foundation* 2, 10–14.

Meares, A. (1980e). *Thoughts*. Melbourne, Australia: Hill of Content.

Meares, A. (1980f). What can the cancer patient expect from intensive meditation? *Australian Family Physician* 9, 322–325.

Meares, A. (1981a). Cancer, psychosomatic illness, and hysteria. *The Lancet* 318, 1037–1038. doi:10.1016/s0140-6736(81)91228-9.

Meares, A. (1981b). *Prayer and beyond*. Melbourne, Australia: Hill of Content.

Meares, A. (1981c). Regression of recurrence of carcinoma of the breast at mastectomy site associated with intensive meditation. *Australian Family Physician* 10, 218–219.

Meares, A. (1982a). Meditation and the prevention of the recurrence of cancer. *Journal of the Helen Vale Foundation* 3, 13–16.

Meares, A. (1982b). *My soul and I*. Melbourne, Australia: Author.

Meares, A. (1982c). Stress, meditation and the regression of cancer. *The Practitioner* 226, 1607–1609.

Meares, A. (1983a). A form of intensive meditation associated with the regression of cancer. *American Journal of Clinical Hypnosis* 25, 114–121. doi:10.1080/00029157.1982.10404082.

Meares, A. (1983b). Psychological mechanisms in the regression of cancer. *The Medical Journal of Australia* 1, 583–584. doi:10.5694/j.1326-5377.1983.tb136229.x.

Meares, A. (1984). *A way of doctoring*. Melbourne, Australia: Hill of Content.

Meares, A. (1976/1984). *From the quiet place*. Melbourne, Australia: Hill of Content.

Meares, A. (1978/1986). *The wealth within: Self-help through a system of relaxing meditation*. Melbourne, Australia: Hill of Content.

Meares, A. (1987a). *Let's be at ease*. Melbourne, Australia: Greenhouse.

Meares, A. (1987b). *Man and woman*. Melbourne, Australia: Greenhouse.

Meares, A. (1988). *The silver years: Practical advice for a rewarding later life*. Melbourne, Australia: Greenhouse.

Meares, A. (1989). *A better life: The guide to meditation*. Melbourne, Australia: Greenhouse.

Meares, A. (1984/1989). "A kind of believing," in *Dialogue on meditation; From the quiet place; A kind of believing* (Melbourne, Australia: Hill of Content).

Meares, A. (1987/1991). *Life without stress: The self-management of stress*. Melbourne, Australia: Penguin.

Pearson, C. (2013). *The supreme awakening: Experiences of enlightenment throughout time – and how you can cultivate them*. Fairfield, IA: Maharishi University of Management Press.

Rosenthal, N. E. (2011/2012). *Transcendence: Healing and transformation through Transcendental Meditation*. New York, NY: Penguin.

Rosenthal, N. E. (2016/2017). *Super Mind: How to boost performance and live a richer and happier life through Transcendental Meditation*. New York, NY: Penguin.

Roth, B. (2018). *Strength in stillness: The power of Transcendental Meditation*. London, United Kingdom: Simon & Schuster.

Shear, J. (1990a). Mystical experience, hermeneutics, and rationality. *International Philosophical Quarterly* 30, 391–401. doi:10.5840/ipq199030436.

Shear, J. (1990b). *The inner dimension: Philosophy and the experience of consciousness*. 2nd ed. London, United Kingdom: Harmonia.

Shear, J. (1994). On mystical experiences as support for the perennial philosophy. *Journal of the American Academy of Religion* 62, 319–342. doi:10.1093/jaarel/lxii.2.319.

Shear, J. (1995). Mystical knowledge? *Sufi* 27, 12–16.

Shear, J. (1996a). Back and forth with Huston Smith. *Sufi* 31, 8–9.

Shear, J. (1996b). "On the existence of a culture independent core component of self," in *East-west encounters in philosophy and religion*, eds. N. Smart and B. S. Murthy (Long Beach, CA: Long Beach), 359–376.

Shear, J. (1996c). The hard problem: Closing the empirical gap. *Journal of Consciousness Studies* 3, 54–68.

Shear, J. (1995/1997a). "Introduction," in *Explaining consciousness – The "hard" problem*, ed. J. Shear (Cambridge, MA: MIT Press), 1–6.

Shear, J. (1995/1997b). “The hard problem: Closing the empirical gap,” in *Explaining consciousness – The “hard” problem*, ed. J. Shear (Cambridge, MA: MIT Press), 359–375.

Shear, J. (1998). Experiential clarification of the problem of self. *Journal of Consciousness Studies* 5, 673–686.

Shear, J. (1998/1999). “Experiential clarification of the problem of self,” in *Models of the self*, eds. S. Gallagher and J. Shear (Devon, United Kingdom: Imprint), 407–420.

Shear, J. (2002). “Ethics and the experience of happiness,” in *Crossing boundaries: Essays on the ethical status of mysticism*, eds. G. W. Barnard and J. J. Kripal (New York, NY: Seven Bridges Press), 361–380.

Shear, J. (2001/2002). “Experimental studies of meditation and consciousness,” in *Religion and psychology: Mapping the terrain*, eds. D. Jonte-Pace and W. B. Parsons (London, United Kingdom: Routledge), 280–294.

Shear, J. (2004). Mysticism and scientific naturalism. *Sophia* 43, 83–99. doi:10.1007/bf02782439.

Shear, J. (2006a). “Conclusion,” in *The experience of meditation: Experts introduce the major traditions*, ed. J. Shear (St. Paul, MN: Paragon House), 259–268.

Shear, J. (2006b). “Introduction,” in *The experience of meditation: Experts introduce the major traditions*, ed. J. Shear (St. Paul, MN: Paragon House), xiii–xxii.

Shear, J. (2006c). “Transcendental Meditation,” in *The experience of meditation: Experts introduce the major traditions*, ed. J. Shear (St. Paul, MN: Paragon House), 23–48.

Shear, J. (2007). “Eastern methods of investigating mind and consciousness,” in *The Blackwell companion to consciousness*, eds. M. Velmans and S. Schneider (Malden, MA: Blackwell), 697–710.

Shear, J. (2011a). “Eastern approaches to altered states of consciousness,” in *Altering consciousness: Multidisciplinary perspectives*, eds. E. Cardeña and M. Winkelman (Santa Barbara, CA: Praeger), 139–158.

Shear, J. (2011b). State-enlivening and practice-makes-perfect approaches to meditation. *Biofeedback* 39, 51–55. doi:10.5298/1081-5937-39.2.07.

Shear, J. (2014a). “Meditation as first-person methodology: Real promise–and problems,” in *Meditation – Neuroscientific approaches and philosophical implications*, eds. S. Schmidt and H. Walach (Cham, Switzerland: Springer International), 57–74.

Shear, J. (2014b). Some reflections on meditation research and consciousness studies. *Journal of Consciousness Studies* 21, 202–215.

Shear, J., and Jevning, R. (1999a). Pure consciousness: Scientific exploration of meditation techniques. *Journal of Consciousness Studies* 6, 189–210.

Shear, J., and Jevning, R. (1999b). “Pure consciousness: Scientific exploration of meditation techniques,” in *The view from within: First-person approaches to the study of consciousness*, eds. F. Varela and J. Shear (Devon, United Kingdom: Imprint Academic), 189–210.

Travis, F. (2001). Autonomic and EEG patterns distinguish transcending from other experiences during Transcendental Meditation practice. *International Journal of Psychophysiology* 42, 1–9. doi:10.1016/s0167-8760(01)00143-x.

Travis, F. (2006). “From I to I: Concepts of self on an object-referral/self-referral continuum,” in *The concept of self in psychology*, ed. A. P. Prescott (New York, NY: Nova Science), 21–43.

Travis, F. (2009). Brain functioning as the ground for spiritual experiences and ethical behavior. *FBI Law Enforcement Bulletin* 78, 26–32. doi:10.1037/e511562010-007.

Travis, F. (2010). The Center for Brain, Consciousness, and Cognition at Maharishi University of Management. *Cognitive Processing* 11, 85–86. doi:10.1007/s10339-009-0349-9.

Travis, F. (2011). “States of consciousness beyond waking, dreaming and sleeping: Perspectives from research on meditation experiences,” in *States of consciousness: Experimental insights into meditation, waking, sleep and dreams*, eds. D. Cvetkovic and I. Cosic (Berlin, Germany: Springer), 223–234.

Travis, F. (2014). Transcendental experiences during meditation practice. *Annals of the New York Academy of Sciences* 1307, 1–8. doi:10.1111/nyas.12316.

Travis, F. (2016). Transcending as a driver of development. *Annals of the New York Academy of Sciences* 1373, 72–77. doi:10.1111/nyas.13071.

Travis, F. (2018). “Long-term changes in experienced yoga practitioners: Growth of higher states of consciousness,” in *Research based perspectives on the psychophysiology of yoga*, eds. S. Telles and N. Singh (Hershey, PA: IGI Global), 35–51.

Travis, F., Munly, K., Olson, T. M., and Sorflaten, J. W. (2005). Maharishi Vedic science addresses the “hard” problem of consciousness. *Journal of Social Behavior and Personality* 17, 123–135.

Travis, F., and Parim, N. (2017). Default mode network activation and Transcendental Meditation practice: Focused attention or automatic self-transcending? *Brain and Cognition* 111, 86–94. doi:10.1016/j.bandc.2016.08.009.

Travis, F., and Pearson, C. (2000). Pure consciousness: Distinct phenomenological and physiological correlates of “consciousness itself”. *International Journal of Neuroscience* 100, 77–89. doi:10.3109/00207450008999678.

Travis, F., and Pearson, C. (2010). “Pure Consciousness: Distinct phenomenological and physiological correlates of ‘consciousness itself’,” in *Consciousness-based education: A foundation for teaching and learning in the academic disciplines*, eds. F. Travis, D. Llewellyn, and C. Pearson (Fairfield, IA: Maharishi University of Management), 343–359.

Travis, F., and Shear, J. (2010a). Focused attention, open monitoring and automatic self-transcending: Categories to organize meditations from Vedic, Buddhist and Chinese traditions. *Consciousness and Cognition* 19, 1110–1118. doi:10.1016/j.concog.2010.01.007.

Travis, F., and Shear, J. (2010b). Reply to Josipovic: Duality and non-duality in meditation research. *Consciousness and Cognition* 19, 1122–1123. doi:10.1016/j.concog.2010.04.003.

Varela, F. J., and Shear, J. (1999a). "First-person accounts: Why, what and how," in *The view from within: First-person approaches to the study of consciousness*, eds. F. Varela and J. Shear (Devon, United Kingdom: Imprint Academic), 1–14.

Varela, F. J., and Shear, J. (1999b). First-person methodologies: What, why, how? *Journal of Consciousness Studies* 6, 1–14.

Wallace, B. A. (1999a). The Buddhist tradition of Samatha: Methods for refining and examining consciousness. *Journal of Consciousness Studies* 6, 175–187.

Wallace, B. A. (1999b). "Training the attention and exploring consciousness in Tibetan Buddhism," in *Toward a science of consciousness III: The third Tucson discussions and debates*, eds. S. R. Hameroff, A. W. Kaszniak, and D. J. Chalmers (Cambridge, MA: MIT Press), 441–448.

Wallace, B. A. (2000). *The taboo of subjectivity. Toward a new science of consciousness*. Oxford, United Kingdom: Oxford University Press.

Wallace, B. A. (2001a). Intersubjectivity in Indo-Tibetan Buddhism. *Journal of Consciousness Studies* 8, 209–230.

Wallace, B. A. (2001b). The potential of emptiness: Vacuum states in physics and consciousness. *The Scientific and Medical Network Review* 77, 21–25.

Wallace, B. A. (2001/2003). *Buddhism with an attitude: The Tibetan seven-point mind-training*. Ithaca, NY: Snow Lion.

Wallace, B. A. (1989/2003). *Choosing reality: A Buddhist view of physics and the mind*. Ithaca, NY: Snow Lion.

Wallace, B. A. (2004). Scientific and Buddhist views of energy. *Subtle Energies & Energy Medicine* 15, 17–25.

Wallace, B. A. (1998/2005). *Balancing the mind: A Tibetan Buddhist approach to refining attention*. Boston, MA: Shambhala.

Wallace, B. A. (2005). *Genuine happiness: Meditation as the path to fulfillment*. Hoboken, NJ: John Wiley.

Wallace, B. A. (2006a). *The attention revolution: Unlocking the power of the focused mind*. Somerville, MA: Wisdom.

Wallace, B. A. (2006b). "Vacuum states of consciousness: A Tibetan Buddhist view," in *Buddhist thought and applied psychological research: Transcending the boundaries*, eds. D. K. Nauriyal, M. S. Drummond, and Y. B. Lal (Oxon, United Kingdom: Routledge), 112–121.

Wallace, B. A. (2007a). *Contemplative science: Where Buddhism and neuroscience converge*. New York, NY: Columbia University Press.

Wallace, B. A. (2007b). *Hidden dimensions: The unification of physics and consciousness*. New York, NY: Columbia University Press.

Wallace, B. A. (2010). *The four immeasurables: Practices to open the heart*. 3rd ed. Ithaca, NY: Snow Lion.

Wallace, B. A. (2011a). *Minding closely: The four applications of mindfulness*. Ithaca, NY: Snow Lion.

Wallace, B. A. (2011b). *Stilling the mind: Shamatha teachings from Düdjom Lingpa's Vajra Essence*. Somerville, MA: Wisdom.

Wallace, B. A. (2012). *Dreaming yourself awake: Lucid dreaming and Tibetan dream yoga for insight and transformation*. Boston, MA: Shambhala.

Wallace, B. A. (2012/2014). *Meditations of a Buddhist skeptic: A manifesto for the mind sciences and contemplative practice*. New York, NY: Columbia University Press.

Wallace, B. A. (2009/2014). *Mind in the balance: Meditation in science, Buddhism, and Christianity*. New York, NY: Columbia University Press.

Wallace, B. A. (2018). *Fathoming the mind: Inquiry and insight in Düdjom Lingpa's Vajra Essence*. Somerville, MA: Wisdom.

Wallace, B. A., and Hodel, B. (2008). *Embracing mind: The common ground of science & spirituality*. Boston, MA: Shambhala.

Wallace, B. A., and Shapiro, S. L. (2006). Mental balance and well-being: Building bridges between Buddhism and Western psychology. *American Psychologist* 61, 690–701. doi:10.1037/0003-066x.61.7.690.

Wallace, B. A., and Wilhelm, S. (1993). *Tibetan Buddhism from the ground up: A practical approach for modern life*. Boston, MA: Wisdom.

Zanesco, A. P., King, B. G., MacLean, K. A., Jacobs, T. L., Aichele, S. R., Wallace, B. A., et al. (2016). Meditation training influences mind wandering and mindless reading. *Psychology of Consciousness* 3, 12. doi:10.1037/cns0000082.
