# Supplementary material for: Silence in Shamatha, Transcendental, and Stillness Meditation: An Evidence Synthesis Based on Expert Texts
Source: Front Psychol. 2020 Jul 8;11:1259. doi: 10.3389/fpsyg.2020.01259 (PMC7360996; doi:10.3389/fpsyg.2020.01259)
Supplement: Supplementary file 7 [file Data_Sheet_7.pdf]

Appendix G: Order Publications Reviewed in Extraction Process

The Shamatha publications reviewed for the Shamatha extraction table (Table S1) are set out in Table F1 in Appendix F. They were reviewed in the following order: (a) Wallace (2006a, 2011b), the two most recent of Wallace's books devoted principally to shamatha practice; (b) Wallace's other books besides Wallace (2018); (c) Wallace (1999a, 2001a), Wallace's two journal articles that are particularly information-rich with regard to the practice and its context; (d) Wallace's (2018) book; and (e) Wallace's book chapters and remaining journal articles. For category (a) the 2006a text was reviewed first, and for category (c) the 2001a text was reviewed first. Within each of categories (b) and (e), the publications were reviewed in the reverse of the order in which they are listed in Table F1.

The TM publications reviewed for the TM extraction table (Table S2) are set out in Table F2 in Appendix F. They were reviewed in the following order: (a) Pearson's (2013) book; (b) Roth's (2018) book; (c) Shear's (1990b) book; (d) Shear's journal articles and book chapters, other than Travis and Shear (2010a, 2010b); (e) Rosenthal's (2011/2012, 2016/2017) books; and (f) Travis' journal articles and book chapters, including Travis and Shear (2010a, 2010b). Within each of categories (d) and (f), the publications were reviewed in the order they are listed in Table F2. For category (e), the 2017 text was reviewed first.

The publications reviewed for the Stillness Meditation extraction table (Table S3) are set out in Table F3 in Appendix F. They were reviewed in the following order: (a) Meares (1967/1968, 1978/1986), Meares' two main books on Stillness Meditation; (b) Meares (1969c, 1973b, 1989, 1987/1991), another four of Meares' books with major presentations of the practice; (c) Meares' remaining books other than those in verse-style (as identified in Table F3); (d) Meares' journal articles; (e) Meares' verse-style books; and (f) McKinnon's books. Within each of categories (a) and (b), the publications were reviewed in the order listed for those categories above. Within each of categories (c), (d) and (e), the publications were reviewed in the order listed in Table F3. For category (f), the publications were reviewed in the following order: McKinnon (1983/2016, 2011, 1991, 2002/2008).

For the sections of each extraction table in which each row refers to a different publication, ordering of those rows/publications reflects the order in which the publications were reviewed, as described above.

References

Pearson, C. (2013). *The supreme awakening: Experiences of enlightenment throughout time – and how you can cultivate them*. Fairfield, IA: Maharishi University of Management Press.

Rosenthal, N. E. (2011/2012). *Transcendence: Healing and transformation through Transcendental Meditation*. New York, NY: Penguin.

Rosenthal, N. E. (2016/2017). *Super Mind: How to boost performance and live a richer and happier life through Transcendental Meditation*. New York, NY: Penguin.

Roth, B. (2018). *Strength in stillness: The power of Transcendental Meditation*. London, United Kingdom: Simon & Schuster.

McKinnon, P. (1991). *Help yourself and your child to happiness*. Melbourne, Australia: David Lovell.

McKinnon, P. (2002/2008). *Let's be still: Teaching Stillness Meditation to children and adolescents*. Melbourne, Australia: David Lovell.

McKinnon, P. (2011). *Living calm in a busy world: Stillness Meditation in the Meares tradition*. Melbourne, Australia: David Lovell.

McKinnon, P. (1983/2016). *In stillness conquer fear: How to break free from anxiety and phobias*. Rev. ed. Melbourne, Australia: Garratt.

Meares, A. (1967/1968). *Relief without drugs: The self-management of tension, anxiety, and pain*. London, United Kingdom: Souvenir Press.

Meares, A. (1969c). *Student problems and a guide to study*. Melbourne, Australia: Hawthorn Press.

Meares, A. (1973b). *Dialogue with youth*. London, United Kingdom: Collins.

Meares, A. (1978/1986). *The wealth within: Self-help through a system of relaxing meditation*. Melbourne, Australia: Hill of Content.

Meares, A. (1989). *A better life: The guide to meditation*. Melbourne, Australia: Greenhouse.

Meares, A. (1987/1991). *Life without stress: The self-management of stress*. Melbourne, Australia: Penguin.

Shear, J. (1990b). *The inner dimension: Philosophy and the experience of consciousness*. 2nd ed. London, United Kingdom: Harmonia.

Travis, F., and Shear, J. (2010a). Focused attention, open monitoring and automatic self-transcending: Categories to organize meditations from Vedic, Buddhist and Chinese traditions. *Consciousness and Cognition* 19, 1110–1118. doi:10.1016/j.concog.2010.01.007.

Travis, F., and Shear, J. (2010b). Reply to Josipovic: Duality and non-duality in meditation research. *Consciousness and Cognition* 19, 1122–1123. doi:10.1016/j.concog.2010.04.003.

Wallace, B. A. (1999a). The Buddhist tradition of Samatha: Methods for refining and examining consciousness. *Journal of Consciousness Studies* 6, 175–187.

Wallace, B. A. (2001a). Intersubjectivity in Indo-Tibetan Buddhism. *Journal of Consciousness Studies* 8, 209–230.

Wallace, B. A. (2006a). *The attention revolution: Unlocking the power of the focused mind*. Somerville, MA: Wisdom.

Wallace, B. A. (2011b). *Stilling the mind: Shamatha teachings from Düdjom Lingpa's Vajra Essence*. Somerville, MA: Wisdom.

Wallace, B. A. (2018). *Fathoming the mind: Inquiry and insight in Düdjom Lingpa's Vajra Essence*. Somerville, MA: Wisdom.
