# Supplementary material for: Silence in Shamatha, Transcendental, and Stillness Meditation: An Evidence Synthesis Based on Expert Texts
Source: Front Psychol. 2020 Jul 8;11:1259. doi: 10.3389/fpsyg.2020.01259 (PMC7360996; doi:10.3389/fpsyg.2020.01259)
Supplement: Supplementary file 8 [file Data_Sheet_8.pdf]

Database Contents List

List of Contents for Table S1 – Shamatha Extraction Table

Part/section no.	Part/section name	Page
Part 1	Technique and interim-states	1
1.1	Key root/source texts	1
1.2	Introduction to shamatha practice and the ten stages of attentional development	1
1.3	Tsongkhapa's practice	2
1.4	Mindfulness and introspection	3
1.4.1	Overarching guidance	3
1.4.2	Mindfulness	3
1.4.3	Introspection	4
1.5	Relaxation, stability, vividness, equanimity	5
1.5.1	Overarching guidance	5
1.5.2	Relaxation	6
1.5.3	Stability	7
1.5.4	Vividness	7
1.5.5	Equanimity	8
1.6	Bare awareness/attention	8
1.7	Excitation and laxity	9
1.7.1	Overarching guidance	9
1.7.2	Excitation	9
1.7.3	Laxity	10
1.7.4	Remedies	10
1.8	Will	11
1.9	Mind-wandering	12
1.10	Non-doing, effortlessness, relinquishment of control, loss of ego	12
1.11	The ten stages	17
1.11.1	Stage 1: Directed attention	17
1.11.2	Stage 2: Continuous attention	17
1.11.3	Stage 3: Resurgent attention	17
1.11.4	Stage 4: Close attention	18
1.11.5	Stage 5: Tamed attention	18
1.11.6	Stage 6: Pacified attention	19
1.11.7	Stage 7: Fully pacified attention	19
1.11.8	Stage 8: Single-pointed attention	19
1.11.9	Stage 9: Attentional balance	20
1.11.10	Stage 10: Shamatha	20
1.12	Practice 1 – Mindfulness of breathing	21
1.12.1	Overarching guidance/comments	21
1.12.2	Basic instructions, awareness of breathing	21
1.12.3	Allowing the breath to flow naturally	22
1.12.4	Dealing with thoughts, distractions and discomfort	22
1.12.5	Breathing becoming calmer/subtler	23
1.12.6	Counting breaths	23
1.12.7	Signs of progress	23
1.13	Practice 2 – Settling the mind in its natural state	24
1.13.1	Overarching guidance/comments	24
1.13.2	Basic instructions	24
1.13.3	Signs of progress	28
1.13.4	Stages of progress	29
1.13.5	Stillness and movement	30
1.14	Practice 3 – Awareness of awareness	30
1.14.1	Overarching guidance/comments	30
1.14.2	Basic instructions	31
1.14.3	Pairing with breath	32
1.14.4	Awareness as an object	32
1.14.5	Signs of progress	33
1.14.6	Awareness of the preceding moment	34

1.15	Possible pitfalls with more subtle shamatha techniques	34
1.16	Other references to flawed meditation	34
1.17	Non-duality	35
1.18	Non-conceptuality	35
1.19	Glimpses of substrate consciousness	36
1.20	Other experiences	36
1.21	Posture, dealing with discomfort	37
1.22	Duration of meditation sessions	38
1.23	Time/effort taken to progress, and factors supporting progress	39
1.23.1	General comments	39
1.23.2	Comments on Tibetan Buddhist practitioners specifically	43
1.24	Participant-based academic studies of the subjective experience	44
Part 2	Goal-state	44
2.1	Terminology	44
2.1.1	Terms used to describe features of the goal-state	44
2.1.2	The term shamatha	45
2.1.3	The term quiescence	45
2.1.4	The term nature of the mind	45
2.2	Access to the first meditative stabilization	45
2.3	Substrate consciousness	46
2.3.1	General comments	46
2.3.2	Sleep, death, coma, fainting, hypnosis	47
2.4	Bhavanga	48
2.5	Substrate	48
2.6	Distinction between substrate consciousness and pristine awareness	49
2.7	Potential for rare individuals to achieve pristine awareness through shamatha practice	50
2.8	Potential for glimpses of pristine awareness	51
2.9	Accessing pristine awareness by releasing grasping	52
2.10	Dormant/implicit conceptualization in substrate consciousness	52
2.11	Use of substrate consciousness for particular purposes	53
2.12	Potential to analyze	53
2.13	Speed at which achieved	54
2.14	Features	54
2.14.1	Recognize in retrospect	54
2.14.2	Conscious	54
2.14.3	Consciousness/awareness itself	55
2.14.4	Awake, wakefulness	56
2.14.5	Non-duality	56
2.14.6	Bliss, joy, pleasantness, wellbeing, absence of discomfort	57
2.14.7	Luminosity, clearness, limpidity, transparency, radiance, purity	58
2.14.8	Cognizance, knowledge	61
2.14.9	Stillness	63
2.14.10	Silence, quietness	63
2.14.11	Relaxation	63
2.14.12	Mental rest	64
2.14.13	Empty, contentless	64
2.14.14	No thoughts	66
2.14.15	No feelings	67
2.14.16	No sense-perceptions or body-perceptions	68
2.14.17	No mental images	69
2.14.18	No memories	69
2.14.19	Possible awareness of rhythm of breathing	70
2.14.20	No grasping to the absent mental content	70
2.14.21	Fullness/potential	70
2.14.22	Calm, ease, peacefulness, absence of disturbance	70
2.14.23	Possible loving-kindness	71
2.14.24	No longer mindful of meditation object	71
2.14.25	Attention on substrate/awareness/consciousness/quiescence/mind	72
2.14.26	Attentional stability	74
2.14.27	Attentional vividness	75

2.14.28	Attentional balance	76
2.14.29	Effortlessness, relinquishment of control, non-doing	77
2.14.30	Natural state of the mind	77
2.14.31	Ground state	78
2.14.32	Loss of ego	78
2.14.33	Simplicity	79
2.14.34	Wholeness, coherence, unity	79
2.14.35	Inner security, safety	79
2.14.36	Inner freedom	80
2.14.37	Timelessness	80
2.14.38	Spaciousness	80
2.14.39	Possible spiritual aspect	80
2.14.40	Possible energy	81
2.14.41	Profound, deep	81
2.14.42	Non-changing	82
2.14.43	Subjective experience possibly varies between meditators	82
2.14.44	Difficulty communicating the experience to those who have not had it	82
	References	83

List of Contents for Table S2 – Transcendental Meditation Extraction Table

Part/section no.	Part/section name	Page
Part 1	Technique and Interim-states	1
1.1	Simple	1
1.2	Easy	1
1.3	Pleasant	2
1.4	Key features of the practice	2
1.4.1	General	2
1.4.2	Dealing with distractions/discomforts	4
1.4.3	Other obstacles	4
1.5	Other aspects of the practice/instruction	4
1.5.1	Learning the practice – general	4
1.5.2	The technique is quick to learn/perfect	4
1.5.3	Selection of mantra	5
1.5.4	Mantras are private/secret	5
1.5.5	Oral rather than written instruction	5
1.5.6	Retreats	5
1.6	Effortlessness, control and relinquishment, no concentration	5
1.6.1	General	5
1.6.2	The natural tendency	8
1.7	Posture	9
1.8	Progression to the goal-state/s	9
1.8.1	General	9
1.8.2	Pure individuality and pure bliss/joy/happiness	11
1.9	Practice duration	13
Part 2	Goal-state/s	13
2.1	Terminology – “pure consciousness”, “transcending”, and related terms	13
2.2	Shear’s logic-based argument that pure consciousness has a unique experiential quality	13
2.3	A fourth major state of consciousness, and other higher states	14
2.4	Statements that may be metaphysical and that do not clearly describe an additional feature of the goal-state/s	14
2.5	Participant-based academic studies of the subjective experience	15
2.5.1	The Consciousness Integration Questionnaire	15
2.5.2	Travis and Pearson (2000)	15
2.5.3	Other	16
2.6	Speed at which achieved	16
2.7	Features	16
2.7.1	Recognize in retrospect	16
2.7.2	Cannot say much about it	17
2.7.3	Conscious	18
2.7.4	Pure consciousness/awareness	18

2.7.5	Awake, wakefulness	19
2.7.6	Non-duality	21
2.7.7	Stillness	22
2.7.8	Silence, quietness	22
2.7.9	Dynamic	23
2.7.10	Clearness	23
2.7.11	Purity	23
2.7.12	Relaxation	24
2.7.13	Mental rest	24
2.7.14	Contentless, reduced mental activity	24
2.7.15	Limited or no abstract content	27
2.7.16	No thoughts	28
2.7.17	No feelings	29
2.7.18	No sense-perceptions	30
2.7.19	No body-perceptions	31
2.7.20	No mental images	32
2.7.21	No memories	32
2.7.22	Cycling	32
2.7.23	Increasing length	33
2.7.24	Calm, ease, peacefulness, absence of disturbance	33
2.7.25	Beyond peacefulness	34
2.7.26	Bliss, joy, happiness	34
2.7.27	No sense of anxiety	36
2.7.28	No longer aware of mantra	36
2.7.29	Effortlessness, relinquishment of control, non-doing, no focus/concentration	37
2.7.30	Attention directed inward	37
2.7.31	Transcendence of discomfort	38
2.7.32	Naturalness	38
2.7.33	Loss of ego	39
2.7.34	Simplicity	39
2.7.35	Pure being/Being	40
2.7.36	Nature of mind/being, ground state, self/Self	40
2.7.37	Wholeness, integration, unity	42
2.7.38	Inner security, safety, strength, contentment	43
2.7.39	Inner freedom, unboundedness	43
2.7.40	Spacelessness	44
2.7.41	Timelessness	45
2.7.42	Knowledge	46
2.7.43	Possible spiritual aspect	46
2.7.44	Possible energy	47
2.7.45	Profound	47
2.7.46	Deep	48
2.7.47	Non-changing	48
2.7.48	Subjective experience varies between meditators and across/within sessions	49
2.7.49	Description in words is limited	51
	References	51

List of Contents for Table S3 – Stillness Meditation Extraction Table

Part/section no.	Part/section name	Page
Part 1	Technique and Interim-states	1
1.1	References to Stillness Meditation	1
1.2	Simple	1
1.3	Easy	1
1.4	Pleasant	2
1.5	Regression	3
1.6	Major presentations of the practice	3
1.6.1	Meares (1967/1968)	3
1.6.2	Meares (1978/1986)	4
1.6.3	Meares (1969c)	5
1.6.4	Meares (1973b)	5

1.6.5 Meares (1989) 6
1.6.6 Meares (1987/1991) 6
1.6.7 Meares (1970) 6
1.6.8 Meares (1976b) 6
1.6.9 Elaborations in other Meares sources 7
1.6.10 McKinnon (1983/2016) 7
1.6.11 McKinnon (2011) 8
1.6.12 McKinnon (1991) 9
1.6.13 McKinnon (2002/2008) 10
1.7 Classes 10
1.8 Effortlessness 13
1.9 Mind-wandering 16
1.10 Slight/minor discomfort 17
1.11 Posture 18
1.11.1 Meares' overarching principle, and McKinnon's contrasting perspective 18
1.11.2 Definite/symmetrical posture 19
1.11.3 Lying 19
1.11.4 Sitting – general 19
1.11.5 Cross-legged sitting 20
1.11.6 Kneeling 20
1.12 Control and relinquishment, discipline and ease 20
1.13 No focus/concentration 21
1.14 Progression to goal-state/s 21
1.15 Difficulties and how to deal with them 22
1.15.1 Trying too hard 22
1.15.2 Dealing with thoughts 23
1.15.3 Letting go 23
1.15.4 Sleep 24
1.16 Unusual subjective phenomena 25
1.17 Practice duration 26
1.18 Can learn via non-class practice 26
Part 2 Goal-state/s 27
2.1 Participant-based academic studies of the subjective experience 27
2.2 Speed at which achieved 27
2.3 Features 28
2.3.1 Recognize in retrospect 28
2.3.2 Conscious 29
2.3.3 Awake, wakefulness, clearness, purity 29
2.3.4 Non-duality 30
2.3.5 Stillness 31
2.3.6 Silence, quietness 32
2.3.7 Depth/height/fullness 33
2.3.8 Relaxation 33
2.3.9 Mental rest 34
2.3.10 Emptiness, nothingness, reduced mental activity 34
2.3.11 No thoughts 35
2.3.12 No feelings 36
2.3.13 Reduced awareness/perception, loss of logical/critical faculties 37
2.3.14 No mental images 39
2.3.15 No memories 40
2.3.16 Ebb and flow 40
2.3.17 Increasing length 40
2.3.18 Calm, ease, peacefulness, absence of disturbance 41
2.3.19 Good/wonderful 43
2.3.20 Beyond calmness/stillness/goodness 45
2.3.21 No sense of anxiety 46
2.3.22 No focus/concentration 46
2.3.23 Transcendence of discomfort 47
2.3.24 Naturalness 47
2.3.25 Effortlessness 48

2.3.26	Control and relinquishment	49
2.3.27	Loss of ego	49
2.3.28	Simplicity, simple/pure being, non-doing	49
2.3.29	Nature of mind/being, self	51
2.3.30	Wholeness, integration, unity	52
2.3.31	Inner security, safety, strength, contentment	53
2.3.32	Inner freedom, unboundedness, spaciousness	54
2.3.33	Timelessness	55
2.3.34	Knowledge, understanding	55
2.3.35	Possible spiritual aspect	57
2.3.36	Profound	58
2.3.37	Deep	59
2.3.38	Subjective experience varies between meditators and across/within sessions	60
2.3.39	Description in words is limited	60
	References	61
