# Supplementary material for: Silence in Shamatha, Transcendental, and Stillness Meditation: An Evidence Synthesis Based on Expert Texts
Source: Front Psychol. 2020 Jul 8;11:1259. doi: 10.3389/fpsyg.2020.01259 (PMC7360996; doi:10.3389/fpsyg.2020.01259)
Supplement: Supplementary file 9 [file Data_Sheet_9.pdf]

Database Table S1: Shamatha Extraction Table

Table S1
Shamatha Extraction Table

Part 1: Technique and Interim-states

1.1 Key root/source texts

Wallace draws on a wide range of root/source texts. He places particular reliance on *Stages of Meditation* by Kamalashila (see, e.g., Wallace, 2006a), the *Vajra Essence* by Düdjom Lingpa (see, e.g., Wallace, 2011b), and *Small Exposition of the Stages of the Path to Enlightenment* by Tsongkhapa (see, e.g., Wallace, 1998/2005). Wallace refers to Kamalashila as an "eighth-century Indian Buddhist contemplative" (Wallace, 2006a, p. 5), and "one of the earliest Indian *mahasiddhas* (highly accomplished yogis) to teach in Tibet" (Wallace and Hodel, 2008, p. 206), and he refers to Kamalashila's text, *Stages of Meditation*, as a "classic work" (Wallace, 2006a, p. 5). Wallace (2011b) notes that Düdjom Lingpa was "a nineteenth century master of the Nyingma order of Tibetan Buddhism" (p. viii) and "one of the most realized and acclaimed Tibetan lamas of his time" (p. x). He adds that: "The *Vajra Essence* was essentially 'downloaded' from the dharmakaya, the buddha mind that is essentially coterminous with the ultimate ground of reality, and brought into our world in 1862 when Düdjom Lingpa was twenty-seven years old. He received it in a vision as a mind *terma*." (p. x); and "It is clear from the opening that this text is not scholastic in nature but is intended for those who are dedicated to practice." (p. x). Wallace (1998/2005) refers to Tsongkhapa (1357-1419) as a "Tibetan Buddhist scholar and contemplative ... renowned in Tibet as one of the greatest sages in the entire history of Indo-Tibetan Buddhism." (p. xii). "Tsongkhapa's discussion [of quiescence] in his *Small Exposition of the Stages of the Path* is remarkably thorough and yet concise, erudite and yet practical, and is marked with a high degree of philosophical and psychological sophistication." – p. xv.

1.2 Introduction to shamatha practice and the ten stages of attentional development

Wallace (2006a)	"As a framework for the gradual development of attention, I have chosen the most complete and detailed description I have found in any contemplative literature – the ten stages described by the eighth-century Indian Buddhist contemplative Kamalashila in his classic work *Stages of Meditation*." – p. 5<br>"These ten stages are sequential." – p. 6<br>"One progresses through each stage by rooting out progressively more subtle forms of the two obstacles: mental agitation and dullness." – p. 6<br>"For the first four stages, you should practice whatever method you find easiest. By stage five, the mind is relatively stable, and you can move on to subtler techniques." – p. 6<br>"For achieving the first four stages, I recommend the practice of *mindfulness of breathing*, variations of which can be found in Zen, Vipassana and Tibetan Buddhism. Mindfulness of breathing means settling your awareness on the sensations involved in your breathing, continually returning your attention there whenever your mind wanders." – p. 6<br>"Beginning with the fifth stage, I recommend a method called *settling the mind in its natural state*. In this technique, you direct your attention to mental experiences, all the events – thoughts, mental images, and emotions – that arise in the domain of the mind. This method is drawn from the *Dzogchen*, or 'Great Perfection' lineage, but is found in other Buddhist traditions as well." – p. 7<br>"With the instructions for the eighth attentional stage onward, we move on to the still subtler practice of maintaining awareness of awareness itself. The technique is called *shamatha without an object*. Here the practice is not so much one of *developing* attentional stability and vividness as it is of *discovering* the stillness and luminosity inherent in awareness itself." – p. 7<br>"The training in mindfulness of breathing may be helpful to anyone ... Many people find the second practice, that of settling the mind in its natural state, to be more challenging, but some meditators take to it naturally. Likewise, the practice of awareness of awareness is subtler still, but it may be optimal from the beginning for those who are strongly drawn to it." – p. 7<br>"You may use any one of the three methods to progress along all ten stages of attentional development, or you may follow the sequence described in this book." – p. 7<br>"Many Buddhist contemplatives have encouraged meditators who are determined to achieve shamatha to continue practicing with just one object. But Padmasambhava, the Indian master instrumental in first bringing Buddhism to Tibet, encouraged the use of multiple methods to counter the tenacious impediments to the achievement of shamatha. There are merits to both views." – pp. 79-80

Wallace (2011b)	"Within Tibetan Buddhism, shamatha practice maps on to the nine stages of attentional development wherein thoughts gradually subside as concentrative power is increased to the point at which one can effortlessly maintain single-pointed focus on a chosen object for at least four hours." – ix "... [S]ince achieving shamatha is a prerequisite to the fully effective practice of vipashyana, everyone – whether Theravada, Mahayana, or Vajrayana – has to pass through the nine stages of attentional development preceding the achievement of shamatha. Nobody bypasses them." – p. 39
Wallace (2012)	"There are ten stages, beginning with the coarsest of attentional states and leading up to the most subtle – the achievement of shamatha itself." – p. 3 "I have found [the] three practices [above] to be the most effective ones for modern people ..." – p. 5
Wallace (2011a)	"We have covered a broad range of practices, and careful discrimination is needed to determine the most suitable methods for your own needs." – p. 243 "At times you may feel relatively balanced ... and meditation may seem inviting. This is a good time for an upright posture and mindfulness of the breath at the apertures of the nostrils ... [A]n excellent practice for everyday conditions – third gear." – p. 244 "Occasionally you may feel quite refreshed, bright, and clear. Settling the mind in its natural state, which requires finesse and clarity, could be the perfect practice. When conditions are smooth, this practice allows you to travel to subtler realms – fourth gear." – p. 245 "Finally, at times when you feel optimally composed and very sharp, you can streamline your practice to awareness of awareness – fifth gear." – p. 245 "Everyone is different, and only you can tell which practice will best fit your physical and mental state at any particular time. Some people immediately gravitate to awareness of awareness. Others find settling the mind in its natural state to be a mainstay." – p. 245 "The type of meditation you engage in should be appropriate to your quality of mind at the moment. You need not perform it perfectly, but it's very important to know that you are practicing correctly." – p. 245 "... [F]or those who simply wish to reach the destination, without stopping along the way, the most direct vehicle is awareness of awareness. Here we take no interest in the mind at all, bypassing it to enter directly into the substrate. This practice is like an express elevator running directly from the surface level of the psyche to the depths of the substrate consciousness." – p. 297
Wallace (2010)	"Another possible object for [shamatha] meditation is the mind itself, as taught in the [Mahamudra] and Dzogchen traditions. Some people find it discouraging, because the object can be very elusive and yet if one can do it, it can be very, very rewarding." – p. 78
Wallace (2005)	"[The awareness of awareness practice is] the most subtle *shamatha* technique I have ever encountered. You may find that settling the mind in its natural state is subtler than mindfulness of breathing, and the *shamatha* practice of being aware of being aware is subtler still." – pp. 38-39
Wallace (2001a)	"... [I]n the practice of quiescence, one refines the attention by means of enhancing attentional stability and vividness and counteracting the mind's habitual tendencies toward alternating excitation and laxity." – pp. 210-211
Wallace (1999a)	"The types of attentional training Buddhists have devised to counteract excitation and laxity are known as *Samatha* (pronounced 'shamata'), the literal meaning of which is *quiescence*." – p. 176 "The central goals of the cultivation of *Samatha* are the development of attentional stability and vividness." – p. 177
Wallace (2018)	"While Buddhism as a whole presents a wide variety of methods for refining one's attention skills by means of training in [shamatha], the strong emphasis in ... Dzokchen is the practice of settling the mind in its natural state." – p. 47

1.3 Tsongkhapa's practice

In examining Buddhist shamatha practice, Wallace (1998/2005) begins with a detailed analysis of Tsongkhapa's guidance in the book *Small Exposition of the Stages of the Path to Enlightenment*. The main practice discussed by Tsongkhapa is not one of the three described above. "... Tsongkhapa expounds on the [shamatha] practice of imagining a visual object, specifically a mental image of the Buddha." – p. 87 "As Tsongkhapa points out ... quiescence cannot be accomplished simply by focusing the attention on a visual object; rather the actual object of [*samadhi*] must be a direct object of mental, not sensory, consciousness ... Nevertheless, the cultivation of quiescence may *begin* with focusing the attention on a physical object, then develop further by attending to a mental image of that object." – p. 146 Tsongkhapa founded the Gelugpa school of Tibetan Buddhism (Wallace, 1998/2005, p. xi), whereas

Dzogchen is part of the Nyingma school (Wallace, 2011b, p. 184). Wallace (1998/2005) notes that, "Tsongkhapa's writings on the whole do not explicitly deal with the [Dzogchen] tradition ..." – p. 239 However, he also notes that Tsongkhapa received instructions on Dzogchen, and later praised them as being "free of excess, omission, and error" (p. 239). In the latter part of his book, Wallace (1998/2005) discusses the relationship between Tsongkhapa's views, and presentations of shamatha practice in Dzogchen and other Buddhist traditions. Since Tsongkhapa's presentation informs Wallace's understanding, this table includes a small number of references to it.

1.4 Mindfulness and introspection

1.4.1 Overarching guidance

Wallace (2006a)	"Mindfulness and introspection go hand in hand ..." – p. 65<br>"Throughout Buddhist literature, the training in shamatha is often likened to training a wild elephant, and the two primary instruments for this are the tether of mindfulness and the goad of introspection." – p. 79
Wallace (2012/2014)	"Mindfulness, introspection, and samadhi are three faculties central to all forms of Buddhist meditation ..." – p. 54
Wallace (2011a)	"... [M]aintaining a continuity of focused attention ... is at the heart of all practices." – p. 33<br>"Mindfulness and introspection are classically described as mental faculties ..." – p. 54 "There are no disagreements or sectarian issues here; in fact, there is a strong congruence in the usage of these two key terms throughout Indian, Theravadin, Zen, and Tibetan Buddhist traditions." – p. 55
Wallace (2010)	"There are two distinct qualities of awareness to cultivate in the [shamatha] practice: mindfulness (*smrti*) and introspection (*samprajanya*). They are defined very specifically in the Tibetan Buddhist context, which is somewhat different from the [Theravada] context." – p. 47
Wallace (1999a)	"... [T]wo mental faculties are said to be indispensable for the cultivation of attentional stability and vividness, namely, mindfulness and introspection." – p. 178

1.4.2 Mindfulness

Wallace (2006a)	"The faculty of *mindfulness* is crucial in shamatha practice. Mindfulness in this context differs somewhat from the way some contemporary meditation teachers present it. Vipassana teachers, for instance, commonly explain mindfulness as moment-to-moment, nonjudgmental awareness of whatever arises. In the context of shamatha, however, *mindfulness* refers to attending continuously to a familiar object, without forgetfulness or distraction." – p. 13<br>"In the Indian and Tibetan Mahayana traditions, mindfulness is defined as the mental faculty of maintaining attention, without forgetfulness or distraction, on a familiar object. Since mindfulness prevents the attention from straying from one's chosen object, it acts as the basis for single-pointed focused attention, known as *samadhi*." – p. 59
Wallace (2012/2014)	"One mental faculty that is indispensable to all forms of Buddhist meditation is mindfulness (Pali *sati*)." – p. 51
Wallace (2012)	"*Mindfulness* can be defined as continuous attention to a chosen object, which requires that one remember what the task is and not become distracted by other phenomena." – p. 9<br>The glossary defines mindfulness as "[c]ontinuous attention to a chosen object, which requires that one remember what the task is and not become distracted by other phenomena" (p. 161).
Wallace (2011a)	"Buddhaghosa's definition of mindfulness continues: 'It's characteristic is not floating'. In our practice of mindfulness, it's very easy to float. When we are not latched on to something ... then it's very easy to simply space out and float. We are so accustomed to compulsively grasping, clinging, identifying with our thoughts, and mistaking them for their referents that when we release our grasp and simply try to be present, we often find ourselves floating – or sleeping! Mindfulness means not floating, not forgetting, and not disengaging." – pp. 56-57 "We are not spacing out or grasping but mindfully present." – p. 57<br>"Buddhaghosa's definition [of mindfulness] continues: 'Its basis is strong noting ...' This means that the basis of mindfulness is strongly engaged attention that notes well, without grasping or clinging." – p. 57

Wallace (2010)	"The sole task of mindfulness is to attend to the object with continuity. It's like the beating of the heart: you always want it to be happening. If it's not there, then try to get it back as quickly as you can." – p. 47
Wallace (2001/2003)	"Mindfulness is the ability to attend to a chosen object with continuity and without distraction. Mindfulness is different from concentration. Concentration entails condensing the attention to a narrow focus. Mindfulness is a state of stable attention that may be wide open and spacious or tightly focused, as one desires." – p. 77
Wallace (2000)	"The task of mindfulness is to attend without distraction to a familiar object of attention ..." – p. 106
Wallace and Wilhelm (1993)	"Mindfulness is a mental factor that allows us to focus upon an object with continuity, without forgetting that object." – p. 110
Wallace (2018)	"... [T]he terms translated as 'mindfulness' in [Pali] (*sati*) [and] Sanskrit ([*smrti*]) ... primarily connote recollection, or bearing in mind." – p. 7

1.4.3 Introspection

Wallace (2006a)	Guidance given at stage 3: "As you continue in this practice ... you need to hone the ability to monitor the quality of your attention. While the main force of your awareness is directed to the meditation object with *mindfulness*, this needs to be supported with the faculty of *introspection*, which allows for the quality control of attention, enabling you to swiftly note when the mind has fallen into either excitation or laxity." – p. 44<br>"*Introspection* is a mental faculty having the function of monitoring the state of one's body and mind. With this faculty we note when the mind has succumbed to either laxity or excitation ..." – p. 65<br>"The *power of introspection* is the faculty of monitoring the quality of your attention ..." – p. 78
Wallace (2009/2014)	"In the Buddhist tradition, introspection is defined as the repeated examination of the state of one's body and mind, and it is regarded as a kind of discerning intelligence." – p. 63<br>"... [W]ith mindfulness we focus continually, without forgetfulness ... and with introspection we closely examine whether the attention has been caught up in laxity or excitation. Thus, introspection 'looks over mindfulness' shoulder'." – pp. 63-64
Wallace (2012)	"... [I]ntrospection allows for a kind of quality control, recognizing when one's attention strays and alerting mindfulness to reassert itself. One focuses one's mind using mindfulness to remember the task, and when attention strays introspection takes notice so that one can guide oneself back to the object of attention." – p. 9
Wallace (2011a)	"Introspection is an expression of intelligence (Skt. [*prajna*]) because it is discerning ... Am I practicing correctly or not? Am I sustaining a flow of mindfulness, or have I fallen into distraction, excitation, laxity, or dullness? Introspection is the quality control monitor for the entire process, repeatedly examining the state of one's body and mind." – p. 58 "Introspection monitors the meditative process and recognizes attentional imbalances: spacing out, laxity, dullness, sleepiness, restlessness, excitation, distraction, agitation, and so forth." – p. 59
Wallace (2010)	"While mindfulness is attending to the meditative object, introspection is attending to the meditating mind, checking on how it's going: 'Am I still trying to control the breath? Am I chattering about the breath as I'm watching it? Am I falling asleep? Am I spaced out?' Introspection also has the task of checking up now and again on the body. Check out the posture ... If you are accustomed to proper meditation, you may find that you have a reliable posture, and it doesn't need much introspection. In earlier phases of meditation, or if you are experimenting with different postures, attention to the body is more important. But the chief task of introspection is to monitor the mind, because the mind tends to change faster than the posture does." – p. 47<br>"Note that introspection is not on all the time ... [Y]ou ... want it poking in intermittently." – p. 48<br>"Introspection needs to be more frequent in the earlier phases of the practice, both towards the mind and the body. Eventually you will learn to rest in a stable posture, and introspection will no longer be necessary for the body." – p. 48 "As you progress, introspection is not needed so often, but it must become more educated and more acute. The types of problems to attend to become more subtle." – p. 48 "By the time that you've moved through gross and subtle excitation and countered both gross and subtle laxity, you're on easy street. From that point, you no longer need introspection. In fact, introspection then becomes a nuisance and detracts from the meditation. It's not a line that you cross, but a stage you move into gradually. There may

	be times even relatively soon in your meditation when you can honestly say, 'I don't need to do anything here'." – p. 49
Wallace (2005)	"... [I]t's crucial to apply introspection intermittently throughout the session ... Introspection entails quality control, monitoring the processes of both the mind and the body." – p. 15
Wallace (2001/2003)	"Introspection is the ability to observe the state of one's mind from moment to moment." – p. 77 "... [W]hen you approach the achievement of quiescence ... the intervention of introspection ... becomes an impediment. When quiescence is achieved, relinquish introspection, release grasping so deeply that introspection itself is released." – p. 133
Wallace (2000)	"... [T]he function of introspection is to monitor the attending awareness." – p. 106
Wallace (1999a)	"... [I]ntrospection has the function of monitoring the meditative process." – p. 178
Wallace (1999b)	"... [I]ntrospection is a kind of meta-cognition that attends to the quality of the attention itself." – p. 443

1.5 Relaxation, stability, vividness, equanimity

1.5.1 Overarching guidance

Wallace (2006a)	"... [T]he initial emphasis in shamatha practice is on relaxation ... The second emphasis is on stability of attention ... Then, having established a foundation of relaxation and stability, we shift the emphasis to cultivating vividness of attention. It is crucially important that stability is not gained at the expense of relaxation, and that the increase in vividness does not coincide with the decrease of stability." – p. 46
Wallace (2011b)	"... [Settling the mind in its natural state] is a practice of deepening relaxation such that stability simply emerges out of deepening levels of relaxation. As the stability deepens because the relaxation is deepening, then clarity and vividness arise right out of the stability. Relaxing then goes deeper, and as you become more and more relaxed ... the stability of the mind just naturally emerges from relaxation. Then, in turn, as the relaxation goes deeper, the stability goes deeper. As stability goes deeper, then further clarity emerges out of the stability ... All three qualities of relaxation, stability and vividness emerge synergistically ..." – p. 139
Wallace (2012/2014)	"One of the most intriguing aspects of Buddhist attentional training concerns the interrelations among the three qualities that are developed in such meditation: relaxation, stability, and vividness. In many scientific studies of attention in normal people – including those with skills such as controlling air traffic, performing music, developing mathematics, and playing chess – psychologists have found an inverse relation between relaxation and attentional arousal. When one is deeply relaxed, there is a low level of attentional vividness, and when one's attention is highly aroused, this is accompanied by intensive effort." – p. 166 "In Buddhist attentional practice, on the contrary, one first emphasizes the cultivation of mental and physical relaxation. Upon this basis, attentional stability is developed. Finally, one focuses on the generation of attentional vividness. This sequence is important – it must not be mixed up." – p. 166
Wallace (2012)	"You need all three qualities, and they should be in synergistic balance. If relaxation is too strong, you are likely to become dull and sleepy. If vividness is too bright and energetic, you may become agitated." – p. 16 "The overall strategy ... is to allow stability to develop from relaxation and vividness to emerge from stability." – p. 17
Wallace (1989/2003)	"The mental qualities to be cultivated ... are clarity, stability, and relaxation. All of us have experienced periods of mental clarity and intense alertness, but at such times the mind is not usually very stable or relaxed. Such moments may occur, for example, while watching a thrilling movie or engaging in a fast-paced, competitive sport. We can also recall times when our minds were stable and calm – for example, after vigorous exercise or just before sleep – but on those occasions vivid mental clarity is rarely experienced. Thus, the emphasis in this training is to strive first of all for mental stability ... As such stability is acquired, one then seeks to heighten one's mental clarity." – p. 195
Wallace and Wilhelm (1993)	"All of us have experienced moments when our attention is extremely vivid. This may occur, for example, while driving a car or motorcycle at high speed on a winding road, or when rock-climbing. But when such

	mental clarity is experienced it is usually combined with a high degree of tension, and the mind is neither serene nor stable. On the other hand, mental stability is a common experience when we are pleasantly tired and we lie down to sleep. But in such cases there is rarely much clarity of awareness." – p. 112 "The challenge of meditative quiescence practice is to cultivate stability integrated with clarity, generating an extraordinarily useful quality of awareness." – p. 112
Wallace (1999a)	"... [T]he development of attentional stability may be likened to mounting one's telescope on a firm platform; while the development of attentional vividness is like highly polishing the lenses and bringing the telescope into clear focus." – p. 177

1.5.2 Relaxation

See also 1.12.4 ("Practice 1 – Mindfulness of breathing – Dealing with thoughts, distractions and discomfort").

Wallace (2006a)	"... [C]oncentration [in the mindfulness of breathing practice] should not be tense but rather balanced. When we discover that we have become distracted from the meditation object, it may feel natural to clamp down more forcefully, tightly concentrating the mind ... If you want to concentrate for a short time and don't mind the side effects of tension and fatigue, you can follow the above strategy. But if you want to follow the path of shamatha, you'll need an alternative." – pp. 14-15<br>Wallace describes his first extended shamatha retreat: "As the weeks went by ... I found myself becoming more and more fatigued. I was draining myself both physically and mentally ... What was wrong? I was trying too hard. The cultivation of shamatha involves balancing the mind, and that includes balancing the effort exerted in the practice with relaxation." – p. 15<br>"Before we can develop attentional stability, we first need to learn to relax." – p. 16<br>"The stability of your attention should emerge from a relaxed mind, not work against it." – p. 31<br>"Meditation is a balancing act between attention and relaxation. Mastering this requires working to counter the natural reflex of trying harder, or calming down, when you see that your mind has become distracted." – p. 32<br>"Generally, the more relaxed we are, the more stable our attention can become. But that relaxation must be balanced with vigilance, otherwise it will lead to laxity, sluggishness, or unbridled daydreaming. Once we have established a foundation of relaxation, we can more strongly emphasize attentional stability." – p. 33<br>"The practice of shamatha results in an anomalous kind of attention. Normally, when the mind is relaxed, attention is slack, and when attention is aroused, this brings with it a state of tension, a tightening of the body and mind. But in this practice, the more you arouse your attention, the more deeply the mind relaxes." – pp. 48-49<br>In the context of stage 4 mindfulness of breathing: "As [conceptual] superimpositions [such as habitual mental images] are released ... you enter deeper and deeper levels of tranquility." – p. 63
Wallace (2011b)	"Because many of us are so wound up, the first step is to relax. Our minds are so stressed out, cluttered, and unstable – so just relax." – p. 115<br>"Given the balance of relaxation, stability, and vividness required, trying your hardest is trying way too hard." – p. 155
Wallace (2012)	"Although in shamatha we are developing concentration, it is achieved not by force but through deep relaxation. This is not the stressful and ultimately exhausting concentration of the fighter pilot ..." – p. 2<br>"A sense of ease and relaxation is indispensable for cultivating attention skills ..." – p. 11
Wallace (2011a)	"Relaxation is the primary quality to be cultivated, and it must never be sacrificed for stability or vividness." – p. 89<br>"In the contemplative practice of shamatha, we simultaneously enhance the stability and vividness of attention while relaxing increasingly deeply. This is a skill not taught in the ordinary world, where highly aroused attention is generally accompanied by a sharp focus, strong effort, and contraction. People who cultivate their attention in skillful shamatha meditation can sustain a state of very high arousal and vigilance, with extraordinary vividness and stability of attention and can still feel utterly fresh after many hours." – p. 250
Wallace (2005)	"Let me emphasize that this is not a concentration technique in the Western sense. We are not bearing down with tight, focused effort." – p. 15

1.5.3 Stability

Wallace (2006a)	"[Attentional stability] is the ability to sustain the focus of your attention without becoming fragmented or derailed by the force of distracting thoughts and sensations." – p. 31
Wallace (2012/2014)	"... [S]tability refers to the ability to sustain one's attention upon the chosen object without forgetfulness or distraction." – p. 187
Wallace and Hodel (2008)	"'Stability' refers to maintaining concentration steadily on the chosen object – one does not stray." – p. 207
Wallace (1989/2003)	"[Stability] allows one to remain focused on the chosen object without being pulled away by conceptual or sensory distractions." – p. 195

1.5.4 Vividness

Wallace (2006a)	"You [shift the emphasis to vividness] by elevating the focus of attention and directing it to a subtler object ... Elevating the focus of attention helps to induce vividness, and attending to a subtle object enhances that further." – p. 46
Wallace (2011b)	Wallace p. 139 uses the term clarity as a synonym for vividness.<br>In the context of the settling the mind in its natural state practice: "Two types of vividness emerge ... One we can call temporal vividness, by which you are able to detect increasingly brief mental events – thoughts, images, impulses – that previously went by so quickly you never noticed them. The other is qualitative vividness. This enables you to detect increasingly subtle mental processes that may linger for seconds on end, but so quietly and unobtrusively that they escaped your attention until now. With such vividness of both kinds, mental states and processes that were previously unconscious are now illuminated with the clear light of consciousness." – p. 139
Wallace (2012/2014)	"[*V*]*ividness* ... includes qualitative and temporal aspects. We may draw on the qualitative vividness of attention to examine the constituent parts of physical phenomena down to the individual particles, or material atoms, of which they are composed. Drawing on the temporal vividness of attention, we may inspect the arising and passing of individual moments of both physical and mental phenomena." – p. 131<br>"The quality of vividness is of two kinds, temporal and qualitative." – p. 188 "Temporal vividness refers to the ability to ascertain very brief, fleeting events ..." – p. 188 "Qualitative vividness refers to the ability to ascertain events that ... are so subtle we fail to recognize them." – p. 188
Wallace (2012)	Increasing vividness is increasing "clarity, luminosity, and brightness" (p. 17).<br>On mindfulness of breathing: "The technique for increasing vividness is to focus your attention on a subtler object, such as the sensations of the breath in the area of the nostrils" (as opposed to the tactile sensations at the abdomen) (p. 17).
Wallace (2011a)	"The dimension of vividness or acuity has both spatial and temporal aspects. Qualitative vividness means high-resolution perception that captures fine details, like high-definition television. Temporal acuity means very rapid observations." – p. 156
Wallace (2010)	"If, in the course of a session, your sense of ease is sustained ... and the continuity is really quite good, you may find yourself beginning to sink into the object. This is a premature phasing out of duality, merging with the object in a way that is not useful, like slipping down into mud. At that point, it's time to exert more effort and increase the vividness." – p. 39<br>"Improving vividness is like focusing more and more finely with the lens of your attention: one of the characteristics of enhanced vividness is that you see greater detail." – p. 62
Wallace and Hodel (2008)	"'Vividness' refers to the degree of clarity or closeness of attention one maintains on the object." – p. 207
Wallace (1989/2003)	"[Clarity/vividness] allows one to examine the fine details of the object." – p. 195
Wallace and Wilhelm (1993)	"Clarity refers more to the vividness of subjective awareness than to the clarity of the object. When it is present we can detect even the subtle and most fleeting qualities of our object." – p. 111

1.5.5 Equanimity

Wallace (2006a)	"Underlying [relaxation, stability and vividness] must be a foundation of equanimity, without which strong attentional and emotional vacillations will likely persist indefinitely." – p. 68

1.6 Bare awareness/attention

Wallace (2006a)	In the stage 2 mindfulness of breathing instructions: "The kind of awareness cultivated here is called *bare attention*, in which the mind is fully focused on the sensory impressions appearing to it, moment to moment, rather than getting caught up in conceptual and emotional responses to those stimuli." – p. 33<br>"... [Bare attention involves] taking the 'fresh produce' of the world straight from the fields of the senses, without prepackaging raw experience with our old, habitual conceptual wrappings. The challenge here is to distinguish what reality is presenting to our senses from moment to moment from what we are superimposing on the world, often unconsciously. This is what the Buddha was referring to when he declared, 'In the seen there is only the seen; in the heard, there is only the heard; in the sensed, there is only the sensed; in the mentally perceived, there is only the mentally perceived'." – p. 37<br>In the context of stage 7: "The practice of settling the mind in its natural state corresponds closely to the psychological description of mindfulness ... as 'a kind of nonelaborative, nonjudgmental, present-centered awareness in which each thought, feeling, or sensation that arises in the attentional field is acknowledged and accepted as it is' [quoting Bishop et al., 2004]." – p. 119 "This description ... reflects the contemporary Vipassana tradition's account of mindfulness as a kind of moment-to-moment 'bare attention' or nonconceptual awareness that does not label or categorize experiences." – p. 119 Wallace p. 120 refers to "the shamatha practice of bare attention applied to the domain of the mind".
Wallace (2011b)	"[The settling the mind in its natural state] practice presents both enormous possibilities and pitfalls ... When they actually occur they tend to be overwhelming. At least memorize what to do when that happens. Understand that [stuff that comes up in the practice] are just nyam ... [R]emember that when you are *actually practicing*. This is where the cultivation of mindfulness entails much more than merely being present with whatever appearances are arising here and now. In Buddhist practice mindfulness also means remembering the teachings you've received so that you can apply them when the appropriate situation arises." – pp. 165-166
Wallace (2009/2014)	On the settling of the mind in its natural state practice: "The kind of awareness we are bringing to the mind is discerning and intelligent, but also nonjudgmental. We are not evaluating one thought as being better or worse than another. You may find at times that you are compulsively engaging in a kind of internal commentary, as if you were ... a critic judging the performances of each of the actors. Give it a rest, and simply observe what's happening on the stage of your mind without commentary. And if internal judgments arise anyway, simply observe them; they too are contents of the mind and therefore grist for the mill." – p. 50<br>Wallace p. 61 notes the Bishop et al. (2004) definition of mindfulness (see entry for Wallace, 2006a in this section above). "That's a good description of the kind of awareness we bring to [the settling the mind in its natural state] practice ..." – pp. 61-62
Wallace (2012)	"*Mindfulness* can be defined as continuous attention to a chosen object, which requires that one remember what the task is and not become distracted by other phenomena." – p. 9<br>In mindfulness of breathing practice: "... [W]ith bare attention simply attend to the sensations themselves, with no conceptual overlay, no cogitation ..." – p. 11
Wallace (2011a)	"Our practice – approaching bare attention, being focused and attentive from moment to moment, and not reacting to whatever is arising – is not yet comprehensive, but it is a fundamental expression of mindfulness." – pp. 57-58<br>In the settling the mind in its natural state practice: "It is crucial to attend very closely, intelligently, and discerningly, being utterly present – but without identification, grasping, aversion, suppression, or dissociation." – pp. 195-196<br>"The practice of settling the mind in its natural state entails a quality of heightened interest and discerning intelligence that is clear, luminous, present, engaged, and highly attentive." – p. 240
Wallace (2005)	Discussing the mindfulness of breathing practice: "In Tibetan and Sanskrit, the word translated as *mindfulness* also means *remembering*. So the cultivation of mindfulness means maintaining an unbroken

flow of remembering ... It doesn't involve any internal commentary. You are simply remembering to attend to the stream of tactile sensations of the in- and out-breaths. The quality of awareness you are cultivating here is a kind of bare attention, a simple witnessing, with no mental analysis or conceptual elaboration." – p. 15
"... [*B*]*are attention* [is] a quality of awareness that is relatively free of conceptual prestructuring, filtration, modification, interpretation, and projection. It's not absolutely free of such conceptual superimpositions. It is unlikely that we can just flick a switch, bringing compulsive conceptual activity to a halt. But we can attenuate it, making it less compulsive ..." – p. 55
"Give your conceptual mind a rest from its normal exertions. Rest your awareness in bare perception, quiet attentiveness." – p. 66

1.7 Excitation and laxity

1.7.1 Overarching guidance

Wallace (2009/2014)	"The two major attentional imbalances we tend to encounter when engaging in meditative practice are laxity and excitation ... Both laxity and excitation result in a loss of mindfulness, by the attention either collapsing in on itself or being compulsively propelled outward." – p. 63
Wallace (2012)	"... [T]here are two major types of distraction that cause us to forget our task: *agitation* and *dullness*." – p. 13
Wallace and Wilhelm (1993)	"There are many inner hindrances to stabilizing the mind, but they boil down to the two extremes of excitement and laxity." – p. 111

1.7.2 Excitation

Wallace (2006a)	"For most people setting out on the path of attentional development, the problem that overwhelms them is *excitation*. There are many reasons the mind becomes agitated and distracted. Anger and fear certainly have this influence, and simply living in a noisy, hectic environment can easily destabilize the mind. But most commonly, the coherence and continuity of attention is undermined by craving, or misguided desires." – p. 29 "The general symptoms of a mind prone to craving are dissatisfaction, restlessness, and anxiety." – p. 29<br><br>Wallace notes that there are three levels of excitation: coarse, medium and subtle (p. 29).<br><br>"When coarse excitation takes over the mind, we completely lose touch with our chosen object of attention." – p. 29<br>"Coarse excitation: The attention completely disengages from the meditative object." – p. 174<br><br>"When medium excitation occurs, you don't completely lose track of your object of attention, but involuntary thoughts occupy the center of attention and the meditative object is displaced to the periphery." – p. 62. Wallace p. 174 makes a similar comment.<br><br>"... [With] subtle excitation ... the meditative object remains at the center of attention, but involuntary thoughts emerge at the periphery." – p. 99. Wallace p. 174 makes a similar comment.
Wallace (2011b)	Wallace p. 184 defines excitation as "[m]ental hyperactivity that prevents focus".
Wallace (2009/2014)	"When excitation arises, the mind becomes distracted and agitated, making it difficult to sustain our attention on anything with continuity." – p. 63
Wallace and Hodel (2008)	The authors refer to distraction as including "daydreams, sense data, strong emotions, mental chatter, and the like." – p. 207
Wallace (1999a)	"Excitation ... is defined as an agitated, intentional mental process that follows after attractive objects ..." – p. 176 "A mental process is said to be *intentional*, not because one intends for it to occur, but because it has its own cognized object or objects." – p. 176

1.7.3 Laxity

Wallace (2006a)	"From the beginning of shamatha training ... some people are more prone to laxity ..." – p. 44<br><br>Wallace notes that there are three levels of laxity: coarse, medium and subtle (p. 44).<br><br>"[*C*]*oarse laxity* ... occurs when your attention mostly disengages from the object and sinks into a spaced-out vacancy ... Abiding in a state of coarse laxity can be very peaceful, with your mind relatively undisturbed by thoughts or emotional upheavals. But if you spend many hours each day in such a state of dullness, Tibetan contemplatives report that this not only has no benefit, it can actually impair your intelligence. The acuity of your mind starts to atrophy, and over the long term, this can do serious damage." – p. 44<br>In the context of stage 5: "... [T]he symptom of [coarse laxity] is that your attention succumbs to dullness, which causes it to largely disengage from its meditative object ... The attention fades, as it were, but instead of fading out, it's more like fading in, stepping onto a slippery slope that leads down to sluggishness, lethargy, and finally sleep. This is a peaceful state of mind, so the ignorant may mistake it for the attainment of shamatha, which literally means quiescence, tranquility, and serenity. True shamatha is imbued not only with a degree of stability far beyond that achieved at this stage of attentional practice but also with an extraordinary vividness that one has hardly begun to develop at this point in the training." – p. 77<br>"Coarse laxity: The attention mostly disengages from the object due to insufficient vividness." – p. 175<br><br>"When [a medium] degree of laxity sets in, the object of meditation appears, but without much vividness." – p. 78 Wallace pp. 99, 175 makes similar comments. "This is subtly different from coarse laxity, and you will discover that distinction only through practice." – p. 78<br><br>"... [With] subtle laxity ... the object of mindfulness appears vividly, but your attention is slightly slack." – p. 117 Wallace p. 175 makes a similar comment. "No one but a highly advanced meditator is even capable of recognizing such a subtle degree of laxity. It is detected only in relation to the exceptionally high degree of vividness of which the trained mind is capable." – p. 117
Wallace (2009/2014)	"When laxity sets in, the mind loses its clarity and we become spaced out or simply fall into a dull, sluggish state on the way to falling asleep." – p. 63
Wallace (2012)	"When ... we are ... dull ... we find our focus hazy. The object of attention lacks vividness. We tune out, surfacing minutes later from daydreams or sleep." – p. 13<br>The glossary defines "dullness, laxity" as "[a] state where one's attention is unfocused, hazy, and tending to drowsiness" (p. 161).
Wallace (2010)	"... [I]f laxity goes farther, it progresses to lethargy ... in which you just feel heavy. Beyond lethargy is sleepiness ... when you begin to nod off. With laxity you have just lost the edge, you're not falling asleep yet." – p. 39<br>"*Question*: After just a little while of counting my breath, I fade out and I don't come to until you ring the bell. Is this what you mean by laxity? *Response*: It is. If you are simply fatigued, it's better to rest." – p. 42
Wallace (2000)	"... [S]ince laxity is difficult to identify, under its influence one may easily overestimate the quality of one's attention." – p. 107
Wallace (2018)	The glossary defines laxity as "[t]he loss of clarity of attention, which is counteracted through the cultivation of vividness in the practice of [shamatha]." – p. 217

1.7.4 Remedies

Wallace (2006a)	"As soon as you detect [the imbalance of excitation or laxity], take the necessary steps to remedy it." – p. 44 Wallace p. 65 makes a similar comment. "Your first antidote to excitation is to relax more deeply; to counteract laxity, arouse your attention." – p. 44<br>"The way to counteract laxity is to arouse the attention, to take a greater interest in the object of meditation." – p. 78<br>In the context of stage 5 settling the mind in its natural state: "... [T]he task at this point is to determine the proper 'pitch' of attention. If you arouse the mind too much in your efforts to remedy laxity, it will easily fall into excitation, but if you relax too much, you will likely succumb to laxity. It's a delicate balancing

	act, and the only way you can meet this challenge is through your own experience, determining for yourself the suitable degree of effort to tune your attention." – p. 78 "With fine-honed introspection, you quickly detect attentional imbalances, and you then take the necessary measures to restore balance." – p. 84 "As the practice becomes subtler, you have to discover for yourself the optimal degree of mental arousal, or tension." – p. 88 "Settling the mind in its natural state, like all shamatha practices, is a balancing act. If you constrict the mind too much, you will become exhausted and stressed out. If you let your mind go too slack, the clarity of attention will fade ..." – p. 89<br>"When laxity sets in, you arouse your attention; and when excitation occurs, you loosen up slightly." – p. 117
Wallace (2009/2014)	"With the faculty of introspection we monitor the quality of our attention, noting as swiftly as possible when either of these two imbalances occurs." – p. 63<br>"When we recognize that laxity has arisen, the immediate remedy is to take a fresh interest in the object of mindfulness. In this case, we apply more effort as we sharpen the focus of attention. In the other case, as soon as we note that the mind has become distracted and is caught up in thoughts, the antidote is to relax a bit, both physically and mentally." – p. 64<br>In the context of the awareness of awareness practice: "When through introspection you notice that your mind has become caught up in distracting thoughts or other stimuli, immediately relax and let go of those objects of the mind. It takes effort to maintain your grip on those distractions, so release that effort as well as the objects that distract you, and let your awareness return home. This is like settling into a deep, dreamless sleep. But instead of gradually losing the clarity of awareness as you normally do when you fall asleep, you maintain a high level of vigilance." – p. 73
Wallace (2012)	"Relaxation and stillness counter agitation, while vigilance counteracts dullness." – p. 13
Wallace (1998/2005)	In the section on Tsongkhapa's vision/understanding:<br>"... [W]hen the mind is no longer prone to [excitation and laxity], it is essential to release the effort that had previously gone to counteracting them. At this point, if one continues to exert the same amount of effort as before, this impedes further progress, for it agitates the mind." – p. 187
Wallace (2001/2003)	"When with introspection you note that the mind is rambling, just draw it back, again and again. If you attend to what is happening in your mind when it is happening, you won't get carried away on the many express trains of imagery and conversation that pass through. The technique is to remain still while your mind is in action. This is very different from the mind burbling on and you burbling along with it." – p. 78
Wallace (1999a)	"Laxity ... is an intentional mental process that occurs when the attention becomes slack and the meditative object is not apprehended with vividness and forcefulness." – p. 176 "A mental process is said to be *intentional*, not because one intends for it to occur, but because it has its own cognized object or objects." – p. 176

1.8 Will

Wallace (2009/2014)	"Simply recognizing that our attention is getting dull or agitated is not enough. As soon as we notice this, we need to exert the right amount of effort to overcome the imbalance. This is an act of will." – p. 64
Wallace (1998/2005)	In the section on Tsongkhapa's vision/understanding:<br>"... [O]ne may recognize the presence of laxity or excitation and still fail to take steps to counteract them ... The remedy, Tsongkhapa declares, is the cultivation of the will, which is here closely associated with intervention and striving ... [W]hen either laxity or excitation occurs, the mind is stimulated by the will to intervene in order to eliminate them ..." – pp. 87-88 At the advanced stage where "effortless, natural [*samadhi*] arises", "the engagement of the will ... is actually a hindrance" (p. 88). At this point: "It is time to let the natural balance of the mind maintain itself without interference." – p. 88
Wallace (1999a)	"... the cultivation of the will, which is here closely associated with intervention and effort." – p. 180

1.9 Mind-wandering

Wallace (2011a)	"Shamatha develops the ability to ascertain whether your mind wanders or not. Achieving stability means that your mind does not wander. Introspection is refined and sharpened like a surgical scalpel to detect and excise even the subtlest attentional perturbations and imbalances." – p. 267

1.10 Non-doing, effortlessness, relinquishment of control, loss of ego

See also 1.8 ("Will"), 1.11 ("The ten stages"), 1.12 to 1.14 (practices 1 to 3), and 1.20 ("Other experiences").

Wallace (2006a)	In the context of stage 3 mindfulness of breathing: "There's a relatively egoless quality to shamatha; while other activities that call for a high level of concentration are effortful and often goal oriented, shamatha entails doing almost nothing. You're passively attending to the sensations of the breath without regulating it in any way. Your ego is mostly taken out of commission as you let the body breathe of its own accord, exerting only a subtle degree of effort to balance attention when it falls into laxity or excitation." – p. 49<br>"In the practice of mindfulness of breathing, you are faced with the challenge of carefully observing, *without controlling*, sensations within the body associated with the breathing. Now [in the settling the mind in its natural state] practice you face a similar challenge of carefully observing events within the mind without regulating or evaluating them in any way." – p. 91<br>"In each of the two shamatha methods introduced thus far – mindfulness of breathing and settling the mind in its natural state – the practices gradually involve doing less and less. When mindfully attending to the breath, there is a great deal you are not doing, but you are still releasing involuntary thoughts when they arise. You do prefer to have a conceptually silent mind, as opposed to having discursive thoughts and images arise one after the other. When settling the mind in its natural state, you are doing even less. Now you don't even prefer thoughts to be absent. Instead of deliberately letting them go – banishing them from your mind – you let them be, without deliberately influencing them in any way. You simply maintain constant mindfulness of the space of the mind and whatever events occur in that space." – p. 118<br>"[From achievement of the eighth stage] onward, you can flow with the momentum of the practice with little or no effort ... Only the slightest degree of effort at the beginning of each session is need[ed] to ward off [the] obstacles [of excitation and laxity] ..." – p. 131<br>In the context of stage 8: "[The awareness of awareness] practice is one of profound inactivity. You are *being* aware of being aware, but you are not really *doing* anything." – pp. 136-137<br>At the ninth attentional stage: "... [Y]ou can slip into meditative equipoise ... with no effort at all." – p. 143
Wallace (2011b)	Referring to the settling of the mind in its natural state practice: "*Let thoughts be as they are*. This means you're not intruding on them ... You're not trying to do anything to the thoughts at all." – p. 116<br>"In [the settling the mind in its natural state] practice the greatest effort comes at the beginning. In the final stages, it's just effortless ... [I]n the course of the practice of shamatha, whatever technique you are following, the degree of effort you need to exert to practice correctly tapers off as you approach accomplishment, and then simply vanishes. You will have far greater stability and vividness than you started off with, but the effort to maintain and establish that vivid, steady apprehension vanishes of its own accord ... You arrive at a point in the practice where, if you keep on exerting effort, keep on applying introspection to try to fix something, you're actually cluttering up your practice." – p. 155 "... [I]n the advanced stages of shamatha you are on a conveyer belt to the substrate consciousness. Effort is no longer required to achieve this ... very significant, accomplishment." – p. 156<br>"In the course of this practice you may at times wonder, 'What's going on? I'm really not doing anything. I am doing the practice correctly, but I am just sitting here ... Such thoughts can easily appear, because you truly are *doing* so little ... The very fact that you are doing nothing so luminously and clearly is facilitating subconscious processes – a balancing, a healing, an illumination, an opening up, a purification." – p. 157
Wallace (2009/2014)	Instructions for mindfulness of breathing practice: "Each time your attention gets caught up in sensory stimuli or thoughts and memories ... release your mind from these preoccupations, and gently return to your breath. Let your attention remain within the field of sensations of your body, and let the world and the activities of your mind flow around you unimpeded, without trying to control or influence them in any way." – p. 40<br>On the settling of the mind in its natural state practice: "When we begin this practice, the normally agitated mind is like a snow globe that has just been shaken, and a flurry of memories and fantasies swirl around, swiftly emerging and disappearing. Settling the mind in its natural state involves letting all these mental activities arise without inhibiting, controlling, or modifying them in any way." – p. 49 "Whatever events arise in your mind – be they pleasant or unpleasant, gentle or harsh, good or bad, long or short – just let

	them be." – p. 50 "As soon as you note that you are distracted, loosen up your body and mind and release your grasp on the thoughts that have captured your attention. This doesn't mean expelling the thoughts themselves. On the contrary, let them continue of their own accord for as long as they persist. But release the effort of identifying with them." – p. 50<br>"Clearly, the benefits of settling the mind into its natural state do not result from 'my' doing anything to the mind. Rather, I am simply observing events arising and passing in the mind, without trying to alter any of them." – p. 59<br>"While engaging in the practice of settling the mind in its natural state, we do not try to alter any of its contents but simply observe whatever arises with unwavering mindfulness. But in the course of our daily lives, mindfulness can play a more active role ... in helping to cultivate wholesome mental states and alleviate unwholesome ones ... Many Buddhist treatises describe mindfulness as ... a mental faculty that exerts a controlling influence on thoughts and intentions. So it is incorrect to assume that mindfulness is always passive." – pp. 62-63<br>In the context of the awareness of awareness practice: "When through introspection you notice that your mind has become caught up in distracting thoughts or other stimuli, immediately relax and let go of those objects of the mind. It takes effort to maintain your grip on those distractions, so release that effort as well as the objects that distract you, and let your awareness return home." – p. 73<br>"The Buddha repeatedly emphasized the importance of bringing the mind under control [via samadhi] ... In this way one learns to subdue the wandering mind, which is likened to taming a rutting elephant." – p. 84
Wallace (2011a)	"Simply by relaxing our body and mind, we will find that continuity, coherence, and stability of attention emerge and increase quite naturally and effortlessly." – p. 36<br>In the settling the mind in its natural state practice: "Observe [the] domain [of the mind] and its contents without distraction, diversion, straying to other senses, grasping, or latching on to anything. Don't identify with thoughts, images, or memories. Don't try to control them, silence them, or sustain them. Simply be present with them as an unbiased observer. Let them be." – p. 158 "Directing your awareness does not necessarily imply grasping. Any expression of repulsion, attraction, preference, or control constitutes grasping ... Attending closely to something without grasping is quite feasible." – p. 162<br>"The Tibetan Dzogchen tradition ... maintains that [the substrate consciousness] can be accessed by means of effort and training [in shamatha] ..." – p. 166<br>In the settling of the mind in its natural state practice: "... [T]he mind is settled without being suppressed, manipulated, or contrived; and it naturally dissolves into its ground state." – p. 167<br>In the section headed "Relinquishing Control" (p. 179) Wallace writes: "Shamatha is indispensable in the investigation of the human need for control." – p. 179 "... [T]here is ... an active sense of ego that declares: 'I am'. This reified sense of self often manifests as the need for control." – p. 179 "In the practice of shamatha, especially in mindfulness of the breath and settling the mind in its natural state, we deliberately give up control ... The human tendency is to control whatever we can, especially when we are attending closely. Instead, in this practice we are developing a nonfluctuating flow of clear attentiveness to something that is readily controllable – without deliberately influencing it." – pp. 179-180 "The notion that quiet, rhythmic breathing is preferable can very easily affect the breath, even without conscious control ... All such forms of control over the breath, no matter how well motivated, are to be avoided in [the mindfulness of breathing] practice. The instruction is to release all intentions – just let the breath be." – p. 180 "In the shamatha practice of mindfulness of the breath, we voluntarily relinquish control of the breath ... [O]ur usual sense of being in control is 95 percent unemployed. We could control many things – move about, speak, think various thoughts, and so forth – but we choose not to. In attending to the breath without controlling it, we still control something: we constrain our attention from roving among myriad objects in the other sense fields, anticipating the future, and remembering the past. Control is exercised by focusing the attention on the breath and deliberately releasing thoughts and distractions." – p. 180 "Furthermore, if our faculty of introspection detects that the mind is falling into excitation or laxity, we exert control by taking countermeasures ... The ego's role in this practice is limited to selecting the object of attention, maintaining this selection, and taking countermeasures against excitation and laxity. This is a limited job description because the vast majority of things that we could be doing and controlling have been eliminated." – pp. 180-181.<br>In the section headed "Ego unemployment" (p. 181) Wallace writes: "[In the practice of mindfulness of the breath] [t]o a very large extent, we are practicing egolessness by relinquishing control of everything except the focus and quality of our attention. We are deliberately seeking not to influence the object of our attention." – p. 181 "In the practice of settling the mind in its natural state, the object of attention is also something over which we can exert some control ... When we bring the force of vigilant, clear, unwavering, discerning awareness to the space of the mind and its contents, it is very easy to react by suppressing a thought or diverting the attention. Nevertheless, in this practice we relinquish control over the mind and its

	contents, just as we did with the breath. We are simply present with whatever appears." – p. 182 "Are we directly challenging the delusional sense of self, the autonomous 'I' that's in charge? Not yet. This is still shamatha, which is being developed as a foundation for efficient and effective vipashyana. Are we exerting any control when we attend to the space of the mind? We are exerting control over our attention, just as we do in selectively attending to the breath. In this case, we are selectively attending to mental events from among the six domains of experience. This is an act of will, so our reified sense of self is still being employed. As we settle the mind in its natural state ... if attention starts to slip into laxity or excitation, the remedies are the same ... This necessarily entails some exertion of will and effort, which will probably be done with a sense that 'I am'. The reified self is exerting itself by selecting a domain or an object of mindfulness. We are exerting our will to balance the attention – but nothing more." – p. 182 "Attending closely to the space of the mind and its contents without preference, even though nothing could be easier than generating or inhibiting thoughts, is not easily accomplished. The challenge is even subtler than attending closely to the breath without modifying it, allowing the body to breathe itself with no sense of being in charge. Bring to the mental domain the same quality of awareness, utter nonattachment, nongrasping, and nonpreference, and simply attend to whatever comes up without influencing it in any way. This is a tall order, but it is the quintessence of the practice ... Observe [mental events] with such a loose sense of relaxation that they continue to arise unhindered." – pp. 182-183 "It is a demanding challenge to relinquish control and maintain a spacious awareness regardless of what arises. The ego, the reified sense of 'I am', is quick to jump in and reassert control, starting with preferences: 'This thought needs a little bit of editing, that one's not appropriate, and some thoughts are completely improper – I won't allow those!'." – p. 183 "Whatever appears has no owner or controller; it simply manifests, plays itself out, and dissolves back into the space of the mind." – p. 183 "Our practice is one of egolessness. Without actively probing into the ego to see whether such a reified entity actually exists, the sense of 'I am' has been virtually idled. At the same time, we are developing stability and vividness of attention, along with the enormously important faculty of metacognition ... Relinquishing control is the essence of the practice." – pp. 183-184<br>"... [W]hen you are settling the mind in its natural state and a discursive thought circles up ... simply observe it passively, without trying to affect it in any way ... As you engage in this practice, there is a sequence of thoughts but no active agent – thoughts just happen." – pp. 184-185 "In this practice, it becomes perfectly clear that thoughts just happen, and they do not require an active agent." – p. 185<br>In the settling the mind in its natural state practice: "Remain as inactive as possible, avoiding any intervention in the space of the mind ... To the best of your ability, do not deliberately generate any ... mental activity." – p. 192<br>"The culmination of the path of shamatha is the effortless maintenance of stability and vividness. Even prior to achieving shamatha, in developmental stages eight and nine there is no need to introduce introspection to monitor the meditative practice: this itself is an interruption. An exertion of will is required to think, 'I have to check up'. You don't have to check up anymore. Mental balance is spontaneous and effortless, and the flow of awareness is stable and clear – uninterrupted even by subtle laxity or excitation – making monitoring and intervention unnecessary." – p. 273
Wallace (2010)	In the instructions for the mindfulness of breathing practice: "If you find that your mind has been carried away, see if you can release the effort that is already being exerted in carrying the mind away. " – p. 34 "... [J]ust see if you can respond to mental agitation and distraction ... by releasing the effort that is sustaining the agitation." – p. 35 "... [T]ake the opportunity to release the effort you may be giving to distracted thought or mental wandering." – p. 35 "A problem may arise in that, as soon as you focus on your breathing, it seems that you can't avoid manipulating it with effort or will. That raises a very interesting question: Can we attend to something closely without an almost irrepressible urge to control it? ... This is not just a little problem, but a challenge that really is mainstream practice. The way you can begin to crack the problem is to relax more into the exhalation. You don't have to blow out. You know perfectly well that exhalation will happen all by itself. When you breathe out, savor that. It feels so nice just to be effortless." – p. 35<br>"In [shamatha] practice, once you have established stability within relaxation, then you can apply more concerted effort. This should be a fine-tuned effort ... Aim to sustain somewhat greater continuity, but without the body/mind tightening up ..." – p. 38 Wallace notes that there may also come a point where "it's time to exert more effort and increase the vividness" (p. 39).<br>In his discussion about the need for relaxation as a foundation for stability: "Discipline is valuable, but not if you sacrifice a sense of ease in the practice." – p. 39<br>Discussing mindfulness of breathing: "... [I]f the attention and breath can move like two dancers, without one grabbing the other and pulling it around, there is not much space left for a gross sense of ego." – p. 42

	"The way to move from the first attentional state to the second is by sustaining the relaxation and applying a subtle degree of effort to maintaining the attention. The continuity must not be won at the expense of relaxation." – p. 61<br>"The chief task at the fourth [attentional stage] ... is to get rid of gross laxity ... The remedy is to pay closer attention. You give a little more effort to it, but too much effort will undermine your stability and cause turbulence again. It's a balancing act ... Give it just the right amount of effort to sustain the stability and improve the vividness ... I think we in the West have a different understanding of the word 'effort' than the Tibetan implies. For us effort seems to be such a gross thing, but the intention of this type of effort is to become more and more subtle." – p. 62<br>"... [I]n the ninth attentional state you don't need any effort at all." – p. 65<br>In the discussion about the tenth stage: "You are utterly intent upon the object, but now it becomes effortless ... It is effortless because you are now beyond any need for introspection, beyond the need to apply antidotes for problems ... This effortlessness comes just prior to [shamatha] ..." – p. 66
Wallace and Hodel (2008)	"Note that by [the] sixth [attentional] stage thoughts are so infrequent that our reference points informing us that we are an individual begin to fade." – p. 211<br>"We can categorize contemplative techniques as tending toward either development or discovery ... In shamatha, for the most part, we are using the will, mindfulness, and introspection to develop certain qualities ... [I]n the Great Perfection, release and discovery are the heart of the practice." – pp. 219-220
Wallace (2007b)	"It is common for novices to try too hard, and it has been found that excessive effort agitates the mind ... A common metaphor in the Buddhist tradition is to tune the attention as one would string a lute – not too tight and not too loose – for too much effort results in nervous imbalances, and too little leads to dullness and lethargy ... [E]ffort must be balanced with concentration." – p. 60
Wallace (2005)	Discussing the mindfulness of breathing practice: "Let me emphasize that this is not a concentration technique in the Western sense. We are not bearing down with tight, focused effort." – p. 15<br>Discussing the settling the mind in its natural state practice: "... [O]bserve whatever comes up in the mind with no preference ... Do this without intervention or manipulation, without control." – p. 24 "... [Y]ou are not controlling your mind, as you *were* doing with mindfulness of the breath. Here you are not trying to modify, identify with, or judge the contents of the mind ... When you are not identifying with the objects, not judging, intervening, suppressing, or empowering mental activities, simply observe how the mind settles." – pp. 26-27<br>Having referred to the mindfulness of breathing and settling the mind in its natural state practices, Wallace discusses the awareness of awareness approach: "In this technique there is nothing to cultivate. We are not developing attentional stability and vividness ... The practice ... is one of releasing that which obscures [the] intrinsic stillness and luminosity of awareness. This is a discovery approach rather than a developmental approach, a process of release rather than control." – p. 39 "The technique ... goes beyond ... directly controlling the mind ..." – p. 39 "In this practice, instead of trying to *cultivate* attentional stability and vividness, *release* that which obscures the innate stillness, stability, and vividness of awareness itself." – p. 40<br>"In the practice of meditative quiescence there are two complementary models: development and discovery." – p. 43 Wallace p. 43 notes that stability and vividness are qualities common to both models. "The [mindfulness of breathing and settling the mind in its natural state practices] both follow the developmental approach. While cultivating mindfulness of the breath, we learn to control the wild steed of the mind. We struggle with this unruly mind with the sense of, 'You have dominated me, but now I am going to turn the tables and master you. I am going to learn to ride you so that you can't kick me off or bruise me'." – p. 43<br>On bare attention: "Give your conceptual mind a rest from its normal exertions. Rest your awareness in bare perception, quiet attentiveness." – p. 66
Wallace (1998/2005)	In the section on Tsongkhapa's vision/understanding:<br>"... [W]hen the mind is no longer prone to [excitation and laxity], it is essential to release the effort that had previously gone to counteracting them. At this point, if one continues to exert the same amount of effort as before, this impedes further progress, for it agitates the mind." – p. 187<br>"... [I]t is said that the greatest effort in this training is needed during the initial stages; and as the mind is increasingly habituated to attentional stability and clarity, less and less effort is needed to maintain and enhance these qualities. At the ninth attentional state, no effort at all is required once the meditative process has begun." – p. 194

Wallace (1989/2003)	“The integration of stability and clarity is very challenging, and the tendency among beginning trainees is to try to accomplish that goal by means of sheer determination and effort. Such an approach, however, leads to mental and physical exhaustion, and if one still perseveres, damage to the body and mind results. Efforts must certainly be given to the cultivation of stability and clarity, but it must be skillfully applied. If the effort is too slack, the mind quickly succumbs to unrestrained conceptual wandering or to an increasingly dull stupor. If the effort is too tight, nervous exhaustion and physical discomfort are the result. When one skillfully combines the qualities of stability, clarity, and relaxation, the quality of one’s effort becomes increasingly refined. At the beginning of the training, one encounters gross levels of conceptual agitation and mental laxity, and a relatively gross type of effort is needed to counter this. But as those obstacles become more subtle, the appropriate effort in the practice also becomes refined.” – p. 195
Wallace (2001/2003)	“Buddhism offers many methods of training attentional stability that can be categorized into two basic approaches: control and release. The control approach entails being able to focus and sustain attention on a chosen object at will. The goal of the control model is to become master of one’s mind. A Tibetan metaphor for the untrained mind is an elephant in a rut, rampaging through experience, driven by its own afflictions and causing havoc. In the control model, the out-of-control elephant of the mind is gradually brought to heel. The criterion for success in the control model of training the attention is straightforward. To assess stability of attention, observe whether the chosen object is held in attention or not. To assess vividness of attention, observe whether the object is clear or not.” – p. 80-81 “The second approach to meditative stabilization is the release model. Let’s take the analogy of a polluted river. The previous model of control is like taking active steps to purify the water in the river by filtration and so on. On the other hand, it is known that even if a river is dead due to excessive, prolonged pollution, if one just stops pouring in more pollutants, the purifying elements in the river itself will begin to reassert themselves. Over time, by releasing the river from continued pollution, it purifies itself. The release model when applied to the mind-stream is similar. Instead of applying specific antidotes to all the toxins in the mind, one simply tries to stop polluting one’s mindstream with grasping onto afflictive thoughts and emotions. This can be done quite simply by maintaining one’s awareness without distraction and without mental grasping. In this way, even when mental toxins arise, the mind does not cling to them, and they are swept away effortlessly. In the release model, there is no object upon which to focus the attention. Meditative stability in the release model utilizes awareness itself without reference to any specific object ... The technical term for the release model is ‘settling the mind in its natural state’.” – p. 81 Wallace p. 82 notes that the mindfulness of breathing practice is a “control model [type] of meditation”. “Stabilizing the attention by focusing on the breath is an example of the control model meditation practice. The attention is engaged by holding onto an object, and when that object is lost, the attention must be redirected again, over and over.” – pp. 84-85 “A metaphor for the mind when you first try to stabilize the attention [in mindfulness of breathing practice] is a bucking bronco – climb on and two to three seconds later you are thrown off. Focus again and get thrown off. With persistence, continuity develops. The wild stallion can be trained.” – p. 85 “The main practice of the release model is called ‘settling the mind in its natural state’.” – p. 86 “The quintessence of the release model for training the attention is to let awareness come to rest without distraction and without grasping. ‘Without distraction’ means not being carried away by whatever drifts through the space of the mind. ‘Without grasping’ means not identifying with or mentally grasping onto any of the events or emotions that come along. Let events arise, play themselves out, and vanish without intervention.” – p 87 “The release model is quite different from the control model ... In the control model, mindfulness is like a rope that is tied to an object. In the release model of ‘settling the mind in its natural state’, the rope is released and mindfulness settles into the space of mental events ... [I]n [the mindfulness of breathing] practice, there is still grasping onto an object ... In this practice of ‘settling the mind in its natural state’, one releases grasping of all kinds ...” – p. 87 “Attend fully to the very nature of the mental phenomena without giving any effort to creating, sustaining, or stopping these events. Let them be, arising, playing themselves out, and dissolving of their own accord.” – pp. 88-89 “Both the control and release models develop attention. The control model utilizes grasping; the release model entails letting go from the core of awareness. In the release model, thoughts, ideation, imagery, memories, are not a problem to be controlled or snuffed out. The gushing fountain of thought is not the problem. The problem is grasping. This is a more subtle practice than the control model. Is it really possible to attend to something without latching onto it?” – p. 90 “Not intervening while observing vigilantly is the crux of the [settling the mind in its natural state] practice.” – p. 90
Wallace (2000)	“As a result of diligently counteracting even the most subtle laxity and excitation as soon as they occur, eventually effortless, sustained attention is said to arise due to the power of habituation. At this point, only an initial impulse of will and effort is needed at the beginning of each meditation session; thereafter,

	uninterrupted, sustained attention occurs effortlessly. Now it is actually a hindrance to engage the will or to exert effort. It is time to let the natural balance of the mind maintain itself without interference." – p. 107<br>On the settling the mind in its natural state practice: "... [O]ne releases the mind so that thoughts flow out freely, without suppressing any of them. As long as thoughts are arising, one observes them without interference, and eventually they disappear ... With sustained practice, without ever suppressing ideation, the mind becomes still and conceptual dispersion ceases of its own accord." – p. 111 "This practice ... allows for a kind of free association of ideas, desires, and emotions. Because one is not intentionally suppressing, evaluating, judging, or directing any thoughts and so on that appear to the mind, and because the attention is maintained within the field of mental phenomena, without being distracted by physical objects, many contents of the unconscious are brought into consciousness." – p. 111 "... [W]ith practice ... trains of thought arise, follow their course, and vanish of their own accord. At no point does one have the sense of being the creator, sustainer or destroyer of thoughts. The sense that 'I am thinking that' occurs only when one conceptually grasps onto and identifies with thoughts." – pp. 111-112
Wallace and Wilhelm (1993)	"Doing our best in this training does not mean to try our hardest; because, if we are trying our hardest, we are trying too hard. And if we try too hard, we will burn out ... Doing our best in meditation means being as skillful as we can at finding the delicate balance between relaxation and exertion." – p. 113<br>"During the early phases of practice, considerable degrees of effort are required, but as we progress, more and more subtle effort suffices. Gradually the meditation becomes effortless ..." – p. 118

1.11 The ten stages

See also 1.10 ("Non-doing, effortlessness, relinquishment of control, loss of ego") and 1.12 to 1.14 (practices 1 to 3).

1.11.1 Stage 1: Directed attention

Wallace (2006a)	"The sign of having reached this stage is simply being able to place your mind on your chosen object of meditation for even a second or two." – p. 13<br>"[In the context of mindfulness of breathing] [y]ou have achieved the first stage once you are able to sustain your attention on the breath for even a few seconds." – p. 22
Wallace (2010)	"Accomplishing the first stage means that you can find your object." – p. 59

1.11.2 Stage 2: Continuous attention

Wallace (2006a)	"In the first stage ... the level of excitation is so coarse that you experience virtually no continuity of attention on your chosen object. The mind jumps around from one object to another like a bird flitting from branch to branch, never at rest. Such turbulence is overcome by persistent skillful practice ... Eventually the mind will begin to calm down and you will experience brief periods of sustained attention, but then you lose it again." – pp. 29-30 "When you can occasionally maintain continuity of awareness ... for about a minute, you have reached the second stage." – p. 30<br>Mindfulness of breathing instructions at stage 2: "Without losing [the] sense of [relaxation/ease], now shift your emphasis to the cultivation of attentional stability." – p. 31
Wallace (2010)	"Continual placement means that you are able to attend to your meditative object, free of gross excitation, for about a minute without forgetting it altogether." – p. 60 "The second stage moves towards continuity, although there still can be plenty of peripheral noise. There's probably background chatter in the mind, and your object may not be very clear. It could be extremely fuzzy, but at least you're not losing it." – p. 60<br>"What does the second stage feel like? It feels good. It's not blissful, certainly not continuously, though you may have a little flash of bliss once in a while. But there is a calm soothing quality to it. It's very quietly pleasant and it's not boring any more. You can do it for an hour, even two or three hours, without feeling bored. It's not terrifically high quality, and it's not intensely interesting, but it is just quietly pleasant and that's worth something." – p. 61

1.11.3 Stage 3: Resurgent attention

Wallace (2006a)	"When you reach the third stage ... during each practice session your attention is fixed most of the time upon your meditation object." – p. 43

	"When you reach the third stage, your attentional stability has increased so that most of the time you remain engaged with the object. Occasionally there are still lapses when you completely forget the object, but you quickly recognize them and patch up these holds in the continuity of attention." – p. 43<br>"Long before you achieve this stage, you may occasionally have a session in which your mind seems to remain on the object most of the time. But don't be fooled! Even amateur golfers occasionally hit a birdie, but that doesn't mean they're ready to go on the pro circuit. The third stage is achieved only when your mind remains focused on the object most of the time in virtually all sessions." – p. 43<br>"For most people, the primary problem in this phase of practice is still coarse excitation ..." – pp. 43-44<br>"From the beginning of shamatha training, however, some people are more prone to laxity ... For the moment, we'll concern ourselves only with *coarse laxity* ..." – p. 44<br>"Throughout all the first three stages, involuntary thoughts flow like a cascading waterfall. But over time, these currents of compulsive ideation carry you away less and less frequently. Coarse excitation gradually subsides, even though thoughts and mental images continue to crop up, as do sounds, smells, and other sensory appearances. Don't try to block out these distractions. Simply let them go and refocus your attention as single-pointedly as you can on your chosen object of meditation." – p. 44

1.11.4 Stage 4: Close attention

Wallace (2006a)	"By maintaining continuity of [the] training in a retreat setting, you will eventually achieve the fourth [stage] ... At this point ... you no longer completely forget your chosen object ... You may have experienced glimpses of this level of attentional stability intermittently before actually achieving this stage, but now it has become normal ... You are now free of coarse excitation. It's as if the attention has acquired a kind of gravity such that it can't be easily buffeted by gusts of involuntary thoughts and sensory distractions." – p. 59<br>"While your attention is no longer prone to coarse excitation, it is still flawed by a medium degree of excitation and coarse laxity." – p. 62
Wallace (2010)	"With the accomplishment of the fourth attentional state ... your mind is imbued with a deep sense of calm." – p. 61<br>"The chief task at the fourth state ... is to get rid of gross laxity." – p. 62
Wallace and Hodel (2008)	"In order to move on to the next stage, it is generally considered necessary to shift from a sensory object to a more subtle, mental object of attention." – p. 210 Wallace p. 207 makes clear that this can involve moving from the mindfulness of breathing practice to the settling the mind in its natural state and/or the awareness of awareness practices.
Wallace (1998/2005)	In the section on Tsongkhapa's vision/understanding:<br>"... [At the fourth attentional stage], due to ... sustained attentional stability and relative pacification of ideation, the sense of duality between the meditating awareness and the object of meditation begins to dissolve." – p. 190

1.11.5 Stage 5: Tamed attention

Wallace (2006a)	"Free of coarse excitation, [in seeking to achieve the fifth stage] you must now confront ... coarse laxity." – p. 77 "In the fifth stage, you rise to the challenge of overcoming coarse laxity without destabilizing your attention." – p. 78 "In addition to the persistent problem of medium excitation ... you now have the task of recognizing and counteracting a medium degree of laxity." – p. 78<br>"... [T]he fifth stage is achieved by the power of introspection ... [The faculty/skill of introspection] must now be honed so that you can detect more and more subtle degrees of laxity and excitation." – p. 78<br>In the context of stage 5 settling the mind in its natural state: "If you feel a natural urge to constrict the mind when it becomes agitated, overcome this urge and loosen up. Let the stability of your attention emerge naturally from the mind at ease, rather than a mind that is strenuously constricted. But see that your mind is not so slack that dullness sets in. Here is the challenge, mentioned earlier, that is especially characteristic of the transition from the fourth to the fifth attentional stage." – p. 84
Wallace (2010)	"The main emphasis of the fifth stage ... is to enhance vividness." – p. 63

1.11.6 Stage 6: Pacified attention

Wallace (2006a)	"[At stage 6] [y]ou must still be on guard against the occurrence of medium laxity ... In addition, you are now prone to and need to be able to detect subtle excitation ..." – p. 99<br>"... [I]nvoluntary thoughts pass through your consciousness like a river slowly flowing through a valley." – p. 99
Wallace (2010)	"By the time you accomplish the sixth state, your senses are pretty much withdrawn and you have very little input, if any, from the external environment." – p. 64
Wallace and Hodel (2008)	"Note that by this sixth stage thoughts are so infrequent that our reference points informing us that we are an individual begin to fade." – p. 211
Wallace (1999a)	"[By the time one has completed the sixth stage] an increasing sense of joy and satisfaction arises while meditating ..." – p. 182

1.11.7 Stage 7: Fully pacified attention

Wallace (2006a)	"... [T]he practice itself now fills you with joy. It is this that motivates you to continue ..." – p. 117<br>"Having overcome the medium degree of laxity, subtle laxity remains ..." – p. 117<br>"Subtle excitation also occurs from time to time." – p. 117<br>"At the seventh stage, these subtle attentional imbalances are swiftly recognized due to your finely honed faculty of introspection, and they are easily remedied ... You have become highly adept at balancing and refining your attention, and the rest of the journey to the realization of shamatha is all downhill." – pp. 117-118<br>"Upon reaching the seventh stage of fully pacified attention, the mind has been so refined that your meditation sessions may last for at least two hours with only the slightest interruptions by laxity and excitation." – p. 118
Wallace (2010)	"When you have overcome even the most subtle laxity, you have achieved that seventh attentional state ..." – p. 64 "You still need introspection, because what you have accomplished at this point is not immutable ... Some laxity or some subtle excitation could set in on occasion." – p. 65

1.11.8 Stage 8: Single-pointed attention

Wallace (2006a)	"... [Upon achieving the eighth stage] wherever you direct [your attention], your awareness is coherent and highly focused. From this point onward, you can flow with the momentum of the practice with little or no effort. You can now sustain a high level of *samadhi*, or highly focused attention, free of the imbalances of even the subtlest laxity and excitation for at least three hours or so. Only the slightest degree of effort at the beginning of each session is need[ed] to ward off these obstacles ... For the first time, the flow of attention proceeds without any interruptions by laxity or excitation, and the overall quality of samadhi is one of stillness. Whereas in the preceding stages, involuntary thoughts arose like a river slowly flowing through a valley, now the mind feels calm, like an ocean unmoved by waves." – p. 131
Wallace (2011a)	"Even prior to achieving shamatha, in developmental stages eight and nine there is no need to introduce introspection to monitor the meditative practice: this itself is an interruption. An exertion of will is required to think, 'I have to check up'. You don't have to check up anymore. Mental balance is spontaneous and effortless, and the flow of awareness is stable and clear – uninterrupted even by subtle laxity or excitation – making monitoring and intervention unnecessary." – p. 273
Wallace (2010)	"You are cruising, and you really don't need introspection much at this point." – p. 65 "Your external senses at this time will be shut down; you will not hear anything. You're locked in, and you just continue with that." – p. 65
Wallace and Hodel (2008)	"Here the overall quality of attention is stillness ..." – p. 211

1.11.9 Stage 9: Attentional balance

Wallace (2006a)	"You are now able to maintain flawless samadhi, effortlessly and continuously for at least four hours ... [Y]ou can slip into meditative equipoise, free of even the subtlest traces of laxity and excitation, with no effort at all." – p. 143<br>"The mind has come to a yet deeper state of stillness and serenity ..." – p. 143
Wallace (2010)	"This is more of the same. The only difference is that in the ninth attentional state you don't need any effort at all." – p. 65
Wallace (1998/2005)	In the section on Tsongkhapa's vision/understanding:<br>"At the ninth attentional state, no effort at all is required once the meditative process has begun." – p. 194<br>"All that is needed in the ninth attentional state is an initial effort to begin the meditative process, then it continues effortlessly." – p. 195
Wallace (1999a)	"... [T]he engagement of the will, of effort, and intervention at this point is actually a hindrance. It is time to let the natural balance of the mind maintain itself without interference." – p. 182

1.11.10 Stage 10: Shamatha

Wallace (2006a)	"The nine preceding stages entail many incremental changes, but the actual accomplishment of shamatha involves a radical transition in your body and mind. You will be like a butterfly emerging from its cocoon. This shift is characterized by specific experiences that take place within a discrete, relatively brief period of time." – p. 155 "According to accounts from the Indo-Tibetan tradition of Buddhism, the first sign of the achievement of shamatha is the experience of a sense of heaviness and numbness on the top of the head ... It is said to feel as if a palm were being placed on the top of your shaved head. It's not unpleasant or harmful, just unusual." – p. 155 "This physical sensation on the top of the head is symptomatic of a shift in your nervous system ... that is correlated with gaining freedom from mental *dysfunction* (*daushtulya*), a general state of mental imbalance characterized by stiffness, rigidity, and unwieldiness. Consequently, you achieve a state of mental *pliancy* (*prashrabdhi*), in which your mind is fit and subtle like never before." – p. 155<br>"Following this sense of pressure on the top of your head, you experience the movement of vital energies moving in your body, and when they have coursed everywhere throughout your body, you feel as if you were filled with the power of this dynamic energy. You are now freed of physical dysfunction, so your body feels buoyant and light like never before. Both your body and mind are now imbued with an exceptional degree of pliancy ..." – p. 156 "When physical pliancy initially arises, the vital energies catalyze an extraordinary sense of physical bliss, which then triggers an equally exceptional experience of mental bliss. This rush of physical and mental rapture is transient, which is a good thing, for it so captivates the attention that you can do little else except enjoy it. Gradually it subsides and you are freed from the turbulence caused by this intense joy. Your attention settles down in perfect stability and vividness. You have now achieved shamatha." – p. 156<br>"With access to the first stabilization, you can effortlessly remain in samadhi for at least four hours at a stretch, without the slightest perturbation from either subtle laxity or excitation." – p. 159<br><br>"The Theravada tradition gives this specific description of achieving shamatha by way of mindfulness of the respiration: You begin this practice ... by focusing on the tactile sensations of the breath, which are the 'sign' for preliminary practice (*parikamma-nimitta*). Eventually you shift your attention to the acquired sign (*uggaha-nimitta*) of the breath, which becomes your meditative object until you achieve shamatha, at which point a third sign appears spontaneously. This is called the counterpart sign (*patibhaga-nimitta*) of the breath, which is a subtle, emblematic representation of the whole quality of the air element." – p. 157<br><br>"... [T]he achievement of shamatha by way of mindfulness of breathing is marked by the first appearance of the counterpart sign of the air element. But because the five factors of stabilization ... are not strong in access concentration, you will find it very difficult to sustain your attention on this very subtle mental image. Your mind will slip into the *bhavanga*, or the ground of becoming ... If you wish to proceed beyond access concentration to the actual state of the first stabilization, you steadfastly focus on the counterpart sign until you can maintain your attention on it for a whole day and night. But if you are content with this level of access concentration and wish to use it as a basis for your practice of vipashyana, or contemplative insight, then you release the counterpart sign and rest for a time in the ground of becoming." – pp. 160-161

Wallace (2011b)	"... [T]here is a temporary, transient phase of ecstasy ... Then it tapers off, it becomes subdued, and what lingers ... is a quiet, percolating, radiating sense of serenity, joy that is very malleable." – p. 145
Wallace (2010)	"The actual attainment of [shamatha] is an event, and it will not leave you wondering whether or not it happened: it will come in like the Star Spangled Banner ... – it's that identifiable ... [Y]ou feel a radical shift in the physical body. A rush of unprecedented ecstasy arises in the body and mind. You may experience foretastes of it prior to attaining [shamatha], but it comes in an unprecedented fashion with actual [shamatha] ... It tapers off and the mind settles into a state of very grounded, vivid effortless stability, with an echo of that bliss. The body also acquires an unprecedented quality of buoyancy and pliancy ... [A]t that point, you have attained [shamatha]." – p. 66<br>"[The counterpart sign] becomes your object if you want to go beyond [shamatha] into the states known as the four meditative stabilizations. You attend to that new sign, and there are specific techniques for making the transition into the actual first stabilization and beyond." – p. 84
Wallace (1999a)	"... [E]xperiences of physical bliss and then mental bliss arise, which are temporarily quite overwhelming. But that rapture soon fades, and with their disappearance ... *Samatha* is fully achieved." – p. 182

1.12 Practice 1 – Mindfulness of breathing

1.12.1 Overarching guidance/comments

Wallace (2006a)	"Mindfulness of breathing means settling your awareness on the sensations involved in breathing, continually returning your attention there whenever your mind wanders." – pp. 6-7
Wallace (2005)	"Over the years, I have ... been taught variations of this practice by a number of Tibetan lamas, but I have relied primarily on my Theravada teachers for this kind of meditation." – p. 12
Wallace (2001a)	"Among the many techniques taught in Buddhism for training the attention, the most widely practised method entails cultivating mindfulness of the breathing." – p. 212

1.12.2 Basic instructions, awareness of breathing

Wallace (2006a)	Instructions given at stage 1...<br>"... [Breathe] through your nostrils, noting the sensations of the respiration wherever they arise within your body. Observe the entire course of each in- and out-breath, noting whether it is long or short, deep or shallow, slow or fast." – p. 17<br>"We began this practice at stage one with a primary emphasis on relaxation." – p. 33<br><br>Instructions given at stage 2...<br>"Without losing [the] sense of [relaxation/ease], now shift your emphasis to the cultivation of attentional stability ... With this aim, instead of being mindful of the various sensations of respiration throughout your whole body, focus your attention just on the sensations of the expansion and contraction of your abdomen with each in- and out-breath. As you did before, note the duration of each inhalation and exhalation, and observe the duration of the pauses between breaths." – p. 31<br>"The kind of awareness cultivated here is called *bare attention* ..." – p. 33<br>"Mindfulness of the entire body [as in stage 1] is very helpful for relaxing the mind, but [the] technique of focusing on the abdomen, which is commonly taught in the Burmese Theravada tradition, can be especially helpful for stabilizing the mind." – p. 33<br>"Keep your mind open and relaxed, never constricted." – p. 34<br><br>Instructions given at stage 3...<br>"In this practice session, shift the emphasis to vividness ... Direct your attention to the tactile sensations of your breath at the apertures of your nostrils or above your upper lip ..." – p. 46<br><br>Instructions given at stage 4...<br>"In the phases of mindfulness of breathing thus far, you have been attending in various ways to the tactile sensations of the respiration. However, to continue all the way along the path of shamatha, eventually you must shift your attention from the tactile sensations of breathing to an 'acquired sign' ... a symbol of the air element that appears before the mind's eye as you progress in shamatha practice. To different people,

	acquired signs associated with the breath practice may appear like a star, a cluster of gems or pearls, a wreath of flowers, a puff of smoke, a cobweb, a cloud, a lotus flower, a wheel, or the moon or sun. The various appearances of the acquired sign are related to the mental dispositions of individual meditators. If you wish to continue on the path of mindfulness of breathing – which here explicitly turns into 'mindfulness *with* breathing' – as soon as such a sign arises, shift your attention to this sign. This will be your object of attention as you proceed along the rest of the nine stages leading to shamatha." – p. 64
Wallace (2011a)	"Mindfulness of the breath can be practiced with the eyes in any comfortable position, open or closed." – p. 248
Wallace (2010)	"When [the acquired sign] arises regularly of its own accord whenever you sit down to your meditation, then it's time to shift the focus of your attention. You move your focus from the tactile sensations of the breath and place your attention on the image that has arisen. That ... mental image ... then remains your object up to the time you reach [shamatha]. There is no definite time when that sign will appear, but it may begin to show up occasionally as early as in the third attentional state." – p. 63
Wallace (1989/2003)	"[The acquired] sign may arise when one can remain focused on the breath for roughly an hour with only a few brief conceptual distractions." – p. 196
Wallace (2001/2003)	"In this meditation, your eyes are open and your gaze is cast down resting in the space in front of you." – p. 83
Wallace (2018)	"... [M]indfulness of breathing ... is ... taught in three phases, focusing on the sensations of the respiration throughout the entire body, on the sensations of the rise and fall of the abdomen with each in-breath and out-breath, and on the sensations of the breath at the nostrils." – p. 3

1.12.3 Allowing the breath to flow naturally

Wallace (2006a)	"Don't impose any rhythm on your breathing. Attend closely to the respiration, but without willfully influencing it in any way." – pp. 17-18<br>"... [Y]ou don't try to regulate the breath in any way ..." – p. 19<br>"... [M]indfulness of breathing involves letting the breath flow in and out with as little interference as possible." – p. 20
Wallace (2012)	"We gently surrender all control of the breath ..." – pp. 9-10<br>"... [L]et the breath flow unimpeded, without constraint or effort ..." – p. 11
Wallace (2011a)	"The notion that quiet, rhythmic breathing is preferable can very easily affect the breath, even without conscious control ... All such forms of control over the breath, no matter how well motivated, are to be avoided in [the mindfulness of breathing] practice. The instruction is to release all intentions – just let the breath be." – p. 180
Wallace (2010)	"A problem may arise in that, as soon as you focus on your breathing, it seems that you can't avoid manipulating it with effort or will. That raises a very interesting question: Can we attend to something closely without an almost irrepressible urge to control it? ... This is not just a little problem, but a challenge that really is mainstream practice. The way you can begin to crack the problem is to relax more into the exhalation. You don't have to blow out. You know perfectly well that exhalation will happen all by itself. When you breathe out, savor that. It feels so nice just to be effortless." – p. 35

1.12.4 Dealing with thoughts, distractions and discomfort

Wallace (2006a)	Comments at stage 1...<br>"Thoughts are bound to arise involuntarily, and your attention may also be pulled away by noises and other stimuli from your environment. When you note that you have become distracted, instead of tightening up and forcing your attention back to the breath, simply let go of these thoughts and distractions. Especially with each out-breath, relax your body, release extraneous thoughts, and happily let your attention settle back into the body. When you see that your mind has wandered, don't get upset. Just be happy that you've noticed the distraction, and gently return to the breath." – p. 18<br>"Again and again, counteract the agitation and turbulence of the mind by relaxing more deeply, not by contracting your body or mind. If any tension builds up in your shoulders, face, or eyes, release it. With

	each exhalation, release involuntary thoughts as if they were dry leaves blown away by a soft breeze. Relax deeply through the entire course of the exhalation, and continue to relax as the next breath flows in effortlessly like the tide." – p. 18<br><br>Comments at stage 2...<br>"... [A]s soon as you see that your mind has wandered, release the effort of clinging to the distracting thought or physical sensation, return to the breath, and relax more deeply ... Thoughts are bound to arise. Simply do your best not to be carried away by them." – p. 32<br>"You might be surprised to see how much discomfort arises in your body ... This is normal ... You may view involuntary thoughts and bodily tension as informative signs of underlying mental and physical imbalances and learn from them ... Let these symptoms remind you to relax the mind ..." – p. 35<br><br>Comments at stage 3...<br>"On the periphery of your awareness, you may still note other sensations throughout your body, as well as sounds and so on. Just let them be, without trying to block them out, and focus your attention single-pointedly on the sensations around the apertures of your nostrils." – p. 46
Wallace (2009/2014)	"As you attend to ... your respiration ...[t]ake note of whatever arises to your five physical senses, moment by moment, and let it be. Notice also what goes on in your mind, including thoughts and emotions that arise in response to stimuli from your environment. Each time your attention gets caught up in sensory stimuli or thoughts and memories ... release your mind from these preoccupations, and gently return to your breath. Let your attention remain within the field of sensations of your body, and let the world and the activities of your mind flow around you unimpeded, without trying to control or influence them in any way." – p. 40
Wallace (2010)	"If you find that your mind has been carried away, see if you can release the effort that is already being exerted in carrying the mind away. " – p. 34 "... [J]ust see if you can respond to mental agitation and distraction ... by releasing the effort that is sustaining the agitation." – p. 35 "... [T]ake the opportunity to release the effort you may be giving to distracted thought or mental wandering." – p. 35

1.12.5 Breathing becoming calmer/subtler

Wallace (2006a)	"As your mind calms, you may find that your respiration becomes subtler, and this results in fainter sensations of breathing. The further you progress in this practice, the subtler the breath becomes. At times it may become so subtle that you can't detect it at all. This challenges you to enhance the vividness of attention. In other words, you have to pay closer and closer attention to these sensations in order to stay mentally engaged with the breath." – p. 48<br>Wallace p. 63 makes a similar comment.
Wallace (2009/2014)	"Eventually, the rhythm of the breath becomes calmer and calmer, as your whole body is soothed." – p. 43

1.12.6 Counting breaths

Wallace (2006a) provides the following instructions at stage 2: "[One] way to ... help calm the distracted, wandering mind is to count the breaths. This is like using training wheels when first learning to ride a bicycle. While [this] practice ... involves a kind of thinking, it can help to simplify the conceptual mind. Instead of having many things to think about during the meditation, you reduce your thinking just to counting your breaths." – pp. 30-31. Wallace pp. 31-33 provides specifics of the breath-counting technique. At stage 3, he suggests that: "Since coarse excitation is still the predominant problem during the third stage ... you may find it helpful to continue counting the breaths." – p. 47 Some of the other Wallace texts also provide instructions on breath-counting.

1.12.7 Signs of progress

Wallace (2006a)	"When awareness is brought to rest on a neutral object, such as the breath, immediately every distressing thought disappears, and the mind becomes peaceful, sublime, and happy." – p. 47<br>In the context of stage 4 mindfulness of breathing: "As [conceptual] superimpositions [such as habitual mental images] are released, the sense of your body having definite physical borders fades and you enter deeper and deeper levels of tranquility." – p. 63
Wallace (2009/2014)	"As this practice is developed and cultivated, negative emotions arise far less frequently, and when they do, they tend to subside without disturbing the mind as much as they did in the past." – p. 44

Wallace (2001/2003)	"... [W]hen the attention is focused on the breath, by the simple fact of the mind abiding in a state of clear awareness, disengaged from perceptual and conceptual stimuli that arouse either craving or aversion, a sense of sweetness and joy begins to bubble up and afflictive thoughts disappear ... [T]he mind quickly reasserts its intrinsic equilibrium, joy, and serenity." – p. 83

1.13 Practice 2 – Settling the mind in its natural state

1.13.1 Overarching guidance/comments

Wallace (2006a)	"... [T]he practice of *settling the mind in its natural state* ... directly prepares you for Mahamudra and Dzogchen practice ... A comparable practice within the Theravada tradition is called 'unfastened mindfulness'." – p. 80<br>"You *can* begin your shamatha practice with this method and continue with it all the way to the achievement of shamatha. You don't need to practice mindfulness of breathing first. However, many people find this method difficult, as they are swept away time and again by their thoughts. For them, mindfulness of breathing may be the most effective way to progress along the first four stages ..." – p. 80<br>"The practice ... is designed to ... [let] the mind gradually settle in its ground state. The 'natural state' of the mind, according to Buddhist contemplatives, is characterized by the three qualities of bliss, luminosity and nonconceptuality." – p. 81<br>"... [The] practice is one of discovering, not developing, the innate stillness and vividness of awareness." – p. 87
Wallace (2005)	Wallace p. 22 explains that he first learnt the practice from Geshe Rabten, a Tibetan Buddhist teacher. "Since then, I have received similar teachings from the late Tibetan contemplative Khenpo Jigmé Phuntsok, perhaps the most renowned yogi in all of Tibet, and from Gyatrul Rinpoche ..." – p. 22
Wallace (2001/2003)	"There is nothing natural about our ordinary state of mind. The typical state of the mind is distracted, carried away by one thought after another. In this state, when the mind focuses, it is grasping, identified with thoughts, memories, hopes, fears, and emotions." – pp. 86-87<br>"... the Dzogchen tradition of settling the mind in its natural state." – p. 119
Wallace (2000)	"... [A] method known by the term *settling the mind in its natural state* has been widely taught and practiced especially in the Great Perfection tradition ..." – p. 110
Wallace (1999a)	"... [T]he technique of 'settling the mind in its natural state' has been devised and taught within the Indo-Tibetan Buddhist tradition ..." – p. 184
Wallace (2018)	"... [The practice] entails *observing* the movements of thoughts rather than *identifying* with them ..." – p. 4

1.13.2 Basic instructions

Wallace (2006a)	"The object of mindfulness in the practice ... [is the] *space of the mind* and whatever events arise within that space." – p. 83 "... I would encourage you to remember these quintessential instructions: settle your mind without distraction and without grasping. Practicing 'without distraction' means not allowing your mind to be carried away by thoughts and sense impressions. Be present here and now, and when thoughts arise pertaining to the past or future or ruminations about the present, don't be abducted by them." – p. 83 "Even when your attention is settled in the present, you may still grasp onto the appearances to the mind. Whenever you prefer one mental object over another, whenever you try to control the contents of your mind, and whenever you identify with anything at all, grasping has set in. Here is the challenge at hand: be attentive to everything that comes up in the mind but don't grasp onto anything ... Whatever comes up in the field of awareness, without distraction or grasping, just let it be." – pp. 83-84<br>"In [the settling the mind in its natural state] practice, it is important that your eyes are open, vacantly resting your gaze in the space in front of you." – p. 86<br>"In the shamatha practice of mindfulness of breathing, you let go of thoughts as soon as you detect them and return your attention to the breath. But now, instead of letting thoughts *go*, you let them *be*. Don't prefer one kind of thought to another. Avoid all kinds of attraction to and repulsion from any mental imagery. Don't even prefer the absence of thoughts to the presence of thoughts. They are not the problem. Being distracted by and grasping onto thoughts is the problem." – pp. 91-92

	"During the course of this training, you will experience periods when your mind seems to be empty. Thoughts and mental images seem to have disappeared. This is a time to arouse the vividness of your attention to see if you can detect subtle mental events that have been lurking just beneath the threshold of your awareness ... If, even under the closest scrutiny, you don't detect any appearances in the space of your mental awareness, simply be aware of that empty space. The object of meditation in this practice is both the space of the mind and anything that arises within it. So you can always continue to practice, with or without distinct appearances arising in the mind." – p. 92<br>"... [M]ake sure that you implement the core instructions of this practice: whatever arises in the mind, do not be carried away by it, and do not grasp onto or identify with it. Just let it be. Watch thoughts, feelings, or other mental events arise, with discerning intelligence be aware of their nature, and let them slip back into the space of awareness without any judgment or intervention on your part." – p. 102<br>"In this practice it is crucial to observe the movement of thoughts without intervention." – p. 119<br><br>In the context of stage 7: "At this point in the practice of shamatha, you will have honed your ability to attend to all kinds of mental processes without distraction and without grasping. Even when the mental toxins of craving, anger, and delusion arise, you will be able to observe them without being sucked into them. And insofar as you don't grasp onto them or identify with them, they become detoxified and can no longer disturb the equilibrium of your mind." – p. 123
Wallace (2011b)	"In brief, [the practice of settling the mind in its natural state] consists of observing all arising mental phenomena without grasping on to them. Your thoughts, emotions, images, and so forth are observed closely with mindfulness, but you do not encourage, discourage, or involve yourself in any way with the arising mental phenomena. The aim at this stage is to settle the mind in the substrate consciousness ..." – p. x<br>Wallace quotes Düdjom Lingpa's instructions from the *Vajra Essence*, "Relax and let thoughts be as they are" (p. 116). Wallace says: "*Let thoughts be as they are*. This means you're not intruding on them ... You're not trying to do anything to the thoughts at all." – p. 116 "What is the difference between that approach and just 'spacing out', sitting there with perpetually wandering thoughts? The answer is in [the *Vajra Essence*]: it is to be 'continually observing them'. You allow any and all thoughts to arise – [including] gross thoughts, subtle thoughts, imagery, emotions – everything. Be completely uninhibited, letting thoughts arise in free association ... But rather than just spacing out, letting them spin endlessly, you observe them continuously. Düdjom Lingpa knows perfectly well that you're not going to do this flawlessly, so you just do your best, 'continually observing them with unwavering mindfulness and careful introspection' ..." – p. 116<br>Quoting from the *Vajra Essence*: "... [E]ventually all coarse and subtle thoughts will be calmed ..." – p. 118. Wallace comments: "This happens because you are watching the thoughts, maintaining vigilant mindfulness, without either getting carried away by them or latching on to them. In a word, you are not grasping. You cannot be carried away by thoughts if you are not grasping on to them, nor can you identify with thoughts without grasping on to them ... So, by observing without distraction, without grasping – by just allowing mental processes to arise and pass of their own accord – over time the coarse and subtle thoughts dissipate ... [B]y not engaging with them, thoughts will usually, over time, thin out. They lose their power over you and become fainter and fainter." – pp. 118-119<br>"The practice is really deliciously simple: 'Settle your mind without distraction and without grasping'." – p. 132<br>"[The] practice entails focusing single-pointedly on thoughts and other mental events while ignoring, to the best of your ability, appearances to the five physical senses. When you cannot detect any thoughts occurring, then you focus on the space of the mind, still turning your attention away from the physical senses." – pp. 132-133<br>"If you're letting yourself be carried away, that implies distraction, so do your best to maintain unwavering mindfulness." – p. 134 "On the other hand, there may be times when you are not carried away, not distracted, but you could still be grasping. Something fascinating may arise, perhaps some very interesting, vivid images. 'What's going to happen with that?' you wonder. As soon as the preference is there, as soon as you latch on to it, and moreover, especially if you are attending to the referent of the image, not just the image as an image, that's when grasping becomes locked in. For example, when a mental image of delicious food arises, if you get carried away, thinking about enjoying such a treat, you've been caught up in distraction and grasping. If you focus on the mental image of the food, and you are holding on to it because you're attracted to it, that's grasping. If you are simply mindful of that image, without being drawn to it and without wanting it to go away, you are practising correctly. When you feel attracted to a thought or image or even an emotion, you might tell yourself, 'I'm not distracted; I'm right here, right in the moment. I really

	like this'. That too is grasping. So just be present, letting your awareness remain as still and as accommodating as space." – pp. 134-135<br>"Don't prefer stillness over movement. Whatever arises, let it be, without grasping on to either." – p. 136<br>"... [W]e are luminously aware of whatever comes up – we are not grasping." – p. 145<br>"Because there is no grasping, appearances and awareness are released as soon as they are observed. They are not crystallized into 'things' due to grasping ... They are just arising and vanishing ... Where normally you would grasp and reify, you are seeing only a flux. This doesn't indicate the realization of emptiness, but reification has become relatively dormant." – p. 153
Wallace (2009/2014)	"Now withdraw your attention from your body into the field of your mind, where you experience mental images, thoughts, emotions, desires, memories, and fantasies. You will still experience visual impressions, sounds, and tactile sensations, but focus your interest and attention on the mind alone." – p. 48<br>"When you first do this practice, you may find that your mind suddenly goes blank. Be patient and continue watching. After a while, images are bound to arise ..." – p. 48<br>"Time and again when thoughts arise, you will immediately be swept up by them, and your attention will be carried away to the referents of those thoughts. For example, if a memory of a personal encounter from this morning arises, your attention will be focused on the people and circumstances involved. That's called daydreaming. In this practice, observe the thoughts and images of that memory as events in their own right – occurring here and now in the space of your mind – without letting your attention be carried back into the past. Likewise, when fantasies, worries, or expectations about the future arise, simply be aware of them in the present moment." – p. 49<br>"Whatever events arise in your mind – be they pleasant or unpleasant, gentle or harsh, good or bad, long or short – just let them be. Observe them without distraction and without mentally grasping on to them, pushing them away, identifying with them, or preferring for them to continue or disappear." – p. 50<br>"At times you may begin to feel spaced out and unfocused. When that happens, revive your awareness by refocusing on the practice, training your attention clearly on the space of your mind and its contents ... At other moments, you may find that you have been distracted and carried away by the contents of your mind ... As soon as you note that you are distracted, loosen up your body and mind and release your grasp on the thoughts that have captured your attention. This doesn't mean expelling the thoughts themselves. On the contrary, let them continue of their own accord for as long as they persist. But release the effort of identifying with them." – p. 50<br>"Whatever thoughts and images arise, simply be aware of them, recognizing that they are only appearances to the mind. Observe them without being sucked into them. Passively but vigilantly let them arise from the space of your mind, and let them dissolve back into that space." – p. 51
Wallace (2012/2014)	"In this practice, single-pointed mindfulness is to be sustained to the best of one's ability, without distraction and without grasping. The term 'grasping' refers to any kind of labeling, identification, or emotional reactions of hope, fear, and so on that commonly arise in response to various mental appearances. One common type of grasping has been aptly called 'cognitive fusion' by modern psychologists. This occurs when one's very sense of identity fuses with one's thoughts. Attention is then diverted away from the immediate occurrence of thoughts themselves in the space of the mind and toward the referents of these thoughts. For example, when an image of a loved one's face spontaneously comes to mind, attention is to remain focused on that image itself. But when cognitive fusion occurs, awareness is distracted from the mental image to the actual person, wherever he or she might be." – p. 185<br>"Attention is focused on the domain of the mind." – p. 186<br>"As soon as a mental event, thought, or image arises, it is noted." – p. 186<br>"A central theme of this method of shamatha is to recognize mental events as such rather than conflating them with the objective referents of subjective experiences." – p. 186
Wallace (2011a)	"In the shamatha method called settling the mind in its natural state (Tib. *sems rnal du babs*), the full force of awareness is focused on the mental domain. We attend specifically to the space of the mind and the mental phenomena that arise within that domain, to the exclusion of the five physical senses." – p. 156<br>"Gently open your eyes, at least partially, and rest your gaze vacantly in the space in front of you, without taking any interest in the visual domain. Rest your awareness in the space between yourself and visual appearances. Keep your eyes open but vacant ..." – p. 158 "Now direct the full force of your attention to the domain of mental experience ... while paying no attention to the five physical sense fields." – p. 158<br>"No matter what arises within this mental space ... simply attend to it and observe its nature, remaining alert but nonreactive." – p. 158 "Observe [the domain of the mind] and its contents without distraction, diversion, straying to other senses, grasping, or latching on to anything. Don't identify with thoughts, images, or memories. Don't try to control them, silence them, or sustain them. Simply be present with them

	as an unbiased observer. Let them be." – p. 158 "It is quite natural to be caught up in thoughts and carried away. Using introspection, note this excitation arising as swiftly as you can. In response, relax more deeply, release all grasping on to thoughts, and continue observing the thoughts themselves." – p. 158<br>"The primary instruction for settling the mind in its natural state is to direct the attention to the space of the mind and whatever arises in it, without distraction or grasping. The very essence of the practice is to selectively attend to the space of the mind while ignoring appearances to the five physical senses." – p. 159<br>"Directing your awareness does not necessarily imply grasping. Any expression of repulsion, attraction, preference, or control constitutes grasping ... Attending closely to something without grasping is quite feasible." – p. 162<br>"Opening the Pandora's box of your mind and allowing free associations to flow, you do not care whether acrid fumes are billowing or iridescent butterflies are lofting into your field of consciousness." – p. 183<br>"... [W]e do not deliberately give attention to somatic experiences ... In this practice, we maintain our focus on the mental aspect ... Although a correlated somatic experience might arise in the body, we do not attend to it." – p. 190<br>"Engaging correctly in this practice, we open wide Pandora's box. This is like the free association that might be encouraged in psychiatric analysis ..." – p. 193<br>"It is crucial to attend very closely, intelligently, and discerningly, being utterly present – but without identification, grasping, aversion, suppression, or dissociation." – pp. 195-196<br>"... the practice of observing without distraction, intervention, distortion, preference, or aversion." – p. 198<br>"The shamatha practices of settling the mind in its natural state and awareness of awareness are generally practiced with the eyes at least a bit open ... The eyes can be hooded while letting in some light ... The gaze should rest vacantly in the space in front of you." – p. 248
Wallace (2005)	"... [O]bserve whatever comes up in the mind with no preference ... Do this without intervention or manipulation, without control ... Meanwhile, let your awareness rest, abiding in its own stillness, even while the mind is active. In this way, the mind gradually settles in its *natural s*tate, which is quite distinct from its *habitual* state. Habitually, the mind oscillates between excitation and laxity. But with this practice the mind gradually comes to rest in its ground state, calm and clear." – p. 24 "Finally, in the same fashion, without control or identification, simply rest in a mode of observation, sometimes called 'naked awareness'. Perceive as nakedly as you can whatever arises in this space ... See if your awareness can be so deeply relaxed that you can observe these mental events without intervention." – p. 24 "It *is* appropriate to have a preference for not being carried away by the events of the mind and not grasping onto them. Discover whether it is possible for you to be calm even when the thoughts passing through your mind are disturbed." – p. 25<br>"... [Y]ou are not controlling your mind, as you *were* doing with mindfulness of the breath. Here you are not trying to modify, identify with, or judge the contents of the mind ... When you are not identifying with the objects, not judging, intervening, suppressing, or empowering mental activities, simply observe how the mind settles." – pp. 26-27<br>"The nineteenth-century Tibetan contemplative Lerab Lingpa summarized the essence of this practice with the advice to settle the mind 'without distraction and without grasping'. On the one hand, don't let your attention be abducted by any of the contents of the mind, drifting off to the past or future or into conceptual elaborations about the present. On the other hand, whatever arises in the field of the mind, don't grasp onto it, identify with it, cling to it, or push it away." – p. 27<br>"Grasping often entails either aversion or attraction." – p. 96
Wallace (1998/2005)	In the section on quiescence in Mahamudra/Dzogchen: "... [W]hen ... [Dzogchen] masters speak of grasping, they refer to the mental process of conceptually identifying, or labeling, the objects of the mind." – p. 236
Wallace (2001/2003)	"The main practice of the release model is called 'settling the mind in its natural state'." – p. 86 "The quintessence of the release model for training the attention is to let awareness come to rest without distraction and without grasping. 'Without distraction' means not being carried away by whatever drifts through the space of the mind. 'Without grasping' means not identifying with or mentally grasping onto any of the events or emotions that come along. Let events arise, play themselves out, and vanish without intervention." – p 87<br>"Bring your awareness into the field of the mind and attend closely. Allow the natural limpidity and luminosity of your own awareness to emerge, shining a bright light in the space of your own mind. Let your awareness hover right in the immediacy of the present, without slipping into thoughts concerning the past or speculation about the future. For all manner of mental events that arise ... see if you can let your awareness remain at rest, non-interactive, and non-judgmental, keeping awareness in a state of stillness like empty space. Observe whatever arises. Observe the nature of each of the phenomena ... without grasping onto

	their referents. Attend fully to the very nature of the mental phenomena without giving any effort to creating, sustaining , or stopping these events. Let them be, arising, playing themselves out, and dissolving of their own accord ... The crucial point is to perceive the mental events without grasping or identifying with them ...” – pp. 88-89
Wallace (2000)	“The distinguishing characteristic of this technique is maintaining the attention without distraction and without conceptual grasping, the latter referring to the mental process of conceptually identifying, or labeling, the objects of the mind. Thus, in this practice, one does not grasp on to the intentional objects of thoughts concerning the past, present, or future, nor does one judge or evaluate the thoughts themselves. Now one does not ... try to get rid of thoughts but rather observes them nonconceptually. Without identifying the objects of the mind *as anything*, one tries simply to perceive them in their own nature, without identifying them within any conceptual framework. Thus, without conceptually grasping onto the contents of the mind, one perceptually ascertains their clear and cognitive nature ...” – p. 110 “Whatever kinds of mental imagery occur ... one is to observe their nature, and to avoid any obsessive evaluation of them as being one thing and not another.” – p. 111 “Whenever thoughts arise, one simply observes them without aversion or approval ...” – p. 111 “[O]ne releases the mind so that thoughts flow out freely, without suppressing any of them. As long as thoughts are arising, one observes them without interference, and eventually they disappear ... With sustained practice, without ever suppressing ideation, the mind becomes still and conceptual dispersion ceases of its own accord.” – p. 111 “This practice ... allows for a kind of free association of ideas, desires, and emotions. Because one is not intentionally suppressing, evaluating, judging, or directing any thoughts and so on that appear to the mind, and because the attention is maintained within the field of mental phenomena, without being distracted by physical objects, many contents of the unconscious are brought into consciousness.” – p. 111
Wallace (1999a)	“... [T]his method is exceptional in that the attention is not fixed upon any object.” – p. 184 “... [O]ne brings the attention into the domain of the mind, and whenever any type of mental event is observed – be it a thought, an image, a feeling, a desire, and so on – one simply takes note of it, without conceptually classifying it, and without trying to suppress or sustain it. Letting one’s mind remain at ease, one watches all manner of mental events arise and pass of their own accord, without intervention of any kind. Settling one’s awareness in the present, the attention is not allowed to stray off in thoughts concerning the past or the future, nor does one latch onto any object in the present.” – p. 184 “Normally when thoughts arise, one conceptually engages with the *referents*, or intentional objects, of those thoughts, but in this practice one perceptually attends to the thoughts themselves, without judging or evaluating them.” – p. 184
Wallace (2018)	“Ordinarily when a thought arises, we have the sense of thinking it, and our attention is diverted to the *referent* of the thought. Similarly, when a desire arises, there is a cognitive fusion of awareness and the desire, so awareness is drawn to the object of desire ... In this practice, we do our best to sustain the stillness of our awareness ... Distinguishing between the stillness of awareness and the comings and goings of the mind is the entry into the practice ...” – p. 6

1.13.3 Signs of progress

See also 1.13.4 (“Practice 2 – Settling the mind in its natural state – Stages of progress”) and 1.20 (“Other experiences”).

Wallace (2006a)	“In this practice, the locus of awareness gradually descends from the superficial level of the coarse mental activity that is immediately accessible through introspection down into the inner recesses of the mind that are normally below the threshold of consciousness ... Especially when you engage in this practice for many hours each day, for days, weeks, or months at a time, you dredge the depths of your own psyche ...” – p. 102 “What happens here is a kind of luminously clear, discerning, free association of thoughts, mental images, memories, desires, fantasies, and emotions. You are plumbing the depths of your own mind ... Once-hidden phenomena are unmasked through the lack of suppression of whatever comes up. This is potentially an extraordinarily deep kind of therapy ... [M]ake sure that you implement the core instructions of this practice ... This is the key to letting the knots of the psyche unravel themselves as the extraordinary healing capacity of the mind reveals itself. This is the path to deep sanity.” – p. 102
Wallace (2011b)	Wallace pp. 135-142 lists various signs of progress in this practice, identified in the *Vajra Essence*. For example (quoting from the *Vajra Essence*): “the impression that all your thoughts are wreaking havoc”; “the ecstatic, blissful sense that mental stillness is pleasurable but movement is painful”; “the perception of all phenomena as brilliantly colored particles”; “intolerable pain throughout your body”; and “unbearable misery”. Another example, in Wallace’s words, is that, “You are meditating, the mind becomes still, and then an intense rush of fear comes over you” (p. 136). “There is just so wide a range [of these experiences]

	– an infinite array of them. All of this stuff arises due to the lead weight of your awareness sinking down through the sedimentary layers of your psyche and stirring these things up. Don't grasp on to them; just relax and be present with them, without grasping and without dissociation." – p. 138<br>"[Increasing vividness in the practice] enables you to detect increasingly subtle mental processes that may linger for seconds on end, but so quietly and unobtrusively that they escaped your attention until now. With such vividness of both kinds, mental states and processes that were previously unconscious are now illuminated with the clear light of consciousness." – p. 139<br>"In conducive circumstances [the] practice allows us to catalyze that which lies between our ordinary conscious awareness and the substrate consciousness." – p. 143<br>"Improve your clarity and the subconscious starts to come into view. Continue honing your attention until you probe all the way through the subconscious to the substrate consciousness ..." – p. 150<br>"Over time, and at its own pace, the density of obsessive thoughts diminishes. They become progressively subtler as you simply let them be, let them dissolve, without compulsively being caught up and carried away by them." – p. 170
Wallace (2009/2014)	"As you continue settling the mind in its natural state, gradually the quantity of thoughts and images will subside. On occasion, you may not notice any contents at all. When that happens, closely observe the background of the empty space in which thoughts and images appear. Note whether it is a sheer nothingness or has any characteristics of its own. As you do so, you may begin to detect very subtle mental events that had previously escaped your notice. Because they are so subtle, they slip under the radar of ordinary consciousness. But now as the vividness and acuity of your attention are heightened, you may become aware of mental processes that had previously been locked within your subconscious ... As your mindfulness become[s] more and more continuous, you may detect these microevents for the first time." – pp. 50-51<br>"You have now set out on one of the greatest expeditions as you explore the hidden recesses of your mind. Long-forgotten memories will emerge out of the blue, strange fantasies may haunt you, and the most bizarre thoughts and desires may lurch up and take you by surprise." – p. 51<br>Wallace p. 69 makes similar comments.
Wallace (2012/2014)	"... [S]ubconscious influences are gradually identified via increasingly refined qualitative vividness." – p. 188
Wallace (2011a)	"With the attention focused on the mental domain, less awareness is directed to the other five fields of experience, which eventually grow dark. Continuing along this trajectory to shamatha, the senses and mind dissolve, and only the awareness of the empty vacuity of the substrate consciousness remains." – p. 190<br>"Practicing this for six or more hours per day over an extended period, things that you would never imagine will appear to your mind – they might shock you." – p. 193<br>"In free association, that which was suppressed or subconscious becomes conscious – fascinating discoveries await!" – p. 196<br>"... [T]he trajectory is to gradually withdraw awareness from the five physical senses, attending exclusively to mental events. Thoughts and mental images gradually unravel and dissipate, and the space of the mind becomes more sparsely populated. Eventually the psyche settles and dissolves into the silent, luminous, blissful substrate consciousness." – pp. 248-249

1.13.4 Stages of progress

Wallace (2011b)	"[When well on the way to shamatha] [a]t times ... you may have significant intervals where nothing manifests – no pleasant or unpleasant visions or emotions – allowing you to settle into serenity, into something very close to the substrate consciousness. Things do get very calm after about the sixth of the nine mental stages prior to shamatha." – p. 155<br>"[At an earlier stage than shamatha] [w]hat you could hope for would be to maintain your presence with [thoughts], without distraction, and without grasping ... That was called *enmeshed mindfulness* ... because you are engaged with the phenomena arising in your mind." – p. 158 "Further down the line you move into the interim stages ... where the occurrence of appearances and dissolution is simultaneous ... This occurs sometime during the middle phase of [the settling the mind in its natural state] practice. Later still, the practice becomes more spacious, and there are periods of sheer stainless, radiant clarity. As you progress down the home stretch, there is just naturally settled mindfulness. Without requiring any artifice on your part, mindfulness has settled in its own way, of its own accord ... [Y]ou have arrived at *naturally settled mindfulness*. The anchor has struck bottom." – p. 158 "With an experience that is 'soothing and gentle, with clear, limpid consciousness ...', and that carries 'a remarkable sense of stillness ...' [quoting Düdjom Lingpa in the *Vajra Essence*], if you haven't achieved shamatha yet, you are really close." – pp. 158-159

Wallace (2018)	"Distinguishing between the stillness of awareness and the comings and goings of the mind is the entry into the practice ..." – p. 6 "Continuing in the practice, four types of mindfulness are experienced in sequence. First is *single-pointed mindfulness*, which occurs when you simultaneously experience the stillness of awareness and the movement of the mind. This is like watching images coming and going in a movie and hearing the soundtrack, while never reifying these appearances – that is, taking them to be inherently real things – or getting caught up in the drama." – p. 6 "... [Later] you enter into an effortless flow of the simultaneous awareness of stillness and motion: this second stage is *manifest mindfulness*. Eruptions of memories and desires, and mental afflictions surge up periodically rather than continuously, and over time, your mind gradually settles in its natural state ..." – pp. 6-7 The third type of mindfulness ("*the absence of mindfulness*"; p. 7) and the fourth type ("*self-illuminating mindfulness*"; p. 7) are closely related to the goal-state, and are therefore discussed in Part 2 ("Goal-state") of this table.

1.13.5 Stillness and movement

Wallace (2006a)	"Even when thoughts are on the move, because you are not distracted by them and don't grasp onto them, your awareness remains still. This is called the *fusion of stillness and movement*." – p. 92
Wallace (2011b)	"[In the fusion of motion and stillness] the space of your awareness is still. Why? Because you are not grasping. You can sense your attention being in motion when you are grasping on to objects, pulling you this way and that, with either craving or aversion ... [I]nsofar as you release the grasping, your awareness will remain still, even in the midst of, or simultaneously with, the movements of your mind. This is called the 'fusion of stillness and motion', when your awareness remains still even while your thoughts are in motion." – p. 149
Wallace (2009/2014)	"When you begin to observe thoughts and images, you may find that they disappear as soon as you notice them. Be patient and relax more deeply. Then you will begin to discover a place of stillness within the motion of your mind." – p. 49<br>"The mind is constantly in motion, but in the midst of the movements of thoughts and images there is a still space of awareness in which you can rest in the present moment, without being jerked around through space and time by the contents of your mind. This is the union of stillness and motion." – pp. 49-50
Wallace (2012/2014)	"By resting awareness in stillness even as mental events come and go, mindfulness becomes imbued with a vivid perception of the simultaneity of stillness and movement ..." – p. 186
Wallace (2005)	"... [L]et your awareness rest, abiding in its own stillness, even while the mind is active." – p. 24<br>"Keep in mind that the stability to be cultivated here is not to be found within the objective contents of the mind but in your subjective awareness of them. By this I mean that you settle in the stillness of the space of the mind, even when that space is filled with movement. So this requires a stability of *not* being carried away by thoughts and emotions. Furthermore, because your attention isn't focused on a fixed point, the stability of awareness has a free-flowing quality." – p. 27
Wallace (2001/2003)	"Let your awareness be completely at rest even when your mind is in motion." – p. 89<br>"Discover the stillness of your own awareness in the midst of the activity of your mind." – p. 276

1.14 Practice 3 – Awareness of awareness

1.14.1 Overarching guidance/comments

Wallace (2006a)	"In the practice ... the attention is not directed *to* anything. It rests in its own nature, simply being aware of its own presence. Nominally, you could say that awareness takes itself as its object. But experientially, this practice is more a matter of taking no object. You simply let your awareness rest, without any referent, in its own innate luminosity and cognizance." – p. 132<br>"It is ... possible to be aware of being aware. Imagine the thought experiment of immersing yourself in a sensory deprivation tank that is so effective that you lose all sensory awareness of your body and physical environment. All you are left with is the space of your mind and the thoughts, images, desires, emotions, and so forth arising in that space. Now imagine that this space of awareness is voided of all contents. Even without a sign – an object that you can identify conceptually and to which you can direct your attention – there can still be an awareness of the sheer luminosity and cognizance of being aware. The mind has been reduced to its bare nature, a relative vacuum state. But that emptiness is still illuminated by consciousness.

	And this awareness is innate, still, and vivid. The qualities of attention you have earlier been developing through the practices of shamatha are already implicit in the nature of awareness itself. They have just been waiting to be unveiled." – pp. 132-133 "... [Y]ou can simply be aware of being aware. You don't need to be in a sensory deprivation tank, and your mind doesn't have to be totally silenced ... [W]ith no other object of awareness, you can cultivate attentional stability and vividness of that awareness." – p. 133
Wallace (2009/2014)	Wallace p. 80 refers to the practice as "turning awareness upon itself", "inverting consciousness", and "[cultivating] ... attention directed toward consciousness itself".
Wallace (2012/2014)	"... [A]ttention is withdrawn not only from the physical senses but also from everything that appears to mental awareness. One focuses in the present moment on the immediate experience of the luminosity and cognizance of awareness itself." – p. 66
Wallace (2011a)	"The nature of this practice is to release any interest or focus of attention and simply rest in an ongoing flow of cognizant luminosity." – p. 230<br>"The practice of awareness of awareness entails the observation of awareness itself, without directing awareness to any appearance. You are not interested in any of the five physical sense fields, the space of the mind, or anything arising in the space of the mind – only in awareness itself. When you achieve shamatha on this trajectory, your awareness is of the substrate consciousness ... Your ordinary psyche has dissolved into the substrate consciousness, and all appearances have dissolved into the substrate." – p. 249
Wallace (2005)	"Although I had been introduced to the ... practice by two Gelukpa teachers, Geshe Rabten and Gen Lamrimpa ... it was Gyatrul Rinpoche who first made this subtle practice accessible to me." – p. 35 "... Gyatrul Rinpoche, a senior lama of the Nyingmapa order, who had also received extensive training in the [Kagyupa] order." – p. 35<br>"... [The practice is] the most subtle *shamatha* technique I have ever encountered." – p 38 "It is the simplest, and potentially the deepest and most powerful as well. In this technique there is nothing to cultivate. We are not developing attentional stability and vividness ... The practice ... is one of releasing that which obscures [the] intrinsic stillness and luminosity of awareness. This is a discovery approach rather than a developmental approach, a process of release rather than control." – p. 39 "In this practice, instead of trying to *cultivate* attentional stability and vividness, *release* that which obscures the innate stillness, stability, and vividness of awareness itself." – p. 40
Wallace (2018)	"In this practice one releases grasping to all the subjective impulses of the mind and observes the flow of mental consciousness itself, thereby counteracting the habit of identifying with any aspect of the ordinary mind." – p. 4

1.14.2 Basic instructions

Wallace (2006a)	Wallace quotes Padmasambhava's instructions, as follows...<br>In the chapter on stage 8: "While steadily gazing into the space in front of you ... gently release your mind, and without having anything on which to meditate, gently release both your body and mind into their natural state. Having nothing on which to meditate, and without any modification or adulteration, rest your attention simply without wavering, in its own natural state ... just as it is. Remain in that state of luminosity, and rest your mind so that it is loose and free ... If you become muddled and unmindful, you have slipped into laxity, or dimness. So clear up this problem, arouse your awareness, and shift your gaze. If you become distracted and excited, it is important that you lower your gaze and release your awareness." – pp. 133-134<br>In the chapter on stage 9 (quoting Padmasambhava): "Vacantly direct your eyes into the space in front of you. See that thoughts pertaining to the past, future, and present, as well as wholesome, unwholesome, and ethically neutral thoughts ... are completely cut off. Bring no concepts to mind. Let the mind, like a cloudless sky, be clear, empty, and evenly devoid of grasping; and settle in utter vacuity." – p. 144
Wallace (2009/2014)	Wallace p. 72 notes that in practices such as choiceless awareness and settling the mind in its natural state, "you have focused your attention on some *thing*". "Your attention has been like a laser pointer directed at the screens of your fields of experience, illuminating them. Now retract that laser pointer into itself. Withdraw your attention into its own nature without taking an interest in anything else, not even the space of the mind or its contents. Let your awareness rest in its own space, and simply be aware of being aware. Whatever objects appear to your consciousness, let them be, without trying to shut them out. Just don't take an interest in them. As soon as they tug your attention outward, release them and let your awareness rest in

	its own nature. Whenever a thought arises, release it immediately and let your mind settle in a nonconceptual mode of quiet, still awareness." – p. 72<br>Quoting "eleventh-century Nepalese Buddhist meditation master Maitripa": "Completely dispense with all thoughts pertaining to the past, future, and present, as well as wholesome, unwholesome, and ethically neutral thoughts ... Bring no thoughts to mind. Let the mind, like a cloudless sky, be clear, empty, and evenly free of grasping, and settle your awareness in a state of utter vacuity" – p. 81<br>"[I]n this meditation you immediately cut off thoughts as soon as they arise and let your awareness rest in its own nature." – p. 81
Wallace (2011a)	"Draw your awareness in upon the very experience of awareness itself." – p. 224<br>"The essence of this practice is to take no interest in anything but awareness itself. Visual, auditory, and mental images appear, but you are not interested. As soon as any thought arises, release it immediately without commentary ... [S]imply let it go ..." – p. 226<br>"The shamatha practices of settling the mind in its natural state and awareness of awareness are generally practiced with the eyes at least a bit open ... The eyes can be hooded while letting in some light ... The gaze should rest vacantly in the space in front of you." – p. 248
Wallace (2005)	"... [W]e do not direct our attention to anything. We simply allow awareness to rest in its own place, conscious of itself." – p. 35 "Rest your gaze in the intervening space between the point where you feel you are looking from and the shapes and colors that appear in your field of vision. Rest your gaze in the space itself, not on any visual object." – p. 36 "Settle your awareness in the very state of being aware ... In this practice, unlike the preceding two, you are not focusing on any object, mental or physical." – p. 36 "Simply let awareness settle in its own nature. Thoughts may come and go, but they do not obscure the nature of awareness; they are expressions of it. Rest in that state of awareness without being carried away or distracted by any contents of awareness, and without grasping onto anything at all." – pp. 36-37<br>"Awareness is equally present during and between thoughts ..." – p. 38 "Although you are not specifically attending to the contents of the mind, you are not unaware of them either." – p. 38 "It is crucial ... to keep the eyes open." – p. 38<br>"... [T]here is no focus on any object of meditation." – p. 40
Wallace and Wilhelm (1993)	"Do not cling to ... thoughts, identify with them, or try to sustain them. But also do not try to suppress them. Simply view them as spontaneous outflows of natural awareness, while centering your attention on the pure, unelaborated awareness from which they arise." – p. 116

1.14.3 Pairing with breath

On occasion, Wallace pairs the awareness of awareness practice with the breath. For example, Wallace (2011a) instructs: "With each inhalation invert your awareness upon this luminous cognizance, taking no interest in any appearances to the mind, thoughts, imagery, or sensory perceptions. With each exhalation, relax from the core, utterly releasing your awareness without latching on to any object or mental appearance ... Whatever thoughts come up, release them immediately ... and rest in the ongoing silent flow of awareness of awareness." – pp. 224-225 Wallace explains: "The classic commentaries mention the oscillating motion of inversion and release of the attention as a preliminary exercise. Conjoining this oscillation with the breath is something I have taught for a number of years as an introductory technique. Although you are doing something to control awareness, this form of grasping is a benign one." – p. 226 Wallace advises that, when more advanced: "Finally, center your awareness where it always was ... without inversion or release. Rest in utter simplicity, sustaining the flow of sheer luminosity and cognizance of awareness ... [T]his practice will take you all the way to shamatha." (p. 226). At pp. 239 to 240 Wallace provides similar instructions.

1.14.4 Awareness as an object

See also 2.14.24 ("No longer mindful of meditation object").

Wallace (2006a)	"With the instructions for the eighth attentional stage onward, we move on to the still subtler practice of maintaining awareness of awareness itself. The technique is called *shamatha without an object*. Here the practice is not so much one of *developing* attentional stability and vividness as it is of *discovering* the stillness and luminosity inherent in awareness itself." – p. 7<br>"In the practice ... the attention is not directed *to* anything. It rests in its own nature, simply being aware of its own presence. Nominally, you could say that awareness takes itself as its object. But experientially, this practice is more a matter of taking no object. You simply let your awareness rest, without any referent, in its own innate luminosity and cognizance." – p. 132

Wallace (2009/2014)	Wallace p. 87 describes how the breath can be used to support the awareness of awareness practice. He then says: "As your mind calms, your breathing will become more and more subtle, and when this happens, disengage your awareness from the breath and rest in the ongoing flow of awareness of awareness. Now direct your attention downward, gently release your mind, and without anything on which to meditate, rest both your body and mind in their natural state. Having nothing on which to meditate ... rest your awareness without wavering, in its own natural state ... just as it is. Remain in this luminous state ..." – pp. 87-88
Wallace (2011a)	In his instructions for the awareness of awareness practice at pp. 224-225, he says: "With each inhalation invert your awareness upon this luminous cognizance, taking no interest in any appearances to the mind, thoughts, imagery, or sensory perceptions." (p. 224); "With each exhalation, relax from the core, utterly releasing your awareness without latching on to any object or mental appearance." (p. 225).
Wallace (2010)	"Another possible object for [shamatha] meditation is the mind itself, as taught in the [Mahamudra] and Dzogchen traditions. Some people find it discouraging, because the object can be very elusive and yet if one can do it, it can be very, very rewarding." – p. 78<br>"The problem is that it's so easy just to space out ... When you practice [shamatha] with the mind itself as your object, it's very easy to just sit there with a blank mind. Sitting with a blank mind is not the same thing as doing [shamatha] on the mind, which has an object, but it's an extremely subtle one." – p. 80
Wallace and Hodel (2008)	"At [the seventh attentional stage] we may also practice *quiescence without a sign*, which means maintaining stability without any object whatsoever." – p. 211
Wallace (2005)	"... [W]e do not direct our attention to anything. We simply allow awareness to rest in its own place, conscious of itself." – p. 35 "In this practice, unlike the preceding two, you are not focusing on any object, mental or physical." – p. 36 "... [T]here is no focus on any object of meditation." – p. 40<br>"Rest in this utter simplicity – aware of being aware, without an object." – p. 189 "When resting in awareness, can you see that there is no object that is awareness? It is not a real entity. It has no inherent identity of its own. It is as empty and open as space itself." – p. 189<br>"You simply let your awareness settle in its own space, without reference to any object. There is now no subject/object relationship ..." – p. 191

1.14.5 Signs of progress

See also 1.20 ("Other experiences").

Wallace (2009/2014)	"... [I]nvoluntary thoughts gradually subside and the mind settles in its natural state." – p. 82<br>"By engaging in the [awareness of awareness practice], you may bring previously unconscious memories, fantasies, and emotions of all kinds into the light of awareness." – p. 91 "As we consciously expose the deep space of the mind through thousands of hours of observation, we penetrate into normally hidden dimensions that are more chaotic, levels where the order and structure of the human psyche are just beginning to emerge. Strata upon strata of mental processes previously concealed within the subconscious manifest, until finally the mind comes to rest in its natural state ... This is an exercise in true depth psychology, in which we observe 'core samples' of the subconscious mind, cutting across many layers of accumulated conceptual structuring." – p. 91
Wallace and Wilhelm (1993)	"... [I]t may not be long before we experience short periods – perhaps up to ten seconds or longer – during which we are able to abide in a natural state of awareness, without grasping onto the thoughts and other events that arise in our consciousness. We may well find this delightfully exhilarating, and our minds may then leap upon the experience with glee. But as soon as our minds grasp in this way, the experience will fade. This can be frustrating." – p. 115 "The remedy is to enter into this state of awareness repeatedly. As we become familiar with it, we can then take it in stride, without expectation or anxiety. We learn to just let it be." – p. 115 "During early stages of practice, we may experience moments of mental quiescence relatively free of conceptualization, and we may wonder whether we are now ascertaining natural awareness. Most likely we are not. Our mind at this point is probably still too gross and unclear for such a realization ... When we ascertain the simple clarity and knowing qualities of the awareness, we are well established in the practice." – p. 117

1.14.6 Awareness of the preceding moment

See also 2.14.3 (“Consciousness/awareness itself”).

Wallace (2012/2014)	“Another form of retrospective awareness is awareness of awareness itself, in which one ascertains the immediately preceding moment of awareness.” – p. 66
Wallace (2011a)	“According to the Buddhist understanding, a single moment of awareness cannot take itself as an object because that moment would have to be subdivided. Therefore the shamatha practice of awareness of awareness is a retrospective observation.” – p. 225 “Even so, one can be aware of a moment of awareness a mere tenth of a second ago ... The experiential sense is one of dwelling in a flow of awareness of awareness. It feels like real-time awareness of a moment of awareness because each moment is so similar to the preceding moment that it doesn’t matter. For all practical purposes, you are aware of awareness in a working memory that approaches real-time observation.” – pp. 225-226<br>“Recall that a primary definition of mindfulness is the all-important faculty of memory. Just as a recording device captures images or sounds for later review, mindfulness captures experiences and recalls them later.” – p. 268

1.15 Possible pitfalls with more subtle shamatha techniques

Wallace (1998/2005)	In the section on quiescence in Mahamudra/Dzogchen:<br>Wallace p. 242 discusses how there are possible pitfalls with more subtle shamatha techniques. The techniques he refers to are an “attending to thoughts” practice, which is similar to or the same as the settling the mind in its natural state method, and a “focusing on non-conceptuality” practice, which appears to be more similar to the awareness of awareness technique. He contrasts these with a less subtle practice, Tsongkhapa’s technique of focusing on a mental image. “[In the practice of attending to thoughts] [a]s one has no fixed object on which to focus the attention, one may easily succumb to mere day-dreaming, drifting from one thought to another, without attentional stability or clarity. Instead of leading to the actual achievement of quiescence, such pseudo-meditation results merely in mental lassitude ... [T]he method of focusing on non-conceptuality as one’s object of quiescence, if not followed properly, may result in blank-mindedness, in which the mind apprehends nothing and is devoid of clarity. Although there may be some degree of attentional stability in this trance-like state, Tsongkhapa cautions that, rather than leading to the achievement of quiescence, such sustained practice actually impairs one’s intelligence.” – p. 242 “Using a visualized mental image ... as Tsongkhapa suggests, provides the meditator with a clearly defined object on which to fix attention ... Thus, the danger of slipping into idle day-dreaming or trance-like mental vacuity is decreased.” – p. 242

1.16 Other references to flawed meditation

Wallace (2010)	“If, in the course of a session, your sense of ease is sustained, and ... continuity is really quite good, you may find yourself beginning to sink into the object. This is a premature phasing out of duality, merging with the object in a way that is not useful, like slipping down into mud. At that point, it’s time to exert more effort and increase the vividness.” – p. 39
Wallace (1998/2005)	In the section comparing Indo-Tibetan and Theravadin presentations of quiescence:<br>“In the context of quiescence, [Mahamudra/Dzogchen scholar] Karma Chagmé characterizes ‘flawed meditation’ as a state of consciousness comparable to deep sleep, in which the physical senses are dormant and mental awareness is unclear. If one persists in that semi-comatose state, he warns, one will be reborn as an animal. This description closely parallels Tsongkhapa’s account of the state of subtle laxity, in which the mind is excessively withdrawn and the full force of attentional clarity is absent. Tsongkhapa claims that by dwelling in such a state, which can easily be confused with meditative equipoise, in the near term one’s intelligence is impaired, and in the long term one is reborn as an animal. Subtle laxity, he insists, must be completely eliminated before quiescence is accomplished.” – p. 266
Wallace (2000)	“... [W]hile one focuses the attention on an unchanging object, there is the possibility of dementia setting in if one allows the potency of attentional vividness to wane. The result of such faulty practice is that one enters a kind of trance, or mental stupor, in which one’s intelligence degenerates. The way to avert this danger is by taking on the difficult challenge of enhancing one’s attentional vividness without sacrificing attentional stability.” – p. 105

	"When the power of mindfulness has fully emerged, the attention no longer strays from its object. At that time, if one does not continue striving to enhance the power of attentional vividness, one may fall into a complacent, pseudo-meditative trance, which may result in dementia." – pp. 106-107
Wallace and Wilhelm (1993)	Discussing the awareness of awareness practice: "... [I]t is important to recognize that this practice is not based upon a vague sort of trance or dull absorption; rather, it calls for vivid, clear awareness." – p. 115
Wallace (1999a)	Wallace p. 183 describes a shamatha practice where, "the object of mindfulness is the mere absence of ideation". This appears to be the awareness of awareness practice, or a practice that is similar. "Tsongkhapa especially emphasizes that while following this method, one must *ascertain* the absence of ideation as one's meditative object, rather than simply letting one's mind go blank. His concern here, I presume, is to ensure that the meditator does not mentally drift into a nebulous trance, but maintains an actively engaged intelligence throughout this training." – p. 183

1.17 Non-duality

Wallace (2006a)	In the context of stage 5: "[The settling the mind in its natural state] practice challenges the existence of an absolutely objective space of the physical senses that is absolutely separate from a subjective space of the mind. You are now on a path to realizing the meaning of nonduality." – p. 87 "... [A]s the deeply ingrained habit of conceptual grasping subsides, you may begin to experience physical objects in a different way ... As the conceptual mind calms down, you will see more clearly what the Buddha meant when he said, 'In the seen there is only the seen' ... Sensory objects take on a quality of transparency, as mere appearances to the mind, rather than solid objects 'out there'. Even your own body appears 'empty' of substance. All that appears to the mind is an interrelated matrix of sensory phenomena, but these qualities no longer appear to belong to something absolutely objective, for that sense of reified duality is diminishing." – p 90
Wallace (2011a)	"The conceptual mind is quiet, so there is no sense of separation between awareness and its object." – p. 208
Wallace (2010)	"When introspection is no longer needed because the problems for which it was designed are no longer present, at that point the reified sense of subject-object dichotomy begins to break down. You are left with just the experience, the event of mindfulness taking place with continuity and with vividness. It's from that space that you move right into the actual accomplishment of [shamatha]. That is an advanced state, but you will almost certainly experience facsimiles of that state prior to achieving it. You get glimpses, or brief tastes, when you know for yourself that, for a while at least, there is no longer a sense of the meditator. The dichotomy between the meditator and meditative object is something that has to be constructed ... We construct it by conceptualizing it concretely ... Insofar as you release this ongoing commentary, you also begin to release the more quiet construct of 'I am meditating'. And you release it by simply attending more closely and with tighter continuity, moving the dominoes closer together, until there is no more space to also say, 'And, oh yes, I am meditating'." – pp. 49-50
Wallace (1998/2005)	In the section on Tsongkhapa's vision/understanding:<br>"... [At the fourth attentional stage], due to ... sustained attentional stability and relative pacification of ideation, the sense of duality between the meditating awareness and the object of meditation begins to dissolve." – p. 190

1.18 Non-conceptuality

Wallace (2006a)	In the context of stage 5: "Another remarkable experience that may arise from [the settling the mind in its natural state] practice is a nonconceptual sense that nothing can harm the mind regardless of whether or not thoughts have ceased. This implies that even when concepts are present, your awareness may remain nonconceptual." – p. 90 "... [I]n this practice, whatever thoughts or mental images arise, you simply observe them, without distraction and without grasping. You nonconceptually note them as mental events in the present, without attending to their referents and without being either attracted to them or repulsed by them. You just let them be. In this way you maintain a nonconceptual awareness of concepts." – p. 91
Wallace (2009/2014)	On the awareness of awareness practice: "... [L]et your mind settle in a nonconceptual mode of quiet, still awareness." – p. 72

1.19 Glimpses of substrate consciousness

Wallace (2011b)	Wallace quotes Düdjom Lingpa in the *Vajra Essence*: "Some especially experience bliss, luminosity, or nonconceptuality, and they settle on this." – p. 123 Wallace comments: "They latch right on to that. This can happen well before you achieve shamatha." – p. 123 "I know someone for whom bliss arises immediately when he sits down to meditate. What do you suppose he does? He really likes bliss, and since it comes to him so easily, he's on it like a hummingbird on a hummingbird feeder. He is grasping on to joy. Unfortunately, when you are doing that, you are not doing the practice. For some people it is luminosity that comes easily. For others it's nonconceptuality. In any of these cases if you grasp, you are not doing the practice." – p. 123
Wallace (2011a)	"... [L]ong before the actual achievement of shamatha, [bliss] represents a manifestation of the substrate consciousness in your immediate experience. Even though such experiences are transient and anomalous, they are indications of the substrate consciousness. Continuing on the path, you are likely to have more such experiences. As you approach the mother lode, resting in the substrate consciousness is utterly luminous, blissful, and nonconceptual." – p. 243
Wallace (2005)	Wallace says that when people experience features of substrate consciousness: "Some people call that *nirvana*, others call it *ultimate reality*, *pure consciousness*, or *pristine awareness*. But most likely they are gaining a glimpse of the substrate consciousness ..." – p. 89
Wallace (1989/2003)	"As one proceeds in [the mindfulness of breathing] training, the mind and the breath are gradually calmed ... The calmed mind that is not afflicted by [craving or aversion] experiences a relative emotional equilibrium, and from this arises an unprecedented state of mental and physical suppleness and well-being. In the early stages of the practice these qualities arise only intermittently, but as one progresses suppleness and joy are experienced with increasing frequency, intensity, and duration." – p. 196

1.20 Other experiences

See also 1.13.3 ("Practice 2 – Settling the mind in its natural state – Signs of progress").

Wallace (2006a)	"Various physical sensations may occur in meditation." – p. 48 Wallace gives examples, including the body feeling large, tingling, vibration and heat. He says that these sensations arise from "shifting and releasing pockets of tension" (p. 48). "Don't be worried about them or make a big deal of them; these are natural consequences of the practice." – p. 48<br><br>In the context of stage 6 settling the mind in its natural state...<br>"Throughout the development of shamatha, even at this relatively advanced stage, a myriad of emotions and other mental and physical conditions may arise, many of them very unexpectedly. This practice of settling the mind in its natural state is especially known for unveiling the suppressed and repressed contents of the mind ..." – p. 100 "One of the more common challenges in this practice is the emergence of fear. As you release your grip on the contents of the mind, you are undermining your normal sense of personal identity, which is constantly reinforced by thinking, by recalling and identifying with your personal history, hopes and plans. Now you are disengaging from these familiar supports for substantiating your ego. As lapses between thoughts occur more and more frequently and for longer periods, your awareness hovers in a kind of empty space, a vacuum devoid of personhood. You may come into the grip of fear as your normal sense of who you are loses its footing ... You have nothing to lose but your false sense of an independent, controlling ego. The only thing being threatened is an illusion. If you don't identify with it, there's nothing to fear ... [I]t is of the upmost importance to observe such fear without distraction and without grasping." – p. 100<br>"Another emotional balance that may crop up at any time throughout this training is depression ... When any of these emotions or attitudes arise during meditation sessions, treat them like any other mental event: watch their emergence, see how they linger, then observe them disappear back into the space of the mind ... Rather than identifying with them, or owning them, let them emerge from the space of awareness and dissolve back without any intervention on your part ..." – p. 101<br>"When you plumb through the strata of the psyche by such practices as settling the mind in its natural state, you may encounter phenomena that appear as demons. It is crucial to understand that demons can appear to your mind as a result of *correct* practice, let alone misguided practice. If you are Tibetan, the demons you encounter may have multiple heads and arms. If you are a Westerner, your demons may arise in forms more widely accepted within our society." – p. 104 Wallace gives the examples of "lust, spiritual arrogance, fear,

	boredom, self-doubt, guilt, and low self-esteem" (p. 103). "As you dredge the depths of your psyche, your own demons will emerge into the light of your consciousness. You can count on it." – p. 104 "Let the demons of the mind reveal themselves and instead of clinging to them or grappling with them, allow them to vanish of their own accord ... This is the practice of allowing the mind to heal itself." – p. 104<br>"... [A] wide array of meditative experiences (Tibetan: *nyam*) ... may occur on the path of shamatha." – p. 105 "... [T]here is no consistency in the specific experiences from one individual to the next." – p. 105<br>Wallace pp. 105-107 provides examples of these experiences. "While many of us would likely respond to some of those disagreeable experiences by stopping the practice or seeking medical help, Düdjom Lingpa actually called them all 'signs of progress'! ... The deeper you venture into the inner wilderness of the mind, the more you encounter all kinds of unexpected and, at times, deeply troubling memories and impulses that manifest both psychologically and physically. At times, these may become so disturbing that psychological counseling or medical treatment may be necessary ..." – p. 107 "It should come as some consolation that the difficulties encountered in the practice of settling the mind in its natural state are finite. Eventually you will emerge through the layers of the psyche into a clear and luminous space of awareness." – p. 109
Wallace (2011b)	Wallace pp. 123-134 provides other examples of nyam.<br>"Nyam comprises a variety of types of anomalous, transient experiences that are catalyzed by authentic meditative practice. The experiences may be pleasant, unpleasant, frightening, euphoric, or ecstatic. They may be interesting or boring. They may be exotic or fascinating or terrifying, psychological or somatic. They are aroused by meditation, in particular by [the] practice mode of free association – settling the mind in its natural state." – p. 124 "Using free association to dredge the depths of your personal samsara can bring up a lot of interesting things that can manifest in some very bizarre and sometimes exceptionally unpleasant physical symptoms. Again, they are just nyam." – p. 129<br>"... [I]n the course of meditative practice if you start experiencing dread, anxiety, insomnia, or paranoia – be sensible. Maybe you are meditating in an imbalanced way; maybe you are dredging up something that is throwing you out of kilter." – p. 131<br>"[Düdjom Lingpa in the *Vajra Essence*] has ... given us the simple antidote [to dealing with nyam]" (p. 164): "just let them be" (p. 165). "The variation among the psyches, substrate consciousness, and karma of individuals is immensely complex. It is impossible to tell someone exactly what is going to happen between the initiation of shamatha meditation and its achievement." – p. 165<br>Wallace p. 166 refers to the different levels of sedimentation in the psyche that shamatha practice can reveal. He says, "You will ... move through the nyam, the meditative experiences of this new stratum. Even the clear, serene strata are also nyam" (p. 166).
Wallace (2011a)	"As you engage in meditative practices ... various mental and physical experiences can manifest vividly as signs of progress on the path. Such meditative experiences (Tib. *nyams*) are defined as anomalous, transient experiences resulting from authentic meditative practice ... The overriding instruction concerning all such experiences, regardless of their content, is to simply be present with them while avoiding any form of grasping, attachment, or aversion." – p. 242<br>"The more deeply you dredge the psyche in intensive practice, the more stuff that will come up, including traumatic memories, deep-seated fears, and powerful emotions." – p. 242 "Dredging the psyche in the practice of shamatha can catalyze transient experiences of paranoia or disturbing feelings in the body." – p. 243
Wallace (2010)	"...[Y]ou may hit a layer of sheer dread where everything that could possibly be a source of danger in your life rises up with a malevolence that seems completely inevitable. You feel you are about to be crushed, and if one thing doesn't demolish you, then another one will ... It is psychically dark and heavy." – p. 168

1.21 Posture, dealing with discomfort

Wallace (2006a)	"It is generally preferable to practice meditation sitting on a cushion with your legs crossed. But if that is uncomfortable, you may either sit on a chair or lie down in the supine position (on your back) ... Whatever position your assume, let your back be straight, and settle your body with a sense of relaxation and ease. Your eyes may be closed, hooded (partially closed), or open, as you wish." – p. 16<br>"If you tend toward excitation, you may find lying down especially helpful for releasing the tightness and restlessness of your body and mind. But if you are more prone to laxity, you may simply fall asleep whenever you lie down, so it may be necessary for you to be upright when meditating." – pp. 19-20

	"Sitting upright with legs crossed is generally the most suitable posture ... But Lerab Lingpa also advised that you be comfortable when meditating. So if sitting cross-legged for extended periods is painful for you, try sitting in a chair or lying in the supine position." – p. 85
Wallace (2009/2014)	In the context of the settling the mind in its natural state practice: "Begin by 'settling your body in its natural state', so that it is imbued with three qualities. The first quality is a physical sense of relaxation, ease, and comfort, which should persist throughout this entire ... session. The meditative practice is challenging enough, so it's important that you don't put yourself through any undue physical discomfort. Second, let your body be as still as possible, avoiding any unnecessary movement, such as fidgeting and scratching. Move only if your legs or back start to ache. Third, assume a posture of vigilance. If you are sitting upright, keep your back straight and slightly raise your sternum ... If you are lying on your back, straighten your body, with your arms stretched out about thirty degrees from your torso. Let your eyes be partly open, but let your gaze be vacant." – p. 47
Wallace (2011a)	"Many people are uncomfortable sitting on the floor. Our backs ache, our knees hurt, and we can't sit still without fidgeting or cutting off the circulation in our legs. Without being comfortable, the essential goal of relaxation cannot be approached." – p. 29 Wallace p. 29 notes how one option to achieve a comfortable posture is to use a "*zabuton*, a Japanese-style sitting cushion". "... [E]xperiment to find a comfortable but solid support for your lower spine ... Experiment with chairs." – p. 30<br>"Establish a comfortable seat, in a sitting or supine posture. Arrange firm support from cushions to minimize discomfort." – p. 32 "I encourage you to explore two primary postures. The first is seated on a chair or in a cross-legged position on the floor, whichever is most comfortable for you. Second explore the very useful option of meditating while lying down: the supine position." – p. 32<br>"I'm a strong advocate of the supine posture in addition to seated postures." – p. 35<br>"Deep stabilization of the mind in shamatha practice is best done in the more grounded postures: seated and supine." – p. 35<br>"You can always shift your posture; an upright position promotes vividness and counteracts laxity, while a supine position promotes relaxation and counteracts excitation." – p. 245
Wallace (2010)	"The Tibetan tradition ... places little or no value on physical pain in the meditation. They say: If it hurts, move ... Of course, you can go overboard with this approach if you start to fidget at the slightest discomfort and scratch every little itch that comes up. Your awareness just decomposes. I would suggest a middle way, but the middle way that I teach and practice tends to be quite gentle. If you find something that is really poking into your consciousness and nagging at you, then I suggest you move ... But don't move at the earliest sign of discomfort ..." – pp. 43-44
Wallace (2005)	"Generally, the optimal and most widely recommended posture is sitting cross-legged on a cushion. If this is too uncomfortable, you may sit in a chair ... But another, less commonly used posture is lying down on the back ... This is especially useful if you have a back problem or if you are physically tired or ill." – p. 12<br>"Whatever posture you adopt, let your body rest at ease, with your spine straight but not rigid." – p. 13<br>"Throughout each meditation session, let your body be as still as possible, with a minimum of fidgeting." – p. 13 "Even if you are lying down, let your posture reflect a sense of vigilance, not just collapsing into drowsiness. If you are sitting up ... [s]it at attention, without slouching forward or tilting to either side." – pp. 13-14<br>"For [the settling the mind in its natural state] practice your head may be slightly inclined." – p. 23
Wallace and Wilhelm (1993)	"It is important not to become slouched forward or to tilt to the side or backward." – p. 113

1.22 Duration of meditation sessions

Wallace (2006a)	"At the beginning of [the] training, meditators are traditionally encouraged to practice for sessions of twenty-four minutes ..." – p. 9<br>"[By the third stage] you will have increased the duration of each session beyond the initial twenty-four minutes to perhaps twice that." – p. 43<br>Guidance at stage 3: "As you advance in the practice, increase the duration of each meditation session and decrease the number of sessions each day. Always go for quality over quantity." – p. 49<br>"[At the fourth stage] [e]ach of your sessions may now last an hour or longer ..." – p. 59

	Guidance at stage 4: "As you progress ... increase the duration of your sessions for as long as you are able to maintain a quality of attention relatively free of laxity and excitation." – p. 64
Wallace (2009/2014)	On the awareness of awareness practice: "As you become more and more familiar with this practice, you may gradually increase the duration of your sessions, but don't let them last so long that the quality of your meditation decreases." – p. 73
Wallace (2011a)	"Maintain short sessions. Finish while you're still fresh and wouldn't mind continuing. Slowly increase the length and frequency of sessions, but never past the point of freshness." – p. 32<br>"A session of twenty-four minutes is a good starting interval ..." – p. 33
Wallace and Wilhelm (1993)	"The chief criterion for determining the length of one's meditation sessions is the quality of one's awareness during the practice. Five minutes of finely conducted meditation is worth more than an hour of low-grade conceptual chatter. Another useful criterion is one's state of mind following meditation. The mind should be refreshed, stable, and clear. If one feels exhausted and dull, one's session was probably too long or of low quality." – p. 115

1.23 Time/effort taken to progress, and factors supporting progress

1.23.1 General comments

Wallace (2006a)	"Most people would find their lives greatly enhanced just by attaining stage two of the ten stages. This level of development takes some effort, but it can be achieved by people who are living a busy life with career and family commitments as long as they are willing to set aside some time for meditation. It can dramatically improve the quality of everything you do and make you more resilient in the face of emotional and physical stressors." – p. 8<br>"For most people, achieving stage three will require a greater commitment than an hour or two spent each day in meditation in the midst of an active life. The more advanced stages ... are accessible to people who dedicate themselves to weeks or months of rigorous practice in a conducive environment. Progress beyond the fourth attentional stage requires a vocational commitment to this training, which may involve full-time practice for months or years at a stretch." – p. 8<br>"According to Tibetan oral tradition, among meditators who are well qualified to embark on this discipline, those of sharpest faculties may be able to achieve all ten stages within three months; those with 'medium' faculties may take six months; and those with 'dull' faculties may require nine months. Such estimates assume that the meditators are living in a contemplative environment and devoting themselves day and night to this discipline. The reference to sharp, medium, and dull faculties pertains to the level of talent and attentional balance individuals bring to this training." – p. 9<br>"[The] training usually does not provide swift gratification or immediate results. It can lead to exceptional mental health and well-being, but this takes time and effort." – p. 39<br>"Many people appear to achieve the second stage while meditating just one or two sessions each day." – p. 45 "If you are practicing for only a session or two each day, you may not progress beyond the second attentional stage. The reason for this is simple: if you are balancing your attention for an hour or so each day, but letting it become fragmented and distracted for the other fifteen hours of waking time each day, then the attentional coherence cultivated during these brief sessions is overwhelmed by the distractions of the rest of the day. The achievement of the [third stage] requires a greater commitment to practice. This will entail multiple sessions of meditation each day, practiced within a quiet, contemplative way of life that supports the cultivation of inner calm and collectedness." – p. 45<br>"... [I]f you are intent on progressing through all the nine stages, the time-tested way to proceed is to radically simplify your life, withdraw temporarily into solitude, and devote yourself full-time to this practice for extended periods. It is not easy to achieve the bliss of shamatha without leaving heavily populated areas, which tend to be noisy and congested. In contrast, in the wilderness, removed from society, a meditator can more easily set his mind at ease and accomplish meditative stabilization." – p. 49<br>"The major challenge at [the third] stage of the practice is to adopt a lifestyle that supports the cultivation of attentional balance, rather than eroding it between sessions. To achieve stage three, the dedicated meditator will need to take up this practice as a serious avocation, spending days or weeks in this practice in the midst of a contemplative way of life in a serene, quiet environment. If we practice only a session or two each day while leading an active life, we may occasionally feel that we've reached the sustained attention of the third stage, but we'll have a hard time stabilizing at that level. The busy-ness of the day intrudes, the mind becomes scattered, and the attentional coherence gained during meditation will likely be lost." – pp. 49-50

	"While you are devoting yourself to shamatha training, it is important to keep other activities to a minimum, for if your behavior between meditation sessions erodes the coherence of attention that you gained during sessions, then you won't be able to gain any ground." – p. 53<br>"Psychologists have found that the time generally needed to acquire expertise in a variety of high-level skills is five to ten thousand hours of training in a discipline of eight hours each day for fifty weeks in the year. This is roughly the degree of commitment required to progress along the entire path to the achievement of shamatha. Between formal meditation sessions, it is vital to maintain a high degree of mindfulness and introspection throughout the day." – pp. 67-68<br>"... [A]fter some thousands of hours of rigorous training, you will reach the sixth attentional stage ..." – p. 99<br>"To reach [stage nine] will almost certainly require many months, or even a few years, of continuous, full-time practice ... [T]he higher stages of shamatha practice will not be achieved by engaging in many brief retreats of weeks or a few months at a time. It requires long, continuous practice without interruption. There are no shortcuts." – p. 143<br>"... [S]ome Buddhists dismiss [shamatha practice] on the grounds that ... by itself [it] does not liberate the mind. Some Mahayana Buddhists marginalize it on the grounds that it is common to Theravada Buddhism, which they regard as an 'inferior vehicle' of spiritual practice. And there are Tibetan Buddhists who similarly overlook it in favor of more esoteric tantric practices." – p. 148<br>"How long does it take to achieve shamatha if one is well prepared and practices diligently and continuously in a conducive environment, with good companions and under the skillful guidance of an experienced mentor? ... [T]he Tibetan oral tradition states that under such optimal conditions, a person of 'sharp faculties' may achieve shamatha in three months, one of 'medium faculties' in six months, and a person of 'dull faculties' may achieve it in nine months. This may well be true for monks and nuns who begin their shamatha practice after years of study and training in ethics. But in the modern world, this appears to be an overly optimistic forecast. Consider that five thousand hours of training, at a rate of fifty hours each week for fifty weeks of the year, is the amount of time commonly required to achieve expertise in a high-level skill. To reach an exceptionally high level of mastery, ten thousand hours may be required." – p. 162<br>"Given the many profound psychological, societal, and environmental differences between people living in industrialized nations and in traditional societies such as rural Tibet, it is impossible to predict with accuracy how long it may take people living in our modern world to achieve shamatha. But there are some encouraging signs that significant progress can be made. At the end of the one-year shamatha retreat led by Gen Lamrimpa in 1988, one meditator sat four sessions each day, each one lasting three hours. Another sat for just two sessions, each more than seven hours long. Neither one, according to Gen Lamrimpa, had achieved shamatha at that point, but both had made very good progress." – pp. 162-163<br>"The contemporary Burmese meditation master Pa-Auk Tawya Sayadaw has concluded with considerable authority that achievement of even the first meditative stabilization is very, very rare in today's world." – p. 165
Wallace (2011b)	"Shamatha requires more careful incubation than most other kinds of meditation ... [For example] [v]ipashyana you can cultivate anywhere ... If you wish to take shamatha all the way to its ground, however, it requires a supportive, serene environment ... and very few preoccupations. The necessary internal conditions are minimal desires, few activities and concerns, contentment, pure ethical discipline, and freedom from obsessive, compulsive thinking. It is my feeling that the achievement of shamatha is so rare today because those circumstances are so rare." – pp. ix-x<br>"... [I]t is possible, if you are a person with very sharp faculties, to achieve shamatha in the first seven days, or in the second seven, or in the third. If so, you can move right on to the next stage, vipashyana." – p. 112<br>"How many [people] have achieved shamatha? It's difficult to say, but since relatively few people are practicing shamatha as described here, it seems likely that relatively few people are achieving the results described here." – pp. 121-122 In this context, Wallace p. 121 notes that people are using various other types of meditation, such as bodhichitta, vipashyana, Vajrayana, Mahamudra, Dzogchen, vipassana and Zen meditation. "People do a little bit of shamatha, are satisfied with that, then think, 'Now let's get on with it – vipashyana. Let's do the important stuff: bodhichitta, vipashyana, stage of generation, stage of completion, and Dzogchen' ... I really don't think that shamatha is unbelievably hard. I think that most people are simply impatient, eager to get on with other things ..." – p. 122
Wallace (2009/2014)	"... [A] senior Tibetan meditation master named Yangthang Rinpoche, renowned among his Tibetan Buddhist peers for profound meditative realizations" said to me (quoting) "it's an open question whether people can still achieve shamatha nowadays" (p. 114). "As Yangthang Rinpoche commented, it is an open question whether people in the modern world can [achieve shamatha] ..." – p. 115

Wallace (2012/2014)	"In short, there appear to be few people in the modern world who authentically teach and practice shamatha and far fewer who have actually attained it." – p. 148<br>"... [T]oday, as Buddhism is being assimilated into our materialistic, fast-paced, multitasking modern world, [the shamatha practices] are commonly misunderstood, marginalized, and dismissed." – p. 148<br>"To achieve [shamatha] may require a year or two of intensive training, meditating ten hours per day." – p. 152<br>"... [S]hamatha has been widely neglected in recent times." – p. 154<br>"Given the vital importance of shamatha in all schools of Buddhism, we must face the question directly: Why is its accomplishment so rare? ... [I]f aspiring contemplatives in the modern world are to achieve shamatha, they must be guided by qualified instructors, provided with an environment conducive to sustained training, and supported financially while they commit themselves to practice ... [T]he prerequisites for achieving shamatha are rare. Naturally, its achievement must also be rare." – pp. 154-155 Wallace p. 155 discusses how the prerequisites for achieving shamatha are highly demanding. Outer conditions include that: "... [O]ne must be able to practice continuously – until shamatha is achieved – in a quiet, healthy, pleasant environment where one's material needs are easily met." – p. 155 "The inner requirements are even far more exacting." – p. 155 They include that: "Until the achievement of shamatha, one must devote oneself to a simple lifestyle, with as few extraneous activities as possible, including socializing, conducting business, and seeking entertainment." (p. 155); and "... [B]oth during and between formal meditation sessions, one must overcome the deeply ingrained habit of letting one's mind be caught up in involuntary thoughts and ruminations" (p. 155).<br>"In order to achieve a greater degree of mental balance and well-being, it can be very helpful to practice shamatha for as little as thirty minutes each day in the midst of an active, socially engaged way of life; nevertheless, one cannot expect to proceed very far toward the first dhyana by this alone. The optimal way to achieve shamatha is to enter retreat and practice continuously and single-pointedly for ten to twelve hours per day until one actually attains this sublime state of meditative equilibrium." – p. 156 "Such complete withdrawal into solitude may not be necessary for everyone. Someone who is truly dedicated to achieving shamatha may formally meditate for as little as six hours each day, even engage with others between sessions, and still progress in the practice. Especially in such circumstances, the quality of environment and companions is vital." – p. 156<br>"One must ensure that all the necessary prerequisites are fulfilled; otherwise, disappointment is inevitable." – p. 156<br>"The widespread marginalization of shamatha may also be due in part to the fact that the necessary prerequisites are almost nowhere to be found in today's world." – p. 156<br>"It is very challenging and sometimes stressful to devote oneself to full-time, solitary practice for many hours each day, for weeks or months on end, without entertainment or other distractions." – p. 185
Wallace (2012)	"The achievement of shamatha is a rarity these days, even among dedicated contemplatives. There are two major reasons for this: In the rush to enter into and complete the 'higher' practices and realizations, shamatha – even though it has been considered a requirement for such achievements – has been de-emphasized in many contemporary contemplative traditions. Second, unless one already has an extremely relaxed and balanced mind, full achievement of shamatha may require many months or even a couple of years of concentrated solitary practice. At one time, in calm, pastoral societies such as Tibet's, well balanced minds were more common, making the achievement of shamatha possible in a shorter time span. But in today's speedy and agitated global civilization, such minds are exceedingly rare." – p. 4
Wallace (2011a)	"The practice of mindfulness is deceptively simple – but not easy to do properly." – p. 61<br>In a discussion about the settling the mind in its natural state practice: "Contemplative science is not a simple practice for anyone ..." – p. 160<br>On the settling the mind in its natural state practice: "As you spend several thousand hours practicing shamatha, for up to twelve hours a day, everything gradually settles like the flakes in a snow globe ... [Y]our mind dissolves into the substrate consciousness ..." – pp. 165-166<br>"If you were to practice settling the mind in its natural state for ten hours a day over a six-month period, then you would likely find that your senses were gradually withdrawing." – p. 248
Wallace (2010)	"Is achieving [shamatha] really possible? It may not be feasible for everyone, but it is generally within reach. The experience of people who did a one-year retreat in 1988 led by the Tibetan contemplative Gen Lamrimpa was very inspiring. It gives me a high degree of confidence that if we approach it very traditionally, if we attend closely to the ... prerequisites and the environment, that we have just as much chance of accomplishing [shamatha] now in the modern West as they had in Tibet five hundred years ago, or in India twenty-five hundred years ago." – p. 66

	"If you really want to achieve [shamatha], there is a time-tested prescription: radically simplify your life for a period and practice in such a way that your whole life is focused on [shamatha] meditation. It's been done with success many times and people know it works. There is another approach that is not so well proven, but could be very interesting. A Tibetan lama recently said that, in principle, it's possible to attain [shamatha] even while leading an active life ... If you could engage in action with calm and with the presence of mind to simply do what needs to be done, it is possible in principle to achieve [shamatha] in an active way of life ... As an approach, it's more risky: it has not been proven very often ... [A]ccomplishing [shamatha] in an active life would be headline news, an important breakthrough for Dharma in the modern world. Accomplishing [shamatha] even by traditional methods, in solitude, would be fantastic." – pp. 67-68<br>"If one is well prepared, has attended to the necessary prerequisites, and applies oneself to the practice full time, with intelligence and skill, in an environment that is conducive for this practice, then one may attain [shamatha] in about six months. Generally speaking, if you really want to achieve [shamatha], then it's best to radically simplify your life, take out a section of time, and just do [shamatha]. They say that if you have really sharp faculties you can achieve [shamatha] in three months. Or, if you are well prepared but less capable, it might take as long as a year. Those are ballpark figures, of course. In the same vein, [Atisa] mentions that if you have not attended very closely to the prerequisites, but just go off into retreat and try to do it with sheer determination then you can meditate for a thousand years and not accomplish it." – p. 81<br>"*Question*: Do you know many people here in the West who have achieved [shamatha]? *Response*: No, I suspect it is very rare. But let's look at the reasons why ... [I]f you don't have a suitable environment, it's going to be really tough if not simply impossible." – p. 82 "Secondly, it's rare to find a qualified teacher in that suitable environment. To do it entirely on your own with a book would be extremely tough. Another reason it's rare is that hardly anybody tries." – p. 82 Wallace pp. 82-83 discusses how Tibetan, South East Asian, and Zen meditators all tend to focus on other practices. "So in the great three branches of the Buddhist tradition, hardly anybody does [shamatha] now ... Now it's very, very rare, but the Dalai Lama is encouraging monks to start tackling it again." – p. 83 "I don't feel comfortable saying that it's not possible any more simply because so few people are doing it. If you did find a proper environment and a suitable teacher, and got your prerequisites in shape, then maybe it wouldn't be rare at all. The experiment has yet to be done ... If people really attend to the tradition, which draws on an immense wealth of experience, I think it's very feasible." – p. 83
Wallace and Hodel (2008)	"How long does it take to achieve shamatha? The Tibetan oral tradition says this depends on the talent of the student as well as on having the right conditions ... Those with 'sharp faculties' may achieve shamatha in three months, those with 'medium faculties' in six months, and a person of 'dull faculties' may take nine months. However, there is a significant difference between the context of Tibetan hermits and monastics living in the isolated reaches and relative tranquility of Tibet prior to the Chinese occupation, and those of us in the modern West. For us I would agree with modern psychologists, who calculate that five thousand hours of concentrated training – eight hours each day for fifty weeks in the year, for a total of nearly two years – are generally required for the accomplishment of many high-level skills. This gives a rough idea of the level of commitment necessary for attaining shamatha." – pp. 207-208
Wallace (2007b)	"Expertise in this mode of observation may require as much has 5,000 to 10,000 hours of training, 8 to 12 hours a day, 7 days a week, for months on end. In addition to this formal practice ... the practitioner must take all necessary steps in terms of lifestyle and emotional regulation to ensure mental health throughout the course of this extremely demanding discipline." – pp. 42-43<br>Wallace p. 44 refers to the "relentless, passive but vigilant observation of whatever arises in the space of the mind, without being carried away by or identifying with it" as "an extraordinarily demanding endeavour".
Wallace (2005)	"If you are intent on elevating your attention to exceptionally high degrees of stability and clarity ... the general counsel from the Buddhist tradition is to radically simplify your life. Withdraw into solitude where you can devote yourself to contemplative practice with as few distractions as possible." – p. 32 Wallace explains that "most of us are unable to do this" (p. 32), and provides some recommendations for practising shamatha as part of an active life (pp. 32-33). One of them is: "... [M]ake sure people don't disrupt your mind during the daytime and that the nights are peaceful." – p. 33
Wallace (1998/2005)	"In Tibetan Buddhist practice, a person seeking to achieve quiescence normally focuses on this training, largely to the exclusion of other activities, until it is brought to its culmination." – p. 93<br>"Continuity of practice is particularly important for the cultivation of quiescence, for if the mind is allowed to indulge freely in distractions between sessions, or if there are long lapses between periods of concerted practice, whatever mental balance that may have been achieved will swiftly deteriorate. For this reason, one is encouraged to simplify one's lifestyle radically during this training ... However, there have been cases of

	individuals achieving quiescence even while maintaining an active lifestyle, presumably by maintaining a high degree of mindfulness and introspection between sessions." – p. 197<br>"Since 1970, I have spent many years in Tibetan and [Theravada] Buddhist centers in Europe and North America. Although training in quiescence is encouraged in a minority of these centers, for the most part it receives little or no emphasis; and I have yet to hear of a single Western Buddhist who has accomplished quiescence as it is presented here." – p. 219 "Modern [Theravada] Buddhist contemplatives tend to overlook the training in quiescence in favor of insight practice alone ..." – p. 220<br>"One factor contributing to the rarity of the achievement of quiescence seems to be that such practice is very demanding and the desired results often do not arise as swiftly or as predictably as one might hope. Boredom, frustration, and hypertension may then easily set in, and the temptation to proceed on to more advanced, interesting, and hopefully fruitful contemplative methods may turn out to be overwhelming." – p. 220 "... [W]hen the Buddhist tradition informs its followers that the most transformative meditations are those that are more advanced ... those seeking liberation and enlightenment may easily follow the urge to pass over this training ... Moreover, the scarcity of contemplatives who have accomplished quiescence means there are few who can teach this training from personal experience from start to finish. Therefore, those who aspire to achieve this goal may be unable to acquire the tacit knowledge that can be acquired only from an accomplished teacher ... [T]hey may well question their own ability to achieve a contemplative state that their own teachers have not reached. This is all the more the case for Western aspirants, who have been brought up in a culture that denies the value, and even the possibility, of achieving such sustained voluntary attention." – p. 221
Wallace (1989/2003)	"[The] refinement of awareness [that occurs in shamatha practice] ... entails a major transformation of consciousness, and this is not easily brought about. In general it can be achieved only by months of continuous mental training usually pursued in solitude, free from the distractions of ordinary daily life." – p. 150<br>"Such training requires very demanding preparation and sustained, undistracted commitment to meditative discipline, and it is fraught with physical and psychological perils. These are some of the reasons why this training is not frequently given nowadays, especially in the West ..." – p. 194
Wallace (2001/2003)	"Achieving quiescence does not take the same amount of time for everyone. Tibetans describe a superior, middling, and inferior ability for quiescence. A person of superior abilities, after becoming well-grounded in the preliminaries, might require three months of intensive effort under skillful guidance. For people of middling abilities, six months is average. For people of inferior abilities, about one year of intensive effort is necessary." – p. 93<br>"... I have never heard any [Western Buddhists] report that they have accomplished quiescence as it is described in authoritative Indian Buddhist treatises on this practice." – p. 97
Wallace and Wilhelm (1993)	"It is not enough to learn a technique for stabilizing the mind and apply oneself to it with diligence. If [the underlying [conditions/prerequisites] are not satisfied, meditative quiescence will never arise ..." – p. 108
Wallace (2018)	"In my experience over the past forty-five years, it is very difficult to find a genuinely conducive environment in which one can continue practicing for as long as it takes to achieve [shamatha]. So I have concluded that it is necessary to create facilities that are specifically designed for such long-term practice. Since very few people with intense dedication to practice are financially independent, it is also necessary to provide financial support for them ..." – p. 194

1.23.2 Comments on Tibetan Buddhist practitioners specifically

Wallace (2006a)	"... [I]t is all the more perplexing that among Tibetan Buddhist meditators today, both inside and outside Tibet, very few devote themselves to sustained shamatha practice." – p. xii "Given the widespread consensus concerning the vital role of shamatha in Buddhist contemplative practice, one might expect that it would be widely practiced and that many people would accomplish it. Oddly enough, there has long been a strong tendency among Tibetan Buddhist contemplatives to marginalize shamatha in favor of more advanced practices. Tsongkhapa commented on this oversight in the fifteenth century, when he said, 'There seem to be very few who achieve even shamatha', and Düdjom Lingpa commented four centuries later, 'Among unrefined people in this degenerate era, very few appear to achieve more than fleeting stability'." – p. 147 "During numerous conversations with seasoned recluses and with His Holiness the Dalai Lama, I have tried to discover whether this statement holds true nowadays. The consensus is that the actual achievement of shamatha today among Tibetan Buddhist contemplatives, both in Tibet and living in exile, is not unknown, but is exceptionally rare." – p. 147

Wallace (2012/2014)	"... I made my first journey to Tibet, to see whether there were still adepts there who had achieved shamatha; I discovered that such people did exist, but they were rare." – p. 148
Wallace (2010)	"Another reason [attaining shamatha is] rare is that hardly anybody tries. The Tibetan meditators I know are practicing *tummo* (psychic heat meditation), or Dzogchen, or [Mahamudra] or Lamrim meditations, but hardly any of them do [shamatha]. It's bizarre, but it's true." – pp. 82-83
Wallace (1998/2005)	"Tsongkhapa commented in the early fifteenth century that there seemed to be very few people who achieved quiescence as it has been taught in the great treatises. In 1980, I began field research to try to discover whether this statement holds true nowadays. During the spring and summer of 1980 ... I enjoyed numerous conversations with a number of seasoned recluses and with His Holiness the Dalia Lama ... The consensus among them was that the achievement of genuine quiescence today among Tibetan Buddhism contemplatives living in exile is not unknown, but is exceptionally rare. In Tibet during the decades prior to the Chinese Communist occupation, there seem to have been considerably more cases ..." – pp. 218-219<br>"During the summer of 1992, I conducted a cursory survey of experienced Tibetan Buddhist contemplatives living ... in the Tibet Autonomous Region and in the areas of western China largely inhabited by Tibetans. Although I learned of hundreds of Tibetan men and women devoting their lives to full-time contemplative practice – as is the case among the Tibetans living in exile – those who have accomplished quiescence would seem to be very rare at best ..." – p. 219
Wallace (2001a)	"Traditionally, Buddhists ... are advised to disengage temporarily from a socially active way of life. Withdrawing for a period of weeks, months, or even years, into solitude, they radically simplify their lifestyle and devote themselves single-pointedly to training the attention, while remaining as free as possible from all distracting influences." – p. 211

1.24 Participant-based academic studies of the subjective experience

Kozasa et al. (2017) (co-authored by Wallace) includes an assessment of participants' stage of progress in their Shamatha practice. "Participants ... took part in a 9-day Shamatha meditation retreat ..." – p. 1237 "All participants practiced meditation for 4 h [per day] ..." – p. 1237 The training included the mindfulness of breathing, settling the mind in its natural state, and awareness of awareness Shamatha practices (p. 1237). "When the retreat finished, the participants were asked to compare their stage of Shamatha before and after the event. In order to answer this question, they were requested to check a summary of the stages, taught during the retreat. These stages, from one to nine, were described by Kamalashila in his classical work Stages of Meditation. The stages ... [culminate] in a state of stability and vividness which can be sustained for hours (stage 9) ..." – p. 1238 The authors do not provide the content of the "summary of the stages". For "less practice" (LP) participants (i.e., participants with "1 year of meditation practice or less"; p. 1237) ($N = 14$), the mean pre-retreat stage was 1.29 ($SD = 0.61$), and the mean post-retreat stage was 2.79 ($SD = 0.98$) (p. 1238). For "more practice" (MP) participants (i.e., participants with "more than 1 year of meditation practice"; p. 1237) ($N = 21$), the mean pre-retreat stage was 2.29 ($SD = 1.23$) and the mean post-retreat stage was 3.95 ($SD = 1.71$) (p. 1238). "When comparing scores before and after the retreat, we found a significant improvement in both [the LP and MP] groups... in the stages of Shamatha ..." – p. 1239 "There were group differences ... in the [stage of Shamatha]." – p. 1239 "It should be noted that only 35 of the 44 participants answered the question about the stages of Shamatha (some, in LP and MP groups, did not answer this question; for example, some of those in the LP group were not sure about their level of Shamatha and others considered it too complex; in the MP group there was some uncertainty about their level of Shamatha)." – p. 1239 The authors p. 1240 note various limitations of the study, including the small sample.

Part 2: Goal-state

2.1 Terminology

2.1.1 Terms used to describe features of the goal-state
See also 2.14.44 ("Difficulty communicating the experience to those who have not had it").

Wallace (2006a)	"While the terms 'joy' and 'luminosity' are familiar in common English usage, and 'nonconceptuality is the absence of something with which we are all familiar, what the terms actually mean at this advanced stage of meditation cannot be imagined unless you have reached that stage yourself. This is where language can be misleading. Words used to describe such states of consciousness must give some idea to nonmeditators and novices of what takes place when high degrees of attentional balance are achieved. But it is a great mistake to assume that just because one has a layperson's knowledge of the meaning of these terms one has understood what they mean in these rarified contexts." – pp. 109-110

2.1.2 The term shamatha

Wallace uses the term *shamatha* in at least two ways. Those two are closely related. The first is to describe the practice. The second is to describe the goal-state, substrate consciousness. As examples of this second usage, Wallace (2011b) refers to "... shamatha, or quiescence – a state of bliss, luminosity, and nonconceptuality" (p. 112), and Wallace (2012/2014) states that, "The quality of awareness that results from the practice of samadhi is known as shamatha, in which the mind is calmly, continuously focused inward and both body and mind are imbued with exceptional degrees of pliancy and well-being" (pp. 150-151).

2.1.3 The term quiescence

Wallace (2006a)	"... [S]hamatha ... literally means quiescence, tranquility, and serenity." – p. 77
Wallace (2011b)	"... shamatha, or quiescence – a state of bliss, luminosity, and nonconceptuality." – p. 112
Wallace (2009/2014)	"... *shamatha* (meditative quiescence) ..." – p. 85
Wallace (2012)	In the glossary: "meditative quiescence. (Sanskrit: *shamatha*) ..." – p. 161
Wallace and Wilhelm (1993)	"In Buddhism the result of this practice is called *meditative quiescence*, or *tranquility*." – p. 105

2.1.4 The term nature of the mind

The term nature of the mind can refer to the goal-state or to pristine awareness, depending on the context. As examples of the former usage: Wallace (2011b, p. 117) refers to a passage where Düdjom Lingpa uses the term "essential nature of the mind", and Wallace says, "Here 'essential nature' refers to conventional nature; we are not speaking of the ultimate nature"; Wallace (2012/2014, p. 151) refers to "the essential nature of the mind – the subtle, luminous continuum of mental consciousness from which all ordinary states of waking and dream consciousness emerge"; Wallace (2012/2014, p. 193) says, "There are many practices in Tibetan Buddhism that are said to result in the realization of the 'nature of mind'". He then explains that these include Shamatha practice, and that they culminate in realizing pristine awareness; and Wallace (2011c, p. 176) states that, "[In achieving shamatha] [y]ou have come to the culmination of this practice, and you realize the nature of the mind, the *conventional* nature of the mind, the *relative* nature of the mind." As an example of the latter usage, Wallace (2006a, p. 137) says that "... [P]rimordial consciousness is the fundamental nature of the mind". Primordial consciousness is another term for pristine awareness (Wallace, 2006a, p. 137).

2.2 Access to the first meditative stabilization

See also 2.14.16 ("No sense-perceptions or body-perceptions").

Wallace (2006a)	"In Buddhism, all ordinary states of human consciousness, while awake or asleep, are said to belong to the *desire realm*, which is so called because this dimension of consciousness is dominated by sensual desires. All the nine stages leading up to the achievement of shamatha also belong to this realm, and it is only with the achievement of the mental and physical pliancy of shamatha that you gain access to the *form realm*." – p. 157 "Theravada Buddhists discuss a variety of counterpart signs that are perceived once you gain access to the form realm." – pp. 157-158 "The initial achievement of shamatha is described as *preliminary* or as *access* to the full realization of the first meditative stabilization (*dhyana*)." – p. 158 "A significant difference between access to the first stabilization and the actual state of that stabilization is that in the former, you gain only a tenuous freedom from the five hindrances, whereas in the latter, your immunity to them is stronger. There is a similar difference in the stability of the five factors of stabilization. There is a corresponding difference in the length of time you can remain immersed in such sublime states of focused attention. With access to the first stabilization, you can effortlessly remain in samadhi for at least four hours at a stretch, without the slightest perturbation from either subtle laxity or excitation. But once you have achieved the actual state of the first stabilization, samadhi can be sustained, according to Buddhaghosa, 'for a whole night and a whole day ...'." – p. 159
Wallace (2009/2014)	"The Buddha himself did not draw the subtle distinction between the full achievement of [the first meditative stabilization] and 'access concentration' to the first stabilization. But later Theravada and Mahayana contemplatives did, and according to many Mahayana Buddhists, this slightly less stable degree of samadhi is sufficient" – p. 85 Wallace p. 85 notes that "achievement of access to the first meditative stabilization [is] commonly known as *shamatha* (meditative quiescence)".

Wallace (2012/2014)	"Later Buddhist contemplatives have drawn a distinction between the actual state of the first dhyana and a lesser degree of samadhi that is on the threshold the first dhyana. In the latter, called 'access concentration' (Pali *upachara-samadhi*), the five hindrances are as dormant as they are in the actual state of the first dhyana, but samadhi is a little less robust. Instead of being able to rest effortlessly in unwavering samadhi for twenty-four hours, one may do so for only four hours – still far beyond anything considered possible according to modern psychology! This is what Tibetan Buddhists refer to when speaking of 'achieving shamatha' and 'settling the mind in its natural state'." – p. 152
Wallace (2011a)	"The achievement of shamatha ... is virtually equated with the achievement of the first dhyana; this is more accurately called 'access to the first dhyana'. Achieving shamatha is right on the threshold, where samadhi can be sustained for about four hours instead of twenty-four. Note that these are not precise limits." – p. 108
Wallace (1998/2005)	In the section on Tsongkhapa's vision/understanding:<br>"The initial achievement of quiescence is simultaneous with reaching the first proximate meditative stabilization ... If one wishes to proceed to more subtle states of quiescence, specific techniques may be followed that result in the achievement of the basic first stabilization." – p. 92

2.3 Substrate consciousness

2.3.1 General comments

Wallace (2006a)	"Both the Theravada and the Indo-Tibetan traditions of Buddhism agree that the cultivation of shamatha leads to an experiential realization of the ground state of the psyche ..." – p. 121 "The *substrate consciousness* (*alayavijñana*), as [the Dzogchen contemplatives] call it, consists of a stream of arising and passing moments of consciousness, so it is not permanent ... But they do regard it as a continuous stream of consciousness from which all mundane cognitive processes arise." – p. 122 "[Substrate consciousness] ... is the basis for the emergence of all appearances to an individual's mindstream." – p. 122 "The substrate consciousness may be characterized as the relative ground state of the individual mind, in the sense that it entails the lowest state of activity, with the highest potential and degree of freedom that can be achieved by evacuating the mind through the practice of samadhi." – p. 122 "By fathoming the nature of the substrate consciousness, one comes to know the nature of consciousness in its relative ground state." – p. 123
Wallace (2011b)	"Accessing [substrate consciousness] is the proper end of shamatha practice." – p. xii<br>"The substrate consciousness, a relative vacuum state of consciousness, is implicitly structured by concepts ..." – p. 15<br>"... substrate consciousness: the ground out of which the psyche and all of the ordinary manifestations of the mind emerge and into which they dissolve." – p. 22<br>Wallace p. 28 indicates that substrate consciousness involves "reified, preconscious structuring".<br>"Just as all sensory and mental appearances arise from [substrate consciousness], so do all subjective mental processes of the coarse mind arise from [it]." – p. 85<br>"What is the ground of your psyche? It's the substrate consciousness ..." – p. 95<br>"Now what is the substrate consciousness attending to? What is appearing to it? It's the substrate – that vacuity, an emptiness." – p. 176
Wallace (2012)	The glossary defines substrate consciousness as: "The ground of the ordinary mind, a continuum that persists from life to life and from which springs all ordinary mental activity. Prior to and more fundamental than the subconscious, it is considered the source of the psyche." – p. 163
Wallace (2011a)	"The psyche is an umbrella term referring to the array of mental states and activities we experience whenever the mind is active." – p. 196<br>"Resting in shamatha, awareness is the substrate consciousness ..." – p. 208
Wallace (2007a)	"Buddhist contemplatives claim that with the achievement of a highly advanced degree of [*samadhi*] known as [*shamatha*], or meditative quiescence, one gains experiential access to the relative ground state of consciousness known in the Great Perfection (Dzogchen) school of Tibetan Buddhism as the 'substrate consciousness' ([*alayavijnana*]). This, they claim, is the individual stream of consciousness from which the psyche and all the physical senses emerge." – p. 15

"The substrate consciousness may be characterized as the relative ground state of the individual mind in the sense that within the context of an individual mind stream, it entails the lowest possible state of activity, with the highest possible potential and degree of freedom or possibility." – p. 18

Wallace (2005) "The psyche ... includes the whole range of conscious and unconscious mental processes that are conditioned by the body, especially the brain, in interaction with the environment." – p. 164

2.3.2 Sleep, death, coma, fainting, hypnosis

Wallace (2006a) "... [S]ubstrate consciousness is repeatedly experienced in dreamless sleep, and it finally manifests at the moment of death." – p. 122
"In a further stage of dream yoga you release the dream ... and rest in a silent, luminous awareness of awareness itself, devoid of any other content. This is the state of lucid dreamless sleep, and in this state you may apprehend the substrate consciousness, and possibly even pristine awareness." – p. 150

Wallace (2011b) "We tap into the substrate consciousness at times quite naturally, without effort, without our having to be a great yogi. This happens, for instance ... in dreamless sleep ... In the dreamless state of deep sleep, mental activities become dormant and we slip into the substrate consciousness. Because our awareness is dull rather than luminous, we don't receive much benefit except for a good night's sleep." – p. 14
"We also experience the substrate consciousness at the time of death." – p. 14 "In the dying process the senses shut down one by one. They retract. The tentacles of awareness withdraw from the five physical senses back into mental awareness, where you still have imagination, thoughts, feelings, and so forth. And then, as you are withdrawing, the derivative mental processes of feeling, discernment, memory, imagination, recognition, and so on are also withdrawn ... [S]ome mental imagery arises, and then that too goes ... [T]he coarse mind ... become[s] utterly dormant by dissolving into the ground of ordinary mind, leaving no vestiges of imagery, personal history, or ego." – p. 15
"... [D]eep hypnosis ... is another situation where you are very close to the ground state of the ordinary mind." – p. 15

Wallace (2009/2014) "... [T]he substrate consciousness most regularly manifests during dreamless sleep ..." – p. 117
"When we fall into deep, dreamless sleep, all sensory and mental appearances dissolve back into the substrate ..." – p. 150

Wallace (2012/2014) "Resting in deep, dreamless sleep, when comatose, and when we die, the ordinary mind naturally withdraws into the substrate consciousness ... On these occasions, if one is not lucid – not recognizing the substrate for what it is – consciousness doesn't ascertain anything, for it has become entirely implicit rather than explicit. It is imbued with great stability but virtually no vividness. But if one retains consciousness while the mind dissolves into the substrate consciousness via shamatha, one apprehends the substrate with exceptional stability and vividness." – p. 188
"[Substrate consciousness] is spontaneously accessed when one falls into deep dreamless sleep, when one faints, and when one dies, but then one normally loses consciousness. However, it is possible to vividly experience the substrate by achieving the state of meditative quiescence, or shamatha." – p. 214

Wallace (2011a) "The substrate consciousness is the relative ground state from which the psyche emerges each time we awaken, and into which the psyche dissolves each time we fall asleep. When you access the substrate consciousness clearly and vividly by way of shamatha, it's like falling deep asleep while remaining luminously awake." – p. 109
"In the Theravadin tradition, the bhavanga is the mind's naturally pure state, but it is normally inaccessible and manifests primarily during deep sleep ... [H]owever, in falling asleep we generally lose clarity, and the ground state is veiled by dullness and sleepiness." – p. 232 "In stark contrast to this is access to the bhavanga via shamatha, which cultivates increasing luminosity and vividness of attention." – p. 232
"Ordinarily when we fall asleep, dullness overpowers our innate luminosity; this is why, on awakening, we can't remember what it was like to be asleep." – p. 232

Wallace (2007b) "When sensory and mental appearances naturally cease, as in deep sleep, the mind is normally so dull that we are incapable of ascertaining the substrate consciousness that manifests." – p. 47

Wallace (2007a) "... [D]eep hypnosis ... also taps into the substrate consciousness." – p. 18 "[Shamatha] entails vivid awareness of this dimension of consciousness, in contrast to the dullness that normally characterizes dreamless sleep." – p. 18

Wallace (2006b)	"... [D]eep hypnosis ... also taps into the substrate consciousness. But this potential is most effectively accessed when one lucidly penetrates to the substrate consciousness by means of meditative quiescence. In this case, one is vividly aware of the substrate, in contrast to the dullness that normally characterizes deep sleep." – p. 117

2.4 Bhavanga

Wallace (2006a)	"Both the Theravada and the Indo-Tibetan traditions of Buddhism agree that the cultivation of shamatha leads to an experiential realization of the ground state of the psyche. Early Buddhist literature refers to this as the bhavanga, literally, 'the ground of becoming' ..." – p. 121 "I believe Dzogchen contemplatives who have achieved shamatha gain access to this same dimension of consciousness, but they interpret it in a somewhat different way." – pp. 121-122
Wallace (2011b)	"... [Substrate consciousness] corresponds closely to the Theravada Buddhist term *bhavanga*, or 'ground of becoming' ..." – p. 14
Wallace (2012/2014)	"... a primitive continuum of awareness, which was known in early Buddhism as the ground of becoming (Skt. *bhavanga*). This bears a strong resemblance to what later schools of Buddhism referred to as the substrate consciousness ..." – p. 102<br>"The ground of becoming described in early Buddhism corresponds to what is called the substrate consciousness (Skt. *alaya-vijñana*) in the Great Perfection tradition of Tibetan Buddhism." – p. 115
Wallace (2011a)	"There are two terms that are used to describe the ground of the mind, and I am convinced that they refer to exactly the same experience, although some might debate this. In the Dzogchen tradition, the relative ground state into which the psyche dissolves in shamatha is called the *alayavijñana*, or substrate consciousness. The term '*alayavijñana*' does not appear in the Pali Canon or in the Theravadin commentaries; however, another term, *bhavanga*, is used instead, which can be translated as 'ground of becoming' ..." – p. 166<br>Wallace p. 208 notes that in the Theravadin tradition the term bhavanga is used to describe a "ground consciousness" or "ground of becoming", but it is "not depicted in two aspects" as in Dzogchen. "... [I]n Dzogchen the two aspects are described" (p. 208) – substrate consciousness and substrate.<br>"The bhavanga is known as the natural, unconfigured state of mind, the ground state or substrate consciousness ..." – p. 233<br>"In the terminology of the early Pali Canon, no distinction is drawn between the substrate and the substrate consciousness – it's a system, which makes good sense. When the ... substrate consciousness is experienced there is no explicit bifurcation of experience between the substrate over there and the consciousness of it over here. Coming out of this experience, we may speak retrospectively about appearances of vacuity that we designate as the substrate. Awareness of it is not a separate thing being posited. One term, 'bhavanga', describes the system. The bhavanga consciousness emphasizes the awareness that occurs." – p. 235
Wallace (2007a)	"... [T]he *bhavanga* ... bears a striking resemblance to the substrate consciousness ..." – p. 95 "... [T]he parallels with the substrate consciousness as described in the Great Perfection school are remarkable, especially in light of the fact that these two traditions of Buddhism have had very little contact over the centuries." – p. 96
Wallace (2005)	Wallace p. 77 discusses the *bhavanga* and then says: "Contemplatives in the Dzogchen tradition of Tibetan Buddhism access this same ground state of individual consciousness, which they call the *substrate consciousness* (*alayavijñana*). Although the Theravada and Dzogchen interpretations of this vacuous state vary in some respects, I am convinced that they are referring to the same experience ..." – p. 77
Wallace (2006b)	"[The *bhavanga*] appears to be identical to the substrate consciousness ..." – p. 112

2.5 Substrate

Wallace (2006a)	"The Dzogchen tradition draws a distinction between the substrate consciousness and the substrate (*alaya*), which is described as the objective, empty space of the mind." – p. 123

Wallace (2011b)	"The substrate is the *alaya*; substrate consciousness is the *alayavijñana*. When you slip into the substrate consciousness, what you're attending to, experiencing, what's appearing to your mind, to your substrate consciousness, is the substrate, the *alaya*. The *alaya* is a vacuity ..." – p. 175
Wallace (2009/2014)	"... a kind of vacuum into which all mental contents have temporarily subsided." – p. 94<br>Quoting Düdjom Lingpa: "The ... substrate is ... devoid of thought, a space-like vacuity and blankness in which appearances are suspended." – p. 94<br>"The basis of the mind and everything that appears to it is the space of the mind known as the substrate." – p. 149 "... [T]he space of the substrate ... [is] a luminous, empty space from which all objective and subjective experiences arise." – p. 149 "... [A]ll our discursive thoughts, mental images, desires, and emotions arise within the substrate." – p. 150
Wallace (2012)	"... a blank, unthinking void, immaterial like space and empty of appearances." – p. 138<br>The glossary defines the substrate as "The space of the mind that appears to the substrate consciousness: a luminous vacuity in which self, others, and objects disappear" (p. 162).
Wallace (2011a)	"The substrate (Skt. *alaya*) and the substrate consciousness are not the same. The substrate is the space of the mind itself, and the substrate consciousness is awareness of that space." – p. 109<br>"... [T]he substrate is an objective appearance to the mind, even though it happens to be a vacuity." – p. 155<br>"Resting in shamatha, awareness is the substrate consciousness, and the object of awareness is the substrate." – p. 208<br>"... [T]he substrate is the space of the mind: the field in which appearances arise. Not only mental appearances, but all visual, auditory, olfactory, gustatory, and tactile appearances ... All perceptual and conceptual phenomena appear within the substrate ..." – pp. 208-209 "In this context, we are using the word 'space' to denote a domain or a field, such as a stage on which actors might perform a play. This word is simply being used to indicate features of actual experience." – p. 209<br>"The substrate, which is formless, shapeless, colorless, and dimensionless, generates every aspect of form, shape, color, and dimension." – pp. 235-236<br>"Eventually, the mind completely settles in its natural state ... What remains is the appearance of the substrate and the awareness of the substrate consciousness ..." – p. 239
Wallace and Hodel (2008)	"The *substrate* (*alaya*) is the objective, empty space of the individual's mind – blank and unthinking, yet luminous ..." – p. 185 "The substrate is experienced by the substrate consciousness." – p. 185<br>"The substrate consciousness is that which can be *conscious of* the substrate ..." – p. 191

2.6 Distinction between substrate consciousness and pristine awareness

Wallace (2006a)	"In contrast to the substrate consciousness, which can be viewed as the relative ground state of the mind, pristine awareness (Tibetan: *rigpa*, Sanskrit: *vidya*) may be characterized as the absolute ground state of consciousness. Also known as primordial consciousness (*jnana*), the realization of this ultimate dimension of consciousness is a central theme of Dzogchen practice. This state entails the lowest possible state of mental activity, with the highest possible potential and degree of freedom of consciousness. Indivisible from primordial consciousness is the absolute space of phenomena (*dharmadhatu*), which transcends the duality of external and internal space." – p. 137 "Out of this space – nondual from primordial consciousness – emerge all the phenomena that make up our experienced world. All appearances of external and internal space, time, matter, and consciousness emerge from the absolute space of phenomena and consist of nothing other than configurations of this space ... While the relative vacuum of the substrate can be ascertained by means of the cultivation of shamatha, this absolute vacuum is usually realized only through the cultivation of vipashyana." – p. 137 "... [P]rimordial consciousness is the fundamental nature of the mind." – p. 137<br>"The unity of absolute space and primordial consciousness is the Great Perfection, often referred to as the 'one taste' of all phenomena." – p. 138<br>"The substrate consciousness may be called a relative, or false, vacuum state of consciousness, for it is different from the substrate that it ascertains; it is qualified by distinct experiences of bliss, luminosity, and nonconceptuality; it is ascertained when the mind is withdrawn from the external world; and it is bound by time and causality – specific to a given individual. Therefore, despite its vacuity, it has an internal structure. The unity of absolute space and primordial consciousness, on the other hand, is the absolute or true, vacuum. Although it, too, is imbued with the qualities of bliss, luminosity and nonconceptuality, these are not present as distinct attributes (as they are in the substrate consciousness)." – p. 138

	"When one realizes the substrate consciousness by achieving shamatha, mental afflictions are only temporarily suppressed ..." – p. 138 Wallace pp. 93, 120, 121, 123 makes similar comments. "... [B]ut as a result of realizing primordial consciousness, it is said that all mental afflictions (*klesha*) and obscurations (*avarana*) are eliminated forever. Likewise, the bliss that is experienced when resting in the relative ground state of consciousness is limited and transient, whereas the inconceivable bliss that is innate to the absolute ground state of primordial consciousness is limitless and eternal." – p. 138<br>"The Dzogchen and Mahamudra traditions, most strongly associated with the Nyingma and Kagyü orders of Tibetan Buddhism, respectively, view the pristine awareness as a perfectly enlightened state of consciousness that is already present, but obscured by mental afflictions and other obscurations." – p. 144<br>"... primordial consciousness, or buddha nature ..." – p. 144<br>"... [Y]ou may receive 'pointing-out instructions' that arouse in you an experience of pristine awareness, but if you have not deeply cultivated the stability and vividness of your attention, it is unlikely that you will be able to sustain that experience." – p. 148
Wallace (2011b)	"The substrate consciousness is the foundation and source of an individual's psyche." – p. xii "... dharmakaya – that is, pristine awareness, or *rigpa* ... The dharmakaya, or buddha mind ... is much deeper than the individual mind ..." – p. xii<br>"The 'absolute space of phenomena' is my translation for *dharmadhatu* ... 'Absolute space' here means the space out of which relative space, time, mind, matter, and all other dualities and phenomena emerge. It is the ground of being, the primordial ground. Its relationship with primordial consciousness (*jñana*) is nondual." – p. 2 "Primordial consciousness, your own *rigpa*, or pristine awareness, is that out of which all relative states of consciousness emerge and is nondual from the absolute space of phenomena." – p. 2<br>"We can establish a three-dimensional model of the mind. First there is the psyche ... The psyche emerges from what Sogyal Rinpoche calls the 'ground of the ordinary mind'." – p. 14 "The Sanskrit term for this second layer, the ground of the ordinary mind, is *alayavijñana*, which is translated as the 'substrate consciousness' ... So the substrate consciousness is deeper than the psyche, but it is still not buddha nature, which is the third and most profound level in our three-tiered model." – p. 14 "The ground of the ordinary mind is individual, conditioned, and linear with time – it is within the causal nexus." – p. 15<br>"When you enter [substrate consciousness] ... [y]ou have reached the ground of the ordinary mind, but you have not tapped into the ultimate ground, primordial consciousness." – p. 27
Wallace (2012/2014)	Wallace p. 191 describes pristine awareness as "the deepest dimension of consciousness".
Wallace (2012)	The glossary defines primordial consciousness as: "The deepest and most fundamental level of the mind. A synonym for buddha-nature, absolute bodhichitta, rigpa, darmakaya, pristine awareness, enlightenment" – p. 161
Wallace (2011a)	"... buddha nature, the deepest level of our own awareness, also called pristine awareness (Tib. *rig pa*)." – p. 7
Wallace and Hodel (2008)	"Primordial consciousness – characterized as the absolute ground, the most basic state of consciousness ..." – p. 192 "The experience of the substrate consciousness produces states that are nonconceptual, luminous, and blissful. However, with the penetration of primordial consciousness it is said that one experiences these as a unity that is beyond words to describe." – p. 193
Wallace (2005)	"There are many subtle differences between [substrate consciousness and pristine awareness], but the most important is that simply dwelling in the substrate consciousness does not purify or liberate the mind from any mental afflictions, whereas dwelling in pristine awareness radically frees the mind of its obscurations ..." – pp. 41-42
Wallace (2001a)	"While the cultivation of meditative quiescence alone may withdraw one's mind into [the bhavanga], that does not ensure that one will actually *ascertain* the clear, empty, luminous nature of the mind. That is ... the goal of Dzogchen ..." – p. 226

2.7 Potential for rare individuals to achieve pristine awareness through shamatha practice

Wallace (2006a)	"The cultivation of shamatha is widely known as a means of ascertaining ... substrate consciousness. But in some cases ... the [awareness of awareness] practice ... may be sufficient for ascertaining the nature of pristine awareness ..." – p. 136 "[In that practice] [t]he illusory, independent ego is temporarily put out of

	work and for exceptional individuals who have 'little dust on their eyes', to use an ancient metaphor, this may be sufficient for fathoming the ultimate nature of the mind and its relation to reality as a whole." – p. 138
Wallace (2011b)	Quoting from the *Vajra Essence* by Düdjom Lingpa: "By [practising the settling the mind in its natural state practice] a person of superior faculties directly actualizes the nature of existence of suchness – ultimate reality ..." – p. 105 Wallace comments: "Here *suchness* is really a synonym for emptiness. *Ultimate reality* as well, generally speaking, is a synonym for emptiness." – p. 105 "... [I]t is possible that a person [practising the settling the mind in its natural state practice, and without ever having done vipashyana], a person of [superior/sharp] faculties, someone very ripe, or as the Buddha said, 'with very little dust on the eyes', could realize emptiness." – p. 105 Wallace notes that this is similar to Padmasambhava's comment that the awareness of awareness practice, in Wallace's words, "may be enough to fathom rigpa" (p. 105). Wallace says, "The [awareness of awareness] approach is expressly designed for shamatha, but for a person of sharp faculties, just that modest shamatha technique may be enough to fathom rigpa." – p. 105
Wallace (2009/2014)	Wallace p. 167 describes a form/phase of practice he describes as "nonmeditation", which appears similar to the awareness of awareness practice (or some phase within it). "This phase of practice is sometimes called 'nonmeditation', for you are not meditating on anything. Simply place your awareness in the space in front of you and maintain unwavering mindfulness without taking anything as your meditative object." – p. 167 "In this practice of 'not doing', you may simply experience a deep inner stillness. Beyond this, you may break through your psyche and even transcend the substrate consciousness, as your awareness settles in its ultimate ground, known as primordial consciousness ..." – p. 167 "For some people, it may take years of dedicated meditative practice before such a realization occurs. But for others it may arise quite soon. This all depends on our degree of spiritual maturity." – p. 168
Wallace (2001/2003)	"The Tibetan Buddhist tradition reports rare and exceptional individuals for whom the practice of settling the mind in its natural state alone has been sufficient to attain full awakening ... But for most of us quiescence alone is not enough." – p. 131
Wallace (1999a)	"For exceptional individuals, the ... method of settling the mind in its natural state may be sufficient for gaining [pristine awareness]; but for most people, further training beyond *Samatha* is required ..." – p. 186

2.8 Potential for glimpses of pristine awareness

Wallace (2011b)	Wallace pp. 151-152 discusses how it is possible for "[t]he substrate consciousness ... [to] become transparent, so things flow through it more easily from below, from rigpa" (p. 152). "So it appears that from the platform of shamatha – even though you have not yet mastered the ground awareness, or rigpa, buddha nature – you can get these 'sneak previews'. You are able to dip your cup into the ocean of rigpa and bring out a little thimbleful." – p. 152<br>"... [E]ven if you get some taste of rigpa, unless you are able to ... sustain it, then it is very easy to lose it." – p. 169
Wallace (2012/2014)	"Without achieving ... an experience of stable and vivid *samadhi* through the achievement of shamatha, we may catch fleeting glimpses of pristine awareness, but we are unlikely to sustain it or readily access it again. Consequently, such breakthrough experiences may soon disappear, leaving only a fading memory and a lingering sense of nostalgia." – p. 219
Wallace (2012)	"While we are developing shamatha, experiences relating to much deeper layers of consciousness may spontaneously appear. These can be likened to shafts of light breaking through the clouds of our normally conditioned consciousness." – p. 18
Wallace (2011a)	"Resting in the luminous, boundless space of the substrate, it is possible that you might have fleeting access to primordial consciousness." – p. 306
Wallace (2001/2003)	Discussing the settling the mind in its natural state practice: "In releasing ... dualistic fixation on mental and sensory objects, primordial awareness begins to shine through the veils of obscuration with greater and greater brilliance." – pp. 87-88

2.9 Accessing pristine awareness by releasing grasping

Wallace (2011a)	"Resting without reification in the substrate consciousness, we experience bliss, luminosity, and nonconceptuality. Each of these has its own appeal, and some people are drawn to one aspect over another. The challenge is to release all preferences, maintaining detached nonchalance. Release all grasping on to bliss. Release all preference for luminosity. Release all clinging to nonconceptuality. Release even the preference for the quiet seclusion of the mind withdrawn from the five senses. Utterly release into open presence." – p. 299 "This is the practice called breaking through (Tib. *khregs chod*) ... Just as we broke through the psyche to reach the substrate consciousness, we now break through the confines and limitations of our individual substrate consciousness to reach the infinite dimension of pristine awareness." – p. 299 "... [P]ristine awareness ... can be experienced by utterly releasing all grasping." – p. 300

2.10 Dormant/implicit conceptualization in substrate consciousness

Wallace (2011b)	"The substrate consciousness ... is implicitly structured by concepts ..." – p. 15<br>"... the reified, preconscious structuring ... of [substrate consciousness]." – p. 28
Wallace (2009/2014)	"Nonconceptuality in this context is experienced as a deep stillness. But it is not absolutely devoid of thoughts, for this dimension of consciousness is subliminally structured by concepts. " – pp. 91-92
Wallace (2012/2014)	"According to the Dzogchen view, even when one is resting in the substrate, there is a dormant proclivity for self-grasping that does not fade away, no matter how long one remains in that state." – p. 220 Wallace p. 220 describes it as a "latent consciousness of the mere appearance of a real self – although none in fact exists". The proclivity can be overcome via insight practice (pp. 220-223).
Wallace and Hodel (2008)	"The substrate consciousness can be compared to a *relative* vacuum. It is relatively empty, but still possesses structure and energy, characterized by such attributes as bliss (spiritual joy or rapture), luminosity (an internal radiance), and a muted sense of duality between subject and object." – p. 192
Wallace (2007b)	"Even though dualistic, discursive thoughts have subsided, [substrate consciousness] ... is implicitly structured by conceptual reification. The mind is temporarily in a state of relative equilibrium ... but as soon as it emerges from that meditative state, the asymmetries of dualistic thinking are catalyzed as before." – p. 112
Wallace (1998/2005)	In the section on Tsongkhapa's vision/understanding:<br>"Upon gaining access to the form realm [by achieving shamatha], one's consciousness continues to be structured by very subtle concepts; but Buddhist contemplatives assert that these are not uniquely human concepts." – p. 91<br><br>In the section on quiescence in Mahamudra/Dzogchen:<br>Wallace notes that on the view he considers "representative of the [Mahamudra] and [Dzogchen] traditions as a whole", "quiescence ... is not considered to be intellectually uncontrived, or unstructured, nor is it free of conceptual grasping" (p. 231). "Although the state of quiescence is said to be non-conceptual, the meaning here is that the mind is not consciously engaged in discursive thought. However, it seems that conceptualization is still operating on a subliminal level, and one's experience is still structured by one's previous conceptual conditioning ... [A]ccording to the [Dzogchen] tradition, one thoroughly transcends conceptual modification and grasping only in the unmediated realization of the empty and clear nature of the mind." – p. 231 "... [T]he achievement of quiescence is taught as a crucial step to reaching the conceptually unstructured and unmediated realization of ultimate truth by means of the cultivation of insight." – p. 232 Wallace notes with reference to particular forms of shamatha practice that "at least some subliminal ideation persists" (p. 239). "... [A]s Karma Chagmé describes it, there remains a lingering thought: 'attention is being sustained'. Thus, the various techniques of quiescence alone are regarded as being insufficient for entering a contemplative state that is truly intellectually uncontrived and free of conceptual grasping." – pp. 239-240 "According to the ... [Dzogchen tradition], in order to realize ultimate truth in a manner that is intellectually unstructured and free of conceptual grasping, one must ... seek out the ultimate nature of phenomena by means of insight practices." – p. 240
Wallace (2001/2003)	Wallace p. 133 refers to "habitual grasping" as being in "a state of abeyance", as opposed to being "completely severed right at the root".

Wallace (2000)	"Even when the mind is settled in meditative stabilization without human conceptual constructs, it is not considered by Buddhist contemplatives to be entirely free of all traces of conceptualization. One's inborn sense of a reified self as the observer and the reified sense of the duality between subject and object are still present, even though they may be dormant while in meditation; and when one emerges from this nonconceptual state, the mind may still grasp onto all phenomena, including consciousness itself, as being real, inherently existing entities. To penetrate to the fundamental nature of appearances and their relation to consciousness, it is said that one must go beyond meditative stabilization and engage in training for the cultivation of contemplative insight." – p. 112<br>Discussing Forman's (1990) concept of pure consciousness: "... [T]he mere fact that one has temporarily disengaged one's attention from all words, thoughts, and mental contents does not necessarily imply that one's experience is no longer at least subliminally structured by one's conceptual framework. The well-known processes of 'precognitive structuring' and 'subliminal priming' of experience are bound to play a role in most, if not all, conscious states. Thus, the experiences of two people with different backgrounds who enter such a state of seemingly pure consciousness may be significantly different, as may be the residual, lingering effects of their experiences." – p. 118<br>Wallace notes that in shamatha practices contemplatives "seek to disengage from their conceptually structured experiences derived from both sensory and mental perception and to enter a state free of all subjective constructs" (p. 119). "However, many Buddhist contemplatives have been quite aware of the common error of mistaking such a conscious state for one that is utterly unstructured by language and concepts. With this recognition, contemplatives ... have devised further contemplative methods for 'breaking through' *all* conceptual mediation to a state of primordial awareness ..." – p. 119
Wallace (1999a)	Wallace p. 183 describes a shamatha practice where "the object of mindfulness is the mere absence of ideation". This appears to be the awareness of awareness practice, or a practice that is similar. In the section on the non-ideation practice, Wallace says: "Buddhist contemplatives raise the question as to whether this non-conceptual state of *Samatha* actually transcends all conceptual structuring and modification and whether the mere suppression of ideation is sufficient for entering a totally non-conceptual state of awareness. The eminent Tibetan Buddhist contemplative Karma Chagmé (1612-1678) voices the general consensus within the Tibetan tradition when he asserts that although this state may easily be mistaken for conceptually unstructured awareness, it is not unmodified by ideation; for one still maintains the conceptual sense that one's attention is being sustained in the absence of conceptualization." – pp. 183-184 In the next section, Wallace notes that the settling the mind in its natural state practice does not involve suppression of ideation (p. 184).
Wallace (2001b)	"According to Buddhism, the *bhavanga* is not a true or absolute vacuum because precognitive conceptual structure of awareness still persists. Even though concepts such as subject and object are not manifest, awareness is still structured by conceptual and biological influences." – p. 24

2.11 Use of substrate consciousness for particular purposes

Wallace discusses how substrate consciousness can be used for different purposes. For example: "Having accessed the substrate consciousness, there is the potential for tapping into memories from past lifetimes ... These may appear in the form of dreams, visions, desires, or fears, whose origin is not to be found in this lifetime." (Wallace, 2011b, p. 30); "... [I]t's clear that by achieving shamatha you arrive at the transparency of the ground of the ordinary mind such that, with a little effort and through skillful means, you can develop clairvoyance." (Wallace, 2011b, p. 152); "... [W]hen you achieve Shamatha you can experientially access dimensions of existence that were previously hidden from view. You've tapped into your substrate consciousness ... and you have access to the form realm ..." (Wallace, 2011b, pp. 160-161)"; "As our awareness gravitates toward the substrate consciousness, we approach a superfluid state of awareness saturated by deep knowledge that is implicit rather than explicit. Moments of inspiration give rise to flows of intuition and creativity. By tapping into this wellspring, we can use insight to resolve practical and interpersonal issues, even when they are not explicit." (Wallace, 2011a, p. 187). These possibilities are not the focus of this table, and have been noted here only for completeness. It appears that use of the substrate consciousness may in each case involve an element of doing or effort.

2.12 Potential to analyze

Although Wallace repeatedly states that substrate consciousness is devoid of thoughts (see 2.14.14, "No thoughts"), Wallace (2011a) indicates that upon attaining the state the meditator is free to think and analyze with a high degree of clarity. It appears that the way to reconcile these comments is to understand that in substrate consciousness the meditator has the *potential* to think and analyze. The

person may exercise that potential by taking the active step of initiating thought and analysis, however upon doing that they will no longer be in the substrate consciousness as it is ordinarily conceived and described by Wallace in the context of Shamatha practice.

Wallace (2011a)	"The Buddha described this very profound state of samadhi, the first dhyana, as being accompanied by coarse and precise investigation. The mind is utterly controlled and settled in a state of equipoise that is nothing like a trance, in which you cannot think or function. To the contrary, in this state you can engage in general investigations or precise analysis of any subject. Your intelligence and conceptual abilities are fully available, but you are completely free of obsessive-compulsive thinking." – p. 97 "This state is imbued with discerning intelligence ..." – p. 97<br>"You are approaching the first dhyana, in which your abilities to think clearly and analytically are available, should you wish to use them for coarse or subtle investigation." – p. 105<br>"Meditative quiescence is not like being gagged and bound. The mind is settled in a state of peaceful, luminous silence in which you can think at any time. Your abilities to investigate and analyze are on tap, but you are no longer subject to obsessive-compulsive ideation." – pp. 110-111<br>"Vipashyana entails active inquiry, in a useful form of grasping that poses pointed questions rather than simply allowing our conceptualizations to go dormant. In order to observe and investigate the nature of the mind that grasps and reifies, we cannot simply relax in the hot-tub experience of the substrate consciousness." – p. 298 "It is crucial to note that, unlike the higher dhyanas, when we achieve access to the first dhyana, we retain our abilities of coarse investigation and subtle analysis ... Undistracted intelligence and comprehensive analytical skills are potentiated in the quiet domain of the substrate consciousness. A mind stripped down to the ground of the substrate consciousness is the ideal laboratory for probing directly into the very nature of mind because only the space of the mind appears – all distractions have vanished." – p. 298

2.13 Speed at which achieved

See 1.23 ("Time/effort taken to progress, and factors supporting progress").

2.14 Features

2.14.1 Recognize in retrospect

Although Wallace does not explicitly address the issue, it appears that, due to the absence of introspection, thinking, and conceptualization during the experience, the meditator would not have any sense at the time of, "I'm experiencing quiescence". That understanding is supported by Wallace's (2006a) comment that: "While abiding in shamatha, you may have little or no experience of the passage of time, for the sense of time requires memory, which is activated through conceptualization; so in the absence of conceptualization, you dwell in a state of consciousness that feels timeless." – p. 162 The detailed descriptions of quiescence in the texts indicate that the meditator can recall the experience upon emerging from it. That understanding is consistent with Wallace's (2011a) remark in relation to mindfulness practices generally, that: "Mindfulness includes not only present-centered mindfulness, but also retrospective memory of your experiences. Following each practice, note how well you maintained your object of attention. Were you able to follow the practice instructions? How did your experience compare with expectations? What will you do differently next time? ..." – p. 22 It is also consistent with his comment: "Recall that a primary definition of mindfulness is the all-important faculty of memory. Just as a recording device captures images or sounds for later review, mindfulness captures experiences and recalls them later." – p. 268

2.14.2 Conscious

See also 2.14.7 ("Luminosity, clearness, limpidity, transparency, radiance, purity").

Wallace (2006a)	"A contemplative may consciously probe [substrate consciousness] through the practice of shamatha ..." – p. 122<br>"The mind has been reduced to its bare nature, a relative vacuum state. But that emptiness is still illuminated by consciousness." – p. 133
Wallace and Hodel (2008)	"The *substrate* (*alaya*) is the objective, empty space of the individual's mind – blank and unthinking, yet luminous ..." – p. 185 "The substrate is experienced by the substrate consciousness." – p. 185<br>"The substrate consciousness is that which can be *conscious of* the substrate ..." – p. 191

2.14.3 Consciousness/awareness itself

See also 1.14 (“Practice 3 – Awareness of awareness”).

Wallace (2006a)	Wallace p. 132 explains that the awareness of awareness practice is about being aware of awareness. He says: “The mind has been reduced to its bare nature, a relative vacuum state. But that emptiness is still illuminated by consciousness. And this awareness is innate, still, and vivid. The qualities of attention you have earlier been developing through the practices of shamatha are already implicit in the nature of awareness itself. They have just been waiting to be unveiled.” – p. 133<br>“... the sheer luminosity and cognizance of being aware.” – p. 133<br>“In a further stage of dream yoga you release the dream ... and rest in a silent, luminous awareness of awareness itself, devoid of any other content. This is the state of lucid dreamless sleep, and in this state you may apprehend the substrate consciousness, and possibly even pristine awareness.” – p. 150
Wallace (2009/2014)	In the awareness of awareness practice: “As you become more and more familiar with this practice, you may gradually unveil the stillness and luminosity that are intrinsic to awareness.” – p. 73<br>“When [in substrate consciousness] the physical senses are dormant and the activities of the mind are calmed, all that remains is mental awareness ...” – pp. 90-91
Wallace (2011a)	“Resting in shamatha, awareness is the substrate consciousness, and the object of awareness is the substrate.” – p. 208<br>In the awareness of awareness practice: “Observe the referent of the term ‘awareness’: that which is luminous and cognizant.” – p. 240<br>Discussing the awareness of awareness practice: “When you achieve shamatha on this trajectory, your awareness is of the substrate consciousness, which is a mode of awareness.” – p. 249<br>“Here we have ascertained the nature of consciousness itself, though not its ultimate nature.” – p. 298
Wallace (2007b)	Wallace conveys the experience by having the reader picture a sensory deprivation tank experience where “you are vigilantly aware of nothing but your own experience of being conscious” (p. 46).
Wallace (2005)	“Right in the nature of awareness itself there is luminosity and clarity ... [I]t is innate to awareness itself.” – p. 39 Wallace then refers to this as “this intrinsic stillness and luminosity of awareness” (p. 39).<br>“... the innate stillness, stability, and vividness of awareness itself.” – p. 40
Wallace (1998/2005)	In the section on Tsongkhapa’s vision/understanding:<br>“... [T]here arises a non-dual awareness of consciousness itself.” – p. 92<br>“... [U]pon the attainment of quiescence ... the attention is withdrawn from all signs and is focused on the nature of awareness itself ...” – p. 108<br>“... [P]aths to quiescence finally result in an experiential realization of the nature of consciousness.” – p. 208<br><br>In the section on quiescence in Mahamudra/Dzogchen:<br>“... [I]t does seem clear from Tsongkhapa’s discussion ... that he believes the cultivation of quiescence to culminate in an experiential realization of the nature of consciousness. This assertion need not be interpreted as contradicting the premise, accepted by Tsongkhapa, that the mind cannot apprehend itself ...” – p. 231 “... [O]nce quiescence is accomplished ... the continuum of one’s attention may attend to previous moments of consciousness. Due to the homogeneity of this mental continuum, the experiential effect would be that of the mind apprehending itself.” – p. 232
Wallace (2000)	“... [Shamatha training is presented by Asanga] as a means for experientially ascertaining the nature of consciousness itself ...” – p. 108 “This assertion need not be interpreted as contradicting the hypothesis that consciousness cannot apprehend itself. That premise denies that a single consciousness can have itself as its own object ... [O]nce meditative stabilization is accomplished, and one’s meditative object dissolves, in this absence of appearances the continuum of one’s attention may attend to *previous* moments of consciousness. Because of the homogeneity of this mental continuum, the experiential effect would be that of the mind apprehending itself.” – p.108<br>“... [I]n this meditative state, one is said to ascertain the essential features of consciousness vividly, single-pointedly, and without conceptual mediation ...” – p. 109
Wallace (1999a)	“... [E]ven though it is ... not possible for a single moment of consciousness to observe itself, one moment of consciousness may recall the experience of the immediately preceding moment of consciousness, which, in turn, may recall its immediately preceding moment – each moment having no other appearances or objects

	arising to it. Thus, due to the homogeneity of this mental continuum ... the experiential effect is that of consciousness apprehending itself." – p. 183

2.14.4 Awake, wakefulness
See also 2.3.2 ("Substrate consciousness – Sleep, death, coma, fainting, hypnosis").

Wallace (2009/2014)	"[The relative ground state of consciousness] is normally inaccessible ... so to unlock the power of the bhavanga, the mind must be fully 'woken up' by meditative development ..." – p. 90<br>In shamatha, "your mental awareness [is] highly stable and alert" (p. 92).
Wallace (2012/2014)	Quoting Matripa: "The nature of meditative equipoise is ... a clear vacuity ... Allowing anything to arise, it is vividly awake." – p. 199
Wallace (2011a)	"When you access the substrate consciousness clearly and vividly by way of shamatha, it's like falling deep asleep while remaining luminously awake." – p. 109
Wallace (2005)	"... [Y]ou don't fall asleep or become dopey. A luminous awareness remains ..." – p. 42

2.14.5 Non-duality
See also 1.17 ("Non-duality").

Wallace (2006a)	"... [Y]ou experience a nondual awareness of consciousness itself." – p. 161
Wallace (2011b)	Wallace p. 173 discusses how having mindfulness of an object implies "a subject over here and an object over there ... tied down by mindfulness". He then explains that, in substrate consciousness, mindfulness of an object is released (p. 173).
Wallace (2009/2014)	"... [Substrate consciousness] is undifferentiated in terms of any distinct sense of subject and object." – p. 92<br>"Describing [the substrate] is difficult because, [in substrate consciousness], due to the relative absence of thoughts of 'I' and 'not I', there is no distinct experience of a division between subject and object. You now have a 'subjective' awareness of the substrate that appears as your object ..." – p. 94
Wallace (2012)	The glossary defines "one pointedness. (Sanskrit: *samadhi*)" as "[p]erfected meditative concentration where observer and observed are nondual" (p. 161).
Wallace and Hodel (2008)	"The substrate consciousness can be compared to a *relative* vacuum. It is relatively empty, but still possesses structure and energy, characterized by such attributes as bliss (spiritual joy or rapture), luminosity (an internal radiance), and a muted sense of duality between subject and object." – p. 192
Wallace (2007b)	"Due to the relatively nonconceptual nature of this state of consciousness, there is no distinct experience of a division between subject and object, self and other. Relatively speaking, the subjective substrate consciousness is nondually aware of the objective substrate ..." – p. 46<br>"[The] luminous space [of the substrate] is undifferentiated in terms of any distinct sense of subject and object." – p. 48
Wallace (2005)	Discussing the awareness of awareness practice: "Subject-versus-object may also vanish, especially if it turns out that the subject/object dichotomy ... consists of nothing more than fabrications that we superimpose on experience. If these are artificial superimpositions, then by releasing them we may settle into a underlying state of nonduality." – p. 40<br>"... [G]rasping onto 'I' and 'mine' vanishes, as does any sense of a subject/object duality." – p. 89
Wallace (1998/2005)	In the section on Tsongkhapa's vision/understanding:<br>"... [T]here arises a non-dual awareness of consciousness itself." – p. 92
Wallace (1999b)	"Even in this state ... the distinction between subject and object remains, for now one retrospectively discerns the nature of preceding moments of consciousness that is disengaged from all appearances." – p. 444

2.14.6 Bliss, joy, pleasantness, wellbeing, absence of discomfort
See also 2.14.22 ("Calm, ease, peacefulness, absence of disturbance") and 1.11.10 ("The ten stages – Stage 10: Shamatha").

Wallace (2006a)	"The 'natural state' of the mind, according to Buddhist contemplatives, is characterized by the three qualities of bliss, luminosity, and nonconceptuality." – p. 81<br>"... [W]hen your mind is settled in its natural state, you discover for yourself the innate qualities of bliss, luminosity, and stillness that have always been there." – p. 91<br>Quoting Düdjom Lingpa: "You may also have an extraordinary sense of joy, luminosity, and non-conceptuality." – p. 109<br>"Contemplatives who have realized the substrate consciousness through the practice of shamatha claim that it is imbued with three attributes: bliss, luminosity, and nonconceptuality." – p. 123<br>Quoting Düdjom Lingpa: "You will become still in an unfluctuating state, in which you will experience joy like the warmth of a fire ..." – p. 161<br>"... [Y]our mind ... is suffused with an inner sense of well-being, in which it is impossible for ... any kind of discomfort to arise." – p. 161
Wallace (2011b)	"The accomplishment of shamatha is accompanied by a powerful experience of bliss, luminosity, and stillness." – p. ix<br>"... [The experience of] "joy in the form realm ... can be achieved by way of shamatha. Shamatha provides you with access to the form realm. It crosses that threshold to the experience of bliss." – p. 106<br>"... shamatha, or quiescence – a state of bliss, luminosity, and nonconceptuality." – p. 112<br>"Three qualities that you will be able to identify distinctly – bliss, luminosity, and nonconceptuality – will manifest in you." – p. 120<br>"... [S]ince our afflictions are attenuated, this is no longer fertile ground for the afflictions to continue to arise. One of the little dividends is bliss. We experience a sense of bliss or joy that is quiet and serene – the joy of shamatha." – p. 145 "... a quiet, percolating, radiating sense of serenity, joy that is very malleable." – p. 145<br>Wallace p. 180 describes substrate consciousness as a "blissful, luminous vacuity".
Wallace (2009/2014)	"... [Substrate consciousness] is characterized by three essential traits: bliss, luminosity, and nonconceptuality. Bliss does not arise in response to any sensory stimulus, for the physical senses are withdrawn ... Nor does it arise in dependence upon pleasant thoughts or mental images, for such mental activities have become dormant. Rather, it appears to be an innate quality of the mind when it has settled in its natural state, beyond the disturbing influences of conscious and unconscious mental activity." – p. 91
Wallace (2012/2014)	"Contemplatives who have become adept in such training report that a sense of inner well-being emerges spontaneously when the mind is freed from its habitual tendencies of laxity and excitation. This remarkable discovery ... asserts that joy is the natural affective state of a balanced mind ..." – p. 167
Wallace (2011a)	"... [s]amadhi feels quite nice ... You are resting in a state that can easily be mistaken for buddha nature, pristine awareness, Atman, Brahman, ultimate reality, or God consciousness. Nevertheless, this is merely a quiescent mind resting in its own luminous, blissful, silent, relative ground state." – p. 40<br>With reference to the first dhyana, as described by the Buddha: "This state is suffused with a blissful well-being; it's not ecstasy or teeth-chattering, incapacitating bliss ... When the mind is settled in the first dhyana, bliss arises from the very nature of awareness." – p. 97<br>"When you achieve samadhi, particularly with the deeply focused attention of shamatha, a sense of well-being arises." – pp. 169-170
Wallace (2010)	"When [shamatha] practice is nested in a proper context, it's possible to recognize ... that our own minds have an avenue to serenity and peace. And from that peace of mind, that sense of ease and contentment ... there arises a happiness and satisfaction. The satisfaction comes from a very simple source: a mind that's not being pummeled to death with afflictions, craving, hostility, and aversion. We just don't normally give ourselves this break." – pp. 31-32
Wallace and Hodel (2008)	"The substrate consciousness can be compared to a *relative* vacuum. It is relatively empty, but still possesses structure and energy, characterized by such attributes as bliss (spiritual joy or rapture) ..." – p. 192
Wallace (2007b)	Wallace pp. 45-46 makes a similar comment to Wallace (2009/2014) p. 91 above.

	Wallace p. 100 refers to "the bliss of achieving meditative quiescence alone". He then explains that "the joy of quiescence stems from temporary freedom from the attentional imbalances of laxity and excitation" (p. 100).
Wallace (2005)	"This crisp and clear bliss arises from the nature of your awareness simply because the mind is stabilized with vividness." – p. 46<br>"... the subtle joy of serenity, an inner calm." – p. 109
Wallace (1998/2005)	In the section on Tsongkhapa's vision/understanding:<br>"In the state of meditative equipoise, only the aspects of awareness, clarity, and joy of the mind appear, and all one's other sense faculties remain dormant." – p. 82<br>Tsongkhapa says, "Pleasure and joy in the sense of a feeling of well-being do occur as a result of the [*samadhi*] to be practiced within this context, but ... they do not arise in conjunction with the [*samadhi*] comprised by the first proximate stabilization ..." – pp. 155-156 On this point, Wallace comments: "Although mental and physical joy are experienced in conjunction with the pliancy that immediately precedes the accomplishment of the quiescence of the first proximate stabilization, these feelings subside with the onset of the first proximate stabilization itself. If one continues on the accomplishment of the first basic stabilization, pleasure and joy are experienced again ..." – p. 156 Tsongkhapa later says "... [W]hen you settle in meditative equipoise, only the aspects of the sheer awareness, clarity, and vivid joy of the mind appear ... Then [by continuing the practice] ... the sense of ongoing joy ... naturally [subsides] ... Joy ... then become[s] subtle." – p. 207<br>"[Quiescence] is ... immediately preceded by an experience of extraordinary joy ..." – p. 212<br>"Tsongkhapa ... states that upon achieving quiescence, the mind disengages from the signs of sensory objects, and only the aspects of the sheer awareness, clarity, and vivid joy of the mind appear. Thus, joy is said to arise from the very nature of consciousness once it is free of the afflictions of laxity and excitation and is disengaged from all sensory and mental appearances." – p. 229
Wallace (2001/2003)	"... [Q]uiescence is a nice place to hang out." – p. 135<br>"By settling the mind in its natural state, a sense of well-being will spring forth from the very nature of awareness ..." – p. 143
Wallace (2000)	"Upon achieving [quiescence], both [Asanga] and Vasubandhu assert ... only the aspects of the sheer awareness, luminosity, and vivid joy of the mind appear. Thus, these contemplatives ... present us with the truly astonishing hypothesis that joy arises from the very nature of consciousness once it is free of the afflictions of laxity and excitation and is disengaged from all sensory and mental appearances." – p. 108<br>"This state is characterized as one of joy, luminosity, and nonconceptuality." – p. 108
Wallace (1999a)	"Only the aspects of the sheer awareness, clarity, and joy of the mind appear ..." – p. 182<br>"... *Samatha* ... is characterized by joy, clarity and non-conceptuality." – p. 183

2.14.7 Luminosity, clearness, limpidity, transparency, radiance, purity

Wallace (2006a)	"The 'natural state' of the mind, according to Buddhist contemplatives, is characterized by the three qualities of bliss, luminosity, and nonconceptuality." – p. 81<br>"... [W]hen your mind is settled in its natural state, you discover for yourself the innate qualities of bliss, luminosity and stillness that have always been there." – p. 91<br>"[In the settling the mind in its natural state practice] [e]ventually you will emerge ... into a clear and luminous space of awareness." – p. 109<br>"... a state of radiant, clear consciousness ..." – p. 122<br>Quoting Padmasambhava: "Let the mind, like a cloudless sky, be clear ... and settle in utter vacuity. By doing so there arises the quiescence of joy, luminosity, and nonconceptuality." – p. 144<br>"Contemplatives who have realized the substrate consciousness through the practice of shamatha claim that it is imbued with three attributes: bliss, luminosity, and nonconceptuality." – p. 123<br>"... the sheer luminosity and cognizance of being aware. The mind has been reduced to its bare nature, a relative vacuum state." – p. 133<br>"... [Y]our mind rests in the luminous vacuity of the substrate consciousness." – p. 161<br>Quoting Düdjom Lingpa: "You will become still in an unfluctuating state, in which you will experience ... clarity like the dawn ..." – p. 161

Wallace (2011b)	"The accomplishment of shamatha is accompanied by a powerful experience of bliss, luminosity, and stillness." – p. ix<br>"When the mind is quiescent – no turbulent thoughts or emotions arising – it is relaxed, still, luminous, and free from effort. That is shamatha." – p. 62<br>"There is only one source of illumination for the entire world: consciousness, which is nondual with space. Because consciousness makes manifest, or illuminates, all the appearances that arise in the space of the mind, space itself is said to be luminous." – p. 95<br>"... limpid, clear, transparent, and luminous consciousness ..." – p. 96<br>"When looking over in the direction of Jane, had the radiant clarity of my awareness not seen colors and images arising, I wouldn't be able to bring up the designation 'body', nor 'Jane's body', let alone 'Jane'. Therefore it is not a purely conceptual process that provides us with the basic appearances themselves. Also bear in mind that the radiant clarity of awareness does not refer exclusively to the visual mode. The luminosity of awareness also manifests as smells, touch ... as well as all other phenomena. So the appearances themselves are rising from the luminosity of consciousness." – p. 98<br>"... shamatha, or quiescence – a state of bliss, luminosity, and nonconceptuality." – p. 112<br>"Three qualities that you will be able to identify distinctly – bliss, luminosity, and nonconceptuality – will manifest in you." – p. 120<br>Wallace p. 120 refers to luminosity as "the radiance, the clarity" in substrate consciousness.<br>"As you go deeper into the nature of awareness, into samadhi, your mind is drawn in as if you were falling asleep, yet it remains luminously aware. Thus everything appears to vanish by itself, just like the whole world vanishes for you when you fall asleep." – p. 153<br>Quoting Düdjom Lingpa: "... clear, limpid consciousness ..." – p. 158<br>"Now what is the substrate consciousness attending to? What is appearing to it? It's the substrate – that vacuity, an emptiness. What makes it luminous? Your own consciousness, which is empty and yet luminous ..." – pp. 176-177<br>"Here is consciousness stripped bare, down to its essential nature of luminosity and cognizance." – p. 177<br>Wallace p. 180 describes substrate consciousness as a "blissful, luminous vacuity".<br>The glossary defines luminosity as "[t]he natural clarity of awareness that makes manifest all appearances" (p. 186). For the term clarity, it simply cross-references to luminosity (p. 184).
Wallace (2009/2014)	"... space of inner purity ..." – p. 59<br>"... pure, luminous nature of your own awareness." – p. 88<br>"... [O]ne's awareness comes to rest in a naturally pure, unencumbered, luminous state known as the *bhavanga* ..." – p. 90<br>"According to many advocates of the Mahayana school of Buddhism, consciousness is characterized by two fundamental qualities: luminosity and cognizance. To get some idea of what is meant by these terms, imagine that you have been immersed in a sensory deprivation tank so efficient that you become entirely unaware of your body and physical environment ... Imagine further that all discursive thoughts, mental images, and other activities of the mind subside. Even in this state of profound inactivity, a kind of vacuity appears to your awareness, and this appearance is produced by the mind's luminous quality. In addition, there is an immediate sense of being aware, and that too is an expression of the mind's luminosity. Consciousness not only *illuminates* this vacuity and its own presence as awareness, it also *knows* that the space of the mind is empty and that there is awareness of that space. That knowing is the cognizance of consciousness, its second defining feature." – p. 90 "Consciousness alone has these two unique qualities. Without it, there are no appearances – no colours, sounds, smells, tastes, tactile sensations, or mental images such as dreams. And without it, nothing is known." – p. 90<br>"... [Substrate consciousness] is characterized by three essential traits: bliss, luminosity, and nonconceptuality ... The luminosity ... is one of the two defining characteristics of consciousness, and it is that which illuminates all the appearances to the mind." – p. 91<br>"What remains is a state of radiant, clear consciousness ..." – p. 92
Wallace (2012/2014)	"Theravadin Buddhist commentators identify [the bhavanga] with a luminous continuum of consciousness ... During the waking state, ordinary consciousness illuminates all appearances, sensory and mental, but this can be diminished or extinguished due to damage to the physical senses or the brain. However, the innate luminosity of the ground of becoming remains unchanged, even if it is obscured by mental or physical influences." – p. 102<br>"From the substrate arises a radiant, clear state of awareness, the substrate consciousness, which illuminates all sensory and mental appearances." – p. 214
Wallace (2012)	"... a radiant, clear dimension of consciousness ..." – p. 138

Wallace (2011a)	"Awareness, by nature, illuminates appearances and knows, or cognizes." – p. 54<br>"The very essence of awareness is to illuminate." – p. 177<br>"The bhavanga is the naturally pure and undefiled state of radiant mind ..." – p. 222<br>"Buddhism offers an enormously useful definition [of consciousness] that is purely phenomenological and experiential, with no metaphysical baggage ... : consciousness is that which is luminous and cognizant." – p. 228 "Only consciousness, here synonymous with awareness, illuminates phenomena and makes them manifest: this is the first distinguishing feature of consciousness ..." – p. 228 "Awareness alone illuminates all appearances of form, sound, smell, taste, tactile sensations, emotions, thoughts, memories, and desires. It illuminates every sensory object from mind to matter and every idea from algebra to ethics. These things only appear due to the luminous nature of awareness." – p. 228-229<br>"This naturally luminous mind is undefiled because all mental activities, including anger, boredom, active compassion, and so forth, have gone dormant." – pp. 231-232 "With all mental processes dormant, what remains is a naturally pure, undefiled state. The afflictions have gone quiet, though not irreversibly so. The sheer radiance of awareness of the substrate consciousness manifests because it is no longer veiled by mental activities." – p. 232<br>"With all mental processes dormant, there remains an exceptional degree of luminosity: the ground luminosity of the substrate consciousness." – p. 232<br>"In the practice of shamatha ... [w]e modulate, refine, and balance the attention to cultivate a vividness that becomes progressively clearer and sharper, in both temporal and qualitative aspects. Vividness reaches a crescendo upon attaining shamatha. Lo and behold, at the end of the tunnel, luminosity meets luminosity!" – p. 232<br>"The result of achieving shamatha is experienced in postmeditation as extraordinary clarity and luminosity, even while one is actively engaged with the world. Everyday experiences take on a bright, high-definition luster ... Commenting on this, Tsongkhapa says that having achieved shamatha, even between sessions your senses of temporal and qualitative vividness may be so acute that you feel you could count individual atoms in the walls of your house." – p. 243
Wallace and Hodel (2008)	"... luminosity (an internal radiance) ..." – p. 192
Wallace (2007b)	"The result is not a trancelike, vegetative, or comatose state. On the contrary, it is a luminous, discerningly intelligent awareness ..." – p. 45<br>"The quality of luminosity is not any kind of interior light similar to what we see with the eyes. Rather, it is an intense vigilance that has the capacity to illuminate, or make consciously manifest, anything that may arise within the space of the mind. To get some idea of what this is like, imagine being wide awake as you are immersed in a sensory deprivation tank ... Then imagine that all your thought processes involving memory and imagination are put on hold, so that you are vigilantly aware of nothing but your own experience of being conscious." – p. 46
Wallace (2005)	In the forward, the Dalai Lama says: "When we withdraw our mind from external objects ... [t]here is a kind of absence, a kind of vacuity. However, as we slowly progress and get used to it, we begin to notice an underlying clarity, a luminosity." – p. viii<br>"It is said that awareness can manifest as all kinds of sensory and mental representations. This quality of awareness to reveal itself in such forms is called its clarity, or luminosity ... It is like radiant space that illuminates everything that arises in that space, yet its radiance remains even in moments when consciousness has no discernible object." – p. 37<br>"Right in the nature of awareness itself there is luminosity and clarity ... [I]t is innate to awareness itself." – p. 39 Wallace refers to this as "this intrinsic stillness and luminosity of awareness" (p. 39).<br>"As the murkiness and perturbations of discursive thoughts calm, the space of the mind becomes increasingly transparent." – p. 165<br>"Allow your awareness to rest in your most intimate knowledge, the knowledge of being aware. Rest in this utter simplicity – aware of being aware, without an object. When you do so, you repose in luminosity itself, the clear and knowing nature of awareness." – p. 189<br>"... [C]an you discern the innate luminosity, the brightness of innate awareness that is always present? This is said to be the nature of awareness." – p. 189
Wallace (1998/2005)	In the section on Tsongkhapa's vision/understanding:<br>"... a state of exceptional mental clarity." – p. 212

Wallace (2001/2003)	"Limpidity has the dual connotation of complete transparency ... and also luminosity or brightness ..." – p. 90 "This is the defining characteristic of the natural state of awareness – it is limpid, clear and luminous and, like space itself, not the least bit sticky." – p. 91
Wallace (2000)	"... [T]he mind is ... seen as an unobscured, clear, and vivid vacuity ..." – p. 109<br>"... [O]ne of the defining characteristics of consciousness is said to be clarity, or luminosity." – p. 109<br>"... *sheer* awareness and the *sheer* clarity of experience ... are the irreducible, defining features of consciousness alone ..." – p. 110 "Clarity and awareness are said to be the salient features of consciousness in general, not only of consciousness that is withdrawn from sensory and conceptual stimuli." – p. 110
Wallace and Wilhelm (1993)	"... the simple clarity and knowing qualities of the awareness ..." – p. 117
Wallace (2001a)	Referring to the bhavanga: "This mode of awareness is said to shine in its own radiance ... and it is experienced as being primordially pure ..." – p. 212
Wallace (1999a)	"Only the aspects of the sheer awareness, clarity, and joy of the mind appear ..." – p. 182<br>"The defining characteristics of consciousness ... are first a sense of *clarity*, or implicit luminosity capable of manifesting as all manner of appearances, and secondly the quality of *cognizance*, or the event of knowing ... [B]y focusing the attention on the *sheer* clarity and the *sheer* cognizance of experience, one attends to the defining characteristics of consciousness alone, as opposed to the qualities of other *objects* of *consciousness*." – p. 183<br>"... *Samatha* ... is characterized by joy, clarity and non-conceptuality." – p. 183
Wallace (2018)	"In the tradition of Buddhism originating in India and evolving further in Tibet ... contemplatives and scholars long ago identified two defining characteristics of consciousness: *luminosity* and *cognizance*." – p. 30 "The characteristic of *luminosity* (the Tibetan word for which may also be rendered as *clarity*) has a twofold meaning. The first is that consciousness is *clear* in the sense of being insubstantial, devoid of materiality. When observed directly, consciousness displays no physical qualities whatsoever ... The second meaning is that consciousness *illuminates*, or *makes manifest*, all sensory and mental appearances. Were it not for consciousness, there would be no appearances of any kind." – pp. 30-31<br>"... [T]he mind's intrinsic luminosity emerges – or, more precisely, becomes manifest." – p. 44 Wallace p. 44 refers to the luminosity as "natural purity". It emerges upon the removal of "defilements", and the defilements "are not intrinsic to the mind itself" (p. 44).<br>"[The substrate consciousness] is lucid, radiant, and transparent; it is clear, or luminous ..." – p. 61<br>The glossary defines luminosity as "[t]he natural clarity of awareness that makes manifest all appearances" (p. 217).
Wallace (2001b)	"Consciousness is luminous in being clear cognisance or knowing." – p. 23
Wallace (1999b)	"Those who have experienced this state describe consciousness as ... being 'clear' or 'luminous', in the sense of having the capacity to give rise to all manner of appearances, and as being of the very nature of 'knowing'." – p. 444

2.14.8 Cognizance, knowledge

Wallace (2006a)	"... the sheer luminosity and cognizance of being aware. The mind has been reduced to its bare nature, a relative vacuum state." – p. 133
Wallace (2011b)	"Here is consciousness stripped bare, down to its essential nature of luminosity and cognizance." – p. 177
Wallace (2009/2014)	"According to many advocates of the Mahayana school of Buddhism, consciousness is characterized by two fundamental qualities: luminosity and cognizance. To get some idea of what is meant by these terms, imagine that you have been immersed in a sensory deprivation tank so efficient that you become entirely unaware of your body and physical environment ... Imagine further that all discursive thoughts, mental images, and other activities of the mind subside. Even in this state of profound inactivity, a kind of vacuity appears to your awareness, and this appearance is produced by the mind's luminous quality. In addition, there is an immediate sense of being aware, and that too is an expression of the mind's luminosity. Consciousness not only *illuminates* this vacuity and its own presence as awareness, it also *knows* that the space of the mind is empty and that there is awareness of that space. That knowing is the cognizance of

	consciousness, its second defining feature." – p. 90 "Consciousness alone has these two unique qualities. Without it, there are no appearances – no colours, sounds, smells, tastes, tactile sensations, or mental images such as dreams. And without it, nothing is known." – p. 90
Wallace (2011a)	"Awareness, by nature, illuminates appearances and knows, or cognizes." – p. 54<br>"As our awareness gravitates toward the substrate consciousness, we approach a superfluid state of awareness saturated by deep knowledge that is implicit rather than explicit. Moments of inspiration give rise to flows of intuition and creativity." – p. 187<br>In a discussion about the awareness of awareness practice: "Being aware is a process like knowing rather than a thing to be known. Knowing happens, and we can be aware of it." – p. 226<br>"Buddhism offers an enormously useful definition [of consciousness] that is purely phenomenological and experiential, with no metaphysical baggage ... : consciousness is that which is luminous and cognizant." – p. 228 "The second quality of consciousness or awareness is cognizance. If you gaze at the palm of your hand, for example, there will be appearances of fresh colors, lines, and shapes. In addition to these appearances arising, something else occurs: you know it's a hand. You recognize its color, shape, and position, and you can report on this knowledge." – p. 229 "The two aspects of luminance and cognizance may operate simultaneously, but they occur independently. At times you may perceive very brilliant appearances but make no sense of them." – p. 229 "On the other hand, let's suppose you have spent up to five thousand hours in the continuous practice of shamatha to the point of impeccable stability and vividness. Directing this awareness upon the luminous vacuity of your own substrate consciousness with intense interest, almost nothing appears. But you may know what you are observing with a high degree of certainty." – p. 229<br>The experience of substrate consciousness in shamatha practice "is not some kind of a trance but a mode of sheer, nonconceptual knowing with three fundamental characteristics: bliss, luminosity, and noconceptuality." – pp. 297-298
Wallace (2005)	"[Awareness] has a second quality, and that is cognizance. Awareness is imbued with a quality of knowing. not only do appearances arise to awareness, but awareness also apprehends these appearances, enabling us to report on them afterward. Images arise, for instance, in a mirror, but the mirror doesn't know they are there. In the space of the mind, on the other hand, appearances arise and we know of their presence." – p. 37<br>"The Tibetan Lama Sogyal Rinpoche has called [the] awareness of being aware 'the knowledge of knowledge'." – p. 39<br>"Allow your awareness to rest in your most intimate knowledge, the knowledge of being aware. Rest in this utter simplicity – aware of being aware, without an object. When you do so, you repose in luminosity itself, the clear and knowing nature of awareness." – p. 189<br>In the awareness of awareness practice: "You simply let your awareness settle in its own space, without reference to any object. There is now no subject/object relationship, just primordial knowing, the knowledge before knowledge of anything else, the knowledge of simply being aware." – p. 191
Wallace (2000)	"Because consciousness is experientially *aware* of ... appearances, its second defining characteristic [after clarity/luminosity] is said to be awareness, or cognizance." – p. 109<br>"... *sheer* awareness and the *sheer* clarity of experience ... are the irreducible, defining features of consciousness alone ..." – p. 110 "Clarity and awareness are said to be the salient features of consciousness in general, not only of consciousness that is withdrawn from sensory and conceptual stimuli." – p. 110
Wallace and Wilhelm (1993)	"... the simple clarity and knowing qualities of the awareness ..." – p. 117
Wallace (1999a)	"Only the aspects of the sheer awareness, clarity, and joy of the mind appear ..." – p. 182<br>"The defining characteristics of consciousness ... are first a sense of *clarity*, or implicit luminosity capable of manifesting as all manner of appearances, and secondly the quality of *cognizance*, or the event of knowing. ... [B]y focusing the attention on the *sheer* clarity and the *sheer* cognizance of experience, one attends to the defining characteristics of consciousness alone, as opposed to the qualities of other *objects* of *consciousness*." – p. 183
Wallace (2018)	"With absolutely nothing appearing to your awareness, what do you know? You still know that you are aware." – p. 4<br>"In the tradition of Buddhism originating in India and evolving further in Tibet ... contemplatives and scholars long ago identified two defining characteristics of consciousness: *luminosity* and *cognizance*."

	– p. 30 “The *cognizance* of consciousness refers to the experience of knowing and understanding the objects that appear to consciousness.” – p. 31
Wallace (2001b)	“Consciousness is luminous in being clear cognisance or knowing.” – p. 23
Wallace (1999b)	“Those who have experienced this state describe consciousness as ... being ‘clear’ or ‘luminous’, in the sense of having the capacity to give rise to all manner of appearances, and as being of the very nature of ‘knowing’.” – p. 444

2.14.9 Stillness

Wallace (2006a)	“... [W]hen your mind is settled in its natural state, you discover for yourself the innate qualities of bliss, luminosity, and stillness that have always been there.” – p. 91<br>Wallace p. 132 explains that the awareness of awareness practice is about being aware of awareness. He says, “The mind has been reduced to its bare nature, a relative vacuum state. But that emptiness is still illuminated by consciousness. And this awareness is innate, still, and vivid.” – p. 133<br>Quoting Düdjom Lingpa: “Eventually all coarse and subtle thoughts will be calmed in the empty expanse of the essential nature of your mind. You will become still in an unfluctuating state.” – p. 161
Wallace (2011b)	“The accomplishment of shamatha is accompanied by a powerful experience of bliss, luminosity, and stillness.” – p. ix<br>“When the mind is quiescent – no turbulent thoughts or emotions arising – it is relaxed, still, luminous, and free from effort. That is shamatha.” – p. 62<br>“... the sheer silence, the stillness, the lack of perturbation ...” – p. 120<br>Quoting Düdjom Lingpa: “... a remarkable sense of stillness ...” – p. 158
Wallace (2009/2014)	“... a deep stillness.” – p. 92<br>“In [the] practice of ‘not doing’, you may simply experience a deep inner stillness.” – p. 167
Wallace (2012)	The glossary defines “meditative quiescence. (Sanskrit: *shamatha*)” as “[a] group of related meditation techniques that lead to mental stillness.” – p. 161
Wallace (2007a)	“According to Buddhist tradition, the qualities of luminosity and stillness are innate to the substrate consciousness ...” – p. 59
Wallace (2005)	“Right in the nature of awareness itself there is luminosity and clarity ... [I]t is innate to awareness itself.” – p. 39 Wallace then refers to this as “this intrinsic stillness and luminosity of awareness” (p. 39).

2.14.10 Silence, quietness

Wallace (2011b)	“... the sheer silence, the stillness, the lack of perturbation ...” – p. 120<br>“... a quiet, percolating, radiating sense of serenity, joy that is very malleable.” – p. 145
Wallace (2009/2014)	“... blissful, luminous, conceptually silent state ...” – p. 93
Wallace (2011a)	“... a luminous, blissful, silent space of awareness ...” – p. 40<br>“... peaceful, luminous silence ...” – p. 111<br>“When the mind goes quiet, what remains is the substrate consciousness.” – p. 196<br>“The conceptual mind is quiet ...” – p. 208<br>“... the silent, luminous, blissful substrate consciousness.” – p. 249

2.14.11 Relaxation

Wallace (2011b)	“When the mind is quiescent – no turbulent thoughts or emotions arising – it is relaxed, still, luminous, and free from effort. That is shamatha.” – p. 62

2.14.12 Mental rest

Wallace (2006a)	"... rest in a silent, luminous awareness of awareness itself, devoid of any other content." – p. 150<br>"... [R]est ... in the [substrate consciousness] ... [A]llow [your consciousness] to remain in a state of tranquility ...[R]est in a state of consciousness free of appearances." – p. 161 "... [Y]our mind rests in the luminous vacuity of the substrate consciousness." – p. 161
Wallace (2009/2014)	In the settling the mind in its natural state practice: "As you discover the luminous, still space of awareness in which the movements of the mind occur, you will begin to discover an inner freedom and place of rest even when the storms of turbulent emotions and desires sweep through this inner domain." – p. 51<br>"...[W]ithout anything on which to meditate, rest both your body and mind in their natural state. Having nothing on which to meditate ... rest your awareness without wavering, in its own natural state ... Remain in this luminous state, resting the mind so that it is loose and free." – p. 88
Wallace (2012/2014)	"Theravadin Buddhist commentaries identify this radiant mind as the naturally pure 'ground of becoming' (Skt. *bhavanga*), the resting state of the mind ..." – p. 115
Wallace and Hodel (2008)	"... resting state of consciousness ..." – p. 195
Wallace (2007b)	"... [T]he mind comes to rest in its natural state ..." – pp. 47-48
Wallace (2005)	"... that state of fluid, restful awareness ..." – p. 42 "... restful quality of awareness ..." – p. 42<br>"It is the resting ground state of consciousness ..." – p. 89

2.14.13 Empty, contentless

Wallace (2006a)	"The 'natural state' of the mind, according to Buddhist contemplatives, is characterized by the three qualities of bliss, luminosity, and nonconceptuality." – p. 81<br>"... empty, clear substrate consciousness." – p. 122<br>"... evacuating the mind through the practice of samadhi." – p. 122<br>"Contemplatives who have realized the substrate consciousness through the practice of shamatha claim that it is imbued with three attributes: bliss, luminosity, and nonconceptuality." – p. 123<br>"The mind has been reduced to its bare nature, a relative vacuum state. But that emptiness is still illuminated by consciousness." – p. 133<br>Quoting Padmasambhava: "Let the mind, like a cloudless sky, be ... empty ... and settle it in utter vacuity. By doing so there arises the quiescence of joy, luminosity, and nonconceptuality." – p. 144<br>"... [O]nly in rare cases does shamatha practice of any kind directly yield insight into emptiness or pristine awareness. The purpose of shamatha meditation is to develop or unveil the stability and vividness of attention." – p. 146 "The achievement of shamatha does not mean that you have realized emptiness, the bedrock insight necessary for Buddhist liberation. And the realization of emptiness does not mean that you have recognized pristine awareness." – p. 148<br>"... rest in a silent, luminous awareness of awareness itself, devoid of any other content." – p. 150<br>"... [D]ivest your consciousness of all signs and thoughts ..." – p. 161 "... [*S*]*ign* refers to any object of attention that can be identified within a conceptual framework." – p. 131<br>"In this state, no 'signs' appear to the mind, or if they do on rare occasions due to a temporary lapse of mindfulness, they quickly disappear by themselves ..." – p. 161<br>"... [Y]our mind rests in the luminous vacuity of the substrate consciousness." – p. 161<br>"No appearances of your own body or anything else arise ..." – p. 161<br>Quoting Düdjom Lingpa: "Eventually all coarse and subtle thoughts will be calmed in the empty expanse of the essential nature of your mind. You will become still in an unfluctuating state, in which you will experience ... nonconceptuality like an ocean unmoved by waves." – p. 161
Wallace (2011b)	On substrate consciousness in the dying context: "... [T]he coarse mind ... become[s] utterly dormant by dissolving into the ground of ordinary mind, leaving no vestiges of imagery, personal history, or ego." – p. 15<br>"[In the substrate consciousness] the five senses are completely dormant, as are the other mental faculties. There the sense of I itself is dormant ... When you are abiding in the substrate consciousness, you've temporarily lost your mind. Your psyche is not there. Your self is not there explicitly. It has become dormant because there are no appearances." – p. 84

	"... of the substrate, the empty, luminous space of the mind." – p. 85<br>"... *shunyata*, emptiness ... is much deeper than the substrate." – p. 85<br>"... directing awareness inward ... right into the central domain of pervasive, empty space. At that point the mind and all appearances – your psyche – disappears. Your mind dissolves into the substrate consciousness, and all appearances dissolve into the substrate." – p. 97<br>"The substrate consciousness isn't really empty. It's like an ocean of 'potential energy', from which the 'kinetic energy' of the appearances of the mind emerge. Only now you have withdrawn limpid clear consciousness not only from the sense fields, but away from internal chitchat, from images – away from all of the mind's contents – such that what you experience is empty and free of appearances. At that moment your mind, your psyche, has vanished. You have lost your mind insofar as the mind is something you designate on that vast array of mental processes that characterize the mind in action. With the mind now dormant, all that remains is the ground of the mind, the substrate consciousness, that from which the mind emerges." – p. 97<br>Wallace p. 97 notes that "empty" is not to be mistaken for "emptiness".<br>"... shamatha, or quiescence – a state of bliss, luminosity, and nonconceptuality." – p. 112<br>"Three qualities that you will be able to identify distinctly – bliss, luminosity, and nonconceptuality – will manifest in you." – p. 120<br>Wallace p. 120 refers to nonconceptuality as "the sheer silence, the stillness, the lack of perturbation, the open spaciousness" of substrate consciousness.<br>"As you go deeper into the nature of awareness, into samadhi, your mind is drawn in as if you were falling asleep, yet it remains luminously aware. Thus everything appears to vanish by itself, just like the whole world vanishes for you when you fall asleep." – p. 153<br>"We normally configure consciousness with language, experience, personal identity, personal history, hopes and fears, joys and sorrows, and so on, but [at this point] [t]here is no clear-cut object of meditation ..." – p. 171<br>Quoting Düdjom Lingpa: "You then slip into the vacuity of the substrate ..." – p. 175 Wallace comments, "I've chosen the word *vacuity* rather than *emptiness* because this shouldn't be mistaken for the realization that all phenomena lack inherent nature. It's just empty, vacuous. There is nothing in it ... There are no appearances except for an occasional 'bubble'." – p. 175<br>"You can get to [the substrate] by withdrawing from the senses and from conceptualization ..." – p. 175<br>"... [W]hen you've achieved shamatha ... [y]our mind, as it were, dissolves ... into the substrate consciousness ..." – p. 176<br>"... [The] substrate consciousness ... is the luminous, vacuous ground of the ordinary mind." – p. 176<br>Wallace p. 180 describes substrate consciousness as a "blissful, luminous vacuity".<br>The glossary defines vacuity as "[a]n absence" or "an emptiness" (p. 190).
Wallace (2009/2014)	"... [T]he physical senses withdraw into mental awareness, so that one becomes oblivious to physical surroundings and even the body, and discursive thoughts and mental images also gradually dissolve into the luminous vacuity of the mind." – p. 80<br>"... [Substrate consciousness] is characterized by three essential traits: bliss, luminosity, and nonconceptuality." – p. 91 "Nonconceptuality in this context is experienced as a deep stillness. But it is not absolutely devoid of thoughts, for this dimension of consciousness is subliminally structured by concepts." – pp. 91-92
Wallace (2012/2014)	"... [In the bhavanga] awareness is withdrawn from all objects, sensory and mental." – p. 115<br>"... [A]ll appearances vanish into the substrate." – p. 219
Wallace (2011a)	"... I might practice shamatha meditation, deactivating the conceptual mind ..." – p. 69<br>"... [A]ll appearances fade into the empty vacuity of the substrate." – p. 166<br>"... [T]he bhavanga ... is the ground state when all the activities of the mind have subsided." – p. 166<br>Wallace pp. 207-209 indicates that his practice involving investigation of the space of the mind "directs mindfulness to the ground of the mind" (p. 208) (i.e., substrate consciousness). He later says: "[S]ometimes the [space of the mind] seems pregnant with potential ... This space is not an empty nothingness – it's not flat empty. Such qualifiers point to a paradox: emptiness is full." – p. 215<br>"This naturally luminous mind is undefiled because all mental activities, including anger, boredom, active compassion, and so forth, have gone dormant ..." – pp. 231-232<br>Discussing the settling the mind in its natural state practice: "The achievement of shamatha is like falling deep asleep while remaining wide awake – your only awareness is of a luminous vacuity called the space of the mind, or substrate." – p. 249

	"As we settle the mind in its natural state, the psyche dissolves into the luminous substrate consciousness, revealing a deeper dimension of mind that is free of ... conceptualizations." – p. 297<br>The experience of substrate consciousness in shamatha practice "is not some kind of a trance but a mode of sheer, nonconceptual knowing with three fundamental characteristics: bliss, luminosity, and noconceptuality." – pp. 297-298
Wallace and Hodel (2008)	"The substrate consciousness can be compared to a *relative* vacuum. It is relatively empty, but still possesses structure and energy, characterized by such attributes as bliss (spiritual joy or rapture), luminosity (an internal radiance), and a muted sense of duality between subject and object." – p. 192<br>"... withdrawn from the five physical senses and from mental activity ..." – p. 194
Wallace (2007b)	"... [V]irtually all thoughts and other mental constructs eventually become dormant." – p. 45<br>"... [T]he normal activities of the mind have subsided." – p. 45<br>Wallace conveys the experience by having the reader picture a sensory deprivation tank experience where "all [their] thought processes involving memory and imagination are put on hold" (p. 46).<br>"The mind may now be likened to a luminously transparent snow globe in which all the normally agitated particles of mental activities have come to rest." – p. 46<br>"... mental appearances naturally cease ..." – p. 47<br>"... [T]he tendency toward reification is temporarily suspended ..." – p. 101
Wallace (1998/2005)	In the section on Tsongkhapa's vision/understanding:<br>"In the state of meditative equipoise, only the aspects of awareness, clarity, and joy of the mind appear, and all one's other sense faculties remain dormant." – p. 82<br>"In this state the innate tendency of reification may have been suspended, for there is no conceptual grasping onto any appearances. However, the mere temporary suspension of discursive thought and reification is by no means equivalent to insight into the emptiness of inherent existence of the mind or any other phenomenon." – pp. 92-93<br>"... '[S]ign' corresponds roughly to the modern philosophical notion of 'conceptual construct'." – pp. 107-108 "[U]pon the attainment of quiescence, as the attention is withdrawn from all signs ... the entire continuum of attention becomes free of ideation and of signs." – p. 108<br>"... [T]he fact that the mind enters a state free of conceptualization does not necessarily mean that it fathoms ultimate truth transcending all conceptual frameworks. It is not enough that the tendency of grasping on to signs and of reifying objects is temporarily suspended during [*samadhi*] ..." – p. 117<br><br>In the section on quiescence in Mahamudra/Dzogchen:<br>"Although the state of quiescence is said to be non-conceptual, the meaning here is that the mind is not consciously engaged in discursive thought." – p. 231<br>"In the state of quiescence, the mind is no longer consciously engaged with human thought, mental imagery, or language, and it is disengaged from the human senses." – p. 232
Wallace (2000)	"[I]n this meditative state, one is said to ascertain the essential features of consciousness vividly, single-pointedly, and without conceptual mediation ... When the mind is observed free of any conceptual fluctuation, it is seen as an unobscured, clear, and vivid vacuity ..." – p. 109<br>Wallace notes that in shamatha practices contemplatives "seek to disengage from their conceptually structured experiences derived from both sensory and mental perception and to enter a state free of all subjective constructs" (p. 119).
Wallace (2001a)	"... a primal state of contentless awareness ..." – p. 212 "... contentless mental awareness ..." – p. 213
Wallace (2018)	"... [Y]our ordinary mind and all its concomitant mental processes go dormant ..." – p. 7<br>Wallace p. 61 refers to the "nonconceptuality" of substrate consciousness, "wherein thoughts have only temporarily subsided, for this is only relative nonconceptuality".
Wallace (1999b)	"Those who have experienced this state describe consciousness as 'empty' of substance ..." – p. 444

2.14.14 No thoughts

See also 2.12 ("Potential to analyze").

Wallace (2006a)	"... [In substrate consciousness] discursive thoughts become dormant ..." – p. 122

	Wallace discusses what happens if in the mindfulness of breathing practice the meditator cannot sustain attention on the counterpart sign: "Your mind will slip into the *bhavanga* ... which is a relative vacuum state of consciousness, voided of all thoughts, mental imagery, and sense perceptions." – p. 160<br>"... [D]ivest your consciousness of all signs and thoughts ..." – p. 161<br>"... with consciousness disengaged from all discursive thought and imagery ..." – p. 161<br>"... [T]he whole of your attention is focused single-pointedly, withdrawn from the physical senses, discursive thoughts, and mental imagery ..." – p. 161<br>Quoting Düdjom Lingpa: "Eventually all coarse and subtle thoughts will be calmed ... You will become still in an unfluctuating state ..." – p. 161
Wallace (2011b)	"When the mind is quiescent – no turbulent thoughts or emotions arising – it is relaxed, still, luminous, and free from effort. That is shamatha." – p. 62<br>"[In the substrate consciousness] the five senses are completely dormant, as are the other mental faculties." – p. 84<br>"... [Y]ou have withdrawn limpid clear consciousness not only from the sense fields, but away from internal chitchat, from images ..." – p. 97<br>Quoting Düdjom Lingpa's guidance on settling the mind in its natural state: "[E]ventually all coarse and subtle thoughts will be calmed in the empty expanse of [substrate consciousness]" – p. 118 Wallace comments on this passage: "... [O]ver time the coarse and subtle thoughts dissolve, dissipate ... and you settle into ... substrate consciousness." – p. 119<br>"Eventually you attain equilibrium, an equipoise wherein coarse and even subtle thoughts have vanished." – p. 170<br>"... [W]hen you've achieved shamatha, roving thoughts have all dissolved." – p. 176
Wallace (2009/2014)	"... [T]he physical senses withdraw into mental awareness, so that one becomes oblivious to physical surroundings and even the body, and discursive thoughts and mental images also gradually dissolve into the luminous vacuity of the mind." – p. 80<br>"... [a] state of profound inactivity ..." – p. 90<br>"Nonconceptuality in this context is experienced as a deep stillness. But it is not absolutely devoid of thoughts, for this dimension of consciousness is subliminally structured by concepts." – pp. 90-91
Wallace (2012/2014)	"All subtle and coarse thoughts vanish ..." – p. 190
Wallace and Hodel (2008)	"... all involuntary, discursive thoughts are pacified." – p. 188
Wallace (2007b)	"... [V]irtually all thoughts ... eventually become dormant." – p. 45<br>"All that remains is a vacuous space of awareness, devoid of thoughts and images." – p. 92
Wallace (2005)	"A luminous awareness remains, without activity ..." – p. 42
Wallace (1998/2005)	In the section on Tsongkhapa's vision/understanding:<br>"... with consciousness disengaged from all discursive thought and imagery ..." – p. 92

2.14.15 No feelings

See also 2.14.6 ("Bliss, joy, pleasantness, wellbeing, absence of discomfort") and 2.14.23 ("Possible loving-kindness").

Wallace (2011b)	On substrate consciousness in the dying context: "... [T]he derivative mental processes of feeling, discernment, memory, imagination, recognition, and so on are also withdrawn." – p. 15<br>"When the mind is quiescent – no turbulent thoughts or emotions arising – it is relaxed, still, luminous, and free from effort. That is shamatha." – p. 62<br>"[In the substrate consciousness] the five senses are completely dormant, as are the other mental faculties." – p. 84<br>"[When well on the way to shamatha] [a]t times ... you may have significant intervals where nothing manifests – no pleasant or unpleasant visions or emotions – allowing you to settle into serenity, into something very close to the substrate consciousness." – p. 155<br>"We normally configure consciousness with language, experience, personal identity, personal history, hopes and fears, joys and sorrows, and so on, but [at this point] [t]here is no clear-cut object of meditation ..." – p. 171

Wallace (2011a)	"There is no absolute demarcation between thoughts and feelings; they arise concomitantly and interdependently." – p. 163<br>"This naturally luminous mind is undefiled because all mental activities, including anger, boredom, active compassion, and so forth, have gone dormant ... When everything goes dormant, this includes wholesome mental states such as loving-kindness, generosity, and compassion. But [it also includes] craving, hostility, envy, and all the mental afflictions." – pp. 231-232

2.14.16 No sense-perceptions or body-perceptions

The entries in this section 2.14.16 below indicate, or are consistent with, the understanding that in the Shamatha goal-state the meditator has no or very limited sensory or bodily awareness. Wallace (1998/2005) includes certain comments that, taken in isolation, appear inconsistent with that understanding. He notes that Karma Chagmé, writing from a Mahamudra/Dzogchen perspective in his book *Spacious Path to Freedom*, "asserts that in the state of quiescence, the five kinds of sensory consciousness do not cease, but remain clear" (p. 231). "The Nyingma Lama Gyatrul Rinpoche explained this passage to me by asserting that whereas in flawed quiescence the senses are totally withdrawn, in flawless meditation sensory objects do *appear* to the senses, but they are not *apprehended.* I believe this interpretation is compatible with the assertion that the *signs* of sensory objects do not appear to the mind while in the state of quiescence; for those objects are not perceived *as anything*." – p. 231 Wallace pp. 262-266 discusses these ideas further, explicitly addressing the question of whether there is sensory or bodily awareness in the state of quiescence. His conclusion appears to be that there may be different experiences of, or within, the first proximate stabilization (see 2.2, "Access to the first meditative stabilization"), including an experience *with* sensory and bodily awareness and an experience without it. He indicates that Karma Chagmé's comments may be referring to the experience *with* that awareness. Wallace's descriptions of the Shamatha goal-state as set out in this section below suggest that he views that state as the experience *without* the awareness.

Wallace (2006a)	"... [In substrate consciousness] all appearances of oneself, others, one's body, and one's environment vanish." – p. 122 "... [T]he mind is drawn inward and the physical senses become dormant." – p. 122<br>Wallace discusses what happens if in the mindfulness of breathing practice the meditator cannot sustain attention on the counterpart sign: "Your mind will slip into the *bhavanga* ... which is a relative vacuum state of consciousness, voided of all thoughts, mental imagery, and sense perceptions." – p. 160<br>"... with the entirety of your awareness withdrawn from your physical senses ..." – p. 161<br>"... [T]he whole of your attention is focused single-pointedly, withdrawn from the physical senses, discursive thoughts, and mental imagery ..." – p. 161<br>"No appearances of your own body or anything else arise ..." – p. 161<br>"... [B]efore entering meditative equipoise, you can cue yourself to emerge from meditation after a designated period, or you can prepare your mind to be aroused from samadhi by a specific sound or other sensory stimulus." – p. 162
Wallace (2011b)	On substrate consciousness in the dying context: "In the dying process the senses shut down one by one. They retract. The tentacles of awareness withdraw from the five physical senses ..." – p. 15<br>"[In substrate consciousness] the five senses are completely dormant, as are the other mental faculties." – p. 84<br>"... [Y]ou have withdrawn limpid clear consciousness not only from the sense fields, but away from internal chitchat, from images ..." – p. 97<br>"As awareness is withdrawn from the sense fields, they disappear ..." – p. 97<br>Quoting Düdjom Lingpa: "You then slip into the vacuity of the substrate, in which self, others, and objects disappear." – p. 175<br>"... withdrawing from the world of the senses ... the senses shutting down ..." – p. 176
Wallace (2009/2014)	"... [T]he physical senses withdraw into mental awareness, so that one becomes oblivious to physical surroundings and even the body, and discursive thoughts and mental images also gradually dissolve into the luminous vacuity of the mind." – p. 80
Wallace (2011a)	"... [T]he senses are said to dissolve." – p. 166
Wallace (2010)	"When the mind starts to enter more deeply into the meditative object, the sense of having a body at all will fall away ..." – p. 43
Wallace and Hodel (2008)	"... appearances to the senses of oneself, one's body, and the environment vanish ..." – p. 188
Wallace (1998/2005)	In the section on Tsongkhapa's vision/understanding:

	"In the state of meditative equipoise, only the aspects of awareness, clarity, and joy of the mind appear, and all one's other sense faculties remain dormant ... [O]ne lacks any sensation of having a body ..." – p. 82
Wallace (2000)	"One has no sense of one's own body ..." – p. 108
Wallace (2001a)	"... [T]he mind is free of all physical sense impressions, including the presence of one's own body ..." – p. 212
Wallace (2018)	"... [Y]ou become oblivious to your body and environment." – p. 7

2.14.17 No mental images

Wallace (2006a)	Wallace discusses what happens if in the mindfulness of breathing practice the meditator cannot sustain attention on the counterpart sign: "Your mind will slip into the *bhavanga* ... which is a relative vacuum state of consciousness, voided of all thoughts, mental imagery, and sense perceptions." – p. 160<br>"... with consciousness disengaged from all discursive thought and imagery ..." – p. 161<br>"... [T]he whole of your attention is focused single-pointedly, withdrawn from the physical senses, discursive thoughts, and mental imagery ..." – p. 161
Wallace (2011b)	On substrate consciousness in the dying context: "... [S]ome mental imagery arises, and then that too goes ... "[T]he coarse mind ... become[s] utterly dormant by dissolving into the ground of ordinary mind, leaving no vestiges of imagery, personal history, or ego." – p. 15<br>"[In substrate consciousness] the five senses are completely dormant, as are the other mental faculties." – p. 84<br>"... [Y]ou have withdrawn limpid clear consciousness not only from the sense fields, but away from internal chitchat, from images ..." – p. 97
Wallace (2009/2014)	"... [T]he physical senses withdraw into mental awareness, so that one becomes oblivious to physical surroundings and even the body, and discursive thoughts and mental images also gradually dissolve into the luminous vacuity of the mind." – p. 80
Wallace (2007b)	"All that remains is a vacuous space of awareness, devoid of thoughts and images." – p. 92
Wallace (1998/2005)	In the section on Tsongkhapa's vision/understanding:<br>"... with consciousness disengaged from all discursive thought and imagery ..." – p. 92

2.14.18 No memories

Wallace (2006a)	"While abiding in shamatha, you may have little or no experience of the passage of time, for the sense of time requires memory, which is activated through conceptualization; so in the absence of conceptualization, you dwell in a state of consciousness that feels timeless." – p. 162
Wallace (2011b)	On substrate consciousness in the dying context: "... [T]he coarse mind ... become[s] utterly dormant by dissolving into the ground of ordinary mind, leaving no vestiges of imagery, personal history, or ego." – p. 15<br>"[In substrate consciousness] the five senses are completely dormant, as are the other mental faculties." – p. 84<br>"We normally configure consciousness with language, experience, personal identity, personal history, hopes and fears, joys and sorrows, and so on, but [at this point] [t]here is no clear-cut object of meditation ..." – p. 171
Wallace (2009/2014)	"As you settle deeper and deeper in this still, luminous state of consciousness, all memories fade away and your ordinary sense of personal identity disappears." – p. 82
Wallace (2007b)	Wallace conveys the experience by having the reader picture a sensory deprivation tank experience where "all [their] thought processes involving memory and imagination are put on hold" (p. 46).

2.14.19 Possible awareness of rhythm of breathing

Wallace (2018)	"... [M]editators who are adept at becoming lucid while still in dreamless sleep report that they are still able to mentally detect the *rhythm* of their respiration even though they are unaware of any *tactile sensations* within their body. This would imply that people who have achieved [shamatha] and are resting in the substrate consciousness may still be aware of the rhythm of their respiration, and such mindfulness of the respiration could continue even as one fully achieves the first [dhyana] ..." – p. 11

2.14.20 No grasping to the absent mental content

See also 2.10 ("Dormant/implicit conceptualization in substrate consciousness").

Wallace (2006a)	Quoting Padmasambhava: "Let the mind, like a cloudless sky, be clear, empty, and evenly devoid of grasping; and settle it in utter vacuity. By doing so there arises the quiescence of joy, luminosity, and nonconceptuality." – p. 144
Wallace (2011b)	Wallace quotes Düdjom Lingpa as saying that substrate consciousness involves "clinging to a blankness, vacuity, and luminosity" – p. 178 In commenting on that passage, Wallace refers to substrate consciousness as involving "clinging on to ... signs – clinging to the experiences of bliss, luminosity, and nonconceptuality" – p. 178 He later indicates that the substrate consciousness involves "grasping on to signs and reifying them" – p. 179 Wallace p. 177-179 makes clear that in pristine awareness (in contrast to substrate consciousness) any such clinging/grasping is released.
Wallace (2005)	"... substrate consciousness, in which grasping has temporarily subsided." – p.89
Wallace (1998/2005)	In the section on Tsongkhapa's vision/understanding: "In this state the innate tendency of reification may have been suspended, for there is no conceptual grasping onto any appearances." – p. 92
Wallace (2001/2003)	"This is the defining characteristic of the natural state of awareness – it is limpid, clear and luminous and, like space itself, not the least bit sticky." – p. 91

2.14.21 Fullness/potential

Wallace (2011b)	"The substrate consciousness isn't really empty. It's like an ocean of 'potential energy', from which the 'kinetic energy' of the appearances of the mind emerge." – p. 97
Wallace (2011a)	"[S]ometimes the [space of the mind] seems pregnant with potential ... This space is not an empty nothingness – it's not flat empty. Such qualifiers point to a paradox: emptiness is full." – p. 215

2.14.22 Calm, ease, peacefulness, absence of disturbance

Wallace states: "That which disrupts the inner equilibrium of the mind is called in Sanskrit a *klesha*, a mental affliction." (Wallace 2005, p. 56); and, similarly, "A mental affliction is a mental factor, or impulse, that disrupts the equilibrium of the mind ..." (Wallace 1998/2005, p. 48). Wallace (2005, p. 56) gives "obsession, jealousy, and anger" as examples of afflictions. He later notes that the "primary mental afflictions" in Buddhism include "variations on the affliction of craving – obsession, desire, and attachment – and varieties of aversion: hatred, aggression, anger, and hostility", as well as "ignorance and delusion" (p. 72). Across his texts, Wallace repeatedly notes that, in the shamatha goal-state, the afflictions are suppressed or go dormant. For example: "When one realizes the substrate consciousness by achieving [shamatha], mental afflictions are ... suppressed ..." (Wallace, 2007a, p. 21); and "Shamatha meditation ... allows the mental afflictions to go dormant" (Wallace 2011a, p. 69). The texts make clear that the suppression/dormancy is only complete while in the state, and reduces upon returning to everyday life.

Wallace (2006a)	"... [A]llow [your consciousness] to remain in a state of tranquility." – p. 161
Wallace (2011b)	"You may seek relief in shamatha, in the peace of that mentally dormant state." – p. 79<br>"... a quiet, percolating, radiating sense of serenity, joy that is very malleable." – p. 145<br>Quoting Düdjom Lingpa: "... soothing and gentle ..." – p. 158
Wallace (2009/2014)	"... calm, luminous, ground state." – p. 59
Wallace (2011a)	"The nature of [the] continuum of quiescence [ranging up to the formless absorptions] is ... calm, and soothing." – pp. 99-100

	"... peaceful, luminous silence ..." – p. 111
Wallace (2010)	"When [shamatha] practice is nested in a proper context, it's possible to recognize ... that our minds have an avenue to serenity and peace. And from that peace of mind, that sense of ease and contentment ..." – pp. 31-32
Wallace and Hodel (2008)	"The Dalai Lama has characterized the substrate as 'a neutral state of mind ... calm, placid or undisturbed ...'." – p. 185
Wallace (2007b)	"... [T]he mind has been brought to a deep equilibrium ..." – p. 63
Wallace (2007a)	"... [W]hen the attention is settled in this deep state of equilibrium ... one experiences a sense of inner peace and well-being." – p. 60
Wallace (2005)	"... quiescence, or simple calm ..." – p. 79<br>"... inner calm." – p. 107 "... inner sanctuary ..." – p. 107<br>"... the subtle joy of serenity, an inner calm." – p. 109
Wallace (1998/2005)	In the section on Tsongkhapa's vision/understanding:<br>Wallace p. 187 discusses how in the final stages of practice excitation and laxity have been eliminated, and effort can then be released. He then says: "The equanimity that arises due to such habituation is characterized by mental balance that is free of excitation and laxity; tranquility in which the mind rests in its own nature without compulsively grasping onto objects; natural, effortless engagement with one's meditative object; and functionality, or fitness of the attention such that it can be employed at will." – p. 187
Wallace (2001/2003)	"Quiescence is ... serene ..." – p. 135
Wallace (1999a)	"... *Samatha* is a serene attentional state in which the hindrances of excitation and laxity have been thoroughly calmed." – pp. 176-177

2.14.23 Possible loving-kindness

Wallace (2011a)	"In the Buddhist understanding, loving-kindness and compassion are not classified as feelings or emotions, although they are commonly accompanied by feelings and emotions. Loving-kindness and compassion are considered aspirations." – p. 137 "In ... substrate consciousness, no ordinary experience manifests. There is no overt sense of loving-kindness or compassion." – p. 137<br>"This naturally luminous mind is undefiled because all mental activities, including anger, boredom, active compassion, and so forth, have gone dormant ... When everything goes dormant, this includes wholesome mental states such as loving-kindness, generosity, and compassion. But [it also includes] craving, hostility, envy, and all the mental afflictions." – pp. 231-232
Wallace (2001a)	Referring to the bhavanga: "Remarkably, Buddhist contemplatives have also concluded that the nature of this ground of becoming is loving kindness ..." – pp. 212-213 "The observation that the *bhavanga* is of the nature of love would imply that empathy is innate to consciousness and exists prior to the emergence of all active mental processes." – p. 213<br>"... [T]he qualities of cognizance and loving kindness are co-existent in the ground state of awareness known as the *bhavanga* ..." – p. 217
Wallace (2018)	"The Buddha ... indicated that loving-kindness is an innate quality of the luminous mind ..." – p. 44

2.14.24 No longer mindful of meditation object

Wallace (2011b)	Wallace p. 173 discusses how Düdjom Lingpa, and Asanga, the "great fifth-century Indian Buddhist contemplative scholar", each state that "mindfulness is released after achieving shamatha". "You reach a point where the mindfulness that you were cultivating and sustaining previously becomes effortless and is released, leaving you simply present in the substrate consciousness. So you are no longer attending with mindfulness – you are no longer holding on to an object without forgetfulness." – p. 173
Wallace (2009/2014)	Wallace p. 87 describes how the breath can be used to support the awareness of awareness practice. He then says: "As your mind calms, your breathing will become more and more subtle, and when this happens,

	disengage your awareness from the breath and rest in the ongoing flow of awareness of awareness. Now direct your attention downward, gently release your mind, and without anything on which to meditate, rest both your body and mind in their natural state. Having nothing on which to meditate ... rest your awareness without wavering, in its own natural state ... just as it is. Remain in this luminous state ...” – pp. 87-88<br>Wallace p. 167 describes a form/phase of practice he describes as “nonmeditation”, which appears similar to the awareness of awareness practice (or some phase within it). “This phase of practice is sometimes called ‘nonmeditation’, for you are not meditating on anything. Simply place your awareness in the space in front of you and maintain unwavering mindfulness without taking anything as your meditative object.” – p. 167<br>“In this practice of ‘not doing’, you may simply experience a deep inner stillness.” – p. 167
Wallace (1998/2005)	In the section on Tsongkhapa’s vision/understanding:<br>“... Tsongkhapa says that as soon as quiescence is achieved, the entire continuum and flow of one’s attention should be single-pointedly focused inwards in the quiescence of the mind ... No longer does one mentally engage with the previously visualized object; rather when that object is dissolved and removed, the mind is placed in the absence of appearances.” – p. 92<br>“The actual state of quiescence ... is characterized by an absence of mindfulness and mental engagement. This absence of mindfulness does not imply that the clarity or stability of attention has been sacrificed, but only that the attention has been disengaged from its accustomed meditative object. It should be recalled that mindfulness as it has been defined in this context ... arises only with respect to a familiar object that has already been ascertained. Similarly, the absence of mental engagement does not imply here a vague, inattentive awareness, but only that the attention is no longer willfully directed to a specific object and held there.” – pp. 211-212<br>“When quiescence is finally achieved, the entire continuum of one’s attention is focused single-pointedly, non-conceptually, and internally in the very quiescence of the mind ... At that point, if occasional thoughts do arise ... the meditator is counseled not to follow after them, but to be *without mindfulness and without mental engagement*. Thus, one now disengages not only from extraneous thoughts and so forth, but even from the meditative object. For the first time in this training, one does not attempt to fix the attention upon a familiar object. One’s consciousness is now left in an absence of appearances, an experience that [Asanga] says is subtle and difficult to realize.” – p. 228
Wallace (2001/2003)	“Until this point, continuity of attention could only be sustained with effort, but once quiescence is achieved, you no longer attend to an object – you release it. You release the object you attend to.” – p. 133<br>“The great fourth-century Indian Buddhist sage Asanga stated that when quiescence is accomplished, one should relinquish mindfulness and simply be present in a state of awareness without form, without any sense of subject and object. Here awareness is drawn into itself ...” – p. 135
Wallace (2000)	“... [O]ne now disengages not only from extraneous thoughts and so forth but even from the meditative object.” – p. 107<br>“... [O]ne’s meditative object dissolves ...” – p. 108<br>Describing the “maintaining the attention in nonconceptuality” practice, which appears to be the awareness of awareness practice, or a practice that is similar: “One lets the mind come to rest like a cloudless sky, clear, luminous, and with no intentional object apart from its own presence. When the attention is sustained in that fashion, all mental engagement with other objects is stopped ...” – p. 109
Wallace (2001a)	“If one ... disengages the attention from the [counterpart sign], without relinquishing the heightened sense of attentional stability and vividness ... one experiences ... the *bhavanga* ...” – p. 212
Wallace (1999a)	“With the achievement of *Samatha*, one disengages the attention from the previous meditative object ... Thus, for the first time in this training, one does not attempt to recall a familiar object or mentally engage with it.” – p. 182

2.14.25 Attention on substrate/awareness/consciousness/quiescence/mind
See also 2.14.24 (“No longer mindful of meditation object”).

Wallace (2006a)	“It is common for contemplatives to settle for access concentration. Asanga advised that as soon as this state of shamatha is achieved, the entire continuum and flow of one’s attention should be single-pointedly focused inwardly on the mind.” – p. 161<br>“... [T]he whole of your attention is focused single-pointedly ... and is immersed in the substrate consciousness.” – p. 161

Wallace (2011b)	"... directing awareness inward ... right into the central domain of pervasive, empty space ... Your mind dissolves into the substrate consciousness, and all appearances dissolve into the substrate." – p. 97<br>"When you slip into the substrate consciousness, what you're attending to, experiencing, what's appearing to your mind, to your substrate consciousness, is the substrate, the *alaya*. The *alaya* is a vacuity ... The substrate, the *alaya*, is luminous, but empty." – p. 175<br>"Now what is the substrate consciousness attending to? What is appearing to it? It's the substrate – that vacuity, an emptiness." – p. 176
Wallace (2009/2014)	"... [With sufficient training] it is said that one may directly, vividly ascertain [the substrate] ..." – p. 94
Wallace (2011a)	"The mind is utterly controlled and settled in a state of equipoise that is nothing like a trance, in which you cannot think or function." – p. 97<br>"... [U]pon achieving shamatha, there is vivid awareness of the substrate." – p. 166<br>"... not some kind of a trance ..." – p. 297
Wallace (2007b)	"The result is not a trancelike, vegetative, or comatose state." – p. 45<br>Wallace conveys the experience by having the reader picture a sensory deprivation tank experience where "you are vigilantly aware of nothing but your own experience of being conscious" (p. 46).<br>"... [I]t is said that one may directly vividly ascertain this relative ground state of consciousness ..." – p. 47<br>"... [O]ne should be able to effortlessly remain [in quiescence] ... with unwavering mindfulness and an extraordinary degree of vividness." – p. 88
Wallace (2005)	"The [awareness of awareness practice] results in a vivid experience of the substrate consciousness ..." – p. 41<br>"... [M]editative stabilization (*dhyana*) ... is not a trancelike state ..." – p. 44
Wallace (1998/2005)	In the section on Tsongkhapa's vision/understanding:<br>"With the achievement of quiescence, the attention is drawn inwards and is maintained continuously, single-pointedly upon its object." – p. 82<br>"... Tsongkhapa says that as soon as quiescence is achieved, the entire continuum and flow of one's attention should be single-pointedly focused inwards in the quiescence of the mind ... No longer does one mentally engage with the previously visualized object; rather when that object is dissolved and removed, the mind is placed in the absence of appearances." – p. 92<br>"... [U]pon the attainment of quiescence ... the attention is withdrawn from all signs and is focused on the nature of awareness itself ..." – p. 108<br>Tsongkhapa quotes from another root text: "The entire continuum and flow of your attention [is] focused in single-pointedness and internally focused in the quiescence of the mind ... [T]he mind is placed in the absence of appearances." – p. 206 He then notes: "... [W]hen you settle in meditative equipoise, only the aspects of the sheer awareness, clarity, and vivid joy of the mind appear, without the appearance of the signs of visual form, sound, and so on." (p. 207); and "... [T]his is the way ideation vanishes: by placing the attention in the absence of mindfulness and mental engagement ... any ideation that arises cannot be prolonged in great diffusion, but naturally subsides." (p. 207). Wallace comments: "While quiescence is initially accomplished by means of mindfulness and mental engagement ... the method for purifying quiescence entails placing the attention in 'the absence of appearances'. Thus, if one has achieved quiescence by focusing on a mental image ... one would now release that image, so that 'only the aspects of just the awareness, clarity, and vivid joy of the mind appear' ... This implies that, regardless of the type of object used during the prior training, upon the achievement of quiescence, the mind is settled in the absence of appearances in which only the salient characteristics of consciousness itself remain." – p. 208<br>"The actual state of quiescence ... is characterized by an absence of mindfulness and mental engagement. This absence of mindfulness does not imply that the clarity or stability of attention has been sacrificed, but only that the attention has been disengaged from its accustomed meditative object. It should be recalled that mindfulness as it has been defined in this context ... arises only with respect to a familiar object that has already been ascertained. Similarly, the absence of mental engagement does not imply here a vague, inattentive awareness, but only that the attention is no longer willfully directed to a specific object and held there." – pp. 211-212<br>"When quiescence is finally achieved, the entire continuum of one's attention is focused single-pointedly, non-conceptually, and internally in the very quiescence of the mind ... At that point, if occasional thoughts do arise ... the meditator is counseled not to follow after them, but to be *without mindfulness and without mental engagement*. Thus, one now disengages not only from extraneous thoughts and so forth, but even from the meditative object. For the first time in this training, one does not attempt to fix the attention upon a

	familiar object. One's consciousness is now left in an absence of appearances, an experience that [Asanga] says is subtle and difficult to realize." – p. 228<br><br>In the section on quiescence in Mahamudra/Dzogchen:<br>"... [O]nce quiescence is accomplished ... the continuum of one's attention may attend to previous moments of consciousness. Due to the homogeneity of this mental continuum, the experiential effect would be that of the mind apprehending itself." – p. 232
Wallace (2000)	Wallace p. 108 makes a similar comment to Wallace (1998/2005, p. 228) above.<br>Describing the "maintaining the attention in nonconceptuality" practice, which appears to be the awareness of awareness practice, or a practice that is similar: "One lets the mind come to rest like a cloudless sky, clear, luminous, and with no intentional object apart from its own presence. When the attention is sustained in that fashion, all mental engagement with other objects is stopped, as if one had fainted or fallen asleep. The crucial difference, however, is that in this meditative state, one is said to ascertain the essential features of consciousness vividly, single-pointedly, and without conceptual mediation." – p. 109
Wallace and Wilhelm (1993)	"... meditative quiescence focused on the mind." – p. 117
Wallace (1999a)	"With the achievement of *Samatha*, one disengages the attention from the previous meditative object, and the entire continuum of one's attention is focused single-pointedly, non-conceptually, and internally in the very nature of consciousness ... One's consciousness is now left in an absence of appearances, an experience that is said to be subtle and difficult to realize" – p. 182<br>"... [B]y focusing the attention on the *sheer* clarity and the *sheer* cognizance of experience, one attends to the defining characteristics of consciousness alone, as opposed to the qualities of other *objects* of *consciousness*." – p. 183
Wallace (2018)	Wallace p. 6 says that in the settling the mind in its natural state practice, "four types of mindfulness are experienced in sequence". The first two concern the interim-states (see 1.13.4, "Practice 2 – Settling the mind in its natural state – Stages of practice"), and the second two ("*the absence of mindfulness*" and "*self-illuminating mindfulness*") concern the goal-state. "... [Y]our ordinary mind and all its concomitant mental processes go dormant: this corresponds to *the absence of mindfulness*. Bear in mind that the terms translated as 'mindfulness' in [Pali] (*sati*) [and] Sanskrit ([*smrti*]) ... primarily connote recollection, or bearing in mind. Now you're not recalling or holding anything in mind: your coarse mind has gone dormant ... But at the same time, your awareness is luminously clear. The coarse mental factor of mindfulness that allowed you to reach this state has also gone dormant; hence it is called *the absence of mindfulness*." – p. 7 "Here is a twenty-first-century analogy: When your computer downloads and installs a software upgrade, it becomes non-operational for a short time before the new software is activated. Similarly [here] the coarse mindfulness that brought you to this point has gone dormant ... but you're wide awake. This is a brief, transitional phase, and it's important not to get stuck here, for if you do so for a prolonged period, your intelligence may atrophy like an unused muscle." – p. 7 "Finally, there arises the fourth type of mindfulness: *self-illuminating mindfulness*. This occurs when you invert your awareness upon itself and the substrate consciousness illuminates and knows itself ... This subtle dimension of mental consciousness is experientially realized with the achievement of [shamatha] ..." – p. 7

2.14.26 Attentional stability

See also 1.14 ("Practice 3 – Awareness of awareness").

Wallace (2006a)	"... exceptional degrees of stability and vividness of your awareness ..." – p. 159<br>"In this state, no 'signs' appear to the mind, or if they do on rare occasions due to a temporary lapse of mindfulness, they quickly disappear by themselves ..." – p. 161
Wallace (2011b)	"... [T]he degree of effort you need to exert to practice correctly tapers off as you approach accomplishment, and then simply vanishes. You will have far greater stability and vividness than you started off with, but the effort to maintain and establish that vivid, steady apprehension vanishes of its own accord." – p. 155<br>"There are no appearances except for an occasional 'bubble'." – p. 175
Wallace (2009/2014)	In shamatha, "your mental awareness [is] highly stable and alert" (p. 92).

Wallace (2012/2014)	"... [W]hen [substrate consciousness] is accessed by way of samadhi, it is found to have three distinctive qualities: bliss, luminosity, and nonconceptuality. This stable, vivid awareness ..." – p. 149<br>"... [I]f one retains consciousness while the mind dissolves into the substrate consciousness via shamatha, one apprehends the substrate with exceptional stability and vividness." – p. 188
Wallace (2010)	"[T]he mind settles into a state of ... vivid effortless stability ..." – p. 66
Wallace and Hodel (2008)	"... the extremely stable state of meditative quiescence ..." – p. 185<br>"... [P]erfect stability and vividness of attention remain as long as we do not let our practice deteriorate." – p. 212
Wallace (1998/2005)	In the section on Tsongkhapa's vision/understanding:<br>Tsongkhapa notes: "... [T]his is the way ideation vanishes: by placing the attention in the absence of mindfulness and mental engagement ... any ideation that arises cannot be prolonged in great diffusion, but naturally subsides." – p. 207<br>"... [I]f occasional thoughts do arise ... the meditator is counseled not to follow after them, but to be *without mindfulness and without mental engagement*." – p. 228<br>"... [A]ny ideation that arises is neither sustained, nor does it proliferate; rather it vanishes of its own accord, like bubbles emerging from water." – pp. 229-230
Wallace (1989/2003)	"[Upon achieving quiescence] the mind is endowed with intense clarity and stability during meditation that can be maintained effortlessly for hours on end. Throughout this time, one's awareness is untainted by even subtle distraction or dullness." – p. 196
Wallace (2001/2003)	"... [T]he mind continues in a high state of stable vigilance." – p. 92<br>"... [W]hen you approach the achievement of quiescence, the mind is ... fearlessly calm, stable with vivid clarity. The mind flows single-pointedly like a laser." – p. 133
Wallace (2000)	"In this state, any thoughts that arise neither are sustained nor do they proliferate; rather they vanish of their own accord, like bubbles emerging from water." – p. 108
Wallace and Wilhelm (1993)	"... [I]n the Buddhist context, meditative quiescence means more than just a peaceful feeling. It is a quality of awareness that is stable and vivid, clearly focused upon its chosen object." – p. 106
Wallace (1999a)	"Any thoughts that arise are not sustained, nor do they proliferate; rather they vanish of their own accord, like bubbles emerging from water." – pp. 182-183

2.14.27 Attentional vividness

See also 1.14 ("Practice 3 – Awareness of awareness").

Wallace (2006a)	Wallace p. 132 explains that the awareness of awareness practice is about being aware of awareness. He says, "The mind has been reduced to its bare nature, a relative vacuum state. But that emptiness is still illuminated by consciousness. And this awareness is innate, still, and vivid. The qualities of attention you have earlier been developing through the practices of shamatha are already implicit in the nature of awareness itself. They have just been waiting to be unveiled." – p. 133<br>"... exceptional degrees of stability and vividness of your awareness ..." – p. 159
Wallace (2011b)	"... [T]he degree of effort you need to exert to practice correctly tapers off as you approach accomplishment, and then simply vanishes. You will have far greater stability and vividness than you started off with, but the effort to maintain and establish that vivid, steady apprehension vanishes of its own accord." – p. 155
Wallace (2012/2014)	"Under normal circumstances, one generally has no clear recognition of this relative ground state of awareness, but it can be vividly apprehended with the meditative achievement of highly focused, stable attention, or *samadhi* ..." – p. 115<br>"... [W]hen [substrate consciousness] is accessed by way of samadhi, it is found to have three distinctive qualities: bliss, luminosity, and nonconceptuality. This stable, vivid awareness ..." – p. 149<br>"... [I]f one retains consciousness while the mind dissolves into the substrate consciousness via shamatha, one apprehends the substrate with exceptional stability and vividness." – p. 188

Wallace (2011a)	“When you access the substrate consciousness clearly and vividly by way of shamatha, it’s like falling deep asleep while remaining luminously awake.” – p. 109<br>“... [U]pon achieving shamatha, there is vivid awareness of the substrate.” – p. 166<br>“In the Theravadin tradition, the bhavanga is the mind’s naturally pure state, but it is normally inaccessible and manifests primarily during deep sleep ... [H]owever, in falling asleep we generally lose clarity, and the ground state is veiled by dullness and sleepiness.” – p. 232 “In stark contrast to this is access to the bhavanga via shamatha, which cultivates increasing luminosity and vividness of attention.” – p. 232 “In the practice of shamatha ... [w]e modulate, refine, and balance the attention to cultivate a vividness that becomes progressively clearer and sharper, in both temporal and qualitative aspects. Vividness reaches a crescendo upon attaining shamatha. Lo and behold, at the end of the tunnel, luminosity meets luminosity!” – p. 232<br>“The result of achieving shamatha is experienced in postmeditation as extraordinary clarity and luminosity, even while one is actively engaged with the world. Everyday experiences take on a bright, high-definition luster ... Commenting on this, Tsongkhapa says that having achieved shamatha, even between sessions your senses of temporal and qualitative vividness may be so acute that you feel you could count individual atoms in the walls of your house.” – p. 243
Wallace (2010)	“... [T]he mind settles into a state of ... vivid effortless stability ...” – p. 66
Wallace and Hodel (2008)	“... [P]erfect stability and vividness of attention remain as long as we do not let our practice deteriorate.” – p. 212
Wallace (2007b)	“... an extraordinary degree of vividness.” – p. 88
Wallace (2007a)	“... [O]ne lucidly penetrates to the substrate consciousness by means of [shamatha] ... [Shamatha] entails vivid awareness of this dimension of consciousness, in contrast to the dullness that normally characterizes dreamless sleep.” – p. 18
Wallace (2005)	“The [awareness of awareness practice] results in a vivid experience of the substrate consciousness ...” – p. 41
Wallace (1989/2003)	“[Upon achieving quiescence] the mind is endowed with intense clarity and stability during meditation that can be maintained effortlessly for hours on end. Throughout this time, one’s awareness is untainted by even subtle distraction or dullness.” – p. 196
Wallace (2001/2003)	“[W]hen you approach the achievement of quiescence, the mind is ... fearlessly calm, stable with vivid clarity. The mind flows single-pointedly like a laser.” – p. 133<br>“In this state of quiescence, the mind is vivid and clear.” – p. 135
Wallace (2000)	“... [T]he mind is ... seen as an unobscured, clear, and vivid vacuity ...” – p. 109
Wallace and Wilhelm (1993)	“... [I]n the Buddhist context, meditative quiescence means more than just a peaceful feeling. It is a quality of awareness that is stable and vivid, clearly focused upon its chosen object.” – p. 106

2.14.28 Attentional balance

See also the references to equilibrium in 2.14.22 (“Calm, ease, peacefulness, absence of disturbance”).

Wallace (2012/2014)	“The result of [shamatha] training is an atypical state of attentional balance, in which a high level of attentional arousal is maintained simultaneously with deep calm and relaxation. For this reason, it is called ‘meditative quiescence’ (Skt. *shamatha*). The mind is now free of both attentional laxity ... and excitation ...” – pp. 166-167
Wallace (2007b)	Referring to quiescence: “... [T]his exceptional level of attentional balance ...” – p. 88
Wallace (2007a)	“... exceptional degree of attentional balance ...” – p. 16
Wallace (1998/2005)	In the section on Tsongkhapa’s vision/understanding:<br>“... [T]he cultivation of quiescence ... may be viewed as a way to eliminate attentional excitation and laxity.” – p. 183

2.14.29 Effortlessness, relinquishment of control, non-doing
See also 1.10 ("Non-doing, effortlessness, relinquishment of control, loss of ego").

Wallace (2011b)	"When the mind is quiescent – no turbulent thoughts or emotions arising – it is relaxed, still, luminous, and free from effort. That is shamatha." – p. 62<br>"In [the settling the mind in its natural state] practice the greatest effort comes at the beginning. In the final stages, it's just effortless ... [I]n the course of the practice of shamatha, whatever technique you are following, the degree of effort you need to exert to practice correctly tapers off as you approach accomplishment, and then simply vanishes. You will have far greater stability and vividness than you started off with, but the effort to maintain and establish that vivid, steady apprehension vanishes of its own accord ... You arrive at a point in the practice where, if you keep on exerting effort, keep on applying introspection to try to fix something, you're actually cluttering up your practice." – p. 155<br>Wallace pp. 177-179 discusses how, once shamatha has been achieved, the meditator may be able to "transcend shamatha and the substrate consciousness, breaking through to pristine awareness" (p. 179). He quotes Düdjom Lingpa as saying that when this happens: "... [T]he rope of inner mindfulness and firmly maintained attention is cut." – p. 178 Wallace comments, "So there is no rope attaching you either to the substrate or to any sign, and no firmly maintained attention, no effortful striving with diligence." – p. 178
Wallace (2012/2014)	"There are many practices in Tibetan Buddhism that are said to result in the realization of the 'nature of mind'." – p. 193 Wallace p. 193 explains that these include shamatha practice, and that they culminate in realizing pristine awareness. "Mindfulness plays a crucial role along this entire path, until it has finally served its ultimate purpose and the rope of mindfulness is cut." – p. 193
Wallace (2011a)	In the settling the mind in its natural state practice: "The psyche gradually settles down toward the substrate consciousness. This might be called a superfluid state because it has no internal resistance – it is nonconceptual, luminous, and blissful." – p. 187<br>"The culmination of the path of shamatha is the effortless maintenance of stability and vividness." – p. 273
Wallace (2010)	"... [T]he mind settles into a state of ... vivid effortless stability ..." – p. 66

2.14.30 Natural state of the mind
See also 2.14.3 ("Consciousness/awareness itself").

Wallace frequently uses the term "natural state of the mind" (or similar) to refer to substrate consciousness (see examples below). However, in cases he also appears to use it to describe the state attained by the meditator from their best attempt at achieving substrate consciousness. For example, in his instructions for one of the meditation practices set out in Wallace (2009/2014), he says, "As always, begin your meditation session by settling your body, speech, and mind in their natural states" (p. 167). Since he regards substrate consciousness as difficult for people in the modern world to achieve (see 1.23, "Time/effort taken to progress, and factors supporting progress"), it is clear that he is not requiring that the meditator commences their practice by achieving substrate consciousness. Note also that sometimes the context makes clear that the instruction to settle or rest the mind in its natural state means undertaking the settling the mind in its natural state practice. For example, Wallace and Hodel (2008) state: "In [the fifth attentional stage] we may also be instructed to 'rest the mind in the natural state'. Here, rather than resisting the intrusion of mental phenomena ... we allow [them] to arise and pass without any interference." – p. 210

Wallace (2006a)	"The 'natural state' of the mind, according to Buddhist contemplatives, is characterized by the three qualities of bliss, luminosity and nonconceptuality." – p. 81<br>"... [W]hen your mind is settled in its natural state, you discover for yourself the innate qualities of bliss, luminosity, and stillness that have always been there." – p. 91<br>Wallace p. 132 explains that the awareness of awareness practice is about being aware of awareness. He says, "The mind has been reduced to its bare nature, a relative vacuum state. But that emptiness is still illuminated by consciousness. And this awareness is innate, still, and vivid." – p. 133
Wallace (2011b)	"Here is consciousness stripped bare, down to its essential nature of luminosity and cognizance." – p. 177<br>The glossary defines luminosity as "[t]he natural clarity of awareness that makes manifest all appearances" (p. 186).
Wallace (2012/2014)	"... the innate luminosity of the ground of becoming ..." – p. 102<br>Wallace p. 215 treats substrate consciousness and the natural state of the mind as equivalent.

Wallace (2011a)	"... [Y]our mind dissolves into the substrate consciousness ... This is settling the mind in its natural state – the relative ground state." – p. 166<br>In the settling the mind in its natural state practice: "... [T]he mind is settled without being suppressed, manipulated, or contrived; and it naturally dissolves into its ground state." – p. 167<br>"... the innate luminosity of awareness ..." – p. 222<br>"... [T]he bhavanga ... is naturally luminous. The bhavanga is the naturally pure and undefiled state of radiant mind ..." – p. 222<br>"... a naturally pure, undefiled state ... The sheer radiance of awareness of the substrate consciousness manifests because it is no longer veiled by mental activities." – p. 232<br>"The bhavanga is known as the natural, unconfigured state of mind ..." – p. 233
Wallace (2007a)	"... the innate luminosity of this substrate consciousness." – p. 16<br>"... imbued with an innate quality of bliss." – p. 60
Wallace (2005)	"... the innate stillness, stability, and vividness of awareness itself." – p. 40<br>Wallace p. 43 refers to the awareness of awareness practice as "[t]he practice of releasing into the natural stability and vividness of awareness ..." – p. 43<br>"This is the natural, unencumbered state of mind ..." – p. 89<br>"... [C]an you discern an ongoing stillness? If so, this is called the innate state of meditative quiescence itself, not to be achieved, already present." – p. 189<br>"... [C]an you discern the innate luminosity, the brightness of innate awareness that is always present? This is said to be the nature of awareness." – p. 189

2.14.31 Ground state

See also 2.6 ("Distinction between substrate consciousness and pristine awareness").

Wallace (2006a)	"The substrate consciousness may be characterized as the relative ground state of the individual mind, in the sense that it entails the lowest state of activity, with the highest potential and degree of freedom that can be achieved by evacuating the mind through the practice of samadhi." – p. 122 "By fathoming the nature of the substrate consciousness, one comes to know the nature of consciousness in its relative ground state." – p. 123<br>"... [S]ubstrate consciousness ... can be viewed as the relative ground state of the mind ..." – p. 137<br>"... relative ground state of consciousness ..." – p. 138
Wallace (2011b)	"... the ground of ordinary mind ..." – p. 15<br>Wallace pp. 14-15, 152, 176 uses the same expression.<br>"... substrate consciousness: the ground out of which the psyche and all of the ordinary manifestations of the mind emerge and into which they dissolve." – p. 22<br>"What is the ground of your psyche? It's the substrate consciousness ..." – p. 95<br>"... ground of the mind ..." – p. 97<br>"So now you know what the mind is in a pretty deep sense, because [substrate consciousness] is not just your psyche. It is the ground from which your psyche emerges." – p. 177
Wallace (2009/2014)	"... calm, luminous, ground state." – p. 59
Wallace (2011a)	"... [T]he bhavanga ... is the ground state when all the activities of the mind have subsided." – p. 166<br>"The bhavanga is known as the natural, unconfigured state of mind, the ground state or substrate consciousness ..." – p. 233
Wallace (2018)	"... ground consciousness." – p. 62

2.14.32 Loss of ego

See also 1.10 ("Non-doing, effortlessness, relinquishment of control, loss of ego").

Wallace (2006a)	Discussing substrate consciousness attained via the awareness of awareness practice: "The illusory, independent ego is temporarily put out of work ..." – p. 137
Wallace (2011b)	On substrate consciousness in the dying context: "... [T]he coarse mind ... become[s] utterly dormant by dissolving into the ground of ordinary mind, leaving no vestiges of imagery, personal history, or ego." – p. 15

	"When you enter [substrate consciousness], the ego has become dormant." – p. 27<br>"[In the substrate consciousness] the sense of I itself is dormant ... When you are abiding in the substrate consciousness, you've temporarily lost your mind. Your psyche is not there. Your self is not there explicitly. It has become dormant because there are no appearances." – p. 84<br>"[In the substrate consciousness] [t]here is no 'I' ..." – p. 85<br>"At that point the mind and all appearances – your psyche – disappears. Your mind dissolves into the substrate consciousness, and all appearances dissolve into the substrate." – p. 97<br>"... [Y]our mind, your psyche, has vanished. You have lost your mind insofar as the mind is something you designate on that vast array of mental processes that characterize the mind in action." – p. 97<br>"We normally configure consciousness with language, experience, personal identity, personal history, hopes and fears, joys and sorrows, and so on, but [at this point] [t]here is no clear-cut object of meditation ..." – p. 171 Quoting Düdjom Lingpa: "You then slip into the vacuity of the substrate, in which self, others, and objects disappear." – p. 175
Wallace (2009/2014)	"As you settle deeper and deeper in this still, luminous state of consciousness, all memories fade away and your ordinary sense of personal identity disappears." – p. 82<br>"All that has been lost is your conceptually constructed sense of your own self. This is an artificial construct. As your mind settles in its natural state, the sense of 'I am' is gradually dismantled." – p. 82<br>"... relative absence of thoughts of 'I' and 'not I' ..." – p. 94
Wallace (2011a)	"Your psyche can withdraw into the bhavanga, and you will have lost your identity as male or female, old or young, fat or skinny; all your personal history will be dormant." – p. 233<br>"As we settle the mind in its natural state, the psyche dissolves into the luminous substrate consciousness, revealing a deeper dimension of mind that is free of ... personal identity, and conceptualizations. This unconfigured state of consciousness is not conditioned by ... individual experience." – p. 297
Wallace (2005)	"Ego-driven, or I-centered, activity is flatlined as much as possible in this kind of meditation ..." – p. 42 The context for this comment indicates that it is referring to what happens in substrate consciousness, and that "kind of meditation" refers to shamatha practice generally.<br>"... this restful quality of awareness that is not ego-driven." – p. 42<br>"... [G]rasping onto 'I' and 'mine' vanishes ..." – p. 89

2.14.33 Simplicity

Wallace (2011a)	For the awareness of awareness practice, Wallace instructs: "[C]enter your awareness where it always was ... Rest in utter simplicity, sustaining the flow of sheer luminosity and cognizance of awareness." – p. 226
Wallace (2005)	"Rest in this utter simplicity – aware of being aware, without an object. When you do so, you repose in luminosity itself, the clear and knowing nature of awareness." – p. 189

2.14.34 Wholeness, coherence, unity

Wallace (2011b)	"... [Y]ou have come to wholeness, to coherence – you have arrived at *naturally settled mindfulness*. The anchor has struck bottom." – p. 158
Wallace (2011a)	"The conceptual mind is quiet, so there is no sense of separation between awareness and its object. This is the experience of the unity of the substrate and consciousness." – p. 208

2.14.35 Inner security, safety

See also 2.14.6 ("Bliss, joy, pleasantness, wellbeing, absence of discomfort") and 2.14.22 ("Calm, ease, peacefulness, absence of disturbance").

Wallace (2012/2014)	Quoting "treasure revealer (Tib. *tertön*)" (p. 217) Lerab Lingpa: "Due to maintaining the mind in its natural state, there may arise ... a nonconceptual sense that nothing can harm the mind ..." – p. 218

2.14.36 Inner freedom

Wallace (2006a)	"The substrate consciousness can be characterized as the relative ground state of the individual mind, in the sense that it entails the lowest state of activity, with the highest potential and degree of freedom that can be achieved by evacuating the mind through the practice of samadhi." – p. 122
Wallace (2009/2014)	In the settling the mind in its natural state practice: "As you discover the luminous, still space of awareness in which the movements of the mind occur, you will begin to discover an inner freedom and place of rest even when the storms of turbulent emotions and desires sweep through this inner domain." – p. 51<br>"Having nothing on which to meditate ... rest your awareness without wavering, in its own natural state ... Remain in this luminous state, resting the mind so that it is loose and free." – p. 88<br>"... [O]ne's awareness comes to rest in a naturally pure, unencumbered, luminous state known as the *bhavanga* ..." – p. 90
Wallace (2007a)	"The substrate consciousness may be characterized as the relative ground state of the individual mind in the sense that within the context of an individual mind stream, it entails the lowest possible state of activity, with the highest possible potential and degree of freedom or possibility." – p. 18
Wallace (2005)	"This is the natural, unencumbered state of mind ..." – p. 89

2.14.37 Timelessness

Wallace (2006a)	"Your mind has become so still and divorced from discursive thoughts that you feel you could remain in meditation uninterruptedly for months or even years, with no awareness of the passage of time." – p. 161<br>"While abiding in shamatha, you may have little or no experience of the passage of time, for the sense of time requires memory, which is activated through conceptualization; so in the absence of conceptualization, you dwell in a state of consciousness that feels timeless. Nevertheless, before entering meditative equipoise, you can cue yourself to emerge from meditation after a designated period, or you can prepare your mind to be aroused from samadhi by a specific sound or other sensory stimulus." – p. 162
Wallace (2011a)	"... [T]imelessness can be experienced in samadhi. From the perspective of one who enters the deep states of absorption in the form and formless realms, which had been thoroughly explored before the Buddha, time has vanished. It vanishes due to suppressing all conceptual processes along with the mental afflictions. The deeper the samadhi, the more timeless one's perspective, and ultimately, time is completely stopped." – p. 292

2.14.38 Spaciousness

Wallace (2011b)	"... the sheer silence, the stillness, the lack of perturbation, the open spaciousness ..." – p. 120
Wallace (2011a)	"... the luminous, boundless space of the substrate ..." – p. 306
Wallace (2001/2003)	"Quiescence is spacious ... without form and substance." – p. 135
Wallace (2000)	"... [I]t seems as if one's mind has become indivisible with space." – p. 108

2.14.39 Possible spiritual aspect

In various places in his texts, Wallace refers to the shamatha practices, or the broader suite of contemplative practices that includes them, as involving some spiritual aspect. For example, Wallace (2005) refers to the path that one takes in following the Dharma as involving "spiritual practice" and, if it goes as intended, "spiritual progress" (p. 19).

Wallace (2011b)	"Having arrived [at, or close to, substrate consciousness] you may think ... This is so transcendental and holy that I doubt that anyone's ever experienced it before." – p. 159
Wallace (2009/2014)	"... [T]he mind gradually settles in its own calm, luminous ground state. If I regard that space of inner purity to be outside myself, or to belong to God, then the benefits from this practice can be attributed to grace. But if I view that space as a deeper dimension of my own being, then I don't need to look outside myself for liberation." – p. 59

2.14.40 Possible energy

Wallace (2006a)	Describing the transition into the Shamatha goal-state: "Following [the] sense of pressure on the top of your head, you experience the movement of vital energies moving in your body, and when they have coursed everywhere throughout your body, you feel as if you were filled with the power of this dynamic energy ... Both your body and mind are now imbued with an exceptional degree of pliancy ..." – p. 156 "When physical pliancy initially arises, the vital energies catalyze an extraordinary sense of physical bliss, which then triggers an equally exceptional experience of mental bliss. This rush of physical and mental rapture is transient, which is a good thing, for it so captivates the attention that you can do little else except enjoy it. Gradually it subsides and you are freed from the turbulence caused by this intense joy. Your attention settles down in perfect stability and vividness. You have now achieved shamatha." – p. 156
Wallace (2011b)	"The substrate consciousness isn't really empty. It's like an ocean of 'potential energy', from which the 'kinetic energy' of the appearances of the mind emerge." – p. 97
Wallace and Hodel (2008)	"The substrate consciousness can be compared to a *relative* vacuum. It is relatively empty, but still possesses structure and energy, characterized by such attributes as bliss (spiritual joy or rapture), luminosity (an internal radiance), and a muted sense of duality between subject and object." – p. 192
Wallace (2004)	Describing the transition into the Shamatha goal-state: "Vital energies that result in unprecedented physical pliancy then pervasively course through the entire body, filling it with the power of this dynamic energy. A sense of bliss then saturates the body, which in turn, triggers an experience of mental bliss. This gradually subsides, leaving both the body and mind balanced and supple." – p. 21

2.14.41 Profound, deep

See also 1.11 ("The ten stages").

Wallace (2011b)	"...[A]chieving shamatha is a faint facsimile of attaining enlightenment." – p. 121<br>"The fact that you could so easily mistake shamatha for enlightenment or the highest stage of Dzogchen is understandable." – p. 156 Wallace p. 156 refers to achieving shamatha as a "very significant ... accomplishment".<br>"... Having arrived [at, or close to, substrate consciousness] you may think, 'I've hit the bonanza – this is dharmakaya! This is so transcendental and holy that I doubt that anyone's ever experienced it before. If I told anybody else about it, they would never be able to understand it ...'." – p. 159 "In reaching this level you have arrived at a remarkable place, but if you cling to it and believe this is ultimate, then you will stray far from the path." – p. 159 Wallace discusses how the caution that "You will very, very easily think you're enlightened" is found in Düdjom Lingpa's the *Vajra Essence*, and "[t]his caveat is echoed in the teachings of Tsongkhapa, Karma Chagmé Rinpoche, and many other great lamas." (p. 160).<br>"So now you know what the mind is in a pretty deep sense, because [substrate consciousness] is not just your psyche. It is the ground from which your psyche emerges." – p. 177
Wallace (2009/2014)	"As you settle deeper and deeper in this still, luminous state of consciousness, all memories fade away and your ordinary sense of personal identity disappears." – p. 82<br>"... a deep stillness." – p. 92<br>"In [the] practice of 'not doing', you may simply experience a deep inner stillness." – p. 167
Wallace (2011a)	"You are resting in a state that can easily be mistaken for buddha nature, pristine awareness, Atman, Brahman, ultimate reality, or God consciousness. Nevertheless, this is merely a quiescent mind resting in its own luminous, blissful, silent, relative ground state." – p. 40
Wallace and Hodel (2008)	"So powerful and liberating is the attainment of shamatha that it is sometimes mistaken for enlightenment." – p. 213<br>"Shamatha ... can lead to extraordinary mental and even physical states, experiences so powerful that one may be deceived into believing that full enlightenment has been attained." – p. 224
Wallace (2007b)	"... [T]he mind has been brought to a deep equilibrium ..." – p. 63
Wallace (2005)	"... [I]t is very easy to mistake [substrate consciousness] for the ultimate nature of consciousness ..." – p. 41 Wallace pp. 77, 198 makes similar comments.

Wallace (1989/2003)	"... [The] refinement of awareness [that occurs in shamatha practice] ... entails a major transformation of consciousness." – p. 150
Wallace (1999b)	"Buddhist contemplatives from various traditions hasten to add ... that this state can easily be confused with the attainment of nirvana, which it emphatically is not ... It simply provides one with direct insight into the phenomenological nature of consciousness, which is knowledge of considerable importance, but not an insight that enables one to accomplish the ultimate aim of Buddhist practice." – pp. 444-445

2.14.42 Non-changing

Wallace (2006a)	Quoting Düdjom Lingpa: "You will become still in an unfluctuating state ..." – p. 161
Wallace (2000)	"... free of any conceptual fluctuation ..." – p. 109

2.14.43 Subjective experience possibly varies between meditators

Wallace generally presents the Shamatha goal-state as a single state. An important feature of the experience is that it does not involve explicit concepts in the form of thoughts, images, memories, and so on (see, e.g., 2.14.13, "Empty, contentless"). However, Wallace makes clear that there remains what he describes as dormant or implicit conceptual structuring (see 2.10, "Dormant/implicit conceptualization in substrate consciousness"). In one text, he indicates that differences in that structuring between meditators can lead to differences in their experiences (Wallace, 2000, p. 118; see 2.10), but he does not give examples of those differences.

2.14.44 Difficulty communicating the experience to those who have not had it
See also 2.1.1 ("Terms used to describe features of the goal-state").

Wallace (2011b)	Wallace compares attaining substrate consciousness to accessing pristine awareness, or rigpa: "To be withdrawn from all appearances and simply dwell in a blank vacuity is relatively easy. You can even imagine it ... Rigpa, however, is inconceivable and ineffable." – p. 179
Wallace (2007b)	"Tibetan contemplatives claim that a shared, highly specialized language concerning rarified subjective experience has been developing within a community of professionally trained observers of the mind. Throughout such training, participants converse among themselves and with their mentors and in this way learn to communicate their inner experiences. Nonparticipants overhearing such communication may believe they understand the kinds of experiences being narrated, but in fact most of what is said will be beyond their imagination, for they have never experienced the states of consciousness that are being probed." – p. 44
Wallace (2007a)	"While some contemplative experiences or realizations are said to be ineffable, many can be described to others, especially to those who engage in similar practices. For example, a group of meditators who devote themselves to sustained, rigorous training of focused attention are able to communicate many of their experiences with one another in ways that might be relatively unintelligible to people who have never meditated." – p. 79<br>"It must be emphasized here that the great majority of discoveries made by Buddhist contemplatives are not of the ineffable, inconceivable kind that is said to be typical of mystical experience. This fact allows for a broad spectrum of meaningful discourse among experienced contemplatives, even though the discourse may be unintelligible to those who lack such meditative training and experience." – p. 82
Wallace (2000)	"Tibetan contemplatives ... declare that theories about the nature of consciousness ... are ... artificial, conceptual constructions; for the experience of consciousness when the mind is settled in meditative stabilization is a state in which words and concepts are suspended. Any subsequent theory is nothing more than a conceptual overlay on an experience that is nonconceptual. The point of such theories, however, is to break down conceptual barriers to entering into this experience and to make such realization somewhat intelligible to those who lack it. But no description or explanation can capture this experience in words or concepts or convey the actual nature of the experience to noncontemplatives." – p. 110
Wallace (2018)	"For someone whose entire experience has been of the ordinary, coarse mind, the substrate consciousness is beyond the range of conscious experience. Talking about it would be like discussing chocolate without ever having tasted it ... This inconceivability is relative to one's experience. Similarly, the attainment of [shamatha] ... is inconceivable to anyone who lacks this experience; there is no way to articulate it so that another person might know what this state is like. Even with a great eloquence and a vast vocabulary,

someone who has experienced it can only convey a vague facsimile in words to someone who has not. For them, it's ineffable." – p. 57 "Bear in mind that when resting in the flow of the substrate consciousness, one of its three fundamental qualities is nonconceptuality. Even if you have a direct, perceptual taste of the substrate consciousness, merely attempting to describe it in words takes you a step away from the experience itself; it's inconceivable even for you." – p. 58

Note. Version 1.0. There is a degree of overlap or interrelationship between the aspects of the technique, interim-states and goal-state described in the different sections of the table. For a proper understanding of each aspect, the relevant section/s should therefore be considered in the context of the table as a whole. To assist in that regard, where a particular section is closely linked to other sections, cross-references have been provided. The headings for each section indicate its subject matter or theme, and aim to capture the main thrust or gist of its content. They do not necessarily capture aspects outside that main thrust or gist, such as statements of a marginal and/or qualifying nature.

References

Kozasa, E. H., Lacerda, S. S., Menezes, C., Wallace, B. A., Radvany, J., Mello, L. E., et al. (2015). Effects of a 9-day shamatha Buddhist meditation retreat on attention, mindfulness and self-compassion in participants with a broad range of meditation experience. *Mindfulness* 6, 1235–1241. doi:10.1007/s12671-015-0385-8.

Wallace, B. A. (1999a). The Buddhist tradition of Samatha: Methods for refining and examining consciousness. *Journal of Consciousness Studies* 6, 175–187.

Wallace, B. A. (1999b). "Training the attention and exploring consciousness in Tibetan Buddhism," in *Toward a science of consciousness III: The third Tucson discussions and debates*, eds. S. R. Hameroff, A. W. Kaszniak, and D. J. Chalmers (Cambridge, MA: MIT Press), 441–448.

Wallace, B. A. (2000). *The taboo of subjectivity. Toward a new science of consciousness. Oxford*, United Kingdom: Oxford University Press.

Wallace, B. A. (2001a). Intersubjectivity in Indo-Tibetan Buddhism. *Journal of Consciousness Studies* 8, 209–230.

Wallace, B. A. (2001b). The potential of emptiness: Vacuum states in physics and consciousness. *The Scientific and Medical Network Review* 77, 21–25.

Wallace, B. A. (2001/2003). *Buddhism with an attitude: The Tibetan seven-point mind-training*. Ithaca, NY: Snow Lion.

Wallace, B. A. (1989/2003). *Choosing reality: A Buddhist view of physics and the mind*. Ithaca, NY: Snow Lion.

Wallace, B. A. (2004). Scientific and Buddhist views of energy. *Subtle Energies & Energy Medicine* 15, 17–25.

Wallace, B. A. (1998/2005). *Balancing the mind: A Tibetan Buddhist approach to refining attention*. Boston, MA: Shambhala.

Wallace, B. A. (2005). *Genuine happiness: Meditation as the path to fulfillment*. Hoboken, NJ: John Wiley.

Wallace, B. A. (2006a). *The attention revolution: Unlocking the power of the focused mind*. Somerville, MA: Wisdom.

Wallace, B. A. (2006b). “Vacuum states of consciousness: A Tibetan Buddhist view,” in *Buddhist thought and applied psychological research: Transcending the boundaries*, eds. D. K. Nauriyal, M. S. Drummond, and Y. B. Lal (Oxon, United Kingdom: Routledge), 112–121.

Wallace, B. A. (2007a). *Contemplative science: Where Buddhism and neuroscience converge*. New York, NY: Columbia University Press.

Wallace, B. A. (2007b). *Hidden dimensions: The unification of physics and consciousness*. New York, NY: Columbia University Press.

Wallace, B. A. (2010). *The four immeasurables: Practices to open the heart*. 3rd ed. Ithaca, NY: Snow Lion.

Wallace, B. A. (2011a). *Minding closely: The four applications of mindfulness*. Ithaca, NY: Snow Lion.

Wallace, B. A. (2011b). *Stilling the mind: Shamatha teachings from Düdjom Lingpa's Vajra Essence*. Somerville, MA: Wisdom.

Wallace, B. A. (2012). *Dreaming yourself awake: Lucid dreaming and Tibetan dream yoga for insight and transformation*. Boston, MA: Shambhala.

Wallace, B. A. (2012/2014). *Meditations of a Buddhist skeptic: A manifesto for the mind sciences and contemplative practice*. New York, NY: Columbia University Press.

Wallace, B. A. (2009/2014). *Mind in the balance: Meditation in science, Buddhism, and Christianity*. New York, NY: Columbia University Press.

Wallace, B. A. (2018). *Fathoming the mind: Inquiry and insight in Düdjom Lingpa's Vajra Essence*. Somerville, MA: Wisdom.

Wallace, B. A., and Hodel, B. (2008). *Embracing mind: The common ground of science & spirituality*. Boston, MA: Shambhala.

Wallace, B. A., and Wilhelm, S. (1993). *Tibetan Buddhism from the ground up: A practical approach for modern life*. Boston, MA: Wisdom.
