# Supplementary material for: Silence in Shamatha, Transcendental, and Stillness Meditation: An Evidence Synthesis Based on Expert Texts
Source: Front Psychol. 2020 Jul 8;11:1259. doi: 10.3389/fpsyg.2020.01259 (PMC7360996; doi:10.3389/fpsyg.2020.01259)
Supplement: Supplementary file 10 [file Data_Sheet_10.pdf]

Database Table S2: Transcendental Meditation Extraction Table

Table S2

Transcendental Meditation Extraction Table

Part 1: Technique and Interim-states

1.1 Simple

Pearson (2013)	"... a simple, natural, effortless procedure ..." – p. 28<br>Pearson p. 394 makes a similar comment.
Roth (2018)	"... simple yet precise technique ..." – p. 5<br>"To define TM, I always start with three adjectives: simple, natural and effortless." – p. 29 "TM is simple ... because there is an elegant simplicity to the practice." – p. 29
Shear and Jevning (1999a)	"... [T]he simplicity of the technique and the importance of effortlessness are major foci of the standardized instruction procedure." – p. 193
Shear (2006c)	"... a simple mental procedure ..." – p. 24<br>"Perhaps the most striking feature of [TM] is the simplicity and effortlessness of the practice." – p. 25
Rosenthal (2016/2017)	"... the simplest and most effortless way I know to access transcendence ..." – p. 8<br>"Yes, the technique is simple and effortless, but for many people, learning it can be – at least initially – complicated and confusing! I have heard many stories from intelligent and sophisticated people who, despite having learned, were not achieving their desired results. A brief consultation with their TM teacher often revealed the source of difficulty, putting the meditator back on track." – p. 24<br>"I had personally found TM to be simple and easy ..." – p. 151<br>Quoting a patient: "It's mind-bogglingly simple ..." – p. 151
Rosenthal (2011/2012)	Referring to the musician Moby: "In the end, he found that TM worked best for him because of its simplicity ... 'I find the simplicity of it to be very effective.'" – p. 156

1.2 Easy

See also 1.6 ("Effortlessness, control and relinquishment, no concentration").

Pearson (2013)	"[TM] is easy to learn ... – so easy even 10-year-old children can do it." – p. 29<br>"Anyone can easily learn and practice it, even children ..." – p. 394
Roth (2018)	"... the [form of meditation] that I find easiest to do ..." – p. 16<br>"Why do so many people think that meditation is difficult? The answer is an unfortunate misunderstanding about the nature of the mind." – p. 31<br>"... [Y]ou will know you are meditating properly in [the] beginning days if ... it's easy to do ..." – p. 64
Shear (1990b)	"... [A]ccording to Maharishi [pure consciousness] can be experienced 'easily [through TM]'." – p. 45<br>"Maharishi Mahesh Yogi ... maintains that with the proper procedure the experience of pure consciousness is 'easy' to gain ... The TM technique he introduced to the West is ... by all reports easy to practice." – p. 101<br>".... easy-to-practice techniques of the TM program ..." – p. 226
Shear (1994)	Shear p. 329 refers to the "ease of learning and practice".
Shear (2006c)	"[TM] is an effortless mental technique that quickly and easily enables any ordinary person to experience [pure consciousness]." – p. 23<br>"... allows the awareness to settle down, quickly and easily ... to [pure consciousness]." – p. 24<br>"The technique is ... quite easy to learn, taking only an hour or two per day over four successive days, followed, where possible, by a dozen or so sessions over the next year where the new meditator's practice can be evaluated, and his or her experiences verified." – pp. 28-29

Rosenthal (2016/2017)	"Yes, the technique is simple and effortless, but for many people, learning it can be – at least initially – complicated and confusing! I have heard many stories from intelligent and sophisticated people who, despite having learned, were not achieving their desired results. A brief consultation with their TM teacher often revealed the source of difficulty, putting the meditator back on track." – p. 24<br>"Although one of the advantages of TM is the ease with which it can be learned and practiced, there are also subtle tricks to getting the most out of it." – p. 73<br>"... TM is easy." – p. 150<br>"... I had personally found TM to be simple and easy ..." – p. 151
Rosenthal (2011/2012)	"... [I]t is an easy form of meditation to learn and practice." – p. 14

1.3 Pleasant

See also 1.8 ("Progression to the goal-state/s") and 2.7.26 ("Bliss, joy, happiness").

Roth (2018)	Quoting Shane Anselmi, a meditator: "... I love it. It is something I look forward to because it brings a relaxed peace; a sense of joy, calm and clarity ... And now, any feeling of anxiety seems to disappear." – pp. 66-67
Shear (1990b)	"... [T]hese experiences often appear to be highly rewarding in themselves ..." – p. 228<br>"... states of consciousness which are ... intrinsically enjoyable and fulfilling." – p. 231<br>"... the delightful, inherently fulfilling ... nature of these deeply intimate experiences of consciousness ..." – p. 231
Rosenthal (2011/2012)	"Soon after starting to meditate regularly, TM became for me – as it is for most people – a pleasant and peaceful retreat." – p. 10<br>Quoting David, a meditator: "When I started meditating, I looked forward to the next session. So in the morning I'd be like 'Oh man, I can't wait to meditate again in the afternoon' – because those would be the moments that I would be free of angst and very like myself for at least twenty minutes, and maybe ten minutes afterward. That was how I wanted to be'." – p. 101<br>"People report TM to be a pleasant experience, look forward to their sessions ..." – p. 141<br>Quoting Roth: "... easy and enjoyable ..." – p. 277

1.4 Key features of the practice

1.4.1 General

Pearson (2013)	"Maharishi emphasizes the importance of remaining innocent." – p. 54
Roth (2018)	"[The teacher] will give you your own mantra – a word or sound that has no meaning associated with it – and teach you how to think it properly, which means easily, effortlessly and silently. You'll learn that you don't need to push out thoughts, or watch your breath, or monitor sensations in your body, or visualize anything." – p. 2<br>"How do you get [to the experience that is aimed for]? With a *mantra*, which is a word or sound that serves as a vehicle to facilitate the process of silently settling down from surface to depth, from noise to stillness. The mantra has no meaning associated with it, and according to ancient meditation texts its effects are known to be positive and life supporting. Its sole purpose is to help the mind access that inner calm without effort." – p. 34 "[The teacher] will give you your mantra and then teach you how to use it properly – naturally and effortlessly – without any concentration or control of the mind. You will learn the subtle mechanics of how to give the attention of your ... mind an inward direction so that automatically you begin to settle down and transcend into stillness." – p. 35
Shear and Jevning (1999a)	"In the standardized TM practice one is taught to use a *mantra*, a sound without any associated meaning, and repeat it effortlessly until the mind becomes absorbed and settles down relaxedly. The *mantras* are said to be so well suited to the nature of the mind that, acting purely as 'resonances', they prompt the mind to relax and, following its natural tendency, settle down, and enjoy the experience of 'deeper levels' of consciousness within. As to the procedure itself, the instructors insist that there is not much to be said in general (that is, independently of reference to the individual's own experiences). All that one does is to think the *mantra* effortlessly ... The crucial points of this instruction are said to be the proper choice of the

	particular *mantra* or sound for each individual, and the personal instruction tailored to the particular experiences of the new meditator, so that he or she learns to use the *mantra* 'properly', that is, effortlessly and without concern for whatever thoughts and experiences that may come and go." – p. 193
Shear (2006b)	"[TM] uses relaxed attention to special sounds (or *mantras*) repeated silently within the mind." – p. xvi
Shear (2006c)	"... [The mantra] is used *purely as* mental *sound*, with no meaning attached. This is an essential point of the practice. Attention to meanings engages the intellect and imagination, and keeps the mind active, rather than leading it to settle down to [pure consciousness]." – p. 27<br>"... [C]are has to be taken that the quality of the sound fits the mind and its natural tendency to settle down ... The *mantras* or sounds used in [TM] have all been time-tested over many centuries to be most useful for enabling the mind to follow its natural impulse for greater happiness, and settle down easily ... until the experience of pure consciousness is gained." – p. 28
Shear (2011a)	"[TM] uses relaxed attention to special sounds (or *mantras*) repeated silently within the mind." – p. 156
Rosenthal (2016/2017)	"The mantras, which have no meaning, are ancient and traditional to the practice. Over the centuries they have been found to have soothing, positive (the TM teachers describe them as 'life-supporting') qualities that are conducive to transcendence." – p. 23<br>Quoting Bob Roth: "The mind, by nature, doesn't like to do 'nothing'. The mind likes to think, imagine, visualize, plan. Thinking the mantra engages the mind. And because the mantra has no meaning, the discriminating intellect doesn't have to get involved." – p. 34<br>"In no way is any content introduced or encouraged as part of TM training." – p. 151
Rosenthal (2011/2012)	"During the training, each student is assigned a specific mantra or sound, with instructions on its proper use ... Mantras are particular soothing sounds, known by experience gathered over centuries to bring on transcendence ... The mantras used in TM derive from the Vedic tradition of ancient India." – p. 16<br>Quoting Roth: "According to tradition, the mantra is a sound whose effects are 'positive and life supporting, from the surface of the mind to its deepest, most powerful levels' ... The mantras come from the ancient Vedic knowledge." – p. 279<br>"TM can be superficially described as thinking or repeating a mantra ... and going back to it when it is forgotten – this sounds similar to descriptions of *focused attention* [meditation] techniques. A deeper analysis reveals that ... TM ... is a technique for transcending its own procedures – appreciating the mantra at 'finer' levels in which the mantra becomes increasingly secondary in experience and ultimately disappears ... While focused arousal involves voluntary sustained attention, TM practice involves automatic moving of attention to mental silence. During TM practice, the subject-object relation that defines customary experiences is transcended. In *focused attention* the object of experience is sustained in awareness – the subject (experience) and object coexist ... In TM, the object of experience fades away – you use the mantra to lose it." – p. 288
Faber et al. (2017)	"TM practice is a process of *transcending* – appreciating the mantra at 'finer' levels in which the mantra becomes increasingly secondary in experience and ultimately disappears ..." – p. 307 "During the process of transcending, the mind sometimes becomes engaged in an undirected stream of thoughts ('undirected mentation'). When this happens and is noticed by the practitioner, they learn how to effortlessly return to the mantra. This fluctuation between transcending and undirected mentation is the natural flow of a TM practice. Contrary to focused attention meditation practices, undirected mentation is not considered to be off-task but an integral part of the meditation process." – p. 307 The authors p. 313 refer to the "continuous cycling between these two phases ... during the practice of TM". "... [T]he present study asked participants to press a button when they noticed that they became engaged in an undirected stream of thoughts and to then return to the process of transcending during TM practice." – p. 308 "Subjectively, the two phases of TM meditation differ in content with the appreciation of the mantra in one phase and the engagement in an undirected stream of thoughts in the other." – p. 308 "Participants were instructed to press a button when they noted that their mind was lost in undirected thought. After the button press, they then went back to the process of transcending during TM practice." – p. 308 "Periods of transcending are initiated by effortlessly attending to a mantra. Periods of undirected mentation are presumably initiated by issues of the body or the mind generating the stream of thoughts away from transcending." – p. 312
Travis and Parim (2017)	"Thoughts other than the mantra can arise during TM practice. They are part of the process of exploring deep inner silence. During TM practice, thoughts are not actively suppressed, and losing track of the mantra

	is not seen as a failure ... TM practice is the process of transcending, coming out onto thought, and transcending again using the 'natural tendency of the mind' ..." – p. 87

1.4.2 Dealing with distractions/discomforts
See also 2.7.31 ("Transcendence of discomfort").

Roth (2018)	"If you feel an itch, by all means scratch it." – p. 30<br>"Do you need to stretch your legs? Or shift positions in the middle of the meditation? Do it." – p. 58

1.4.3 Other obstacles
See also 1.6 ("Effortlessness, control and relinquishment, no concentration") and 2.7.27 ("No sense of anxiety").

Rosenthal (2016/2017)	Quoting Roth: "If the question is what are the obstacles that might prevent people from experiencing [pure consciousness], then the answer is stress in the physiology. If your body has a backlog of 'stored up' stress or tension, this could slow the process of transcending for a bit. But the deep rest gained during the practice allows those stresses to be dissolved, which then allows the mind to settle down in subsequent meditations. The only other obstacle would be not meditating regularly ... You have to take the few minutes, ideally twice a day every day, to meditate. If you do that, the rest is automatic." – pp. 260-261

1.5 Other aspects of the practice/instruction
See also 1.6 ("Effortlessness, control and relinquishment, no concentration") and 1.7 ("Posture").

1.5.1 Learning the practice – general

The present table does not focus on aspects of the technique or experience that are class-specific. However, to understand the technique it helps to have some appreciation for how it is learned. The most detailed description is provided by Roth in response to questions in Rosenthal (2011/2012): "[TM] is taught through an initial seven-step course of personal instruction by a certified TM teacher, which is followed by a lifetime of follow-up mentoring that you can access at your convenience. The first two steps are free public lectures – about 60 minutes each – which provide a comprehensive intellectual understanding of the practice. The third step is a personal interview (10 minutes) with a certified teacher. The fourth step (90 to 120 minutes) is personal, one-on-one instruction; this is when you actually learn to meditate. Steps five through seven (60 to 90 minutes each) are follow-up meetings during which you will consolidate both your intellectual understanding and your direct experience of the technique .... [F]or a person to gain initial mastery, sessions four through seven must take place on four consecutive days ..." – pp. 282-283 "After you have completed these seven steps and are enjoying your twice-daily meditation, you can continue to meet with your TM teacher as needed for the rest of your life, to 'check' and fine-tune your technique. These checking sessions are free, can be scheduled at your convenience, and last twenty to thirty minutes." – p. 283 The experts also frequently note that, as Shear (2006c) puts it: "A ... distinguishing feature of the TM program is its complete standardization. For the technique is precisely the same whenever it is taught through any recognized TM center." – p. 31

1.5.2 The technique is quick to learn/perfect
See also 2.6 ("Speed at which achieved").

Roth (2018)	"TM is not an acquired skill that you will get 'better' at after weeks or months of practice. You master it in a few hours over a few days, and then it's yours for the rest of your life." – p. 30 "... [W]hether you are new to the practice or seasoned in TM, transcending ... is natural to the practice because the ability to transcend is not an acquired skill that requires a lot of effort. In fact, it requires no effort at all." – p. 37
Travis and Parim (2017)	"Subjects quickly master [TM] practice." – p. 87

The experts consider that empirical studies on TM support this point. Roth (2018, p. 36) says: "The ease of learning was confirmed by [Travis and Parim, 2017], which found that individuals practising TM for just one month reported the same frequency of transcendent experiences as individuals practising for five years." Pearson (2013, p. 407) discusses a study that compared beginner and experienced meditators. He concludes: "This study showed that when one learns [TM] ... the technique is quickly mastered and there is no 'practice effect' with time. Whether you've experienced [pure consciousness] ten times or 10,000 times, the brainwave pattern is about the same. What does increase over time is EEG coherence throughout the day, suggesting the brain is using more of its potential and [pure consciousness] is becoming integrated with the waking, dreaming, and sleeping states." Based on the EEG studies, Rosenthal (2016/2017, p. 240) says: "In other words, beyond two months, there is no evidence of further change in the EEGs of people practicing TM ... Once you get the hang of it ... your EEG during TM is likely to look the same regardless of whether you have been meditating for three months or thirty years." Rosenthal pp. 240-241 makes comments about the EEG of meditators in daily life (as distinct from

during meditation) that are along the same lines as those of Pearson. Rosenthal (2011/2012, pp. 22-23) says, similarly: "Even novice meditators show these brain wave changes, starting within weeks (or even days) after they begin to meditate. By two months, their brain waves during meditation are indistinguishable from those of meditators who have been practising for decades. [Fred] Travis regards this as confirmation that TM is a technique that is easily learned. Where you do see EEG differences between novices and seasoned meditators is when the people are awake but not meditating. In seasoned meditators, more brain wave coherence can be seen throughout the day." Rosenthal (2011/2012, p. 36) makes similar comments. Travis and Shear (2010a, p. 1116) discuss the "research reporting the lack of a novice/expert dichotomy among TM meditators". "For instance, two cross-sectional studies ... report that brain wave patterns reach high levels during TM practice after a few months practice, and that progressive changes in EEG patterns are seen in activity after the meditation session ...". Travis and Parim (2017, p. 87) make similar comments.

1.5.3 Selection of mantra

Roth (2018)	"... [Y]ou will be given the mantra that is best suited for you ..." – p. 57
Shear (2006c)	"Experience has ... shown that different *mantras* are appropriate for different people ... So for maximum effectiveness the *mantras* used in [TM] are assigned individually in accord with specific criteria." – p. 28
Rosenthal (2011/2012)	"There is a set number of mantras, and the TM teacher selects one for each student based on certain criteria." – p. 16<br>Quoting Roth: "... [N]o, there is not a different mantra for all 6.7 billion people in the world. You could say it's a bit like blood types ... There are a set number of types ... and a trained person can determine which blood type to use for which individual." – p. 279 "... [A] certified TM teacher has spent many months in graduate-level study learning how to select the proper mantra ..." – p. 279

1.5.4 Mantras are private/secret

Rosenthal (2011/2012)	"Students are asked not to reveal their mantras to others. My colleague, Bob Roth ... says that in his experience, when students compare their mantras, the resulting confusion and self-consciousness can inhibit learning and the easy flow of proper meditation. I don't regard my mantra as some deep dark secret, but not discussing it helps me maintain a sense of privacy and specialness about it." – pp. 16-17

1.5.5 Oral rather than written instruction

Roth (2018)	"For well over five thousand years, the TM technique was passed down from teacher to student, one to one: never in groups, never from a book." – p. 2<br>"You typically learn this meditation from a professionally trained teacher who will instruct you in a one-to-one session." – p. 2
Shear (2006c)	"... [T]he instruction ... has to be 'live', with one-to-one feedback by an expertly trained teacher. As Maharishi explains, 'By necessity the practice ... is verbal; it is imparted by personal instruction. It cannot be imparted through a book ...'." – p. 29
Rosenthal (2011/2012)	Quoting Roth: "... TM is always taught through personal, one-on-one instruction by a qualified teacher, initially over a four day period." (p. 280); "Every person is different. Everyone learns at a different pace, and no book or tape can anticipate the questions a particular person may have, or when they'll arise."(p. 280).

1.5.6 Retreats

Although initial instructions are typically provided in a non-retreat environment (see 1.5.1, "Learning the practice – general"), and it appears that ordinarily meditators are not expected to attend retreats, retreats can play a part in the maintenance or development of practice. In Rosenthal (2016/2017), Roth comments: "Weekend retreats, or 'residence courses', are great!" – p. 265

1.6 Effortlessness, control and relinquishment, no concentration

See also 1.4 ("Key features of the practice") and 1.5 ("Other aspects of the practice/instruction").

1.6.1 General

Pearson (2013)	"... a simple, natural, effortless procedure ..." – p. 28

	Pearson p. 394 makes a similar comment.<br>"[TM] is ... effortless to practice ..." – p. 29<br>"... [TM] gives [the transcending] experience effortlessly." – p. 33<br>"... [T]he [TM] technique is unique in a variety of respects – in its effortlessness ..." – p. 37<br>Pearson pp. 45, 393, 400 also refers to TM as effortless.<br>"... The [TM] technique differs from most other procedures in that it involves no concentration or control." – p. 29<br>"Where many other types of meditation involve concentrating on an idea or mental image, focusing on one's breathing, trying to still or 'blank' the mind, or trying to be mindful of the present moment, [TM] differs in being effortless. It does not involve controlling the mind or trying to change one's breathing or relax the body. There is no contemplating of values or ideals, thinking positive thoughts, or examining feelings or beliefs." – p. 394
Roth (2018)	"... to *transcend* – to effortlessly access the deep stillness that lies within every human being ..." – p. 5<br>"There is no concentration or control of the mind; nothing guided; no suggestion or passive observation." – p. 17<br>"... [W]e don't try to control those turbulent waves on the surface, and we don't watch them dispassionately, either. We simply access the calm at the ocean's depth." – p. 17<br>"To define TM, I always start with three adjectives: simple, natural and effortless." – p. 29 "It's natural because there is no suggestion or manipulation involved." – p. 29 "It's effortless because there is no concentration or control required." – p. 29<br>"I once taught a record company executive, and when I said he could move during his meditation, his eyes actually welled up with tears of relief. For decades, he had worked hard trying to master meditation techniques that required concentration and control of the mind and body. He always felt like a failure if he had too many thoughts or needed to scratch an itch or move his leg to get more comfortable." – pp. 30-31<br>Roth pp. 34, 64 also refers to TM as effortless.<br>Roth states that most of the instruction in the initial TM course "involves learning how to meditate – how to use the mantra properly, without effort, concentration, or control" (p. 57).<br>"... the correct mechanics of TM practice: how not to concentrate or control your mind ..." – p. 63
Shear (1990b)	"The TM technique according to Maharishi ... involves neither contemplation nor concentration, but utilizes instead what Maharishi describes as the mind's natural (innate) responses to (the thought of) particular sounds. When the proper sound (or 'mantra') is thought in the prescribed effortless and 'innocent' (i.e., non-directed) way, the mind ... finds the experience comfortable and automatically tends to 'settle down'. The experience of the 'mantra' becomes progressively more abstract and finally disappears entirely ..." – p. 101
Shear and Jevning (1999a)	"... [T]he simplicity of the technique and the importance of effortlessness are major foci of the standardized instruction procedure." – p. 193
Shear (2006c)	"Perhaps the most striking feature of [TM] is the simplicity and effortlessness of the practice." – p. 25<br>"... without any effort." – p. 25<br>"... [T]he procedure has to be one that enhances the mind's settling down, rather than stirring it up through any activities such as concentration or effort. With the right mental object and the right method of using it, however, the whole process unfolds effortlessly and automatically." – p. 27<br>"... [T]he whole process unfolds by itself ..." – p. 28<br>"... [T]he practice of [TM] is quite effortless. In fact, it has to be, since effort inevitably stirs up the mind and moves it away from its completely settled state of pure consciousness." – p. 28<br>"Meditation procedures usually involve some sort of concentration ... or contemplation ... Many procedures also involve trying to remember some state, object, or intended result outside of sitting meditation as well. One of the most important features of the TM practice, however, is that it does not involve any of these kinds of activities at all ... [T]here is no attempt to generate, remember, and/or anticipate results ... [A]ll such attempts would tend to keep the mind involved in its ordinary sorts of surface activities rather than allowing it to settle down naturally to [pure consciousness]." – p. 30<br>"[The] experience is gained through [TM] automatically. It emerges innocently through the practice, without any attempt to look for it. Indeed, thinking about the experience and attempting to find it will only stir the mind up and make it harder to attain." – p. 34
Shear (2011b)	"Once one has learned [TM], the whole process is virtually automatic. All one does is start it and let it unfold by itself. Any effort to sustain or otherwise manipulate it only gets in the way." – p. 53

Shear (2014b)	"The use of *mantras* in TM eschews effort and concentration ..." – p. 211
Rosenthal (2016/2017)	"Paradoxically, forcing the mantra ... or pushing yourself to get the technique exactly 'right' is often counterproductive. Effortlessness ... is the key to successful transcendence." – p. 26<br>"Essentially, [the word 'innocently'] means 'without expectation as to the outcome." – p. 26<br>Quoting Barry, a meditator: "... [In] TM ... the only thing you control is the mantra. Then everything else just happens around us." – p. 101<br>"In mindfulness, the meditator is systematically trained to intentionally focus attention on something in particular, whereas in [TM] emphasis is placed on the automatic ease with which a mantra is accessed." – p. 150<br>"TM ... is not associated with focus and concentration ..." – p. 160<br>In an interview with Roth, Rosenthal says: "I have seen people learn to meditate and then stop altogether – often even when they know they have been helped! Why do people do this?" – p. 261 Roth replies: "[One of the main reasons] is that people stop meditating correctly. They inadvertently introduce into the practice some effort, some concentration or control of the mind. As soon as they do that, they are no longer practicing TM, so the experience is no longer effortless, no longer enjoyable. It is no longer relaxing and rejuvenating." – p. 262
Rosenthal (2011/2012)	"Here is another crucial but little known point: The mantra alone is not enough ... Using the mantra properly – effortlessly, without concentration or deliberate control of the mind – is as important as the sound itself." – p. 16<br>"There's no effort, no need to concentrate or control my mind." – p. 18<br>"At certain points during any session, I may experience transcendence ... I am not thinking of anything in particular. Rather, transcendence is an experience of unfocused bliss, which may last for seconds or minutes. It comes and goes mysteriously and cannot be forced. It is a gift of the meditation, one that comes on its own through effortless, innocent practice." – pp. 18-19<br>Quoting Lyn, a meditator: "What I immediately found in [TM] that was different was its ease. In all other methods there was effort involved: effort in getting quiet; effort in redirecting thoughts or being mindful." – p. 207<br>Quoting businessman George Crowley: "My nature is that I'm a very disciplined person, and what seemed counterintuitive to me was the notion that the mantra is most effective when I'm not focused on it. So I was trying to force my focus onto the mantra. By trying to impose my will as opposed to just letting the mantra happen, I was actually defeating one of the most important aspects of the practice. Now I recognize that I should not experience the mantra as a singularity of focus, but rather let it innocently enter my consciousness. And if thoughts emerge while I'm experiencing my mantra, I need to just recognize that they're there and gently return to the mantra – not try to grab it by the throat ..." – pp. 218-219<br>Quoting Roth: "... [N]or does [TM] involve 'mindfulness' or any other form of meditation based on 'focus', 'concentration', or 'control' of the mind." – p. 277<br>"... TM ... does not involve any concentration, manipulation, suggestion, or control of the mind. There's no mindfulness, no contemplation, no visualization ..."- p. 278
Travis and Shear (2010a)	"We suggest a third category of meditation practice, *automatic self-transcending*, which includes techniques designed to transcend their own activity." – p. 1110 "The category of *automatic self-transcending* is marked by the absence of both (a) focus and (b) individual control or effort." – p. 1111 "*Automatic self-transcending* practices involve transcending of the procedures of the meditation. Since cognitive control increases mental activity, self-transcending procedures would need to involve minimal cognitive control – be automatic or effortless." – p. 1115 TM falls within this category (p. 1115).<br>"TM can be superficially described as thinking or repeating a mantra – a word without meaning – and going back to it when it is forgotten (Raffone and Srinivasan, 2009) – this sounds similar to descriptions of focused *attention* techniques. A deeper analysis reveals that the TM technique is a technique for transcending its own procedures – appreciating the mantra at 'finer' levels in which the mantra becomes increasingly secondary in experience and ultimately disappears and self-awareness becomes more primary ... While focused arousal involves voluntary sustained attention, TM practice involves automatic moving of attention to mental silence." – p. 1116<br>"The TM technique is automatic at the outset, rather than through extensive practice. This is said to be because it uses the 'natural tendency of the mind' to transcend perception of the mantra ..." – p. 1116
Travis (2011)	"Meditation techniques in [the automatic self-transcending] category do not attempt to control the movement of attention or to monitor ongoing experience; rather they are designed to transcend their own activity – to allow a state of consciousness to emerge when mental activity and cognitive control has been

	transcended. Techniques in this third category ... must be automatic, because any intention to control the attention would keep the mind active and not allow mental activity to settle to silence." – p. 224
Travis (2014)	"Meditation practices in the *automatic self-transcending* category transcend cognitive and affective processes to reveal ... pure consciousness." – p. 1<br>"Any intention or individual directing of the mind leads to increased activity in a localized area – the mind cannot transcend." – p. 3
Travis and Parim (2017)	"The [TM] practice [is] described ... as not involving cognitive control – neither focusing the mind nor directing of attention. Rather, [TM] practice was described as using the *natural tendency of the mind* to transcend." – p. 90

1.6.2 The natural tendency

Pearson (2013)	Pearson addresses the question of how TM allows the mind to settle into pure consciousness: "It simply creates the proper initial condition. Once this condition is created, the mind settles inward by itself, spontaneously and naturally." – p. 395 Under the heading "The natural tendency of the mind", Pearson states: "Maharishi ... observes that [the mind] moves purposefully, in a specific direction – always toward greater happiness, toward fields of what he calls 'greater charm'. For instance, if someone were to start playing your favorite song nearby, your mind would require no effort from you to move away from this page and toward the music." – p. 395 "The field of pure consciousness offers in superabundance everything the mind seeks – it gives the experience of pure bliss, freedom from limitations, unbounded creativity and intelligence. [TM] simply allows the mind to remain awake and alert without being focused on a thought. Once this initial condition is created, the mind spontaneously settles inward." – p. 396 "Maharishi compares the process to diving – you just take the correct angle and let go, and the force of gravity does the rest ... The procedure, using the very nature of the mind, is simple and automatic." – p. 396<br>Quoting Maharishi Mahesh Yogi: "To go within is so simple; it is so natural for every man to go to a field of greater happiness." – p. 455
Roth (2018)	"Unlike other meditation practices, thoughts are part of the process in TM. There is no need to control your monkey mind because, in fact, your mind is not a monkey that needs controlling. It is not wandering aimlessly because your mind is not wandering at all." – p. 32 "It was Maharishi's insight that the natural tendency of the mind is not to wander without direction but rather to seek something more satisfying, more charming; greater knowledge, more happiness. And this insight is at the core of understanding not only how TM works but also how it differs from other forms of meditation." – p. 32 "... [Y]our mind is drawn naturally to something more satisfying." – p. 33 Roth pp. 32-33 gives three examples: (a) listening to bad music and then attention automatically going to good music played in another room; (b) listening to a boring speaker and attention being drawn to an interesting conversation; and (c) being attracted to an absorbing book in preference to a boring one. Roth p. 34 indicates that in TM the mind moves towards the "most satisfying level of awareness". "[TM] makes use of the mind's natural tendency to seek more satisfaction and then gives the attention an inward direction, so that instantly, automatically and effortlessly the attention is drawn within. The active thinking mind settles down towards the most satisfying level of awareness." – p. 34<br>"It's the nature of the mind to be drawn effortlessly to fields of greater satisfaction." – p. 37<br>Quoting Donna Rockwell, a clinical psychologist and meditator: "Yes, you think the mantra but you don't have to strain about it. Your mind goes where it wants to go, towards something more satisfying." – p. 61
Shear and Jevning (1999a)	"Maharishi attributes [the] settling [of the mind] to the fact that the 'subtler' levels of the mind are more enjoyable to experience. Thus as the mind settles down 'it finds the way increasingly attractive' ..." – p. 193
Shear (2006c)	"The TM technique ... allows the mind of any ordinary person to settle down automatically, until it comes to experience [pure consciousness]. It does this by using the natural tendency of the mind." – p. 26 "What is this natural tendency of the mind? It is to enjoy. The mind tends to move to a field of greater happiness automatically. If we are walking with a friend, for example, and hear some music that we like, the mind automatically goes in that direction. Also, as it turns out, the deeper levels of inner awareness feel very good, and the deeper we go within, the better we feel ... [T]hroughout history, and across different civilizations, those who have experienced the full range of mind agree that the deepest level of inner awareness is what can be described as *pure happiness* or fulfillment." – p. 26 "... [A]s Maharishi explains, since the deeper levels of the mind are naturally more attractive, once the mind is pointed in the right inward direction 'it finds increasing charm at every step of its progress', and, 'drawn by its very tendency to go to a

	field of greater happiness, finds its way to transcend the subtlest state of thinking and arrive at the bliss' of pure consciousness, spontaneously and automatically." – p. 26<br>"The question may arise of why, if the mind automatically tends to move toward a field of greater happiness, it should need any technique in the first place. Sometimes, of course, people do experience this field spontaneously ... But this is very rare. And when it happens, the experience is often not repeatable ... [W]hat is needed is a procedure that interacts with the mind in such a way that the activity of the procedure automatically becomes less and less and ceases entirely, until the procedure itself is transcended ..." – pp. 31-32<br>"... [H]appiness increases as the mind settles, and this draws the mind quite spontaneously to the state of pure consciousness ..." – p. 34<br>"All that is needed is a gentle prompt, pointing the direction to pure consciousness and happiness within, and the rest unfolds naturally, all on its own." – p. 48
Rosenthal (2011/2012)	In response to the question from Rosenthal, "How does TM work?", Roth responds, "[TM] makes use of the mind's natural tendency to be drawn – or attracted – toward fields of greater charm or satisfaction" (p. 277). Roth pp. 277-278 provides music and books examples along similar lines to those provided in Roth (2018) above. He then says: "We infer that deep within the mind ... is a reservoir of energy, intelligence, and happiness. You would logically expect that this field would be an incredibly satisfying place to 'be' and it is – after all, it is your own inner Self." – p. 278 "It's like leaning over to dive into a pool – once you're in the right position, the movement just naturally happens. With no effort at all, your attention moves to increasingly subtle, quiet, creative, and energetic levels of the mind." – p. 278
Travis and Shear (2010a)	"TM is described as being automatic in that one uses the 'natural tendency of the mind' to transcend ..." – p. 1116
Travis and Parim (2017)	"The *natural tendency* of the mind is more than mind wandering ... The movement of the mind to more interesting experiences is what is intended by the phrase the *natural tendency of the mind*. The mind takes that direction due to the inherent pleasure in the experience, rather than through cognitive control. Thus, meditation practices that provide an experience that is pleasurable to the individual could be readily learned and mastered without extensive practice." – pp. 90-91

1.7 Posture

Pearson (2013)	"[TM] is practiced ... while sitting comfortably with eyes closed." – p. 29<br>Pearson p. 394 makes a similar comment.
Roth (2018)	"You'll ... learn that there is no need to sit in any particular position. You can sit up comfortably in a chair at home, at work on a train or plane; on a park bench – basically, wherever it's comfortable." – p. 2<br>"... TM is practised ... sitting up comfortably in a chair (or on your bed or wherever you can sit up comfortably), with your eyes closed." – p. 30<br>"How do I sit? The answer is: comfortably ... [T]here is no particular way to sit ..." – p. 58 "Nor do you need any special type of chair. In my office, students sit on folding chairs, easy chairs, or a sofa. Just use a chair you like." – p. 58
Rosenthal (2016/2017)	"Sit comfortably in a chair ... with your eyes closed ..." – p. 22

1.8 Progression to the goal-state/s

See also the sections above, and 2.7.18 ("No sense-perceptions").

1.8.1 General

Pearson (2013)	Quoting a meditator describing their first session: "I began to drift down into deeper and deeper levels of relaxation ..." – p. 28<br>"... [M]ental activity [settles] inward through finer values of thought, then [transcends] thought altogether ..." – p. 45<br>"... [The mind settles] effortlessly to subtler, more refined levels of thought until one experiences ... pure consciousness." – p. 47

	Quoting a meditator: "... [M]y mind settles down, thoughts become less and then suddenly all thought activity ceases ...." – p. 52<br>Quoting a meditator: "Meditation begins as most do. I feel as if I am a small stone sinking into an ocean of filtered light. The light seems to be coming from all directions. I slow, almost pause, at certain levels before my specific gravity increases again, and I sink deeper into this ocean of consciousness – no real experience of having entered this ocean – just being there and going deeper and deeper. It is beyond peaceful, beyond serene" (p. 53); "Then something quite extraordinary happens. The ocean disappears. I mean it is just gone, and I am in an indescribable place" (p. 53).<br>"[TM] allows the mind to settle inward, through less active states. Eventually one experiences the very faintest level of thought and, [transcends] even that ..." – p. 395
Roth (2018)	"... TM simply allows the active-thinking mind to settle down to its own state of inner stillness ..." – p. 17<br>Quoting George Harrison from the Beatles: "... [T]he mantra becomes subtler and subtler until finally you transcend even the mantra." – p. 40<br>Describing Roth's own first session of TM: "As [the teacher] led me through the beginning steps ... I felt my mind and body sinking into a state of deep relaxation ... [W]ithin seconds of my first experience meditating, I felt this wave of physiological peace come over me. The tension in my neck, shoulders and stomach muscles were the first to go – and my racing mind settled down." – p. 152<br>Quoting the singer Katy Perry: "Thoughts will come and go, and it will be five minutes before I can get to that stillness." – p. 164
Shear (1996b)	"[A]ll the fluctuations of mind ... settle down ..." – p. 367
Shear (1995/1997b)	"... allowing all noticeable activity of mind to 'settle down'." – p. 372
Shear and Jevning (1999a)	"... [A]s the mind settles down 'it finds the way increasingly attractive' [quoting Maharishi Mahesh Yogi] ... spontaneously experiencing progressively 'finer' aspects of the *mantra*, until finally it settles entirely, transcending all experience of the object (the *mantra*) that had been engaging it." – p. 193
Shear (2004)	"... [O]ne's attention reportedly becomes absorbed in [the mantra], other objects recede from one's awareness, awareness of the [mantra] itself becomes subtler and subtler, and one finds oneself settled into a completely objectless state of awareness ..." – p. 86
Shear (2006c)	"Many different experiences can occur as the mind settles down ... These experiences naturally vary widely from individual to individual ... Teachers of [TM] also emphasize that it is a definite mistake to look for any particular experiences in meditation. The activity of looking for particular experiences tends to block the innocence of the practice, keeping the mind stirred up on the level of thoughts and expectations, and impeding its natural tendency to settle down ... Proper practice of [TM] produces [a] refinement [in mental activity] independently of whether or not one happens to have any particular 'flashy' experience. Moreover, since the experiences produced by the refined activity ... can be highly abstract, and therefore, at first, hardly noticed (much less remembered), a great deal of refinement can take place without one's noticing any remarkable experiences in meditation at all." – p. 32 "Nevertheless, specific, easily recognizable experiences of deep levels of consciousness do often occur during the practice of [TM] ..." – pp. 32-33 "... [A]s one settles down in meditation, one can experience more and more subtle states of the 'object', until the subtlest state of the object is experienced and then transcended ..." – p. 33 Shear pp. 33-35 discusses pure consciousness. He then says: "... [J]ust before reaching its completely settled state of pure consciousness, the mind may pause at a level that can be called *pure* happiness, or bliss." – p. 35
Shear (2007)	Shear pp. 699-700 states that the understanding in Vedanta is that the meditator moves from surface awareness to pure consciousness, the deepest level of awareness. The levels include, from surface inwards: the senses, the "discursive thinking *mind*", "the *intellect* capable of discriminating between the mind's diverse contents", the ego, pure positive affect, and pure consciousness (p. 699).
Rosenthal (2016/2017)	"As ... thoughts mingle with the blissful peace of transcendence, they become less bothersome, disturbing, or problematic." – p. 26<br>"... [I]t often takes the first five or six minutes of a TM session for my mind and body to settle down ... Then comes a period of stillness, and only in the last third of the session do I enjoy the full bliss of transcendence." – p. 27<br>"Once in a while, some people feel minor anxiety on first meditating ... If that should happen, checking in with your TM teacher usually resolves the matter within a few minutes." – p. 35

	"Could transcendence be construed as a type of mind-wandering?" – p. 158 "The ... question, whether transcendence is a type of mental wandering, is ... interesting." – p. 158 Rosenthal does not provide a clear answer, but he notes that TM has been found to be "accompanied by increased [default mode network] activity" (p. 160), and implies that this may reflect mind-wandering (p. 163).<br>Quoting Roger, a meditator: "My mind was quieting. Thoughts didn't capture my attention or my awareness. They would just pass by." – p. 221
Rosenthal (2011/2012)	Quoting Roth: "... [Y]our attention moves to increasingly subtle, quiet, creative, and energetic levels of the mind." – p. 278
Travis (2001)	Travis p. 2 refers to a TM session as involving "transcending", Transcendental Consciousness, and "other" experiences. He defines "transcending" as "taking the mind from the experience of a thought to finer states of the thought" (quoting Maharishi Mahesh Yogi). "[The] 'other' category comprises a range of possible experiences when the mind is out of the process of transcending, primarily characterized by an increase in mental and physical activity." – p. 2
Travis et al. (2005)	Quoting a meditator: "... [M]y thoughts become less and less concrete, less and less absorbing ..." – p. 127
Travis and Shear (2010a)	"... [T]he rate of transcending may vary, person-to-person, and often meditation-to-meditation, owing to differences in the mind and body when one sits to meditate." – p. 1116

1.8.2 Pure individuality and pure bliss/joy/happiness

Shear (1990b)	Shear p. 42 indicates that "bliss" refers to "that which when experienced in its pure form is recognized to be the ultimate object of all desires, that is, that which one actually desired in everything that one had ever desired (including all forms of aesthetic, erotic, and intellectual desire), whether or not one had ever suspected that such an ultimate object of desire and completely fulfilling experience existed."<br>Shear pp. 43-44 discusses how Maharishi Mahesh Yogi stated that moving towards the pure consciousness goal-state there is an experience of "pure individuality", where (in Shear's words) "one's mind is said to settle beyond all objects of experience and be left only with its own individual self-awareness". "This experience of pure individuality is likened to a wave on an ocean of bliss. It is then said to settle into the ocean itself, transcending all distinctions between subject and object." – p. 44 "... [The] 'ocean' [is] devoid of all specific objects of experience yet still bliss-permeated." – p. 45 "... Maharishi describes the experience of an ocean of bliss as arising prior to the experience of [pure consciousness]." – p. 45 In the pure individuality experience, "individuality, the 'I' of ordinary awareness, is said to be [experienced] in its pure state" (p. 45). Shear p. 46 refers to it as "a level retaining individual perspective and associated with unbounded beauty or bliss". "While devoid of all objects, this experience still retains the subject-object distinction and awareness of the (empty) perceptual field where objects of experience are usually located." – p. 45<br>Shear pp. 163-164 indicates that the "bliss-permeated" (p. 164) pure individuality involves pure bliss, joy or happiness. Having discussed pure bliss/joy/happiness, Shear says: "The [final part of the meditation] experience appears to unfold in two readily distinguishable stages" (p. 164). The first is bliss-permeated pure individuality, and the second is pure consciousness. "... [T]he experience of pure happiness appears to be a permeating component of the experience of pure individuality, the penultimate experience of self within." – p. 164 Shear p. 166 refers to pure happiness as the "penultimately deep aspect of the nature of self". "The experience is generally described ... as that which we want in everything else that we want ..." – p. 163 Shear describes it as "happiness ... in its pure form, isolated from all other sorts of experiential contents" (p. 163). Shear p. 163 notes that "fulfillment" is another term "frequently used to describe the experience". "... [T]he experience of pure happiness contains empirical content, namely happiness, and is thus not absolutely transcendental in the sense of transcending all empirical content. But it is (phenomenologically) transcendental to all objects in the space-time manifold ... [I]t is identified as the 'quality' permeating the experience of pure individuality ..." – p. 167<br>Shear p. 171 refers to "an inner sense of bliss and wellbeing".<br>"... [T]he experience of pure individuality appears in general to be accompanied by a strong intuitive response to the effect that *this* is one's true personal identity, what one 'really is' ..." – pp. 172-173
Shear (2002)	Shear discusses what he calls "pure positive affect". "The ... experience ... is only slightly less abstract [than pure consciousness]. It has as its content only the reportedly delightful and fulfilling sensation of unbounded positive affect (variously referred to as pure happiness, bliss, joy, etc.), experienced independently of all other qualities or objects of awareness whatsoever." – p. 363 "[L]ike [pure

	consciousness], it has no objects, words, symbols, or thoughts in it. But unlike ... pure consciousness, [it] does have definite content, namely the abstract but nevertheless, by all reports, profoundly fulfilling experience of ultimate positive affect ... [I]n [Vedanta] the experience of pure bliss is said to emerge when the mind in meditation has settled within itself, beyond all determinate experience, where [quoting Maharishi Mahesh Yogi] 'It holds its individuality in the void – the abstract fullness around it'. Here, 'pure bliss' emerges, an 'intensity of happiness beyond the superlative', where no greater happiness or gain can be conceived." – p. 363 "... [T]he experience displays the ultimate affective object of all of our experiences (i.e., what we ultimately seek affectively in all of our other experiences) ..." – p. 365<br>Shear p. 366 discusses problems in "[e]valuating the phenomenological sameness of experiences we have been calling 'pure positive affect'". One is that: "[D]escriptions from different individuals and cultures of what we have been referring to as a single simple experience use terms such as 'happiness', 'bliss', 'ecstasy', and even 'pure beauty'; these terms do not have precise, generally agreed-upon significance, and they can easily be taken to have nonequivalent connotations above and beyond that of simple 'positive affect'." – p. 366 "... [T]he experience I call 'pure positive affect' can equally well be called one of 'pure happiness'. Indeed, it traditionally has been referred to in these terms and others equivalent to it ('infinite joy', 'pure delight', 'absolute bliss', etc.)." – p. 370
Shear (2006c)	"... [J]ust before reaching its completely settled state of pure consciousness, the mind may pause at a level that can be called *pure* happiness, or bliss ... [I]n the experience of pure happiness, happiness is experienced independently of all other things. Here the experience is not that of being happy *about* some thing or other. It is just happiness, all by itself. In other words, as one settles toward pure consciousness in meditation, one gradually becomes more and more familiar with the experience of fulfillment in itself." – p. 35<br>Shear p. 44 refers to "what can be called pure happiness, bliss, or fulfillment itself".
Shear (2011a)	Shear p. 150 discusses levels of awareness similar to those described in Shear (2007) (see 1.8.1, "Progression to the goal-state/s – General"). Referring to the levels as experienced in the major meditation traditions: "Noticing the levels may require that the settling [of the mind] ... take place slowly, especially because the deepest levels are exceedingly abstract. Depending on the technique practiced and one's habits of attention, one may simply 'pass through' any particular level without noticing it, especially at the beginning of one's practice. But all the levels are said to be experienceable by sufficiently advanced practitioners." – p. 150<br>The three deepest levels are "pure individuality or ego", "pure bliss (pure positive affect)" and "pure consciousness (pure emptiness)" (p. 150). Shear p. 151 says that pure individuality or ego "at first may appear to be completely empty and thus seem to be the experience of pure consciousness ...". "For it is completely devoid of all sensations, thoughts, images, and other localized phenomenal objects – all the kinds of things, in other words, that we are ordinarily aware of ... It is in effect experience of the 'space' of mind – the phenomenological manifold. Traditionally the experience is likened to that of being *a disembodied observer* in the midst of *vast emptiness*." – p. 151 "... [U]nlike the experience of pure consciousness ... this experience is not *completely* empty, phenomenologically speaking. For the sense of being a disembodied observer or 'mind's eye' *in the midst* of vast emptiness makes it clear that it still contains the 'I-it' *structure* of ordinary experience, even if the 'it' has been reduced so far towards nothingness that nothing but the emptiness of the phenomenal manifold remains to be experienced. In the middle of the experience, one does not think 'Ah, emptiness', since this would be a thought, and there are no thoughts or other phenomenal objects here. But the empty structure can be remembered, and recognized conceptually, when one returns to more ordinary levels of awareness. It can also be remembered as permeated by abstract undifferentiated objectless bliss." – pp. 151-152 Shear p. 152 says that pure bliss "is even more abstract". "For here there is no longer any sense even of being an observer or having a vantage point. Thus even the empty subject-object duality of [the pure individuality or ego level] is no longer present. All that remains is abstract bliss or well-being itself – 'happiness beyond the superlative', as the *Bhagavad Gita*, canonical to most Indian traditions, puts it." – p. 152
Shear (2011b)	"Other well-known experiences described in the traditional literature and reported by TM meditators include one 'pure individuality' where the meditator experiences himself or herself as a pure, seemingly disembodied consciousness in the midst of a vast, empty phenomenological space; another 'pure bliss' involves experience of pure positive affect by itself, independent of any awareness of localized phenomenal objects." – p. 54
Shear (2014a)	Shear p. 59 introduces the levels of awareness in similar terms to Shear (2011a) above. He then goes on: "... [Pure individuality] is likened to being *a disembodied observer in the midst of vast emptiness*. Yet phenomenologically speaking, it is not entirely empty. For despite the absence of objects, the

phenomenological I-it 'intentional' relationship remains. *One* is *there* as disembodied pure observer, a bare 'mind's eye' so to speak, aware *of* the vast empty phenomenological space surrounding in all directions ..." – p. 59 "Thus, as empty as this experience seems, it still retains the *structure* of ordinary experience. After one emerges from the experience, it is also often remembered as permeated by abstract well-being or bliss." – p. 60 Shear p. 60 makes comments about pure bliss similar to those in Shear (2011a).
Shear p. 71 acknowledges that a research program investigating the above map of levels of awareness could fail. "The map could be incorrect in various ways." – p. 71

1.9 Practice duration

Pearson (2013)	"[TM] is practised for 20 minutes twice a day ..." – p. 29<br>"It is practised for 15 or 20 minutes twice daily ..." – p. 394
Roth (2018)	"... [T]he TM technique has been honed to 20 minutes, twice a day ..." – p. 2<br>Roth p. 30 makes a similar comment.
Shear (2006c)	"... the normal routine of two fifteen or twenty-minute meditations per day ..." – p. 30
Rosenthal (2016/2017)	"... twice a day ... for twenty minutes each time." – p. 22
Rosenthal (2011/2012)	Quoting Roth: "... [TM] ... is practised ... for fifteen to twenty minutes twice a day." – p. 276

Part 2: Goal-state/s

2.1 Terminology – "pure consciousness", "transcending", and related terms

The five experts use a range of terms to describe the goal-state/s, however the most common are "pure consciousness", "Transcendental Consciousness", and "transcendence". For ease of understanding, this table mainly uses those terms.

The experts use the term "transcending" in two main ways. Often they use it to refer to the meditator accessing the goal-state/s. For example: Roth (2018, p. 5) says, "... to *transcend* – to effortlessly access the deep stillness that lies within every human being ..."; and Travis (2011, p. 231) refers to "transcending – the repeated experience of pure ... consciousness". In other places, the experts use the term in a much broader manner, that encompasses any movement towards deeper levels of experience in the practice, including deeper *interim*-states. For example: Pearson (2013) states that "[TM] enables mental activity to settle inward, spontaneously and naturally. Maharishi called this process *transcending*, meaning *to go beyond*." (p. 29); Roth, quoted in Rosenthal (2016/2017), says, "The definition of 'transcend' is to 'go beyond'. So in every meditation you do go beyond the surface, excited level of thinking to a quieter, more settled level of thought. And then as you continue to meditate ... you will periodically transcend, or settle down, to the *next* quieter level of thinking – and so on." (p. 260); and Travis and Parim (2017) refer to "transcending – awareness moving to increasingly more abstract, more charming, levels of inner experience" (p. 92).

2.2 Shear's logic-based argument that pure consciousness has a unique experiential quality

Across his texts, Shear repeatedly argues that pure consciousness has a unique experiential quality. For example, in Shear (1990b) he says: "... [A]s the following argument shows, there can be at most only one [pure consciousness] experience. Suppose that there exist two such experiences A and B. If A and B are different, at least one of them has some empirical content not present in the other, and they cannot both be experiences totally devoid of empirical content. Thus we see that pure consciousness can be defined uniquely as that experience which has absolutely no identifiable empirical qualities within it, that is, which is devoid of identifiable spatio-temporal content." – p. 104 Similarly, in Shear (1996b) he comments: "... [I]t is easy to see that any two experiences which fulfill the defining characteristic of being instances of 'pure consciousness' have to be phenomenologically identical. Consider any two such experiences, A and B. If they *were* (phenomenologically) different, then at least one of them, say A, would have to have some content that the other did not. But then that experience would have empirical content (i.e., content which could be either present or absent), and would not fulfill the defining characteristic of an instance of a 'pure consciousness' experience, contradicting the original supposition. In short, the fact that any experience fulfilling the defining characteristic of a pure consciousness experience can have no empirical content at all implies that all such experiences are phenomenologically identical, and therefore, not culture-specific. In other words, the definition of 'pure consciousness' identifies a unique experience ..." – p. 369 Consistently, he later remarks that the property of being "completely devoid of empirical qualities" "defines a single, logically unique experience" (p. 370). However, in Shear (2002) he observes: "These uniqueness arguments ... are rather formal, and the reality of most of our experiences is that they do not fit the formalisms of our

linguistic categories and characterizations so neatly." – p. 366 He goes on to discuss the possibility of a meditator having an experience that seemed to be contentless, but then having a further (deeper) experience that revealed there had in fact been some content (see 2.7.15, "Limited or no abstract content"). He then refers to the alternative to the experiential uniqueness as being that the experiences are "closely related clusters", or "clusters of related experiences" (p. 367).

2.3 A fourth major state of consciousness, and other higher states

Each of the five experts adopts, argues for, or notes the understanding advanced by Maharishi Mahesh Yogi that pure consciousness represents, in Pearson's (2013, p. 30) words, "a fourth major state of consciousness, distinct from the familiar states of waking dreaming, and deep sleep" (Rosenthal, 2016/2017, p. 4; Roth, 2018, p. 40; Shear, 2006c, pp. 34-35; Travis, 2014, p. 2). On Maharishi's understanding, which is also acknowledged and frequently adopted by the experts, "[pure consciousness] forms the portal to still higher states of consciousness ..." (Pearson, 2013, 21). "The fourth state ... forms the gateway to the higher states. With regular experience of the fourth state, the three higher states develop naturally" (Pearson, 2013, pp. 38-39). The higher states are not the focus of the present table, but it helps to have some appreciation for them. The most detailed descriptions are provided by Pearson (2013), and the passages that follow capture the essential aspects. "Maharishi's model comprises seven states of consciousness altogether, of which waking, dreaming, and deep sleep are but the first three." – p. 38 "Each [of the higher states] is a progressive stage of awakening, of enlightenment. Each opens a distinctive new world of experience and knowledge ... And each has a corresponding style of physiological functioning, a unique physiological signature." – p. 39 "[T]he [TM] technique's ultimate purpose [is] to cultivate higher states of consciousness – enlightenment." – p. 39 In the fifth state, termed Cosmic Consciousness by the Maharishi, "[pure consciousness] becomes permanent, forming an underlying continuum that coexists with waking, dreaming, and sleeping" (p. 173). "... [T]here are no fantastic visions, no fireworks. Developing Cosmic Consciousness means becoming normal and natural ..." – p. 174 "It develops [gradually] as the mind becomes increasingly familiar with pure consciousness and the body becomes increasingly refined and free of accumulated stress, strain, and fatigue." – p. 174 Pearson p. 41 explains that the sixth state, God Consciousness, involves "[refined] perceptual abilities". "One gains the ability to perceive finer, subtler strata of creation. In time one perceives the very finest material structure, indescribably radiant, glorious, celestial." – p. 41 The seventh and final state is Unity Consciousness. In this state, one "experience[s] all things in terms of their innermost essence ... pure consciousness" (p. 41). "This is what Maharishi calls supreme knowledge, *total knowledge*." – p. 320 "... [I]n [Unity Consciousness], knowledge is complete." – p. 321 "This highest stage of enlightenment ... brings the ability to know anything, do anything, and accomplish anything." – p. 322

2.4 Statements that may be metaphysical and that do not clearly describe an additional feature of the goal-state/s

A number of passages in the expert texts give the impression that they may be describing metaphysical understandings concerning pure consciousness, and it is not clear whether they are also describing experiential features in addition to those referred to in section 2.7 below. Examples of these are provided in this section 2.4. These types of passages have not been included in section 2.7.

Pearson (2013)	Describing insights of the Vedic *rishis*/seers: "In the innermost depth of the mind lies the fundamental reality of nature itself, the all-pervading field of unity from which the infinite diversity of the universe is born. Everything in nature, from the flowers on the mountainsides to galaxies without end, arises from within this ocean of crystal clear consciousness." – pp. 25-26<br>"Deep within everyone, Maharishi taught, is an ocean of pure consciousness, pure intelligence, pure bliss – the field of unity from which the diversity of the universe arises." – p. 29<br>Pearson pp. 33-37 indicates that, consistent with the Vedic tradition, pure consciousness can be considered "a field of unity ... in which everything has its being and apart from which nothing could exist." – p. 33<br>"Maharishi has described [pure consciousness] as the *total potential* and the *total reality* of consciousness. One transcends individuality and gains universality." – p. 45<br>"In [pure consciousness], the mind opens to its unbounded inner potential, the limitless creativity and intelligence at its source." – p. 48<br>"[Pure consciousness] is the source of nature's intelligence, the unmanifest, nonchanging field of unity underlying the infinitely diverse, ever-changing world of forms and phenomena." – p. 48 "Maharishi thus describes pure consciousness as a field of pure existence, pure *Being* – as *the Absolute*, the silent, eternal, nonchanging field that underlies *the relative*, the ever-changing world of space and time." – p. 48<br>"[In pure consciousness] [t]he mind is open to its total potential, an unlimited reservoir of creativity and intelligence." – p. 50 "One's universal, unbounded cosmic Self is now awake within itself." – p. 50<br>"Pure consciousness is the unified field, the source of natural law, the intelligence that manages the universe in perfect harmony." – p. 180<br>In Maharishi's understanding, "[Pure consciousness] is the ultimate essence of everything ..." – p. 264

	"The ancient Vedic tradition of knowledge, which Maharishi revived, identifies a single field of unity at the basis of life – a field of abstract pure consciousness. Pure consciousness, in this tradition, underlies not only all perceptions, thoughts, and feelings but also all forms and phenomena in the universe." – p. 437
Roth (2018)	"... a field of limitless creativity, intelligence and energy ..." – p. 17
Shear (1990b)	Shear quotes Maharishi Mahesh Yogi: "The source of thought, the source of creative energy, thus comes within the scope of the conscious mind and fills it with strength ... This is how the conscious mind is enlarged to its maximum capacity, embracing within its fold the source of creative intelligence and thus becoming infinitely powerful." – p. 227 Shear indicates that this is a case where an "ancient meditation [tradition]" has "[drawn] conclusions quite beyond those of our own analysis" (p. 226), and notes that "our own results obviously fall far short of such traditional claims of ... 'mind ... becoming infinitely powerful', etc." (p. 227).
Rosenthal (2016/2017)	Quoting Roth: "According to ancient meditation texts, this level is also the source of an individual's unbounded creativity, intelligence, energy, and happiness ... The texts call it the 'source of thought', 'pure consciousness' ..." – p. 259
Heaton and Travis (2014)	"... [T]he all-pervading field of pure consciousness, by virtue of being conscious, is awake to itself. This makes consciousness anywhere conscious of consciousness everywhere – every point of creation is conscious of the whole. Maharishi ... described this dynamic nature of consciousness as the basis of all creative processes in nature: '... The whole field of change emerges from this field of non-change ...'." – p. 24

2.5 Participant-based academic studies of the subjective experience

2.5.1 The Consciousness Integration Questionnaire

A strong focus in Rosenthal (2016/2017) is reporting the findings from a questionnaire completed by "607 meditators, referred to by various TM centers in the United States and South Africa" (p. 54). Rosenthal notes that "[t]here was a wide range of duration in TM practice [of the meditators], with a median of four years." (p. 54). The questionnaire was designed by "Fred Travis, Gerry Geer, and I, along with many other TM scholars" (p. 54). It comprised two subscales: the State of Consciousness scale and the Impact on Life scale (p. 54). Only one item in the State of Consciousness scale asks explicitly about the experience during TM: "While meditating, have you experienced periods of time that are different from ordinary waking experience – often silent and calm without thoughts?" – p. 269 Ninety-five percent of participants said yes (p. 55). The questionnaire also gave respondents the opportunity to describe their experiences, and Rosenthal (2016/2017) includes some of the descriptions. At p. 284 he notes as a limitation that: "... [T]he respondents cannot be said to be representative of TM practitioners as a whole. For example, they might be more diligent or interested in TM – or might have had greater benefits from it – than the average TM practitioner."

2.5.2 Travis and Pearson (2000)

Travis and Pearson (2000) includes a qualitative study investigating the "phenomenological correlates" of the TM goal-state/s, and Travis places heavy reliance on the findings in later publications (e.g., Arenander and Travis, 2004, pp. 120-121; Travis, 2006, pp. 25-26; Travis, 2014, p. 3; Travis et al., 2005, pp. 127-128; Travis and Shear, 2010a, p. 1115, 2010b, p. 1123). Participants were 52 college students, who had an average of 5.4 years of TM practice (p. 80). They were asked to describe "the fine details of [their] deepest experiences during [their] practice" (p. 80). "Three major themes emerged from ... analysis [of the responses]": "absence of space, time, or body sense" (described by 68% of participants), "peaceful" (32%) and "unbounded" (20%) (p. 81). In the Discussion section, the authors say: "'Time, space, and body sense' are the framework for understanding the waking experience. Specific qualities (color, shape, size, movement, *etc.*) are the content of waking experiences. During pure consciousness experiences, both the fundamental framework and the content of waking experience were reported to be absent." – p. 82 In the Results section, however, they do not comment on the reports of the absence of content (as distinct from the absence of space, time or body-sense), so it is not clear what percentage of participants provided those reports. It is also not clear what percentage of participants described the experiences as involving an absence of space, time *and* body sense, as opposed to the absence of merely one or two of those three experiential qualities. In discussing the study, Travis (2014, p. 3) states that: "All of [the participants'] reports were of a state where thinking, feeling, and individual intention were missing, but Self-awareness remained", however neither Travis and Pearson (2000) or Travis (2014) provide data summaries or analysis to support that claim.

2.5.3 Other

Some of the descriptions of individual meditators in the experts' texts are taken from academic studies.

2.6 Speed at which achieved

See also 1.5.2 ("The technique is quick to learn/perfect").

Pearson (2013)	Quoting a meditator describing their first session: "... I experienced a silent, inner state of no thoughts, just pure awareness and nothing else ..." – p. 29
Shear and Jevning (1999a)	"... [R]eports of [pure consciousness] are widespread among [TM] meditators ... often within the first few weeks of practice ..." – p. 197
Shear (2006a)	Referring to a range of meditation practices (including TM) that Shear argues are designed to achieve pure consciousness: "People might practice a meditation procedure for a long time without getting the deep experiences we have been discussing." – p. 267 The "deep experiences" he refers to may include pure happiness, pure consciousness, or higher states of consciousness said to be experienced in daily life.
Shear (2006c)	"[TM] is an effortless mental technique that quickly and easily enables any ordinary person to experience [pure consciousness]." – p. 23<br>"... allows the awareness to settle down, quickly and easily, to [pure consciousness]." – p. 24<br>"... [I]t is quite common for people to begin to experience ... pure consciousness... within the first few days of learning it." – p. 25<br>"... [T]he growth of consciousness the average person can gain quickly from the practice of the basic TM technique is remarkable ..." – p. 42
Shear (2011b)	"The whole process is so effortless and natural that ... even new meditators usually taste the depths of inner silence very quickly, often within the first few days of beginning the practice." – p. 53
Rosenthal (2016/2017)	"A few people dive into transcendence the very first time they learn to meditate ... but for most others the state comes more slowly. I fell into the second category, and it was several weeks before I even realized I was transcending." – p. 36
Rosenthal (2011/2012)	"After a few years of practice, it has allowed me to enter a place inside my mind that is difficult to describe with any better word than *transcendence*." – p. 8<br>"After a month or two, finally, I *got* it. For a brief moment, I felt what it was to transcend, and I knew I could do it again." – p. 25<br>"It is the profoundly relaxed alertness free of specific thoughts, along with the loss of personal boundaries of time or space, that are the essential elements of transcendence. Once people get to that point, which usually happens within the first month or two of learning ..." – pp. 42-43<br>"During her third TM session, Nicole transcended for the first time – a deep, blissful experience, lasting about ten to fifteen minutes." – p. 50
Travis (2006)	"... [I]ndividuals quickly master transcending during TM practice – after a few months practice ..." – p. 24

2.7 Features

2.7.1 Recognize in retrospect

Shear (1990b)	"It can of course be asked what the experience of pure consciousness ... is remembered *as*? The answer appears to be 'Not *as* anything'. By all reports one remembers simply that one was; one was awake, without being aware of any thing, without even noticing *that* one was aware. Yet one was aware, awake, not unconscious – *this* one remembers. In memory it is clearly distinguished from unconsciousness, as for, example, from the 'blackout' of deep sleep; it is clearly distinguished from unconsciousness because one *remembers*, one remembers being awake, just simply consciously *being*." – p. 103

Shear (1996b)	Shear p. 371 discusses looking within to investigate the nature of the state being experienced. "... [S]ince looking is a mental activity, the very activity of looking itself keeps the mind stirred up, and pure consciousness ... becomes isolated only when all the activity of mind settles down into the absolutely settled state of *samadhi*. Naturally then, it is only to be expected that one should be highly unlikely to be able to discover what completely settled (pure) consciousness is like as long as such activity continues." – p. 371
Shear (1995/1997b)	"... [I]t *cannot* be found by *searching* for it, because ... it must be devoid of all discrete activities and contents, including in particular the activity of searching for it and the experience of any imaginable thing searched for." – p. 370<br>"This experience, in order to be remembered at all, would [seem] to have to have *some* kind of quale associated with it ..." – p. 373
Shear (2004)	"... [O]ne finds oneself settled into a completely objectless state of awareness that one becomes intellectually conscious of only *afterward*, as one returns to ordinary mental activity." – p. 86
Shear (2006c)	"... [I]t is not obvious where to look to find [the] experience. Indeed, any *where* or direction that one looks is only likely to be misleading, since the experience lies entirely outside of the realm of ordinary experience. Furthermore, the very *activity of looking* will be counterproductive, since ... the experience occurs only when all activity ceases." – p. 31<br>"... [T]he fact that one can look back and remember [the experience] differentiates [it] readily from deep, dreamless sleep ..." – p. 34<br>"Dreamless sleep is tantamount to unconsciousness, and cannot even be remembered. Pure consciousness can be experienced and remembered." – p. 35
Rosenthal (2016/2017)	"... [I]f you're asking yourself whether you're transcending, you're not." – p. 100
Travis and Pearson (2000)	"... [T]he knower is conscious through the experience, and can, afterwards, describe it." – p. 79<br>"... [T]he subjects are awake during this state and they can describe [it] afterwards ..." – p. 82

2.7.2 Cannot say much about it

See also 2.7.15 ("Limited or no abstract content") and 2.7.49 ("Description in words is limited").

Shear (1990b)	"It can of course be asked what the experience of pure consciousness ... is remembered *as*? The answer appears to be 'Not *as* anything'. By all reports one remembers simply that one was; one was awake, without being aware of any thing, without even noticing *that* one was aware. Yet one was aware, awake, not unconscious – *this* one remembers. In memory it is clearly distinguished from unconsciousness, as for, example, from the 'blackout' of deep sleep; it is clearly distinguished from unconsciousness because one *remembers*, one remembers being awake, just simply consciously *being*." – p. 103
Shear (1995/1997b)	"This experience, in order to be remembered at all, would [seem] to have to have *some* kind of quale associated with it, even if it is nothing more than some sense of an existing nothingness – a sort of 'void' quale. But logically it would have to be *the simplest possible* quale – one tantamount to a phenomenal nothing." – p. 373
Shear (1998/1999)	"We should first note that nothing much can be said about the experience itself. For its identifying characteristic is that it is devoid of all empirical content ..." – p. 412 "What then is the experience *like*? It is not *like* anything. What is it remembered *as*? Not *as* anything. Nevertheless it *is* remembered. One remembers having the experience, but there is nothing 'to' it. No content, shape, structure, or anything else." – p. 413
Shear and Jevning (1999a)	"What is this experience like? By all accounts it is not *like* anything. For it has no content in it at all to make it more like any one thing than any other. And inasmuch as it contains no phenomenal content at all, no colours, sounds, thoughts, anticipations, etc. – or even any subjective manifold where such content could be located – whatever one can *imagine* is necessarily irrelevant to it. In short, the experience is simply awareness itself, unimaginable and ... ungraspable." – p. 195
Shear (2002)	"Not much can be said about the internal nature of ... pure consciousness, for phenomenologically, by all reports, there is nothing there ... to talk about at all." – p. 363

Shear (2004)	"... [Pure consciousness] is ... utterly abstract. So abstract, in fact, that [its] phenomenological nature is *inconceivable*. For no images or empirical contents whatsoever – not even that of a spatio-temporal manifold for content to appear in – seem relevant to it at all." – p. 87
Shear (2006b)	"... [T]he terms of our ordinary language, such as 'red', 'blue', 'big', 'small', 'left', 'right', 'here', and 'there' do not apply to [the experience] at all. And it cannot even be imagined, for anything we can imagine has properties that are irrelevant to this unique experience. What then is the experience *like*? It is not *like* anything. It is just itself." – p. xviii "Concrete terms and images thus appear completely incapable of describing this experience. As a result, meditation traditions regularly use a variety of highly abstract terms when referring to it. But even these abstract terms appear unable to convey its nature properly." – p. xviii Shear p. xix refers to the experience as "different ... from anything we can conceive or imagine".
Shear (2007)	"What then is the experience like? By all accounts, it is not *like* anything. One can have it and remember it – one knows that one was not asleep. But one does not remember it *as* anything at all. It is just *itself* – unimaginable and indescribable." – p. 700<br>"The experience itself is extraordinarily abstract. Indeed it is the logical ultimate of abstraction, since by all accounts it is what remains after everything that can possibly be removed from experience has been removed, while one nevertheless remains awake." – p. 700<br>"... extreme abstractness ..." – p. 700
Shear (2011a)	"... [I]t is *impossible* to imagine. For if anything we can imagine were in it, it would not be this completely empty experience." – p. 140<br>Quoting a meditator having what Shear describes as a pure consciousness experience: "My meditations are characterized by the experience of 'no experience' ... I just remain in the Absolute for the entire sitting and nothing else seems to happen, other than the feeling of bliss permeating me completely." – pp. 142-143
Shear (2011b)	"The experience ... has absolutely nothing in it. This makes it easy to describe. But it also makes it unimaginable. For because it is devoid of all objects of experience whatsoever, anything one can imagine is external, and therefore irrelevant, to it." – p. 52 "... [T]he closest one can come [to imagining it] is to imagine oneself as disembodied awareness in empty space, but this is misleading, for even phenomenological space is absent from the experience ..." – p. 52
Shear (2014a)	"It is ... completely *unimaginable*, for anything one can imagine has phenomenal content, and the identifying characteristic of this experience is the absence of all such content." – p. 60
Travis et al. (2005)	Quoting a meditator: "... [T]here is nothing I can report about this state. I am completely full, vibrant, and alive, but I am completely still. It's absolute silence." – p. 128

2.7.3 Conscious

See 2.7.1 ("Recognize in retrospect") and 2.7.4 ("Pure consciousness/awareness").

Rosenthal (2016/2017)	As an example of an exception to the "standard aspects of the [state]", Rosenthal p. 36 notes: "... [S]ome people report going into a state of awareness where awareness of any sort disappears – until consciousness returns – when, lo and behold! twenty minutes have passed. I know that sounds pretty much like a nap, but having experienced such ... states myself, I can say that when I emerge, I do not feel as if I have taken a nap. Although I cannot swear no z's slipped in, I am clearheaded in a non-postnap way, wide awake, and ready to get on with the day. Others have reported similar experiences."<br>Quoting Ray Dalio, founder and president of the hedge fund Bridgewater Associates: "You go into a different state – neither conscious nor unconscious. When you're meditating, you're just not aware. Everything disappears, in a sense. But unlike when you're sleeping, if a pin drops all of a sudden, it can reverberate through you; it's shocking." – pp. 186-187
Travis (2006)	"[Pure consciousness] is a state of awareness in that the knower does not black out. There is no gap in time. Being or pure self-awareness is maintained." – p. 24

2.7.4 Pure consciousness/awareness

Pearson (2013)	Quoting a meditator describing their first session: "... I experienced a silent, inner state of no thoughts, just pure awareness and nothing else ..." – p. 29<br>"... a state of *pure* consciousness, consciousness aware only of its own unbounded nature." – pp. 29-30

	"... experience of consciousness in its pure form, awake to itself alone – unbounded pure awareness, our innermost Self." – p. 41<br>"[In pure consciousness] [t]he sea of consciousness is aware only of itself, unmixed with anything else. Maharishi calls this a state of *pure consciousness*, pure awareness, pure wakefulness." – p. 44<br>Quoting Maharishi Mahesh Yogi: "... just pure consciousness aware of its own unbounded nature." – p. 45<br>"One experiences consciousness in its pure state ..." – p. 45<br>"[Pure consciousness] is self-referral, absorbed in itself, aware of itself alone, its own object of perception." – p. 50<br>Quoting a meditator: "... I am just aware of awareness, you could say, wide awake inside but not thinking." – p. 52
Shear (1990b)	"Consciousness alone remains as the experience." – p. 102
Shear (1995/1997b)	"... the fundamental nature of human consciousness." – p. 370<br>"... bare consciousness itself ..." – p. 373
Shear (1998/1999)	"... [I]t is often referred to as 'pure consciousness' (since there had to be consciousness for there to be a rememberable experience) ..." – p. 413
Shear and Jevning (1999a)	"The experience would appear to be one of consciousness alone by itself – pure, silent, and empty of all 'phenomenal' objects." – p. 194<br>"... pure, unmanifest, absolutely objectless consciousness." – p. 195<br>"... [T]he experience is simply awareness itself ..." – p. 195
Shear (2002)	"... a state in which consciousness exists alone, completely by itself ..." – p. 363
Shear (2006b)	"[The experience] is commonly referred to as being of 'pure consciousness', since one had to *be conscious* to have and remember it, and there was nothing *else* there. This usage can be confusing, however, for consciousness is usually thought of as having some *object* or content that it is *of*, and there is no object or any sense of 'of' here at all." – p. xviii
Shear (2006c)	"... pure consciousness resting in its own nature ..." – p. 33<br>"... consciousness itself in its pure, simplest state." – p. 33
Shear (2007)	"[The experience of pure consciousness] suggests a simple ... definition of 'consciousness' as 'that which remains when all phenomenal content is removed and one remains awake'." – p. 708
Shear (2014a)	"Vedanta and Yoga ... refer to [the experience] as 'pure consciousness', for one can hardly be thought to remember something where one's consciousness was not present." – p. 60
Shear (2014b)	"[Pure consciousness] as described seems very much like experiencing nothingness itself, or even being nothingness itself. Nevertheless it is, as reported, a conscious mental even that, unlike unconsciousness can actually be remembered." – p. 213
Travis and Pearson (2000)	"... 'consciousness itself' without particular consciousness." – p. 79
Arenander and Travis (2004)	"The content of pure consciousness is awareness itself." – p. 113
Heaton and Travis (2014)	"... [C]onsciousness is awake only to itself." – p. 25

2.7.5 Awake, wakefulness

Pearson (2013)	"... uniquely different from the familiar states of waking, dreaming, and sleeping." – p. 21<br>"... remaining perfectly alert and awake" – p. 25<br>"... an ocean of pure consciousness, a limitless sea of pure wakefulness." – p. 25<br>"... restfully alert state ..." – p. 30<br>"[The mind] can become calm, quiet, silent, while remaining awake. This is [pure consciousness]." – p. 44

	"Your mind is awake but silent and serene." – p. 44<br>Pearson p. 44, 50 also refers to being awake.<br>"... *pure consciousness*, pure awareness, pure wakefulness." – p. 44<br>"Unlike the ordinary waking state, in which the mind is awake only at its surface, the mind now is awake to its depth." – p. 45<br>"[Pure consciousness] is still, silent, an ocean of pure wakefulness ..." – p. 50<br>Quoting a meditator: "... I am in an indescribable place. I am alone with my Self, and I have never been so clearly and entirely and fully awake. There is nothing but That – no ocean [of consciousness], no me, no anything, but totality. And I am fully awake to it." – p. 53<br>"... [T]he mind is awake and alert." – p. 175
Roth (2018)	"... [D]eep within is a level that is calm yet alert; silent yet wide awake." – p. 17<br>"... TM provides more than just rest. It produces deep rest and inner alertness, together – or ... a unique state of restful alertness." – p. 19<br>"What does it feel like to meditate? ... Mentally, it's calming yet awakening ..." – p. 58<br>"You are just settling down, physically and mentally, while remaining wide awake inside." – p. 65<br>"... I was fully aware, fully awake." – p. 152<br>Quoting the singer Katy Perry: "I go to this incredible place where I'm not even thinking anything. And yes I'm awake." – p. 164
Shear (1990b)	"... a state of inner wakefulness ..." – p. 100<br>"... leaving the mind awake, yet with no object of experience, that is, awake in the experience of pure consciousness." – p. 101<br>Shear p. 102 also refers to being awake.<br>"It can of course be asked what the experience of pure consciousness ... is remembered *as*? The answer appears to be 'Not *as* anything'. By all reports one remembers simply that one was; one was awake, without being aware of any thing, without even noticing *that* one was aware. Yet one was aware, awake, not unconscious – *this* one remembers. In memory it is clearly distinguished from unconsciousness, as for, example, from the 'blackout' of deep sleep; it is clearly distinguished from unconsciousness because one *remembers*, one remembers being awake, just simply consciously *being*." – p. 103
Shear and Jevning (1999a)	"... a purely contentless wakeful mental state ..." – p. 194<br>"... the mind ... remaining alert ... silent and fully awake inside ..." – pp. 194-195
Shear (2006c)	"... the mind wide awake ..." – p. 32
Shear (2007)	"[The experience of pure consciousness] suggests a simple ... definition of 'consciousness' as 'that which remains when all phenomenal content is removed and one remains awake'." – p. 708
Rosenthal (2016/2017)	"How strange it is to be alert and peaceful at the same time ..." – p. 35
Rosenthal (2011/2012)	"... alert but focused on nothing in particular ..." – p. 42<br>"It is the profoundly relaxed alertness free of specific thoughts, along with the loss of personal boundaries of time or space, that are the essential elements of transcendence." – pp. 42-43<br>Quoting actor Laura Dern: "You don't fall asleep but it almost feels like a nap because you're in that dreamy state." – p. 230<br>Quoting Roth: "... a state of 'restful alertness' ..." (p. 276); "... [T]here is a level, deep within the mind, that is ever silent, settled, fully expanded, and fully alert" (p. 277).
Travis and Pearson (2000)	Quoting a participant: "... [A] couple of times per week I experience deep, unbounded silence, during which I am completely aware and awake ..." – p. 81<br>"... [T]he subjects are awake during this state ..." – p. 82
Travis et al. (2005)	Quoting a meditator: "I am completely full, vibrant, and alive, but I am completely still. It's absolute silence." – p. 128<br>"It is being awake in the midst of silence." – p. 128
Travis and Parim (2017)	"In this research, transcendence is described as a perfectly peaceful state in which the mind is very awake, but still ..." – p. 87

Travis (2018)	"The mind is wide awake ..." – p. 36

2.7.6 Non-duality

Pearson (2013)	Quoting Maharishi Mahesh Yogi: "[Pure consciousness] is ... beyond the division of subject and object." – p. 45<br>"Every human experience, Maharishi observes, has three fundamental components: a knower, a known, and a process of knowing linking the two – an experiencer, an object of experience, and a process of experiencing ..." – p. 47 "In [pure consciousness], [the three components] become identical – consciousness becomes its own object of experience. No perceptions, thoughts, or feelings intervene between knower and known. There is nothing to bridge because there is no longer any distinction between subject and object – the subject *is* the object." – p. 47 "Thus Maharishi describes the fourth state as *self-referral consciousness* – consciousness refers only to itself. The ordinary waking state, in contrast, is *object-referral*, focused on objects, which includes thoughts and feelings." – p. 47<br>"[In pure consciousness] [c]onsciousness is unified. Pure consciousness is the knower of itself. Knower, knowing, and known are unified." – p. 50<br>Quoting a meditator: "It felt as if I were floating in Brahman [totality]. But that expression suggests two things: Brahman and me. Yet there is only one thing." – p. 53
Shear (1990b)	"... transcendental pure awareness completely devoid of all objects, sense of individuality and object-orientation ..." – p. 43<br>Shear p. 44 describes how moving beyond the pure individuality stage involves "... transcending all distinctions between subject and object." – p. 44<br>"According to Maharishi ... the ordinary structure of experience is: subject is conscious of object, or 'I – conscious – it'. During the practice of [TM], however, the intensity and specificity of the 'it's gradually (and spontaneously) diminish in intensity until the subject finds himself (or herself) alone by himself, simply self conscious, without any object of awareness, yet definitely awake." – p. 102
Shear (2006b)	"... [E]very experience would seem to require an experiencer, and there are no *two* things present here ..." – p. xviii
Shear (2014a)	"Even bare subject-object orientation ... [is] absent." – p. 60
Rosenthal (2016/2017)	"Usually, when we think about something, we engage in a process called 'duality', in which the person thinking and the thought itself seem like two separate entities ... In pure consciousness, however, no such division occurs." – p. 35 "When you transcend, I , thou, and it all blend. There is a feeling of 'oneness'." – p. 35<br>Rosenthal p. 234 refers to the "concept of nonduality in which the experiencer, the object of the experience, and the experience itself merge into unity". "Since returning to TM ... experience has taught me that transcendence is in fact a state of nonduality in which thinker, thought, and object of the thought lose their boundaries and merge into pure awareness." – p. 234
Rosenthal (2011/2012)	"During TM practice, the subject-object relation that defines customary experiences is transcended ... In TM, the object of experience fades away – you use the mantra to lose it." – p. 288
Travis et al. (2005)	"The three distinct elements of ordinary waking experience (knower, known, and the process of knowing) have become one. Maharishi ... explains: 'When we have transcended the field of the experience of the subtlest object, the experiencer is left by himself without an experience, without an object of experience, and without the process of experiencing' ..." – p. 126
Travis and Shear (2010a)	"During TM practice, the subject-object relation that defines customary experience is transcended." – p. 1116 "... [T]he object of experience fades away – you use the mantra to lose it. When the mantra disappears, the subject, or the experiencer, as Maharishi puts it, 'finds himself awake to his/her own existence ..." – p. 1116
Travis (2011)	"Pure consciousness is a non-dual state of awareness – the self is both the subject and object of awareness." – p. 225 "In pure consciousness, the three-part structure of experiencer, object of experience, and process linking the two has been transcended." – p. 225

2.7.7 Stillness

Pearson (2013)	"... a state of perfect stillness, beyond all perceptions, thoughts, and feelings ..." – p. 25<br>"[Pure consciousness] is still, silent ..." – p. 50
Roth (2018)	The book is titled "Strength in Stillness".<br>"... to *transcend* – to effortlessly access the deep stillness that lies within every human being ..." – p. 5<br>"... state of inner stillness at the deepest level of awareness ..." – p. 17<br>Roth pp. 34, 35, 42 also refers to stillness.<br>"... pure stillness ..." – p. 42<br>Quoting the singer Katy Perry: "Thoughts will come and go, and it will be five minutes before I can get to that stillness. I go to this incredible place where I'm not even thinking anything." – p. 164
Rosenthal (2016/2017)	"... stillness, expansiveness, boundlessness, and peace ..." – p. 5<br>"... utterly still ..." – p. 30<br>"What, then, are the usual elements of transcendence? That was the question I asked a group of students, at Loyola University's Stritch School of Medicine in Chicago, who had learned to meditate. They replied with gusto: stillness, quiet, no boundaries, no thoughts, and bliss. In short, they captured the essential spirit of the state." – p. 31
Travis et al. (2005)	Quoting a meditator: "... I am completely still. It's absolute silence." – p. 128
Travis and Parim (2017)	"In this research, transcendence is described as a perfectly peaceful state in which the mind is very awake, but still ..." – p. 87

2.7.8 Silence, quietness

Pearson (2013)	Quoting a meditator describing their first session: "... I experienced a silent, inner state of no thoughts, just pure awareness and nothing else ..." – p. 29<br>"... a state of deep inner silence and peace ..." – p. 29<br>"[The mind] can become calm, quiet, silent, while remaining awake. This is [pure consciousness]." – p. 44<br>"Your mind is awake but silent and serene." – p. 44<br>"[Pure consciousness] is the most simple and natural experience a person can have – the experience of awareness in its most quiet state." – p. 49<br>"[Pure consciousness] is still, silent ..." – p. 50<br>Quoting a meditator: "I will never forget the first experience I had of the [TM] technique ... The movement of my awareness from the active level ... to the field of silence within myself ... was like diving into a pond of pure joy." – p. 51<br>Quoting a meditator: "[M]y mind settles down, thoughts become less and then suddenly all thought activity ceases and I slip into an unbounded ocean of awareness which is pure, quiet, unexcited ..." – p. 52<br>Quoting a meditator: "... I sometimes reach a state of complete silence ..." – p. 52<br>"... an inner sanctuary of infinite silence, peace, and joy." – p. 175
Roth (2018)	"... [D]eep within is a level that is calm yet alert; silent yet wide awake." – p. 17<br>"It is your own quiet inner self ..." – p. 17<br>"... the quietest, deepest level ..." – p. 34<br>"... quiet, peaceful, transcendent state of awareness." – p. 37
Shear and Jevning (1999a)	"The experience would appear to be one of consciousness alone by itself – pure, silent, and empty of all 'phenomenal' objects." – p. 194<br>"... silent and fully awake inside ..." – p. 195<br>"... alert silence of pure consciousness ..." – p. 205<br>"... simplest, non-active, completely silent state [of consciousness] ..." – p. 205
Shear (2006b)	"... *absolute*, *pure silence*." – p. xviii
Shear (2006c)	"... completely silent state ..." – p. 26
Rosenthal (2016/2017)	"... peace and quiet." – p. 31

	"What, then, are the usual elements of transcendence? That was the question I asked a group of students, at Loyola University's Stritch School of Medicine in Chicago, who had learned to meditate. They replied with gusto: stillness, quiet, no boundaries, no thoughts, and bliss. In short, they captured the essential spirit of the state." – p. 31<br>"It is quiet there. Still. Peaceful. Thoughts may still come and go but sooner or later ... silence ... all thoughts have gone." – p. 35
Rosenthal (2011/2012)	"... I use the mantra in a way that allows my mind to settle into quietude." – p. 18<br>"As I continue to meditate, all that mental noise quiets, and I welcome the silence." – p. 18
Travis and Pearson (2000)	Quoting a participant: "... [A] couple of times per week I experience deep, unbounded silence, during which I am completely aware and awake, but no thoughts are present." – p. 81
Arenander and Travis (2004)	"... complete silence ..." – p. 114
Travis et al. (2005)	Quoting a meditator: "... I am completely still. It's absolute silence." – p. 128<br>"It is being awake in the midst of silence." – p. 128
Travis (2009)	"This silent interiority of the mind has been called pure consciousness." – p. 28

2.7.9 Dynamic

Pearson (2013)	"In the early stages of transcending, one experiences pure consciousness as silent, 'flat'. But as one becomes increasingly familiar with the experience ... one can experience, within this sea of silence, the internal, unmanifest reverberations, the inner dynamics of consciousness. To phrase it more accurately (since there is no duality at this level), *consciousness experiences itself* as infinitely silent and infinitely dynamic, both together." – p. 444

2.7.10 Clearness

Pearson (2013)	"... crystal clear consciousness." – p. 26
Roth (2018)	Quoting Shane Anselmi, a meditator: "[The practice] is something I look forward to because it brings a relaxed peace; a sense of joy, calm and clarity ..." – pp. 66-67<br>Quoting Deborah-lee Furness: "... I like the analogy of a glass of water ... When you're stressed ... [your mind is] kind of cloudy. And after I meditate, all that sinks to the bottom, and the water is clear ..." – p. 78
Shear (2006b)	"The experience is held to occur when the mind is unusually clear and settled ..." – p. xix
Rosenthal (2011/2012)	Quoting Craig Pearson, describing his own experience: "There's definitely a diving deep within, a settling down of mental activity and deep integration. Do I experience clear, pure unbounded awareness in some of the extraordinary ways I've discovered it described by some of the great people through history? No, I've only had that experience a few times in my life. But there are people who sit around me in the Golden Dome [of the Maharishi University of Management in Iowa] who have that experience on a regular basis. And with continuing practice, these are the kinds of experiences everyone can have." – p. 42

2.7.11 Purity
See also 2.7.4 ("Pure consciousness/awareness").

Pearson (2013)	Quoting a meditator: "... I slip into an unbounded ocean of awareness which is pure, quiet, unexcited ..." – p. 52
Rosenthal (2011/2012)	Quoting Craig Pearson, describing his own experience: "There's definitely a diving deep within, a settling down of mental activity and deep integration. Do I experience clear, pure unbounded awareness in some of the extraordinary ways I've discovered it described by some of the great people through history? No, I've only had that experience a few times in my life. But there are people who sit around me in the Golden Dome [of the Maharishi University of Management in Iowa] who have that experience on a regular basis. And with continuing practice, these are the kinds of experiences everyone can have." – p. 42

2.7.12 Relaxation

Pearson (2013)	Quoting a meditator: "... [In the state] I feel relaxed." – p. 52
Roth (2018)	"... [Y]ou will know you are meditating properly in [the] beginning days if ... you feel some deep relaxation during the practice ..." – p. 64<br>Quoting Shane Anselmi, a meditator: "[The practice] is something I look forward to because it brings a relaxed peace; a sense of joy, calm and clarity ..." – pp. 66-67<br>Describing his first session of TM: "As [the teacher] led me through the beginning steps ... I felt my mind and body sinking into a state of deep relaxation ... [W]ithin seconds of my first experience meditating, I felt this wave of physiological peace come over me." – p. 152
Shear (1998/1999)	"... TM's use of *mantras* (mental repetition of specific sounds to let one relax utterly, while remaining awake)." – p. 413
Rosenthal (2016/2017)	"... [T]he sense of oneness is blissful and relaxing." – p. 35
Rosenthal (2011/2012)	"It is the profoundly relaxed alertness free of specific thoughts, along with the loss of personal boundaries of time or space, that are the essential elements of transcendence." – pp. 42-43

2.7.13 Mental rest

In general, when the experts speak about rest they refer to bodily rest, rather than to mental rest specifically (see, e.g.: the Pearson, 2013 references to stress and rest in 2.7.27, "No sense of anxiety"; Roth, 2018, pp. 18-19; and Shear, 2001/2002, p. 280). On occasion, they use terms such as "restful alertness" without indicating whether it refers to bodily or mental rest (e.g., Pearson, 2013, p. 30). A limited number statements refer (or arguably refer) specifically to mental rest, and these are extracted below.

Roth (2018)	"Jerry Seinfeld told me he's felt the effects of deep rest so profoundly that he likens TM to a phone charger for the mind." – p. 91<br>Quoting the singer Katy Perry: "Fundamentally, TM is the deepest rest I ever get." – p. 163
Shear (2006c)	"When the mind settles within to this completely restful state, the body also settles down and gains a unique state of rest ..." – p. 24
Rosenthal (2016/2017)	"Both body and mind can rest in a special way – different from sleep or other types of rest." – pp. 30-31<br>Quoting a patient, Moira: "... [M]ostly I experience a wonderful, restful feeling." – p. 38

2.7.14 Contentless, reduced mental activity

See also 2.7.15 ("Limited or no abstract content"), 2.7.22 ("Cycling"), and 2.7.23 ("Increasing length").

Pearson (2013)	Quoting a meditator describing their first session: "... I experienced a silent, inner state of no thoughts, just pure awareness and nothing else ..." – p. 29<br>"Your consciousness is free of content, awake but without perceptions, thoughts, and feelings." – p. 44<br>Quoting Maharishi Mahesh Yogi: "[TM] ... [allows] the excitations of the mind gradually to settle down until the least excited state of mind is reached." – p. 45<br>"[Pure consciousness] ... [has] no content other than its own nonchanging, unbounded nature." – p. 50<br>Quoting a meditator: "I have had the experience of transcending all activity ..." – p. 52<br>Quoting a meditator: "In this state, I am not aware of any thought or any thing ..." – p. 52<br>Quoting a client discussing pure consciousness as encountered in daily life: "There was only silence, crystal-clear pure consciousness. There was nothing else but 'That' – no thoughts, no intellect, no ego, no experience of some 'thing'." – p. 186<br>"... [In pure consciousness] there is no 'content' ... One transcends the relative contents of consciousness – concepts, values, language, thought, memory, emotion – to experience consciousness in its pure form ..." – p. 456
Roth (2018)	"People ask how they will know when the twenty minutes of the meditation are up. I smile and say 'Look at your watch!' This is not hypnosis, and you're not in a zone. You are just settling down, physically and mentally, while remaining wide awake inside." – p. 65

Shear (1990b)	"... transcendental pure awareness completely devoid of all objects, sense of individuality and object-orientation ..." – p. 43<br>"... completely objectless ..." – p. 46<br>"... awareness independent of all objective content ..." – p. 46<br>Having referred to techniques that keep the mind active, Shear describes pure consciousness as "completely non-active" (p. 100).<br>"... no object of experience ..." – p. 101<br>Shear p. 102 also refers to the objectlessness.<br>"... [According to] the common descriptions the experience ... is defined as being totally devoid of any empirical, spatiotemporally identifiable content ..." – p. 102 "[This phrase] refers to ... an experience which is uniquely identifiable precisely *by* its total absence of any empirical content." – pp. 102-103<br>"It can of course be asked what the experience of pure consciousness ... is remembered *as*? The answer appears to be 'Not *as* anything'. By all reports one remembers simply that one was; one was awake, without being aware of any thing, without even noticing *that* one was aware." – p. 103<br>"[T]he experience ... cannot be said to be an experience of any determinate thing and cannot properly be described in terms of empirical qualities (colors, feelings, spatio and temporal relationships, meanings, etc.) ..." – pp. 103-104<br>"... [P]ure consciousness can be defined uniquely as that experience which has absolutely no identifiable empirical qualities within it, that is, which is devoid of identifiable spatio-temporal content." – p. 104<br>"... [T]his experience has no parts or components ..." – p. 105<br>"... [A]ll the objective contents of experience ... fade out and disappear ..." – p. 115<br>"... [Pure consciousness] has no content *in* it at all ..." – p. 137<br>"... the least excited ... state of consciousness ..." – p. 204<br>"... qualityless pure consciousness ..." – p. 211
Shear (1994)	Referring to "introvertive mystical experience", which Shear presents as equivalent to pure consciousness: "[T]he defining characteristic of this experience ... is that it contains no empirical content whatsoever." – p. 322
Shear (1996b)	"... [I]ts defining characteristic is the complete absence of everything that we ordinarily take to be objects of experience. That is, it is defined as being utterly devoid of all thoughts, sensations, and empirical qualities, devoid even of the sense of there being any spatio-temporal manifold present in which objects and qualities could be located at all." – p. 366<br>"[A]ll the fluctuations of the mind ... settle down ..." – p. 367<br>Shear p. 369 defines "empirical content" as "content which could be either present or absent".<br>"... [P]ure consciousness ... becomes isolated only when all the activity of mind settles down ..." – p. 371
Shear (1995/1997b)	"... consciousness *devoid of all its discrete activities and contents* ..." – p. 370<br>"... removing attention from all phenomenal *objects of* consciousness ... leaving *consciousness* alone by itself." – p. 370<br>"... devoid of all determinate mental content (sensations, intentions, thoughts, and even any subjective spatio-temporal manifold in which such objects of awareness could appear)." – p. 372
Shear (1998/1999)	"... [I]ts identifying characteristic is that it is devoid of all empirical content. That is ... the experience has absolutely no discernible sensations, perceptions, images, thoughts, etc., in it. Indeed, by all reports, it does not even have a spatio-temporal manifold for such phenomenological objects to be located in." – pp. 412-413<br>"[I]t is often referred to as ... 'pure void' (since it is utterly devoid of content)." – p. 413<br>TM is "designed to allow one's attention to withdraw from all ordinary contents of awareness ... the idea being that once all phenomenal objects ... are gone from one's awareness – while one nevertheless stays awake – what remains can only be awareness or consciousness itself." (p. 413).<br>"... absolutely devoid of phenomenal content." – p. 414
Shear and Jevning (1999a)	The authors refer to "a procedure that could allow the mind to divert its attention from all mental content whatsoever" (p. 191).<br>"... a purely contentless wakeful mental state ..." – p. 194<br>"The experience would appear to be one of consciousness alone by itself – pure, silent, and empty of all 'phenomenal' objects." – p. 194<br>"... [I]t contains no phenomenal content at all, no colours, sounds, thoughts, anticipations, etc. – or even any subjective manifold where such content could be located ..." – p. 195

	"... its defining characteristic – the complete absence of empirical phenomenal content ..." – p. 195 "... simplest, non-active, completely silent state [of consciousness] ..." – p. 205
Shear (2002)	"... completely devoid of all determinate content whatsoever. That is ... a state in which consciousness exists alone, completely by itself, with no discernable phenomenological objects or qualities in it at all." – p. 363
Shear (2001/2002)	"... allow the meditator's mental activity to diminish and cease altogether in an empirically qualityless state ... referred to as 'pure consciousness'..." – p. 280
Shear (2006b)	"This state is quite unique. For it has no content in it at all – no perceptions, feelings, thoughts, or anything else – not even, indeed, any time or space in which such things could be located." – p. xviii "Since the experience appears to be devoid of all content, it is also often referred to as pure 'emptiness'. But ... there is no *sense of* emptiness in it ..." – pp. xviii-xix
Shear (2006c)	"... undisturbed by thoughts, perceptions, and other mental activity ..." – p. 24 "... no images, sensations, thoughts, feelings, or any of the other contents of experience we are ordinarily familiar with." – p. 33
Shear (2007)	"The defining characteristic of [pure consciousness] is the *complete absence* of all sounds, tastes, thoughts, feelings, images, and any thing else that one can ever imagine." – p. 700 "... [T]radition after tradition emphasizes that all thinking and concepts ... have to be left behind before the experience of pure consciousness, *as it is in and by itself*, can emerge with clarity." – p. 700 "... [The experience of pure consciousness] suggests a simple ... definition of 'consciousness' as 'that which remains when all phenomenal content is removed and one remains awake'." – p. 708
Shear (2011a)	"... [The experience] has nothing in it [at] all – no perception of objects, no colors or sounds, no feelings or emotions, no thoughts." – p. 140 "Buddhist traditions generally refer to [the experience] as 'emptiness', reflecting the fact that it is empty of everything that can be imagined." – p. 140
Shear (2011b)	"... [P]ure consciousness/emptiness is completely devoid of all perceptions, sensations, thought, will, and other identifiable phenomenal content ..." – p. 53
Shear (2014a)	"... [Pure consciousness] contains no phenomenal content at all." – p. 60 "... completely devoid of phenomenal content ..." – p. 60
Shear (2014b)	"[Pure consciousness] as described seems very much like experiencing nothingness itself, or even being nothingness itself. Nevertheless it is, as reported, a conscious mental event that, unlike unconsciousness, can actually be remembered. This experience, I would think, is the one that can most reasonably be referred to as *completely* empty." – p. 213
Rosenthal (2016/2017)	"One's sense of self dissolves into a pleasant emptiness, which eventually fills up again with vibrant thoughts, feelings, and all the bric-a-brac of mental life ..." – p. 35 "Just as our thoughts come and go, so (at times) do other boundaries, such as our sense of time and space." – p. 35
Travis and Pearson (2000)	"Pure consciousness is 'pure' in the sense that it is free from the processes and contents of knowing." – p. 79 "The 'content' of pure consciousness is self-awareness." – p. 79 "The subjective descriptions of pure consciousness [by the participants] portray a state of *self-awareness ... without waking processing and contents*. Pure consciousness [appears] to be self-awareness isolated from the processes and objects of experience." – p. 82
Arenander and Travis (2004)	"... devoid of mental content." – p. 113 "The content of pure consciousness is awareness itself." – p. 113
Travis et al. (2005)	"Subjects report [pure consciousness] during TM practice when mental activities cease." – p. 131
Travis and Shear (2010b)	"... [T]he basic non-dual experience ('pure consciousness' ...) is devoid of phenomenological content ..." – p. 1122 "... [A]ll phenomenal content is absent ..." – p. 1122

Travis (2011)	"... a state of consciousness ... [where] mental activity ... has been transcended." – p. 224
Travis (2014)	"Meditation practices in the *automatic self-transcending* category transcend cognitive and affective processes to reveal ... pure consciousness." – p. 1<br>"... [M]ental activity decreases in intensity and frequency, and ultimately ceases." – p. 3<br>"On the one hand you can say there is no content in pure consciousness. On the other, you could say the content is wakefulness itself or consciousness itself." – p. 3
Faber et al. (2017)	"During the process of transcending, the mind sometimes becomes engaged in an undirected stream of thoughts ('undirected mentation'). When this happens and is noticed by the practitioner, they learn how to effortlessly return to the mantra. This fluctuation between transcending and undirected mentation is the natural flow of a TM practice. Contrary to focused attention meditation practices, undirected mentation is not considered to be off-task but an integral part of the meditation process." – p. 307
Travis (2018)	"[Pure consciousness] has been defined as the state of least activity of the mind – free from thinking or perception ..." – p. 37

2.7.15 Limited or no abstract content

See also 2.2 ("Shear's logic-based argument that pure consciousness has a unique experiential quality").

Shear (1990b)	"... [I]f [the Maharishi's] account ... is correct, [the experience] appears to be the most abstract experience possible, for everything has been extracted from consciousness that can possibly be extracted, everything, that is, except consciousness itself." – p. 102<br>"... [P]ure consciousness ... appears to be the logical ultimate of abstraction, the simplest and most abstract experience conceivable ..." – p. 220
Shear (1990a)	Referring to "introvertive mystical experience", which Shear presents as equivalent to pure consciousness: "... [I]ts defining characteristic is that ... it is completely devoid of all empirical qualities and content – including even abstract contents such as blissfulness, 'the divine', etc." – p. 396 "... [T]he experience traditionally is often reported as experienced in close association with (e.g., immediately before and/or after) other paradigmatic introvertive mystical experiences (unboundedness, bliss, sense of the divine, etc.), and is often held to be the ground of full development of these other experiences." – p. 398
Shear (1995/1997b)	"This experience, in order to be remembered at all, would [seem] to have to have *some* kind of quale associated with it, even if it is nothing more than some sense of an existing nothingness – a sort of 'void' quale. But logically it would have to be *the simplest possible* quale – one tantamount to a phenomenal nothing." – p. 373
Shear (1998/1999)	Shear p. 416 addresses what he calls the "*phenomenological critique*", which "[asks] if the experience can really be *qualityless*". "... [I]t is not implausible to suspect that putative examples of pure consciousness experiences might have *some* content, though too abstract to be noticed and identified by the ordinary observer. That is, there might be some content in the experience that would be apparent to one who had undergone sufficient phenomenological training to uncover typically unnoticed subtleties of one's experience. Here one can readily allow this logical possibility. Nevertheless, there would still be a *family* of closely related experiences well-defined here ..." – p. 416
Shear and Jevning (1999a)	The authors note that when a person honestly reports a contentless experience, they are reporting having had an experience that "*seemed to them*" to be contentless (p. 200). "... [T]hey might have been having experiences which only seemed contentless to them, because of the absence of any *noticed* colours, sounds, feelings, thoughts, spatio-temporal extension, etc., despite the fact that there actually *was* some content that they simply hadn't noticed ... [C]ommonsensically, it does not seem out of the question that one could have an experience with some unobtrusive kind of content that one had not noticed, but which, once one's attention is drawn to it, one could look back at one's memory and conclude, 'Oh, yes. Now I see – *that* was there, after all'. Indeed, it is possible that phenomenological training could be just the kind of thing that could enable someone to notice such previously unnoticed, extremely unobtrusive content." – p. 200<br>"[H]owever ... the experiences ... would still be identifiably 'the same' by virtue of their complete absence of any ordinarily observable content: colours, thoughts, extension, etc. – even if there *were* some differences potentially articulatable in terms of as yet unnoticed, abstract content. That is, we would still have a readily identifiable class of highly unusual experiences that were 'the same' insofar as our ordinary criteria (colours, extension, etc.) for distinguishing the contents of experiences are concerned." – p. 200

Shear (2002)	Referring to pure consciousness and pure positive affect, which are said to be contentless: "It is easy to suppose ... that someone could have an experience the content of which was so faint and abstract that the experience would *seem* absolutely contentless, even though it was not. He or she could then be confident that the experience was empirically contentless – and then have another, more abstract experience that made it clear that the first one *did*, in fact, have some empirical content (content that can be either present or absent in any given experience), namely the content now recognized to be absent in the second. This then also raises the possibility of someone's having two experiences that are so abstract that he could be tempted to call both 'contentless', yet that afterward both seem somehow different (presumably because of some faint content in at least one of them), despite the fact that one could not locate definitely any differentiating content in either of them. This, of course, might lead one to conclude (mistakenly) that one could have two phenomenologically different experiences of purely contentless awareness." – p. 366 "The case of the sameness or difference of different people's putative experiences of pure consciousness experiences is of course even more problematic, for there is no possibility here of comparing them by inspection of the relevant memories." – p. 366 "... [M]y own view, despite the above difficulties, is that 'pure consciousness' is clearly phenomenologically unique ... The deciding factor for me ... is that paradigmatic accounts in culture after culture assert as emphatically as possible not only that there is no content discernible in the experiences, but that there is not even any phenomenological 'space' (subjective temporal and/or spatiotemporal manifold) for such experiences to be *in*. Thus, for these experiences having no *place* for any phenomenological qualities to be located in, the very notion of their having any content at all, anything that can qualitatively differentiate one putative instance from another, appears flatly unintelligible. This, at any rate, appears to be the natural response of many (including myself) who think they have had the experience in question." – p. 367
Shear (2004)	"... [Pure consciousness] is ... utterly abstract. So abstract, in fact, that [its] phenomenological nature is *inconceivable*. For no images or empirical contents whatsoever – not even that of a spatio-temporal manifold for content to appear in – seem relevant to it at all." – p. 87
Shear (2007)	"The experience itself is extraordinarily abstract. Indeed it is the logical ultimate of abstraction, since by all accounts it is what remains after everything that can possibly be removed from experience has been removed, while one nevertheless remains awake." – p. 700<br>"... extreme abstractness ..." – p. 700<br>"It should be noted ... that even if the natural response to this experience is to describe it as 'contentless', it is still appropriate to raise the question of whether it is in fact actually *completely* contentless. For while experiences associated with the [expected] physiological state might be so subtle and abstract that they naturally *seem* to be completely contentless, they might nevertheless actually have *some*, albeit very abstract, content. Indeed, one might, for example, have an experience that one at first took to be contentless, only to conclude after having another even more abstract one later that it did in fact have some content now recognizable by the fact that it is absent from the newer experience." – pp. 702-703 "This is not an entirely fanciful possibility. Texts of Eastern traditions list various extremely abstract experiences, and major traditions have debated related questions for centuries. Some traditions (e.g., Theravada) argue (against Yoga, Vedanta, and Zen) that all states properly called 'conscious' have to have *some* content, even if nothing but 'voidness' itself. Others (e.g., Tibetan Buddhism) are sometimes thought to hold that several apparently contentless experiences exist. Others (e.g., Yoga) hold that experiences can differ by having different physiological substrates, even when, as completely objectless, they are indistinguishable phenomenologically." – p. 703 "These distinctions, however, have little or no bearing on the topic of meditation as a tool for empirical investigation of consciousness. Thus, for simplicity, the phrase 'pure consciousness' will continue to be used here to refer to the experience of empirically contentless awareness, without concern, in the absence of relevant research, for whether the experience is absolutely contentless or only seems to be so, or whether the phrase as used here ultimately turns out to refer to a single experience or a family of closely related ones." – p. 703

2.7.16 No thoughts

Pearson (2013)	"... a state of perfect stillness, beyond all perceptions, thoughts, and feelings ..." – p. 25<br>"It lies beyond the boundaries ... of perception and thought ..." – p. 25<br>Quoting a meditator describing their first session: "... I experienced a silent, inner state of no thoughts ..." – p. 29<br>"... a state of deep inner silence and peace, beyond thought ..." – p. 29<br>"... beyond perceptions, thoughts, and feelings." – p. 41<br>Pearson pp. 44, 45, 46, 47, 186, 265 also refers to the absence of thoughts.

Quoting Maharishi Mahesh Yogi: "... no object of thought or perception ..." – p. 45
"Transcending thinking altogether ..." – p. 45
Quoting a meditator: " ... no longer any thoughts or feelings ..." – p. 52
Quoting a meditator: "... [A]ll thought activity ceases ... In this state I am not aware of any thought ..." – p. 52
"... [O]ne ... comes to rest beyond perceptions, thoughts, and feeling in ... pure consciousness." – p. 395

Roth (2018)
"... state of inner stillness ... that actually *transcends*, or goes beyond, all thoughts and feelings." – p. 17
Quoting the singer Katy Perry: "... I go to this incredible place where I'm not even thinking anything." – p. 164

Shear (1990b)
Quoting Maharishi Mahesh Yogi: "... beyond all thinking and beyond feeling." – p. 45
"... devoid of all sensations and thought ..." – p. 115

Shear (2006b)
"... enable the mind to leave behind all of its ordinary activities of thinking and perceiving ..." – p. xviii
"... no perceptions, feelings, thoughts, or anything else ..." – p. xviii

Shear (2006c)
"... no images, sensations, thoughts, feelings ..." – p. 33

Rosenthal (2016/2017)
"What, then, are the usual elements of transcendence? That was the question I asked a group of students, at Loyola University's Stritch School of Medicine in Chicago, who had learned to meditate. They replied with gusto: stillness, quiet, no boundaries, no thoughts, and bliss. In short, they captured the essential spirit of the state." – p. 31
"Thoughts may still come and go but sooner or later ... silence ... all thoughts have gone." – p. 35
"One's sense of self dissolves into a pleasant emptiness, which eventually fills up again with vibrant thoughts, feelings, and all the bric-a-brac of mental life ..." – p. 35 "Just as our thoughts come and go, so (at times) do other boundaries, such as our sense of time and space ..." – p. 35
Quoting a patient, Moira: "When I have fully transcended, I don't remember too much. Sometimes ideas for my current paintings drift in and out, but mostly I experience a wonderful, restful feeling." – p. 38
Rosenthal p. 46 refers to "the mixed experience so common during TM, in which active thoughts and quiet transcendence may occur at various times – or even simultaneously – during a single session".
Quoting Elaine, a meditator: "... I seem to melt into [the silence], using my mantra to gently come back when I start having thoughts – which was fairly often at first." – p. 217

Rosenthal (2011/2012)
"At certain points during any session, I may experience transcendence ... I am not thinking of anything in particular. Rather, transcendence is an experience of unfocused bliss, which may last for seconds or minutes. It comes and goes mysteriously and cannot be forced. It is a gift of the meditation, one that comes on its own through effortless, innocent practice." – pp. 18-19
"It is the profoundly relaxed alertness free of specific thoughts, along with the loss of personal boundaries of time or space, that are the essential elements of transcendence." – pp. 42-43
Referring to Martin Scorsese: "When Marty starts to meditate, there are 'a thousand thoughts, which eventually settle down into a calm, floating space.'" – p. 227

Travis and Pearson (2000)
Quoting a participant: "... [N]o thoughts are present." – p. 81

Arenander and Travis (2004)
"... without the cognitive activity of thoughts, perceptions, and feelings." – p. 114

Travis (2009)
"... [B]odily sensations, sensory perceptions, thoughts, and feelings are absent." – p. 28

Travis (2014)
"In pure consciousness, there can be no shadow of thought ..." – p. 2

2.7.17 No feelings
See also 2.7.26 ("Bliss, joy, happiness")

Pearson (2013)
"... a state of perfect stillness, beyond all perceptions, thoughts, and feelings ..." – p. 25
"... beyond perceptions, thoughts, and feelings." – p. 41
Pearson pp. 44, 46, 47 also refers to the absence of feelings.
Quoting a meditator: " ... no longer any thoughts or feelings ..." – p. 52

	"... [O]ne ... comes to rest beyond perceptions, thoughts, and feeling in ... pure consciousness." – p. 395
Roth (2018)	"... state of inner stillness ... that actually *transcends*, or goes beyond, all thoughts and feelings." – p. 17
Shear (1990b)	Quoting Maharishi Mahesh Yogi: "... beyond all thinking and beyond feeling." – p. 45
Shear (1994)	Referring to "introvertive mystical experience", which Shear presents as equivalent to pure consciousness: "... completely devoid of all determinate phenomenological contents (sense perceptions, images, thoughts, emotions, sense of individual identity, etc.) whatsoever." – p. 320
Shear (2006b)	"... no perceptions, feelings, thoughts, or anything else ..." – p. xviii
Shear (2006c)	"... no images, sensations, thoughts, feelings ..." – p. 33
Shear (2011a)	"... no feelings or emotions ..." – p. 140
Arenander and Travis (2004)	"... without the cognitive activity of thoughts, perceptions, and feelings." – p. 114
Travis (2009)	"... [B]odily sensations, sensory perceptions, thoughts, and feelings are absent." – p. 28
Travis (2014)	"Meditation practices in the *automatic self-transcending* category transcend cognitive and affective processes to reveal ... pure consciousness." – p. 1

2.7.18 No sense-perceptions

Pearson, Shear and Travis express the clear view that pure consciousness does not involve sense-perceptions, as shown by the passages extracted in this "No sense-perceptions" section below. Roth says little on this point, probably because he is more focused on any movement in the direction of the goal-state/s than he is on the goal-state/s alone (see 2.1, "Terminology ..."). Contrary to Pearson, Shear and Travis, Rosenthal presents sensory experiences as a potential feature of the goal-state/s. He regards "spectacular" sensory experiences as reflecting very deep experiences of transcendence (see 2.7.48, "Subjective experience varies between meditators and across/within sessions"), but, as the following passages show, he also considers that transcending and transcendence can involve more ordinary sensory experiences: "Even though the eyes are closed, perceptions of light may ... arise during a session. A colleague of mine, while meditating, has several times seen a light hovering in the center of her visual field, like a pearl glowing on the horizon ..." (Rosenthal, 2011/2012, p. 41); quoting Tony, a meditator, "I have a large number of transcending experiences. The one that stands out the most is after the first month I had no mantra and thoughts – just blank emptiness that had a warm comforting feeling. However, I was fully aware of the outside surroundings. I've also seen colours during transcending." (2011/2012, p. 196); and quoting a respondent to the Consciousness Integration questionnaire, describing their experience of transcendence, "I am aware only of whiteness surrounding me" (2016/2017, p. 38). If the understanding of Pearson, Shear and Travis is adopted, it seems that these sensory experiences should be regarded as interim, not goal, states. That conclusion fits better with other of Rosenthal's descriptions, for example: "Sometimes, as I hear my mantra, I have an image of myself standing on a vast and empty beach. I see the sands stretching into the distance, the sea and the sky. Then, gradually, the sea falls away, the beach disappears beneath my feet, the sky becomes abstract, and I enter a state of pure consciousness ..." (Rosenthal, 2011/2012, p. 42).

Pearson (2013)	"... a state of perfect stillness, beyond all perceptions, thoughts, and feelings ..." – p. 25<br>"It lies beyond the boundaries ... of perception and thought ..." – p. 25<br>Quoting a meditator describing their first session: "... I experienced a silent, inner state of no thoughts, just pure awareness and nothing else; then again I became aware of my surroundings." – p. 29<br>"... beyond perceptions, thoughts, and feelings." – p. 41<br>Pearson pp. 44, 46, 47, 265 also refers to the absence of perceptions.<br>Quoting Maharishi Mahesh Yogi: "... no object of thought or perception ..." – p. 45<br>Quoting a meditator: "In this state, I am not aware of any thought or any thing ..." – p. 52<br>"In [pure consciousness] ... the senses ... are transcended." – p. 323<br>"... [O]ne ... comes to rest beyond perceptions, thoughts, and feeling in ... pure consciousness." – p. 395
Shear (1990b)	Quoting Maharishi Mahesh Yogi: "... [it] lies beyond all seeing, hearing, touching, smelling and tasting ..." – p. 45<br>"... without any object of perception." – p. 100<br>"... devoid of all sensations and thought ..." – p. 115

Shear and Jevning (1999a)	"... [W]hen the mind has become completely settled ... one steps outside all activity of perception ..." – pp. 194-195
Shear (2006b)	"... enable the mind to leave behind all of its ordinary activities of thinking and perceiving ..." – p. xviii<br>"... no perceptions, feelings, thoughts, or anything else ..." – p. xviii
Shear (2006c)	"... no images, sensations, thoughts, feelings ..." – p. 33
Rosenthal (2016/2017)	Quoting Ray Dalio, founder and president of the hedge fund Bridgewater Associates: "You go into a different state – neither conscious nor unconscious. When you're meditating, you're just not aware. Everything disappears, in a sense. But unlike when you're sleeping, if a pin drops all of a sudden, it can reverberate through you; it's shocking." – pp. 186-187<br>Quoting Elaine, a meditator: "While I'm aware of what is going on around me, I feel calm and detached ... There is quite often time that I 'lose', becoming aware of returning to myself ... Not wanting the experience to end, I'll sometimes go back in for a few minutes before opening my eyes." – p. 217
Arenander and Travis (2004)	"... without the cognitive activity of thoughts, perceptions, and feelings." – p. 114
Travis (2009)	"... [B]odily sensations, sensory perceptions, thoughts, and feelings are absent." – p. 28

2.7.19 No body-perceptions

The passages from the Pearson, Shear and Travis texts extracted in this "No body-perceptions" section below indicate that in pure consciousness the meditator is not aware of their body. Like for the "No-sense perceptions" feature above, Roth says little on this point. The passages from Rosenthal extracted in this section below suggest that the meditator may have some body-perceptions in the TM goal-state/s. However, if the understanding of Pearson, Shear and Travis is correct, it seems that the experiences described by Rosenthal should be considered interim, rather than goal, states.

Pearson (2013)	Quoting a meditator: "... no longer ... even a body ..." – p. 52
Shear (1990b)	Quoting a meditator describing what Shear refers to as an "advanced experience" (p. 231): "Sometimes the sense of infinity is so strong that I lose the sensation of body or matter – just infinite, unbounded awareness, an eternal, never-changing continuum of consciousness." – p. 230
Shear (2006b)	"... [T]he experience contains no trace of body, personality, or anything else we ordinarily identify ourselves *as* ..." – p. xviii
Shear (2014a)	"Any bodily awareness would, of course, be incompatible with [pure consciousness]." – p. 68
Shear (2014b)	"[The TM experience is said to be] accompanied spontaneously by suspension of perceptible respiration." – p. 211
Rosenthal (2016/2017)	Quoting a participant describing their experience of transcendence: "I can hear my heart beat in my ears. I am aware only of whiteness surrounding me." – p. 38
Rosenthal (2011/2012)	"Transcendence, however, is not all in the mind; many descriptions hint at concurrent bodily experience ... In the words of journalist Tim Page, 'I imagine I'm one of the few people who will say this – the sort of all-body tingling that you get in a particular deep meditation reminds me a little bit of the after-glow after an orgasm. It's got that same kind of satisfaction and the same sort of tapping into some kind of deep peace that you sometimes have after that." – p. 39<br>Quoting businessman George Crowley: "One of the ways I know I'm transcending is I begin to feel all these releases throughout my body. They're like little shocks or tingling sensations in my head ... My stomach starts to gurgle. My legs feel lighter. And there have been a few times when I'll just burst out laughing because it's such an incredibly pleasurable experience by the time I get there." – p. 219
Travis and Pearson (2000)	When asked to describe their deepest experiences in TM, 68% of participants referred to them as involving an "absence of space, time, or body-sense" (p. 81).
Travis (2009)	"... [B]odily sensations, sensory perceptions, thoughts, and feelings are absent." – p. 28

Travis and Parim (2017)	"... without the sense of body or environment." – p. 87

2.7.20 No mental images

Shear (1990b)	"... independent of all self-imagery and other empirical content." – p. 172
Shear (1990a)	Referring to "introvertive mystical experience", which Shear presents as equivalent to pure consciousness: "... [T]he reports in diverse cultures throughout the world agree that the experiencers' beliefs, symbols, images, etc. ... are not found in the experience at all." – p. 394
Shear (1994)	Referring to "introvertive mystical experience", which Shear presents as equivalent to pure consciousness: "... completely devoid of all determinate phenomenological contents (sense perceptions, images, thoughts, emotions, sense of individual identity, etc.) whatsoever." – p. 320<br>Shear p. 322 makes a similar comment.
Shear (2006c)	"... no images, sensations, thoughts, feelings ..." – p. 33
Heaton and Travis (2014)	"... unmixed with images, thoughts, feelings, or any other objects of perception ..." – p. 18

2.7.21 No memories

Pearson (2013)	"In [pure consciousness], there is no 'content' ... One transcends the relative contents of consciousness – concepts, values, language, thought, memory, emotion – to experience consciousness in its pure form ..." – p. 456

2.7.22 Cycling
See also 2.7.23 ("Increasing length").

Rosenthal (2016/2017)	"Thoughts may still come and go but sooner or later ... silence ... all thoughts have gone." – p. 35<br>"One's sense of self dissolves into a pleasant emptiness, which eventually fills up again with vibrant thoughts, feelings, and all the bric-a-brac of mental life ..." – p. 35 "Just as our thoughts come and go, so (at times) do other boundaries, such as our sense of time and space ..." – p. 35<br>Quoting a patient, Moira: "When I have fully transcended, I don't remember too much. Sometimes ideas for my current paintings drift in and out, but mostly I experience a wonderful, restful feeling." – p. 38<br>Rosenthal p. 46 refers to "the mixed experience so common during TM, in which active thoughts and quiet transcendence may occur at various times – or even simultaneously – during a single session".<br>Quoting Elaine, a meditator: "... I seem to melt into [the silence], using my mantra to gently come back when I start having thoughts – which was fairly often at first." (p. 217); "While I'm aware of what is going on around me, I feel calm and detached ... There is quite often time that I 'lose', becoming aware of returning to myself ... Not wanting the experience to end, I'll sometimes go back in for a few minutes before opening my eyes." (p. 217).
Rosenthal (2011/2012)	"At certain points during any session, I may experience transcendence ... I am not thinking of anything in particular. Rather, transcendence is an experience of unfocused bliss, which may last for seconds or minutes. It comes and goes mysteriously and cannot be forced. It is a gift of the meditation, one that comes on its own through effortless, innocent practice." – pp. 18-19
Travis (2011)	One "[cycles] through pure consciousness many times in each session ..." (p. 229).
Faber et al. (2017)	"During the process of transcending, the mind sometimes becomes engaged in an undirected stream of thoughts ('undirected mentation'). When this happens and is noticed by the practitioner, they learn how to effortlessly return to the mantra. This fluctuation between transcending and undirected mentation is the natural flow of a TM practice. Contrary to focused attention meditation practices, undirected mentation is not considered to be off-task but an integral part of the meditation process." – p. 307 The authors p. 313 refer to the "continuous cycling between these two phases ... during the practice of TM".
Travis and Parim (2017)	"... TM practice is the process of transcending, coming out onto thought, and transcending again using the 'natural tendency of the mind' ..." – p. 87

2.7.23 Increasing length
See also 2.7.22 ("Cycling").

Pearson (2013)	"When one first learns to meditate, one may experience [pure consciousness] only for brief moments, at the deepest points of meditation. The experience is so natural that one may be hardly aware of it. In time, with regular practice, one maintains the experience for longer periods ..." – p. 51<br>"When one first learns [TM], one typically experiences [pure consciousness] only for brief moments, at the deepest points of meditation. But in time, with repeated, twice-daily experience, one's mind becomes so familiar with [it] that one begins to maintain the experience of it for longer periods." – p. 173<br>"... [W]ith repeated transcending, stress dissolves and the nervous system becomes more flexible and refined. This allows deeper and clearer experiences of [pure consciousness], more frequent and longer-lasting. Eventually the nervous system becomes virtually free of stress." – p. 175
Rosenthal (2016/2017)	"Thoughts may still come and go but sooner or later ... silence ... all thoughts have gone." – p. 35<br>Quoting Elaine, a meditator: "... I seem to melt into [the silence], using my mantra to gently come back when I start having thoughts – which was fairly often at first." – p. 217

2.7.24 Calm, ease, peacefulness, absence of disturbance

Pearson (2013)	"... a state of deep inner silence and peace ..." – p. 29<br>"Our minds are continuously stirred by perceptions, thoughts, and feelings, much as the ocean is swept into waves by winds and currents. But like an ocean the mind can settle down. It can become calm, quiet, silent, while remaining awake. This is [pure consciousness]." – p. 44<br>"Your mind is awake but silent and serene." – p. 44<br>Quoting a meditator: "... I slip into an unbounded ocean of awareness which is pure, quiet, unexcited ..." – p. 52<br>Quoting a meditator: ".... [I experience] bliss (sometimes quiet, sometimes bubbly) and peace." – p. 52<br>"... an inner sanctuary of infinite silence, peace, and joy. This inner center cannot be shaken, cannot be touched – it is pure silent, transcendental bliss consciousness ..." – p. 175<br>Quoting a meditator discussing pure consciousness as encountered in daily life: "I am connected to the universal value of life. Things don't disturb that level of life." – p. 185
Roth (2018)	"... I use an analogy ... You are in a little boat in the middle of the Atlantic Ocean ... [I]f you could look at a cross section ... you would see that only the surface is in turmoil. The Atlantic is several miles deep, and at its depth, the ocean is very, very calm. Down there is an unbounded expanse of peace and tranquility, entirely undisturbed by the turbulence above." – p. 11<br>"... [D]eep within is a level that is calm yet alert; silent yet wide awake." – p. 17<br>"... inner calm ..." – p. 34<br>"... quiet, peaceful, transcendent state of awareness." – p. 37<br>"What does it feel like to meditate? ... Mentally, it's calming yet awakening ..." – p. 58<br>Quoting Donna Rockwell, a clinical psychologist and meditator: "From the very first time it felt like my brain was settling into a nice warm bath. My brain calms down ..." – p. 61<br>Quoting Shane Anselmi, a meditator: "[The practice] is something I look forward to because it brings a relaxed peace; a sense of joy, calm and clarity ..." – pp. 66-67<br>Quoting Deborah-lee Furness: "When I meditate, I'm joining my true self, which is this calm, peaceful, blissful, limitless self." – p. 78<br>Head of the TM organization, Dr Tony Nader, describing his first TM session/s: "Right away, I experienced ... transcendence – a deep inner calm, a deep inner bliss and a great expansion of awareness." – p. 193
Shear (2006c)	"... undisturbed by thoughts, perceptions, and other mental activity ..." – p. 24
Rosenthal (2016/2017)	"... stillness, expansiveness, boundlessness, and peace ..." – p. 5<br>"... inner expansive calm ..." – p. 5<br>"As ... thoughts mingle with the blissful peace of transcendence, they become less bothersome, disturbing, or problematic." – p. 26<br>"... peace and quiet." – p. 31
Rosenthal (2011/2012)	"... a technique that allows me to be by myself, very calm and happy ..." – p. 26<br>"The emotions most often associated with transcendence are serenity and bliss." – p. 38<br>Quoting a meditator: "During transcendence I experience a feeling of total calmness and peace ..." – p. 39

	Quoting meditator Tim Page: "... the feeling ... of sliding into this very, very natural peace ..." – p. 93<br>Quoting a meditator: "The evening TM helps set my mind at ease ..." – p. 136<br>Quoting a meditator: "TM is a tranquil oasis against the self-critical and stormy thoughts I used to have upon awakening. It gives me a sense of peace ..." – p. 136<br>Quoting Lyn, a meditator: "One of the gifts of TM is the peace and calm it brings." – p. 209<br>Quoting Paul McCartney: "I think [TM] offers a moment in your day to be at peace with yourself ..." – p. 226<br>Referring to Martin Scorsese: "When Marty starts to meditate, there are 'a thousand thoughts, which eventually settle down into a calm, floating space'." – p. 227
Travis and Pearson (2000)	When asked to describe their deepest experiences in TM, 32% of participants referred to them as peaceful (p. 81).
Travis et al. (2005)	Quoting a meditator: "... an inner peace that is very, very nourishing." – p. 128
Travis and Parim (2017)	"In this research, transcendence is described as a perfectly peaceful state in which the mind is very awake, but still ..." – p. 87

2.7.25 Beyond peacefulness

See also 2.7.2 ("Cannot say much about it") and 2.7.15 ("Limited or no abstract content")

Pearson (2013)	Pearson p. 53 quotes a meditator who describes "going deeper and deeper" into an ocean of consciousness, remarking "It is beyond peaceful, beyond serene.". It is not clear from the passage whether the meditator is meaning that the experience is extremely peaceful/serene, or entirely beyond those experiential qualities. The meditator goes on to say: "Then something quite extraordinary happens. The ocean disappears. I mean it is just gone, and I am in an indescribable place." – p. 53

2.7.26 Bliss, joy, happiness

See also 1.8.2 ("Pure individuality and pure bliss/joy/happiness").

Pearson (2013)	"No experience fills the mind with greater happiness." – p. 45<br>"In [pure consciousness], we experience pure, unbounded bliss consciousness." – p. 46<br>"Maharishi has called [pure consciousness] an ocean of *pure knowledge, power, and bliss*." – p. 48<br>Quoting a meditator describing their first session: "The movement of my awareness ... to the field of silence within myself ... was like diving into a pond of pure joy. I felt so much happiness; the laughter just bubbled up automatically." – p. 51<br>Quoting a meditator: "[I experience] bliss (sometimes quiet, sometimes bubbly) and peace." – p. 52<br>"... an inner sanctuary of silence, peace, and joy ... [I]t is pure, silent, transcendental bliss consciousness ..." – p. 175<br>Discussing pure consciousness as encountered in daily life: "The mind is grounded in its essential nature, the Self, an immovable ocean of bliss." – p. 180<br>"[Pure consciousness] ... gives the experience of pure bliss ..." – p. 396
Roth (2018)	"... a field of satisfaction and happiness ..." – p. 34<br>Quoting Shane Anselmi, a meditator: "[The practice] is something I look forward to because it brings a relaxed peace; a sense of joy, calm and clarity ..." – pp. 66-67<br>Quoting Deborah-lee Furness: "When I meditate, I'm joining my true self, which is this calm, peaceful, blissful, limitless self." – p. 78<br>Head of the TM organization, Dr Tony Nader, describing his first TM session/s: "Right away, I experienced ... transcendence – a deep inner calm, a deep inner bliss and a great expansion of awareness." – p. 193
Shear (1990b)	Shear is not entirely clear about whether pure consciousness involves bliss. His comments at pp. 44-45 and 163-164 could easily be read as suggesting that it is only the interim, pure individuality stage that is "bliss-permeated", not pure consciousness (the goal-state) (see 1.8.2, "Pure individuality and pure bliss/joy/happiness"). However, at p. 172 he states: "[T]he experience of pure consciousness turns out to be associated with a greater degree of happiness, joy, and comfort than is ever likely to be associated with any external experience or self-imagery."<br>Quoting a meditator: "... [M]editations were wonderfully soft, delicate, blissful, and full." – p. 230

Shear (1990a)	Referring to "introvertive mystical experience", which Shear presents as equivalent to pure consciousness: "... [I]ts defining characteristic is that ... it is completely devoid of all empirical qualities and content – including even abstract contents such as blissfulness, 'the divine', etc." – p. 396 "[T]he experience traditionally is often reported as experienced in close association with (e.g. immediately before and/or after) other paradigmatic introvertive mystical experiences (unboundedness, bliss, sense of the divine, etc.), and is often held to be the ground of full development of these other experiences." – p. 398
Shear (2002)	Referring to pure consciousness and pure positive affect: "... [T]he two experiences are so closely associated that they are sometimes conflated ... despite the fact that they are clearly two phenomenologically different experiences, one having no phenomenological content at all, and the other having the abstract content of pure happiness, or bliss." – p. 378
Shear (2006b)	"Tradition after tradition reports that the experience is intimately associated with profound experiences of bliss, happiness, and freedom." – p. xix
Shear (2006c)	"... [T]he deeper levels of inner awareness feel very good, and the deeper we go within, the better we feel ... [T]hroughout history, and across different civilizations, those who have experienced the full range of mind agree that the deepest level of inner awareness is what can be described as *pure happiness* or fulfillment." – p. 26<br>"... 'the bliss' [quoting Maharishi Mahesh Yogi] of pure consciousness ..." – p. 26<br>"... [H]appiness increases as the mind settles, and this draws the mind quite spontaneously to the state of pure consciousness ..." – p. 34<br>"[J]ust before reaching its completely settled state of pure consciousness, the mind may pause at a level that can be called *pure* happiness, or bliss." – p. 35<br>Shear p. 44 refers to the "deepest level of pure consciousness" as being "intimately associated with what can be called pure happiness, bliss, or fulfillment itself".
Shear (2007)	Shear p. 699 states that in Vedanta "pure positive affect (happiness, bliss, etc.)" is considered the second deepest level of consciousness, and pure consciousness the deepest.
Shear (2011a)	Quoting a meditator having what Shear describes as a pure consciousness experience: "My meditations are characterized by the experience of 'no experience' ... I just remain in the Absolute for the entire sitting and nothing else seems to happen, other than the feeling of bliss permeating me completely." – pp. 142-143
Shear (2014a)	"... [Pure consciousness] contains no phenomenal content at all. Even bare subject-object orientation and abstract, objectless bliss are absent." – p. 60
Rosenthal (2016/2017)	"Your mind feels perfectly settled ... suffused with joy ..." – p. 31<br>"What, then, are the usual elements of transcendence? That was the question I asked a group of students, at Loyola University's Stritch School of Medicine in Chicago, who had learned to meditate. They replied with gusto: stillness, quiet, no boundaries, no thoughts, and bliss. In short, they captured the essential spirit of the state." – p. 31<br>"... [T]he sense of oneness is blissful and relaxing." – p. 35<br>"One property of transcendence that the Loyola students nailed is the feeling of bliss ... Beyond the components of joy and happiness, for which there are many other words, 'bliss' also conveys a sense of serenity and peacefulness. I don't know any other single word that captures all these elements, which together typify the state of transcendence." – p. 36<br>Quoting a patient, Moira: "... [M]ostly I experience a wonderful, restful feeling." – p. 38<br>"My own transcendent experiences and those of most of my patients are pleasant but unsensational." – p. 39<br>Quoting Ray Dalio, founder and president of the hedge fund Bridgewater Associates: "It is a combination of relaxation and a very blissful experience. That sounds more like an orgasm than it really is, but it is blissful in the sense that I just feel really good and relaxed and in good shape." – p. 186<br>Rosenthal p. 234 refers to the non-dual experience in TM as "highly pleasurable".
Rosenthal (2011/2012)	"[Transcendence] is a blissful state that encompasses elements of serenity, peace, and acceptance, but also exhilaration and a sense of new possibilities, both for now and for the future." – p. 8<br>"... the ... wonderful state of consciousness called *transcendence* ..." – p. 11<br>"... a technique that allows me to be by myself, very calm and happy ..." – p. 26<br>"The emotions most often associated with transcendence are serenity and bliss. The word 'bliss', as opposed to 'joy' or 'happiness', is seldom heard in daily life, but it really does capture the calm pleasure

	experienced during transcendence, a pleasure directed at nothing in particular and at everything all at the same time." – p. 38<br>Quoting filmmaker David Lynch: "When I had my first meditation, this inner bliss revealed itself so powerfully – thick happiness came rushing in." – p. 94<br>Referring to prisoners meditating: "Each day it gives them a positive experience – a way to find something good inside themselves, something that's worth accessing." – p. 204

2.7.27 No sense of anxiety
See also 2.7.24 ("Calm, ease, peacefulness, absence of disturbance")

Pearson (2013)	"[In pure consciousness] [t]he body is deeply restful and is dissolving fatigue and stress." – p. 50<br>Quoting a meditator: " ... [T]he concerns and worries that may have been gripping my mind fall away ..." – p. 52<br>"... [W]ith repeated transcending, stress dissolves and the nervous system becomes more flexible and refined. This allows deeper and clearer experiences of [pure consciousness], more frequent and longer-lasting. Eventually the nervous system becomes virtually free of stress." – p. 175<br>"... [T]he body enjoys exceptionally deep rest. Freed of the usual demands upon it, the body uses the opportunity to dissolve accumulated stress and fatigue." – p. 396 "As [TM] enables the mind to transcend, in the same stroke it allows the body to dissolve the stresses that hinder transcending." – p. 396
Roth (2018)	Quoting Shane Anselmi, a meditator: "[The practice] is something I look forward to because it brings a relaxed peace ... And now, any feeling of anxiety seems to disappear." – pp. 66-67<br>Describing his first session of TM: "As [the teacher] led me through the beginning steps ... I felt my mind and body sinking into a state of deep relaxation ... The tension in my neck, shoulders and stomach muscles were the first to go ..." – p. 152
Rosenthal (2011/2012)	"Meditators often say that they feel a gentle release of stress during TM sessions, either physically or psychologically. I often feel that, too." – p. 28<br>Quoting a meditator: "It is as if 'I' exist and yet there is another 'I' which exists and is eternal and totally without anxiety or involvement in the relative world." – p. 39<br>Quoting Mark, a meditator: "It takes all the stress out of my mind." – p. 195
Heaton et al. (2012)	"The state of restfulness gained during the practice of [TM] is said to dissolve the stress in the mind and the body." – p. 70
Heaton and Travis (2014)	"The state of restful alertness gained during the practice is said to dissolve the stress in the mind and the body ..." – p. 20

2.7.28 No longer aware of mantra

Roth (2018)	Quoting George Harrison from the Beatles: "... [T]he mantra becomes subtler and subtler until finally you transcend even the mantra." – p. 40
Shear (1990b)	"The experience of the 'mantra' becomes progressively more abstract and finally disappears entirely ..." – p. 101
Shear (1995/1997b)	"... removing attention from all phenomenal *objects of* consciousness until ultimately even the meditation procedure itself is left behind (or 'transcended') ... leaving *consciousness* alone by itself." – p. 370<br>"... repetition of meaningless *mantras*, ultimately to be forgotten ..." – p. 370
Shear (1998/1999)	TM is "designed to allow one's attention to withdraw from all ordinary contents of awareness, including finally even the procedure itself ..." – p. 413
Shear and Jevning (1999a)	"... [T]here is nothing in principle paradoxical in the notion of a procedure which could first bring all of one's awareness to a single point, and then transcend its own activity – whether through fatigue or relaxation – thus bringing both itself and all other mental activity to a halt." – p. 192<br>According to the account of Maharishi Mahesh Yogi: "[F]inally [the mind] settles entirely, transcending all experience of the object (the *mantra*) that had been engaging it." – p. 193

Varela and Shear (1999b)	"... [T]he aim is to develop the method until it annihilates itself, yielding states of 'pure' (contentless) consciousness." – p. 8
Shear (2006c)	"... [T]he activity of the procedure automatically becomes less and less and ceases entirely, until the procedure itself is transcended ..." – pp. 31-32
Shear (2011b)	"According to Maharishi Mahesh Yogi ... the technique works by 'transcending its own activity'. This means that it is designed so that its activity, once started, automatically tends to diminish and disappear. This allows experience of subtler states of inner awareness including, when the activity of the technique has disappeared entirely ... pure consciousness ... The technique tends to transcend its own activity and disappear spontaneously." – p. 53
Shear (2014b)	Referring to automatic self-transcending practices (which include TM): "Practices of this kind, once started, are reported to automatically 'transcend' their own activity and disappear, to be started up again later if appropriate." – p. 209
Rosenthal (2011/2012)	"... [E]ffortlessly thinking the mantra repetitively takes you beyond the mantra ..." – pp. 14-15<br>"... TM ... is a technique for transcending its own procedures – appreciating the mantra at 'finer' levels in which the mantra becomes increasingly secondary in experience and ultimately disappears ... [T]he object of experience fades away – you use the mantra to lose it." – p. 288
Travis and Shear (2010a)	The authors say that automatic self-transcending practices (which include TM) are "designed to transcend their own activity" (p. 1110). "... [T]he TM technique is a technique for transcending its own procedures – ... the mantra becomes increasingly secondary in experience and ultimately disappears ... In TM, the object of experience fades away – you use the mantra to lose it." – p. 1116

2.7.29 Effortlessness, relinquishment of control, non-doing, no focus/concentration

See 1.6 ("Effortlessness, control and relinquishment, no concentration").

Shear (2006c)	"... [S]o long as one is attending to one's breath (or anything else), one is busy experiencing, and cannot be in the completely simple, contentless state of pure consciousness itself." – p. 36
Shear (2011b)	"In ... TM, the meditator is of course actually doing something. Yet pure consciousness/emptiness is completely devoid of all perceptions, sensations, thought, will, and other identifiable phenomenal content, so there is no possibility of doing anything here at all. Thus, this state can hardly be thought of as involving what one was practicing in ... TM ..." – p. 53
Rosenthal (2011/2012)	"... [Transcendence] is an experience of unfocused bliss ..." – p. 18<br>"During transcendence, there is consciousness *with no specific focus* ... This idea of being aware but not of anything in particular may seem quite weird until you actually have the experience." – p. 38<br>Rosenthal p. 38 describes bliss as "a calm pleasure ... directed at nothing in particular and at everything all at the same time."<br>"... I enter a state of pure consciousness, alert but focused on nothing in particular ..." – p. 42
Arenander and Travis (2004)	"... [P]ractitioners of TM practice report that the absence of any concentration or effort unfolds experiences of [pure consciousness]." – p. 121
Travis and Shear (2010a)	The authors say that automatic self-transcending practices (which include TM) are "marked by the absence of both (a) focus and (b) individual control or effort" (p. 1111).
Travis (2011)	"... a state of consciousness ... [where] cognitive control ... has been transcended." – p. 224
Travis (2014)	"... a state of being rather than thinking or doing, called pure consciousness." – p. 1<br>"In pure consciousness, there can be no shadow of ... individual intention." – p. 2

2.7.30 Attention directed inward

Pearson (2013)	"In this state, attention has settled inward ..." – p. 41<br>"Attention has settled inward." – p. 50

Roth (2018)	"You will learn the subtle mechanics of how to give the attention of your ... mind an inward direction so that automatically you begin to settle down and transcend into stillness." – p. 35
Shear (1990b)	"Maharishi's analysis of the process of meditating effectively makes it clear that, as he understands it, gaining experience of [pure consciousness] requires a 'reversal' of our ordinary direction of attention." – p. 43
Shear and Jevning (1999a)	Referring to traditional accounts that include TM: "What is needed ... is to 'reverse' the direction of one's attention and turn it from its ordinary 'outward' orientation to feelings, thoughts, sensations, and objects 'inward' towards consciousness itself. We normally, of course, tend to think of awareness of such things as feelings and thoughts as 'inward' in contrast to 'outer' awareness of the physical world in general ... But the notion of 'inward' used here is much more radical. For here even awareness of one's own most private, internal thoughts and feelings is still external to one's awareness itself, for they still appear *to* one's awareness, in front of one's 'mind's eye', so to speak, and the 'inward' referred to here is intended to indicate a complete reversal of attention, away from thoughts and feelings as much as from external objects, back into *awareness itself.* This radical redirection of awareness, away from all of its ordinary objects, whether sensations, images, or thoughts, is then said in time to produce experience of both the ground and deep structure of all of our consciousness awareness." – pp. 190-191
Rosenthal (2011/2012)	"... TM ... reliably turns the attention of your mind within." – p. 278<br>"TM involves automatic moving of attention to mental silence." – p. 288
Travis and Shear (2010a)	"... TM practice involves automatic moving of attention to mental silence." – p. 1116

2.7.31 Transcendence of discomfort

Rosenthal (2016/2017)	Quoting Laura, a patient: "TM has the ability to remove pain ... It's some sort of miracle thing. I've ... practiced TM during [painful medical procedures], and not felt the discomfort one would expect." – pp. 154-155<br>Quoting Elaine, a meditator: "I become aware of bodily sensations of pain or discomfort, knowing that they will melt away, and by the end of the meditation they have mostly gone." – p. 217
Rosenthal (2011/2012)	"As part of deep relaxation, concerns or unpleasant memories, which sometimes take the form of physical pains or tension, may come and go of their own accord. I don't argue with them, worry about them, or try to analyze them. I just let them come and go." – p. 28

2.7.32 Naturalness

See also 2.7.36 ("Nature of mind/being, ground state, self/Self").

Pearson (2013)	"... a state of consciousness that is simple and natural ..." – p. 21<br>"... [Pure consciousness] is a natural state." – p. 33<br>"There is nothing more simple or more natural [than pure consciousness]." – p. 44<br>"[Pure consciousness] is the most simple and natural experience a person can have – the experience of awareness in its most quiet state." – p. 49
Roth (2018)	Quoting Lena Dunham, the actor: "... [W]hat [TM] does for me is bring me back to my most natural state ..." – p. 189
Shear (1990b)	Quoting a meditator describing an experience of unboundedness that Shear refers to as advanced: "It is a feeling of great freedom, but also one of naturalness ..." – p. 230
Rosenthal (2016/2017)	Quoting Roth: "I must emphasize that transcending is not a flashy experience. It is quite simple and natural. We are just settling down to our own quiet, inner, unbounded self." – p. 261
Rosenthal (2011/2012)	Quoting meditator Tim Page: "... the feeling ... of sliding into this very, very natural peace ..." – p. 93<br>Quoting Roth: "... completely natural state ..." – p. 278
Travis and Pearson (2000)	"Pure consciousness is a direct experience of the natural structure of human experience during systematic meditation practice." – p. 79

2.7.33 Loss of ego

Pearson (2013)	"It lies ... beyond individuality, time, and space." – p. 25<br>"One transcends individuality ..." – p. 45<br>Quoting a meditator: "There is no longer any sense of 'me' and 'not me' ..." – p. 52<br>Quoting a meditator: "I could say the individual 'I' had become the Cosmic 'I'. But that is not quite it. Rather it's as if there never was anything but the Cosmic I. The individual 'I' never existed to begin with ..." – p. 53<br>Quoting a meditator: "... I began to experience it not just as a state with no thought but rather as having no boundaries; then as unbounded, beyond the limitations of my individuality ..." – p. 183<br>Quoting a meditator discussing pure consciousness as encountered in daily life: "... no ego ..." – p. 186
Shear (1990b)	"... transcendental pure awareness completely devoid of ... sense of individuality ..." – p. 43<br>Quoting Maharishi Mahesh Yogi: "... [T]he intellect, losing its individuality, begins to gain universality, begins to gain the unbounded status of Being." – p. 44<br>"... entirely beyond ... all awareness of individuality ..." – p. 45
Shear (1994)	Referring to "introvertive mystical experience", which Shear presents as equivalent to pure consciousness: "... beyond the phenomenal ego ..." – p. 320
Shear (2006b)	"... [T]he experience contains no trace of body, personality, or anything else we ordinarily identify ourselves *as* ..." – p. xviii
Rosenthal (2016/2017)	"One's sense of self dissolves into a pleasant emptiness ..." – p. 35
Rosenthal (2011/2012)	"During a state of transcendence, people commonly lose their sense of boundaries (where you begin and end). One ten-year-old boy ... told me that once during a session he forgot where his hands were, only to discover later that they were on the floor propping him up." – pp. 37-38<br>Quoting meditator Tim Page: "It allows me a sort of vacation from myself, to lose my egoism ..." – p. 93
Travis (2009)	Travis pp. 28-29 refers to the goal-state/s in TM as spiritual experience/s. "The author defines spiritual experiences as those of wholeness – ... that part of us not tied to ... our ... personality." – p. 28
Travis (2018)	"... [In pure consciousness] the boundaries that define our individuality ... are transcended." – p. 36

2.7.34 Simplicity

Pearson (2013)	"... a state of consciousness that is simple and natural ..." – p. 21<br>"... Maharishi describes [pure consciousness] as *the simplest form of human awareness* ... There is nothing more simple or more natural." – p. 44<br>"[Pure consciousness] is the most simple and natural experience a person can have – the experience of awareness in its most quiet state." – p. 49<br>Quoting a meditator discussing pure consciousness as encountered in daily life: "It was just that simplest state of awareness ..." – p. 186
Shear (1990b)	"... just simply consciously *being*." – p. 103<br>"... the simplest and most abstract experience conceivable ..." – p. 220
Shear (1994)	"... [N]othing could be phenomenologically simpler than an experience with no empirical content at all." – p. 322
Shear (1995/1997b)	Shear p. 370 refers to pure consciousness as "the *simplest state* of awareness".<br>"... consciousness in its simplest state ..." – p. 372<br>"... *the simplest possible* quale ..." – p. 373
Shear and Jevning (1999a)	"... simplest, non-active, completely silent state [of consciousness] ..." – p. 205
Shear (2006c)	" ... simplest, most basic state." – p. 24<br>"... the simplest, least excited state of consciousness ..." – p. 25

	"... simplest, nonactive state." – p. 27
Shear (2011a)	"The experience ... is extraordinarily simple. Indeed, it appears to be the logical ultimate of simplicity." – p. 140
Rosenthal (2016/2017)	Quoting Roth: "I must emphasize that transcending is not a flashy experience. It is quite simple and natural. We are just settling down to our own quiet, inner, unbounded self." – p. 261
Travis (2018)	"... [Pure consciousness] is the simplest form of human awareness in which the experience is awake to his own existence ..." – p. 36

2.7.35 Pure being/Being

Pearson (2013)	Quoting a meditator discussing pure consciousness as encountered in daily life: "I was that pure Being, pure simple awareness. That was my Self. It was my home, my abode." (p. 186); "... that silent state of pure Being, pure self-referral consciousness ..." (p. 186).<br>Pearson p. 265 treats the term "pure Being" as equivalent to pure consciousness.
Roth (2018)	"... the deepest core of your own being." – p. 57
Shear (1990b)	"... gains experience of pure 'Being'." – p. 45<br>"... just simply consciously *being*." – p. 103
Shear (1998/1999)	"... [I]t is often referred to as ... 'pure being' (since nothing positive can be said about it except that it *was*) ..." – p. 413
Shear and Jevning (1999a)	The authors refer to the word "Being" in TM as a "metaphysically tinged [term]" (p. 193).
Shear (2006b)	Referring to the experience of pure consciousness in TM and other meditation traditions: "The experience is often referred to as pure 'being', for it had to have *been* to be experienced and remembered. On the other hand, it is often referred to as 'nonbeing', for there is nothing identifiable that one can say it *contained* or *was*." – p. xviii
Shear (2011a)	"Vedanta ... refers to [the experience] as 'Being', since all that can be said about it when one emerges seems to be that it *was*. Many Buddhists also often refer to it as 'nonbeing', since there seems to be nothing there." – p. 140
Shear (2014a)	"Different traditions interpret the experience differently. Advaita Vedanta refers to it as 'pure Being', since all that one can say directly about the experience is that it *was* ... Buddhist traditions generally refer to it as 'non-being' and/or 'emptiness', emphasizing the absence of every conceivable thing." – p. 60
Rosenthal (2016/2017)	Quoting Dave, a meditator: "It's pure being. It's is-ness, pure am-ness. It is the essential nature of existence, the very principle of awareness." – pp. 238-239
Rosenthal (2011/2012)	Rosenthal pp. 25-26 discusses how a benefit of TM is that it allows the meditator to "just be" (p. 26).
Travis et al. (2005)	Quoting Maharishi Mahesh Yogi: "... the state of Being ..." – p. 127
Travis (2006)	"Being or pure self-awareness is maintained." – p. 24
Travis (2014)	"... a state of being rather than thinking or doing, called pure consciousness." – p. 1

2.7.36 Nature of mind/being, ground state, self/Self
See also 2.7.46 ("Deep").

Pearson (2013)	Pearson pp. 33-37 indicates that, consistent with the Vedic tradition, pure consciousness is "sometimes termed *the ground of being* or *the divine ground*" (p. 33), and that in that tradition it is considered to involve "[realizing] one's true nature, the eternal Self" (p. 33).<br>"... unbounded pure awareness, our innermost Self." – p. 41

	"... the unbounded Self." – p.41<br>"This is our true self, our self in its pure state, uncolored by waves of feeling, thought, and perception. So distinct is this from the self we ordinarily experience that Maharishi distinguishes it as the 'higher Self', with a capital 'S'." – p. 46<br>Quoting a meditator: "I have had the experience of transcending all activity and experiencing awareness as an unbounded unity. There is no longer any sense of 'me' and 'not me', no longer any thought or feelings or even a body – just the Self, and that is all there is, and that is all I am." – p. 52<br>Quoting a meditator: "... I am in an indescribable place. I am alone with my Self ... There is nothing but That – no ocean [of consciousness], no me, no anything, but totality." – p. 53<br>"In [pure consciousness], you experience your true Self, unbounded and eternal." – p. 177<br>Discussing pure consciousness as encountered in daily life: "The mind is grounded in its essential nature, the Self, an immovable ocean of bliss." – p. 180<br>Quoting a meditator discussing pure consciousness as encountered in daily life: "I was that pure Being, pure simple awareness. That was my Self. It was my home, my abode." – p. 186 "It was so natural ... that I realized that 'it' had always been there." – p. 186<br>"[O]ne experiences pure consciousness as one's innermost Self." – p. 323
Roth (2018)	"It is your own quiet inner self ... It's always been there, within you. It just gets lost or overshadowed easily by the constant noise and distractions of the day." – p. 17<br>"... the deepest core of your own being." – p. 57<br>Quoting Deborah-lee Furness: "When I meditate, I'm joining my true self, which is this calm, peaceful, blissful, limitless self." – p. 78<br>"... [M]editating gives direct access to your own innermost, unbounded self." – p. 119
Shear (1990b)	"... leaving the experience of pure consciousness itself ... identifiable as the self." – p. 115<br>"... the ground state ..." – p. 198<br>"... the ground state or basis of mind ..." – p. 199<br>"... the least excited, ground state of consciousness ..." – p. 204
Shear (1996b)	"... ground of mind ..." – p. 371
Shear (1995/1997b)	"[The] deeper structures of consciousness ... emerge from an underlying 'ground' of consciousness which is also experienceable." – p. 370 Shear p. 370 makes clear that he is referring to pure consciousness.
Shear (1998/1999)	"... [TM] associate[s] the experience with what [it] take[s] to be the true, underlying nature of self." – p. 414
Shear and Jevning (1999a)	"[Experiential reports] [place] 'pure consciousness' at the ground of all of our inner awareness ..." – p. 204
Shear (2006b)	"... enable the mind to ... settle so deeply within its own nature that it reaches its own source ..." – p. xviii<br>Referring to the experience of pure consciousness in TM and other meditation traditions: "Since the experience contains no trace of body, personality, or anything else we ordinarily identify ourselves *as*, it is often referred to as 'non-self'. Yet since every experience would seem to require an experiencer, and there are no *two* things present here, the experience is also widely taken to display pure 'Self' (spelled with a capital 'S' to distinguish it from what we ordinarily mean by 'self'.)" (p. xviii); "The experience is also widely taken to have deep metaphysical significance. For it is almost universally thought to display a level of reality deeper than our individual personalities and minds. Precisely what this deeper level is taken to be, however, often differs very greatly from tradition to tradition. For in accord with their respective meta-physical perspectives, different traditions take it to display things as different as the ground of self, the 'nonself' underlying self-awareness, the Self of everything, and God. Thus even where experiences seem quite similar, interpretations can differ greatly." (p. xix).
Shear (2006c)	"... [O]ne's mind is awake in its original nature ..." – p. 24
Shear (2011b)	"... ground of consciousness ..." – p. 51
Shear (2014a)	"... ground of everyone's awareness ..." – p. 60
Rosenthal (2016/2017)	"... [T]he state we access during TM is simply a deep part of ourselves." – p. 31

	Quoting Dave, a meditator: "It's pure being. It's is-ness, pure am-ness. It is the essential nature of existence, the very principle of awareness." – pp. 238-239<br>Quoting Roth: "In terms of [TM], we hypothesize that ... deep within every human being there is a level of the mind that is *already* calm, settled, and silent ..." (p. 259); "It is your own inner self." (p. 259); "We are just settling down to our own quiet, inner, unbounded self." (p. 261)
Rosenthal (2011/2012)	Quoting a meditator: "It is as if 'I' exist and yet there is another 'I' which exists and is eternal and totally without anxiety or involvement in the relative world." – p. 39<br>Quoting Roth: "... [T]here is a level, deep within the mind, that is ever silent, settled, fully expanded, and fully alert ... It exists within every human being." (p. 277); "... [I]t is your own inner Self." (p. 278).
Arenander and Travis (2004)	The authors refer to pure consciousness as "the 'ground' of all experiences of self-awareness" (p. 112) and as "a foundational state of consciousness" (p. 113).<br>"... the state of the self alone ... This level of experience is often written with a capital *S* – Self – to differentiate it from the experience of self-awareness identified with ongoing experiences, thoughts, and actions." – p. 114
Travis et al. (2005)	"The content of Transcendental Consciousness is pure self-awareness – the self experiences the self through the self." – p. 126<br>"[Pure consciousness] appears to be a fundamental state. It cannot be reduced to more fundamental elements, because it is a state devoid of other elements ..." – p. 131
Travis (2006)	"... [T]he self is only aware of itself ..." – p. 23<br>"Being or pure self-awareness is maintained." – p. 24<br>"Pure self-awareness is Self-awareness ..." – p. 26<br>"It is the deepest aspect of our selves – called the Self." – p. 36
Travis (2011)	"Meditation techniques in the *automatic self-transcending* category ... [reveal] a ground state of human consciousness." – p. 224
Travis (2016)	"Transcending leads to the experience of one's innermost self ..." – p. 76
Gobec and Travis (2018)	"The process of transcending during TM practice gives the experience of a deeper, richer, more silent sense of self ..." – p. 69

2.7.37 Wholeness, integration, unity

Pearson (2013)	Quoting Maharishi Mahesh Yogi: "[Pure consciousness] is wholeness, aware of itself ..." – p. 45<br>Quoting a meditator: "I have had the experience of transcending all activity and experiencing awareness as an unbounded unity." – p. 52<br>Quoting a meditator: "The experience is very nourishing ... The transcendent is a completeness." – p. 52<br>Quoting a meditator discussing pure consciousness as encountered in daily life: "... [M]y awareness is this unbounded wholeness of my Self, quietly accompanying the thoughts and feelings in my daily life." – p. 183
Shear (2011a)	"... the unifying ground ..." – p. 148
Rosenthal (2016/2017)	"... [T]he sense of oneness is blissful and relaxing." – p. 35<br>"The concept of nonduality in which the experiencer, the object of the experience, and the experience itself merge into unity ..." – p. 234
Rosenthal (2011/2012)	Quoting Craig Pearson, describing his own experience: "There's definitely a diving deep within, a settling down of mental activity and deep integration." – p. 42
Travis and Pearson (2000)	Quoting a participant: "I feel completely whole ..." – p. 81
Travis et al. (2005)	Quoting a meditator: "I am completely full, vibrant, and alive, but I am completely still. It's absolute silence." – p. 128<br>Quoting a meditator: "... I feel right at home ... Everything seems right." – p. 128

Travis (2009)	Travis pp. 28-29 refers to the goal-state/s in TM as spiritual experience/s. "The author defines spiritual experiences as those of wholeness ..." – p. 28

2.7.38 Inner security, safety, strength, contentment
See also 1.3 ("Pleasant"), 2.7.24 ("Calm, ease, peacefulness, absence of disturbance"), and 2.7.26 ("Bliss, joy, happiness").

Pearson (2013)	"... an inner sanctuary of silence, peace, and joy." – p. 175<br>Quoting a meditator discussing pure consciousness as encountered in daily life: "... inner feeling of invincibility or bliss." (p. 185); "I am connected to the universal value of life. Things don't disturb that level of life. Not only is there a feeling of strength, invincibility, and bliss, but also the feeling of great comfort. I feel that I am taken care of ..." (p. 185).
Roth (2018)	The book is titled "Strength in Stillness".<br>"... a field of satisfaction and happiness ..." – p. 34<br>"What does it feel like to meditate? ... Mentally, it's calming yet awakening – and deeply satisfying" – p. 58<br>Head of the TM organization, Dr Tony Nader, describing his first TM session/s: "Right away, I experienced ... transcendence ... It was tremendously satisfying." – p. 193
Shear (2006b)	Referring to the experience of pure consciousness in TM and other meditation traditions: "... [S]ince ... one feels quite contented or 'full' after experiencing it, it is also often referred to as pure 'fullness'." – p. xix
Rosenthal (2011/2012)	Quoting Jonah, a meditator: "When I got my mantra and transcended, I was able to connect with that deep, silent, blissful, fulfilling, absolute level of life. I felt satisfied." – p. 169<br>Quoting Roth: "... an incredibly satisfying place to 'be' ..." – p. 278
Travis et al. (2005)	Quoting a meditator: "... an inner peace that is very, very nourishing ... I feel right at home ... Everything seems right." – p. 128

2.7.39 Inner freedom, unboundedness

Pearson (2013)	"... the experience of infinite pure consciousness experienced during [TM] ..." – p 32<br>"... unbounded pure awareness ..." – p. 41<br>"... unbounded awareness, the fully expanded state of mind ..." – p. 41<br>"... the unbounded Self." – p. 41<br>Quoting Maharishi Mahesh Yogi: "... just pure consciousness aware of its own unbounded nature." – p. 45<br>"... [C]onsciousness expands to the maximum." – p. 45<br>"One experiences consciousness in its pure state, infinite and unbounded." – p. 45<br>Pearson pp. 46, 47, 48, 50, 177, 321 also refers to unboundedness.<br>"Pure consciousness is unbounded, universal." – p. 50<br>Quoting a meditator: "... experiencing awareness as an unbounded unity." – p. 52<br>Quoting a meditator: "... I slip into an unbounded ocean of awareness ...." – p. 52<br>Quoting a meditator: "The experience is one of evenness and expansion, of infinity, and I am that infinity." – p. 52<br>Under the heading "Freedom and liberation", Pearson p. 177 says: "... [Pure consciousness] brings the liberation of unbounded awareness. This freedom becomes perpetual in Cosmic Consciousness, with the mind ever awake in its unbounded reality."<br>Quoting a meditator: "I began to experience it not just as a state with no thought but rather as having no boundaries; then as unbounded, beyond the limitations of my individuality ..." – p. 183<br>"... [P]ure consciousness ... gives the experience of ... freedom from limitations ..." – p. 396
Roth (2018)	"... an unbounded expanse of peace and tranquility ..." – p. 11<br>"The purpose of TM is to open the door to this unbounded field." – p. 17<br>Roth pp. 42, 119 also refers to unboundedness.<br>Quoting Deborah-lee Furness: "When I meditate, I'm joining my true self, which is this calm, peaceful, blissful, limitless self." – p. 78<br>Head of the TM organization, Dr Tony Nader, describing his first TM session/s: "Right away, I experienced ... transcendence – a deep inner calm, a deep inner bliss and a great expansion of awareness." – p. 193
Shear (1990b)	Shear frequently refers to unboundedness – e.g., p. 136.

	Quoting a meditator describing what Shear refers to as an "advanced experience": "One of the most regular experiences in my meditation is of expanded awareness, of no longer being confined to the inside of my head, but being as infinite or more infinite than the universe. Sometimes I feel the boundaries of the mind being pushed out, like the ever-widening circumference of a circle, until the circle disappears and only infinity remains." (p. 230); "It is a feeling of great freedom ... Sometimes the sense of infinity is so strong that I lose the sensation of body or matter – just infinite, unbounded awareness, an eternal, never-changing continuum of consciousness." (p. 230).
Shear (1990a)	Referring to "introvertive mystical experience", which Shear presents as equivalent to pure consciousness: "... [T]he experience traditionally is often reported as experienced in close association with (e.g., immediately before and/or after) other paradigmatic introvertive mystical experiences (unboundedness, bliss, sense of the divine, etc.), and is often held to be the ground of full development of these other experiences." – p. 398
Shear (2006b)	"Tradition after tradition reports that the experience is intimately associated with profound experiences of bliss, happiness, and freedom." – p. xix
Rosenthal (2016/2017)	"... stillness, expansiveness, boundlessness, and peace ..." – p. 5<br>"... inner expansive calm ..." – p. 5<br>"What, then, are the usual elements of transcendence? That was the question I asked a group of students, at Loyola University's Stritch School of Medicine in Chicago, who had learned to meditate. They replied with gusto: stillness, quiet, no boundaries, no thoughts, and bliss. In short, they captured the essential spirit of the state." – p. 31
Rosenthal (2011/2012)	"During a state of transcendence, people commonly lose their sense of boundaries (where you begin and end). One ten-year-old boy ... told me that once during a session he forgot where his hands were, only to discover later that they were on the floor propping him up." – pp. 37-38<br>Quoting Ronald, a meditator: "It's a joyful little dream for twenty, thirty minutes. I'm relaxed, free." – p. 195<br>Quoting Roth: "... [T]here is a level, deep within the mind, that is ever silent, settled, fully expanded, and fully alert." – p. 277
Travis and Pearson (2000)	When asked to describe their deepest experiences in TM, 20% of participants referred to them as unbounded (p. 81).<br>Quoting a participant: "... [A] couple of times per week I experience deep, unbounded silence ..." – p. 81
Travis et al. (2005)	Quoting a meditator: "It's a feeling of freedom, of no restraints. In this state boundaries do not exist." – p. 128
Travis (2014)	"Silence, expansion, and evenness begin to dominate awareness ..." – p. 3

2.7.40 Spacelessness

Pearson (2013)	"It lies ... beyond individuality, time, and space." – p. 25<br>Quoting a meditator: "... I slip into an unbounded ocean of awareness which is ... infinitely extended beyond space and time." – p. 52<br>"... untouched by anything in time and space." – p. 261<br>"... beyond time and space." – p. 265
Roth (2018)	Quoting George Harrison from the Beatles: "... [T]hat level is timeless, spaceless ..." – p. 41
Shear (1990b)	"... independent of all spatiotemporal orientations and distinctions ..." – p. 46<br>"... awareness independent of ... spatio-temporal awareness ..." – p. 46<br>"... devoid of any empirical, spatiotemporally identifiable content ..." – p. 102<br>Shear pp. 104, 163, 198, 223 also refers to the absence of spatiotemporal content.<br>"... [T]his experience has no parts or components (indeed, even the manifolds of space and time, the very contexts in which parts can be distinguished, are not present in the experience) ..." – p. 105

Shear (1990a)	Referring to “introvertive mystical experience”, which Shear presents as equivalent to pure consciousness: “... [N]ot only is the experience described as devoid of spatio-temporal content, but by all accounts even spatiality and temporality themselves are not present in it.” – p. 392
Shear (2006b)	“... [I]t has no content in it at all – no perceptions, feelings, thoughts, or anything else – not even, indeed, any time or space in which such things could be located.” – p. xviii
Shear (2006c)	“... [T]here is not even any internal place or ‘space’ – any here or there, up or down, or any ‘where’ at all – in which any [images, sensations, thoughts or feelings] could be located.” – p. 33
Shear (2007)	“... [Pure consciousness] is devoid even of any subjective ‘space’ in which phenomenal objects could appear.” – p. 700
Shear (2011b)	“... [E]ven phenomenological space is absent from the experience ...” – p. 52
Rosenthal (2016/2017)	“Just as our thoughts come and go, so (at times) do other boundaries, such as our sense of time and space.” – p. 35
Rosenthal (2011/2012)	“... I may experience transcendence – a feeling that I have gone beyond time and space.” – p. 18<br>“... [T]he sense of time, place, or date may ... go missing.” – p. 38<br>“It is the profoundly relaxed alertness free of specific thoughts, along with the loss of personal boundaries of time or space, that are the essential elements of transcendence.” – pp. 42-43
Travis and Pearson (2000)	When asked to describe their deepest experiences in TM, 68% of participants referred to them as involving an “absence of space, time, or body-sense” (p. 81).<br>Quoting a participant: “There is no awareness of where I am ...” – p. 81
Travis et al. (2005)	Quoting a meditator: “Space has no meaning.” – p. 128<br>“There is no contrast ... to mark distance between objects ...” – p. 128

2.7.41 Timelessness

Pearson (2013)	“It lies ... beyond individuality, time, and space.” – p. 25<br>Quoting a meditator: “... I slip into an unbounded ocean of awareness which is ... infinitely extended beyond space and time.” – p. 52<br>“... untouched by anything in time and space.” – p. 261<br>“... beyond time and space.” – p. 265
Roth (2018)	Quoting George Harrison from the Beatles: “... [T]hat level is timeless, spaceless ... So you even don’t know how long you have been there.” – p. 41<br>“Many people tell me they feel that time passes quickly.” – p. 58
Shear (1990b)	“... independent of all spatiotemporal orientations and distinctions ...” – p. 46<br>“... awareness independent of ... spatio-temporal awareness ...” – p. 46<br>“... devoid of any empirical, spatiotemporally identifiable content ...” – p. 102<br>Shear pp. 104, 163, 198, 223 also refers to the absence of spatiotemporal content.<br>“... [T]his experience has no parts or components (indeed, even the manifolds of space and time, the very contexts in which parts can be distinguished, are not present in the experience) ...” – p. 105
Shear (1990a)	Referring to “introvertive mystical experience”, which Shear presents as equivalent to pure consciousness: “... [N]ot only is the experience described as devoid of spatio-temporal content, but by all accounts even spatiality and temporality themselves are not present in it.” – p. 392
Shear (2006b)	“... [I]t has no content in it at all – no perceptions, feelings, thoughts, or anything else – not even, indeed, any time or space in which such things could be located.” – p. xviii
Rosenthal (2016/2017)	“Just as our thoughts come and go, so (at times) do other boundaries, such as our sense of time and space ...” – p. 35

	Quoting Elaine, a meditator: "There is quite often time that I 'lose', becoming aware of returning to myself ... Not wanting the experience to end, I'll sometimes go back in for a few minutes before opening my eyes. When this happens, I'm surprised that it is time to stop ..." – p. 217
Rosenthal (2011/2012)	"... I may experience transcendence – a feeling that I have gone beyond time and space." – p. 18<br>"... [T]he sense of time, place, or date may ... go missing. As one successful businessman ... said, 'Transcendence is timeless. Before I know it, the twenty minutes are up." – p. 38<br>"It is the profoundly relaxed alertness free of specific thoughts, along with the loss of personal boundaries of time or space, that are the essential elements of transcendence." – pp. 42-43
Travis and Pearson (2000)	When asked to describe their deepest experiences in TM, 68% of participants referred to them as involving an "absence of space, time, or body-sense" (p. 81).<br>Quoting a participant: "There is no awareness of ... the passage of time." – p. 81
Travis et al. (2005)	Quoting a meditator: "Time has no meaning." – p. 128<br>"There is no contrast to mark the passage of time ..." – p. 128

2.7.42 Knowledge

Pearson (2013)	Pearson p. 39 notes that one of the "basic principles of states of consciousness" put forward by Maharishi Mahesh Yogi was that "[k]nowledge is different in different states of consciousness". "Each [of the seven states] involves a unique mode of knowledge and experience, of ourselves and the surrounding world." – p. 39<br>"Ordinary waking consciousness brings us knowledge of specific things ... [Pure consciousness] brings the experience of what Maharishi calls *pure knowledge* – the knowledge gained when consciousness knows itself, by itself, in its pure unbounded state ... This is the 'absolute state of knowledge', a state of knowing*ness*." – p. 47<br>"Maharishi has called [pure consciousness] an ocean of *pure knowledge, power, and bliss*." – p. 48
Rosenthal (2011/2012)	Quoting a meditator: "During transcendence I experience a feeling of total calmness and peace. I also experience a knowing that is very beautiful. I think this knowing is a result of being connected to the absolute aspect of existence. It is as if 'I' exist and yet there is another 'I' which exists and is eternal and totally without anxiety or involvement in the relative world." – p. 39

2.7.43 Possible spiritual aspect

Pearson (2013)	"Maharishi distinguishes two aspects of God – impersonal and personal." – p. 264 "... [T]he impersonal aspect of God is pure Being, the unbounded Absolute – pure consciousness." – p. 264 For completeness, note that Pearson says: "Experiencing the personal aspect of God, on the other hand, is a matter of *sensory* experience in [daily life]." – p. 265 As such, the personal aspect is not relevant for the present table.
Shear (2011a)	Shear p. 146 notes that "there is wide agreement among the major [meditation] traditions" that pure consciousness (and certain other higher states) "represent important advanced stages of spiritual growth".
Rosenthal (2011/2012)	"... [M]any meditators ... have a sense of being connected to something greater or more profound in the universe. For example, one doctor in his late fifties described transcendence as 'a touch of heaven', while a psychologist in her sixties ... offered the following description: 'During transcendence I experience a feeling of total calmness and peace. I also experience a knowing that is very beautiful. I think this knowing is a result of being connected to the absolute aspect of existence. It is as if 'I' exist and yet there is another 'I' which exists and is eternal and totally without anxiety or involvement in the relative world.'" – pp. 38-39<br>"One twelve-year-old at the Maharishi grade school for children in Fairfield, Iowa, wrote, 'When I meditate, it's like I'm floating up to heaven or I'm there already'." – p. 41<br>"... [T]ens of thousands of people are recognizing TM as a simple and powerful method for addressing their spiritual longings." – p. 253
Travis (2009)	Travis pp. 28-29 refers to the goal-state/s in TM as spiritual experience/s. "The author defines spiritual experiences as those of wholeness – those of our universal nature, or that part of us not tied to time, space, or our individual body or personality." – p. 28

Heaton and Travis (2014)	"This inner state has been identified with the spiritual essence of life ..." – p. 18

2.7.44 Possible energy

Rosenthal (2016/2017) quotes a meditator, Elaine, who says: "... I seem to melt into [the silence] ... Then ... I start to experience an energy running through me, almost like a cell phone being charged" (p. 217). Some other aspects of Elaine's description of her meditation experience fit with the descriptions of pure consciousness provided by the experts. However, Elaine also says in her description, "I'm aware of what is going on around me" (p. 217), which is inconsistent with the experts' statements that the experience does not involve sense-perceptions (see 2.7.18, "No sense-perceptions"). That comment gives reason to query whether Elaine's experience of energy is a description of a goal-state or an interim-state. Rosenthal (2016/2017) also quotes a meditator, Roger, who refers to some of his meditation experiences as involving a sense of energy: "... [I]t felt as though [an] energy flow was entering my head, flowing through my body and over its surface." – p. 222 Rosenthal p. 221 says that this experience is "quite unusual". Roger's references to the energy flowing through his body are inconsistent with the experts' statements that pure consciousness does not involve body-perceptions (see 2.7.19, "No body-perceptions"). That suggests that his experience might be better treated as an interim-state rather than a goal-state. Some of Roth's statements in Rosenthal (2011/2012) also indicate that energy is in some sense an aspect of pure consciousness. For example, he says: "We infer that deep within the mind ... is a reservoir of energy, intelligence and happiness" (p. 278); and that, as one practices, their "attention moves to increasingly subtle, quiet, creative, and energetic levels of the mind" (p. 278). Statements such as these, however, do not necessarily mean that a meditator would report that in the goal-state/s they experienced energy as a feature distinct from the other experiential qualities identified in this table.

2.7.45 Profound

See also 2.7.48 ("Subjective experience varies between meditators and across/within sessions").

Pearson (2013)	Quoting a meditator: "Then something quite extraordinary happens. The ocean [of consciousness] disappears. I mean it is just gone, and I am in an indescribable place." – p. 53
Roth (2018)	"... [T]ranscendence ... doesn't come simply by making changes in our daily routine on a horizontal level ... Real transcendence is more profound than that. It's vertical." – p. 42<br>"Sometimes the experience in [TM] is profound; oftentimes it can seem mundane." – p. 45<br>Quoting Jerry Seinfeld: "When I tell people I get up to [do TM], they ask me how it feels. It doesn't feel like much of anything." – p. 48<br>"It was both unique and as familiar and natural as could be." – p. 152
Shear (1990b)	Referring to pure consciousness and the higher states: "... remarkable experiences ..." – p. 1
Shear (1996b)	"... profound settling of mental activity ..." – p. 367
Shear (2006c)	"... [P]roper practice of [TM] produces [a] refinement [in mental activity] independently of whether or not one happens to have any particular 'flashy' experience. Moreover, since the experiences produced by the refined activity ... can be highly abstract, and therefore, at first, hardly noticed (much less remembered), a great deal of refinement can take place without one's noticing any remarkable experiences in meditation at all." – p. 32 "What one *is* likely to notice with proper practice, however are ..." – p. 32 Shear p. 32 lists a number of facets of personal growth experienced in daily life. He then says: "Flashy experiences ... are not methods for such growth." – p. 32 "Nevertheless, specific, easily recognizable experiences of deep levels of consciousness do often occur during the practice of [TM] ..." – pp. 32-33<br>"Quite aside from ... advanced experiences [of higher states of consciousness in daily life that can flow from the experience of pure consciousness in TM] ... the growth of consciousness the average person can gain quickly from the practice of the basic TM technique is remarkable enough." – p. 42 Shear pp. 42-43 describes various aspects of that growth. "... [T]hese are the sorts of things that develop spontaneously, bit by bit, day by day, when one has begun the practice." – p. 43
Shear (2014b)	Shear p. 202 describes pure consciousness as "remarkable", and as "highly unusual" compared to ordinary states of consciousness. Shear p. 213 also refers to it as "highly unusual".
Rosenthal (2016/2017)	Describing his first experience of transcendence: "It was a state both thrilling in its novelty yet strangely familiar." – p. 31<br>"My own transcendent experiences and those of most of my patients are pleasant but unsensational." – p. 39<br>Quoting Roth: "I must emphasize that transcending is not a flashy experience. It is quite simple and natural. We are just settling down to our own quiet, inner, unbounded self." – p. 261

Rosenthal (2011/2012)	"For some people even their first session can be an epiphany. David Lynch, in his book *Catching the Big Fish*, compares the experience of his first TM session to cutting an elevator cable and falling into bliss." – p. 24<br>Referring to Martin Scorsese: "When Marty starts to meditate, there are 'a thousand thoughts, which eventually settle down into a calm, floating space. It is an extraordinary experience.'" – p. 227

2.7.46 Deep

See also 2.7.36 ("Nature of mind/being, ground state, self/Self") and 2.7.48 ("Subjective experience varies between meditators and across/within sessions").

Pearson (2013)	"... a state of deep inner silence and peace ..." – p. 29<br>"When one first learns to meditate, one may experience [pure consciousness] only for brief moments, at the deepest points of meditation. The experience is so natural that one may be hardly aware of it. In time, with regular practice, one maintains the experience for longer periods ..." – p. 51<br>"... [W]ith repeated transcending, stress dissolves and the nervous system becomes more flexible and refined. This allows deeper and clearer experiences of [pure consciousness], more frequent and longer-lasting. Eventually the nervous system becomes virtually free of stress." – p. 175<br>Quoting a meditator: "Gradually over the years, as the experience of pure consciousness became increasingly familiar in [TM], I began to experience it not just as a state with no thought but rather as having no boundaries; then as unbounded, beyond the limitations of my individuality; then as the unbounded, unchanging essence of my existence ..." – p. 183<br>Quoting a meditator: "The experience of bliss consciousness has become more clear, intense, and stable not only during [TM] but also during activity." – p. 184<br>Pearson p. 265 indicates that pure consciousness is experienced "in the deepest moments of transcendence".<br>"Physiological stress impedes transcending ... As [TM] enables the mind to transcend, in the same stroke it allows the body to dissolve the stresses that hinder transcending." – p. 396
Roth (2018)	"... [D]eep within is a level that is calm yet alert; silent yet wide awake." – p. 17<br>"... state of inner stillness at the deepest level of awareness ..." – p. 17<br>"... the quietest, deepest level ..." – p. 34<br>Roth pp. 37, 55 also refers to pure consciousness as the deepest level.<br>"... the unbounded stillness that lies deep within." – p. 42<br>"... the deepest core of your own being." – p. 57
Shear (1995/1997b)	Shear p. 370 refers to the "deeper structures of consciousness" as emerging from pure consciousness.
Shear (2006b)	Shear p. xviii refers to pure consciousness as one of the "deepest levels of inner awareness".
Shear (2006c)	"... deep experience ..." – p. 24
Shear (2007)	Shear pp. 699-700 refers to pure consciousness as the deepest level of consciousness.
Rosenthal (2016/2017)	Quoting Elaine, a meditator: "There is quite often time that I 'lose', becoming aware of returning to myself ... Not wanting the experience to end, I'll sometimes go back in for a few minutes before opening my eyes." – p. 217
Rosenthal (2011/2012)	Quoting Jonah, a meditator: "When I got my mantra and transcended, I was able to connect with that deep, silent, blissful, fulfilling, absolute level of life. I felt satisfied." – p. 169<br>Quoting Roth: "... deep within the mind ..." – p. 277
Travis and Pearson (2000)	Quoting a participant: "... [A] couple of times per week I experience deep, unbounded silence ..." – p. 81
Travis (2006)	"It is the deepest aspect of our selves – called the Self." – p. 36

2.7.47 Non-changing

Pearson (2013)	"[Pure consciousness] ... [has] no content other than its own nonchanging, unbounded nature." – p. 50
Roth (2018)	"... a field of satisfaction and happiness that does not fluctuate .." – p. 34

Shear (1990b)	Quoting a meditator describing what Shear refers to as an "advanced experience": "... an eternal, never-changing continuum of consciousness." (p. 230).
Shear (1996b)	"... pure unfluctuating consciousness ..." – p. 367
Shear (2007)	"... non-fluctuating nature ..." – p. 704
Travis (2014)	"Silence, expansion, and evenness begin to dominate awareness ..." – p. 3
Heaton and Travis (2014)	"... [I]t transcends ... all relative, changing experience ..." – p. 18<br>Quoting Maharishi Mahesh Yogi: "... this field of non-change ..." – p. 24

2.7.48 Subjective experience varies between meditators and across/within sessions

See also 1.5.2 ("The technique is quick to learn/perfect"), 2.7.18 ("No sense-perceptions"), 2.7.45 ("Profound"), and 2.7.46 ("Deep").

Pearson (2013)	"Each person's experiences will be unique, depending on the ever-changing condition of their nervous system." – p. 54<br>"... [W]ith repeated transcending, stress dissolves and the nervous system becomes more flexible and refined. This allows deeper and clearer experiences of [pure consciousness], more frequent and longer-lasting. Eventually the nervous system becomes virtually free of stress." – p. 175<br>Quoting a meditator: "Gradually over the years, as the experience of pure consciousness became increasingly familiar in [TM], I began to experience it not just as a state with no thought but rather as having no boundaries; then as unbounded, beyond the limitations of my individuality; then as the unbounded, unchanging essence of my existence ..." – p. 183<br>Quoting a meditator: "The experience of bliss consciousness has become more clear, intense, and stable not only during [TM] but also during activity." – p. 184
Roth (2018)	Roth pp. 43-44 provides descriptions by Billie Jean King and William Wordsworth of experiences that were not attained in TM practice but that he considers, like TM, involved transcendence. "Does [transcendence] always feel like the ways that Billie Jean King or William Wordsworth describe it? Of course not. Sometimes the experience in meditation is profound; oftentimes it can seem mundane. But each time you meditate, even if your mind is filled with a million thoughts, you do settle down toward levels of the mind that are deeper, quieter, more satisfying." – p. 45<br>Quoting Jerry Seinfeld: "TM never doesn't work perfectly." – p. 91 "I agree with Jerry, but I have to add that *perfectly* is a tricky word. After you have been practising TM for even a few days, you will notice that every meditation is different ... The experience of transcending is fluid, and every session is influenced by the condition of the body at the time you meditate. I like to say that there is no single perfect experience in meditation – there are countless perfect experiences. People sometimes worry that they didn't have the 'best' meditation session. When asked about this, Maharishi used to say, 'Even in a shallow dive, you still get wet.'" – p. 92 "... [I]t's true that you cannot predict what any one particular meditation session may be like because your body is different each time you meditate (for example, Did you sleep well the night before? Eat a big meal right before meditation? Pull an all-nighter studying for an exam?) ..." – p. 95<br>"... [M]y experiences have continued to deepen ... over the nearly fifty years that I have continued to meditate." – p. 96
Rosenthal (2016/2017)	"... [T]he quality of your transcendent experiences during meditation may have no bearing whatsoever on how much benefit you get ... This is hard to believe because it is natural to think that exotic and esoteric experiences will do you the most good ... I remember being quite discouraged because I didn't have such experiences ... In time I would harvest the fruits, [a TM teacher-friend] assured me – and she was right." – p. 33 "Here are a few examples of people who report a similar disconnect between their banal experiences during TM and the dramatic results of their practice." – p. 33 Rosenthal notes that artist, Mandy Weisel, said that in her TM practice "mostly she has no thoughts at all, just a sense of openness and space, and a nice feeling that seems to be centered around the front of her head" (p. 34). He also quotes Jerry Seinfeld: "It doesn't feel like anything. It doesn't do anything. I don't get it." – p. 34 Rosenthal refers to the "powerful effect TM has on [Seinfeld's] day *despite* how uneventful the sessions themselves feel" (p. 34).<br>"The descriptions of [pure consciousness] that we've encountered – stillness, peace, unity, bliss – capture many of the standard aspects of the [state], but there are many exceptions. For example, some people report going into a state of awareness where awareness of any sort disappears – until consciousness returns – when, lo and behold! twenty minutes have passed. I know that sounds pretty much like a nap, but having

	experienced such ... states myself, I can say that when I emerge, I do not feel as if I have taken a nap. Although I cannot swear no z's slipped in, I am clearheaded in a non-postnap way, wide awake, and ready to get on with the day. Others have reported similar experiences." – p. 36<br>"Actually, Bob Roth tells me that many people rarely have experiences that can be readily identified as clear transcendence yet love their meditation practice because it produces such powerful and positive benefits in their lives." – p. 37<br>"Then there are those who have spectacular transcendent experiences ..." – p. 36<br>"Keep these thoughts in mind as you listen to vivid pyrotechnic descriptions, especially if all *you* may experience is stillness, quiet, bliss, and pure consciousness – which is what happens mostly for me (and which is really quite a lot, when you think of it). It has taken me years, in fact, to fully accept the value of this less spectacular form of transcendence, but now I do finally believe that its benefits are just as profound ..." – p. 37 "Even so, I must confess that a lingering trace of transcendence envy can crop up. Recently, that occurred when I was visiting with my friend Richard Friedman ... He was telling me about the copper-colored light that infuses his inner space as he practices TM. 'I also see phosphenes', he said ... My first instinct was to ask, 'Why don't I see phosphenes?'" – p. 37 "One famous example of a spectacular transcendent experience is described by iconic filmmaker and veteran meditator David Lynch, in his book *Catching the Big Fish*. According to him, the very first time he meditated, it felt as though the cable of an elevator in which he was riding had been severed. It was as though he literally fell into a pool of bliss. Some people reading that ecstatic description have felt inadequate in never having had such a thrilling experience during meditation, no less on their very first attempt." – p. 37<br>Quoting a patient, Moira: "As I am transcending, I often feel as though vibrant color is washing over me again and again. At other times I feel a sort of pulsating aurora borealis. I describe it that way because the hues are vivid with no hard edges and it moves upwards, filling me with beautiful color. When I have fully transcended, I don't remember too much. Sometimes ideas for my current paintings drift in and out, but mostly I experience a wonderful, restful feeling." – p. 38 Rosenthal p. 39 views Moira's experience as spectacular, presumably due to the colours etc.<br>"My own transcendent experiences and those of most of my patients are pleasant but unsensational." – p. 39<br>"Someone once asked [Maharishi Mahesh Yogi] if a deeper meditation is better than one that seems more on the surface. Maharishi replied that both are good. When asked why, he said, 'Because even in a shallow dive we get wet'." – p. 39<br>"The actual content of thoughts and experiences during the session is highly variable ..." – p. 39
Rosenthal (2011/2012)	"Although there is no such thing as a 'typical' TM session, certain qualities pervade every meditation: deep relaxation, inner peace, and (sometimes) a highly enjoyable shift in consciousness." – p. 18<br>"For some people even their first session can be an epiphany. David Lynch, in his book *Catching the Big Fish*, compares the experience of his first TM session to cutting an elevator cable and falling into bliss." – p. 24<br>"I should emphasize that such exuberant experiences – floating, splashing, diving into pure consciousness or visions of light – are by no means routine. Craig Pearson ... speaks to this point in describing his own experience with TM, which he has been practicing for the past forty years: 'There's definitely a diving deep within, a settling down of mental activity and deep integration. Do I experience clear, pure unbounded awareness in some of the extraordinary ways I've discovered it described by some of the great people through history? No, I've only had that experience a few times in my life. But there are people who sit around me in the Golden Dome [of the Maharishi University of Management in Iowa] who have that experience on a regular basis. And with continuing practice, these are the kinds of experiences everyone can have'." – p. 42 "Fortunately, four-star graphics are [not] a necessary part of transcendence ... It is the profoundly relaxed alertness free of specific thoughts, along with the loss of personal boundaries of time or space, that are the essential elements of transcendence ... If you see lights or find yourself splashing about in pure consciousness – as you may – it's a delightful bonus." – pp. 42-43<br>Referring to meditator Tom O'Connor: "When asked whether he transcends regularly during meditation, he's not sure. 'I have very pleasant experiences and I feel like there's a lot going on, but I don't know if I could say, 'That time I was transcending. This time I wasn't.'" – p. 201<br>"... [W]e cannot control what sort of transcendent experiences we are going to have – because that depends on the individual and the changing state of the nervous system. As you may recall, some regular TMers have never experienced something they can identify as [pure consciousness]. They close their eyes, think their mantra, and the next thing they know, their twenty minutes are up. Yet these people typically find TM just as fulfilling and transforming as those who splash around in pools of bliss or see lights hovering on the horizon. So, don't judge the value of TM for yourself by the quality of the transcendent experiences you may or may not have during meditation. It is *outside* of meditation that one truly enjoys the benefits of TM." – p. 275

2.7.49 Description in words is limited
See also 2.7.2 (“Cannot say much about it”).

Pearson (2013)	Quoting a meditator: “... [S]omething quite extraordinary happens. The ocean [of consciousness] disappears. I mean it is just gone, and I am in an indescribable place.” – p. 53 “As those who have had ... experiences [of pure consciousness and higher states] almost unanimously declare, they are fundamentally ineffable, indescribable, beyond language – they can be known and understood only through the experience itself.” – p. 55 “Utterly beyond words – yet [historical figures] strove to describe [the experiences] nonetheless. They may declare they failed, but they succeeded in fashioning beautifully-wrought signposts to exalted stages of human development.” – p. 55
Roth (2018)	“... [F]or the rest of us non-poets, transcendence can be a challenge to put into words. What does it feel like? It’s like trying to describe happiness in words. Or telling someone who has never eaten a kiwi what it tastes like ... [Y]ou will never fully convey the experience precisely through words. It must be experienced firsthand.” – p. 45
Shear (1990a)	Referring to “introvertive mystical experience”, which Shear presents as equivalent to pure consciousness: “The extreme abstractness of the relevant descriptions, however, makes them by themselves less than ideal evidence; for the notion of an empirically qualityless experience is likely to appear less than fully intelligible – if not completely unintelligible – to one who has not had such an experience.” – p. 396
Shear (2011a)	“... beyond the grasp of the intellect ...” – p. 148
Rosenthal (2011/2012)	“How is it possible to convey a feeling or state of consciousness if the person you are talking to has not had the experience?” – p. 37 Referring to one description of transcendence, Rosenthal says: “One curious aspect of this quote is that, even while observing how hard it is to describe transcendence, it does quite a good job of doing just that. We learn that transcendence ...” – p. 37

Note. Version 1.0. There is a degree of overlap or interrelationship between the aspects of the technique, interim-states and goal-state/s described in the different sections of the table. For a proper understanding of each aspect, the relevant section/s should therefore be considered in the context of the table as a whole. To assist in that regard, where a particular section is closely linked to other sections, cross-references have been provided. The headings for each section indicate its subject matter or theme, and aim to capture the main thrust or gist of its content. They do not necessarily capture aspects outside that main thrust or gist, such as statements of a marginal and/or qualifying nature.

References

Arenander, A., and Travis, F. (2004). “Brain patterns of self-awareness,” in *Deficits of self-awareness in psychiatric disorders*, eds. B. Beitman and J. Nair (New York, NY: Norton), 112–126.

Faber, P. L., Travis, F., Milz, P., and Parim, N. (2017). EEG microstates during different phases of Transcendental Meditation practice. *Cognitive Processing* 18, 307–314. doi:10.1007/s10339-017-0812-y.

Gobec, S., and Travis, F. (2018). Effects of Maharishi Yoga Asanas on mood states, happiness, and experiences during meditation. *International Journal of Yoga* 11, 66–71. doi:10.4103/ijoy.IJOY_66_16.

Heaton, D. P., and Travis, F. (2014). “Consciousness, empathy, and the brain,” in *Organizing through empathy*, eds. K. Pavlovich and K. Krahnke (New York, NY: Routledge), 17–33.

Heaton, D. P., Travis, F., and Subramaniam, R. (2012). “A consciousness-based approach to management education for integrity,” in *Handbook of research on teaching ethics in*

business and management education, eds. C. Wankel and A. Stachowicz-Stanusch (Hershey, PA: IGI Global), 65–79.

Pearson, C. (2013). *The supreme awakening: Experiences of enlightenment throughout time – and how you can cultivate them*. Fairfield, IA: Maharishi University of Management Press.

Rosenthal, N. E. (2011/2012). *Transcendence: Healing and transformation through Transcendental Meditation*. New York, NY: Penguin.

Rosenthal, N. E. (2016/2017). *Super Mind: How to boost performance and live a richer and happier life through Transcendental Meditation*. New York, NY: Penguin.

Roth, B. (2018). *Strength in stillness: The power of Transcendental Meditation*. London, United Kingdom: Simon & Schuster.

Shear, J. (1990a). Mystical experience, hermeneutics, and rationality. International *Philosophical Quarterly* 30, 391–401. doi:10.5840/ipq199030436.

Shear, J. (1990b). *The inner dimension: Philosophy and the experience of consciousness*. 2nd ed. London, United Kingdom: Harmonia.

Shear, J. (1994). On mystical experiences as support for the perennial philosophy. *Journal of the American Academy of Religion* 62, 319–342. doi:10.1093/jaarel/lxii.2.319.

Shear, J. (1996b). "On the existence of a culture independent core component of self," in *East-west encounters in philosophy and religion*, eds. N. Smart and B. S. Murthy (Long Beach, CA: Long Beach), 359–376.

Shear, J. (1995/1997b). "The hard problem: Closing the empirical gap," in *Explaining consciousness – The "hard" problem*, ed. J. Shear (Cambridge, MA: MIT Press), 359–375.

Shear, J. (1998/1999). "Experiential clarification of the problem of self," in *Models of the self*, eds. S. Gallagher and J. Shear (Devon, United Kingdom: Imprint), 407–420.

Shear, J. (2002). "Ethics and the experience of happiness," in *Crossing boundaries: Essays on the ethical status of mysticism*, eds. G. W. Barnard and J. J. Kripal (New York, NY: Seven Bridges Press), 361–380.

Shear, J. (2001/2002). "Experimental studies of meditation and consciousness," in *Religion and psychology: Mapping the terrain*, eds. D. Jonte-Pace and W. B. Parsons (London, United Kingdom: Routledge), 280–294.

Shear, J. (2004). Mysticism and scientific naturalism. *Sophia* 43, 83–99. doi:10.1007/bf02782439.

Shear, J. (2006a). "Conclusion," in *The experience of meditation: Experts introduce the major traditions*, ed. J. Shear (St. Paul, MN: Paragon House), 259–268.

Shear, J. (2006b). "Introduction," in *The experience of meditation: Experts introduce the major traditions*, ed. J. Shear (St. Paul, MN: Paragon House), xiii–xxii.

Shear, J. (2006c). “Transcendental Meditation,” in *The experience of meditation: Experts introduce the major traditions*, ed. J. Shear (St. Paul, MN: Paragon House), 23–48.

Shear, J. (2007). “Eastern methods of investigating mind and consciousness,” in *The Blackwell companion to consciousness*, eds. M. Velmans and S. Schneider (Malden, MA: Blackwell), 697–710.

Shear, J. (2011a). “Eastern approaches to altered states of consciousness,” in *Altering consciousness: Multidisciplinary perspectives*, eds. E. Cardeña and M. Winkelman (Santa Barbara, CA: Praeger), 139–158.

Shear, J. (2011b). State-enlivening and practice-makes-perfect approaches to meditation. *Biofeedback* 39, 51–55. doi:10.5298/1081-5937-39.2.07.

Shear, J. (2014a). “Meditation as first-person methodology: Real promise–and problems,” in *Meditation – Neuroscientific approaches and philosophical implications*, eds. S. Schmidt and H. Walach (Cham, Switzerland: Springer International), 57–74.

Shear, J. (2014b). Some reflections on meditation research and consciousness studies. *Journal of Consciousness Studies* 21, 202–215.

Shear, J., and Jevning, R. (1999a). Pure consciousness: Scientific exploration of meditation techniques. *Journal of Consciousness Studies* 6, 189–210.

Travis, F. (2001). Autonomic and EEG patterns distinguish transcending from other experiences during Transcendental Meditation practice. *International Journal of Psychophysiology* 42, 1–9. doi:10.1016/s0167-8760(01)00143-x.

Travis, F. (2006). “From I to I: Concepts of self on an object-referral/self-referral continuum,” in *The concept of self in psychology*, ed. A. P. Prescott (New York, NY: Nova Science), 21–43.

Travis, F. (2009). Brain functioning as the ground for spiritual experiences and ethical behavior. *FBI Law Enforcement Bulletin* 78, 26–32. doi:10.1037/e511562010-007.

Travis, F. (2011). “States of consciousness beyond waking, dreaming and sleeping: Perspectives from research on meditation experiences,” in *States of consciousness: Experimental insights into meditation, waking, sleep and dreams*, eds. D. Cvetkovic and I. Cosic (Berlin, Germany: Springer), 223–234.

Travis, F. (2014). Transcendental experiences during meditation practice. *Annals of the New York Academy of Sciences* 1307, 1–8. doi:10.1111/nyas.12316.

Travis, F. (2016). Transcending as a driver of development. *Annals of the New York Academy of Sciences* 1373, 72–77. doi:10.1111/nyas.13071.

Travis, F. (2018). “Long-term changes in experienced yoga practitioners: Growth of higher states of consciousness,” in *Research based perspectives on the psychophysiology of yoga*, eds. S. Telles and N. Singh (Hershey, PA: IGI Global), 35–51.

Travis, F., Munly, K., Olson, T. M., and Sorflaten, J. W. (2005). Maharishi Vedic science addresses the “hard” problem of consciousness. *Journal of Social Behavior and Personality* 17, 123–135.

Travis, F., and Parim, N. (2017). Default mode network activation and Transcendental Meditation practice: Focused attention or automatic self-transcending? *Brain and Cognition* 111, 86–94. doi:10.1016/j.bandc.2016.08.009.

Travis, F., and Pearson, C. (2000). Pure consciousness: Distinct phenomenological and physiological correlates of “consciousness itself”. *International Journal of Neuroscience* 100, 77–89. doi:10.3109/00207450008999678.

Travis, F., and Shear, J. (2010a). Focused attention, open monitoring and automatic self-transcending: Categories to organize meditations from Vedic, Buddhist and Chinese traditions. *Consciousness and Cognition* 19, 1110–1118. doi:10.1016/j.concog.2010.01.007.

Travis, F., and Shear, J. (2010b). Reply to Josipovic: Duality and non-duality in meditation research. *Consciousness and Cognition* 19, 1122–1123. doi:10.1016/j.concog.2010.04.003.

Varela, F. J., and Shear, J. (1999b). First-person methodologies: What, why, how? *Journal of Consciousness Studies* 6, 1–14.
