# Supplementary material for: Silence in Shamatha, Transcendental, and Stillness Meditation: An Evidence Synthesis Based on Expert Texts
Source: Front Psychol. 2020 Jul 8;11:1259. doi: 10.3389/fpsyg.2020.01259 (PMC7360996; doi:10.3389/fpsyg.2020.01259)
Supplement: Supplementary file 11 [file Data_Sheet_11.pdf]

Database Table S3: Stillness Meditation Extraction Table

Table S3

Stillness Meditation Extraction Table

Part 1: Technique and Interim-states

1.1 References to Stillness Meditation

Stillness Meditation is a term coined by McKinnon to describe the practice developed by Meares. Meares referred to the practice using a variety of terms. For example, in his first book on the approach (Meares, 1967/1968) he described it as "relaxing mental exercises". In the mid-1970s he adopted the term *Mental Ataraxis*, in order to distinguish it from other types of meditation (Meares, 1976b, 1978/1986, pp. xi-xii, 6). He explained that "ataraxis" is a Greek word meaning "absence of disturbance". Later, Meares ceased referring to Mental Ataraxis, on the basis that the term was not well understood (Meares, 1989, pp. 108-109). From the late 1970s onwards, he often described the approach simply as "meditation". McKinnon generally refers to the practice as either Stillness Meditation, or Stillness Meditation Therapy (SMT). For clarity, the term Stillness Meditation is mainly used in this table.

1.2 Simple

Meares (1967/1968)	"[The practice] may seem almost too simple to believe." – p. xiv<br>"... relatively simple procedure ..."– p. xxi<br>"... simple in the extreme." – p. 57<br>"... very simple ..." – p. 74
Meares (1978/1986)	"Very simple." – p. 23<br>"... simple ..." – pp. x, 3<br>"... process of extreme simplicity ..." – p. 6<br>"[Stillness Meditation] is in fact something very simple." – pp. 149-150
McKinnon (1983/2016)	"Utter simplicity [is] all that [is] required ..." – p. 191
McKinnon (2011)	"Perhaps the greatest attribute of [Stillness Meditation] is its absolute simplicity and naturalness." – p. 33<br>"[Stillness Meditation] is of the utmost simplicity ..." – pp. 39<br>McKinnon p. 75 makes a similar comment.<br>"I can't emphasise enough the simplicity and naturalness of this meditative approach." – p. 66<br>Quoting a client, "Rachael": "... such a simple easy-to-practise approach ..." – p. 181

1.3 Easy

Meares (1967/1968)	"The exercises are not difficult." – p. 74<br>"We practice ... until we can do it quite easily and naturally." – p. 161
Meares (1978/1986)	"... [A]lmost everyone can learn without much difficulty ..." – p. x<br>"... [A]lmost anyone can learn." – p. 3<br>Stillness Meditation is effortless – "No effort. No trying. No striving ... Utterly easy." – p. 23<br>"... [T]here is this easy, natural experience of letting ourself go with the relaxation without restraint." – p. 25<br>"In [Stillness Meditation] it is very easy to acquire the self discipline of transcending physical discomfort." – p. 50<br>"In learning how to transcend the discomforts of daily life through meditative experience we do not have to tolerate any unpleasantness, discomfort or hurt at all ..." – pp. 52-53 "In the meditative approach the individual experiences only potential discomfort ..." – p. 53
Meares (1969c)	"The whole process is quite effortless. And because of this it feels good. We enjoy doing it." – p. 84
Meares (1973b)	"[Transcending discomfort] is something ... which anyone with a little patience can soon muster." – p. 253<br>"[Learning the relaxation of our muscles, the experience of that relaxation in our mind, and letting our mind transcend minor discomfort] takes a little time and a little practice. We need to strive for most things that

	are worth while. So it is with this. But remember that in this the striving has this utterly effortless quality about it which means that it is no striving at all." – p. 254<br>"It is essential to [assume] a posture that is potentially uncomfortable. I say 'potentially uncomfortable' because our mind transcends the discomfort, and then there is no discomfort at all." – p. 255
Meares (1989)	"[Stillness Meditation] is quite easy and simple ..." – p. 107
Meares (1988)	"But when we say that it is 'easy' we must not think of it as 'slack'. No. That is not it at all. A firm discipline, but being quite easy and relaxed about it in our own self." – p. 96
McKinnon (2011)	"You can be reassured that to learn this very natural Stillness Meditation is not arduous ..." – p. 69<br>"[I]n approaching this practice, *think* simplicity and naturalness. It's not complicated in any way – it's all easy!" – p. 69<br>Quoting a client, "Amy": "... perfectly natural and all so easy." – p. 177<br>Quoting a client, "Rachael": "... such a simple easy-to-practise approach ..." – p. 181<br>Quoting "Brian", a former patient of Meares: "Not easy at first, but after some months something happened and I became a different person. It was like magic." – p. 191<br>"Stillness Meditation is easy." – p. 230
McKinnon (1991)	"[Stillness Meditation] is easily learned ... It does not require years of rigorous training to attain levels of perfection far beyond the reach of the average person; nor does it require ... retreat from the real world." – p. 71<br>"The [young] children I have worked with ... have afterwards volunteered such remarks as, '... it's really peaceful, very easy ...'." – p. 129<br>"... [Stillness Meditation] is *easy*, *pleasant* ... wonderful ..." – p. 133

1.4 Pleasant
See also 2.3.19 ("Good/wonderful").

Meares (1978/1986)	"... [W]e do not have to tolerate any unpleasantness ..." – p. 53
Meares (1969c)	"The calm and the ease of it is a pleasant sensation. The exercises are not a chore, something that you would like to put off or avoid, they are something to look forward to and enjoy." – p. 87
Meares (1989)	"[Stillness Meditation] is ... in fact ... quite pleasant" – p. 107<br>"[Stillness Meditation] is really a very natural, easy, pleasant way of letting [the mind] work." – p. 108
Meares (1987/1991)	"As we learn [Stillness Meditation] it soon becomes a pleasant experience. It is something to which we look forward. This comes with the ease that there is about it. There is no making ourselves relax, no making ourselves meditate. It is all very simple and natural. That is why we soon come to like doing it." – pp. 116-117 Meares p. 122 makes a comment along the same lines.
Meares (1976/1980)	"Self-discipline of [the kind in Stillness Meditation] is not in any way an unpleasant exercise. In fact the feeling of easy control of ourselves in itself brings a sense of satisfaction and inner pleasure." – p. 146
Meares (1983a)	Referring to patients with cancer who have used Stillness Meditation: "Those patients who come to like meditating do best. There are two aspects to this. Patients like meditation because of the calm and relief from distress and pain which follows after the meditation. But beyond this, there is also a liking of the experience itself." – p. 119
McKinnon (1983/2016)	"... pleasant feeling of deliberate effortless *being* ..." – p. 195
McKinnon (2011)	"As one woman ... recently stated: 'I look forward so much to my daily stillness now – it's as if my body craves it!'" – p. 68
McKinnon (1991)	"... [Stillness Meditation] is *easy*, *pleasant* ... wonderful." – p. 133
McKinnon (2002/2008)	"... a pleasant thing to do." – p. 35

Quoting adolescents who have tried Stillness Meditation: “I enjoyed it very much ...”, “I found it very enjoyable ...”, “I enjoyed [it] ...”, “I thoroughly enjoyed the stillness ...”, “I really enjoyed it ...” – p. 86

1.5 Regression

Regression is a term that was used by Meares to describe the mental process that he theorized occurred in Stillness Meditation. Meares contended that the practice involved what he termed *atavistic regression*, where the mind reverts to a more simple and primitive manner of operating that he argued was characteristic in early stages of human evolutionary development (Meares, 1967/1968, pp. 61-62, 87, 1969c, pp. 81-83, 1978/1986, pp. 141-144, 1988, p. 73). Meares distinguished atavistic regression from *age regression*, meaning regression towards childhood. He argued that atavistic regression is a natural mental function that occurs in various circumstances of everyday life, including, most notably, periods of daydream or reverie. His claim was that the regression in Stillness Meditation is the same as in daydream or reverie, except that in the meditation it is more complete, and is achieved by intentional practice, rather than arising spontaneously. Meares regarded atavistic regression as providing the essential theoretical underpinning for Stillness Meditation. For the purposes of this table, and unless the context requires otherwise, regression can be conceptualized simply as the mental relaxation that is said to take place in the meditation (Meares, 1969c, p. 83).

1.6 Major presentations of the practice

This section is to be read with together with the other sections in Part 1 of this table. See in particular 1.8 (“Effortlessness”), and 1.10 (“Slight/minor discomfort”).

1.6.1 Meares (1967/1968)

“A basic principle of [Stillness Meditation] is the use of physical relaxation as a key to mental relaxation. This takes place in two stages. First we must learn complete physical relaxation, and second we must learn how to use this physical relaxation to promote calm and ease of our mind.” – p. 58

Physical relaxation...

“We bring our body into relaxation by allowing the tension to go from our muscles.” – p. 78
“... [We allow] our muscles to let go.” – p. 78
“... [W]e allow our eyelids to close.” – p. 90
“As we do this we keep ourselves aware of the relaxation.” – p. 78
“[T]his conscious awareness of the relaxed and easy feeling is a very important part of all our exercises.” – p. 78
Meares describes exercises involving progressive relaxation of the muscles (p. 79).
“We present ... ideas to our mind” such as (p. 79):

- “We allow [the muscles of our legs] to let go.”
- “We really feel it.”
- “Our whole body is relaxed.”
- “We feel this easy comfortable relaxation come all through us.”
- “... [O]ur mind learns to be calm and at ease again.”

“Once we [learn this process, it] is really quite effortless.” – p. 94
“[If] we make an effort ... the whole thing goes wrong, and we are immediately aware that we are not relaxed.” – p. 95

Using physical relaxation to promote calm and ease of mind...

Relaxation of the mind...
Meares describes a continuation of the exercises (p. 81).
“... [T]he sequence is logical and straightforward. We feel the relaxation of our muscles. Our relaxed muscles are calm. We can feel the sensation of calm in them, we feel the calm of it all through us. We feel the calm of it in our mind.” – p. 82

Regression...
“When we have attained relaxation of our mind, we have already started on the way to regression.” – p. 82
Meares describes a continuation of the exercises: “We let ourselves go. – We let go, and we drift. – We drift in the calm of it. – Just letting ourselves go, we drift more and more.” – p. 83
“We let ourselves go in the calm. ... [W]e feel ourselves drifting in the calm ...” – p. 83
“When we achieve this drifting sensation, we have ... regressed.” – p. 83
“[In regression] we let our mind wander, and are no longer concerned with our immediate surroundings.” – pp. 82-83
“... [W]e leave our mind to wander uncontrolled as in a state of reverie.” – p. 93

Meares pp. 160-161 summarizes the process to this point: “We let our bodies relax; we feel the calm that goes with it; we feel it all through us, in our body, in our mind; we let ourselves go; we let go and we drift; we drift in the calm of it ...”.

Trains/sequences of thought...
Once regression has been mastered, trains/sequences of thought can be presented to the mind for relief of specific symptoms (p. 92).
“... [We present] to our mind very simple ideas for improvement while still in the relaxed and regressed state.”– p. 92
Meares pp. 96-114 provides suggested trains of thought for particular symptoms.

1.6.2 Meares (1978/1986)

Meares (1978/1986, p. vii) states: “This work does not replace [*Relief Without Drugs*, Meares (1967/1968)], nor is it an attempt to bring it up to date. In its simplicity, that book remains the one for the beginner ... whose prime aim is the relief of bodily symptoms. The present book clarifies and extends the description [of Stillness Meditation] so that the reader should have little difficulty in attaining the more complete experience which lies beyond relaxation ...”

“There are three important steps in learning [Stillness Meditation].” – p. x
- “... [C]omplete, physical relaxation ...”;
- “... [E]xperience the relaxation of our body in our whole being so that our mind fully participates in it.”; and
- “... [E]xperience ... this relaxation in the face of minor discomfort.”

Physical relaxation...

The initial step in Stillness Meditation is to “let our mind [experience] the relaxation of our body” (p. 6).
“[Physical relaxation] is not a part of the meditative experience.” – p. 7
“[It] is merely a prelude to relaxing our mind ... the door by which we enter, something through which we must pass.” – p. 7
Meares describes the progressive relaxation of the muscles (pp. 7-9).
“The contracting and relaxing of our muscles is far too mechanical to help in real meditative experience.” – p. 9
It keeps the mind alert/active, and functioning at a rational/practical level, which impedes further progress (p. 9).
So once the mind has been educated in the feeling of relaxation, the contraction and relaxation of the muscles is abandoned (p. 9).
“We must make a transition ... to a global experience of physical relaxation.” – p. 9
“The global relaxation of all our muscles is a total experience. All our muscles. All through us. The totality of it all. We experience it as utter and complete. As we let it come we may experience ourselves as sinking into it, sinking into the relaxation, deeper and deeper, more and more completely. Enveloped by it, as a mist silently envelops us on a mountain. It is calm, it is cool, it is fresh and it is silent. We are relaxed completely. More than feeling it. Just being it.” – pp. 9-10
“Our eyes close as the physical relaxation comes to us.” – p. 12
“At the start, our awareness of our physical relaxation is very important, but as we progress ... the need for this awareness passes” – p. 13 “... [That] awareness ... can detract from the higher aspects of the complete experience.” – p. 14

Experiencing the physical relaxation in our whole being...

Experiencing the physical relaxation...
“Our awareness of relaxation and the feeling of it are both necessary steps in experiencing relaxation, but once this is achieved [they] should cease.” – p. 19. “[Otherwise] the greater experience of stillness will elude us.” – p. 19
Meares is referring here to intellectual awareness (e.g., the meditator’s awareness that their leg/body is relaxed; pp. 17-18).
“When we are aware of our physical relaxation, our mind has to stand apart ... in order to attain the awareness of something else. But when we experience the relaxation there is no standing apart, and our mind itself participates ...” – p. 21
“More than our body relaxes. Now it is our whole self that is relaxed, our ego, our total being ... [W]e, ourselves, are participating in it ... It is no longer a matter of our muscles or our body being relaxed because the distinction between our body and ourself has faded. It is all one. The relaxation becomes a total experience which involves our whole being.” – pp. 19-20

Relaxation of the mind...
“The experience of our physical relaxation brings with it the relaxation of our mind.” – p. 21
“Our mind participates with our body in total relaxation. This comes about through the *experiencing*; not through knowing that we are relaxed, feeling relaxed or being aware of it. Being it.” – p. 23
“The secret to the relaxing of our mind is the total experience of the relaxation of our body ... It is not just the experience of our whole body being relaxed. More than our body it is our whole self.” – p. 24
“When the relaxation includes every aspect of our being, then our mind itself participates in it ... When everything is relaxed, our mind is relaxed too.” – p. 24 “As we experience the relaxation of our body, we let the experience spread out, as it were, until it invades our total being – invades the whole of us. It now encompasses far more than our body. It comes as quietness, and an ease, pervading

everything – our thoughts, our feelings, our whole being. It all follows quite easily and naturally from experiencing the relaxation of our body. We just allow it to become more complete, totally complete ... [O]ur mind is relaxed like the rest of ourself." – pp. 24-25
"... [We let] ourself participate with complete freedom in the experience of relaxation." – p. 25
"... [W]e let ourselves relax in our whole being, and it is in our mind." – p. 25
"... [W]e can intensify the process by letting ourselves experience the natural calm within us." – p. 26. "... [W]e let ourselves feel the calm within us ... Our letting go intensifies the process, and the calm within us becomes a reality." – p. 27

Beyond relaxation of the mind...
"We experience the relaxation of our body, and we experience the relaxation of our mind, but it is more, it is our whole being. As we let ourself go with it the boundaries of body and mind disintegrate. We let go still more completely and a greater experience of unity comes to us. It is our being, our whole being of which our body and our mind are merely different facets. We come to experience the relaxation of our body and of our mind as the calm and ease of our whole being." – p. 27
"Body relaxed; mind relaxed; more than our body and our mind; it is our whole being. We experience the completeness of the relaxation. It is in every aspect of ourself." – p. 31
"... [We] let ourselves be. Of all human activity, of both body and mind, this is the simplest and the most primitive – the act of just being in all its simplicity and naturalness with nothing added at all; not even being alive, as this involves our awareness. It is the unadorned, primordial experience of existence. This is *simple being.*" – p. 27

"Once we have learned to relax our mind [by experiencing the relaxation of the body] we no longer need to concern ourselves about experiencing the relaxation of our body ... Once our mind has learned how to do it, there is no need for us to repeat these preliminary steps each time. We now know what we want, and our mind has learned how to attain it. When we want to relax our mind, the relaxation of it is just there, quite naturally ..." – p. 31

Experiencing the relaxation in the face of minor discomfort...

See 1.10 ("Slight/minor discomfort").

1.6.3 Meares (1969c)

Streamlined presentation. Two steps: – p. 84
- Relaxation of the body – progressive relaxation of the muscles; and
- Relaxation of the mind.

The instructions for the relaxation of the mind are short and simple: "We now come to experience the relaxation of our body in our mind. Our body is relaxed. We are relaxed. It is all through us. Feel this. Experience it. This is the secret of it ... Remember you do not have to make yourself relax. It is there. You just let it come. The whole process is quite effortless ... We experience the feeling of the relaxation of our body; we experience it in our mind." – p. 84

Refers to Meares (1967/1968) as providing much greater detail (p. 88).

1.6.4 Meares (1973b)

Meares' instructions for Stillness Meditation are provided as part of guidance for students on how to expand their minds without drugs. It appears that, for this reason, Meares includes a step, "let the mind run free", that is not found as a discrete step in his other texts. This seems to reflect a different approach to dividing up the overall instructions into steps, rather than a substantive difference in the instructions/practice. In other words, leaving aside Meares' (1973b) novel partitioning of the steps, his descriptions of Stillness Meditation are basically in line with the guidance that he provides in his other publications.

Expanding the mind using Stillness Meditation involves four steps (pp. 251-255):
- Let the muscles relax – progressive relaxation of the muscles;
- Experience the relaxation of the body in the mind;
- Let the mind transcend minor discomfort; and
- Let the mind run free.

Experience the relaxation of the body in the mind...
"More than just feeling it – being it." – p. 252
"There is an effortlessness about it all." – p. 252

Let the mind run free...
"When you have mastered [the] three initial steps, you just allow your mind to run free. You let go; your mind takes on activity of its own [in the form of ideas and sensations] ... With a little experience it is usual for the ideas and sensations to become progressively fewer." – p. 254

Refers to Meares (1967/1968) as providing more detail (p. 103).

1.6.5 Meares (1989)

"The first thing we must learn is the physical relaxation of our body." – p. 109
"The next step is to *experience* the physical relaxation of your body." – p. 111
"[Then] we come to the meditation itself." – p. 113

Physical relaxation...
"We learn the physical relaxation ... by the progressive relaxation of our muscles." – p. 110
"The physical relaxation ... has very little to do with meditation, which is a process of the mind. But physical relaxation ... is a necessary step in learning the mental state of meditation. Please don't skip the physical relaxation and think you will go straight on to the mental process. It does not work! Remember there are always preliminaries to each new skill we acquire ... When we can meditate, we do not think of it as much connected with physical relaxation." – pp. 109-110
"You will soon come to know that physical relaxation is not a part of meditation. But your mind must learn how to relax your body physically before it can master the real thing." – p. 111

Experiencing the physical relaxation...
"Don't dismiss this as unimportant. Like physical relaxation, it has no part in the fullness of meditation. But it is a necessary step in getting there." – p. 111
"What we want is just a simple, relaxed, natural, uncritical awareness of our physical relaxation. The feeling of it." – p. 112. "As we become aware of the relaxation of our body, our mind comes to participate in it." – p. 111

The meditation itself...
"So you are going to practise the physical relaxation ... and let yourself experience this relaxation in your mind. Then gradually, this other experience, the stillness, naturalness and effortlessness ... will come to you. And you are into meditation." – p. 114

1.6.6 Meares (1987/1991)

Presents Stillness Meditation as "5MX" – five mental exercises (pp. 121-128).
Only the first, second, third and fifth are relevant for the present table:
- Let the muscles relax – progressive relaxation of the muscles;
- Experience the relaxation in the mind;
- Let the mind transcend minor discomfort; and
- Practise 5 minutes daily.

The fourth exercise (experience the ease of mind in daily living) is not relevant for this table because it concerns the experience following, rather than during, a meditation session.

Experience the relaxation in the mind...
"More than just feeling it. Being it." – p. 123
"There is an effortlessness about it all." – p. 124

Refers to Meares (1978/1986) for more detail (p. 113).

1.6.7 Meares (1970)

Refers to Meares (1967/1968) as providing the detail (p. 66), but summarizes the approach as follows: "Practice letting your mind experience the natural ease of things ... Sit quietly. Just let yourself be. You don't have to make yourself relax. It is all quite effortless ... You just let yourself be, and the relaxation is there because it is natural to you." – p. 67

1.6.8 Meares (1976b)

"There are three simple stages in [Stillness Meditation]" – p. 909
"The first is the complete physical relaxation of the body." – p. 909
"The next step is to learn to experience the complete physical relaxation of the body." – p. 909

"The third step ... is to meditate in a situation of slight discomfort." – p. 909

Physical relaxation...
"This is a total experience and is not achieved by progressive relaxation of muscle groups, which has the effect of keeping the mind alert and so preventing the atavistic regression which is an essential element of any deep meditative experience." – p. 909

Experience the physical relaxation...
"As we come to experience our relaxation it is not so much that our body is relaxed, but rather that we are relaxed. Our whole self. More than our body and our mind. Our whole being. In this state our mind participates in [the experience] ... Stillness comes. Not all at once ..." – p. 909

1.6.9 Elaborations in other Meares sources

Meares (1976/1984) "... Relax the body.
All the members of the body, arms, legs,
And all the parts within.
But it is not of the body.
Nothing to do with the body at all.
The prelude.
Without the prelude we cannot proceed.
How necessary!" – p. 11

1.6.10 McKinnon (1983/2016)

Referring to her own experience of learning Stillness Meditation from Meares: "... [I]t was necessary during my early sessions to *consciously* let go of physical tension. Dr Meares intimated that the release of physical tension must come first; ease within the body would lead to ease within the mind. This too, was to be simple in its execution. There was none of the usual progressive muscle relaxation – just the simple acceptance of one's natural human ability to relax ... to let go!" – p. 191

"... [F]irst the body deeply relaxes, becoming heavy and still. Physical ease *always precedes* mental ease." – p. 211

"[Descriptions of Stillness Meditation are] very subjective ... I will take you through three varying descriptions ..." – p. 218

Approach 1...

Four steps (pp. 219-221)...
- Adopt the correct physical posture;
- Let go physically;
- Trains of thought; and
- Stillness.

Posture...
See 1.11 ("Posture").

Let go physically...
"... [R]ecognise tension throughout your body. For the first few practices, begin from the feet and work up through the body, sensing the tension and letting it release ... After you have practised regularly for a while, you will realise that any need to physically let go of tension will be unnecessary; you will sit down to practise and physical ease will just occur." – pp. 219-220

Trains of thought...
"The mind takes longer to become *easy* than does the body. Newcomers ... often find it helpful to have a few words or phrases with which to get started. These are not rigid thought patterns on which to concentrate, but simply ideas or trains of thought that encourage people to give in and experience the effortlessness ... These words or phrases ... should continue only for a few minutes, since the whole idea of [Stillness Meditation] is never to concentrate, visualise or focus our attention or awareness upon anything ... [U]se [the words or phrases] if you need to, just to commence and until the mind eventually loses interest in pursuing them." – p. 220 McKinnon gives examples of these trains of thought, including "effortlessness", "letting go", and "more and more" (p. 221).

Stillness...
Emerges naturally – see 1.14 ("Progression to the goal-state/s") and Part 2.

"The four steps ... are really one effortless experience. The process of [Stillness Meditation] is not a series of steps to be mastered ... Each factor is important, but the stillness experience is a 'global' experience and is not divisible in this way." – p. 221

Approach 2...

Five steps (p. 222)...
- Desire;
- Commitment;
- Environment;
- Posture; and
- Practice.

The desire, commitment and environment steps are not important for the purposes of this table.

Posture...
See 1.11 ("Posture").

Practice...
"... [W]e let our *practice* take place ... We are not going to *make* ourself relax in any way." – p. 224
"As beginners ... it may be helpful to observe tension anywhere throughout your being, and *gently let it go*." – p. 224
For example: "... [W]e ... allow a smoothing out of the muscles of our face ... and we sense the ease in our face, radiating throughout our head and flowing down ... to the very centre of our being ..." – p. 225

Approach 3...

In form of a preamble/monologue, as might be presented at the start of a Stillness Meditation class (pp. 225-228).

The central instruction is to let go or just let.
Specific instructions include:
- Observe parts of the body where there is tension/gripping, let go and allow it to ease away.
- Let yourself go beyond thoughts and distractions.
- Let your awareness of the teacher's voice go.
- Experience the stillness, and let go into it more and more.
- Just let yourself simply be.

1.6.11 McKinnon (2011)

"When [Meares] published *Relief Without Drugs* [1967/1968] ... the 'relaxing mental exercises' were set down in such a way that some element of Progressive Muscle Relaxation (PMR) took place. Ten years later, that structure was largely abandoned. His later work, *The Wealth Within* [1978/1986], guides the reader further ..." – p. 43

McKinnon (2011) describes the classes she attended with Meares (which commenced in 1974; McKinnon, 1991, p. 72): "... [Meares] placed no importance on deliberately relaxing the muscles of the body. One entered the room, adopted the correct sitting posture ... and allowed the eyes to close with the one intention of 'letting go and simply being there'. After some minutes of his rather mumbled, murmured and extremely non-directive preamble, the remainder of the session was comprised of ... an experience of deep and ever-deepening stillness." – p. 44 "But Meares ... was changing, as one does with life experience. One day, I found myself startled to note that he used no verbal preamble, no monologue whatsoever. And definitely no simulation of PMR or any input at all that would detract from the utter simplicity of the ideal stillness experience. His teaching of an 'absence of disturbance' was now complete." – p. 44

"In [Stillness Meditation] one learns to completely let go physically, to completely let go mentally – and then, one step more, to completely let go *beyond* any discomfort or disturbance ... And that's when, little by little, one will gradually experience stillness of mind ..." – p. 67

McKinnon p. 74 refers to the Meares (1976b) presentation of the practice as involving:
- "Complete physical relaxation";
- "Experiencing the relaxation so that it pervades our whole being"; and
- "Practising in circumstances of slight discomfort".

McKinnon says, "These principles set the scene for your meditation as a beginner and then long into the future" (p. 74).

McKinnon pp. 78-81 presents the practice in the form of a detailed preamble/monologue as might be provided in a Stillness Meditation class. The central instruction is to let go or just let. Specific instructions include:

- "You may feel tension ... in parts of your body ... [J]ust *let go* a little more ... Even more than letting go, you simply *let* ..." – p. 78
- "Just simply *allow* your eyes to close, without trying in any way." – p. 78
- "*You don't have to do anything* ... Just let yourself ..." – p. 78
- "Without trying to do so ... just let all tension become ease ..." – p. 79
- "And now gradually this becomes *more than feeling* ... Rest within your body is the pathway to rest within your mind." – p. 79
- "And now you might begin to *experience* that restfulness even more." – p. 80
- "... [A]llow a smoothing out of the muscles of your face ..." – p. 80
- "There is no effort to be made at all. No technique. No need to try or do anything. There is only the experience of letting yourself be ... more and more into complete *stillness*." – p. 80
- "And you experience only *stillness* at this time and you *experience it more and more and more* ..." – p. 80
- "And gradually this stillness, this calm of your body is also *within your mind* ... A complete stillness of yourself." – p. 81
- "Now your whole being is ... gradually becoming a unified being of stillness ... [Y]ou *experience* it deeply ... more and more and more completely ... [S]imply being still ..." – p. 81
- "*Just letting ... just being ... just effortless ... stillness* ..." – p. 81

"The experience follows a series of undefined, merging stages – a seamless transition to the experience of being still." – p. 84

1.6.12 McKinnon (1991)

On the trains of thought as described by Meares in *Relief Without Drugs* (Meares 1967/1968)...
"In ... *Relief Without Drugs*, Dr Meares outlines some dialogue or visualisation either as a means of achieving the initial relaxation required or as an aid to those people attempting to overcome some particular problem. While this can be of great assistance in particular circumstances and when correctly followed, for the simple reduction and relief of anxiety Dr Meares subsequently moved away from the use of the mind in any way at all while meditating." – p. 74

In describing the practice, McKinnon starts by emphasizing that: "... [Stillness Meditation is an] exercise ... of *not trying*. It is a totally effortless experience ... The paradox of [Stillness Meditation] is that the less effort we make, the more we achieve." – p. 76

McKinnon presents Stillness Meditation in four steps (pp. 76-78):

- Posture of minor discomfort;
- Physical relaxation;
- Letting the mind run freely; and
- Capturing stillness.

Posture of minor discomfort...
See 1.10 ("Slight/minor discomfort").

Physical relaxation...
"Letting our eyes fall closed, we sense any tension throughout the body and feel it fade away, letting it go ... Don't try to relax, just let the tension go, wherever you feel it and whenever it returns. Eventually all areas will relax." – pp. 76-77

Letting the mind run freely...
"The mind will be busy in the first attempts at practice ... [L]et the mind run freely until, of its own accord, and in due course, it relearns the ability to relax and be still ... A few gentle reminder words can be useful to beginners to commence with and words such as 'letting go', 'drifting', 'going with it', 'easy' and so on are suitable. In time the mind too, will relax quite readily." – p. 77

Capturing stillness...
"At first stillness of mind will be impeded by obstacles such as too many thoughts, itches and aches, perhaps anxiety and other distractions. If we ignore these, gradually, as we sit there, ideas will fade, thoughts lose their logical content and awareness of our surroundings becomes unimportant. Noises and distractions are transcended and the whole self is very still ..." – p. 77

In a later section, McKinnon says, "Attention should be given to the 'letting go' of tightness (tension) in the legs, arms, trunk (or tummy), shoulders and especially the face: 'Eyes gently closing, face smoothing out, giving in all over, throughout our whole self, easy and effortless, no need to do anything, no need to think of anything, just simply be'. And then nothing more. *Only complete quiet* ..." – pp. 126-127 McKinnon p. 129 says, "With young children, a few minutes spent in demonstrating the difference in feeling between a clenched hand and a relaxed hand, or a clenched jaw and a relaxed jaw, is useful."

1.6.13 McKinnon (2002/2008)

McKinnon (2002/2008) describes how Stillness Meditation can be taught in schools.

A preliminary/preparatory exercise...
"Explain to the [primary school] children that to experience stillness it is not necessary to do anything. They are simply going to be still. [As a preliminary/preparatory exercise] [e]xplain the correct, upright posture and invite the children to sit like that, to let their muscles go ... Take the children through muscle clenching." – p. 50 McKinnon pp. 50-51 describes a progressive relaxation exercise.
"Remember that during the experience of stillness, a focus on progressive muscle relaxation is not desirable. However, in these early stages of learning, a minute or so spent [on clenching and relaxing] is an important aid in demonstrating the different feeling between tension and ease ... [W]e will [then] let attention to these details go." – p. 52
Along the same lines, McKinnon later provides guidance on teaching adolescents: "Invite the class to spend a few minutes identifying tension within their body." – p. 65 She describes the progressive relaxation exercise (p. 65). "The meditation teacher must remember that Stillness Meditation is not related to the relaxation technique known as Progressive Muscle Relaxation. The time spent in identifying tension is simply a way of assisting students to recognise how their body is reacting." – p. 65

The meditation itself...
"Seated in correct posture, the students do nothing but allow themselves to give in more and more to complete relaxation ... The stillness will come as the student learns to simply sit in effortless repose." – p. 45
"In Stillness Meditation there is nothing at all to do. The aim is simply to 'be' ..." – p. 52
"... [P]hysical ease is the pathway to mental ease ..." – p. 65

1.7 Classes

Individuals typically learn Stillness Meditation by attending meditation sessions led or supported by a meditation teacher, or by practising alone (at home or in some other environment). Meditators who learn by practising alone can base their practice purely on Stillness Meditation texts, or during a meditation session they can listen to one of the audio-recordings that have been produced by Stillness Meditation teachers. If a teacher is present or an audio-recording is played, the teacher could be said to directly facilitate the Stillness Meditation experience. In this table, the term "classes" is used to refer to Stillness Meditation sessions where a teacher is present, and leads or supports the meditators. Importantly, the term should not be read as suggesting any kind of logical step-by-step instruction in the sessions, as that is regarded as contrary to the Stillness Meditation experience. Due to the potential for that suggestion, McKinnon tends not to use the term classes (P. McKinnon, personal communication, August 19, 2018). The term is relied on in this table purely for ease of reference, and bearing in mind the similar issues or ambiguities that can arise with alternative descriptors such as "teacher-led sessions" or "facilitated sessions".

To properly understand Stillness Meditation, it is helpful to have some appreciation of what happens in classes. The most detailed description of Stillness Meditation classes is provided by Meares (1989, pp. 8-75), which concerns the teaching of the practice to school students. As a starting point, Meares emphasizes that the Stillness Meditation teacher should themselves have genuine ease of mind, supported by their own Stillness Meditation practice (pp. 10-17, 18, 21). He then discusses two critical elements of Stillness Meditation teaching: the use of non-logical communication (pp. 18-34), and the development of rapport (pp. 39-52). Non-logical communication is utilized in order to avoid provoking logical/critical/intellectual activity on the part of the meditator (p. 18). Meares elaborates: "We fail ... if we say to the meditating student such things as, 'Let yourself relax. Let the stillness come to your mind.' In speaking like this we automatically keep the student's mind functioning critically ... He or she keeps evaluating what we have said. And [his/her] progress towards stillness of mind is effectively halted." (p. 19). In other texts, Meares notes that in the classes all logical communication is to be avoided (e.g., Meares, 1971a, p. 676, 1973a, p. 734). The practice can be discussed in a logical fashion on a separate occasion prior to the class, or it can be discussed after a class has finished (e.g., Meares, 1989, pp. 44-52), but during a class, and in the immediate lead-up, there is no logical discussion (Meares, 1971a, p. 676).

Meares (1989) discusses the following forms of non-logical communication used by the Stillness Meditation teacher: (a) "non-logical use of words and phrases" (p. 20), for example, "Good", "Easy", and "That's right" (p. 21); (b) "varying [the] degree of physical closeness with [the meditator]" (p. 22); (c) what Meares refers to as "non-verbal phonation" (p. 23) – "Umms" and "Ahhs" (p. 23) made in a "long, slow, natural exhalation of ... breath" (p. 25); (d) touch (pp. 26-31); and (e) increasing periods of tranquil silence (pp. 32-33). The non-logical words/phrases and the non-verbal phonation are used in the preamble/monologue that the teacher speaks at the start of the meditation session (pp. 55-56), and, more briefly, at the end of the session, when the teacher brings the meditators out of the meditation (pp. 59-60, 68). Meares notes that "[t]ouch is by far the most important" of the forms of non-logical communication (p. 26), since it most powerfully communicates to the meditator the teacher's own ease of mind (pp. 28-29). Consistent with this, Meares (1983a, p. 116) comments, "[M]y own genuine ease of mind is the most important single factor in preparing the patient for meditation". Meares (1979a) provides his most detailed description of the use of touch, however it concerns patients using intensive meditation for treatment of cancer, and touch was likely more extensive in that context. McKinnon (1983/2016) explains that, in the

more general context, Meares used touch "especially on the shoulders, chest and forehead of the meditator" (p. 210), an approach that she maintains in her own teaching. She says, "Touch is the difference within [Stillness Meditation] that seems most able to engender profound healing" (McKinnon, 2011, p. 95). Meares (1989) clearly conveys that the touch is to extend very naturally from the ease of the teacher, rather than being a technical or mechanized procedure (see, e.g., p. 31). McKinnon (2011, p. 96) echoes this, in saying that there is "no particular therapeutic touch but a sensitive interaction with another human being to communicate reassurance and security ...". Elsewhere McKinnon describes the touch as calming, reassuring and therapeutic (McKinnon, 2002/2008, p. 34, 2011, p. 18).

Meares (1989) refers to rapport as a "decisive factor" (p. 39), and discusses at length what it means and how to develop it (pp. 39-52). While the detail of that discussion is not important for the present table, it is helpful to note the following passage, which communicates the key underlying idea: "We must always remember that [Stillness Meditation] involves a dropping of both our physical and psychological defences. Students can come to do this more easily and more completely if they are in the presence of someone whom they trust, who will watch over them, guard them, both physically and psychologically." (p. 24).

Meares held small private group meditation sessions, for paying patients, accommodating about 15 people (McKinnon, 1991, p. 72, 2011, p. 201, 1983/2016, pp. 187-188; Meares, 1979d, p. 121, 1983a, p. 117). For a long period he also conducted large group sessions, usually attended by 50 to 60 people, which were open to the general public, and conducted on a pro bono basis (McKinnon, 1983/2016, pp. 187-188, 2011, p. 201; Meares, 1973a). The small private group sessions were preceded by a separate one-on-one consultation with each patient where Meares undertook a physical examination, and provided a brief demonstration and experience of Stillness Meditation (Meares, 1967b, p. 45, 1971a, p. 675). For the small group meditation sessions, Meares would lead each meditator into the meditation room and, when they were all seated, he would begin with a non-logical preamble as referred to above (McKinnon, 2011, p. 199; Meares, 1971a, 1983a). To ensure that the privacy of each meditator was maintained, there was no interaction between meditators, and chairs in the meditation room faced in the same direction (McKinnon, 2011, p. 91; Meares, 1983a, p. 117). Meares would gradually transition from the preamble to a tranquil silence that would last for the remainder of the session, a period of about 40 minutes (McKinnon, 2011, p. 44; Meares, 1971a, p. 676). He would then bring the patients out of the meditative state using the non-logical words/phrases and non-verbal phonation (Meares, 1989, pp. 59-60, 68), and by "telling [them] to open their eyes and rest quietly" (Meares, 1971a, p. 676). Patients would be led from the room one-by-one, and would have a few minutes conversation with Meares before departing (McKinnon, 2011, p. 199; Meares, 1971a, p. 676). For the public sessions, there was typically no separate consultation with Meares before meeting him for the meditation (Meares, 1973a, pp. 733-734). The sessions were essentially the same as the private ones (McKinnon, 1991, p. 72; Meares, 1973a, p. 734), however less attention could be given to each individual owing to the much larger number of attendees (McKinnon, 1991, p. 72).

McKinnon (2011) states that her classes "replicate and imitate [Meares'] procedures" (p. 91). She clearly communicates her determination to preserve the integrity of Meares' approach, rather than modifying it in any substantial way (McKinnon, 2002/2008, p. 32, 2011). McKinnon's classes differ to Meares' approach only in relatively minor respects. As her teaching is not conducted in a medical context, there is no physical examination. Her clients also enter the meditation room by themselves, rather than being led by the teacher. McKinnon (1991, p. 127) describes her preambles as non-logical, but that appears to reflect a slightly different assessment of what constitutes logical content to that of Meares. As noted above, Meares considered that simple sentences like "Let yourself relax", and "Let the stillness come to your mind" contain logical content. McKinnon's preambles include content the same as, or similar to, her meditation instructions set out in McKinnon (2011, pp. 78-81, 1983/2016, pp. 225-228), as summarized at 1.6 ("Major presentations of the practice") above. Much of that content is similar to the two sentences used by Meares as examples of logical content. Nonetheless, McKinnon clearly recognizes the importance of discouraging meditators from engaging in logical processing, and her preambles are targeted to that end. Whether logical or not, the content is clearly imbued with the idea of effortlessness. McKinnon's (2002/2008, p. 54) way of putting this is that the content is non-directive. She refers to the preamble as being about creating a calming and supportive atmosphere (McKinnon, 1991, p. 127, 2011, p. 107), as distinct from providing formal/logical instruction (McKinnon, 1983/2016, p. 209). In that vein she observes that "[l]istening, hearing and understanding information are not a necessary part of [Stillness Meditation]" (McKinnon, 2011, p. 107). McKinnon (2002/2008, 2011, pp. 89-98) provides detailed expositions of the Stillness Meditation teacher's role, and other aspects of Stillness Meditation classes.

The remainder of this section sets out a number of passages relating to Meares' classes and initial consultations. Most of them have been selected on the basis that they cast light on a particular issue regarding the practice: whether Meares incorporated in his classes or initial consultations progressive muscle relaxation and/or deliberate awareness or feeling of that relaxation as referred to in this table above. In addition to helping to address that issue, the passages give a picture of Meares' classes in his own words, providing an illustration of the various aspects of Stillness Meditation classes described above.

Meares (1989)	Meares notes that in the Yoga and Zen traditions it is common for the teacher to make no verbal communication (pp. 19-20). He then says, "Such a procedure is very disturbing to Westerners. We can do better than that." – p. 20<br>Meares gives an example of his preambles used in Stillness Meditation classes for children: "'Just let your arms rest on the desk in front of you.' Said slowly and naturally. 'Comfortable.' A little pause. 'Quite comfy.' Another little pause. 'Good.' Perhaps an exhalation that fills in the silence. 'That's good.' 'See

	your hands relaxed.' 'Feel it.' 'Feels good.' 'We can let our eyes close, and we feel the relaxation more completely.' 'That's good.' 'More.' 'More and more completely.'" – p. 55
Meares (1967b)	Describing his initial consultation with a patient prior to one of the small, private Stillness Meditation sessions: "I demonstrate [mental relaxation] to the patient by letting myself relax into [Stillness Meditation] for a few moments. He sees that there is nothing odd or difficult about it ... I then ask him to do the same thing. Now and then I help his physical relaxation by lifting his arms and letting them flop on the chair." – pp. 44-45
Meares (1971a)	"[The consultation] concludes with a brief physical examination and some introduction to [Stillness Meditation] while [the patient is] still lying on the examination couch." – p. 675<br><br>Describing a typical small, private Stillness Meditation session: "In the group room I avoid all logical communication, as this would only keep the patient alert ... I say very little. 'Good – easy – natural – it's all effortless. Utterly natural – just letting ourselves go.' These words and phrases are said very slowly with long pauses between each. But the main communication is by touch. I lean over the back of the chair and hold the patient's shoulders. I lift up one arm and let it flop back on the arm of the chair. Then I do the same with the other arm. 'All effortless – natural – we experience the ease of our body – it's all through us – natural – through our whole being.' I let my hands rest on the patient's forehead. 'Deeply – just letting ourselves go with it – letting go freely.' ... Nearly everyone is in quite [a] deep [state of meditation] in a few minutes. My words give way to unverbalized phonation, 'Ummm-ah' ... I make these unverbalized communications very leisurely, very slowly. The pauses between them are drawn out. Silences develop, and the patients drift still deeper into [the meditation]." – p. 676
Meares (1973a)	Describing a typical large, public Stillness Meditation session: "As the participants arrive ... I show them to their seat. This few seconds of individual contact with the participant is of the utmost importance. I say, 'We are just going to let ourselves relax very completely'. As they sit down I make a point of arranging their arms so that they rest on their thighs. The main purpose of this manoeuvre is a communication of ease through the medium of touch. I have found that these few seconds give adequate rapport for the participant to be sufficiently secure to be able to abandon himself offguard in the regression of meditative experience ... As I take one person to his seat, I communicate with others by quietly touching them on the shoulder as I move past. There is no introductory statement as to what I want them to do ... The idea of what is expected of the participant is communicated paralogically, by my behaviour, by my touching, by my unverbalized phonations, and by my own relaxation. Most of the people who come in first are well into [the] ... meditative state before the later arrivals have taken their seats ... In a leisurely manner I say ... 'Good', 'Easy', 'Effortless', 'Letting ourselves go', 'Ease of our body', 'Ease of our mind'. As the regression of the participants becomes more complete, I say less and less. There are pauses, and pauses continue into silence." – p. 734
Meares (1979a)	"All [cancer] patients are first seen in a psychiatric interview. This is followed by a leisurely physical examination ... [T]he main purpose of [the] procedures [comprising the examination] is to accustom the patient to relaxing as he is being touched, so we can gain the rapport essential for further tactile communication." – p. 122 "In subsequent sessions the ... patients are seen in a small group of ten to fifteen ... With the patient seated in the group initial touch is made on the shoulders ... The patient's arms can be raised and allowed to fall back on the side of the chair; the patient rightly interprets such moves as aids to his physical relaxation but, more importantly, they are stepping stones towards a greater experience." – p. 122 Meares goes on to describe other aspects of the therapeutic touch (pp. 122-123). "The communication by touch ... is accompanied by reassuring phrases which do not evoke any critical activity in the patient. 'Good', 'Letting ourself go with it', 'The natural ease within us', 'The simple naturalness of it all'. These give way to unverbalized phonation made in long easy exhalations – 'Umm', 'Ahh' ..." – p. 123
Meares (1983a)	"Any logical communication that I might make alerts the patient's critical faculties to evaluate what I have said, and so defeats the purpose of the procedure, which is to lead the patient to stillness of mind. Even such simple, but logical ideas, as, 'Your arms are relaxed', have this effect, as the patient immediately thinks about the relaxation of his arms'." – p. 117

1.8 Effortlessness

Meares (1967/1968)	On the exercise involving the progressive relaxation of the muscles: "... I described how the idea of relaxation is presented to the mind, and how we then experience the relaxation – both the feeling and the act. Once we can do it, this process is really quite effortless." – p. 94
Meares (1978/1986)	Effortless – pp. x, 24<br>"There is no making ourselves relax. We cannot relax by act of will. The procedure is completely free from effort and striving ... [T]here must be no attempt at trying to make ourselves do it; no making ourselves relax ... [W]e abandon all trying, striving and attempts to use our will ..." – p. 11<br>"Our eyes close as the physical relaxation comes to us ... When the muscles of our eyelids are relaxed, our eyes close. Quite effortlessly." – p. 12<br>"... [I]n [Stillness Meditation] there is utter effortlessness. No effort. No trying. No striving. No making ourself do it. No effort at all. No striving for effortlessness. No making ourself go with it. It is just there. Going with it in utter effortlessness." – p. 23<br>"Real effortlessness is essential for [Stillness Meditation]." – p. 23<br>"The effortlessness ... leads us into stillness of our mind." – p. 23<br>"We learn to meditate without effort." – p. 23<br>"[We let go] [e]ffortlessly." – p. 25<br>Meares pp. 46-47 gives the example of a "very tense young man" he saw who was doing 3 to 4 hours practice a day, but in the wrong way. "I ... discovered that he had become preoccupied about the subjective experience of [Stillness Meditation]. He kept striving for a greater and greater feeling of it ... He had lost the ease and effortlessness of it ..."<br>"I have sometimes had conversations like this with people who have come to seek my help.<br>'You want me to try to relax.'<br>'No. No trying. I don't want you to do anything.'<br>'You want me to go to sleep.'<br>'No. I don't want you to go to sleep. I don't want you to do anything.'" – p. 153
Meares (1969c)	"When you have had a little experience of [Stillness Meditation], you will notice that ... the whole process has been quite effortless." – p. 86
Meares (1973b)	"Let your eyes close. I say, 'Let your eyes close.' You don't have to close them – it is all effortless. As you relax, they just close." – p. 251<br>"[In experiencing the relaxation of the body in the mind] [t]here is an effortlessness about it all. Trying to relax, or trying to experience it, is not a part of it at all; and trying puts a stop to this effortless experience which comes quite easily and naturally when we just let go." – p. 252<br>With reference to Stillness Meditation generally: "I must emphasize the effortlessness. This is really the key to it all. Not only effortlessness of the body, and the mind too, but a total effortlessness of being." – p. 255
Meares (1989)	"No trying to meditate. No trying to control our thoughts. As we do it, the meditation comes to us without our striving for it." – p. 116<br>"The whole experience of this type of meditation is something that comes to us. It is not something that we bring about by an act of will. It comes to us, but before it can come we have to arrange the right conditions. We use our will to sit down and practise physical relaxation ... We have to use our will to sit down and prepare to meditate, but here the use of our will ceases, and we just let the meditation come." – p. 116<br>"We don't strive, we just let go of ourselves. More than this, we just let ourselves ... Perhaps there is an analogy in going to sleep. We prepare ourselves for sleep. We go to bed. We don't strive to go to sleep. We let ourselves go to sleep. In other words we have set up the right circumstances, and we let our mind work in this other way which we call sleep. In meditation we set up the circumstances, and we let our mind work in this other way we call meditation. In neither is there any striving. We let go. We take off the brakes. And our mind slips into this other way of working." – pp. 116-117
Meares (1970)	"Just let yourself be ... It is all quite effortless ... You just let yourself be, and the relaxation is there because it is natural to you." – p. 67 "The other point I wish to emphasize is the effortlessness of it all. There is no making yourself do it. It is just there, all natural, and utterly effortless." – p. 68
Meares (1977a)	Meares pp. 132-133 discusses how one of his patients experienced a relapse of cancer when Meares went overseas and she started meditating incorrectly: "It gradually became clear that she had changed ... her meditation ... In [a] burst of confidence, she departed from the extreme simplicity of [Stillness Meditation]

	... She would tell her cancer to get better. She would will it to get better. 'I will make you get better'. And ... she came upon the way of visualizing her cancer getting better ... However, when she returned to the extreme simplicity of [Stillness Meditation] her breast softened again, she put on weight, and strength returned." – p. 133
Meares (1978c)	Meares pp. 85-87 discusses the same patient referred to in Meares (1977a) above, and his comments are similar. He concludes, "My interpretation ... is that the patient's clear visualization of a selected subject demanded alertness and active control of her mind which of necessity prevented ... regression ..." – p. 87
Meares (1983a)	"The whole procedure is effortless. Any attempt on the part of the patient to try to induce the stillness destroys the whole process." – p. 115<br>"It is important that the patient capture the effortless quality of the experience. Any attempt to make oneself meditate or make the mind be still is self defeating. There can be no striving in any form." – p. 119
Meares (1977b)	"... And the stillness.<br>More than letting it be.<br>All without effort.<br>Just letting ..." – p. 18<br><br>"... We need the calm and the stillness<br>In the whole of our being ...<br><br>How can I get it<br>In the whole of my being?<br><br>No one can *get it*, of that I'm sure.<br>*It comes*.<br>It's just that it comes.<br><br>Well, how does it come?<br><br>It comes when we let it.<br>It's as simple as that ..." – p. 96<br><br>"... Tell me,<br>How can I make it simple?<br><br>You can't.<br>It is something you cannot do. ...<br><br>Well, what can I do?<br><br>You let it come ..." – p. 111
Meares (1979b)	"... If you think of meditation<br>As technique,<br>You simply do not know<br>What it's all about." – p. 55
Meares (1984)	"... No forcing ourself<br>No striving to do it<br>No contemplation of any image<br>No awareness of our breathing<br>No repetition of a mantra<br>No forcing ourself to maintain a posture ..." – p. 18<br><br>"... There is no doing,<br>No striving.<br>The patient is simply shown<br>How to let it come ..." – p. 41

	"... Striving<br>Keeps our mind alert<br>And so destroys<br>The effect of meditation ..." – p. 117
Meares (1976/1984)	"... Loose rein, and we are carried along.<br><br>Trust this that bears us away.<br>Guide it, and we falter.<br>And the moment is lost." – p. 21
Meares (1987a)	"... Just leave open the door,<br>And little by little ...<br>[the stillness/ease] [c]omes to our being." – p. 7<br><br>"... I shall open the door.<br>[Ease] will see it and come in ..." – p. 48
McKinnon (1983/2016)	"[Stillness Meditation] does not involve technique ..." – p. 190<br>McKinnon p. 289 makes a similar comment.<br>"Utter simplicity [is] all that [is] required, without effort." – p. 191<br>"[Stillness Meditation] is typified [by] an effortless experience, unencumbered by technique ..." – p. 207<br>"... [W]e do not *try* or *strive* or *do* anything." – pp. 210-211<br>On the progressive relaxation exercise described in McKinnon's (1983/2016) first of three descriptions of Stillness Meditation: "Please don't try or force, but simply sense the tension and let go ... [The] 'letting go' should be quite effortless, without deliberate trying or striving. Just let it happen naturally. If we try to *make* ourselves relax, the effort ... is counterproductive to what we seek." – p. 220<br>"We are not going to *make* ourself relax in any way. To try to do so would completely contradict the experience. Remember, stillness is an experience of *not trying*." – p. 224<br>"There is no effort. No technique. No need to try in any way, or do anything." – p. 225<br>"[T]he calming occurs without our trying or making any effort at all. Metaphorically, as long as we chase the butterfly in the garden it will continue to elude us – but if we sit quietly and wait patiently, it is likely to land on our shoulder." – p. 250
McKinnon (2011)	"Perhaps the greatest attribute of [Stillness Meditation] is its absolute simplicity and naturalness. In this approach to meditation there is no need to *do* anything." – p. 33<br>"[Stillness Meditation] is characterised by its absence of technique ... [Meares] definitely did not subscribe to any effort being made ..." – p. 39<br>"[In Stillness Meditation] there is no focus ... or any kind of doing." – p. 39<br>"The calm of one's being is right there within. We don't have to *do* anything to access it: we simply learn and experience its existence in complete stillness, effortlessly. No technique is required to experience the sense of one's innate calm." – p. 40<br>"One learns to *let go* physically and mentally, *without effort of any kind*." – p. 40<br>"... without effort of any kind ..." – p. 45<br>"In essence ... Stillness Meditation is one utterly simple experience of *doing nothing*." – p. 45<br>"[Stillness Meditation] is not concerned with learning a technique ..." – p. 45<br>"[In] Stillness Meditation ... we don't have to actually *do* anything." – p. 65<br>"Stillness Meditation offers the choice to do nothing; the permission to just *be*." – p. 74<br>"No effort of any kind is made – but for your personal desire to make some changes." – p. 74<br>"Stillness Meditation involves no technique." – p. 75<br>"Stillness Meditation does not require an act of will. Any making or trying will obstruct [the experience]. There again is that paradox: [T]he more we try the less calm we can capture." – p. 75<br>"Stillness Meditation is a simple ... experience of learning to let go ... but more than letting go, just simply letting ..." – p. 75<br>"... [D]irecting the mind in any way ... is not conducive to [Stillness Meditation]." – p. 76<br>"... [L]ittle by little ... mental activity slows down and gradually, gradually, by doing nothing, stillness effortlessly follows." – pp. 76-77<br>"... [T]here is nothing to *do* ... *You don't have to do anything* ..." – p. 78<br>"Stillness Meditation is *always* an experience of *not doing* ..." – p. 79

	"There is no effort to be made at all. No technique. No need to try or do anything. There is only the experience of letting yourself be ..." – p. 80<br>"There is no effort. Nothing to do, no technique ... No need to try in any way, or to *do* anything ..." – p. 81<br>"You can't 'make' your mind be still. That's contradictory to the whole idea. All that is required is to 'let' or 'allow' the mind to *experience* effortlessness." – p. 100<br>Quoting a client, "Mary": "... I simply had to learn to relax my body and not strive to achieve anything. Then, I found, the stillness comes; it just happens!" – p. 172<br>Quoting a client, "Amy": "... method without method ..." – p. 179<br>"... [T]here is no technique ..." – p. 226<br>"[Stillness Meditation] is characterised by an absence of technique. It involves no deliberate use of the mind." – p. 228<br>"... [T]his is an experience of *not* doing." – p. 230<br>"... free of ... effort of any kind." – p. 238
McKinnon (1991)	"In his writings Dr Meares carefully avoided the use of the word 'technique' when describing his way of meditation. He emphasized that a technique implies structure and logical thinking, whereas his idea of meditation is a way that is arrived at without the logical or active use of the mind." – pp. 74-75<br>"... [Stillness Meditation is an] exercise ... of *not trying*. It is a totally effortless experience ... The paradox of [Stillness Meditation] is that the less effort we make, the more we achieve." – p. 76<br>"[Stillness Meditation], the art of doing nothing, is as simple and natural as just *being*." – p. 78<br>"... [N]o need to do anything ..." – p. 127
McKinnon (2002/2008)	"[Stillness Meditation] is not related to any kind of mind control ..." – p. 15<br>"Though [Stillness Meditation] does not involve a formal technique, it must be taught correctly." – p. 27<br>"... [Meditators] feel safe to just 'be' – without having to make any effort [and] without using the mind to direct or create the experience." – p. 45

1.9 Mind-wandering

See also 1.15.2 ("Dealing with thoughts") and 1.14 ("Progression to the goal-state/s").

Meares (1967/1968)	"[In regression] we let our mind wander, and are no longer concerned with our immediate surroundings." – pp. 82-83. "... [W]e leave our mind to wander uncontrolled as in a state of reverie." – p. 93
Meares (1969c)	"When you have finished your practising, you may realise that your mind has been roving quite uncontrolled over all manner of topics ... It is an experience similar to normal reverie, and it indicates that the desired regression is beginning to come quite naturally." – p. 86
Meares (1973b)	"[In Stillness Meditation] you just allow your mind to run free. You let go; your mind takes on activity of its own." – p. 254<br>"As you let go, ideas may come to you; but if you are letting your mind run free, they are just ideas. They are not a series of thoughts leading one to the other ... With a little experience it is usual for the ideas ... to become progressively fewer." – p. 254
Meares (1989)	On thoughts where the mind just jumps/flits from topic-to-topic: "These wandering thoughts are on the path to meditation, and they will come now and then even when you are quite experienced." – p. 115
McKinnon (1983/2016)	"... [G]radually our rational thought becomes suspended ... Thoughts become disconnected and unimportant to us." – p. 211<br>"Thoughts lose their logical or critical content and become barely distinguishable ..." – p. 221<br>"With practice, thoughts will separate and recede, becoming more dream-like and less logical." – p. 269
McKinnon (2011)	"In [Stillness Meditation] we allow the mind the freedom to roam without our attention." – p. 100<br>Quoting a client, "Jennifer": "Initially I knew I was thinking. But, as instructed, I paid no attention and the thoughts became more dreamlike than actual." – p. 101<br>"... while thoughts go free and melt in peace." – p. 203
McKinnon (1991)	"The mind will be busy in the first attempts at practice ... [L]et the mind run freely until, of its own accord, and in due course, it relearns the ability to relax and be still." – p. 77

1.10 Slight/minor discomfort
See also 1.11 (“Posture”).

Meares (1967/1968)	“Assume a position that is not too comfortable.” – p. 68<br>“... [T]he most effective relaxation for releasing our inner tension comes when we achieve relaxation while we are slightly uncomfortable physically.” – p. 68<br>“... [I]t is more difficult to attain real mental relaxation if we are physically too comfortable, because we then achieve our feeling of relaxation through the physical comfort of our body and not by activity of our mind.” – p. 59. Meares gives the example of lying comfortably on a bed (p. 68).<br>“... [S]ome minor physical discomfort is very important ...” – p. 69<br>“We practice ... until we can do it quite easily and naturally. Then we do it in increasingly uncomfortable situations.” – p. 161. “We can practice in positions of varying discomfort according to our taste and the degree to which we have mastered the exercises.” – p. 91<br>“... [O]ur mind [becomes] calm and relaxed so that the discomfort simply does not disturb us. And because it does not disturb us, we do not really perceive it as discomfort at all.” – p. 163<br><br>Meares pp. 58-59, 68-70, 82, 91, 160-163 makes other statements along similar lines to the above.
Meares (1978/1986)	Meares (1978/1986, pp. x, 7, 10-11, 33-47, 48-51, 53-58) addresses this point in more detail than Meares (1967/1968) above, but the gist is the same.<br><br>To highlight select points:<br>“... [T]he beginner needs to be reasonably comfortable ...” – p. 10. “We need to start our experience of physical relaxation in a position of comfort, but this can be overdone.” – p. 10. Accordingly, “[t]he first step is to sit in [an] armchair ... in complete comfort, but at the same time ... in a position of symmetry” (p. 7). “We do not just flop into the chair with our body slumped ...” – p. 7<br>“I have overwhelming evidence for the importance of discomfort in meditation. It rests on my experience with many hundreds of patients. There is no doubt whatsoever that those patients who have combined the judicious use of discomfort with their meditative practice have, in fact, gained much more from the experience. I could quote you many case histories to prove the point.” – p. 48<br>“... [Stillness Meditation involves] relaxation in the face of minor discomfort.” – p. x<br>“... [Stillness Meditation requires] such a posture that we are just slightly uncomfortable.” – p. x<br>“... [I]n [Stillness Meditation] we need to commence our meditation in circumstances of slight discomfort. ... Then, as our meditation proceeds, we cease to be aware of our initial slight discomfort. This transcendence of slight discomfort is an essential feature of [Stillness Meditation], and it is conveniently arranged by assuming a posture suitable to our own individual personal needs.” – p. 33. “We let our mind relax, and the discomfort has gone.” – p. 53<br>“The means of producing the discomfort is immaterial.” – p. 53<br>Meares discusses how the discomfort can be produced by the posture, or by other methods such as a clothes peg pinching the skin, lying on a rough surface, or being in the cold (pp. 53-58).<br>“As we relax, our mind transcends the discomfort of our posture and then there is no discomfort. This is really the most important aspect of the whole process of meditation. It is the getting beyond discomfort which allows us to experience the essential stillness of mind.” – p. 48<br>“The transcendence of minor discomfort in [Stillness Meditation] deepens the meditative process. ... [The] depth of meditation comes to us more easily and more completely when we have transcended some degree of discomfort.” – pp. 48-49<br>“... [W]e are seeking a posture that is slightly uncomfortable, but the discomfort must not be so great that we cannot transcend it ...” – p. 38 “If we make ourselves so uncomfortable that we are unable to transcend the discomfort, the whole process fails, and the level of our anxiety is increased.” – p. 50 “If you should find that you are making yourself endure discomfort, there is something wrong. Either you have not yet learned to let your mind relax ... or you may have been too enthusiastic and set yourself in a posture of too great discomfort for your present skill in meditation” – p. 53<br>“In meditation it is very easy to acquire the self discipline of transcending physical discomfort.” – p. 50<br>“At first the degree of discomfort should be very small indeed. Just enough to be very slightly uncomfortable. By starting with only a minimum of discomfort we do not set ourselves a task that is beyond our ability to achieve.” – p. 49
Meares (1973b)	“I don’t want you to endure discomfort, I want you to transcend it. To use your mind so that the discomforting influence simply does not worry you. Then of course there is no discomfort.” – p. 252

Meares (1989)	Meares speaks of the meditator's first attempt at Stillness Meditation: "Sit in a comfortable chair. ... Lie on the floor, if you prefer it. Comfy. In these first stages we need to be comfortable." – p. 110 "... [W]hen we first start off, we should be quite comfortable. No discomfort at all." – p. 119<br><br>On the need for minor discomfort at subsequent stages...<br>"Enduring pain involves striving and act of will. None of this is for us ... If you are in pain, how can your mind be silent, when striving to overcome it? ... So we do not sit in a posture that is painful." – pp. 117-118<br>"In [Stillness Meditation], we meditate in a posture that is slightly uncomfortable. Only slightly uncomfortable. So that when we start to meditate we cease to be aware that we were slightly uncomfortable. We transcend the slight discomfort. What I have just said is not quite right. We let our mind transcend [the] slight discomfort. No striving. We let it be transcended." – p. 118<br>"The discomfort must never be more severe than we can transcend without effort." – p. 119<br>"We must keep changing our posture from time to time as our meditation gets better, and our ability to transcend slight discomfort is improved." – p. 118 "... [A]s we become better at meditation our mind learns to ignore minor discomforts, so we need to increase our discomfort so as to give our mind sufficient to work on, as it were." – p.121<br>"I have seen as patients many people who have been practising in comfortable postures without gaining any help, but when they have switched to our way of doing it, they have been astounded at their improvement." – p. 118
Meares (1987/1991)	"We let our mind run quietly, and we are soon unaware of the initial slight discomfort." – p. 80<br>Meares p. 115 makes the same comment.<br>"Remember the floor is the only place to learn it. You cannot learn it lying in bed ..." – p. 80<br>"It does not matter how we induce the slight discomfort. This will depend on age, and on the physical and mental condition of the meditator." – p. 115. "The important factor is that the discomfort must never be so great that it is not transcended in the first few minutes of meditation." – p. 115
Meares (1983a)	"Enduring pain makes the stillness impossible. On the other hand, meditation in a position of comfort produces a kind of drowsy numbness which is quite different from the crystal clear stillness of [Stillness Meditation]." – p. 118<br>"... [A]s the meditation comes, the slight discomfort is transcended ... It is very important that this procedure be continued in progressive fashion." – p. 118<br>"The only purpose of the posture is to provide a degree of discomfort which is commensurate with the meditator's ability to transcend it." – p. 118

1.11 Posture

See also 1.10 ("Slight/minor discomfort").

1.11.1 Meares' overarching principle, and McKinnon's contrasting perspective

Meares (1978/1986)	"In [Stillness Meditation] it does not matter what posture we assume provided certain conditions are satisfied. Our posture must provide some initial slight discomfort which we transcend as our meditation proceeds ... It must give us the feeling of doing with ease something which is really slightly difficult, and it must be such that it prevents us falling asleep. Beyond these considerations the actual nature of our physical posture is immaterial." – pp. 34-35
Meares (1989)	"The posture in which we meditate has only one purpose, that is, it must provide a slight degree of discomfort, which we can transcend when we start to meditate." – p. 119<br>Meares p. 124 makes a comment along the same lines.
McKinnon (1983/2016)	McKinnon p. 271 comments on postures alternative to sitting on a straight backed chair: "I think [such advanced/alternative postures] belong in the realm of esoteric practices and I don't think they are necessary for [Stillness Meditation]." McKinnon then notes, however, that some of those postures may be appropriate for certain people/circumstances (see 1.11.3, 1.11.5, and 1.11.6 below). Referring apparently to Meares' idea of progressively increasing the discomfort, McKinnon p. 272 says, "... I believe it's unwise to flit from one ... posture to another too frequently – it seems to me that the mind enjoys regularity".

McKinnon (2011)	"I think advanced postures are more suited to taking place within practices such as yoga ... [T]he upright posture, simply seated in an average chair, will always be more suitable. Advanced postures are not necessary for a better stillness experience." – pp. 102-103

1.11.2 Definite/symmetrical posture

Meares (1967/1968)	"Use a symmetrical posture." – p. 69<br>"... [O]ur mental relaxation is more effective if we retain a symmetrical posture ..." – p. 70
Meares (1978/1986)	"... [W]e should assume a definite posture to practise [Stillness Meditation]." – p. 33<br>For example, in the armchair the meditator maintains symmetry and keeps their head up, rather than letting it fall forwards (p. 33). "By assuming a definite posture we prevent ourselves falling asleep." – p. 34

1.11.3 Lying

Meares (1967/1968)	"[The lying posture] is the basic posture and the easiest ..." – p. 70
Meares (1978/1986)	"We can meditate lying on the floor ... but never in bed. Bed is too comfortable, and the experience of ease comes from nerve endings in our body and not by act of our mind." – p. 37
Meares (1987/1991)	"Remember the floor is the only place to learn it. You cannot learn it lying in bed ..." – p. 80
McKinnon (1983/2016)	"Lying flat on the floor can be helpful if you are extremely tense, or if through illness or pain you are unable to withstand sitting for any length of time ... But in my experience, lying flat ... never quite brings about the mental relaxation that is conducive to deep stillness." – p. 272
McKinnon (2011)	"When lying down, there is an assumption of rest and sleep. But it's not the state of mind that occurs in pure stillness. I suggest [lying down] only when extreme pain or severe illness prevents the upright posture." – p. 102

1.11.4 Sitting – general

Meares (1967/1968)	"[When using a sitting posture] [a]t first it is wise to use an armchair ..." – p. 70<br>"It is best to sit up in the chair rather straight without the body slumped ..." – p. 70<br>"When a fair degree of relaxation can be attained in an armchair, try a straight-backed ... chair." – p. 70
Meares (1978/1986)	"It is best to make our first venture into [Stillness Meditation] just sitting in an armchair" – p. 7<br>"The transition from our armchair to the upright chair is a necessary step in [Stillness Meditation]." – p. 36<br>"... [W]e sit in the upright chair so that we ourselves are upright, and during the whole of our meditation we maintain our upright position without slumping forward ... Arms are on [our] thighs ... Head is erect. It is erect but at the same time easy." – p. 36<br>"During the first few sessions we may find that our head slowly sags forward, and we have to bring it back to the upright position ... With a little practice we find that our head is maintained in the upright position without conscious effort on our part. It just stays there. Quite effortlessly." – p. 37<br>Meares discusses other progressions in sitting posture that can be made, for example sitting on the edge of the chair (with the back no longer supported), and sitting on a stool (p. 36).
McKinnon (1983/2016)	"... [T]he most suitable posture is sitting erect in a straight backed chair with your feet resting flat on the floor ... Your head and neck are held erect – not tense, just evenly balanced ... When seated correctly one is poised in calm control – relaxed yet disciplined, upright but at ease." – p. 219<br>"Seated symmetrically ... we remain poised and alert ... Our body is disciplined, erect and still ... We are completely balanced, poised and at ease." – p. 224
McKinnon (2011)	"... [S]light discomfort means being seated symmetrically, erect and balanced in any kind of upright chair. A common chair provides sufficient 'discomfort' ... This posture keeps the meditator poised and alert. It also provides a sense of calm control ..." – p. 67<br>"... [J]ust sit, symmetrically, erect and balanced on an upright chair ... Your spine is upright, your head erect and balanced ... The posture helps you remain poised and alert and with a sense of calm control." – p. 78

1.11.5 Cross-legged sitting

Meares (1967/1968)	“In this [posture] we sit on a cushion cross-legged on the floor.” – p. 71<br>“Try to keep the back and the neck fairly straight so that the muscular effort to maintain the position is reduced to a minimum.” – p. 71
Meares (1978/1986)	“[Sitting on the floor] with legs crossed is the most generally satisfactory posture for those who can manage it ... Our back [is] straight and our head held erect.” – pp. 37-38
McKinnon (1983/2016)	“Sitting on the floor cross-legged ... may suit some very well.” – pp. 271-272

1.11.6 Kneeling

Meares (1978/1986)	“We can meditate in a kneeling posture.” – p. 41<br>“In the kneeling posture we need to maintain much greater control.” – p. 41
McKinnon (1983/2016)	“... [K]neeling is challenging and takes practice – it is not for beginners.” – p. 271

1.12 Control and relinquishment, discipline and ease

See also 1.8 (“Effortlessness”), 1.15.3 (“Letting go”), and 2.3.26 (“Control and relinquishment”).

Meares (1967/1968)	Meares indicates that Stillness Meditation involves a “self-discipline” that “comes easily, naturally, and without effort” (p. 162).
Meares (1978/1986)	“The symmetry of [the posture] ... introduces ... the feeling of some control into the apparently paradoxical feeling of utter relaxation.” – p. 7<br>“Although our relaxation is global and complete ... there remains sufficient tone in certain muscles to maintain posture.” – p. 11. For example, in the armchair posture: “We need to maintain just sufficient tone in the muscles of our neck to prevent our head flopping to one side or falling forward. When we have captured the experience, the maintenance of our head in an erect position comes quite naturally, effortlessly and without our awareness that we are, in fact, keeping it in this position.” – p. 11<br>“The effortlessness of [Stillness Meditation] initiates us into the discipline of ease.” – p. 23<br>“... [W]e learn to do something which could be difficult, and we learn to do it quite effortlessly.” – p. 23<br>“Pure being is a defenceless state, and unconscious protective reactions intervene to prevent us exposing ourselves to the potential danger of defencelessness.” – p. 27<br>“By assuming a definite posture for [Stillness Meditation] we establish the idea of easy control and self-discipline. In one sense the maintenance of a definite posture is a task which involves some effort and some difficulty. But in [Stillness Meditation] ... the same task becomes quite effortless and involves no difficulty whatsoever.” – p. 34<br>“The [cross-legged seated posture] provides opportunity for the complete relaxation of some muscle groups and at the same time the maintenance of tone in others, and so enhances our experience of simultaneous relaxation and control. Controlled ease. The Discipline of Ease.” – pp. 39-40<br>“... simultaneous experience of complete relaxation and effortless control.” – p. 40
Meares (1973b)	“[Learning the relaxation of our muscles, the experience of that relaxation in our mind, and letting our mind transcend discomfort] takes a little time and a little practice. We need to strive for most things that are worthwhile. So it is with this. But remember that in this the striving has this utterly effortless quality about it which means that it is no striving at all.” – p. 254
Meares (1989)	“[Stillness Meditation] requires an element of self-discipline, not much, but some.” – p. 107<br>Taking the seated posture as an example: “You will remember that we started off with the physical relaxation of the body. This must never be complete in all the muscles of the body. If it were, we would be flopped like a jelly fish.” – p. 125. “[W]e must maintain enough tension in the muscles of the neck to hold our head in an upright position. But the tension in the muscles that holds our head from flopping is natural and effortless, just as it is in our ordinary life.” – p. 125
Meares (1987/1991)	“It is a kind of easy self-discipline that comes naturally and effortlessly.” – p. 125
Meares (1970)	“Cultivate an effortless self-discipline.” – p. 77

Meares (1976/1980)	"... an effortless type of self-discipline." – p. 146<br>"... a kind of easy, natural, effortless self-discipline." – p. 146<br>"... effortless self-discipline ..." – p. 147
Meares (1988)	Having discussed conventional ideas of discipline as involving austerities: "However, there is another form of discipline. I call it the Discipline of Ease: the discipline of relaxation of the mind. The idea is this: Discipline need not be something that is nasty and unpleasant, something that we would avoid if we could. If we can get our mind running right, self discipline is there without any effort on our part at all. But when we say that it is 'easy' we must not think of it as 'slack'. No. That is not it at all. A firm discipline, but being quite easy and relaxed about it in our own self." – p. 96
Meares (1978c)	"[In regression] [t]he mind is no longer controller ..." – p. 87
Meares (1977b)	On cancer patients using intensive Stillness Meditation as a treatment, Meares reflects:<br><br>"... Fight without fight.<br>No effort in that ..." – p. 108
Meares (1979b)	"... When is striving not striving? ...<br>When we strive<br>With ease in our mind!" – p. 4
Meares (1984)	"... In the meditation ...<br>We abandon ourselves<br>Freely<br>As a falling leaf<br>In the autumn wind ..." – p. 76
Meares (1987a)	"... Ease comes of the union<br>Of restraint and abandonment.<br><br>We can learn restraint<br>In the discipline of meditation,<br>And abandonment comes<br>In the letting go of meditation ..." – p. 47
McKinnon (1983/2016)	"In [the seated] posture we gradually develop a sense of calm control, which, paradoxically, leads to ... the giving in of total 'global' relaxation." – p. 224<br>"A disciplined experience of effortlessness." – p. 224
McKinnon (2011)	"[The seated] posture provides a sense of calm control, from which, believe it or not, we are more able to let go into full 'global' relaxation." – p. 67
McKinnon (2002/2008)	"... [The] posture results in a pleasant and balanced sense of ease and control." – p. 36

1.13 No focus/concentration

See 2.3.22 ("No focus/concentration").

1.14 Progression to the goal-state/s

See also 1.6 ("Major presentations of the practice"), 1.9 ("Mind-wandering"), and 1.16 ("Unusual subjective phenomena").

Meares (1978/1986)	"First of all [the mind's] logical, critical activity is reduced until this finally ceases. Then the vividness of our perception is reduced, noises come to be heard as sounds with little or no meaning. As the process continues there is little or no thought, and little or no awareness of sounds or activity around us. The process continues, and stillness comes to us." – p. 6<br>Stillness Meditation involves a "progressive reduction in mental activity" (p. 6).
Meares (1973b)	"When we practise [Stillness Meditation], the mind empties itself ..." – p. 254

McKinnon (1983/2016)	"Initially, as you sit to practise, there will be many distractions in the form of aches and pains and a stiff neck, or back or muscles in general. There may be boredom, irritation or despair. Itches will become evident for no apparent reason and the desire to cough or to sneeze may tempt you." – p. 217 "There will also be awareness of tension and there may even be mild panic or other unwelcome sensations. But please stay with it. Your body is learning something new; your *mind* is learning something new." – p. 217. "Through daily stillness, in due course you will begin to feel at least physically relaxed and you might begin to enjoy the time spent in this way ... As physical relaxation develops, activity in the brain slows down and the mind begins to relax as well. But at first, mental rest is elusive." – p. 217 "In time, the activity of the mind *will* diminish. Intermittently, moments of calm will occur. At another time, or perhaps on another day, more calm descends and thoughts are less intrusive, until eventually in exquisite stillness, nothing disturbs us at all." – p. 218
McKinnon (2011)	"In Stillness Meditation we enter the space with our mind busy, distractions and thoughts buzzing, doubts and fears arising, impatience surfacing. But ... little by little the mental activity slows down and gradually, gradually, by doing nothing, stillness effortlessly follows." – pp. 76-77<br>"In the first learning stage, awareness may be present and distractions, discomforts or even fear may interfere with the stillness ... [In the second stage] you will find, gradually ... the ability to surmount any disturbance ... Only calmness prevails ..." – p. 84<br>McKinnon pp. 101-102 quotes a client, "Jennifer", describing her first experience of Stillness Meditation, attending a class with McKinnon: "... [S]oon I began to feel my body becoming warm and heavy ... Initially I knew I was thinking. But, as instructed, I paid no attention and the thoughts became more dreamlike than actual. Also at first I was quite aware of my surroundings, of others in the room, of [McKinnon's] presence, but after a while that awareness disappeared. Towards the conclusion of the session there was no visual image at all. Instead, I was, it seemed, embraced in a warm greyness or nothingness really – yet it felt like everything. My mind was light and my body was heavy. Well, actually, I couldn't feel my body at all; I just knew it was there. But this was a very pleasant sensation. I wasn't asleep either, because I knew what was happening. Everything I needed – safety, security, strength, peace – was within that stillness."
McKinnon (1991)	"At first one would be fairly alert and guarded, a natural human response. Then relaxation would gradually occur, notably in the arms and legs and later, as tension lessened, throughout the whole body in a complete and global 'letting go'. The mind, of course, being the most alert, takes much longer to relax. Most people can usually relax their body fairly readily after repeating the process regularly for a time. But the mind, being our prime measure of defence, takes longer as self-awareness and the awareness of noise or the presence of others is inclined to be distracting at first. For me, it took a great many sessions before I really allowed my mind to be free and therefore still. The higher the anxiety and tension the greater the defence. But in its own time it occurred, bringing with it a velvety 'nothingness'." – p. 73<br>"At first stillness of mind will be impeded by obstacles such as too many thoughts, itches and aches, perhaps anxiety and other distractions. If we ignore these, gradually, as we sit there, ideas will fade, thoughts lose their logical content and awareness of our surroundings becomes unimportant. Noises and distractions are transcended and the whole self is very still ..." – p. 77
McKinnon (2002/2008)	"As the meditation commences students will be aware of their physical bodies, but, as deep relaxation takes place, movement gradually slows and sensation ... diminishes as physical awareness lessens. Everyday sounds ... will diminish and physical discomfort [and] surrounding distractions will become insignificant." – p. 45

1.15 Difficulties and how to deal with them

The difficulties described below are those most relevant for this table. For a description of other difficulties, and how they can be dealt with, see McKinnon (2011, pp. 112-121).

1.15.1 Trying too hard

See also 1.15.2 ("Dealing with thoughts") and 1.15.3 ("Letting go").

Meares (1975)	"With those who have had difficulty in mastering [Stillness Meditation], the trouble has nearly always been that they are trying too hard. Noone can force himself to relax. It is just an effortless experience." – p. 50
McKinnon (2011)	"It can ... be difficult – at first – not to try too hard to achieve stillness." – p. 40

1.15.2 Dealing with thoughts

Meares (1967/1968)	"A common difficulty is that our thoughts seem to become too active." – pp. 85-86 "The main problem is ... the way they intrude into our consciousness and ... disturb us." – p. 86<br>"We cope with this difficulty simply by giving our thoughts full rein ... and letting them wander where they will without us worrying about them ... Our thoughts wander far and wide; but it does not disturb us ... We just let the thoughts go ... We let them go easily, just where they like, and it does not worry us." – p. 86<br><br>"There is another approach to this same problem ..." – p. 86<br>Meares explains that it involves returning to the progressive relaxation of the muscles (p. 86).<br>"... [T]he mind is kept occupied so that other thoughts do not get the chance to intrude and worry us." – p. 86
Meares (1978/1986)	Meares (1978/1986, pp. 30-31) makes similar comments to those in Meares (1967/1968) above.<br><br>To highlight select points:<br>"The most common barrier to the relaxation of the mind is the persistence of unwanted thoughts." – p. 30<br>"The important point is that ... unwanted thoughts should not disturb us." – p. 30<br>"Unwanted thoughts that are [realistic/reasoned/logical/sensible] can be quietened simply by [temporarily] feeling the relaxation of the body." – p. 30 "[Once the thoughts are quietened] [w]e are able gradually to change from the conscious feeling of relaxation to the simpler mental process of just experiencing it ..." – p. 31<br>"When our unwanted thoughts are merely flitting around in unreasoned fashion we can simply let them run on ... and they gradually settle down." – p. 31
Meares (1989)	To an imagined question from a student, "I can't think of nothing", Meares' responds: "... If we just coast along with [the experience], our mind goes quiet of itself, without us doing anything about it." – p. 46<br>On thoughts where the mind just jumps/flits from topic-to-topic: "... [J]ust ignore it. Let it go. Take no notice of it. Above all, don't let yourself get fussed by it." – p. 115
Meares (1987/1991)	"At first, until the meditator has learned [Stillness Meditation] there will be moments of stillness, but these are soon interrupted by the intrusion of thoughts. Do not try to dispel the thoughts by actively driving them from the mind. Just let them be, and they will fizzle out, cease, and stillness will come again. Then thoughts will recur. And again, if they are let alone, stillness will supervene. And gradually the space between the thoughts, the stillness that we want, will become longer and longer." – p. 114
Meares (1988)	"Thoughts may come. Don't let them disturb you. Don't try to force them away. Just let them be, and in a few moments they fizzle out. ... Don't let the thoughts worry you or try to drive them out." – p. 74
Meares (1976/1984)	"... What stops it coming?<br><br>Questions, of course.<br><br>Questions beneath the surface.<br>How can there be calm,<br>With questions beneath the surface?" – p. 30
McKinnon (2011)	"Your mind may be active. Seeking to focus, to pay attention, to construct, to resolve, to process information, to chatter on any topic that presents itself ... You simply disregard this unnecessary intrusion." – p. 81

1.15.3 Letting go

Meares (1967/1968)	"[A common difficulty] is that the anxious person is continually holding himself in, while in [Stillness Meditation] he is asked to let himself go." – p. 88. "We are really rather afraid to let go. This applies to all of us, but especially to those who are tense and anxious. When we suffer from tension we are all the time holding ourselves in, as it were. We keep a hold on ourselves. We keep ourselves in check." – pp. 87-88<br>"Some people experience difficulty in that they remain too alert to everything that is going on around them." – p. 88<br>"There are two things which help us to overcome this difficulty." – p. 88

	“The first is that we know that we are completely safe.” – p. 88<br>“The other way ... is to become more and more familiar with the sensation of letting go.” – p. 88<br>Meares pp. 87-89, 99-100 provides more fine-grained detail.
Meares (1978/1986)	Meares (1978/1986, pp. 15, 21, 25-29, 113-114) addresses the points made in Meares (1967/1968) in further detail, but the gist is the same.<br><br>To highlight select points:<br>“Pure being is a defenceless state, and unconscious protective reactions intervene to prevent us exposing ourselves to the potential danger of defencelessness. When we first attempt to just let ourselves be, we often find that against our conscious wish there is something keeping us alert. This is a manifestation of an unconscious protective reaction. It may show itself in various forms.” – p. 27. Meares notes that these forms include: remaining alert (i.e., failing to participate in the relaxation; pp. 15, 21, 27); the mind becoming unusually active (p. 27); becoming unduly restless (p. 27); a transient panic reaction (pp. 25-26, 28); and a sudden jerk of the whole body (p. 28).<br>“Letting ourself go. This is unguarded behaviour. It is risky. Inborn protective mechanisms hold us back. We find that we cannot let go really freely, although we would very much like to do so.” – p. 29.<br>“We can get around the unconscious protective reactions that prevent us from letting go. We do this by repetition. Familiarity brings a sense of security. Each time we relax, it is as though our unconscious mind lets us go a little further. Gradually it learns that no harm comes to us from this experience, we find that we are no longer holding ourselves back, and we are able to let go more freely.” – p. 29
McKinnon (1983/2016)	McKinnon notes as part of her description of her initial meditation sessions: “But within myself I questioned myself ... the anxiety that welled up within me at intervals ... the eerie absence of any kind of stimulus as my mind grew used to letting go.” – p. 191 Later she notes how some increase in anxiety in initial sessions is not uncommon, reflecting the idea that the mind is learning something new (p. 273).
McKinnon (2011)	“... [P]erhaps the most challenging task for the novice meditator is the act of letting go ... Letting go might sound quite simple. But this is not so for all people! Many, especially those whose anxiety is high, need generous encouragement in the act of letting go.” – pp. 59-60<br>“As the stillness is repeated more and more, the mind learns gradually that it is safe to let go.” – p. 60<br>McKinnon p. 60 makes other similar comments to those in the two passages above.<br>“In some cases, a temporary rise in anxiety may occur as part of learning [Stillness Meditation] ... [T]he brain is learning that it’s safe to let go *beyond* thought and feeling and *beyond* disturbance, rather than remain attentive, guarded and tense.” – p. 77<br>“It is not uncommon to feel afraid or to experience anxiety while [in the initial learning phase] ...” – p. 113<br>“It is in having the courage to let go beyond fear that change becomes possible.” – p. 114<br>Quoting “Brian”, a former patient of Meares: “Not easy at first, but after some months something happened and I became a different person. It was like magic.” – p. 191
McKinnon (1991)	“At first one would be fairly alert and guarded, a natural human response ... The mind, of course, being the most alert, takes much longer [than the body] to relax ... [T]he mind, being our prime measure of defence, takes longer as self-awareness and the awareness of noise or the presence of others is inclined to be distracting at first. For me, it took a great many sessions before I really allowed my mind to be free and therefore still. The higher the anxiety and tension the greater the defence. But in its own time it occurred, bringing with it a velvety ‘nothingness’.” – p. 73
McKinnon (2002/2008)	“In learning to let go in [Stillness Meditation], it is normal for many to experience a temporary increase in their anxiety ... This is a very normal response in learning to let go and occurs when, in the process, one is temporarily off-guard ... However, this reaction is unlikely to recur once the need for stillness is accepted and the practice is established and maintained.” – p. 40

1.15.4 Sleep

Meares (1967/1968)	“If you have a tendency to fall asleep [during Stillness Meditation], use a more uncomfortable posture ...” – p. 89
Meares (1978/1986)	“By assuming a definite posture we prevent ourselves falling asleep.” – p. 34<br>“... [Our posture] must be such that it prevents us falling asleep.” – p. 35

Meares (1989)	"If you tend to go to sleep, or even get drowsy, just meditate in a slightly less comfortable position, and the trouble about going to sleep vanishes." – p. 117
Meares (1987/1991)	"If we are concerned about sleep, it is best to [meditate] lying flat on your back on the floor." – p. 80
Meares (1969b)	"Some degree of discomfort is ... necessary to prevent one falling asleep." – p. 197
McKinnon (1983/2016)	"Increased alertness can be maintained by keeping the posture more erect, using an even harder chair or stool, keeping cooler or temporarily making the mental decision to aim to stay awake ... [I]f you are simply too tired ... go to bed and truly sleep instead." – p. 271
McKinnon (2011)	"... [I]ncrease the physical discomfort a little. Avoid being too comfortable in terms of cosiness or warmth. Or, you may in fact be in need of sleep." – p. 105
McKinnon (1991)	"Slight discomfort keeps us alert though relaxed ..." – p. 126
McKinnon (2002/2008)	"... [D]rowsiness can be lessened if [the meditator] increase[s] the element of discomfort in their posture and if the teacher ensures that the room temperature is not too warm. The student can also be encouraged to actually determine to remain alert." – p. 41

1.16 Unusual subjective phenomena

Meares (1978/1986)	"As our mind relaxes we may come to experience various subjective phenomena. Other than the experience of very great calm and ease, subjective phenomena are not a part of [Stillness Meditation]." – p. 156<br>"Strange subjective phenomena cease spontaneously unless they are purposely catalysed by act of mind. If you will ignore any strange subjective phenomena, they will soon peter out. Their demise can be accelerated by actively experiencing our own inner calm and ease." – p. 156 "The important consideration is that these phenomena soon cease of their own accord unless the individual keeps activating them purposely by act of mind." – p. 157<br>Meares describes some of the phenomena, including: sensations concerning the body, such as floating or the body or face seeming much larger than usual (pp. 157-158); visual phenomena (colours, patterns, shapes; p. 158); and dramatic changes in mood (p. 159).
Meares (1973b)	"[In Stillness Meditation] you just allow your mind to run free. You let go; your mind takes on activity of its own ... [A] great variety of experiences may come to you." – p. 254 "... [Sensations] ... may come to you. ... There may be sensations of colour, of sound, or feelings in the skin or within the body itself." – p. 254<br>"With a little experience it is usual for the ... sensations to become progressively fewer." – p. 254
Meares (1989)	"Sometimes spontaneous sensory phenomena occur in [Stillness Meditation]. The meditator may see lights or colours ... He may get the feeling of movement, or of floating, or of being outside his body observing himself ... Ignore these phenomena. Do not seek them out. Do not be disturbed by them ... They are not common." – pp. 125-126
Meares (1976/1984)	"How do we gauge it?<br>What is our yardstick?<br>Some talk of wonderous things,<br>Of lights and shapes and colours,<br>Of the drifting soul,<br>Of voyage in space and time.<br>No.<br>This is not it.<br>Tinsel and gaudy.<br>Only the calm and the stillness ..." – p. 55
McKinnon (2011)	Quoting a client, "Jennifer": "At one stage, a variety of colours and images moved in and out of my mental view. I had been advised to ignore these ... and soon they passed." – p. 101<br>"[T]ransient happenings may include general discomfort, nausea, dizziness, feeling 'off-centre' or out of balance. Some may witness inexplicable phenomena such as out-of-the-body experiences, images, physical or visual sensations, etc. The advice is always to pay no attention to these and they quickly pass." – p. 115

1.17 Practice duration

Meares (1967/1968)	"About 10 minutes twice a day is all the time required for [Stillness Meditation]." – p. 71
Meares (1978/1986)	"Ten minutes twice a day is usually sufficient for most people." – p. x<br>"Most of us can make very great changes for the better with ten minutes [Stillness Meditation] two or three times a day." – p. 46
Meares (1969c)	In the context of a discussion about using Stillness Meditation as a way to generate greater output in study/work: "It takes you about twenty minutes or half an hour a day." – p. 87
Meares (1973b)	"Do it for ten minutes two or three times a day." – p. 257
Meares (1989)	"Ten or fifteen minutes daily in [Stillness Meditation] will do much to prepare ourselves to teach [it]." – p. 17
Meares (1987/1991)	"Five or ten minutes effective meditation in the morning will make an extraordinary difference." – p. 9<br>Meares p. 33 makes a similar comment.<br>"Ten minutes twice a day has produced dramatic relief [from stress] in some hundreds of people who have consulted me professionally." – p. 116<br><br>"Allow yourself five minutes meditative experience daily. Just five minutes." – p. 126<br>The difference between the guidance given here (5 minutes a day) and the guidance in the earlier part of the book (5 or 10 minutes either once or twice a day) probably reflects that the guidance here is provided in an appendix to the book, and the appendix was written in the early 1970s (p. 128).
McKinnon (1983/2016)	"... [W]hen challenges or stressful situations occur, not only do we maintain our regular times of stillness, but we learn to recognise that sometimes we need to increase those practise times." – p. 213<br>McKinnon p. 243 makes a similar comment.<br>"... [B]eginners should, as a minimum, practise for ten to twenty minutes twice a day. One can meditate for longer periods and can, if necessary, get by on a little less." – p. 269<br>"In the case of very challenging anxiety conditions such as agoraphobia ... I suggest sessions lasting one hour, at least once, if not several times a week ... over and above your regular practice times." – p. 270
McKinnon (2011)	"... [T]en minutes twice daily is a basic regular practice time." – p. 107<br>"Recommended personal practice is ten to 20 minutes twice daily." – p. 232

1.18 Can learn via non-class practice

Meares (1978/1986)	"... [W]e can in fact learn to [practise Stillness Meditation] without the presence of [a] teacher ... My evidence for stating this so dogmatically is in some hundreds of letters that I have received from people all over the world telling me of their improved health and state of mind after practising ..." – p. 29
Meares (1989)	"A great number of people all around the world have gained immeasurably from what they have read. I know this because they have taken the trouble to write and tell me about it. But the help that they have had comes from their doing what I describe, and not just from reading about it." – p. 14
Meares (1969b)	"Now every day I receive letters from people in many countries of the world saying that they have gained relief from practice of [Stillness Meditation]." – p. 11
Meares (1975)	".... I know this to be true from the number of older people whom I have never seen who have written to me about my book *Relief Without Drugs* [Meares, 1967/1968)]. Many of these people have described great changes in themselves ... by practising [Stillness Meditation] ..." – p. 11<br>"A great number of people have written to me saying they have [practised Stillness Meditation] and that it has changed their whole outlook on life." – pp. 49-50
McKinnon (1983/2016)	"Letters of appreciation and, later, many emails from across the globe ... have also come with a message of relief, gratitude and hope [in response to my sharing of my story]." – p. 12

McKinnon (2011)	"Some assistance from a teacher may be required as beginners learn to truly let go." – p. 41<br>"It is important to note that, with dedication, Stillness Meditation can be very successfully practised alone." – p. 42<br>McKinnon tells of a woman approaching her at a book signing. "Quietly she spoke to me: 'I read your book a few years ago. I have practised as you described and my life has been transformed'." – p. 42<br>"So while learning with an experienced teacher is ideal, it may not always be necessary." – p. 42<br>"While learning and practising through books and recordings can obtain some very good results, the effects of stillness will be magnified [through attendance at Stillness Meditation classes]." – p. 90<br>"... [T]he stillness experience is always easier to capture [in a class] than at home ..." – p. 118<br>Quoting "Sue", who practised Stillness Meditation in New Zealand after reading *Relief Without Drugs* (Meares, 1967/1968): "It didn't seem possible that relief could be so readily at hand. But it was. Very quickly I was able to let go and dissolve from a state of muscular relaxation to mental peace. It changed my life around." – p. 193<br>"For best results, and where it is possible, [the] benefits [of Stillness Meditation] can be increased by learning the skill with an experienced teacher and within a therapeutic environment." – p. 231<br>"Understandably, there are those for whom it may be impossible to access this level of assistance ... [W]ith the support of recommended texts one can begin the journey alone with excellent outcomes." – pp. 231-232
McKinnon (2002/2008)	"The deep state of stillness is always enhanced when experienced with the facilitation of a meditation teacher." – p. 39

Part 2: Goal-state/s

2.1 Participant-based academic studies of the subjective experience

Two quantitative and/or qualitative academic studies have examined the subjective experience of the goal-state/s in Stillness Meditation: Seymour (1999) and Hosemans (2017). Seymour is a qualitative study, and Hosemans uses mixed methods (qualitative and quantitative). The studies were undertaken after Meares' death in 1986. McKinnon's books were published prior to Hosemans (2017), but McKinnon (2002/2008, pp. 78-79, 2011, pp. 222-223) provide short summaries of the findings in Seymour. Seymour was based on interviews with six Stillness Meditation meditators and McKinnon. McKinnon (2002/2008, 2011) only notes one of Seymour's findings that concerns a relevant aspect of the Stillness Meditation goal-state/s: "Data ... indicated that there is only a slight difference between the experiences (within stillness) of experienced students and new students" (McKinnon, 2002/2008, p. 79, 2011, p. 223). McKinnon does not elaborate on the finding, and there is no evidence she relies on it in her more detailed descriptions of the goal-state/s.

2.2 Speed at which achieved

Meares (1967/1968)	"Do not expect too much too quickly. It does not come all at once, neither the relief of your [anxiety/tension] symptoms nor even mastering [the technique]." – p. 65 "In this respect it is well to remember that very dramatic changes in one's mental state, either for the better or for the worse, are in themselves evidence of instability. Be content with a slow and steady improvement." – p. 65<br>"It takes a little time to learn [the relaxing in Stillness Meditation]." – p. 65<br>"If you are able to capture just a moment of calm, it will not be long before you can achieve the full state." – p. 82
Meares (1973b)	On transcending minor discomfort: "What I am talking about is something quite simple, which anyone with a little patience can soon master." – p. 253<br>On learning the stages up to transcending minor discomfort: "[It] takes a little time and a little practice." – p. 254<br>"[The reduction in anxiety in Stillness Meditation] soon comes if you really practise the relaxation of the muscles and experience this in your mind." – p. 257
Meares (1988)	"... [W]hen we have done it for a few days we get moments of stillness, just moments ..." – p. 26
Meares (1971a)	Meares describes the small, private group sessions that he conducted: "Nearly everyone is in quite [a] deep [state of meditation] in a few minutes. My words give way to unverbalized phonation ... Silences develop, and the patients drift still deeper into [the meditation]." – p. 676

Meares (1973a)	Meares describes the large, public group sessions that he conducted: "Most of the people who come in [to the meditation room] first are well into [the] ... meditative state before the later arrivals have taken their seats." – p. 734 McKinnon (2011, p. 201) notes that members of the public were allowed one or two of the large group sessions at no cost, and if they wished to have further sessions with Meares would need to attend his small, private sessions, which were charged for. That suggests that the people in the large group sessions that Meares describes were attending only their first or second group session.
McKinnon (1983/2016)	"It's very important that I impress upon you that the ability to capture a state of stillness will not usually occur readily at first. In fact, this may not happen for quite a long time. Though the exercise itself is easy, for some people it can take up to two years of regular practice to reach deep and fulfilling stillness ... But be consoled: ... the experience will have a positive effect ... from the time you begin." – p. 217<br>"It may take some weeks of regular sessions before you even feel relaxed in any way at all." – p. 217<br>"The achievement of deep stillness takes time ..."– p. 269<br>"Some people can move into the stillness 'state' quite readily ... Our gifts differ, and what comes naturally to one may not do so for another." – p. 274
McKinnon (2011)	Quoting a client, "Jennifer", describing her first group meditation session following an introductory session with McKinnon: "I was suspended in stillness ..." – p. 101<br>"In learning to be still, people are often impatient in waiting for the changes to occur. We have to remember that, as individuals, our progress will always be individual. Some may progress almost immediately and others must persist ..." – pp. 114-115<br>Quoting a client, "Jenny": "It took a few weeks to experience the stillness fully ..." – p. 168<br>Quoting a client, "Mary": "... I found that a state of calm came to me very easily and quickly." – p. 172<br>Quoting a client, "Marguerite": "After three months I have learned and experienced so much. I can relax, I can enjoy moments of stillness." – p. 176
McKinnon (1991)	"Although some people find it can take time and many ups and downs before they feel they are freely meditating, the rewards begin as soon as the practice is embarked upon." – p. 96
McKinnon (2002/2008)	"Stillness Meditation obtains results without requiring lengthy procedures or years of training ..." – p. 16<br>"The mind will take longer to relax than the body and in some cases this may take some weeks of practise." – p. 45

2.3 Features

See also 1.6 ("Major presentations of the practice"), 1.14 ("Progression to goal-state/s"), and 1.16 ("Unusual subjective phenomena").

2.3.1 Recognize in retrospect

Meares (1978/1986)	"We may find that we ask ourselves, 'Is my mind relaxed?' If this happens, of course it is not relaxed as the fact of enquiring ... involves our critical faculties and so of necessity prevents relaxation ... It is only when our meditative experience comes to an end, and we have again become aware of the realities around us, that we know that we have just emerged from a state of mental relaxation." – p. 24<br>Meares p. 14 makes similar comments.<br>"Any awareness of our state of mind at the time of meditation means that we are not deep as we are obviously functioning at a critical level. But afterwards we can be aware that we have been oblivious of our surroundings and experiencing stillness of mind." – p. 49<br>"The meditator experiences regression as the loss of logical thinking, dulling of perception and the loss of awareness of his immediate surroundings. But the knowledge that he has had this experience only comes after he has made the counter-regression back to normal thinking." – p. 141 "... This is obvious, as we cannot examine our state of mind logically at a time when our logical thinking is in abeyance. It is in retrospect that we realise our mind had not been thinking about anything very much. We heard some noises, but they did not seem to have any particular significance, and for the time being we had really forgotten where we were. When ideas such as these come to us, we know that we have emerged from a state of regression." – pp. 141-142
Meares (1973b)	"When we practise [Stillness Meditation], the mind empties itself and we experience only a sensation of extreme calm; or we may feel very little at all, and it is only in retrospect that we are aware of the calm we have experienced." – p. 254

Meares (1989)	"If we come to think that our mind is still, it is not still. This is obvious because we are thinking. It is only in retrospect that we come to know that our mind has been still. When we have finished, we suddenly realise that we were not thinking about anything. When our mind is still, there is nothing. Just quietness. Just *being*." – p. 115
Meares (1976/1980)	"If we wonder how we are going, we are not doing it properly. It is only afterwards that we know if the experience was complete." – p. 54<br>"[In Stillness Meditation] we are unaware of being anxious or otherwise. If we examine ourselves, our state of mind changes, and we temporarily cease to be in [the stillness]. But in the normal course of events, as we return to full wakeful alertness, we are aware that we are less anxious, and that the level of our nervous tension is much reduced as a result of the experience." – p. 190
Meares (1988)	"... [I]f the patient thinks his mind is still, he is not achieving the desired result, because his awareness means that his mind is *not* still. The awareness of stillness of the mind should only be apparent at the end of meditation, and in retrospect." – p. 83
Meares (1976b)	"Of course we can only tell that our mind has been still after it has happened. We obviously cannot be aware that our mind is still at the time, as such awareness involves mental activity." – p. 909
Meares (1979d)	"There is a profound sense of stillness. But the meditator is only aware of this in the moments of lesser stillness – when the meditation is profound the process of awareness has ceased and the meditator is no longer aware of his mental state." – p. 120
Meares (1982c)	"Of course, if the patient feels that his mind is still, he is not achieving the desired goal, as his very awareness means that his mind is not still. The awareness that the mind has been still only comes when the meditation has ended." – p. 1607
Meares (1983a)	"... [I]f the patient thinks that his mind is still, he is deluding himself, as the process of thinking necessitates activity of the mind. It is only afterwards that the meditator becomes aware of the stillness. When the meditative experience has come to an end, the meditator is then aware in retrospect that his mind has been still." – p. 115
McKinnon (1983/2016)	"Very often the general experience of stillness is not available to us until we leave it and return to our usual state of alertness. If we then pause to look back, we will sense quiescence and repose ..." – p. 211
McKinnon (2011)	"The essence of the experience is largely discovered in retrospect." – p. 100

2.3.2 Conscious

Meares (1978/1986)	"The beginner sometimes feels that, if the mind is still and empty of thought, the individual must be either asleep or unconscious. This is not so. There is this other state ... The person who is unconscious has no awareness at all ... In the mental relaxation of [Stillness Meditation], we experience the stillness of it. The experiencing is a very simple ... function of the mind which does not involve clear consciousness as in the case of awareness." – p. 22<br>Quoting a patient: "It was terrific ... still conscious, but completely relaxed." – p. 25<br>"... [I]n [Stillness Meditation] we are neither asleep nor unconscious." – p. 151<br>"In the experience beyond relaxation we are not asleep, we are not unconscious ..." – p. 153
Meares (1984)	"... Not asleep<br>Not fully conscious ..." – p. 18

2.3.3 Awake, wakefulness, clearness, purity

Meares (1967/1968)	"[In Stillness Meditation] the mind is ... fully awake." – p. 59
Meares (1978/1986)	"The relaxation of our mind is not due to drowsiness ... [In Stillness Meditation] our mind is profoundly relaxed, but not drowsy, not sleepy, not asleep ..." – p. 22<br>"The beginner sometimes feels that, if the mind is still and empty of thought, the individual must be either asleep or unconscious. This is not so." – p. 22

	"The natural calm within is to be distinguished from the calm of drowsiness. It is the ease of the smooth functioning of our body and our mind." – p. 26<br>"... [T]he experience is ... intense and real." – p. 27<br>"... [I]n [Stillness Meditation] we are neither asleep nor unconscious ... We are awake, but calm, and defenceless." – p. 151<br>"In the experience beyond relaxation we are not asleep, we are not unconscious ..." – p. 153
Meares (1989)	"An effortless stillness in which we are not asleep, not unconscious, or drowsy ..." – p. 109
Meares (1987/1991)	"This is not the tranquility of drowsy somnolence. The mind is clear but still." – p. 114<br>"The final experience is simply being, in which we are neither asleep nor are we fully awake." – p. 127
Meares (1967b)	"[The patient] is not asleep, nor is he fully awake." – p. 45
Meares (1979d)	"The individual is not asleep, not stuporose, but simply experiencing his essential being." – p. 120
Meares (1983a)	"... crystal clear stillness ..." – p. 118
Meares (1977b)	"... And in the stillness,<br>The clarity ..." – p. 75
Meares (1984)	"... Not asleep<br>Not fully conscious<br>Mind is clear ..." – p. 18
Meares (1976/1984)	"... It is clear,<br>And the clarity of it is part of us ..." – p. 54<br><br>"... Only the calm and the stillness.<br>The purity beyond words ..." – p. 55
Meares (1987a)	"Ease is not drowsy ...<br><br>Drowsiness,<br>Like a mountain mist,<br>Blankets the clarity of what we see.<br>In ease it's all so clear ..." – p. 34
McKinnon (2011)	"The experience of stillness is one that is calm and clear and quite alert – but very still." – p. 67<br>"... Meares likens his idea of stillness to simple naturalness ... He is describing an experience that is untarnished by the addition of anything more – a pure experience of life." – p. 72<br>"*... pure stillness ...*" – p. 85<br>"... untainted simplicity ..." – p. 139
McKinnon (2002/2008)	"... calm, clear relaxation of the mind where the meditator is entirely still." – p. 35

2.3.4 Non-duality

See also 1.6.2 (Meares, 1978/1986 presentation of the practice).

Meares (1978/1986)	"More than our body relaxes. Now it is our whole self that is relaxed, our ego, our total being ... [W]e, ourselves, are participating in it. It is no longer a matter of our muscles or our body being relaxed because the distinction between our body and ourself has faded. It is all one. The relaxation becomes a total experience which involves our whole being." – pp. 19-20 "Experiencing our physical relaxation is more profound than being aware of it or feeling it. Being aware of our relaxation involves two entities. There is ourself, or our ego, which is aware, and then there is the relaxation of which we are aware. There are the same two entities when we feel our relaxation. Ourself, which does the feeling, and the thing we feel ... It would be easy to say that the same must apply when we come to experiencing; we who experience and the thing experienced. This is where my use of words fails. The idea that I am trying to communicate is that this duality is transcended in this kind of experience ... The separate entities cease to be separate. There is now only one. The experience, ourself and the relaxation are all one." – p. 20

2.3.5 Stillness

Meares (1978/1986)	"... [Stillness Meditation allows] the mind to come to a state of quiet and stillness." – p. ix<br>"... stillness come[s] to our mind ..." – p. x<br>"... stillness comes to us." – p. 6<br>In the complete experience of Stillness Meditation, the mind is "quite still" (p. 22).<br>"[Stillness Meditation] allows us to experience the essential stillness of mind." – p. 48<br>"A fifty-year-old married woman had complained that she had been very agitated ever since she could remember. Later when speaking of [Stillness Meditation] she said, 'Getting stillness with it ... So still, so free'." – p. 149<br>"... [Stillness Meditation] is a simple pathway to the stillness that lies deep within each one of us." – p. 160<br>"... that stillness where anxiety is gone ..." – p. 160
Meares (1989)	"[Stillness Meditation] ... is characterised by a very natural, calm, easy, effortless, stillness of mind." – p. 108<br>"When our mind is still, there is nothing. Just quietness. Just *being*." – p. 115
Meares (1988)	"[Stillness Meditation] is characterised by a great simplicity, a deep naturalness. It is a profound stillness of the mind." – p. 83
Meares (1977c)	"[Stillness Meditation] is characterized by stillness. There is an absence of intellectual activity, an absence of sensory experience and an absence of emotion ... The subject may be aware of his existential being but little else." – p. 131
Meares (1979d)	"There is a profound sense of stillness." – p. 120
Meares (1982c)	"[Stillness Meditation] is characterized by a great simplicity, a deep naturalness. It is just a profound stillness of the mind." – p. 1607
Meares (1983a)	"[Stillness Meditation] is characterized by simplicity, a very profound simplicity; and by naturalness, a very easy naturalness. The mind is simply allowed to be still." – p. 115<br>"... crystal clear stillness ..." – p. 118<br>"When the patient first sits down to meditate, he may experience feelings of calm, ease, naturalness and the feeling that it is good. These feelings pass as the stillness comes. They may recur from time to time, only to pass off spontaneously ..." – p. 119
Meares (1976/1984)	"It feels good,<br>It feels calm,<br>Even sublime.<br>What then do we say,<br>When we go beyond these things?<br>Still, without numbness.<br>Call it the foretaste<br>Of feeling beyond feeling." – p. 32<br><br>"Oblivion?<br>Oblivion that goes into nothingness.<br>Far from it.<br>... [I]n the stillness we are there,<br>In the depth and the height<br>And all that is around us.<br>In the essence of it." – p. 35
Meares (1987a)	"...With the mind effortless and still,<br>In a state of complete tranquility ..." – p. 50
McKinnon (1983/2016)	"... a velvety stillness ..." – p. 195<br>"... *profound experience of being still*." – p. 205

	"In time, the activity of the mind *will* diminish. Intermittently, moments of calm will occur. At another time, or perhaps on another day, more calm descends and thoughts are less intrusive, until eventually in exquisite stillness, nothing disturbs us at all." – p. 218<br>"The whole self is very still ..." – p. 221<br>"... silence and stillness ..." – p. 228
McKinnon (2011)	"... the experience of only stillness ... an all-encompassing silence." – p. 44<br>"... very deep and far reaching stillness" – p. 44<br>"... [L]ittle by little ... mental activity slows down and gradually, gradually, by doing nothing, stillness effortlessly follows." – pp. 76-77<br>"... an ever-pervading stillness and the calm within it." – p. 80<br>"... *pure stillness* ..." – p. 85<br>"The stillness will eventually be experienced globally, free of disturbance of any kind ..." – p. 84<br>"The mind is rarely blank, but it becomes still and undisturbed, with an ebb and flow of random and usually irrelevant thought in the background." – p. 100
McKinnon (1991)	"... a velvety 'nothingness'." – p. 73<br>"The aim of [Stillness Meditation] is to *bring oneself to a state of complete stillness, mind and body, wherein deep relaxation is achieved together with a time of rest for the mind* ..." – p. 74<br>"[Stillness Meditation] is directed towards stillness only." – p. 74
McKinnon (2002/2008)	"... profound stillness." – p. 14<br>"... entire physical and mental stillness." – p. 28<br>"The ultimate sensation of stillness is a state of complete security and ease, where body and mind are unified in a calm sense of 'being' – beyond effort ... of any kind." – p. 45<br>"... completely still and silent ..." – p. 52<br>"... stillness of body and stillness of mind." – p. 53

2.3.6 Silence, quietness

Meares (1978/1986)	"... [Stillness Meditation allows] the mind to come to a state of quiet and stillness." – p. ix<br>Meares coined the term "Mental Ataraxis" to refer to the meditation, and to distinguish it from other forms of meditation (pp. xi-xii, 6). He explains: "[The Greek term] '*ataraxis*' simply means 'an absence of disturbance'. Mental Ataraxis concerns quiet of mind and peace of mind." – p. xii Meares later stopped using the term on the basis that it was confusing for some people (Meares, 1989, pp. 108-109).<br>"[The experience] comes as quietness, and an ease, pervading everything – our thoughts, our feelings, our whole being." – pp. 24-25
Meares (1989)	"... [O]ur mind goes quiet of itself ..." – p. 46<br>"Nothing to disturb the quiet within." – p. 122<br>"When our mind is still, there is nothing. Just quietness. Just *being*." – p. 115
Meares (1987/1991)	"Let the mind run quiet. Just quiet." – p. 17<br>Meares pp. 21, 80, 115 makes similar comments.<br>"... just quietness, a stillness of effortless tranquility." – p. 114
Meares (1988)	"The idea is just to let our mind be quiet ... It is just a quietness, a stillness of the mind. Thoughts may come ... Just let them be, and in a few moments they fizzle out. A silence comes to our mind. Then a few more thoughts. Then silence again. It is just a coming and going." – pp. 73-74
Meares (1979b)	Writing from the perspective of a notional patient in conversation with himself as Stillness Meditation teacher/therapist:<br><br>"You harp on the silence<br>And the stillness ..." – p. 79
Meares (1984)	"... The silent stillness<br>Of the meditation ..." – p. 46
Meares (1987a)	"Ease is quietness of mind ..." – p. 25

	"... The storm whips up the waves,<br>But deeper down the world is quiet." – p. 42
McKinnon (1983/2016)	"... silence and stillness ..." – p. 228
McKinnon (2011)	"... the experience of only stillness ... an all-encompassing silence." – p. 44<br>"... quiet and silence ..." – p. 162
McKinnon (1991)	"*Only complete quiet* ..." – p. 127
McKinnon (2002/2008)	"... completely still and silent ..." – p. 52

2.3.7 Depth/height/fullness

Meares (1978/1986)	"The experience is both deep and high ..." – p. 27
Meares (1981b)	"... A stillness –<br>The nothingness<br>Brimful of meaning." – p. 59
Meares (1976/1984)	"Oblivion?<br>Oblivion that goes into nothingness.<br>Far from it.<br>... [I]n the stillness we are there,<br>In the depth and the height<br>And all that is around us.<br>In the essence of it." – p. 35
McKinnon (2011)	Quoting a client, "Jennifer": "... I was, it seemed, embraced in a ... nothingness really – yet it felt like everything." – pp. 101-102

2.3.8 Relaxation

Meares (1967/1968)	"... profound mental relaxation ..." – p. 59
Meares (1978/1986)	"[Stillness Meditation] is much more than mental relaxation ..." – p. xi<br>"[Stillness Meditation enables access to] the relaxation of our mind and the inner experience which lies beyond." – p. 7<br>"... [In Stillness Meditation] our mind is profoundly relaxed ..." – p. 22<br>"We experience the relaxation of our body, and we experience the relaxation of our mind, but it is more, it is our whole being ... We come to experience the relaxation of our body and of our mind as the calm and ease of our whole being." – p. 27 "Body relaxed; mind relaxed; more than our body and our mind; it is in our whole being. We experience the completeness of the relaxation. It is in every aspect of ourself." – p. 31<br>"The ... relaxation is not simply of our body, and not simply of our mind, but is of our whole being." – p. 31<br>"Once we have learned to relax our mind [by experiencing the relaxation of the body] we no longer need to concern ourselves about experiencing the relaxation of the body ... Once our mind has learned how to do it, there is no need for us to repeat these preliminary steps each time. We now know what we want, and our mind has learned how to attain it. When we want to relax our mind, the relaxation of it is just there, quite naturally ..." – p. 31<br>"We are relaxed, but we experience something far beyond relaxation. It is our inner ease. The calm that is natural to us. ..." – p. 149
Meares (1970)	"You just let yourself be, and the relaxation is there because it is natural to you." – p. 67
Meares (1976b)	"As we come to experience our relaxation it is not so much that our body is relaxed, but rather that we are relaxed. Our whole self. More than our body and our mind. Our whole being." – p. 909
McKinnon (2011)	"... experience of profound physical and mental relaxation ..." – p. 238

McKinnon (1991)	"The aim of [Stillness Meditation] is to *bring oneself to a state of complete stillness, mind and body, wherein deep relaxation is achieved together with a time of rest for the mind* ..." – p. 74<br>"... [T]he whole self is very still – just *being* – in a state of peace even beyond relaxation." – p. 77<br>"[Stillness Meditation] ... is far more than relaxation." – p. 96<br>"We all have memories of a momentary daydream (or reverie) when our mind is temporarily free of constructive thought ... That is the closest description of mental relaxation according to [Stillness Meditation]." – p. 124

2.3.9 Mental rest

McKinnon (1983/2016)	"The mind is rested, not activated." – p. 190<br>"... [Stillness Meditation] concerns a natural experience of mental rest ..." – p. 205<br>"... [In Stillness Meditation] the mind, in fact the whole *being* ... is temporarily resting ..." – p. 207<br>"... mental rest – stillness ..." – p. 240<br>"... quintessential rest for the whole being." – p. 276
McKinnon (2011)	"... stillness of mind – or, in other words, true mental rest." – p. 67<br>"'What is [Stillness Meditation]?' 'Just natural rest for the mind.'" – p. 99<br>Quoting a client, "Elizabeth": "... an experience of complete physical and mental rest ..." – pp. 183-184<br>"The intention is ... to experience only inner calm where the mind is rested ..." – p. 226
McKinnon (1991)	"... [W]e gain *rest for the mind*: mental relaxation." – p. 8<br>"I think the meditative state could best be described as a sensation of immense security embodying a kind of 'knowledge' – or a kind of peace – that allays anxiety, together with an all-pervading restfulness." – pp. 73-74<br>"The aim of [Stillness Meditation] is to *bring oneself to a state of complete stillness, mind and body, wherein deep relaxation is achieved together with a time of rest for the mind* ..." – p. 74
McKinnon (2002/2008)	"... refreshing ..." – p. 45

2.3.10 Emptiness, nothingness, reduced mental activity

See also the sections below, in particular 2.3.11 ("No thoughts").

Meares (1978/1986)	Stillness Meditation involves a "progressive reduction in mental activity" (p. 6).
Meares (1973b)	"When we practise [Stillness Meditation], the mind empties itself and we experience only a sensation of extreme calm; or we may feel very little at all, and it is only in retrospect that we are aware of the calm we have experienced." – p. 254<br>"When you have been doing [Stillness Meditation] for a little while, you will probably find that you have very little mental activity at all." – p. 256
Meares (1989)	"When our mind is still, there is nothing. Just quietness. Just *being*." – p. 115
Meares (1987/1991)	"With a little practise, thoughts intrude less and less. Eventually there comes an emptiness of mind. A sense of being. Just pure being." – p. 127
Meares (1977c)	"[Stillness Meditation] is characterized by stillness. There is an absence of intellectual activity, an absence of sensory experience and an absence of emotion ... The subject may be aware of his existential being but little else." – p. 131
Meares (1983a)	"When the patient first sits down to meditate, he may experience feelings of calm, ease, naturalness and the feeling that it is good. These feelings pass as the stillness comes. They may recur from time to time, only to pass off spontaneously ..." – p. 119
Meares (1983b)	"... [T]here is little or no conscious mental activity." – p. 583
Meares (1976/1984)	"Oblivion?<br>Oblivion that goes into nothingness.<br>Far from it.<br>... [I]n the stillness we are there,

In the depth and the height
And all that is around us.
In the essence of it." – p. 35

McKinnon (1983/2016)
"In time, the activity of the mind *will* diminish. Intermittently, moments of calm will occur. At another time, or perhaps on another day, more calm descends and thoughts are less intrusive, until eventually in exquisite stillness, nothing disturbs us at all." – p. 218
"... an inner knowledge of being untroubled ..." – p. 268

McKinnon (2011)
"Stillness Meditation is a simple ... experience of ... just simply letting ... into a period of non-activity and effortless surrender." – p. 75
"... [L]ittle by little ... mental activity slows down and gradually, gradually, by doing nothing, stillness effortlessly follows." – pp. 76-77
"The mind is rarely blank, but it becomes still and undisturbed, with an ebb and flow of random and usually irrelevant thought in the background." – p. 100
"During stillness, there is ... no logic, no activity – just the calm ..." – p. 130

McKinnon (1991)
"... a velvety 'nothingness'." – p. 73

2.3.11 No thoughts

See also 2.3.10 ("Emptiness, nothingness, reduced mental activity") and 1.15.2 ("Dealing with thoughts").

Meares (1978/1986)
"... [T]here is little or no thought ..." – p. 6
In the complete experience of Stillness Meditation, the mind is "empty of thought" (p. 22).
"Thought ceases ..." – p. 25

Meares (1989)
"Thoughts come. Then there are little moments without thought. Then they come again. At the start it is just like that." – p. 46
"Just the natural stillness of it all. Without thought or feeling." – p. 109

Meares (1987/1991)
"With a little practise, thoughts intrude less and less. Eventually there comes an emptiness of the mind." – p. 127

Meares (1976/1980)
"In deep meditation there is an almost complete cessation of all thought ..." – p. 189

Meares (1988)
"Thoughts may come. Just let them be, and in a few moments they fizzle out. A silence comes to our mind. Then a few more thoughts. Then silence again. It is just a coming and going." – p. 74

Meares (1977c)
"[Stillness Meditation] is characterized by stillness. There is an absence of intellectual activity, an absence of sensory experience and an absence of emotion ... The subject may be aware of his existential being but little else." – p. 131

McKinnon (1983/2016)
"... [G]radually our rational thought becomes suspended ... Thoughts become disconnected and unimportant to us." – p. 211
"In time, the activity of the mind *will* diminish. Intermittently, moments of calm will occur. At another time, or perhaps on another day, more calm descends and thoughts are less intrusive, until eventually in exquisite stillness, nothing disturbs us at all." – p. 218
"Thoughts lose their logical or critical content and become barely distinguishable, washing over your consciousness like the ebb and flow of the tide ..." – p. 221
"With practice, thoughts will separate and recede, becoming more dream-like and less logical. The mind will never be completely 'blank' but between those separated thoughts, a resting mind will be experienced." – p. 269

McKinnon (2011)
"... [L]ogical activity ceases ... There is little or no thought ..." – p. 69
"Thoughts may flit in and out and around, but they are unarticulated." – p. 85
"The mind is rarely blank, but it becomes still and undisturbed, with an ebb and flow of random and usually irrelevant thought in the background." – p. 100
Quoting a client, "Jennifer": "Initially I knew I was thinking. But, as instructed, I paid no attention and the thoughts became more dreamlike than actual." – p. 101 Then, as she encounters the stillness: "... [T]here were really no consistent or logical thoughts – that is, I wasn't actually thinking ..." – p. 102

	"... letting go of attention, feeling and thought." – p. 109<br>"During stillness, there is ... no logic, no activity – just the calm ..." – p. 130<br>Quoting Francine Cockerill and Kaye Hakopian, teachers at McKinnon's Stillness Meditation Therapy Centre: "With practice ... thoughts become more like a daydream ..." – p. 164<br>"... while thoughts go free and melt in peace." – p. 203
McKinnon (1991)	"[G]radually ... ideas will fade, thoughts lose their logical content ..." – p. 77
McKinnon (2002/2008)	"Even within the deepest meditation, some thoughts will be present, though they will be somewhat dreamlike, less connected and of a less logical or 'normal' content'." – p. 45

2.3.12 No feelings

See also 2.3.19 ("Good/wonderful").

Meares (1978/1986)	"Thought ceases, we are beyond feeling, and our mind is relaxed like the rest of ourself." – p. 25
Meares (1973b)	"When we practise [Stillness Meditation], the mind empties itself and we experience only a sensation of extreme calm; or we may feel very little at all, and it is only in retrospect that we are aware of the calm we have experienced." – p. 254
Meares (1989)	"Just the natural stillness of it all. Without thought or feeling." – p. 109
Meares (1977c)	"[Stillness Meditation] is characterized by stillness. There is an absence of intellectual activity, an absence of sensory experience and an absence of emotion ... The subject may be aware of his existential being but little else." – p. 131
Meares (1983a)	"When the patient first sits down to meditate, he may experience feelings of calm, ease, naturalness and the feeling that it is good. These feelings pass as the stillness comes. They may recur from time to time, only to pass off spontaneously ..." – p. 119
Meares (1977b)	"... Beyond thinking<br>Beyond feeling ..." – p. 18
Meares (1979b)	"... How can you meditate<br>When you are feeling?<br>It's the experience<br>Of naturalness<br>Of such profound simplicity<br>It's beyond all feeling." – p. 83
Meares (1976/1984)	"Feel ourselves relax,<br>Feel the calm,<br>Feel the stillness,<br>Joy in it, ecstasy,<br>But it is only the path.<br>Flowers by the wayside,<br>Vistas between the trees,<br>It is beyond all this<br>Where we seek to go." – p. 12<br><br>"... Feel the calm.<br>Calm of our body, calm of our mind.<br>Calm of our whole self.<br>But more than the calm.<br>It too is the path,<br>The road we must find,<br>The way we must take.<br>That is the joy of the way,<br>Not the end that we seek." – p. 18

"It feels good,
It feels calm,
Even sublime.
What then do we say,
When we go beyond these things?
Still, without numbness.
Call it the foretaste
Of feeling beyond feeling." – p. 32

"Feel the calm.
Feel it all through us.
Feel it as part of us.

What good comes of feeling?

More than feeling.
Experience it ..." – p. 48

Meares (1987a) "... Ease is a pathway to the sublime ..." – p. 59

McKinnon (1983/2016) "You stay a while in this stillness. It's a good feeling." – p. 227
"One feels very little, apart from an inner knowledge of being untroubled, within an all-pervading comfort, certainty and the security of peace." – p. 268

McKinnon (2011) "... [F]eeling is contrary to pure stillness." – p. 85
"It is pure calm, beyond feeling or sensation." – p. 101
"... letting go of attention, feeling and thought." – p. 109

2.3.13 Reduced awareness/perception, loss of logical/critical faculties
See also 2.3.11 ("No thoughts") and 2.3.23 ("Transcendence of discomfort").

Meares (1967/1968) In the relaxed/regressed state "the critical faculties are largely in abeyance" (p. xv).
"[In regression] we are less alert and ... the critical faculties of our mind are less active." – p. 61
"The main features of [regression] are that we are less alert and less critical." – p. 82
"[In regression we are] no longer concerned with our immediate surroundings ..." – pp. 82-83 "We ... [allow] ourselves to neglect what is going on around us. We let ourselves lose awareness of the things in the room where we are. We temporarily abandon our critical faculties. If a truck passes in the street, we hear the noise, we don't think of it as being a heavily laden truck going past in the street; it is just a noise. This is what I mean by allowing ourselves to be uncritical." – p. 83
Meares pp. 88-89, 93 makes comments along the same lines to those in this row above.

Meares (1978/1986) Meares makes comments similar to those in Meares (1967/1968) above.

To highlight select points:
"First of all [the mind's] logical, critical activity is reduced until this finally ceases. Then the vividness of our perception is reduced, noises come to be heard as sounds with little or no meaning. As the process continues there is little or no thought, and little or no awareness of sounds or activity around us." – p. 6
Meares describes his experience meditating while floating in his swimming pool: "I [would] float ... in very cold water in winter in a very relaxed state so that I would no longer be conscious of the cold." – p. 10
"... [A]ll awareness of our breathing is lost." – p. 15
"Awareness of our breathing ... prevents the experience of the ... stillness ..." – p. 15
"In [Stillness Meditation], it is not so much that our mind does not perceive practical realities, but rather that it perceives [them] in such simple and harmonious fashion that the mundane aspect of practical reality is completely transcended." – p. 18
"I know of two busy housewives who can capture [the Stillness Meditation experience] very completely while sitting in the family circle looking at TV ... [A]s the [experience] comes to them the TV voices become blurred and meaningless and finally fade from consciousness." – p. 45
"If [meditating outside and] the wind is fierce and the cold chilling ... [a]s our [experience] becomes more complete ... our awareness of all this becomes less vivid until it finally fades. But it is still there, somehow influencing us even though it is not through our clear consciousness." – p. 45

	"In meditating outside, the wind becomes a distracting influence. It blows in our face and ruffles our hair, but we are undisturbed. Flies and insects may buzz about us and crawl on our face. We are only vaguely aware of them, insignificant in the depth of our awareness of the stillness within us." – p. 54<br>Meares speaks about meditating at the surf beach, near the water's edge: "Every now and then with a gust of wind or a larger wave I am suddenly showered with cold spray. I feel it, of course, but I am in no way disturbed. There is no reaction physically or emotionally, and the ease of mind continues." – p. 55<br>"Let us be quite clear about this. When we are practising we are, in fact, in a kind of trance because we are no longer aware of the things around us." – p. 63<br>"The meditator experiences regression as the loss of logical thinking, dulling of perception and the loss of awareness of his immediate surroundings. But the knowledge that he has had this experience only comes after he has made the counter-regression back to normal thinking." – p. 141 "It is in retrospect that we realise our mind had not been thinking about anything very much. We heard some noises, but they did not seem to have any particular significance, and for the time being we had really forgotten where we were. When ideas such as these come to us, we know that we have emerged from a state of regression." – pp. 141-142
Meares (1989)	"No awareness of breathing. Just a stillness of the mind." – p. 17<br>On meditating in summer sitting by a garden sprinkler: "A gust of wind would suddenly shower me with icy drops of water. But when my meditation was going well, it was not icy at all. Just a vague awareness of what was happening. Nothing to disturb the quiet within." – p. 122<br>"... [A]s long as we are aware of our breathing, or aware of anything else for that matter, our mind is not still." – p. 123
Meares (1987/1991)	"A stillness comes to our mind, and the noises around us recede further and further away." – p. 127
Meares (1969b)	"I have taught many patients ... how to go into a trance. They practice [Stillness Meditation] ..." – p. 77
Meares (1976/1980)	"In ... [Stillness Meditation] the individual becomes increasingly unaware of his immediate surroundings ..." – p. 189
Meares (1967a)	"... [E]astern mystics ... learn to experience pain in pure form without any psychological overlay at all. The pain is felt as a bare sensation, and as such, there is little hurt in it." – p. 117<br>Meares talks at length in several of his other texts about how Stillness Meditation also leads to an experience of pain with little or no hurt. This table extracts only select passages on this point that are most relevant to the meditator's awareness/perception in the meditation.
Meares (1967b)	"... [W]e temporarily cease to be alert and critical." – p. 44<br>Meares p. 46 makes similar comments.
Meares (1967d)	Stillness Meditation enables the experience of "pain in pure form" (p. 11). "By this I mean pain without any psychological overlay at all. Strange as it may seem, pain experienced in this way loses its hurt." – p. 11
Meares (1968b)	"The pure sensation of pain, in the complete absence of any psychological embellishment, and in the absence of any alert preoccupation with the [pain], loses its hurt." – p. 56
Meares (1971a)	"[In Stillness Meditation sessions that I conduct, the session] is terminated by my telling the group to open their eyes and rest quietly." – p. 676
Meares (1973a)	"[At the end of the Stillness Meditation session] I waken [the meditators] by announcing in a loud voice, 'That's good. Just let your eyes open now, and rest quietly for a moment'. After a few moments I give a little talk for 10 minutes ... I talk sharply and incisively so as to aid the counter-regression to fully alert functioning." – p. 734
Meares (1977c)	"[Stillness Meditation] is characterized by stillness. There is an absence of intellectual activity, an absence of sensory experience and an absence of emotion ... The subject may be aware of his existential being but little else." – p. 131
Meares (1978c)	Meares pp. 85-87 makes clear that Stillness Meditation does not involve "vivid visualization", meaning visualization as it is normally referred to in a meditation context. However, he indicates that regression (as in Stillness Meditation) could be seen to involve "dim visual awareness" (pp. 87-88). Referring to this, he

	says: "... [T]here is another type of visualization which is consistent with regression to simple functioning ... [I]t is possible to perceive objects in a way that is something less than a clear visual experience, and at the same time is distinct from simply thinking of the object ... It is [the vague] day dream quality that I term dim visual awareness. The mind is no longer controller or alert ..." – p. 87
Meares (1979a)	Referring to the use of touch in Stillness Meditation sessions conducted by Meares: "... [W]e do not want the patient to be asleep or in a stupor so that he is unaware of our touch ... What we do want is for the patient to be aware of our touch, but only dimly aware, as something which is part of all that is going on around him. He lets himself go along with it so that he experiences the essential naturalness of it all, to the very core of his being." – p. 122<br>"... [T]he regression of mental functioning [in Stillness Meditation] is shown by the loss of alert critical thinking, the dulling of discomfort and pain, the absence of reaction to external stimuli, and the vague perception of things in their fundamental simplicity." – p. 123
Meares (1976/1984)	"Drowsy?<br>How could we achieve such a thing,<br>If we were less than alert?<br>If we are alert we have not got it,<br>Of that there is no doubt whatsoever." – p. 38
McKinnon (1983/2016)	"In moments we may become aware of *un*awareness – and then of course we are aware again until that, too, drifts away." – p. 211<br>"[A]s the mind becomes still, our physical sensations, discomforts, distractions and so on, gradually disappear. In the stillness, we are learning to let go beyond ... awareness of any kind." – pp. 212-213<br>"Noises, discomfort and other distractions fade into the background to assume unimportance." – p. 221<br>"You are distantly aware of quiet words of reassurance [from the teacher]. You let that awareness go, and the words become more and more distant, less frequent, until they too disappear and there is stillness." – p. 227
McKinnon (2011)	"... [V]ibrancy of perception fades, the distraction of background noises are heard as meaningless sounds in the distance of no importance ... There is ... little or no awareness of anything surrounding the meditator." – p. 69<br>"Nor does [Stillness Meditation] have anything to do with hypnotic trances ..." – p. 74<br>"Now your whole being is ... gradually becoming a unified being of stillness. You can sense it – without awareness, you know it somehow – and you experience it deeply ..." – p. 81<br>"In learning stillness, the teacher assists the meditator to let go beyond the present. " – p. 93 "The teacher allows the meditator to [let go] of background sounds, by not paying attention: sounds are not important right now. Neither is awareness: of the body, the environment or the senses." – pp. 93-94 "Especially not important are distractions or discomforts ..." – p. 94<br>"It is pure calm, beyond feeling or sensation. There is no awareness either – it is beyond awareness ..." – p. 101<br>Quoting a client, "Amy": "... beyond body ..." – p. 179<br>Referring to classes with Meares' at his premises near Parliament Station: "The City of Melbourne is building Parliament Station at that time. The pneumatic drilling fades to a whisper as sounds lessen within the stillness." – p. 199<br>"... [N]o colour, light or sound disturbs." – p. 203
McKinnon (1991)	"... [A]wareness of our surroundings becomes unimportant. Noises and distractions are transcended and the whole self is very still." – p. 77

2.3.14 No mental images

See also 2.3.10 ("Emptiness, nothingness, reduced mental activity").

Meares (1978/1986)	"It is quite common [in the interim-states] to experience various visual phenomena ... I emphasise that these phenomena cease when we let ourselves return to the experience of our own inner ease." – p. 46
McKinnon (2011)	Quoting a client, "Jennifer": "At one stage [in the interim-states], a variety of colours and images moved in and out of my mental view. I had been advised to ignore these, letting them go and soon they passed ... Towards the conclusion of the session there was no visual image at all." – p. 101

	"Images, colours, shapes and patterns are commonly experienced [in the interim-states] by beginners. I hope this won't disappoint you, but it most likely will never happen again!" – p. 110<br>"[In the interim-states] [s]ome may witness inexplicable phenomena such as ... images ... or visual sensations ... The advice is always to pay no attention to these and they quickly pass." – p. 115

2.3.15 No memories
See also 2.3.10 ("Emptiness, nothingness, reduced mental activity").

McKinnon (1983/2016)	"... inwardly removed from distracting sounds, thoughts or memories." – p. 195

2.3.16 Ebb and flow
See also 2.3.17 ("Increasing length")

Meares (1989)	"Thoughts come. Then there are little moments without thought. Then they come again. At the start it is just like that." – p. 46
Meares (1987/1991)	"At first, until the meditator has learned [Stillness Meditation] there will be moments of stillness, but these are soon interrupted by the intrusion of thoughts. Do not try to dispel the thoughts by actively driving them from the mind. Just let them be, and they will fizzle out, cease, and stillness will come again. Then thoughts will recur. And again, if they are let alone, stillness will supervene. And gradually the space between the thoughts, the stillness that we want, will become longer and longer." – p. 114
Meares (1988)	"Thoughts may come. Just let them be, and in a few moments they fizzle out. A silence comes to our mind. Then a few more thoughts. Then silence again. It is just a coming and going." – p. 74
Meares (1983a)	"When the patient first sits down to meditate, he may experience feelings of calm, ease, naturalness and the feeling that it is good. These feelings pass as the stillness comes. They may recur from time to time, only to pass off spontaneously ..." – p. 119
McKinnon (1983/2016)	"In moments we may become aware of *un*awareness – and then of course we are aware again until that, too, drifts away." – p. 211<br>"In time, the activity of the mind *will* diminish. Intermittently, moments of calm will occur. At another time, or perhaps on another day, more calm descends and thoughts are less intrusive, until eventually in exquisite stillness, nothing disturbs us at all." – p. 218<br>"Thoughts lose their logical or critical content and become barely distinguishable, washing over your consciousness like the ebb and flow of the tide ..." – p. 221<br>"With practice, thoughts will separate and recede, becoming more dream-like and less logical. The mind will never be completely 'blank' but between those separated thoughts, a resting mind will be experienced." – p. 269
McKinnon (2011)	"Thoughts may flit in and out and around, but they are unarticulated." – p. 85<br>"The mind is rarely blank, but it becomes still and undisturbed, with an ebb and flow of random and usually irrelevant thought in the background." – p. 100<br>Quoting a client, "Jennifer": "Initially I knew I was thinking. But, as instructed, I paid no attention and the thoughts became more dreamlike than actual." – p. 101 Then, as she encounters the stillness: "... [T]here were really no consistent or logical thoughts – that is, I wasn't actually thinking ..." – p. 102
McKinnon (2002/2008)	"Even within the deepest meditation, some thoughts will be present, though they will be somewhat dreamlike, less connected and of a less logical or 'normal' content'." – p. 45

2.3.17 Increasing length
See also 2.3.16 ("Ebb and flow")

Meares (1978/1986)	"At first the relaxation of our mind waxes and wanes ... There are moments of completeness and moments in which the experience is less complete. Moments of stillness may be interrupted by moments of full alertness ... With practice the phases of stillness become longer and longer ..." – p. 25
Meares (1989)	"The stillness comes and goes as an ebb and flow, with longer and longer periods of stillness as we become more experienced." – p. 17

	"The stillness is never complete for long periods. There is always a coming and going, an ebb and flow, a little moment of stillness and then more thoughts again." – pp. 114-115
Meares (1987/1991)	"At first, until the meditator has learned [Stillness Meditation] there will be moments of stillness, but these are soon interrupted by the intrusion of thoughts ... [As we progress] gradually the space between the thoughts, the stillness that we want, will become longer and longer." – p. 114
Meares (1976b)	"Stillness comes. Not all at once. At first momentarily and fleetingly. Then for longer periods." – p. 909
McKinnon (2011)	"... [L]ittle by little, one will gradually experience stillness of mind ..." – p. 67<br>Quoting Francine Cockerill and Kaye Hakopian, teachers at McKinnon's Stillness Meditation Therapy Centre: "In the early stages of learning, quietness of the mind is only glimpsed. With practice ... retrospectively, extended stillness of mind can be recalled." – p. 164

2.3.18 Calm, ease, peacefulness, absence of disturbance

For the idea that, by relinquishing or moving beyond awareness, the meditator's environment, and discomforts, no longer disturb them, see also 2.3.13 ("Reduced awareness/perception, loss of logical/critical faculties") and 2.3.23 ("Transcendence of discomfort").

Meares (1967/1968)	"... [In Stillness Meditation] the mind is deeply calm and at ease ..." – p. 59<br>"In moments of ... complete mental relaxation our mind fills with calm ..." – p. 61<br>"... calm and ease of mind." – p. 62
Meares (1978/1986)	"... [Stillness Meditation] brings us calm and ease of mind ..." – p. vii<br>"By [Stillness Meditation] we can let calm ... come to our mind ..." – p. x<br>Meares coined the term "Mental Ataraxis" to refer to the meditation, and to distinguish it from other forms of meditation (pp. xi-xii, 6). He explains: "[The Greek term] '*ataraxis*' simply means 'an absence of disturbance'. Mental Ataraxis concerns quiet of mind and peace of mind." – p. xii Meares later stopped using the term on the basis that it was confusing for some people (Meares, 1989, pp. 108-109).<br>"There is ease ... in this letting go." – p. 25<br>"We let ourselves experience the essential calm of our being ... We are aware of the natural calm deep within us. We let ourselves experience it." – p. 25<br>"The experience of calm is felt differently by people: [quoting patients] '...a calm without trying.' 'It was terrific ... still conscious, but completely relaxed.' 'A calm of mind I've never felt before.' 'It was like *finding* some sort of calm ... some pool of calm.' '...a calm that has remained with me.' 'Calm within myself.' 'I feel so much calmer, it's incredible!'" – p. 25<br>"The natural calm within is ... the ease of the smooth functioning of our body and our mind." – p. 26<br>"We come to experience the relaxation of our body and of our mind as the calm and ease of our whole being." – p. 27<br>"Conscious only of ease ... The stillness of our thought is ease. So is the calm of our mind and all feeling gives way to ease." – p. 31<br>"... [W]e have the experience of our own inner ease." – p. 146<br>"We are relaxed, but we experience something far beyond relaxation. It is our inner ease. The calm that is natural to us." – p. 149<br>"... [I]n the experience of meditation, [our normal] background of anxiety fades. There is a calm which we do not experience in ordinary life situations ..." – p. 151<br>"We are awake, but calm, and defenceless." – p. 151<br>"As our mind relaxes we may come to experience various subjective phenomena. Other than the experience of very great calm and ease, subjective phenomena are not a part of [Stillness Meditation]." – p. 156<br>"Calm comes to us ..." – p. 161
Meares (1973b)	"... the inner calm which is natural to us." – p. 177<br>"A man as he was going [from a Stillness Meditation session] said that he had never before experienced such a depth of calm." – p. 254<br>"When we practise [Stillness Meditation], the mind empties itself and we experience only a sensation of extreme calm; or we may feel very little at all, and it is only in retrospect that we are aware of the calm we have experienced." – p. 254
Meares (1989)	"[Stillness Meditation] is characterised by a very natural, calm, easy, effortless, stillness of mind." – p. 108<br>"We aim for ease. A very profound ease." – p. 109<br>"Nothing to disturb the quiet within." – p. 122

Meares (1987/1991)	"... [O]ur brain runs easy ..." – p. 23<br>"Let our mind run easy." – p. 25<br>"We are not asleep. Just tranquil. Profoundly tranquil." – p. 80<br>"... a stillness of effortless tranquility." – p. 114<br>"... letting ourselves experience the inner ease which is a natural part of us." – p. 124
Meares (1978b)	"The natural ease of our own self ..." – p. 6
Meares (1983a)	"When the patient first sits down to meditate, he may experience feelings of calm, ease, naturalness and the feeling that it is good. These feelings pass as the stillness comes. They may recur from time to time, only to pass off spontaneously ..." – p. 119
Meares (1983b)	With reference to patients with cancer practising intensive Stillness Meditation: "Through intensive meditation, the patient experiences a profound sense of calm ..." – p. 583
Meares (1976/1984)	"... Feel the calm.<br>Calm of our body, calm of our mind.<br>Calm of our whole self.<br>But more than the calm.<br>It too is the path,<br>The road we must find,<br>The way we must take.<br>That is the joy of the way,<br>Not the end that we seek." – p. 18<br><br>"It feels good,<br>It feels calm,<br>Even sublime.<br>What then do we say,<br>When we go beyond these things?<br>Still, without numbness.<br>Call it the foretaste<br>Of feeling beyond feeling." – p. 32<br><br>"Being profoundly calm<br>Is being profoundly calm.<br>It is not being.<br>When being comes we are beyond calm.<br>How could you be calm,<br>If you were simply being?" – p. 36<br><br>"Feel the calm.<br>Feel it all through us.<br>Feel it as part of us.<br><br>What good comes of feeling?<br><br>More than feeling.<br>Experience it ..." – p. 48
Meares (1987a)	"Ease is quietness of mind ..." – p. 25<br><br>"... With the mind effortless and still,<br>In a state of complete tranquility ..." – p. 50
McKinnon (1983/2016)	"... ever-deepening calm." – p. 211<br>"... quiescence and repose ..." – p. 211<br>"In time, the activity of the mind *will* diminish. Intermittently, moments of calm will occur. At another time, or perhaps on another day, more calm descends and thoughts are less intrusive, until eventually in exquisite stillness, nothing disturbs us at all." – p. 218

	"... [J]ust *experience* the ease of being effortlessly still." – p. 221<br>"... [Y]ou sense the peace." – p. 228
McKinnon (2011)	"... [W]e simply ... experience [the calm] in complete stillness, effortlessly." – p. 40<br>"... an ever-pervading stillness and the calm within it ..." – p. 80<br>"The stillness will eventually be experienced globally, free of disturbance of any kind ..." – p. 84<br>"It is pure calm ..." – p. 101<br>"During stillness, there is ... no logic, no activity – just the calm ..." – p. 130<br>"It is in untainted simplicity ... that calm can be accessed." – p. 139<br>"The mind is rarely blank, but it becomes still and undisturbed, with an ebb and flow of random and usually irrelevant thought in the background." – p. 100<br>Quoting a client, "Jenny": "So calming. A wonderful calmness ..." – p. 168<br>Quoting a client, "Cathy": "I have experienced inner calm and inner strength ..." – p. 173<br>Quoting a client, "Amy": "... I was making contact with the peace and ease of being that I'd forgotten." – p. 179<br>"It must be a little taste of heaven, this precious calm ..." – p. 203<br>"Deep peace, secure, at one with all ..." – p. 203<br>Quoting a client, "Alan": "The peace and calm I experience ... has been ... positively life changing." – p. 219<br>"The intention is ... to experience only inner calm where the mind is rested ..." – p. 226
McKinnon (1991)	"I think the meditative state could best be described as a sensation of immense security embodying a kind of 'knowledge' – or a kind of peace – that allays anxiety, together with an all-pervading restfulness." – pp. 73-74 "Calm inner peace, largely a retrospective experience, is the product of stillness." – p. 74<br>"The [young] children I have worked with ... have afterwards volunteered such remarks as, '... it's really peaceful, very easy ...'." – p. 129
McKinnon (2002/2008)	"The ultimate sensation of stillness is a state of complete security and ease, where body and mind are unified in a calm sense of 'being' – beyond effort ... of any kind." – p. 45<br>"After the experience has concluded, the meditator can usually reflect on having experienced a state of serenity and security ... or contentment. He or she may describe the experience as 'relaxing, refreshing, lovely, peaceful and calm'." – p. 45

2.3.19 Good/wonderful

See also 2.3.12 ("No feelings")

Meares (1978/1986)	"The effortlessness of [Stillness Meditation] is experienced as something quite wonderful." – p. 23<br>Quoting a patient: "It was terrific ... still conscious, but completely relaxed." – p. 25<br>"When [the ease] comes to us it is really an experience of the highest order. Something that far transcends the ordinary experience of life as most of us know it. People ... have often said to me, 'That was wonderful, truly wonderful'." – p. 26<br>"Do not expect ecstasy ... Expect rather the experience of deep naturalness. Utter naturalness ... Simplicity. Such profound simplicity ..." – p. 145<br>"As [the experience of ease] comes to us, we can let our mind go further so that the experiencing of it becomes something quite wonderful." – p. 146<br>"As we let the experience ... come to us we feel that it is good ... The evidence for this is that many people make comments to this effect without my having suggested anything to them. We experience it as good. But it has become clear to me that this general feeling as a variety of different aspects." – p. 155<br>Meares goes on to discuss some of these aspects. "The experience ... feels good because of our reduced level of anxiety. It feels good, we feel good. But this experience often transcends the awareness of good health and ease of mind. It is good to feel good in the sense that feeling good is our natural biological state, and when we feel good in this way, it is good because we have attained our full biological potential in this area." – p. 155. "In [Stillness Meditation] we may experience a sense of good that lies deep within ourselves. This is something difficult to describe, but to some people it is very real. In [Stillness Meditation], with the absence of anxiety and the suspension of our psychological protective reactions, we gain a glimpse into our inner being – something which is otherwise closed to us. We experience something of ourselves which we had not previously experienced! We experience something very good – an awareness of a source of good within us ... This is exactly how some people feel it and of course this is true; true in the sense of material reality, for we all do have a capacity for good. It is just that in [Stillness Meditation] we come face to face, as it were, with this natural capacity for good which is within us." – p. 155

Meares (1969c)	"The whole process is quite effortless. And because of this it feels good." – p. 84
Meares (1976/1980)	"We can gain pleasure from the relief of anxiety ... Many people [to whom I have taught Stillness Meditation] have spoken to me of the wonderful feeling which has come to them following the relief of severe chronic anxiety ... [I]t is a positive pleasure of a high order." – p. 134
Meares (1983a)	"When the patient first sits down to meditate, he may experience feelings of calm, ease, naturalness and the feeling that it is good. These feelings pass as the stillness comes. They may recur from time to time, only to pass off spontaneously ..." – p. 119
Meares (1979b)	"... Joy?<br>Where do we feel it?<br>Neither heart nor mind.<br>It's in our being<br>Where we meditate." – p. 62
Meares (1976/1984)	"Feel ourselves relax,<br>Feel the calm,<br>Feel the stillness,<br>Joy in it, ecstasy,<br>But it is only the path.<br>Flowers by the wayside,<br>Vistas between the trees,<br>It is beyond all this<br>Where we seek to go." – p. 12<br><br>"It feels good,<br>It feels calm,<br>Even sublime.<br>What then do we say,<br>When we go beyond these things?<br>Still, without numbness.<br>Call it the foretaste<br>Of feeling beyond feeling." – p. 32
Meares (1987a)	"... Ease is a pathway to the sublime ..." – p. 59
McKinnon (1983/2016)	"You stay a while in this stillness. It's a good feeling. You let go into it more and more and more completely, and you sense something wonderful, a temporary journey into something new." – p. 227-228<br>"What do I feel when meditating? At a much later date ... a profound sense of ease and well-being is experienced." – p. 268
McKinnon (2011)	"Only calmness prevails ... producing ease and wellbeing ..." – p. 84<br>"... [T]he quality [of the Stillness Meditation experience] ... will vary [across sessions] – sometimes it will be 'wonderful', other times 'good' and other times 'not so good'. And that's all right too! It's still 'working', even if a practice session seems on some occasions to be less fruitful than on others." – p. 85<br>This passage is talking about the Stillness Meditation experience as a whole, not just the stillness experience.<br>"When authors speak of meditation, many descriptive words are used such as bliss, harmony, suspension, feeling centred, grounded, attuned, balanced in purity, perfection, joy ... But these are irrelevant [to the experience of stillness]." – p. 85<br>"It is like an undisturbed encounter with 'being' – so pleasant, that one would prefer to remain within that experience indefinitely." – p. 85<br>Quoting a client, "Jennifer": "I was suspended in stillness, held in [a] power which I knew was very good ..." – p. 102<br>Quoting a client, "Jenny": "This experience has been wonderful! So calming. A wonderful calmness ..." – p. 168<br>Quoting a client, "Chris": "[I]t takes no effort and feels wonderful." – p. 169<br>"... contented heart." – p. 203

McKinnon (1991)	"The [young] children I have worked with ... have afterwards volunteered such remarks as, 'it felt good ... nice ...'." – p. 129<br>"[Nine to 11 year olds'] responses have included such comments as ... 'It's good' ..." – p. 131
McKinnon (2002/2008)	"After the experience has concluded, the meditator ... may describe [it] as 'relaxing, refreshing, lovely, peaceful and calm' ... It is not usual to describe this serenity in terms such as 'bliss or rapture or exaltation' ... such expressions are best left to those who actively pursue more dramatic forms of meditation!" – p. 45<br>Quoting an adolescent: "... [T]he experience was wonderful." – p. 86

2.3.20 Beyond calmness/stillness/goodness

Meares (1978/1986)	"We experience pure being ... In the experience beyond relaxation we are not asleep, we are not unconscious, we are doing something. We are being. Just being. ... We simply experience our being in pure form." – p. 153 "We think of ourselves as being serene, as being tranquil, as being still or as being in repose. In each case we relate our being to some other quality. In the experience of pure being this is absent. It goes beyond these concepts in its profound simplicity and utter naturalness. Just being." – p. 154
Meares (1983a)	"When the patient first sits down to meditate, he may experience feelings of calm, ease, naturalness and the feeling that it is good. These feelings pass as the stillness comes. They may recur from time to time, only to pass off spontaneously ..." – p. 119
Meares (1979b)	"... How can you meditate<br>When you are feeling?<br>It's the experience<br>Of naturalness<br>Of such profound simplicity<br>It's beyond all feeling." – p. 83
Meares (1976/1984)	"Feel ourselves relax,<br>Feel the calm,<br>Feel the stillness,<br>Joy in it, ecstasy,<br>But it is only the path.<br>Flowers by the wayside,<br>Vistas between the trees,<br>It is beyond all this<br>Where we seek to go." – p. 12<br><br>"... Feel the calm.<br>Calm of our body, calm of our mind.<br>Calm of our whole self.<br>But more than the calm.<br>It too is the path,<br>The road we must find,<br>The way we must take.<br>That is the joy of the way,<br>Not the end that we seek." – p. 18<br><br>"It feels good,<br>It feels calm,<br>Even sublime.<br>What then do we say,<br>When we go beyond these things?<br>Still, without numbness.<br>Call it the foretaste<br>Of feeling beyond feeling." – p. 32<br><br>"Being profoundly calm<br>Is being profoundly calm.<br>It is not being.

	When being comes we are beyond calm.<br>How could you be calm,<br>If you were simply being?" – p. 36
McKinnon (2011)	"When authors speak of meditation, many descriptive words are used such as bliss, harmony, suspension, feeling centred, grounded, attuned, balanced in purity, perfection, joy ... But these are irrelevant [to the experience of stillness]." – p. 85

2.3.21 No sense of anxiety

See also 2.3.18 ("Calm, ease, peacefulness, absence of disturbance").

Meares (1967/1968)	"In regression ... there is an absence of anxiety." – p. 61<br>"In moments of ... complete mental relaxation our mind fills with calm, and there is an absence of any feeling of anxiety." – p. 61
Meares (1978/1986)	"[In Stillness Meditation] ... anxiety is allayed." – p. x<br>"The most important effect of [Stillness Meditation] is a reduction in ... anxiety." – p. 3<br>"[In the stillness] natural processes of our mind act freely to reduce our anxieties."– p. 6<br>"We become aware of the natural calm within us when our mind is completely free from anxiety." – p. 26<br>"In transcending minor discomfort in [Stillness Meditation] we reduce the level of our anxiety." – p. 49<br>"In the experience beyond relaxation there is complete freedom from anxiety." – p. 151<br>"... [I]n the experience of meditation, [our normal] background of anxiety fades." – p. 151<br>"... [Through Stillness Meditation we] reach that stillness where anxiety is gone ..." – p. 160
Meares (1976/1980)	"[In Stillness Meditation] we are unaware of being anxious or otherwise. If we examine ourselves, our state of mind changes, and we temporarily cease to be in [the stillness]. But in the normal course of events, as we return to full wakeful alertness, we are aware that we are less anxious, and that the level of our nervous tension is much reduced as a result of the experience." – 190
Meares (1977c)	"... profound reduction of anxiety ..." – p. 131
Meares (1983a)	"In the meditation there is a profound reduction of anxiety." – p. 119
Meares (1984)	"... [Stillness Meditation] [r]educes our anxiety<br>And brings us once more<br>A sense of calm within our self." – p. 80
McKinnon (2011)	"Within [the stillness], and for scant moments only, the mind is without anxiety." – p. 84
McKinnon (1991)	"I think the meditative state could best be described as a sensation of immense security embodying a kind of 'knowledge' – or a kind of peace – that allays anxiety, together with an all-pervading restfulness." – pp. 73-74

2.3.22 No focus/concentration

Meares (1978/1986)	"... [In Stillness Meditation there is an] absence of any specific topic as an object on which the mind is focused." – p. 6<br>"Some systems of meditation involve intense concentration ... but in [Stillness Meditation] there is utter effortlessness." – p. 23
Meares (1989)	"No concentrating on some topic." – p. 109
McKinnon (1983/2016)	"... [T]he whole idea of [Stillness Meditation] is never to concentrate ... or focus our attention ... upon anything." – p. 220<br>"... [Y]ou don't require *concentration* within the [Stillness Meditation] experience ..." – p. 253<br>"... [I]f you are focusing [or] directing your thoughts ... your mind is not [resting/still]." – p. 268
McKinnon (2011)	"... [In Stillness Meditation] there is no focus upon rituals or breath, or paying attention ..." – p. 39<br>"Paradoxically, the less attention we give, the more powerful the experience." – p. 40<br>"There is no concentration involved; nothing whatsoever to focus on or be aware of." – p. 74

	"Concentrating ... in any way ... is not conducive to [Stillness Meditation]." – p. 76<br>"... [I]n Stillness Meditation, the less attention we give, the deeper the experience ..." – p. 93<br>"In learning stillness, the teacher assists the meditator to let go beyond the present." – p. 93 "The teacher allows the meditator to [let go] of background sounds, by not paying attention: sounds are not important right now. Neither is awareness: of the body, the environment or the senses." – pp. 93-94 "Especially not important are distractions or discomforts ..." – p. 94<br>"... no directives or focus of any kind to be attended to." – p. 109<br>"... letting go of attention, feeling and thought." – p. 109<br>"In [Stillness Meditation] there is no ... mental focus." – p. 226<br>"... free of ... focus ... of any kind." – p. 238

2.3.23 Transcendence of discomfort

See also 1.10 (Slight/minor discomfort) and 2.3.13 ("Reduced awareness/perception, loss of logical/critical faculties").

Meares (1967/1968)	"... [T]he discomfort [in Stillness Meditation] simply does not disturb us. And because it does not disturb us, we do not really perceive it as discomfort at all." – p. 163
Meares (1978/1986)	"As we relax, our mind transcends the discomfort of our posture and then there is no discomfort." – p. 48
Meares (1973b)	"I don't want you to endure discomfort, I want you to transcend it. To use your mind so that the discomforting influence simply does not worry you. Then of course there is no discomfort." – p. 252
Meares (1987/1991)	"We let our mind run quietly, and we are soon unaware of the initial slight discomfort." – p. 80<br>Meares p. 115 makes the same comment.
Meares (1979a)	"... [T]he regression of mental functioning [in Stillness Meditation] is shown by the loss of alert critical thinking, the dulling of discomfort and pain, the absence of reaction to external stimuli, and the vague perception of things in their fundamental simplicity." – p. 123
McKinnon (1983/2016)	"[A]s the mind becomes still, our ... discomforts ... gradually disappear." – p. 212
McKinnon (2011)	Quoting a client, "Jennifer": "All discomfort of any kind was gone." – p. 102

2.3.24 Naturalness

Meares (1978/1986)	In Stillness Meditation, the mind experiences "its own naturalness" (p. 7).<br>"... [T]here is this easy, natural experience of letting ourself go with the relaxation without restraint." – p. 25<br>"There is ease and naturalness in this letting go." – p. 25<br>"We let ourselves experience the essential calm of our being ... We are aware of the natural calm deep within us. We let ourselves experience it. Naturally. It is all natural." – p. 25<br>"The full meditative experience is beyond the relaxation of the body and mind, beyond the transcendence of discomfort. What is it then? Is it something strange and unnatural? No. There is nothing strange or bizarre about it at all. Nor is there anything dramatic or exciting. Do not expect ecstasy or any outlandish distortions of the mind. Expect rather the experience of deep naturalness. Utter naturalness. It is only when this comes to us that we realise that true naturalness is something quite foreign to us in our ordinary lives." – p. 145<br>"We are relaxed, but we experience something far beyond relaxation. It is our inner ease. The calm that is natural to us." – p. 149<br>"In our meditation we experience naturalness. It is more than the naturalness of the meditative experience, it is the naturalness of it all, of our own self. A deepening and expanding sense of naturalness. There is nothing odd or strange or peculiar about it. We experience the naturalness of what we are doing. As we become immersed in it, the sense of naturalness spreads out, so that it is not only what we are doing that is natural, but we are natural in ourself. We experience our own naturalness ..." – p. 149
Meares (1989)	"[Stillness Meditation] is characterised by a very natural, calm, easy, effortless, stillness of mind." – p. 108<br>"We aim for naturalness. Complete naturalness." – p. 109
Meares (1978b)	"The natural ease of our own self ..." – p. 6

Meares (1988)	"[In Stillness Meditation we let] our mind learn how to run simply, quietly, naturally. Naturally is the key word." – p. 26<br>"[Stillness Meditation] is characterised by a great simplicity, a deep naturalness. It is a profound stillness of the mind." – p. 83
Meares (1982c)	"[Stillness Meditation] is characterized by a great simplicity, a deep naturalness. It is just a profound stillness of the mind." – p. 1607
Meares (1983a)	"[Stillness Meditation] is characterized by simplicity, a very profound simplicity; and by naturalness, a very easy naturalness. The mind is simply allowed to be still." – p. 115<br>"When the patient first sits down to meditate, he may experience feelings of calm, ease, naturalness and the feeling that it is good. These feelings pass as the stillness comes. They may recur from time to time, only to pass off spontaneously ..." – p. 119
Meares (1984)	"... We experience a profound state<br>Of heavenly naturalness,<br>Just the act of being ..." – p. 18<br><br>"... In meditation we experience<br>The ultimate naturalness of our own self ..." – p. 52
McKinnon (2011)	"Simple, obvious, unusual – when immersed in that experience ... one could not fail to be impressed." – p. 44<br>Quoting a client, "Amy": "... I go ... beyond everything, perfectly natural and all so easy. In the stillness I return home, to me." – p. 177
McKinnon (2002/2008)	"It is not usual to describe this serenity in terms such as 'bliss or rapture or exaltation' ... such expressions are best left to those who actively pursue more dramatic forms of meditation!" – p. 45

2.3.25 Effortlessness

See also 1.8 ("Effortlessness").

Meares (1978/1986)	In Stillness Meditation, the mind experiences "its own ... effortlessness" (p. 7).<br>Quoting a patient: "... a calm without trying." – p. 25
Meares (1973b)	"I must emphasize the effortlessness. This is really the key to it all. Not only effortlessness of the body, and the mind too, but a total effortlessness of being." – p. 255
Meares (1989)	"[Stillness Meditation] ... is characterised by a very natural, calm, easy, effortless, stillness of mind." – p. 108<br>"An effortless stillness ... just effortlessly still." – p. 109
Meares (1987/1991)	"... a stillness of effortless tranquility." – p. 114
Meares (1977b)	"... And the stillness.<br>More than letting it be.<br>All without effort.<br>Just letting ..." – p. 18
Meares (1987a)	"... With the mind effortless and still,<br>In a state of complete tranquility ..." – p. 50
McKinnon (1983/2016)	"... deliberate effortless *being* ..." – p. 195<br>"... effortlessly still." – p. 221
McKinnon (2011)	"... [In the stillness experience] all effort is abandoned ..." – p. 69<br>"Stillness Meditation is a simple ... experience of ... just simply letting ... into a period of non-activity and effortless surrender." – p. 75<br>"When we deeply sense the stillness ... there comes a kind of release from the ordinary effort of living." – p. 85

McKinnon (2002/2008) “The ultimate sensation of stillness is a state of complete security and ease, where body and mind are unified in a calm sense of ‘being’ – beyond effort ... of any kind.” – p. 45

2.3.26 Control and relinquishment

See also 1.12 (“Control and relinquishment, discipline and ease”) and 2.3.37 (“Deep”).

Meares (1978/1986) “We experience the letting go of ourself. This involves a sense of freedom, of very great freedom, defencelessness, of completely letting go, of utter abandonment. There is a sense of real abandonment ...” – p. 147 “Besides the letting go of ourself, there is also a deeper process of simply ‘letting ourself’ ... Normally our psychological protective mechanisms from experiencing ‘letting ourself.’ But in the meditative state, and the reduction of anxiety which goes with it, the ‘letting ourself’ is quite safe.” – p. 147
“In the state beyond relaxation we experience complete defencelessness.” – p. 151
“We are awake, but calm, and defenceless.” – p. 151
“... [I]n meditation we experience ourselves as we are. Defenceless. Our true self.” – p. 152
“... [a] loosening of normal control.” – p. 157

Meares (1979a) “... [The patient] is free to abandon himself ...” – p. 123

Meares (1979b) “... So let your mind learn
In the unguarded abandon
Of meditation.” – p. 37

Meares (1984) “In the meditation ...
We abandon ourselves
Freely
As a falling leaf
In the autumn wind ...” – p. 76

Meares (1987a) “... Ease comes of the union
Of restraint and abandonment.

We can learn restraint
In the discipline of meditation,
And abandonment comes
In the letting go of meditation ...” – p. 47

McKinnon (2011) “... effortless surrender.” – p. 75
“... [Y]ou experience [the stillness] ... [C]alm control ...” – p. 81
Quoting a client, “Jennifer”: “I also sensed a control in myself, not known before ...” – p. 102

2.3.27 Loss of ego

See 2.3.4 (“Non-duality”), 2.3.10 (“Emptiness, nothingness, reduced mental activity”), 2.3.13 (“Reduced awareness/perception, loss of logical/critical faculties”), and 2.3.32 (“Inner freedom, unboundedness, spaciousness”).

McKinnon (2011) “During stillness, there is no ego ...” – p. 130

2.3.28 Simplicity, simple/pure being, non-doing

On non-doing, see also 1.8 (“Effortlessness”).

Meares (1978/1986) “In [Stillness Meditation] we let ourselves be. Of all human activity, of both body and mind, this is the simplest ... – the act of just being in all its simplicity and naturalness with nothing added at all ... It is the unadorned, primordial experience of existence. This is *simple being.*” – p. 27
“Pure being ...” – p. 27
“The full meditative experience is beyond the relaxation of the body and mind, beyond the transcendence of discomfort. What is it then? Is it something strange and unnatural? No. There is nothing strange or bizarre about it at all. Nor is there anything dramatic or exciting. Do not expect ecstasy or any outlandish distortions of the mind. Expect rather the experience of deep naturalness ... Simplicity. Such profound simplicity that we are almost overwhelmed by it, immersed in it ... This morning I saw as a patient a twenty-

	three-year-old married speech therapist. It was her third session and she is already much less tense. As she left, she looked at me and said, 'It is the utter simplicity of it'." – p. 145<br>"In our meditation we experience simplicity." – p. 145 "We experience the simplicity of our meditation, and soon find that we are experiencing a sense of our own simplicity. The process deepens. It soon becomes more than a sense of simplicity. It is simplicity itself. We are it. It is us. Not the sensation, not a quality that we take on, but the thing itself." – p. 150<br>"There is a simplicity of such depth ... It is a sense of being. Just being in its purest form." – p. 150<br>"We experience pure being ... In the experience beyond relaxation we are not asleep, we are not unconscious, we are doing something. We are being. Just being ... We simply experience our being in pure form." – p. 153 "We think of ourselves as being serene, as being tranquil, as being still or as being in repose. In each case we relate our being to some other quality. In the experience of pure being this is absent. It goes beyond these concepts in its profound simplicity and utter naturalness. Just being." – p. 154
Meares (1989)	"When our mind is still, there is nothing. Just quietness. Just *being*." – p. 115<br>"In meditation we are just being. There is a very great simplicity." – p. 115 "Something of profound simplicity." – p. 116<br>"There is great simplicity about it. Profound simplicity." – p. 129
Meares (1987/1991)	"At the start it is best just to let ourselves experience a sense of being. Just being. Not even being in the room. Not even being alive. Just being. This state of mental activity, or rather inactivity, is a step towards the real stillness of mind experienced in full meditation." – p. 114<br>"Eventually there comes an emptiness of the mind. A sense of being. Just pure being." – p. 127<br>"The final experience is simply being ..." – p. 127
Meares (1970)	"You just let yourself be ..." – p. 67
Meares (1988)	"[Stillness Meditation] is characterised by a great simplicity, a deep naturalness. It is a profound stillness of the mind." – p. 83
Meares (1977c)	"[Stillness Meditation] is characterized by stillness. There is an absence of intellectual activity, an absence of sensory experience and an absence of emotion ... The subject may be aware of his existential being but little else." – p. 131
Meares (1979d)	"The individual is ... simply experiencing his essential being." – p. 120
Meares (1982c)	Meares p. 1607 makes a similar comment to Meares (1988, p. 83) above.
Meares (1983a)	"[Stillness Meditation] is characterized by simplicity, a very profound simplicity; and by naturalness, a very easy naturalness. The mind is simply allowed to be still." – p. 115
Meares (1979b)	"... If you grasp<br>That it is simple,<br>You grasp it all." – p. 12
McKinnon (1983/2016)	"... the art of doing absolutely nothing." – p. 221<br>"... [S]tillness is really the experience of *doing nothing* ... that is, simply *being* ..." – p. 222
McKinnon (2011)	"[The stillness] is like an undisturbed encounter with 'being' ..." – p. 85<br>"... effortlessly being alive." – p. 86<br>"... [I]t is ... perhaps best described as a state of being that just *is*." – p. 101<br>Quoting a client: "[I]t's truly such a simple experience!" – p. 120<br>Quoting Francine Cockerill and Kaye Hakopian, teachers at McKinnon's Stillness Meditation Therapy Centre: "The experience of stillness is very simple. Some might say, 'Is that it?' meaning that they may be unimpressed or unconvinced of its effectiveness – at least until time proves otherwise." – p. 164<br>Quoting a client, "Amy": "... the naturalness of my being beyond body, beyond mind ..." – p. 179
McKinnon (1991)	"[Stillness Meditation] is *being* not *doing*." – p. 74<br>"... [T]he whole self is very still – just *being* ..." – p. 77<br>"... [J]ust simply be." – p. 127

McKinnon (2002/2008)	"The ultimate sensation of stillness is a state of complete security and ease, where body and mind are unified in a calm sense of 'being' – beyond effort ... of any kind." – p. 45

2.3.29 Nature of mind/being, self

Meares (1978/1986)	In Stillness Meditation, the mind experiences "its own naturalness, ease and effortlessness" (p. 7).<br>"We let ourselves experience the essential calm of our being ... We are aware of the natural calm deep within us. We let ourselves experience it." – p. 25<br>"[T]he natural calm within us ..." – p. 26<br>"... the calm within us ..." – p. 26<br>"[Stillness Meditation] allows us to experience the essential stillness of mind." – p. 48<br>"... [W]e have the experience of our own inner ease." – p. 146<br>"In [Stillness Meditation] we experience the inner freedom that is part of our being." – p. 147<br>"We are relaxed, but we experience something far beyond relaxation. It is our inner ease. The calm that is natural to us." – p. 149<br>"In our meditation we experience naturalness. It is more than the naturalness of the meditative experience, it is the naturalness of ... our own self." – p. 149<br>"We experience our own naturalness ..." – p. 149<br>"... [I]n [Stillness Meditation] we experience ourselves as we are. Defenceless. Our own true self." – p. 152<br>"In [Stillness Meditation] we may experience a sense of good that lies deep within ourselves ... We experience something very good – an awareness of a source of good within us ... [I]n [Stillness Meditation] we come face to face, as it were, with this natural capacity for good which is within us." – p. 155<br>"... the stillness that lies deep within each one of us." – p. 160
Meares (1973b)	"... the inner calm which is natural to us." – p. 177<br>"Not only effortlessness of the body, and the mind too, but a total effortlessness of being." – p. 255
Meares (1987/1991)	"... letting ourselves experience the inner ease which is a natural part of us." – p. 124
Meares (1970)	"You just let yourself be, and the relaxation is there because it is natural to you." – p. 67
Meares (1978b)	"The natural ease of our own self ..." – p. 6
Meares (1976b)	"... the experiencing of our own essential ease." – p. 909
Meares (1979d)	"The individual is ... simply experiencing his essential being." – p. 120
Meares (1979b)	"... Meditation<br>Is simply a way<br>Of releasing<br>What is already there." – p. 2
Meares (1984)	"... the stillness<br>Of our own self ..." – p. 35<br><br>"... In meditation we experience<br>The ultimate naturalness of our own self ..." – p. 52<br><br>"... We release the profound naturalness<br>That lies within us ..." – p. 106
Meares (1987a)	"... Ease is the natural state<br>Of our natural being ..." – p. 40
McKinnon (1983/2016)	"... our own intrinsic calm and stillness of being." – p. 206<br>"... our own stillness." – p. 210<br>"... [T]he stillness is there within ourselves." – p. 211<br>"... [W]e aim to experience only *our own inner stillness*." – p. 220<br>"... simple yet profound experience of one's own natural inner calm" – p. 289

McKinnon (2011)	"The experience already *is* ... it is there within each person." – p. 39<br>"The calm of one's being is right there within." – p. 40<br>"... one's innate calm." – p. 40<br>"The stillness is already within you ... waiting to be unlocked." – p. 75<br>"... *your own natural calm*." – p. 78<br>"... your own utter calm ..." – p. 81<br>"... our own natural calmness ..." – p. 83<br>"... own natural calm." – p. 94<br>"Meares' message is simple ... Ease ... is there within each person." – p. 123<br>"... [T]he stillness is there within." – p. 149<br>Quoting a client, "Amy": "In the stillness I return home, to me." (p. 177); "... letting go into the natural ease of being, being the naturalness of my being beyond body, beyond mind ..." (p. 179); "It's about ... the simplicity and naturalness of our own ease of being." (p. 180); "... the stillness of our own being is revealed ..." (p. 180).<br>"[Stillness Meditation] involves experiencing the natural stillness within." – p. 228
McKinnon (1991)	"... [The stillness] is already there, within us, waiting for release." – p. 132
McKinnon (2002/2008)	"... Stillness Meditation is essentially the formal practice of experiencing one's own natural inner calm." – p. 7<br>"... the natural experience of one's own essential stillness." – p. 13<br>"There is simply the profound experience of the person's own natural stillness." – p. 15

2.3.30 Wholeness, integration, unity

See also 2.3.32 ("Inner freedom, unboundedness, spaciousness") below.

Meares (1978/1986)	"More than our body relaxes. Now it is our whole self that is relaxed, our ego, our total being." – p. 19 "It is no longer a matter of our muscles or our body being relaxed because the distinction between our body and ourself has faded. It is all one. The relaxation becomes a total experience which involves our whole being." – pp. 19-20 "The separate entities cease to be separate. There is now only one. The experience, ourself and the relaxation are all one." – p. 20<br>"The fact that the relaxation is not simply of our body, and not simply of our mind, but is of our whole being brings a sense of integration and unity. There are no separate parts. A profound unity. Something deep. We sense this unity as our inner being. Oneness, our indivisible self." – pp. 31-32<br>"We experience our body, our mind, our inner self, our own self. But it is all one. A complete integration ... [I]n our meditation we experience this integration. It is more than an integration of discrete parts. It is wholeness. This is our being." – p. 149 "[T]here is no body and mind, or inner self and our own self, it is just the experience of our being." – p. 149<br>"In our meditation we experience something of the natural harmony of things. In ordinary life, when we care to, we can think about the natural harmony of the universe, and closer at hand, the natural harmony of nature as we see it in our daily life. But we rarely experience this harmony as part of ourself ... Now, when we come to meditate it is quite different ... [I]n [the] [stillness of mind] the sense of harmony comes to us. Then as we go further, it is no longer just a sense of harmony. It becomes the experience of harmony so that we, body and mind and our whole being, are participating in it." – p. 150<br>"... true harmony in ourselves." – p. 160
Meares (1984/1989)	"May I come to be<br>More completely at one<br>With all those things<br>That bring<br>Unity to life?" – p. 52<br><br>Viewed in context, this verse implies that Stillness Meditation can help achieve that outcome.
McKinnon (1983/2016)	"Soon, during meditation, I began to come upon a velvety stillness and an at-one-ness with my-*self* and my surroundings ..." – p. 195<br>"... our *whole being* is at one ..." – p. 225
McKinnon (2011)	"Within [the stillness] we feel ... very whole ..." – p. 85

	"When authors speak of meditation, many descriptive words are used such as bliss, harmony, suspension, feeling centred, grounded, attuned, balanced in purity, perfection, joy ... But these are irrelevant [to the experience of stillness]. In stillness ... [w]e are in profound accord with oneself and one's place in existence." – p. 85<br>"Within [Stillness Meditation] there is an indescribable sensation of secure communion with all things." – p. 118<br>Quoting a client, "Amy": "My inner core was rebuilding ..." – p. 179<br>"Deep peace, secure, at one with all ..." – p. 203
McKinnon (2002/2008)	"The ultimate sensation of stillness is a state of complete security and ease, where body and mind are unified in a calm sense of 'being' – beyond effort ... of any kind." – p. 45

2.3.31 Inner security, safety, strength, contentment

See also 2.3.18 ("Calm, ease, peacefulness, absence of disturbance"), 2.3.19 ("Good/wonderful"), and 2.3.26 ("Control and relinquishment").

Meares (1983b)	With reference to patients with cancer practising intensive Stillness Meditation: "Through intensive meditation, the patient experiences a profound sense of calm and inner security." – p. 583
Meares (1979b)	"Can you give strength<br>To another?<br><br>You can muster the strength<br>That lies deep within him.<br>It grows in the calm<br>And the stillness." – p. 32
McKinnon (1983/2016)	"Soon, during meditation, I began to come upon a velvety stillness ... And an inner 'knowing' of the essence of stillness and strength of life evolved ..." – p. 195<br>"... I found I now began ... to *experience* the inner strength ... ; with that came an increased sense of security." – p. 196<br>"... something new, different, calm, secure, safe and strong – and utterly *still*." – p. 225<br>"... the security of peace." – p. 268
McKinnon (2011)	"... [Y]ou experience [the stillness] ... An inner strength ..." – p. 81<br>"*... stillness ... embraced in ... spaciousness and comforting enclosure ...*" – p. 81<br>"Within [the stillness] we feel very safe ..." – p. 85<br>"[In the stillness] ... [w]e are ... somehow, very strong." – p. 85<br>"... [T]here is a simple experience of cohesive strength, serenity, comfort and ease." – p. 85<br>"... a very safe and content knowledge that all is well." – p. 101<br>Quoting a client, "Jennifer": "Everything I needed – safety, security, strength, peace – was within that stillness ... I sensed myself and how good it was to be so peaceful and strong." – p. 102<br>Quoting a client, "Cathy": "I have experienced inner calm and inner strength ..." – p. 173<br>Quoting a client, "Amy": "I felt a spark of hope, as if I could be my own pillar of strength again but in a whole new way that was totally natural and so easy ..." – p. 179<br>"Deep peace, secure, at one with all ..." – p. 203
McKinnon (1991)	"I think the meditative state could best be described as a sensation of immense security embodying a kind of 'knowledge' – or a kind of peace – that allays anxiety, together with an all-pervading restfulness." – pp. 73-74
McKinnon (2002/2008)	"The ultimate sensation of stillness is a state of complete security and ease, where body and mind are unified in a calm sense of 'being' – beyond effort ... of any kind." – p. 45<br>"After the experience has concluded, the meditator can usually reflect on having experienced a state of serenity and security ... or contentment." – p. 45

2.3.32 Inner freedom, unboundedness, spaciousness

See also 2.3.30 ("Wholeness, integration, unity"), and the various references to letting the mind run freely, and participating freely in the experience, in Part 1.

Meares (1978/1986)	"We experience the letting go of ourself. This involves a sense of freedom, of very great freedom ..." – p. 147 "Besides the letting go of ourself, there is also a deeper process of simply 'letting ourself'." – p. 147 "... [I]n our meditative relaxation there is this deep 'letting of ourself'. A kind of freedom of our being ... " – p. 147 "In the letting go of [Stillness Meditation] we experience the inner freedom that is part of our being." – p. 147 "There is an experience of freedom. We are free inwardly." – p. 148 "Our mind is free from its usual constraints." – p. 148<br>"A fifty-year-old married woman had complained that she had been very agitated ever since she could remember. Later when speaking of [Stillness Meditation] she said, 'Getting stillness with it. A freedom. So still, so free. Nothing holding me back like there used to be'." – p. 149<br>"In the experience beyond relaxation there is complete freedom from anxiety." – p. 151<br>"In the experience beyond relaxation our physical identity merges with the world around us. In our ordinary waking life we feel that there is a very clear distinction between what is me and what is not me. Our physical being is clearly determined by the boundaries of our body. But in the experience of [Stillness Meditation] the sharpness of this distinction is lost. We have our place with these other things around us. Like ourselves they are part of it all, and in this greater picture the boundaries between ourself and these other things seems less important. It comes to be a matter of no importance at all. In fact there is nothing between us and these other things, and our physical identity merges with them." – pp. 152-153<br>"Besides our physical identity, our personal identity also undergoes changes ... In [Stillness Meditation] the confines of our personal identity are expanded. There comes about a change so that we are no longer confined to our body, but are part of the world around us. It is my world just as it has been my body, and the seat of my identity is now of the world rather than my body." – p. 153
Meares (1987/1991)	"... letting his mind run freely [in Stillness Meditation] ..." – p. 39
Meares (1988)	"... [I]nner freedom of mind is likely to come to us in ... [Stillness Meditation]." – p. 91
Meares (1977b)	"... The freedom within" – p. 96
Meares (1979b)	"You say in doing this<br>My mind can work more freely.<br><br>Of course,<br>In meditation<br>The brakes are off.<br>What is there<br>To hold it back?" – p. 15<br><br>"I would seek<br>A greater freedom within ...<br><br>... [F]reedom to let go<br>That's what we learn." – p. 17<br><br>"... How does our mind run freely?<br>That's what we teach." – p. 35
Meares (1987a)	"Ease<br>Unties the leash<br>That tethers our mind ..." – p. 46
McKinnon (2011)	"... an experience without restraint ..." – p. 45<br>"*... stillness ... embraced in ... spaciousness and comforting enclosure ...*" – p. 81<br>"... closed yet spacious ..." – p. 101<br>"Within [Stillness Meditation] there is an indescribable sensation of secure communion with all things." – p. 118

2.3.33 Timelessness

Meares (1976/1980)	"The perception of the passage of time is distorted." – p. 190
McKinnon (2011)	"... *stillness ... embraced in timelessness* ..." – p. 81<br>" ... suspended in time ..." – p. 85<br>"For me [the stillness] is something timeless ..." – p. 101<br>"... [T]his precious calm, a space imbued with ease and timelessness." – p. 203

2.3.34 Knowledge, understanding

Meares and McKinnon make clear that new insights can emerge from the goal-states. They might be in the form of specific knowledge that can be articulated to others, or they might be what Meares describes as non-verbal. With reference to one of these non-verbal understandings, Meares (1978/1986, p. 154) explains: "You, the reader, may well ask, 'If you have had experience of this ... understanding, why don't you tell us about it?' The answer, of course, is that this understanding does not come through reason and the logical workings of our mind, so it is not possible to describe it accurately in logical writing ... I am trying rather to lead you into an experience ... and the understanding ... will come to you by this indirect means." On this basis Meares refrains from attempting to describe the understandings precisely, however, he does talk about them in general terms. For example, he says, "... [I]f we first reduce our anxiety by [Stillness Meditation], then understanding of the deeper nature of our being can come to us. In particular, we can gain some sense of our true identity, of the meaning of our sexuality, of our relationship between the realistic and spiritual value systems within us, and of our place in the natural order of things" (Meares, 1978/1986, p. 73). As the nature of the insights is not important for the purposes of this table, the table does not include Meares' other comments about them. The extracted statements in this section below focus on Meares' and McKinnon's more general comments about the goal-states being related to some form of knowledge.

Meares (1978/1986)	"... [A]s our meditation proceeds ... ideas come to our awareness ... [T]he new ideas may bring us flashes of insight which are beyond us in our ordinary waking state." – p. 146
Meares (1973b)	"... [I]n the actual periods of [Stillness Meditation], we often gain new insights, new understanding. There is a peculiar quality in the understanding which comes to us in these circumstances. There is a depth in it because it comes about as a result of the prelogical functions of the mind." – pp. 257-258
Meares (1977b)	Meares puts himself in the position of a notional cancer patient who has practised Stillness Meditation intensively. Of cancer, experienced together with the Stillness Meditation practice, he reflects:<br><br>"It has taught me to know<br>In a way<br>That had not come to me before ...<br><br>[It] has brought new knowing<br>To my mind<br><br>But it's not my mind at all.<br>Just knowing,<br>It has taught me this." – p. 84
Meares (1979b)	"... [I]n the calm and the stillness<br>There's a different kind of knowing." – p. 27<br><br>"... Knowing is for two plus two,<br>But what we want<br>Lies far beyond<br>Such a simple process of the mind." – p. 61<br><br>"... [I]n the calm and the stillness<br>There comes a way of knowing." – p. 80<br><br>"You talk of this other knowing.<br>I'm an ordinary person,<br>Tell me<br>What is it?

	It is the knowing<br>Of ordinary people<br><br>When it is not confused<br>By their thinking about it." – p. 81
Meares (1980e)	"To think<br>Without thoughts in our mind;<br>And we come to know<br>What we did not know before." – p. 3<br><br>"... [I]n stillness<br>And not knowing<br>We come to know." – p. 5<br><br>"In a momentary encounter<br>We may transcend<br>A lifetime of knowing." – p. 15
Meares (1982b)	"... All is calm and quiet;<br>Then there comes this other experience.<br>And again<br>I know what I did not know before." – p. 32<br><br>"My soul,<br>You let it come slowly and wisely,<br>In fragments,<br>Piece after piece,<br>So I myself<br>Must put it together<br>In the great mosaic." – p. 39
Meares (1976/1984)	"... [I]n stillness it comes<br>The knowing beyond words.<br>Does the rosebud work it out,<br>How to unfold her petals?<br>It comes in the stillness,<br>And then we understand." – p. 22<br><br>"... It comes in the calm and the stillness.<br>To know beyond words ..." – p. 23
Meares (1987a)	"Ease brings understanding.<br>What's the understanding that comes with ease?<br>It's something more<br>Than is written down in black and white.<br>It's the spirit of what we need to know<br>Rather than the knowledge itself." – p. 9<br><br>"Ease lets comes to us<br>The meaning of what poets write,<br>Without such ease<br>Our mind is ever guarding us,<br>And so obscures<br>This other reality of our being." – p. 30<br><br>"Create<br>By diligence and hard work.<br>That's one kind of creating.<br>For the other,

	The break-through<br>Comes when we allow our mind<br>Transcend the constriction<br>Of all we've been taught,<br>And we burst through to something new." – p. 46
Meares (1984/1989)	"The only knowledge<br>Is that form of knowing<br>Which lets us understand<br>We need not know." – p. 50
McKinnon (1983/2016)	"Soon, during meditation, I began to come upon a velvety stillness ... And an inner 'knowing' of the essence of stillness and strength of life evolved ..." – p. 195
McKinnon (2011)	"... a very safe and content knowledge that all is well." – p. 101<br>Quoting a client, "Jennifer", in her first Stillness Meditation class: "I ... sensed ... a kind of knowing. It seemed as if answers were there for me, all problems could easily be solved." – p. 102<br>"... a surge of wisdom ..." – p. 203
McKinnon (1991)	"I think the meditative state could best be described as a sensation of immense security embodying a kind of 'knowledge' – or a kind of peace – that allays anxiety, together with an all-pervading restfulness." – pp. 73-74

2.3.35 Possible spiritual aspect

Although Meares presented Stillness Meditation as principally a practical self-help technique for people with anxiety, pain and other afflictions (e.g., Meares, 1967/1968), in his later writings he discussed how the stillness may be experienced as having some spiritual quality, at least by people inclined towards spiritual experience. For instance, Meares (1981b), one of his verse-style books, explores the idea that, at least for certain people, the practice might be conceived as a form of wordless prayer. The connections between meditation and prayer are also considered in Meares (1976/1980). McKinnon has a similar perspective. She states that "Stillness Meditation ... is not related in any way to ... religion. In itself it is not a spiritual practice. It is a practical and disciplined therapy. With improved health as its outcome, one is more able to expand one's entire life – both the physical outer life and the mental and spiritual inner life. When [it] is practised, spiritual development may occur – just as physical ease may be attained, just as the emotions may be tempered and just as the logical mental faculties may be sharpened" (McKinnon, 2011, p. 117). Meares' and McKinnon's detailed reflections on the soul and the inner self (e.g., McKinnon, 2011 pp. 137-149; Meares, 1982b) can be seen as explorations of spiritual life deriving, in part, from their own experience of the practice. For the purposes of this table, it is sufficient to note the spiritual aspect without attempting to describe it in detail. The clearest passages relating to this element are set out in this section below. These are provided for illustrative purposes, and are not intended to be exhaustive.

Meares (1979b)	"I sometimes wish<br>I were closer to God.<br><br>It may well be true<br>That God<br>Is in the noise and the bustle,<br>But perhaps<br>It is easier to find Him<br>In the calm and the stillness." – p. 88
Meares (1984)	"... [O]thers make no call<br>On what is religious or spiritual,<br>But simply feel their meditation<br>As a profound experience<br>In the naturalness of their being." – p. 47<br><br>"In the meditation ...<br>There comes an awareness<br>Of another dimension of our being.<br>An awareness that's not an awareness.<br>An experience in which we are not aware

	of what we are experiencing ...<br>It concerns the spiritual aspect of our being.<br>Spiritual.<br>Nothing to do with religion ...<br>Beyond our body and our mind<br>This is the third component<br>Of what we are ...<br>There lies this other land,<br>The land of the spirit<br>Of which we catch glimpses<br>Just glimpses ...<br>In meditation.<br><br>If it were only myself<br>Who caught these glimpses<br>I would have to agree with you<br>That I was probably mistaken.<br>But this is not so.<br>Many people<br>Who meditate in this way<br>Have indicated to me<br>That they have had glimpses<br>Of this third dimension<br>Of their being ..." – p. 147
McKinnon (1991)	"... Dr Meares ... recognised the spiritual dimension found by many of his patients in the practice of [Stillness Meditation] ... There is no doubt that many people find their spiritual need within the practice of stillness of mind. For those who desire this, meditation – with an open heart and mind and being, and free from the fetters of the material world – can be a very successful way to God." – p. 87

2.3.36 Profound

Meares and McKinnon use the term profound to describe the following aspects of the experience: the stillness (McKinnon, 2002/2008, pp. 14-15, 1983/2016, p. 205; Meares, 1979d, p. 120, 1982c, p. 1607, 1988, p. 83); relaxation (McKinnon, 2011, p. 238; Meares, 1967/1968, p. 59, 1978/1986, p. 22); calm (McKinnon, 1983/2016, p. 289; Meares 1983b, p. 583, 1976/1984, p. 36); ease (McKinnon, 1983/2016, p. 268; Meares, 1989, p. 109); tranquility (Meares, 1987/1991, p. 80); reduction in anxiety (Meares, 1977c, p. 131, 1983a, p. 119); naturalness (Meares, 1984, pp. 18, 47, 106); simplicity (Meares, 1979b, p. 83, 1983a, p. 115, 1978/1986, pp. 145, 154, 1989, p. 116); unity or harmony (McKinnon, 2011, p. 85; Meares, 1978/1986, p. 31); and inner security (Meares, 1983b, p. 583). The extracted statements below provide further elaboration.

Meares (1978/1986)	"The natural calm within us is to be distinguished from the calm of drowsiness. It is the ease of the smooth functioning of our body and our mind. Expressed like this it sounds rather pedestrian. This is so only because so few of us ever experience the smooth functioning of our body and mind. When it comes to us it is really an experience of the highest order. Something that far transcends the ordinary experience of life as most of us know it. People whom I have been leading into [Stillness Meditation] have often said to me, 'That was wonderful, truly wonderful'. From the expression of their face I have known that what they said was true, and that for the first time in their lives they had experienced the natural calm within us." – p. 26<br>"The full meditative experience is beyond the relaxation of the body and mind, beyond the transcendence of discomfort. What is it then? Is it something strange and unnatural? No. There is nothing strange or bizarre about it at all. Nor is there anything dramatic or exciting. Do not expect ecstasy or any outlandish distortions of the mind. Expect rather the experience of deep naturalness. Utter naturalness. It is only when this comes to us that we realise that true naturalness is something quite foreign to us in our ordinary lives. Simplicity. Such profound simplicity that we are almost overwhelmed in it, immersed in it." – p. 145<br>"In our meditation we experience naturalness. It is more than the naturalness of the meditative experience, it is the naturalness of it all, of our own self ... There is nothing odd or strange or peculiar about it." – p. 149
McKinnon (2011)	"Simple, obvious, unusual – when immersed in that experience ... one could not fail to be impressed." – p. 44

	Quoting Francine Cockerill and Kaye Hakopian, teachers at McKinnon's Stillness Meditation Therapy Centre: "The experience of stillness is very simple. Some might say, 'Is that it?' meaning that they may be unimpressed or unconvinced of its effectiveness – at least until time proves otherwise." – p. 164

2.3.37 Deep

Meares (1978/1986)	"... [W]e can ... estimate the depth which we have attained by the subjective phenomena which we have experienced ... Any awareness of our state of mind at the time of meditation means that we are not deep as we are obviously functioning at a critical level. But afterwards we can be aware that we have been oblivious of our surroundings and experiencing stillness of mind." – p. 49<br>"We experience the letting go of ourself. This involves a sense of freedom, of very great freedom, defencelessness, of completely letting go, of utter abandonment. There is a sense of real abandonment ..." – p. 147 "Besides the letting go of ourself, there is also a deeper process of simply 'letting ourself' ..." – p. 147<br>"We experience the simplicity of our meditation, and soon find that we are experiencing a sense of our own simplicity. The process deepens. It soon becomes more than a sense of simplicity. It is simplicity itself. We are it. It is us. Not the sensation, not a quality that we take on, but the thing itself." – p. 150
Meares (1971a)	Meares describes the small, private group sessions that he conducted: "Nearly everyone is in quite [a] deep [state of meditation] in a few minutes. My words give way to unverbalized phonation ... Silences develop, and the patients drift still deeper into [the meditation]." – p. 676
Meares (1976/1984)	"We go deeply,<br>Deeply in ourselves.<br>And there is calm and the stillness.<br>And more ..." – p. 52<br><br>"How do we gauge it?<br>What is our yardstick?<br>Some talk of wonderous things,<br>Of lights and shapes and colours,<br>Of the drifting soul,<br>Of voyage in space and time.<br>No.<br>This is not it.<br>Tinsel and gaudy.<br>Only the calm and the stillness ..." – p. 55
McKinnon (1983/2016)	McKinnon p. 193 says of her initial experience with Stillness Meditation: "The effect of the frequent visits [to Meares' classes] and my home practice was dramatic, to say the least. I was experiencing something that was most definitely new to my adult life: two hours each week of absolute stillness, as well as the times I took for daily practice. And gradually, and unbelievably, something was changing." – p. 193 Describing a later period, she says, "As I experienced and practised [Stillness Meditation] more and questioned it *less*, I began to glimpse the right idea, and the experience itself became freer ... Soon ... I began to come upon a velvety stillness ..." – p. 195<br>"... ever-deepening calm." – p. 211<br>"You let go into [the stillness] more and more and more completely ..." – p. 227<br>"Why is one stillness session really deep and at other times nothing seems to happen at all? ... That is normal and will eventually stabilise. Nevertheless, benefits will be forthcoming regardless." – p. 274
McKinnon (2011)	Describing Meares' meditation sessions: "[After his preamble] the remainder of the session was comprised of ... an experience of deep and ever-deepening stillness." – p. 44<br>"... very deep and far reaching stillness ..." – p. 44<br>"As the stillness is repeated more and more, the mind learned gradually that it is safe to let go." – p. 60<br>"... [Y]ou experience only *stillness* at this time and you *experience it more and more and more* ... an ever-pervading stillness and the calm within it ..." – p. 80<br>"You can sense [the stillness] ... and you *experience* it deeply ... more and more and more completely ..." – p. 81<br>"The depth of stillness evolves through repetition and practice ..." – p. 84

2.3.38 Subjective experience varies between meditators and across/within sessions
See also 2.3.36 ("Profound") and 2.3.37 ("Deep").

McKinnon (1983/2016)	"It is not really possible to specify how one feels [in Stillness Meditation], since the ... experience is individual to each [person]." – p. 268<br>"... [E]ach meditative experience is as unique as the meditator." – p. 276
McKinnon (2011)	"The experience of Stillness Meditation is individual and will always be expressed personally. Each person's experience will vary from one time to the next, too. And the quality will also vary ..." – p. 85<br>"Each person will experience something unique to themselves." – p. 101<br>"The practice of meditation ... is very subjective ... It is unlikely that any one individual experience of meditation will ever be replicated." – p. 148

2.3.39 Description in words is limited

Meares (1978/1986)	"[In seeking to describe the non-duality] my use of words fails." – p. 20<br>"Words fail. We can only fully understand meditation by experiencing it. This is true. But it should not deter us in our quest as we can understand many other human experiences only when we have experienced them. Many years ago I had been discussing meditation with a very old and saintly yogi in the Himalayas. One morning he said to me, 'You can show a child a banana, but you cannot tell him how it tastes'. This is a profound truth. And of course this is so with meditation." – p. 145
Meares (1989)	"I have emphasised that the thinking part of our mind drifts into inactivity so that logical thought ceases. I am writing this for you in logical fashion. You can see the problem. It is almost impossible to describe by the logical use of words a process which is outside the framework of logic." – p. 113
Meares (1979b)	"What is meditation<br>Just tell me that?<br><br>A thousand ask the question,<br>Yet none reply;<br>But when it comes<br>There are none<br>Who do not know." -p. 93<br><br>"Is there something more<br>Than can be taught in words?<br><br>It comes in the stillness." – p. 96
McKinnon (2011)	"This is an experience far, far beyond words." – p. 85<br>"To describe Stillness Meditation is impossible ... because description involves understanding, which involves feeling, and feeling is contrary to pure stillness. Stillness Meditation is beyond words, because words involve understanding and feeling and perception. It is beyond sensation too, as sensation involves awareness which involves understanding, feeling and perception. Stillness ... is almost beyond definition, which of course involves all those facets of the intellect and the logic surrounding them!" – pp. 85-86 "Like an affinity with a work of art or the veiled message of poetry, Stillness Meditation is a lived reality that is limited by language." – p. 86

Note. Version 1.0. There is a degree of overlap or interrelationship between the aspects of the technique, interim-states and goal-states described in the different sections of the table. For a proper understanding of each aspect, the relevant section/s should therefore be considered in the context of the table as a whole. To assist in that regard, where a particular section is closely linked to other sections, cross-references have been provided. The headings for each section indicate its subject matter or theme, and aim to capture the main thrust or gist of its content. They do not necessarily capture aspects outside that main thrust or gist, such as statements of a marginal and/or qualifying nature.

References

Hosemans, D. J. F. (2017). *Equanimity and the attenuation of psychological distress*. Doctoral dissertation, Monash University, Australia. Available at: https://figshare.com/articles/Equanimity_and_the_Attenuation_of_Psychological_Distress/5508625.

McKinnon, P. (1991). *Help yourself and your child to happiness*. Melbourne, Australia: David Lovell.

McKinnon, P. (2002/2008). *Let's be still: Teaching Stillness Meditation to children and adolescents*. Melbourne, Australia: David Lovell.

McKinnon, P. (2011). *Living calm in a busy world: Stillness Meditation in the Meares tradition*. Melbourne, Australia: David Lovell.

McKinnon, P. (1983/2016). *In stillness conquer fear: How to break free from anxiety and phobias*. Rev. ed. Melbourne, Australia: Garratt.

Meares, A. (1967a). Pain and the psychiatrist. *Australian and New Zealand Journal of Psychiatry* 1, 116–117. doi:10.3109/00048676709159179.

Meares, A. (1967b). Psychological control of organically determined pain. *Annals of the Australian College of Dental Surgeons* 1, 42–46.

Meares, A. (1967d). Teaching the patient control of organically determined pain. *The Medical Journal of Australia* 1, 11–12. doi:10.5694/j.1326-5377.1967.tb20984.x.

Meares, A. (1968b). Psychological mechanisms in the relief of pain by hypnosis. *American Journal of Clinical Hypnosis* 11, 55–57. doi:10.1080/00029157.1968.10402002.

Meares, A. (1967/1968). *Relief without drugs: The self-management of tension, anxiety, and pain*. London, United Kingdom: Souvenir Press.

Meares, A. (1969b). *Strange places and simple truths: A psychiatrist looks at eastern mysticism*. United Kingdom, London: Souvenir Press.

Meares, A. (1969c). *Student problems and a guide to study*. Melbourne, Australia: Hawthorn Press.

Meares, A. (1970). *The way up: The practical psychology of success*. London, United Kingdom: Souvenir Press.

Meares, A. (1971a). Group relaxing hypnosis. *The Medical Journal of Australia* 2, 675–676.

Meares, A. (1973a). A psychiatric experiment in community service. *The Medical Journal of Australia* 15, 733–734. doi:10.5694/j.1326-5377.1973.tb110661.x.

Meares, A. (1973b). *Dialogue with youth*. London, United Kingdom: Collins.

Meares, A. (1975). *Why be old? How to avoid the psychological reactions of ageing*. London, United Kingdom: Collins.

Meares, A. (1976b). *The relief of anxiety through relaxing meditation*. Australian Family Physician 5, 906–910.

Meares, A. (1977a). Atavistic regression as a factor in the remission of cancer. *The Medical Journal of Australia* 2, 132–133. doi:10.5694/j.1326-5377.1977.tb99090.x.

Meares, A. (1977b). *Cancer: Another way?*. Melbourne, Australia: Hill of Content.

Meares, A. (1977c). The quality of meditation effective in the regression of cancer. *The Journal of the American Society of Psychosomatic Dentistry and Medicine* 25, 129–132.

Meares, A. (1978b). *The hidden powers of leadership*. Melbourne, Australia: Hill of Content.

Meares, A. (1978c). Vivid visualization and dim visual awareness in the regression of cancer in meditation. *The Journal of the American Society of Psychosomatic Dentistry and Medicine* 25, 85–88.

Meares, A. (1979a). Atavistic communication by touch in the psychological treatment of cancer by intensive meditation. *Journal of Holistic Health* 4, 120–124.

Meares, A. (1979b). *Dialogue on meditation*. Melbourne, Australia: Hill of Content.

Meares, A. (1979d). *Meditation: A psychological approach to cancer treatment*. The Practitioner 222, 119–122.

Meares, A. (1976/1980). *Let's be human: New reactions for old*. Melbourne, Australia: Hill of Content.

Meares, A. (1980e). *Thoughts*. Melbourne, Australia: Hill of Content.

Meares, A. (1981b). *Prayer and beyond*. Melbourne, Australia: Hill of Content.

Meares, A. (1982b). *My soul and I*. Melbourne, Australia: Author.

Meares, A. (1982c). *Stress, meditation and the regression of cancer*. The Practitioner 226, 1607–1609.

Meares, A. (1983a). A form of intensive meditation associated with the regression of cancer. *American Journal of Clinical Hypnosis* 25, 114–121. doi:10.1080/00029157.1982.10404082.

Meares, A. (1983b). Psychological mechanisms in the regression of cancer. *The Medical Journal of Australia* 1, 583–584. doi:10.5694/j.1326-5377.1983.tb136229.x.

Meares, A. (1984). *A way of doctoring*. Melbourne, Australia: Hill of Content.

Meares, A. (1976/1984). *From the quiet place*. Melbourne, Australia: Hill of Content.

Meares, A. (1978/1986). *The wealth within: Self-help through a system of relaxing meditation*. Melbourne, Australia: Hill of Content.

Meares, A. (1987a). *Let's be at ease*. Melbourne, Australia: Greenhouse.

Meares, A. (1988). *The silver years: Practical advice for a rewarding later life*. Melbourne, Australia: Greenhouse.

Meares, A. (1989). *A better life: The guide to meditation*. Melbourne, Australia: Greenhouse.

Meares, A. (1984/1989). "A kind of believing," in *Dialogue on meditation; From the quiet place; A kind of believing* (Melbourne, Australia: Hill of Content).

Meares, A. (1987/1991). *Life without stress: The self-management of stress*. Melbourne, Australia: Penguin.

Seymour, M. (1999). *The teacher's role and the student's experience in Ainslie Meares' approach to Stillness Meditation: Moving beyond technique to create an environment of trust*. Unpublished master's thesis, La Trobe University, Australia.
